# Disease Diagnostics and Potential Coinfections by *Vibrio coralliilyticus* During an Ongoing Coral Disease Outbreak in Florida

**DOI:** 10.3389/fmicb.2020.569354

**Published:** 2020-10-26

**Authors:** Blake Ushijima, Julie L. Meyer, Sharon Thompson, Kelly Pitts, Michael F. Marusich, Jessica Tittl, Elizabeth Weatherup, Jacqueline Reu, Raquel Wetzell, Greta S. Aeby, Claudia C. Häse, Valerie J. Paul

**Affiliations:** ^1^Smithsonian Marine Station, Fort Pierce, FL, United States; ^2^Soil and Water Sciences Department, University of Florida, Gainesville, FL, United States; ^3^mAbDx, Inc., Eugene, OR, United States; ^4^Carlson College of Veterinary Medicine, Oregon State University, Corvallis, OR, United States

**Keywords:** stony coral tissue loss disease, *Vibrio coralliilyticus*, coral disease, coral coinfections, immunoassay, digital droplet PCR

## Abstract

A deadly coral disease outbreak has been devastating the Florida Reef Tract since 2014. This disease, stony coral tissue loss disease (SCTLD), affects at least 22 coral species causing the progressive destruction of tissue. The etiological agents responsible for SCTLD are unidentified, but pathogenic bacteria are suspected. Virulence screens of 400 isolates identified four potentially pathogenic strains of *Vibrio* spp. subsequently identified as *V. coralliilyticus*. Strains of this species are known coral pathogens; however, cultures were unable to consistently elicit tissue loss, suggesting an opportunistic role. Using an improved immunoassay, the VcpA *RapidTest*, a toxic zinc-metalloprotease produced by *V. coralliilyticus* was detected on 22.3% of diseased *Montastraea cavernosa* (*n* = 67) and 23.5% of diseased *Orbicella faveolata* (*n* = 24). VcpA^+^ corals had significantly higher mortality rates and faster disease progression. For VcpA^–^ fragments, 21.6% and 33.3% of *M. cavernosa* and *O. faveolata*, respectively, died within 21 d of observation, while 100% of similarly sized VcpA^+^ fragments of both species died during the same period. Further physiological and genomic analysis found no apparent differences between the Atlantic *V. coralliilyticus* strains cultured here and pathogens from the Indo-Pacific but highlighted the diversity among strains and their immense genetic potential. In all, *V. coralliilyticus* may be causing coinfections that exacerbate existing SCTLD lesions, which could contribute to the intraspecific differences observed between colonies. This study describes potential coinfections contributing to SCTLD virulence as well as diagnostic tools capable of tracking the pathogen involved, which are important contributions to the management and understanding of SCTLD.

## Introduction

Coral reefs currently face a variety of threats, including anthropogenic climate change, pollution, overfishing, and disease. Unfortunately, these threats and documented coral disease outbreaks have increased over time, further damaging important reef ecosystems (Harvell et al., 1999; Porter et al., 2001; Bruno et al., 2007; Maynard et al., 2015). Some of the more destructive outbreaks are the result of tissue loss diseases (sometimes referred to as white syndromes) (Richardson et al., 1998; Aeby, 2005; Sánchez et al., 2015; Aeby et al., 2016; Ushijima et al., 2016; Gignoux-Wolfsohn et al., 2020), which actively destroy the coral tissue loss (i.e., tissue loss) (Work and Aeby, 2006). Coral diseases can directly kill off these ecosystem engineers primarily responsible for reef accretion, often leading to damaging phase-shifts to algal-dominated systems (Aronson and Precht, 2001; Nugues, 2002; Miller et al., 2009).

Currently, Caribbean reefs are being devastated by a multi-year outbreak of stony coral tissue loss disease (SCTLD), which could be considered one of the worst recorded Caribbean coral disease outbreaks (Precht et al., 2016; Muller et al., 2020). SCTLD is unprecedented because of a combination of various attributes including its duration, distribution, and virulence. This outbreak has been ongoing since late 2014, where it was first observed in Florida around Miami-Dade county (Precht et al., 2016). This highly virulent disease has since spread north and south along almost the entirety of the Florida Reef Tract at a rate of approximately 100 m per day (Florida Keys National Marine Sanctuary, 2018; Muller et al., 2020). Unfortunately, by early 2019, SCTLD has spread outside of the Florida Reef Tract and into various Caribbean reefs (Alvarez-Filip et al., 2019). There are at least 20 different coral species susceptible to SCTLD, which includes important reef-builders like *Montastraea cavernosa, Orbicella faveolata*, *Colpophyllia natans*, and *Pseudodiploria strigosa* (Precht et al., 2016; Walton et al., 2018; Aeby et al., 2019; Muller et al., 2020). Interspecific differences in disease susceptibility are observed among coral species (Precht et al., 2016; Florida Keys National Marine Sanctuary, 2018); some species typically display chronic to subacute tissue loss (e.g., *M. cavernosa*) while others have acute tissue loss (e.g., *C. natans*) (Aeby et al., 2019). However, the reason behind these differences, along with intraspecific variability between lesion progression rates and presentation, are currently unknown.

The etiological agent(s) responsible for SCTLD have yet to be identified, but disease is spread through seawater and/or direct contact, suggesting an infectious agent is responsible (Aeby et al., 2019). During previous studies, SCTLD lesions could be arrested using broad-spectrum antibiotics, implying pathogenic bacteria are important for disease progression (Aeby et al., 2019). Additionally, microbial community analysis has identified various sequences belonging to the bacterial orders Flavobacteriales, Clostridiales, Rhodobacterales, Alteromonadales, and Vibrionales enriched at the disease lesions compared to the apparently heathy areas of the same colonies or healthy neighboring colonies (Meyer et al., 2019). A similar study identified sequences belonging to Rhodobacterales and Rhizobiales in disease tissue that matched those in the surrounding sediment, suggesting an environmental reservoir for at least some of these lesion-associated bacteria (Rosales et al., 2020). For both studies, the disease-associated sequences were not found in every disease lesion, and it is still unknown if a single etiological agent is responsible for SCTLD. There are no indications as to what role these bacteria are playing in SCTLD; if they are simply saprophytic colonizers, opportunistic pathogens, or primary causes of disease.

The study presented here began with culturing potential pathogens associated with SCTLD and the discovery of *Vibrio coralliilyticus* on a subset of diseased corals. Strains of *V. coralliilyticus* have been implicated in outbreaks of disease (vibriosis) affecting various coral and shellfish species in the Indo-Pacific and Mediterranean (Ben-Haim and Rosenberg, 2002; Estes et al., 2004; Sussman et al., 2008; Vezzulli et al., 2010; Ushijima et al., 2014a, 2016; Richards et al., 2015), which prompted examination of the extent of the association between this bacterium and corals with SCTLD. Additionally, diseased corals from the field were screened using an improved version of an immunoassay (Gharaibeh et al., 2013; Wilson et al., 2013), the VcpA *RapidTest* (mAbDx, Inc.), to detect a toxic zinc-metalloprotease produced by *V. coralliilyticus*, VcpA, and to identify any correlations between its presence and SCTLD progression. To complement the immunoassay, a quantitative assay was also developed using droplet digital PCR (ddPCR) specific for the gene encoding VcpA. Furthermore, strains of *V. coralliilyticus* cultured from SCTLD lesions were tested directly on healthy corals to determine if they are a primary cause of infection or playing a more secondary role. Lastly, the Atlantic *V. coralliilyticus* strains cultured during this study were compared to pathogenic strains from the Indo-Pacific to identify potential differences in their physiology or genetic potential that may link to virulence. This study adds to the understanding of SCTLD, which has no identified etiological agent(s) or associated diagnostic tools, as well as of *Vibrio coralliilyticus* biology that may be a more extensive threat to corals reefs than previously believed.

## Materials and Methods

### Seawater Source and Storage

Oceanic seawater was used for the bacterial growth media and aquarium experiments. All seawater was collected from an intake pipe extending approximately 1,600 m offshore South Hutchinson Island, St. Lucie County, FL, which was then filtered progressively through a 20, 1.0, 0.5, and 0.35 μm pore filters into storage containers. Seawater in storage was constantly re-circulated through a 20 μm pore filter, a filter canister with ROX 0.8 aquarium carbon (Bulk Reef Supply), and a 36-watt Turbo-twist 12× UV sterilizer (Coralife) in series. Prior to use, all seawater was filtered through a 0.35 and 0.22 μm pore filter in series to remove bacteria. This filtered seawater (FSW) was used as a base for all indicated bacterial growth media and manipulative experiments with corals.

### Bacterial Growth Conditions

All bacteria were grown on a seawater-based medium (Table 1), seawater broth (SWB). SWB was prepared by mixing 4 g/L of tryptone (Fisher Scientific) and 2 g/L of yeast extract (Fisher Scientific) in FSW, which was supplemented with 15 g/L of agar (TekNova) to make seawater agar (SWA). For liquid cultures that needed to be grown and used in the same day, the SWB was supplemented with 2 ml/L of glycerol (Fisher Scientific) prior to autoclaving (GSWB) to support growth. TCBS agar was used for the selective growth of vibrios and was prepared using the manufacturer’s instructions, except it was supplemented with 10 g/L of NaCl to support the growth of marine strains. All cultures were grown at 28°C that included shaking 250 rpm for liquid cultures, unless otherwise stated. At the start of every experiment, required bacterial strains were revived from cryostocks stored at −80°C. A small aliquot of the stock was scraped into fresh SWB or onto SWA using a sterile loop or pipette tip, and then the inoculated media was incubated for 15 h before use.

**TABLE 1:** Bacterial isolates used in this study.

Strains	Description	Citation
*V. coralliilyticus* ATCC BAA-450	Type strain; coral pathogen; isolated from reef near Zanzibar.	Ben-Haim and Rosenberg, 2002
*V. coralliilyticus* OCN008	Coral pathogen; isolated from a reef in Kāne‘ohe Bay, HI, United States.	Ushijima et al., 2014a
*V. coralliilyticus* OCN014	Coral pathogen; isolated from a reef near Palmyra Atoll.	Ushijima et al., 2016
*V. coralliilyticus* RE22	Oyster larvae pathogen; isolated from hatchery at Netarts Bay, OR, United States.	Estes et al., 2004
*V. coralliilyticus* RE98	Oyster larvae pathogen; isolated from hatchery at Netarts Bay, OR, United States.	Estes et al., 2004
*V. coralliilyticus* OfT6-17	From a transmission using diseased *M. cavernosa* from Ft. Lauderdale, FL.	This study
*V. coralliilyticus* OfT6-21	From a transmission using diseased *M. cavernosa* from Ft. Lauderdale, FL.	This study
*V. coralliilyticus* OfT7-21	From a transmission using diseased *M. cavernosa* from Ft. Lauderdale, FL.	This study
*V. coralliilyticus* MmMcT2-4	From a transmission using disease *M. meandrites* from the FL Keys.	This study
*Vibrio* sp. McD22-P3	From a diseased *M. cavernosa* with acute tissue loss collected from the FL Keys; non-coralliilyticus control bacterium.	This study
*Pseudoalteromonas* sp. McH1-7	Non-pathogenic bacterial control isolated from a healthy *M. cavernosa* fragment; control bacterium for infection experiments.	This study
Mutant strains		
OCN008 Δ*fliG*	Aflagellate mutant with a deletion of *fliG2*; flagellum protein; control bacterium for motility experiments.	Ushijima and Häse, 2018
OCN008 Δ*vcpR*	Mutant with a deletion of *vcpR*; quorum sensing regulator; control bacterium for biofilm assays.	Guillemette et al., 2020
OCN008 Δ*vcpAB*	Mutant with deletions of *vcpA* and *vcpB*; zinc-metalloprotease; control bacterium for protease assays and VcpA immunoassays.	Guillemette et al., 2020

### Coral Collections and Husbandry

Both portions of and whole colonies were collected on SCUBA via hammer and chisel from various locations throughout the Florida Keys and in Broward County (near Ft. Lauderdale), FL (Supplementary File S1) under appropriate state and federal permits. Individuals selected for collection at each location were at least 5 m apart to avoid collecting colonies of the same genotype. Colonies were transported to dive vessels in sealed 18.9 L plastic bags filled with ocean water. Apparently healthy corals were obtained from the NOAA Key West Nursery and cared for at the Smithsonian Marine Station (SMS) facilities in Fort Pierce, FL (see below) before use in experiments. Additional healthy corals were collected during the January 2020 collection cruise outside of the Dry Tortugas National Park organized and supported by the Florida Department of Environmental Protection Office of Resilience and Coastal Protection-Southeast Region (FL-DEP), which was considered outside of the SCTLD endemic regions at the time of collection.

All corals collected from the field or from a nursery were transported to the Smithsonian Marine Station (SMS) facility where all manipulative experiments took place. For transport, corals were wrapped in plastic bubble wrap moistened with seawater and then placed in a cooler. After arrival, corals were gently rinsed with FSW. Diseased corals were kept in buckets containing approximately 13 L of FSW and a weighted airline to aerate and create water motion. The buckets were kept in a larger, insulated water tables filled with freshwater (approximately 570 L) on a recirculating system with 800 w heaters (Aquatop) and a 1 hp chiller (AquaEuroUSA) to maintain temperature. The water temperature was initially set at the same temperature the corals were in during collection and then slowly adjusted to 28°C (a maximum 0.5°C change per day) before use in experiments. Tables holding diseased corals were outside in a quarantine area under ambient light conditions underneath a clear plastic canopy and one layer of shade cloth that blocked approximately 50% of the light. Each table was also individually covered with clear plastic and one layer of the same shade cloth to achieve a light level between 200 and 500 μmol photons m^–2^ sec^–1^ depending on the time of day and weather. Partial water changes (approximately 50% water exchange) were conducted at different intervals depending on the experiment (see below). Sterilized plastic scoops were used for all water changes. Scoops were rinsed and scrubbed in a 10% calcium hypochlorite solution, rinsed with freshwater several times, and then left to dry for at least 24 h before each use.

Apparently healthy corals were kept in an indoor facility within multiple large recirculating systems each holding approximately 570 L of FSW. The FSW was recirculated through a sump with chillers and heaters to maintain a temperature of 25.5°C ± 0.3°C. Before being pumped back into the holding tank the seawater was passed through a UV sterilizer (same model described above). Within each table, there were two circulation pumps (AquaTop MaxFlow MCP-5) to create water motion and a row of six blue-white 30 cm^2^ LED panels (HQPR) above each table providing 150 to 250 μmol photons m^–2^ sec^–1^ for captive corals. Targeted feeding of healthy corals occurred three times weekly alternating between a mix of Reef Roids (PolypLab), Marine Snow (Two Little Fishes), and LPS or SPS Max (Dr. G’s Marine Aquaculture) according to each manufacturer’s recommendations.

Healthy and diseased corals were fragmented with a masonry saw (Husqvarna MS 360) fitted with a 35.56 cm diamond, continuous rim, circular saw blade. FSW was constantly sprayed over the blade for cooling, and corals were covered with plastic bubble wrap during cutting to reduce unnecessary tissue damage. The system was cleaned with 70% ethanol and flushed with freshwater between cutting sessions.

### Sampling and Isolation of Coral-Associated Bacteria

Only diseased corals that were infected in the laboratory from transmission experiments were sampled to culture potential pathogens. Healthy corals were placed into direct contact with the lesions of diseased corals that were set up and cared for in the same manner as a previously described study (Aeby et al., 2019). Once a fragment was suspected to be infected, it was removed from contact with the diseased fragment and monitored for 24 to 48 h to ensure disease progression. Samples were taken directly from the progressing lesion and polyps directly adjacent to the lesion. All tanks were maintained at 28°C with partial water changes every other day.

To isolate bacteria from disease lesions, the tip of a sterile 30 ml syringe was used to agitate an area of the coral while at the same time drawing in any mucus and tissue. The mucus/tissue samples were transferred to sterile conicals and vortexed vigorously for 2–3 min before being serially diluted in autoclaved FSW. After dilution, 50 μl aliquots were spread onto SWA with sterile glass beads and then incubated at 28°C for 72 h. Following incubation, 25–100 colonies per coral fragment were picked and streaked out on SWA, depending on diversity. Colonies were selected based on colony morphology to maximize diversity. Isolates were streaked out 2–3 times to ensure purity. Once an axenic culture appeared to be achieved, the isolates were grown in SWB for 24–48 h and cryopreserved with 30% glycerol (final concentration) at −80°C.

### Coral Inoculation Experiments

To test if isolates from diseased corals could elicit disease signs, apparently healthy coral fragments were exposed to batches of isolates according to a previously described experimental design (Ushijima et al., 2012, 2016). Healthy *M. cavernosa*, *C. natans*, *O. faveolata*, and were provided by the NOAA Key West Coral Nursery. Healthy *Meandrina meandrites* were collected near Big Pine Key, FL by Florida Fish and Wildlife Research Institute. All apparently healthy corals were used as hosts for virulence screens. Corals were cut using a masonry saw, as described above, into approximately 3 to 4 cm^2^ fragments. The cut corals were allowed to recover for at least 24 h before use in any experiments. For the initial screens for virulence, 3 to 4 fragments of apparently healthy corals were placed into 5 L of FSW using a previously described aquarium set up (Aeby et al., 2019). For all inoculation experiments with potential pathogens, the water temperature was maintained at 28°C, and after 5 days post-inoculation, partial FSW exchanges using sterilized scoops were conducted every other day. All tanks were maintained in the outside facility using the water sources and conditions described above.

To prepare the preserved isolates for virulence screens, strains were revived from −80°C by streaking them out on SWA to check both viability and purity. After a 48-h incubation, 3–4 single colonies of each isolate were used to inoculate 10 ml of GSWB. Cultures were grown with aeration to an optical density measured at 600 nm (OD_600nm_) of approximately 0.8 (equal to about 10^9^–10^10^ CFU/ml) measured using a 180 UV-Vis Spectrophotometer (Genesys). After incubation, the cultures were centrifuged at 5,000 x g for 12 min and then the supernatant was carefully decanted. The cultures were then organized into groups of 5 isolates, based upon their previously assigned ID codes and the time when they reached their appropriate OD_600nm_. Each group of five was then inoculated into a single tank of coral, one isolate at a time. For inoculation, each bacterial pellet was mixed with 1 ml of FSW from the intended tank by gentle pipetting, and then the suspension was gently pipetted directly over each coral fragment. The inoculum for each tank (5 x 10 ml cultures) was evenly distributed between all the fragments within the tank. The bubbling in the tank was then left off for 2 h. Each fragment was photographed daily and monitored for disease signs. If tissue loss was observed, the fragment was monitored to ensure tissue loss progressed but was removed after more than approximately 50% of the fragment was diseased. If a group of isolates was able to initiate disease signs, then all five isolates were tested individually along with the 5-isolate group. To test individual isolates, a 100 ml SWB culture was grown and then a 50 ml aliquot was centrifuged to be used to inoculate a single tank. If an isolate was seemingly able to induce disease in two or more coral fragments, then it progressed to the next stage of testing.

Once potentially pathogenic isolates were identified, virulence was evaluated in a controlled, previously described experimental design (Ushijima et al., 2012, 2016). For every replicate (block), a single coral fragment was used per tank and all fragments within the block originated from the same coral colony to control for intraspecific differences or their pre-existing microflora. One coral served as a negative control that was exposed to FSW, one was a bacterial control that was inoculated with the non-pathogenic strain McH1-7 (Table 1), and the remaining tanks were inoculated with a potential pathogen (Table 2 and Supplementary File S2). Non-pathogenic strain McH1-7 was isolated from a healthy *M. cavernosa* maintained at the Smithsonian Marine Station. Bacterial growth, preparation, and inoculation were carried out in an identical manner as described above. The apparently healthy corals used for the inoculation experiments were not initially screened for VcpA because the identity of the *Vibrio* isolates was determined after the completion of these experiments. However, the coral genotypes used for the controlled inoculation experiments (i.e., experiments with non-inoculated control fragments) originated from the NOAA Key West Nursery years before SCTLD reached Key West, and these coral colonies were kept in the closed aquarium systems at the SMS and not collected directly from reefs affected by SCTLD.

**TABLE 2:** *V. coralliilyticus* virulence experiments.

Strain ID	Isolated from (transmission type)	Virulence screens (diseased/total corals)	Screens incubation period range	Controlled experiments (diseased/total corals)	Incubation period range
OfT6-17	MCAV → OFAV	3/4 MCAV; 1/10 OFAV	8–10 days; 6 days	0/4 MCAV	N/A
OfT6-21	MCAV → OFAV	1/5 MCAV; 8/10 OFAV	9 days; 3–8 days	1/7 MCAV; 0/8 OFAV; 1/3 MMEA; 0/2 CNAT	8 days; N/A; 12 days; N/A
OfT7-21	MCAV → OFAV	0/6 MCAV; 4/12 OFAV	N/A; 3–9 days		
MmMcT2-4	MMEAN → MCAV	1/3 MMEA; 0/2 MCAV; 0/2 OFAV	2 days; N/A; N/A	0/3 MMEA	N/A

*MCAV = M. cavernosa; OFAV = O. faveolata; MMEA = M. meandrites; CNAT = C. natans. N/A = Not applicable*

To observe the effect of specific bacterial strains on the feeding behavior of *M. cavernosa*, cultures were mixed with a commercial coral food, fed to fragments, and then behavior recorded. Each bacterial strain was grown to an OD_600nm_ of 0.8 as described above. After centrifuging 50 ml of culture into a pellet and decanting the supernatant, 1 ml of FSW and 50 mg of the coral food Reef Roids (PolypLab Inc.) was added and gently mixed via pipetting. The mixture was incubated at 28°C for 2 h before being inoculated onto a single coral fragment in 5 L of FSW. The air was kept off during the hour monitoring/recording period. Coral fragments used in this study were starved for 7 days prior to the experiment. For experiments without food, the culture was inoculated onto the coral fragment as described above.

### Immunochemical Detection of *Vibrio coralliilyticus* protease A (VcpA)

VcpA was detected with the VcpA *RapidTest* (mAbDx, Inc.), an improved and simplified version of a 2-site immunoassay for VcpA previously described by Gharaibeh et al. (2013) (Figure 2 and Supplementary File S3). The VcpA immunoassay was previously developed for isolates believed to be *Vibrio tubiashii* in Gharaibeh et al. (2013). However, the isolates used in the immunoassay development, and additional strains of alleged *V. tubiashii*, have since been reclassified as *V. coralliilyticus* (Wilson et al., 2013; Richards et al., 2015). Modifications to improve sensitivity and incorporate the test into robust single-use cassettes suitable for both laboratory and field use are described in detail in Supplementary File S4. Analytic performance characteristics and user protocols are described in Supplementary File S5.

### VcpA Screening and Lesion Monitoring

Diseased coral fragments were individually housed in tanks with 5 L of FSW. Diseased fragments could not be cut to identical dimensions because of limitations on coral and lesion size (Supplementary File S1). Each fragment was photographed daily with a partial water change every other day, with special care taken to not cross-contaminate tanks with chronic or sub-acute lesions with those with apparent acute lesions. Fragments were not tested for VcpA immediately after transport to reduce the risk of false positives from contamination, only after they were separated into their own tanks. For testing, a sterile needleless syringe was used to syringe up approximately 10 ml of disease tissue and mucus at the lesion front. The sample was then transferred to a sterile conical. The sample was vortexed for 2 to 3 min and added in 50 μl aliquots (150–250 μl total) to the sample well of a VcpA *RapidTest* cassette (Figure 2 and Supplementary File S4). After all the sample entered the cassette, a few drops (∼100 μl) of dH_2_O water was added to the sample well and was allowed to flow through to clear the test viewing window. Results were then scored visually as plus/minus, using control positive samples (15 h *V. coralliilyticus* cultures diluted 1:100 in FSW) and control negative samples (sterile seawater lacking VcpA) as reference tests. All tests were read within 20 min of starting the test. A duplicate test was run for every sample except where noted.

### Detection of *vcpA* Using Digital Droplet PCR

Bacterial isolates were grown in SWB at room temperature in a shaking incubator (200 rpm) until turbid. Cells were pelleted from 1.5 ml of culture and DNA was extracted from the pellet with a DNeasy PowerSoil Kit (Qiagen). After inconsistent results with previously published *vcpA* primers (Gharaibeh et al., 2009), new PCR primers were designed for the 1,824-bp zinc-metalloprotease gene (vibriolysin-like protein). Standard end-point PCR was performed in 25-μl reactions with One*Taq* 2× Master Mix (New England Biolabs) and 0.5 μM concentrations each of the vibriolysin F primer (5′- GGCGAACCAACTTTACTGGA-3′) and vibriolysin R primer (5′- GGTCAGTCACTGGCGTACCT-3′). Following an initial denaturation at 94°C for 3 min, thermocycling proceeded with 35 cycles of 94°C for 30 sec, 60°C for 30 sec, and 72°C for 30 sec, followed by a final extension of 72°C for 3 min. The 197-bp amplification product was confirmed by visualization on a 1.5% agarose gel. Strong amplification of this product was confirmed with Atlantic-based *V. coralliilyticus* strains OfT6-17, OfT6-21, OfT7-21, MCA25, and MCA32, as well as Pacific-based *V. coralliilyticus* strains RE22 and OCN014. Target specificity was confirmed by Sanger sequencing of the amplification product for strain OfT6-17. Melt curve analysis was performed after quantitative PCR amplification on a StepOnePlus real-time PCR system (Applied Biosystems) with the following thermocycling conditions: initial denaturation at 95°C for 20 sec, followed by 40 cycles of 95°C for 3 sec and 60°C for 30 sec and a melt curve stage of 95°C for 15 sec, 60°C for 1 min, and 95°C for 15 sec. Droplet digital PCR was performed at the University of Florida Interdisciplinary Center for Biotechnology Research with an annealing temperature of 60°C and 2 ng of input DNA per reaction.

### Colony PCR

To rapidly screen isolates for the presence of the *V. coralliilyticus* genes *vcpA* and *vcpR*, a quick-lysis and culture/colony PCR protocol was used. An isolated colony or 10 μl of an overnight SWB culture was added to 100 μl of ice-cold sterile PCR-grade water kept on ice in a 1.5 ml microcentrifuge tube. The tube was mixed on a vortex for 1 min, secured in a bubble rack, and placed into water at a rolling boil for 5 min. After boiling, the tubes were chilled on ice before being used as template for PCR. For PCR, 1 μl of the chilled template was used in a 20 μl reaction using MangoMix (Bioline) and the manufacturer’s recommended protocols. The primer pairs vibriolysin F and vibriolysin R (see above) and vcpR-int-F (5’-ATTGCAGAAATCGCTCAGGT-3’) and vcpR-int-R (5’-ACCATTGCCGAAGTTAGGTT-3’) amplify a 197 bp product for *vcpA* and a 179 bp product for *vcpR*, respectively. The products were visualized on a 1% agarose gel using SYBR Safe gel stain (ThermoFisher Scientific) with the manufacturer’s recommended protocol.

### Bacterial Physiological Tests

It was unclear if the *V. coralliilyticus* strains from Florida were defective in any specific virulence-related traits compared to known pathogenic isolates from other regions. So physiological tests compared characteristics between the *V. coralliilyticus* strains that commonly relate to virulence for a variety of bacterial pathogens. All statistical tests were run, and graphs were created with Graphpad Prism (ver. 8.4.2). Before each experiment, strains (Table 1) were revived from the −80°C cryostock by streaking them onto SWA and then incubating them for 15 h before use.

Antibiotic resistance could prevent effective disease treatment, so resistance to antibiotics effective against SCTLD was a pertinent first test for these *V. coralliilyticus* isolates (Aeby et al., 2019; Neely et al., 2020). Antibiotic susceptibility tests were conducted in 96-well plates. Antibiotic stocks (1000×) were diluted in SWB and 100 μl was aliquoted per well (*n* = 3 for each dilution series). The blanks for each plate was SWB and the antibiotic stock used. SWB without antibiotics served as a control. For inoculation, a 15-h old SWB culture was diluted 1:100 in FSW, then 0.5 μl was inoculated into each well. The plates were then incubated in a humidified incubator at 28°C for 24 h, before the OD_600nm_ was measured using a plate reader. For all OD_600nm_ readings, the corresponding blank measurement was first subtracted before any data analysis. The minimum inhibitory concentration (MIC) was estimated based on the growth of each strain at the different concentrations of each antibiotic. Complete inhibition was considered to be an OD_600nm_ below 0.1 after 24 h of growth.

Any intraspecific differences in growth rates could attribute to differences in virulence, therefore growth rates were compared among strains. For growth experiments, 2–3 isolated colonies were picked to inoculate a 2 ml SWB starter culture that was incubated for 15 h. Then 0.5 μl of the starter culture was used to inoculate a 96-well plate previously filled with 100 μl of SWB per well. For salinity experiments, the total salinity of SWB (initially at 35 ppt) was reduced using deionized water and measured using a refractometer before being autoclaved. If growth was going to be measured at a specific salinity, the starter culture was grown at the corresponding salinity. For each strain, 6 replicates were set up per plate, along with wells with uninoculated media for blanks. Plates were then incubated in an Incu-Shaker 10LR (Benchmark Scientific) incubator under a consistent temperature ( ± 0.5°C) at 100 rpm. The optical density was measured at 600 nm (OD_600nm_) using an Epoch R Microplate Spectrophotometer (BioTek) at specific time points and then immediately returned to incubation. The mean OD_600nm_ measurements were graphed over time, and then the slope during logarithmic growth was calculated and compared between conditions and strains using a repeated measrues ANOVA on strain and salinity with a post-hoc Tukey’s multiple comparisons test. To compare the effect of temperature (23 versus 29°C) on the logarithmic growth for each individual strain, a linear regression line was generated for time points 3 to 8 h post-inoculation for the data sets gathered (logarithmic growth phase). Then using a built-in linear regression analysis, the differences between the slopes were compared.

Swarming motility, movement across a solid or semisolid surface, could contribute to pathogen movement on or within a coral. Therefore, to evaluate apparent swarming motility, 2–3 colonies from an overnight plate were touched with a sterile toothpick and stabbed halfway through a thick SWA plate (40 ml). For SWA with different salinities, they were adjusted with deionized water as described above before autoclaving. The agar concentration of the media was 1.5% (TekNova), analogous to other studies on *Vibrio* swarming (McCarter, 1998). After inoculation, plates were incubated for 4 days and the radius (from the point of the initial stab to the edge of growth) was measured every 24 h using a caliper. The mean swarming radii at each salinity was compared separately using a two-way repeated measured ANOVA based on strain and time with a post-hoc Tukey’s multiple comparisons test. Additionally, the more relevant effects of salinity and strain on swarming ability were analyzed using measurement from the day 4 timepoint in a two-way ANOVA and a Tukey’s multiple comparisons test. To check for swimming motility, 1 μl of a 15-h old SWB culture was added to 10 μl of autoclaved FSW on a hanging drop slide. Swimming motility was confirmed using light microscopy.

Protease activity has been linked to virulence in *V. coralliilyticus*, specifically the toxic metalloprotease VcpA (Ben-Haim et al., 2003; Hasegawa et al., 2008; Sussman et al., 2009). To measure protease activity, azocasein assays were carried out using a protocol adapted from previous studies (Charney and Tomarelli, 1947; Hasegawa et al., 2008). Using an overnight SWB culture of each strain, 50 ml of GSWB was inoculated with 100 μl of the starter culture and incubated. The culture density was monitored using a spectrophotometer until reaching an OD_600nm_ of approximately 0.8, equivalent to early logarithmic growth phase. After reaching the appropriate density, 100 μl of culture was removed and the remaining cultures returned to the incubator. The culture aliquot was mixed with 200 μl of 0.5% sodium bicarbonate buffer (pH 8.3) and 200 μl of 2.0% azocasein in 0.5% sodium bicarbonate buffer (pH 8.3) in a 1.5 ml microcentrifuge tube. The contents were vortexed and then incubated at 30°C for 10 min. After incubation, 500 μl of 20% trichloroacetic acid was added, vortexed, and then incubated at room temperature (23°C) for 5 min. The tubes were then centrifuged at 10,000 x g for 5 min at 4°C. Being careful to not disturb the pellet, 500 μl of the supernatant was added to 1 mL of 1 M NaOH and absorbance was read at 440 nm. This process was repeated after the cultures had been incubating for 15 h and reached an OD_600nm_ of approximately 3.0, representing stationary phase. The readings at 440 nm were standardized to culture density and presented as a ratio of OD_440nm_ to OD_600nm_. A mutant, OCN008Δ*vcpAB*, with clean deletions of the major *V. coralliilyticus* proteases, genes *vcpA* and *vcpB*, was used as a negative control (Guillemette et al., 2020). The mean OD_400nm_/OD_600nm_ ratio for each strain at logarithmic and stationary phase was compared at every time point using a 2-wayANOVA based on strain and time with a post-hoc Tukey’s multiple comparisons test.

For other pathogenic vibrios like *V. cholera* or *V. parahaemolyticus*, biofilm production has been linked to virulence or host colonization (Silva and Benitez, 2016; Aagesen et al., 2018). To measure extracellular polysaccharide (EPS) production, indicative of biofilm formation, a modified crystal violet protocol was used based on previous studies (O’Toole, 2011; Guillemette et al., 2020). For each strain, an overnight SWB culture was diluted 1:1000 in fresh SWB, and then 1 mL aliquots of the diluted cultures were transferred to a 24-well plate (*n* = 4 for each strain). The plates were then incubated in a humidified incubator at 28°C for 48 h. After incubation, the cultures were aspirated from the wells and the wells were washed 3 times with 1 mL of sterile FSW. After washing, 1 ml of 0.1% crystal violet solution was added and then incubated at room temperature for 15 min. After incubation, the crystal violet solution was aspirated off and then the wells were washed 3 times with 1 ml aliquots of FSW. The plates were then dried overnight with their lids off and inverted. When each well was completely dry, 1 ml of 30% acetic acid solution was added into each well and then incubated at room temperature for 15 min. After incubation, 500 μl of solution from each well was transferred to a new 24-well plate and the absorbance read at 550 nm using a plate reader. A mutant, OCN008Δ*vcpR*, with a deletion of the regulator *vcpR* and upregulated EPS production was used as a positive control (Guillemette et al., 2020). The mean OD_550nm_ for each strain was compared using a one-way ANOVA with a post-hoc Tukey’s multiple comparisons test.

Standard biochemical and carbon utilization tests were run for each strain using the API 20E (BioMérieux) test strip assays to further identify any other potential differences that may contribute to strain differences. These assays are generally used to differentiate between different species of bacteria and can detect physiological differences between strains. The manufacturer’s instructions were followed with the following modifications. Each bacterial stock was streaked out from their cryostock on SWA and incubated for 15 h at 28°C. Then 2–3 colonies from each strain was resuspended in sterile 3% (w/v) NaCl solution instead of the API media. The manufacturer’s protocol was then followed, strips were incubated at 28°C in a humidified incubator for 24 h, and then read using the provided key and directions for additional reagents.

### Data Analysis

All data analysis for the survival curves, disease progression, and bacterial characterization experiments was done in Graphpad Prism (ver. 8.4.2). For all statistical tests used, *n*-values and *p*-values are reported with the results. For the survival analysis outcomes (Supplementary File S1), fragments were categorized as complete mortality, slow progression, or stopped. A fragment was considered to have complete mortality when no apparently living tissue was left. For fragments that did not die during the 21-day monitoring period but had progressing lesions based on their photo analysis (see below), they were considered to have slow progression. These fragments were considered alive in the survival analysis. Fragments with disease progression that arrested during the 21-day monitoring period and did not progress any further were considered to have stopped progression. However, fragments with lesions that stopped and then began to progress again were considered to have slow progression.

Initial measurements for disease progression were quantified using the daily photographs and ImageJ (NIH). The grating beneath the fragments served as a set scale; each square is 1.5 cm^2^. The total area of apparently healthy tissue of each fragment was measured at the start of the experiment (day 0), 24 h post, and then every other day after that. If a fragment had complete mortality, the photograph from the previous day was also measured and included in the analysis. Each measurement was divided by the day 0 measurement and multiplied by 100% to calculate the percent remaining healthy tissue at each time point to standardize the data for the unequal sizes of the corals. The percent remaining healthy tissue was then plotted over time in Prism. The area under the curve (AUC) was calculated for each fragment, with a lower AUC value corresponding to faster disease progression. All statistical tests, graphing, and data organization was done in Graphpad Prism for these experiments using the built-in functions.

### Bacterial Genome Sequencing and Analysis

Vibrios have high genomic flexibility that has allowed them to adapt to a wide range of niches and this ecological specialization may not be reflected in marker genes such as 16S rRNA genes (Hunt et al., 2008; Thompson et al., 2009). Therefore, we sequenced the genomes of the first *V. coralliilyticus* isolates from the Atlantic for comparison to previously described coral pathogens from the Pacific and Indian Oceans. Eight newly isolated strains of *V. coralliilyticus* from both diseased and healthy corals (Table 1) were grown in SWB at room temperature in a shaking incubator (200 rpm) until turbid. Cells were pelleted from 1.5 ml of culture and DNA was extracted from the pellet with a DNeasy PowerSoil Kit (Qiagen). Libraries for whole genome sequencing were produced with a Nextera FLEX kit (Illumina) using up to 9 ng of input DNA, as quantified by TapeStation (Agilant). Libraries were sent to the University of Florida Interdisciplinary Center for Biotechnology Research and sequenced on an Illumina MiSeq with the 2 x 150 bp v. 2 cycle format. Sequencing reads were quality-filtered with the Minoche (Minoche et al., 2011) filtering pipeline in illumina-utils v. 2.3 (Eren et al., 2013), and Illumina adapters and Nextera transposase sequences were removed with cutadapt v. 1.8.1 (Martin, 2011). Genomes were assembled with SPAdes v 3.13.0 (Nurk et al., 2013). Average nucleotide identity was determined with the Average Nucleotide Identity calculator from the enveomics toolbox (Rodriguez-R and Konstantinidis, 2016). Genome content of the eight new isolates of *V. coralliilyticus* from the Atlantic were compared to the type strain ATCC BAA-450 from the Indian Ocean (Kimes et al., 2011) and Indo-Pacific strains RE22 (Richards et al., 2018), RE98 (Richards et al., 2014), P1 (Santos Ede et al., 2011), OCN008 (Ushijima et al., 2013), and OCN014 (Ushijima et al., 2014b). These 14 strains of *V. coralliilyticus* were annotated with Prokka v. 1.12 (Seemann, 2014) and pangenome analysis was completed with Roary v. 3.12.0 (Page et al., 2015). An approximate maximum-likelihood phylogenetic tree of the 14 genomes was created from the alignment of core genes with FastTree v. 2.1.7 (Price et al., 2010) and plotted with Phadango v. 1.3.0 (Hadfield et al., 2018). Virulence factors in strains of *V. coralliilyticus* were compared to other *Vibrio* pathogens with the Virulence Factor Database 2019 VFanalyzer (Liu et al., 2019), including the human pathogens *V. cholerae* O1 biovar El Tor strain N16961, *V. parahaemolyticus* strain RIMD2210633, and *V. vulnificus* strain CMCP6 present within the database. In addition to the 14 genomes of *V. coralliilyticus*, the genomes of other *Vibrio* pathogens of corals, including *V. proteolyticus* strain NBRC 13287 (Merkel et al., 1964), *V. shilonii* strain AK1 (Kushmaro et al., 1997), and *V. shiloi* strain A203 (Moreira et al., 2014) were uploaded to the VFanalyzer. Secondary metabolites for all 14 genomes of *V. coralliilyticus* and the other six *Vibrio* species were identified with the bacterial version of AntiSMASH v. 5.0 (Blin et al., 2019). Raw sequencing reads and assembled genomes are publicly available from the National Center for Biotechnology Information under BioProject PRJNA625269. The genome of strain MmMcT2-4 was too incomplete for acceptance into GenBank and is provided as a supplemental file (Supplementary File S10).

## Results

### Potentially Pathogenic Vibrios From Infected Corals Identified as *V. coralliilyticus*

Culturing potential pathogens on SWA plates led to a total of 400 isolates from 8 different diseased coral fragments. The bacterial isolates were organized into 80 groups each consisting of 5 isolates, of which, 8 groups elicited tissue loss or bleaching when screened against healthy corals. After additional screens with the individual isolates from these 8 groups against healthy corals, 13 individual isolates elicited tissue loss in some replicates but not consistently. Based on their 16S rRNA gene sequences, 4 of the 13 isolates, OfT6-17, OfT6-21, OfT7-21, and MmMcT2-4, were identified as belonging to Vibrionaceae (Table 2; Supplementary File S2). The other remaining 9 isolates were unable to reproduce disease signs during controlled infection experiments, thus they were not further pursued as pathogens causing SCTLD. All 4 *Vibrio* isolates (from 3 different infected corals) were confirmed to be *V. coralliilyticus* using the immunoassay specific to the VcpA protease produced by this species (described below), as well as endpoint PCR using primers specific to the *V. coralliilyticus* genes *vcpA* and *vcpR*. This species has multiple strains demonstrated to be pathogenic to coral (Ben-Haim and Rosenberg, 2002; Sussman et al., 2008; Vezzulli et al., 2010; Ushijima et al., 2014a, 2016) and a rapid immunoassay was already available for this bacterium (Gharaibeh et al., 2013), so additional experiments continued with these 4 isolates.

### Screening and Detection of the *V. coralliilyticus* Metalloprotease VcpA on Diseased Corals

**FIGURE 1:**
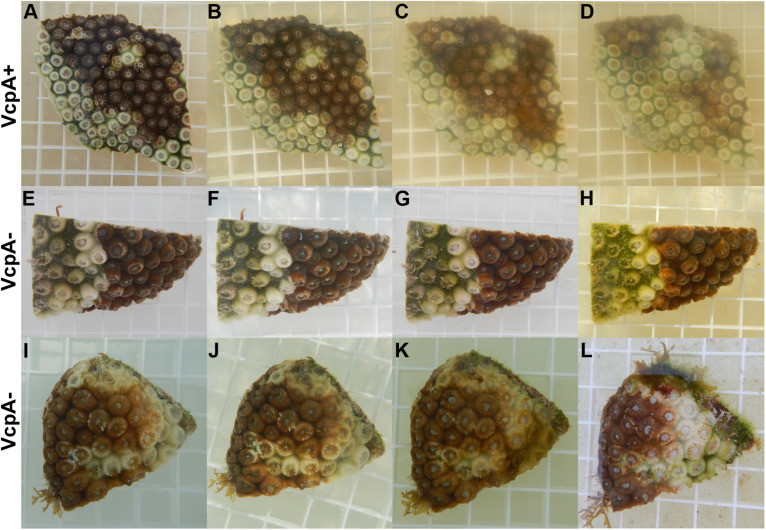
Photos of various disease lesions on *M. cavernosa*. Photos of VcpA^+^ genotype McD-8 with tissue loss on **(A)** day 0, **(B)** day 1, **(C)** day 3, and **(D)** day 4 of observation. Photos of VcpA^–^ genotype McD-23 with tissue loss on **(E)** day 0, **(F)** day 1, **(G)** day 4, and **(H)** day 16 (with some tissue discoloration). Photos of VcpA- genotype McD-35 with tissue loss and bleaching on **(I)** day 0, **(J)** day 1, **(K)** day 4, and **(L)** day 16. The plastic grating in each figure represent 1.5 cm × 1.5 cm squares.

**FIGURE 2:**
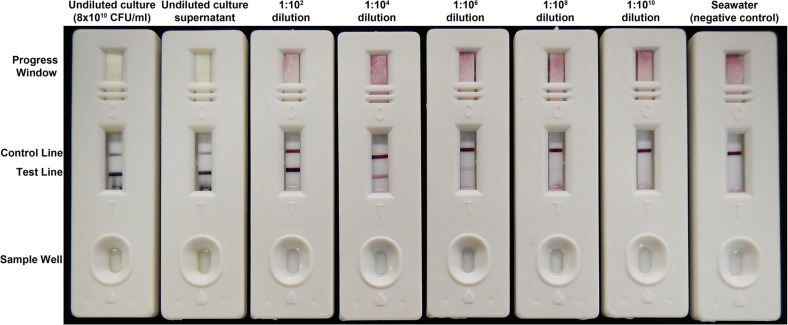
The semiquantitative VcpA *RapidTest*. A dilution series with a *V. coralliilyticus* strain RE22 culture grown to OD_600nm_ 2.0 (approximately 8 × 10^10^ CFU/ml). For this series, 150 μl of sample was loaded into the sample well and left to run for 15 min. The appearance and intensity of the test line equates to the presence and concentration of VcpA. The control line indicates the assay is running properly. The progress window allows the user to see if the sample has diffused through the entire test strip. The dilutions are with the cell-free culture supernatant diluted in sterile FSW and the negative control is FSW.

*Vibrio coralliilyticus* was cultured from 3 of 8 infected coral fragments, however, it was unclear how extensive the relationship of this species was with SCTLD lesions. Therefore, diseased corals (Figure 1) were screened with a new immunoassay specific to *V. coralliilyticus*, the VcpA *RapidTest* (mAbDx, Inc.) (Figure 2 and Supplementary Files S3 S4).

Using the improved VcpA *RapidTest* assay, the presence of VcpA was screened for on incoming diseased corals (Supplementary File S1). Most of the diseased corals were *M. cavernosa* (*n* = 67), but other species included *O. faveolata* (*n* = 24), *C. natans* (*n* = 7), and *P. strigosa* (*n* = 4). For *M. cavernosa*, 16.6% of colonies originated from the Ft. Lauderdale site (*n* = 24) and 27.9% of colonies from Florida Keys sites (*n* = 43) were positive for VcpA (mean 22.25%) (Figures 1A–D and Supplementary File S2). For *O. faveolata*, four colonies originated from the Ft. Lauderdale area and 20 were from the Florida Keys, with 25% and 22% positive for VcpA, respectively (mean 23.5%). The seven *C. natans* and four *P. strigosa* originated from the Florida Keys, but all the samples from these 2 species tested negative for VcpA. Additionally, all apparently healthy colonies originally from the NOAA Key West Nursery, *M. cavernosa* (*n* = 8), *C. natans* (*n* = 5), and *O. faveolata* (*n* = 7), cared for at the Smithsonian Marine Station facility tested negative for VcpA immunoreactivity. In addition, apparently healthy coral colonies collected near the Dry Tortugas National Park for separate experiments also all tested negative for VcpA. The healthy corals collected during the FL-DEP cruise consisted of *M. cavernosa* (*n* = 8), *O. faveolata* (*n* = 3), *C. natans* (*n* = 3), *P. strigosa* (*n* = 3), and *Porites astreoides* (*n* = 2).

To confirm and quantify the presence of *V. coralliilyticus* and VcpA on diseased corals, droplet digital PCR (ddPCR) was utilized to enumerate copies of *vcpA* within coral samples screened with the VcpA *RapidTest*. Samples from 12 diseased *M. cavernosa* fragments from the Florida Keys (Table 3 and Supplementary File S1) were selected for ddPCR analysis, which had samples frozen at −80°C immediately after the *RapidTest* screening. Samples positive for VcpA had between 83 and 922 copies of *vcpA* per ng of total DNA, while VcpA negative samples had between 0 and 11 copies (Table 3). Overall, there were significantly more copies of *vcpA* in VcpA^+^ samples (mean = 187, *n* = 7) compared to VcpA^–^ samples (mean = 3.2, *n* = 5; Mann-Whitney test, *p* = 0.013) (Table 3). These results suggest that approximately 20% of diseased *M. cavernosa* and *O. faveolata* are colonized by populations of *V. coralliilyticus* that are producing the VcpA metalloprotease.

**TABLE 3:** Digital PCR detection of *vcpA* in comparison to VcpA *RapidTest* results.

Coral ID	VcpA *RapidTest* result	copies of *vcpA*/ng DNA	Fragment size (cm^2^)	Tissue loss?	Bleaching?	Outcome	Days until outcome
McD-1	+	159	20	+	−	Complete mortality	12
McD-2	+	119	25	+	−	Complete mortality	19
McD-3	−	11	25	+	−	Disease stopped	5
McD-4	+	922	10	+	+	Complete mortality	8
McD-5	−	3	10	−	+	Disease stopped	3
McD-6	−	1	6	+	+	Disease stopped	9
McD-7	+	285	18	+	−	Complete mortality	1.5
McD-8	+	155	40	+	−	Complete mortality	3
McD-29	−	0	15	+	+	Slow progression	21*
McD-33	+	83	20	+	−	Complete mortality	11
McD-35	−	1	16	+	+	Slow progression	21*
McD-36	+	132	24	+	−	Complete mortality	21

*^∗^Disease lesion progressed over the 21-day monitoring period, but the coral did not completely die during this period.*

### Corals Positive for *V. coralliilyticus* Have Higher Rates of Mortality and Disease Progression

**FIGURE 3:**
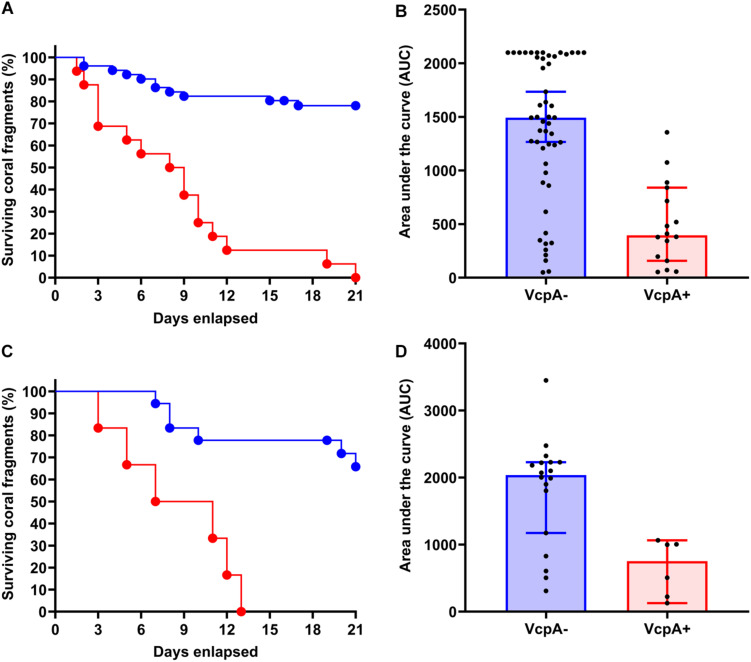
Comparison of survival rates and disease progression of diseased *M. cavernosa* and *O. faveolata* testing positive and negative for the toxic protein VcpA. **(A)** Kaplan-Meyer survival curves of diseased *M. cavernosa* separated by VcpA assay result. **(B)** Area under the curve (AUC) calculation of disease progression for *M. cavernosa* separated by VcpA assay result. **(C)** Kaplan-Meyer survival curves of diseased *O. faveolata* separated by VcpA assay result. **(D)** Area under the curve (AUC) calculation of disease progression for *O. faveolata* separated by VcpA assay result. Corals negative for VcpA are represented by blue lines with circles or blue bars. Corals positive for VcpA are represented by red lines with circles or red bars. Bars on the AUC analysis represents the median of the sample set and the error bars represent the 90% confidence interval.

**FIGURE 4:**
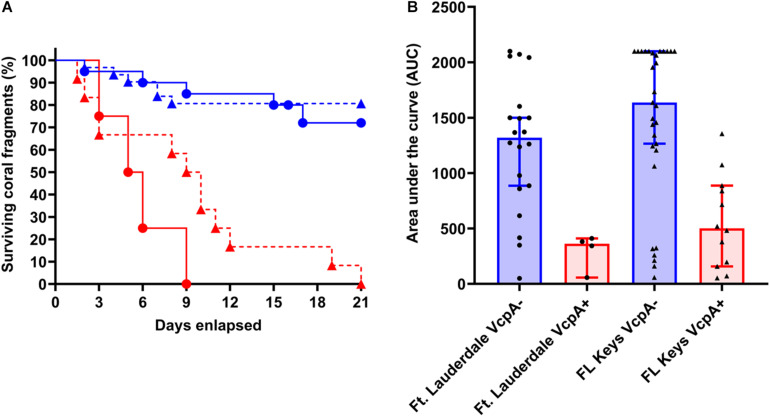
Survival rates and disease progression of diseased *M. cavernosa* compared by collection location and VcpA assay result. **(A)** Kaplan-Meyer survival curves of diseased *M. cavernosa* separated by collection origin and VcpA assay result. **(B)** Area under the curve (AUC) calculation of disease progression for *M. cavernosa* separated by collection origin and VcpA assay result. Corals negative for VcpA are represented by blue lines with circles (Ft. Lauderdale origin), blue dashed lines with triangles (FL Keys origin), blue bars with black circles (Ft. Lauderdale origin), or blue bars with black triangles (FL Keys origin). Corals positive for VcpA are represented by red lines with circles (Ft. Lauderdale origin), red dashed lines with triangles (FL Keys origin), red bars with black circles (Ft. Lauderdale origin), or red bars with black triangles (FL Keys origin). Bars on the AUC analysis represents the median of the sample set and the bars represent the 90% confidence interval.

There was intraspecific variation in survival and disease progression between the abovementioned diseased corals (*n* = 102), which seemed to be independent of their captivity conditions, which were being controlled (Supplementary Files S1 S6). Therefore, data on disease progression and survival over a 21-day period were analyzed for correlations to coral origin or the presence of VcpA. There were significant differences between diseased corals that tested VcpA^+^ compared to VcpA^–^ fragments. VcpA^+^ *M. cavernosa* had lower survival rates (Mantel-Cox test, *p* < 0.0001) (Figures 1A–D) compared to VcpA^–^ fragments (Figures 1E–L, 3A). In addition, VcpA^+^ fragments had faster disease progression than VcpA^–^ fragments (two-tailed Wilcoxon test, *p* < 0.0001) (Figure 3B). Disease progression was based on the area under the curve (AUC) calculated when the percent remaining healthy tissue is plotted over time for each fragment. The AUC measurements were standardized to fragment size and initial tissue because the percent remaining healthy tissue compared to the initial area (day 0) of tissue was plotted instead of raw area measurements to calculate the AUC. The VcpA^–^ *M. cavernosa* mean survival was 17.4 d (*n* = 53) compared to the VcpA^+^ fragments with a mean of 8.5 days (*n* = 14). During the 21-d observation period, 21.6% of VcpA^–^ and 100% of VcpA^+^ *M. cavernosa* fragments experienced complete mortality. For the VcpA^+^ fragments (*n* = 14), 78.6% had lesions with active tissue loss without obvious bleaching (Figures 1A–D and Supplementary File S6), 21.4% had tissue loss and bleaching (Figures 1I–L), and none had bleaching only lesions. When the diseased *M. cavernosa* were compared by collection location and their VcpA result (Figure 4), the only significant differences among survival (Mantel-Cox test, *p* < 0.0001, for either region) and disease progression (two-tailed Wilcoxon test, *p* = 0.0051 Ft. Lauderdale; *p* = 0.002 FL Keys) were between corals positive and negative for VcpA. Similarly, diseased *O. faveolata* followed the same pattern with lower survival (Mantel-Cox test, *p* = 0.0002) and faster disease progression (two-tailed Wilcoxon test, *p* = 0.0058) with VcpA^+^ fragments (Figures 3C,D). The mean survival of VcpA^–^ *O. faveolata* fragments was 18 d (*n* = 18) and 9 d (*n* = 6) for VcpA^+^ fragments, with 33.33% of VcpA^–^ and 100% of VcpA^+^ having complete mortality during the 21-day observation period.

Collection location alone did not correlate with survival for *M. cavernosa* (Mantel-Cox Test, *p* = 0.83) or disease progression rates (two-tailed Wilcoxon test, *p* = 0.43) (Figure 4). Survival ranged from 2 to 21 d (mean 15.3 d, *n* = 24) for Ft. Lauderdale corals and 1.5 to 21 d (mean = 15.5 d, *n* = 43) for the Florida Keys fragments. Similarly, there was no difference between survival (Mantel-Cox Test, *p* = 0.82) and disease progression (two-tailed Wilcoxon test, *p* = 0.79) for *O. faveolata* from Ft. Lauderdale or the Florida Keys. Survival ranged from 3 to 21 d (mean 13.8 d, *n* = 4) for Ft. Lauderdale corals and 5 to 21 d (mean = 16 d, *n* = 20) for the Florida Keys fragments. In all, these correlations suggest that the intraspecific differences between disease presentation on *M. cavernosa* and *O. faveolata* are in part due to the presence of VcpA-producing *V. coralliilyticus* populations on a subset of diseased corals.

### Inoculation of Healthy Corals With *V. coralliilyticus* Did Not Consistently Initiate Tissue Loss

Although *V. coralliilyticus* was not detected on all diseased corals, it was unclear if this bacterium was infecting corals in addition to the unknown SCTLD pathogen(s) or potentially exacerbating pre-existing SCTLD lesions. Therefore, axenic cultures of *V. coralliilyticus* were tested for their virulence with apparently healthy corals using controlled inoculation experiments (Table 2). Due to the limited availability of healthy corals, the inoculation experiments were focused on one strain, *V. coralliilyticus* strain OfT6-21, the most virulent strain during the screening experiments. Interestingly, OfT6-21 did not induce disease signs with apparently healthy *O. faveolata* (*n* = 8), while only 1 healthy *M. cavernosa* developed tissue loss after 8 days post-exposure to OfT6-21 (*n* = 7) (Table 2). Additionally, 1 of 3 healthy *Meandrina meandrites* fragments developed tissue loss 12 days post-exposure to OfT6-21. All inoculation experiments were monitored for at least 21 days. Disease signs were not attributed to inadequate husbandry because no FSW control corals or those exposed to the McH1-7 bacterial controls developed tissue loss or bleaching. Therefore, *V. coralliilyticus* was likely not acting as a primary pathogen and only caused tissue loss on a subset of corals tested; however, because of the association with VcpA and acute lesions, it may be initiating secondary infections or contributing to coinfections.

### Physical Response of Corals to *V. coralliilyticus*

When healthy corals were being inoculated with *V. coralliilyticus*, an immediate physical response to this bacterium was observed (Supplementary File S7). Apparently healthy fragments of *M. cavernosa* responded to exposure to OfT6-21 with a muco-ciliary response within 5 min of inoculation (Supplementary File S7B). All corals treated with OfT6-21 appeared to increase mucus production and apparently aggregate the bacterial inoculum (*n* = 6). This behavior was not observed with cultures of McH1-7, which was isolated from apparently healthy *M. cavernosa* (Supplementary File S7A).

**FIGURE 5:**
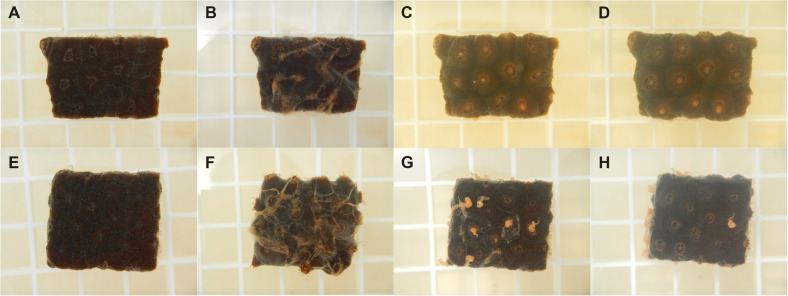
Physical response of *M. cavernosa* to coral feed mixed with *V. coralliilyticus*. Photos of *M. cavernosa* fragments fed coral feed mixed with **(A–D)** *Pseudoalteromonas* sp. McH1-7 isolated from healthy coral, or **(E–H)** *V. coralliilyticus* strain OfT6-21 from diseased coral. Photos were taken **(A,E)** before feeding, **(B,F)** 15 min post-feeding, **(C,G)** 60 min post-feeding, and **(D,H)** 120 min post-feeding. The grating in the photos are 1.5 cm × 1.5 cm.

A similar response was observed when OfT6-21 and McH1-7 cultures were mixed with coral feed (Reef Roids, PolypLab Inc.) and inoculated onto apparently healthy *M. cavernosa* that had been starved for 7 days. Only corals treated with OfT6-21 exhibited an obvious abnormal behavioral response (Figure 5 and Supplementary File S8). Regardless of the treatment, corals began to consume the food mixture within 5 min (Figures 5A,E). After 15 min, the food mixture with McH1-7 was completely consumed by the corals (Figure 5C), after which the polyps re-extended all their tentacles again. In contrast, the fragments fed the mixture with *V. coralliilyticus* seemingly expelled the food particles and the polyps remained retracted (Figure 5G). After 30 min, the mixture with McH1-7 was apparently digested by the coral with no obvious signs of stress (Figure 5D). However, the mixture with *V. coralliilyticus* was expelled in masses of mucus and remained loosely associated on top of the fragment (Figure 5H). Eventually, the coral appeared to slough these particles off its surface over the next 60 min until the end of observation (120 min post inoculation). Though mainly behavioral data, it appears that *V. coralliilyticus* triggers a muco-ciliary response in healthy *M. cavernosa* that can deter feeding, which could partially explain this bacterium’s inability to consistently induce disease with healthy corals.

### All *V. coralliilyticus* Strains Are Resistant to Most Antibiotics Effective Against SCTLD

**FIGURE 6:**
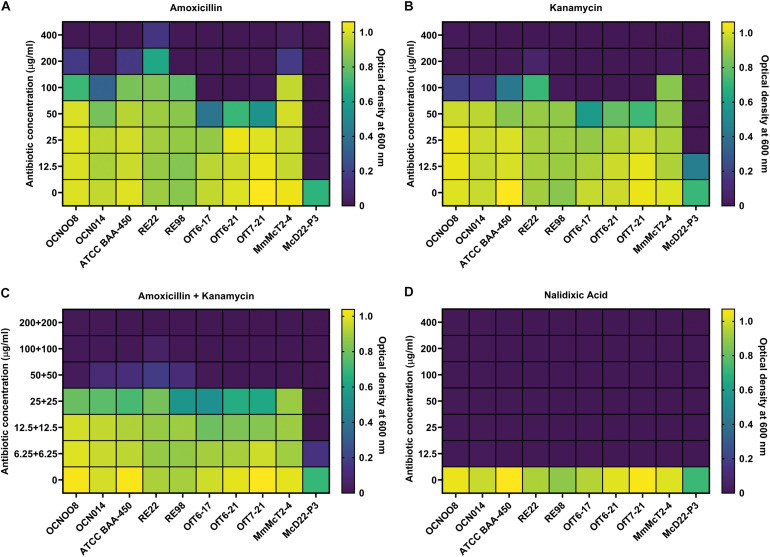
Susceptibility of various *V. coralliilyticus* strains to select antibiotics. Heat maps of bacterial cultures grown with varying concentrations of the antibiotics **(A)** amoxicillin, **(B)** kanamycin, **(C)** a 1:1 mixture of amoxicillin and kanamycin, and **(D)** nalidixic acid. Bacterial growth was measured as optical density at 600 nm. Strains of *V. coralliilyticus* from the Indo-Pacific (OCN008, OCN014, BAA-450, RE22, and RE98), the Atlantic (OfT6-17, OfT6-21, OfT7-21, and MmMcT2-4), and the non-*coralliilyticus* control strain McD22-P3 were tested. Measurements were taken after 24 h of growth and are the mean of three replicates.

The progression of SCTLD was arrested with certain antibiotics in laboratory aquaria (Aeby et al., 2019), and current *in situ* treatments include the use of an amoxicillin paste (Florida Keys National Marine Sanctuary, 2018), therefore, resistance to these antibiotics was evaluated using the *V. coralliilyticus* strains from this study and known pathogenic strains from the Indo-Pacific (Table 1). Resistance to the beta-lactam amoxicillin, the aminoglycoside kanamycin, and the quinolone nalidixic acid was evaluated and the minimum inhibitory concentration (MIC) was estimated. For amoxicillin, all *V. coralliilyticus* were generally resistant to this antibiotic (Figure 6). For the Indo-Pacific strains BAA-450 and OCN014 the MIC for amoxicillin was between 100 and 200 μg/ml, the MIC for OCN008 was 200–400 μg/ml, and the MIC for RE22 was greater than 400 μg/ml (Figure 6A). In contrast, the MIC of amoxicillin for the Atlantic strains OfT6-17, OfT6-21, and OfT7-21 was between 50 and 100 μg/ml, but strain MmMcT2-4 had an MIC between 200 and 400 μg/ml (Figure 6A). For kanamycin, the MIC for most of the strains was 50–100 μg/ml, except for OCN008 and MmMcT2-4 that had an MIC between 100 and 200 μg/ml, and RE22 with an MIC between 200 and 400 μg/ml (Figure 6B). An equal combination of amoxicillin and kanamycin, previously used by Aeby et al. (2019), inhibited the growth of most strains with 50 μg/ml of each antibiotic, however, 100–200 μg/ml of each antibiotic in combination was required to inhibit the growth of OCN008 and RE22 (Figure 6C). Interestingly, the MIC for nalidixic acid was less than 12.5 μg/ml for all strains tested (Figure 6D). The MIC for the non-*coralliilyticus Vibrio* sp. McD22-P3 was 4–8× lower in comparison. In general, amoxicillin or kanamycin are ineffective at inhibiting *V. coralliilyticus* growth in culture.

### Physiological Characteristics Do Not Differ Between Atlantic Strains and Indo-Pacific Strains of *V. coralliilyticus*

Multiple Indo-Pacific *V. coralliilyticus* strains are coral and shellfish pathogens, so various biochemical and physiological characteristics were compared between the Indo-Pacific and the Atlantic-Caribbean strains isolated in this study to identify any potential differences that may relate to pathogenesis. Growth rate, salinity tolerance, swarming, biofilm production, and protease activity were compared among nine different *V. coralliilyticus* strains as well as *Vibrio* sp. McD22-P3, a non-*coralliilyticus* strain from a diseased *M. cavernosa* as a control (Table 1). Both strain (two-way ANOVA, *p* < 0.0001) and salinity (two-way ANOVA, *p* < 0.0001) had a significant effect on the growth of the bacteria. Post-hoc comparisons revealed that MmMcT2-4, had a slower logarithmic growth (based on the slope of the graph during 7–12 h for MmMcT2-4 and 3–8 h for all other strains) at all salinities (Tukey’s multiple comparison’s test, *p* < 0.01, *n* = 6) (Supplementary File S9). Interestingly, the Florida Keys isolate MmMcT2-4 appeared to have a longer lag phase compared to the other strains, reaching log phase approximately 4 h after the other strains. When the growth rate was compared between 23 and 29°C, there were no significant differences between the slopes of the linear regression lines generated for logarithmic growth phase (3 to 8 h post-inoculation) based on the Graphpad Prism linear regression analysis comparing the slopes for all *V. coralliilyticus* strains (*n* = 6 each strain): OCN008 (*p* = 0.65), OCN014 (*p* = 0.77), BAA-450 (*p* = 0.72), RE22 (*p* = 0.90), RE98 (*p* = 0.88), OfT6-17 (*p* = 0.64), OfT6-21 (*p* = 0.60), OfT7-21 (*p* = 0.59), McD22-P3 (*p* = 0.96) (Supplementary File S10). MmMcT2-4 may have a slightly faster growth rate at lower temperatures (linear regression analysis, *p* = 0.06); however, more detailed investigations are needed to make any solid conclusions.

The strain measured (two-way ANOVA, *p* < 0.0001 *n* = 3 for each salinity) and incubation time (two-way ANOVA, *p* < 0.0001) both significantly influenced the swarming radii when compared at each salinity tested separately (Supplementary File S11). However, the more biologically relevant comparison was the effect of salinity and strain on swarming radii. At 4 d post-inoculation, the strain used (two-way ANOVA, *p* < 0.0001, *n* = 3) and salinity (two-way ANOVA, *p* < 0.0001) significantly influenced swarming radii. For most of the *V. coralliilyticus* strains, swarming radii were not significantly different between 35 and 25 ppt based on Tukey’s multiple comparisons test. Most strains had smaller swimming radii at 10 ppt compared to 25 ppt (Tukey’s multiple comparisons test, *n* = 3): OCN008 (*p* = 0.002), OCN014 (*p* = 0.001), BAA-450 (*p* = 0.01), RE98 (*p* = 0.01), OfT6-21 (*p* = 0.04). The major exceptions were Pacific oyster-associated strain RE22 and FL Keys strain MmMcT2-4. For RE22, salinity did not impact swarming behavior between 35 and 25 ppt (Tukey’s multiple comparison test, *p* = 0.99, *n* = 3) or 25 ppt and 10 ppt (Tukey’s multiple comparison test, *p* = 0.07, *n* = 3). McMcT2-4 was similar in that swarming behavior between 35 and 25 ppt (Tukey’s multiple comparison test, *p* = 0.18, *n* = 3) or 25 ppt and 10 ppt (Tukey’s multiple comparison test, *p* = 0.34, *n* = 3) was not significantly different. An aflagellate OCN008Δ*fliG*1 strain (Ushijima and Häse, 2018) was comparable to most of the other *V. coralliilyticus* strains, suggesting the apparent swarming motility observed was not due to flagellar-based movement across the agar. Additionally, all nine *V. coralliilyticus* strains, except the OCN008Δ*fliG*1 strain, displayed swimming motility when observed under light microscopy (data not shown).

**FIGURE 7:**
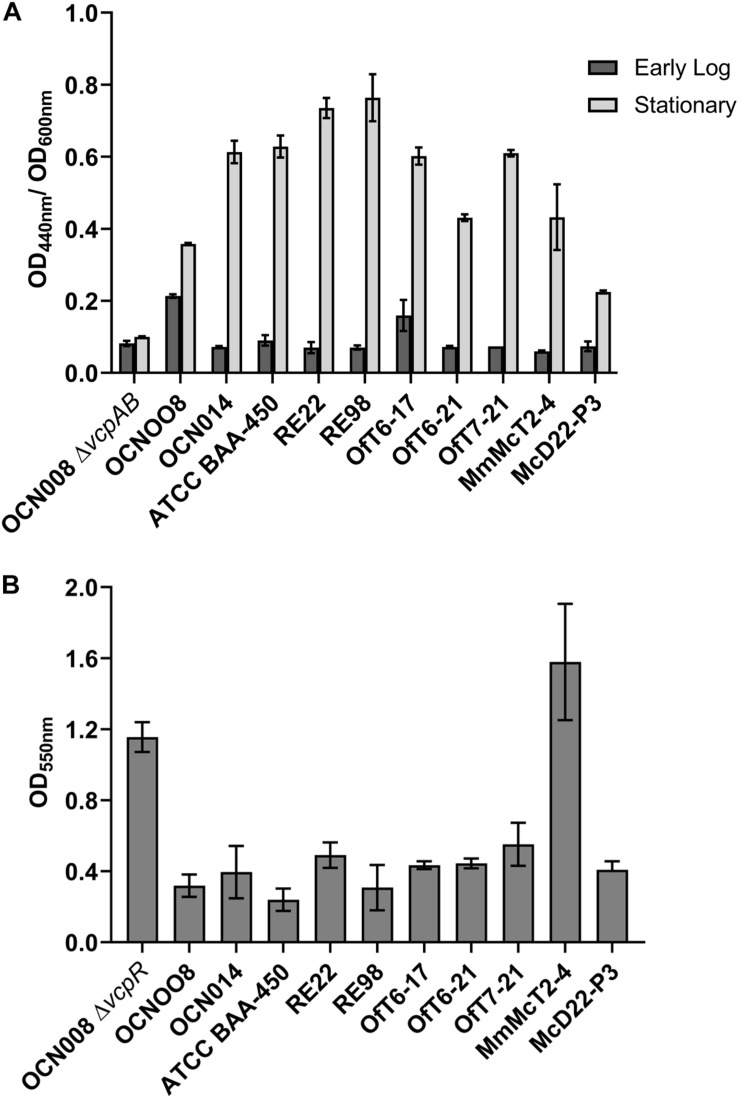
Physiological comparison of Atlantic and Indo-Pacific *V. coralliilyticus* strains. **(A)** Azocasein assays with dark gray bars representing mean protease activity at early logarithmic cultures (OD_600nm_ = 0.8) and light gray bars representing stationary phase (OD_600nm_ = 3.0). All absorbance measurements taken at 440 nm were standardized to the culture density (OD_600nm_). The graph displays the mean of three replicates and bars represent the standard error of the mean. **(B)** Crystal violet extracellular polysaccharide (biofilm) assays. The graph displays the mean of four replicates and the bars represent the standard error of the mean.

All nine *V. coralliilyticus* strains investigated during this study elicited a positive reaction on the VcpA *RapidTest* assay and yet they differ in reported virulence. The VcpA *RapidTest* generates qualitative, plus or minus results. Therefore, to examine the total protease activity of each strain in more detail to determine if protease activity correlates with virulence, the total protease activity of each strain was measured using a biochemical azocasein assay (*n* = 3 per strain) (Figure 7A). There were significant differences between strains (two-way ANOVA, *p* < 0.001) and phase of growth (two-way ANOVA, *p* < 0.001) on the protease assays. At early log phase, in comparison to the protease mutant (OCN008Δ*vcpAB*), only the Pacific strain OCN008 (Dunnett’s multiple comparisons test, *p* < 0.0001) and Atlantic strain OfT6-17 (Dunnett’s multiple comparisons test, *p* = 0.03) had significantly higher protease activity. In contrast, at stationary phase (OD_600nm_ = 2.0), all *V. coralliilyticus* strains had significantly higher protease activities compared to the Δ*vcpAB* negative control (Dunnett’s multiple comparisons test, *p* < 0.0001). However, there were no obvious patterns between total protease activity and the origins of the strains or their reported virulence. For example, Pacific strain OCN014 had higher protease activity than Atlantic strain OfT6-21 (Tukey’s multiple comparison test, *p* < 0.0001), but equivalent to another Atlantic strain OfT6-17 (Tukey’s multiple comparison test, *p* > 0.99). Therefore, other characteristics were pursued to identify any potential correlations. In addition to protease activity, extracellular polysaccharide (EPS) production, as a proxy for biofilm formation, was also measured (Figure 7B). In comparison to the control, the OCN008Δ*vcpR* strain that has enhanced EPS production (Guillemette et al., 2020), only the Atlantic strain MmMcT2-4 had comparable EPS production (Dunnett’s multiple comparison test, *p* = 0.16, *n* = 4).

Lastly, all nine strains were compared using the API 20E assay strips (BioMérieux) to detect any potential differences using biochemical assays (Supplementary File S12). Out of the 21 tests conducted, there were only 5 tests where the strains differed: β-galactosidase (ONPG), where OCN014 was the only strain lacking activity; arginine dihydrolase (ADH), where RE22 was the only strain lacking activity; citrate utilization (CIT), where only OCN008, OCN014, and MmMcT2-4 were able to utilize this carbon source; mannose fermentation (MAN), where RE98 was the only strain unable to ferment this sugar; and amygdalin fermentation (AMY), where OCN014, RE98, BAA-450, and MmMcT2-4 were the only strains unable to ferment this sugar. In all, there are some *V. coralliilyticus* strains with unique characteristics, however, there were no obvious correlations between their physiology, origin, or virulence.

### Genome Analysis of *V. coralliilyticus* Isolates

**FIGURE 8:**
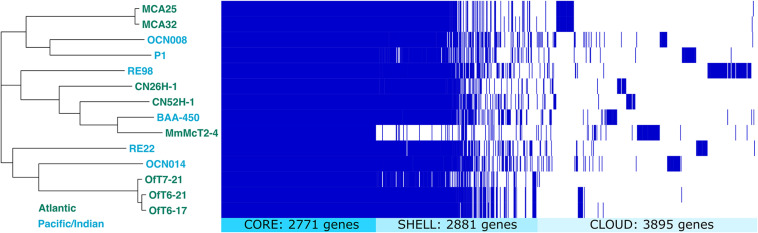
Pangenome comparison of fourteen *V. coralliilyticus* strains. Clustering of genomes is based on the alignment of 2,771 core genes present in all strains. Shell genes were present in three to 13 of the genomes. Cloud genes were present in only one or two of the genomes.

Pairwise comparison of the average nucleotide identity of shared genes for eight newly isolated strains of *V. coralliilyticus* ranged from 96.8 to 100% (Supplementary File S13). All three strains isolated from diseased *O. faveolata* corals, strains OfT6-17, OfT6-21, and OfT7-21, had 100% sequence identity of shared genes. Likewise, the two strains isolated from healthy *M. cavernosa*, strains MCA-25 and MCA-32 (Table 1 and Supplementary File S2), had 100% sequence identity of shared genes. However, pangenome analysis showed that each of the eight genomes contained unique combinations of genes such that some genes present in one strain were not found in all strains, even when shared genes were identical (Figure 8). While most of these draft genomes are of excellent quality and more than 90% complete (Supplementary File S15), it is possible that some of these genomes, if finished completely (i.e., a closed, circular genome was obtained), may be completely identical to each other.

The pangenome of all 14 strains of *V. coralliilyticus* with sequenced genomes contained a total of 9,547 genes, with 2,771 core genes (present in all strains), 2,881 shell genes (present in three to 13 of the strains), and 3,895 cloud genes (present in only one or two strains) (Figure 8). Overall, the phylogenetic tree based on the alignment of core genes did not cluster genomes based on geographic origin (Atlantic versus Indian or Pacific Oceans), nor by host type. For example, strains RE22 and RE98 were both isolated from Pacific oysters in Netarts Bay, Oregon, United States, but cluster with strains isolated from corals instead of with each other. In general, strains from the same coral species clustered together, for example, the three strains from a diseased *M. cavernosa* infection of a healthy *O. faveolata* (OfT6-17, OfT6-21, OfT7-21) clustered together and the two strains from an apparently healthy *C. natans* (CN26H-1 and CN52H-1) clustered together.

**FIGURE 9:**
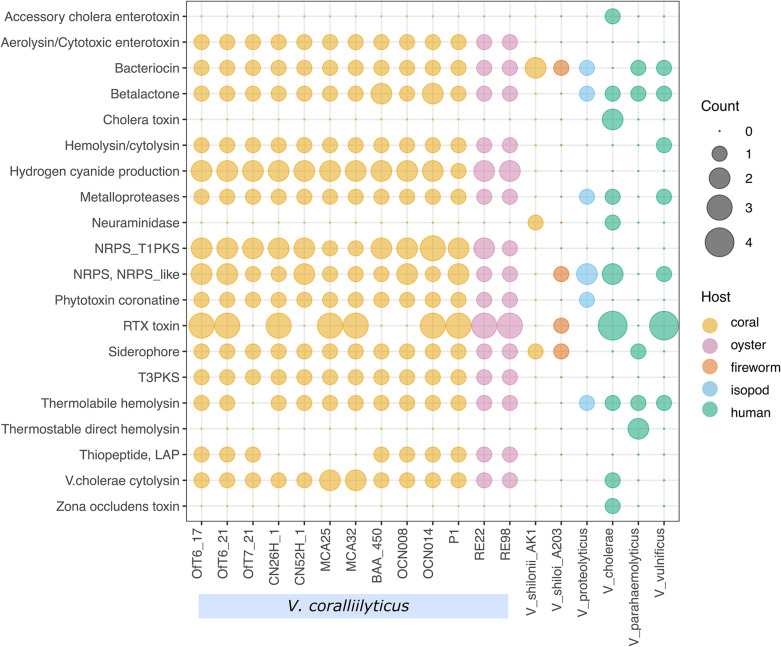
Virulence factors and secondary metabolites in nineteen pathogenic *Vibrio* species. Gene counts associated with virulence factors and the biosynthesis of secondary metabolites in V. coralliilyticus and other vibrios from corals, fireworms, isopods, and human-associated pathogens.

A comparison of 13 of the 14 genomes of *V. coralliilyticus* with six other vibrio pathogens, including the marine invertebrate pathogens *V. proteolyticus* NBRC 13287, *V. shilonii* AK1, and *V. shiloi* A203, as well as the human pathogens *V. cholerae* O1 biovar El Tor strain N16961, *V. parahaemolyticus* strain RIMD2210633, and *V. vulnificus* strain CMCP6, revealed diverse genes for the production of toxins and secondary metabolites in *V. coralliilyticus* (Figure 9). *V. coralliilyticus* strain MmMcT2-4 was excluded from the results of this analysis for clarity as the genome was only 70% complete. Overall, the *V. coralliilyticus* genomes have the potential to make more kinds of toxins and secondary metabolites than any of the other six *Vibrio* species. First, all of the *V. coralliilyticus* genomes have a vibriolysin-like zinc-metalloprotease. Vibriolysin metalloproteases identified in *V. proteolyticus*, *V. chloerae*, and *V. vulnificus* were not closely related to the vibriolysin-like zinc-metalloprotease in the *V. coralliilyticus* genomes. In other words, the vibriolysin primers developed here for *V. coralliilyticus* would not work for other *Vibrio* species. All the *V. coralliilyticus* genomes have genes to produce hydrogen cyanide, hemolysin/cytolysin, and aerolysin/cytotoxic enterotoxin that were generally absent in the other pathogenic vibrios. In addition, all the *V. coralliilyticus* genomes have the gene for *V. cholerae* cytolysin that are not present in other pathogenic vibrios except for *V. cholerae*. Genes for the biosynthesis of siderophores were present in all *V. coralliilyticus* genomes, but inconsistently found in other pathogenic vibrios.

Perhaps most interesting is the observation that all the *V. coralliilyticus* genomes isolated from diseased corals and oysters have the genes to produce a thiopeptide, which contains a linear azole/azoline-containing peptide (LAP), while the four strains of *V. coralliilyticus* isolated from healthy corals, do not. Thiopeptides are a diverse class of antibiotics that inhibit protein synthesis in Gram-positive bacteria, but generally have no effect on Gram-negative bacteria (Bagley et al., 2005).

All the *V. coralliilyticus* genomes also have diverse genes for the secretion of these toxins and secondary metabolites, including Type I, Type II, Type III, Type IV, and Type VI secretion systems. In particular, T3SS and T6SS are important secretion systems for pathogenesis, with their needle-like delivery of toxins and enzymes through host membranes (Costa et al., 2015). The presence of an active T6SS in *V. coralliilyticus* was confirmed in a previous study (Guillemette et al., 2020). Lastly, all the *V. coralliilyticus* genomes contained several genes for multidrug export proteins (*emrB*, *mepA*), multidrug resistance proteins (*mdtABCEGHKLMN*, *mexAB*, *norM*), multidrug transporter (*emrE*), and putative multidrug resistance protein (*emrK*), which may contribute to antibiotic resistance in *V. coralliilyticus*.

## Discussion

The study presented here initially focused on culturing potential pathogens from SCTLD lesions. Subsequently, various bacteria were identified that sometime elicited disease lesions when inoculated onto apparently healthy corals. These isolates belonged to the bacterial families Alteromonadaceae, Rhodobacteraceae, and Vibrionaceae, which, in a previous study, are families enriched for in SCTLD lesions (Meyer et al., 2019; Rosales et al., 2020). However, these isolates do not appear to be the primary cause of SCTLD as they do not consistently elicit disease during controlled experiments. Although, it was curious that all the Vibrionaceae isolated from diseased corals during the virulence screens were identified as the known coral pathogen *V. coralliilyticus*, yet, they were unable to repeatedly initiate disease and elicited a strong response from healthy corals. Along with an already available immunoassay that can rapidly screen for a toxic protein produced by this bacterium, these circumstances prompted further investigations with diseased corals from the field.

The bacterium *V. coralliilyticus* is generally described as a primary cause of disease for a variety of invertebrates, including corals. However, coral diseases can be complex, polymicrobial infections involving multiple pathogenic organisms (Bourne et al., 2009; Bourne and Webster, 2013; Meyer et al., 2017). This complexity is demonstrated in this study, where *V. coralliilyticus* was not believed to be acting as a primary cause of disease but instead its presence is linked to more acute infections of SCTLD. This observation is further supported by the screening of diseased corals collected from SCTLD endemic regions using an improved immunoassay specific for toxic protein VcpA. Although, the primary etiological agent(s) for SCTLD is unknown, the results presented here elucidate a potential reason for differences in disease presentation between cases.

Previous studies have described inter- and intraspecific differences between corals with SCTLD (Precht et al., 2016; Aeby et al., 2019), which could be explained by differences in host immune responses or different etiologies. *V. coralliilyticus* may be playing an important role in SCTLD pathogenesis because of the strong correlation with its presence and increased disease progression and mortality rates. While immune responses were not investigated here, a positive correlation between the presence of a proteolytic toxin produced by the bacterium *V. coralliilyticus* and more virulent disease lesions was discovered. VcpA is toxic zinc-metalloprotease that is a shellfish virulence factor secreted by *V. coralliilyticus* and is a reliable biomarker for the presence of viable pathogen and lethal vibriosis in shellfish larvae (Hasegawa et al., 2008, 2009). It was able to be detected by the VcpA *RapidTest*, a simple single-step assay contained in a single use disposable cassette (Supplementary File S4) and is suitable for use either in a lab or in the field. The tests followed the protocol designed for shellfish samples (Supplementary File S5) with the modifications described above. The VcpA metalloprotease is responsible for a majority of the proteolytic activity in *V. coralliilyticus* cultures (Hasegawa et al., 2008, 2009; Santos Ede et al., 2011). Purified metalloprotease from *V. coralliilyticus* can inactivate photosystem II in zooxanthellae or cause tissue degradation on exposed corals (Ben-Haim et al., 2003; Sussman et al., 2009). This is consistent with reports on strains of *V. coralliilyticus* capable of causing bleaching or tissue loss with various coral hosts (Ben-Haim et al., 2003; Sussman et al., 2008; Vezzulli et al., 2010; Ushijima et al., 2014a, 2016).

The *V. coralliilyticus* strains described in this study were unable to reproducibly induce obvious disease signs when applied to apparently healthy corals, even when using a concentrated dose of 10^8^ CFU/ml. It is possible there are specific environmental factors required for disease, like elevated seawater temperatures, which are linked to increased virulence for some *V. coralliilyticus* strains (Ben-Haim et al., 2003; Ushijima et al., 2016). But in this study, VcpA, or copies of the corresponding gene, was not detected on every active lesion, suggesting that this bacterium is not the primary cause of SCTLD. Additionally, apparently healthy corals appear to recognize and physically remove disease-associated strains of *V. coralliilyticus*, which indicates this bacterium may not normally colonize healthy corals or is maintained at sub-infectious concentrations. Furthermore, preliminary studies by other groups suggest that protease production is only linked to active tissue loss (Gavish et al., 2018), suggesting that active *V. coralliilyticus* infections are being detected by the VcpA assay. It is possible the VcpA antibodies may bind a structurally similar protein, so to further verify the results from the immunoassays, digital droplet PCR (ddPCR) was used to quantify the concentration of the *vcpA* gene in diseased samples. Therefore, with the dual verification using antibodies specific to the VcpA protein and a quantitative PCR method specific to the *vcpA* DNA sequence, there was a high level of confidence in the detection of *V. coralliilyticus.* The ddPCR results found that VcpA^–^ corals had no copies or significantly fewer copies of *vcpA* compared to VcpA^+^ fragments. Previous studies have demonstrated that a specific concentration of *V. coralliilyticus* must be present before gross disease signs manifest and VcpA is produced, therefore, the mere presence of this bacterium does not necessarily indicate infection (Ben-Haim and Rosenberg, 2002; Hasegawa and Häse, 2009; Ushijima et al., 2014a, 2016).

There are several explanations for the association of *V. coralliilyticus* and diseased corals, ranging from it being an opportunistic colonizer to an opportunistic pathogen causing secondary or coinfections. First, it could simply be that *V. coralliilyticus* is an opportunistic colonizer of SCTLD lesions, taking advantage of the excess nutrients released from lysed coral tissue and the potential reduced ability of the coral to slough bacteria. Although this is possible, the strong correlation between increased virulence and the presence of VcpA suggests that this bacterium is playing a more pathogenic role. Second, *V. coralliilyticus* could be acting as an opportunistic pathogen causing secondary or coinfections. If *V. coralliilyticus* is causing secondary infections (as a secondary pathogen), then that would require a preceding disruption of the host immune system or an initial infection by a primary pathogen (Pasman, 2012). However, when healthy corals were exposed to *V. coralliilyticus*, there were still some instances of tissue loss developing after exposure, albeit a rare occurrence during controlled experiments. More likely, *V. coralliilyticus* is causing a coinfection; infections by opportunistic pathogens that can occur without a previous infection but are more likely to occur with a preexisting condition (Pasman, 2012). A common medical example of this are the coinfections resulting in bacterial pneumonia after an initial case of influenza (Morris et al., 2017). However, similar relationships have been observed with coral diseases as well. The Hawaiian rice coral, *Montipora capitata*, is susceptible to chronic tissue loss diseases caused by pathogens such as *Vibrio owensii* (Ushijima et al., 2012), but these can become acute infections following exposure to *Pseudoalteromonas piratica* (Beurmann et al., 2017). Acute infections of healthy *M. capitata* by *P. piratica* are possible, but fragments with preexisting chronic infections are almost two-times more likely to develop acute tissue loss with a three-times shorter incubation period (Beurmann et al., 2017).

An analogous scenario may be occurring with SCTLD lesions with coinfections by *V. coralliilyticus*. On average, the diseased *M. cavernosa* had chronic to sub-acute lesions, while VcpA^+^ fragments exhibited acute tissue loss with complete mortality within 21 days. Additionally, the production of VcpA is positively correlated with cell density (Hasegawa and Häse, 2009), implying a higher concentration of *V. coralliilyticus* on VcpA^+^ lesions, which was also demonstrated with the ddPCR results, thus suggesting that a SCTLD lesion allows pathogenic *V. coralliilyticus* strains to better colonize *M. cavernosa* and contribute to host damage, which it is normally unable to do with healthy corals. However, it is unclear if other pathogens are needed for this coinfection or if only *V. coralliilyticus* is required, as well as what is the cause for the acute lesions on some VcpA^–^ fragments. These results highlight the need for understanding SCTLD pathogenesis, which may be more complex than initially assumed, as well as the importance of effective diagnostics.

In addition to the complexity of SCTLD, these results emphasize the extensive genetic potential of *V. coralliilyticus*. While the initial goal was to identify any commonalities between *V. coralliilyticus* strains from different regions or those that can directly infect corals, their genetic plasticity further supports the notion that this species has an incredibly diverse population structure. This has been suggested by other studies (Pollock et al., 2010; Kimes et al., 2011; Castillo et al., 2018), which is now supported by additional strains from the Atlantic. Furthermore, there appeared to be no correlation between phylogeny of the different strains and their original host, which could suggest a general level of virulence toward various hosts encoded by their core genes, or the evolution of different strains through the acquisition of mobile genetic elements. Regardless, this would imply that populations of *V. coralliilyticus* are already or can be transformed into generalist opportunistic pathogens posing a threat to various organisms. Additionally, these results highlight the potential for this species to acquire genetic material, which can lead to the evolution of more virulent strains. Interestingly, there were differences identified between strains isolated from diseased corals and healthy corals, like the biosynthesis genes for thiopeptide antibiotics. This bacterium does possess antibacterial systems like a T6SS and can alter the microflora of corals like *M. cavernosa* (Welsh et al., 2017; Guillemette et al., 2020), which is not surprising since it is believed that the coral microflora protects them from pathogens such as *V. coralliilyticus* (Nissimov et al., 2009; Rypien et al., 2010; Charlotte et al., 2011; Rosado et al., 2018). However, it is currently unclear if *V. coralliilyticus* produces thiopeptide antibiotics or if their production would support their colonization of corals. Additionally, the various other potential virulence factors encoded within this species call for further inquiry into the role *V. coralliilyticus* plays in SCTLD.

This study may demonstrate an interesting scenario in coral disease, but also highlights how important understanding the etiology of a disease is to management. Diagnostic tools like the *RapidTest* immunoassays can provide results in less than 10 min, but development of this assay would have been impossible without first identifying and understanding *V. coralliilyticus*. Furthermore, outside of the results of this study, there are no known intraspecific markers for more virulent lesions, which would be an important diagnostic for intervention efforts. Amoxicillin is used as a treatment for SCTLD in the field (Florida Keys National Marine Sanctuary, 2018; Neely et al., 2020), but coinfections by *V. coralliilyticus*, which is highly resistant to this antibiotic, may be problematic for mitigation efforts. Unfortunately, it is unclear what concentrations of amoxicillin *V. coralliilyticus* would experience in the field, so this statement is currently pure speculation. The authors would like to note, in response to the results presented here, we do not condone the adoption of quinolones or related compounds for use in the environment because of their importance in human medicine and the emergence of widespread resistance (Ruiz, 2003; Jacoby, 2005). However, the potential interactions between coinfections and treatments warrant further investigations to support the management of this devastating disease. As for any disease outbreak, continued research into the etiology and pathogenesis are essential for effective transmission management, treatment of disease individuals, and protection of healthy populations.

## Data Availability Statement

The datasets presented in this study can be found in online repositories. The names of the repository/repositories and accession number(s) can be found in the article/Supplementary Material.

## Author Contributions

BU, CH, and VP: project conception. BU, JM, ST, EW, JR, RW, and VP: experimental design. BU, JM, ST, KP, MM, JT, EW, JR, RW, GA, and VP: performing experiments. BU, JM, KP, JT, JR, RW, and VP: data analysis. BU, JM, and EW: draft writing. BU, JM, MM, JT, GA, and VP: draft reviewing and editing. BU, JM, MM, GA, CH, and VP: funding acquisition. All authors contributed to the article and approved the submitted version.

## Conflict of Interest

MM was employed by and owns shares of the company mAbDx. The remaining authors declare that the research was conducted in the absence of any commercial or financial relationships that could be construed as a potential conflict of interest.
