# Supplementary material for: Disease Diagnostics and Potential Coinfections by Vibrio coralliilyticus During an Ongoing Coral Disease Outbreak in Florida
Source: Front Microbiol. 2020 Oct 26;11:569354. doi: 10.3389/fmicb.2020.569354 (PMC7649382; doi:10.3389/fmicb.2020.569354)
Supplement: Supplementary file 1 [file Data_Sheet_1.zip › S Files/Supplementary File (S2).docx]

**Supplementary File S2. Origin of coral hosts for the Florida *V. coralliilyticus* strains.**

| **Strains** | **Diseased coral species** | **Diseased coral origin (GPS coordinates)** | **Healthy or infected coral species** | **Healthy or infected coral origin** |
| --- | --- | --- | --- | --- |
| *V. coralliilyticus*  OfT6-17 | *M. cavernosa* | Ft. Lauderdale, FL  (26° 08'54.91" N 81°05'45.29" W) | *O. faveolata* | NOAA Key West Nursery |
| *V. coralliilyticus*  OfT6-21 | *M. cavernosa* | Ft. Lauderdale, FL  (26° 08'54.91" N 81°05'45.29" W) | *O. faveolata* | NOAA Key West Nursery |
| *V. coralliilyticus*  OfT7-21 | *M. cavernosa* | Ft. Lauderdale, FL  (26° 08'54.91" N 81°05'45.29" W) | *O. faveolata* | NOAA Key West Nursery |
| *V. coralliilyticus* MmMcT2-4 | *M. meandrites* | Big Pine Key, FL Keys  (24° 32'31.2" N  81°24'42.0" W) | *M. cavernosa* | NOAA Key West Nursery |
| ^1^*V. coralliilyticus* MCA25 | N/A | N/A | *M. cavernosa* | NOAA Key West Nursery |
| ^1^*V. coralliilyticus* MCA32 | N/A | N/A | *M. cavernosa* | NOAA Key West Nursery |
| ^1^*V. coralliilyticus* CN26H-1 | N/A | N/A | *C. natans* | NOAA Key West Nursery |
| ^1^*V. coralliilyticus* CN52H-1 | N/A | N/A | *C. natans* | NOAA Key West Nursery |
| ^2^*Vibrio* sp.  McD21-C1 | *M. cavernosa* | Sand Key, FL Keys  (24°27'14.4" N  81°52'39.0" W) | N/A | N/A |

^1^Isolated directly from an apparently healthy coral fragment; ^2^isolated directly from a diseased coral fragment; N/A =not applicable.
