# Supplementary material for: Disease Diagnostics and Potential Coinfections by Vibrio coralliilyticus During an Ongoing Coral Disease Outbreak in Florida
Source: Front Microbiol. 2020 Oct 26;11:569354. doi: 10.3389/fmicb.2020.569354 (PMC7649382; doi:10.3389/fmicb.2020.569354)
Supplement: Supplementary file 1 [file Data_Sheet_1.zip › S Files/Supplementary File (S2).pdf]

Supplementary File S2. Origin of coral hosts for the Florida *V. coralliilyticus* strains.

Strains	Diseased coral species	Diseased coral origin (GPS coordinates)	Healthy or infected coral species	Healthy or infected coral origin
V. coralliilyticus OfT6-17	*M. cavernosa*	Ft. Lauderdale, FL (26° 08'54.91" N 81°05'45.29" W)	*O. faveolata*	NOAA Key West Nursery
V. coralliilyticus OfT6-21	*M. cavernosa*	Ft. Lauderdale, FL (26° 08'54.91" N 81°05'45.29" W)	*O. faveolata*	NOAA Key West Nursery
V. coralliilyticus OfT7-21	*M. cavernosa*	Ft. Lauderdale, FL (26° 08'54.91" N 81°05'45.29" W)	*O. faveolata*	NOAA Key West Nursery
V. coralliilyticus MmMcT2-4	*M. meandrites*	Big Pine Key, FL Keys (24° 32'31.2" N 81°24'42.0" W)	*M. cavernosa*	NOAA Key West Nursery
[1]*V. coralliilyticus* MCA25	N/A	N/A	*M. cavernosa*	NOAA Key West Nursery
[1]*V. coralliilyticus* MCA32	N/A	N/A	*M. cavernosa*	NOAA Key West Nursery
[1]*V. coralliilyticus* CN26H-1	N/A	N/A	*C. natans*	NOAA Key West Nursery
[1]*V. coralliilyticus* CN52H-1	N/A	N/A	*C. natans*	NOAA Key West Nursery
[2]*Vibrio* sp. McD21-C1	*M. cavernosa*	Sand Key, FL Keys (24°27'14.4" N 81°52'39.0" W)	N/A	N/A

[1]Isolated directly from an apparently healthy coral fragment; [2]isolated directly from a diseased coral fragment; N/A =not applicable.
