# Supplementary material for: Disease Diagnostics and Potential Coinfections by Vibrio coralliilyticus During an Ongoing Coral Disease Outbreak in Florida
Source: Front Microbiol. 2020 Oct 26;11:569354. doi: 10.3389/fmicb.2020.569354 (PMC7649382; doi:10.3389/fmicb.2020.569354)
Supplement: Supplementary file 1 [file Data_Sheet_1.zip › S Files/Supplementary File (S4).pdf]

Supplemental File S4

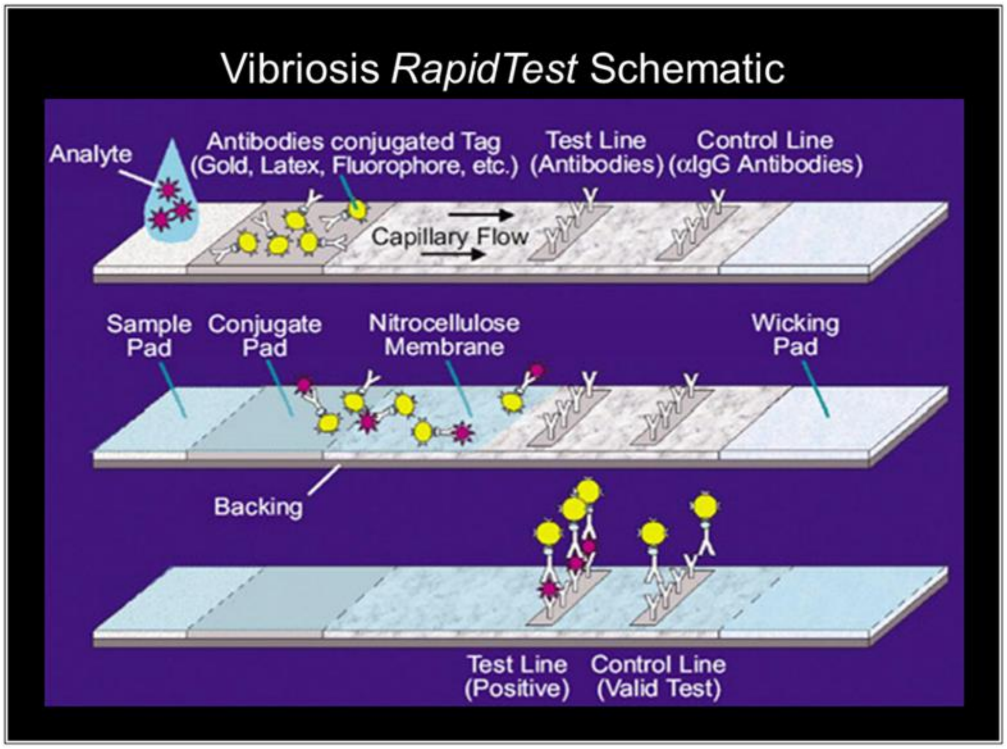

Supplemental Figure S3 is a schematic of the VcpA *RapidTest,* which is an improved and simplified version of a prototype 2-site immunoassay for VcpA previously described by (Gharaibeh et al., 2013). The prototype assay was successfully transitioned from a complicated lab-based test into a robust, highly sensitive, yet extremely simple diagnostic test, suitable for either lab or in-field use. Sensitivity and specificity of the prototype assay was improved by 1) reversing orientation of the VcpA-specific capture and detector monoclonal antibodies (mAbs), 2) optimizing sensitivity and stability of the nano-gold particle detector mAbs, 3) incorporating a proprietary blocking agent on the sample pad to reduce background to undetectable levels, and 4) addition of low protein binding sample filtration pad under the sample well to facilitate use of turbid samples without the need for extensive processing. Finally, the test was simplified greatly and made easy to use by incorporating all components into robust, single use plastic cassettes.

In summary, the VcpA *RapidTest* is robust (rugged, durable, and reliable), easy to use (simply add a few drops of unprocessed seawater to the sample port), rapid (≤20 minutes) and highly sensitive. The limit of detection (LOD) of purified recombinant VcpA by the current Vibriosis *RapidTest* is 5 ng/ml, which is a 100-fold increase in sensitivity over the LOD (500 ng/ml) of the prototype test and 20-fold below the concentration of VcpA that causes mortality of oyster larva under laboratory conditions (100 ng/ml, Hasegawa et al., 2009).

The VcpA *RapidTest* uses a pair of mAbs, both of which are specific for VcpA, but bind two different epitopes on VcpA. One mAb is used to capture soluble VcpA from a sample while the second mAb is used to detect the captured VcpA. The assay is therefore a 2-site

immunocapture/detector assay. The assay is termed a Lateral Flow Immunoassay (LFI) because samples flow laterally through a series of pads and nitrocellulose matrices. When a sample is loaded, it flows laterally through the conjugate zone, solubilizing gold-detector mAbs and then through the capture mAb zone. Capture mAbs immobilized on the Test Line bind to one epitope on a molecule of VcpA, while soluble detector mAbs (labeled with colloidal nano-gold particles) bind a second epitope on the same molecule of VcpA. If the target antigen is present in the sample, sandwiches of capture mAb:antigen:gold-detector mAb accumulate on the Test Line which then becomes dark and visible to the naked eye. Intensity of signal is proportional to antigen concentration.

A procedural control line is composed of a third set of antibodies that can specifically bind and immobilize gold-conjugated anti-VcpA capture mAbs directly, in either the presence or absence of VcpA. A positive signal on the control line therefore confirms that the test was conducted properly and that the sample flowed through the entire device.
