# Supplementary material for: Disease Diagnostics and Potential Coinfections by Vibrio coralliilyticus During an Ongoing Coral Disease Outbreak in Florida
Source: Front Microbiol. 2020 Oct 26;11:569354. doi: 10.3389/fmicb.2020.569354 (PMC7649382; doi:10.3389/fmicb.2020.569354)
Supplement: Supplementary file 1 [file Data_Sheet_1.zip › S Files/Supplementary File (S5).pdf]

Supplemental File S5

The **VcpA *RapidTest*** can be used to detect the shellfish toxin VcpA. VcpA is a metalloprotease secreted by a variety of vibrio species that are pathogenic for shellfish larvae and can cause shellfish vibriosis (Hasegawa et al., 2008, 2009; Hasegawa and Häse, 2009). Purified VcpA is toxic to oyster larvae (*Crassostrea gigas*) when present at concentrations greater than 100 ng/ml under laboratory conditions (Hasegawa et al., 2009). The Vibriosis *RapidTest* generates a positive signal if VcpA is present at levels of 5 ng/ml or higher and therefore may be useful to detect incipient infections when there is still time for effective treatment. The tests below were run using samples (150 µl) of artificial seawater spiked with varying amounts of recombinant VcpA (rVcpA). “C” indicates position of the procedural control line (a positive signal on “C” provides proof that the sample flowed correctly through the test zone). “T” indicates position of the Test line, and a positive signal on “T” is generated only when samples contain rVcpA at a concentration at 5 ng/ml and higher.

<u>Visual Reference Card</u>

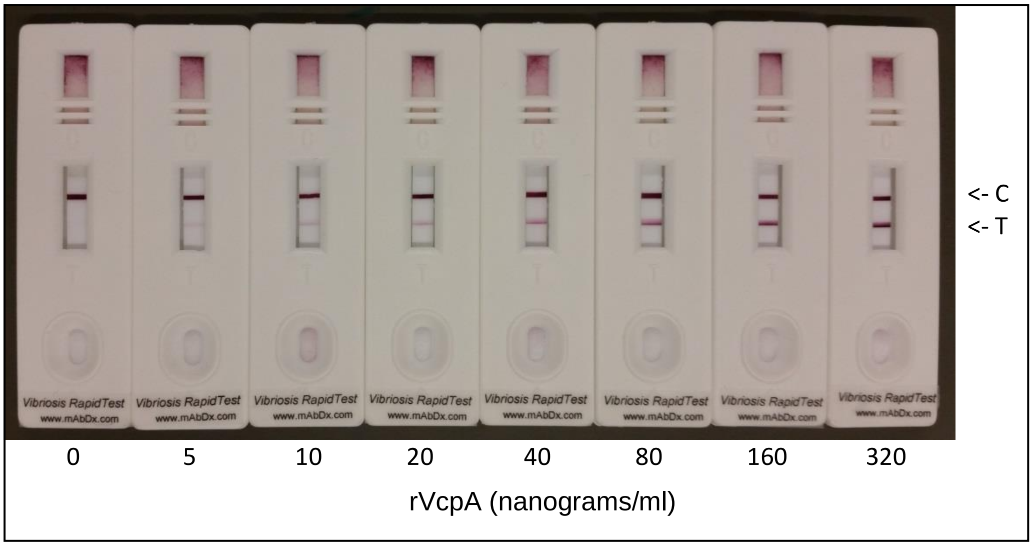

Sample Preparation:

*Most samples can be used “as is”, that is, straight out of a tank, tub, or faucet. Appropriate samples are: seawater, larvae-containing seawater, or algae-containing seawater. There is normally no need to pre-filter or otherwise clean up a sample before use. **NOTE: Freshwater samples cannot be used as they will generate “false positive” test results.**

*Some samples, such as compacted larvae collected by sieving, must first be resuspended in fresh seawater at a high larval concentration, e.g., 1 part larvae paste + 10 parts fresh seawater, and the suspension then incubated at room temperature for 1-2 hours.

Test Procedure:

NOTE: All steps are done at room temperature (~22°C).

1. Place a test cassette flat on a hard surface where it can remain undisturbed for 2 h. The cassette label, sample well and test viewing window should be right-side up as pictured above.

2. Load 4 drops (~150 µl) of a sample into the sample well.

3. Allow all the liquid sample to enter the device. **This should take at most 20 minutes.** Any debris in the sample will be trapped on the sample pad and will not affect subsequent steps or results.

4. Wash by adding 4 drops of clean freshwater (not seawater) to the sample well. Distilled water is preferred but high-quality tap water is suitable.

5. The test should develop as follows:

   -The viewing window will first become pink and will then clear, except for the “Procedural control” and “VcpA” lines.

   -The “Procedural control” line should change from its initial light blue color to a dark purple. **If the control line does not change from light blue to dark purple, discard the test because the sample did not flow properly. Similarly, the test should be discarded and repeated if the “vent” window remains clear and does not fill with purple dye. See above for examples of correctly processed tests.**

   -If VcpA is present in the sample, the VcpA test line will also become purple, similar in appearance to the developed control line. Samples with low levels of VcpA will generate faint VcpA test lines while higher levels of VcpA will generate progressively darker VcpA test lines.

6. Samples containing moderate to high concentrations of VcpA will generate a strong positive VcpA test line within 20 minutes. The concentration of VcpA in a sample can be measured by allowing the test to run for a total of 2 hours and then matching up the intensity of the sample’s VcpA test line with the intensity of a reference VcpA test line on the “Visual Reference Card”

above. If the intensity of a sample's VcpA test line is intermediate between two reference lines, the concentration of VcpA in the sample can be estimated as being intermediate to the same degree between the two reference values of VcpA.
