# Supplementary material for: Disease Diagnostics and Potential Coinfections by Vibrio coralliilyticus During an Ongoing Coral Disease Outbreak in Florida
Source: Front Microbiol. 2020 Oct 26;11:569354. doi: 10.3389/fmicb.2020.569354 (PMC7649382; doi:10.3389/fmicb.2020.569354)
Supplement: Supplementary file 1 [file Data_Sheet_1.zip › S Files/Supplementary File (S6).pdf]

Supplementary File S6

Laboratory diseased *M. cavernosa* (based on collection location)

	Total	Ft. Lauderdale	FL Keys
**# coral fragments**	**67**	**24**	**43**
**Disease stopped**	26 (38.8%)	8 (33.3%)	18 (41.9%)
**Slow progression over >21 d**	14 (21.0%)	7 (29.2%)	7 (16.3%)
**Complete mortality <21 d**	27 (40.3%)	9 (37.5%)	18 (41.8%)
**VcpA+**	16 (23.9%)	4 (16.7%)	12 (27.9%)
**Tissue loss only**	32 (47.8%)	8 (33.3%)	24 (55.8%)
**Tissue loss + Bleaching**	23 (34.3%)	14 (58.3%)	9 (20.9%)
**Bleaching only**	12 (17.9%)	2 (0.83%)	10 (23.2%)

*Percents are out of the total corals from a specific location (Total, Ft. Lauderdale, or FL Keys)

Laboratory diseased *M. cavernosa* (based on VcpA test)

	Total	VcpA-	VcpA+
**# coral fragments**	**67**	**53**	**16**
**Disease stopped**	26 (38.8%)	26 (49.0%)	0 (0.0%)
**Slow progression over >21 d**	14 (20.9%)	14 (26.4%)	0 (0.0%)
**Complete mortality <21 d**	29 (43.3%)	13 (25.5%)	16 (100.0%)
**Tissue loss only**	34 (50.7%)	21 (39.6%)	13 (68.8%)
**Tissue loss + Bleaching**	23 (34.3%)	20 (37.7%)	3 (21.4%)
**Bleaching only**	12 (17.9%)	12 (22.6%)	0 (0.0%)

*Percents are out of the total corals with a specific VcpA assay result (Total, VcpA-, or VcpA+)

Laboratory diseased *O. faveolata* (based on collection location)

	Total	Ft. Lauderdale	FL Keys
**# coral fragments**	**24**	**4**	**20**
**Disease stopped**	8<br>(33.3%)	2<br>(50.0%)	6<br>(30.0%)
**Slow progression over >21 d**	2<br>(0.8%)	0<br>(0.0%)	2<br>(10.0%)
**Complete mortality <21 d**	12<br>(50.0%)	2<br>(50.0%)	10<br>(50.0%)
**VcpA+**	5<br>(20.8%)	1<br>(25.0%)	4<br>(20.0%)
**Tissue loss only**	10<br>(41.7%)	2<br>(50.0%)	8<br>(40.0%)
**Tissue loss + Bleaching**	9<br>(37.5%)	1<br>(25.0%)	8<br>(40.0%)
**Bleaching only**	3<br>(12.5%)	1<br>(25.0%)	2<br>(10.0%)

*Percents are out of the total corals from a specific location (Total, Ft. Lauderdale, or FL Keys)

Laboratory diseased *O. faveolata* (based on VcpA test)

	Total	VcpA-	VcpA+
**# coral fragments**	**24**	**18**	**6**
**Disease stopped**	8<br>(33.3%)	8<br>(44.4%)	0<br>(0.0%)
**Slow progression over >21 d**	2<br>(0.8%)	2<br>(11.1%)	0<br>(0.0%)
**Complete mortality <21 d**	12<br>(50.0%)	6<br>(33.3%)	6<br>(100.0%)
**Tissue loss only**	10<br>(41.7%)	4<br>(22.2%)	6<br>(100.0%)
**Tissue loss + Bleaching**	9<br>(37.5%)	9<br>(50.0%)	0<br>(0.0%)
**Bleaching only**	3<br>(12.5%)	3<br>(16.7%)	0<br>(0.0%)

*Percents are out of the total corals with a specific VcpA assay result (Total, VcpA-, or VcpA+)
