# Supplementary material for: Disease Diagnostics and Potential Coinfections by Vibrio coralliilyticus During an Ongoing Coral Disease Outbreak in Florida
Source: Front Microbiol. 2020 Oct 26;11:569354. doi: 10.3389/fmicb.2020.569354 (PMC7649382; doi:10.3389/fmicb.2020.569354)
Supplement: Supplementary file 2 [file Data_Sheet_2.zip › S files2/Supplementary File (S10).pdf]

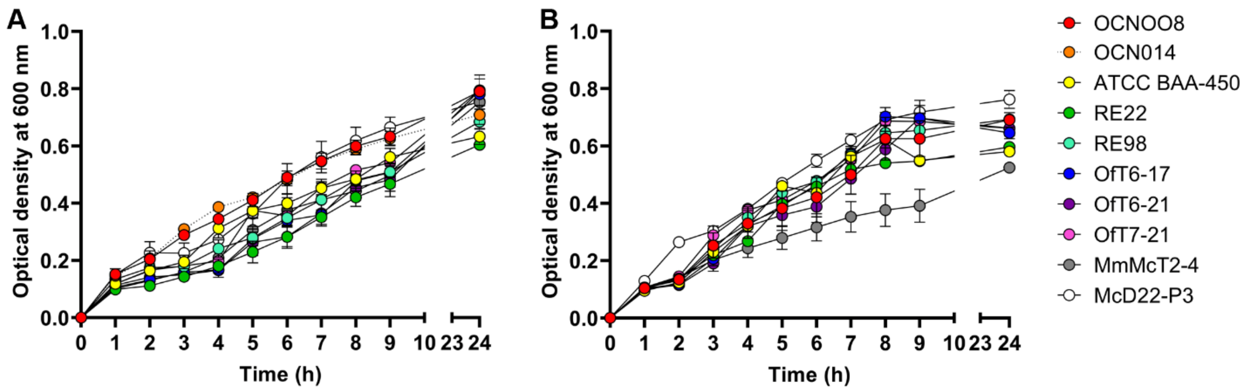

Supplementary File S10. Growth of various *V. coralliilyticus* at different temperature. The mean OD_{600} of various strains of *V. coralliilyticus* in SWB incubated at A) 23 °C or B) 29 °C. A total of 6 replicates were conducted for each strain and condition. The error bars represent the standard error of the mean.
