# Supplementary material for: Disease Diagnostics and Potential Coinfections by Vibrio coralliilyticus During an Ongoing Coral Disease Outbreak in Florida
Source: Front Microbiol. 2020 Oct 26;11:569354. doi: 10.3389/fmicb.2020.569354 (PMC7649382; doi:10.3389/fmicb.2020.569354)
Supplement: Supplementary file 2 [file Data_Sheet_2.zip › S files2/Supplementary File (S11).pdf]

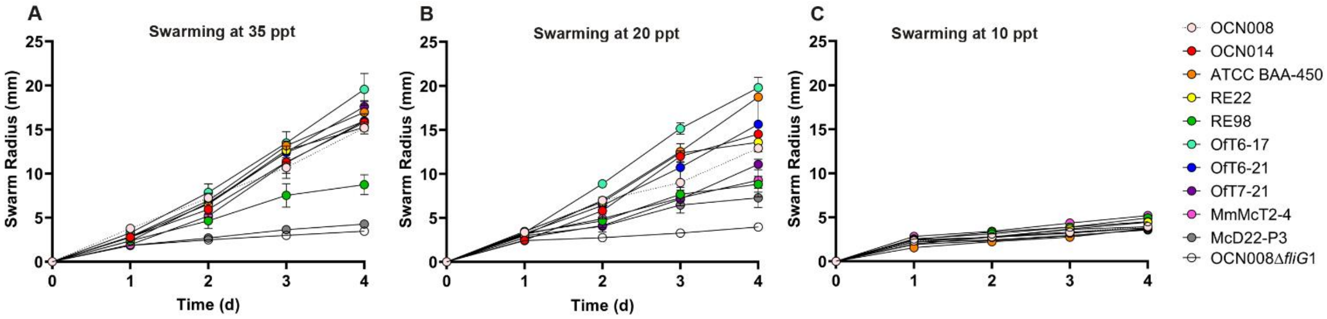

Supplementary File S11. The swarming radii of various *V. coralliilyticus* strains at different salinities. The mean swarming radii of various strains over a 4-day period were measured on SWA adjusted to A) 35 ppt, B) 20 ppt, or C) 10 ppt. A total of 3 replicates were conducted for each strain and condition. The error bars represent the standard error of the mean.
