# Supplementary material for: Disease Diagnostics and Potential Coinfections by Vibrio coralliilyticus During an Ongoing Coral Disease Outbreak in Florida
Source: Front Microbiol. 2020 Oct 26;11:569354. doi: 10.3389/fmicb.2020.569354 (PMC7649382; doi:10.3389/fmicb.2020.569354)
Supplement: Supplementary file 2 [file Data_Sheet_2.zip › S files2/Supplementary File (S12).pdf]

Supplementary File S12. API 20E results

API 20E Test	Activity	*V. coralliilyticus* OCN008	*V. coralliilyticus* OCN014	*V. coralliilyticus* BAA-450	*V. coralliilyticus* RE22	*V. coralliilyticus* RE98	*V. coralliilyticus* OfT6-17	*V. coralliilyticus* OfT6-21	*V. coralliilyticus* OfT7-21	*V. coralliilyticus* MmMcT2-4	*Vibrio* sp. McD22-P3
ONPG	β-galactosidase	+	-	+	+	+	+	+	+	+	+
ADH	Arginine dihydrolase	+	+	+	-	+	+	+	+	+	+
LDC	Lysine decarboxylase	-	-	-	-	-	-	-	-	-	-
ODC	Ornithine decarboxylase	-	-	-	-	-	-	-	-	-	-
CIT	Citrate utilization	+	+	-	-	-	-	-	-	+	-
H2S	Hydrogen sulfide production	-	-	-	-	-	-	-	-	-	-
URE	Urease	-	-	-	-	-	-	-	-	-	-
TDA	Tryptophan deaminase	+	+	+	+	+	+	+	+	+	+
IND	Indole production	+	+	+	+	+	+	+	+	+	+
VP	Voges-Proskauer (acetoin)	-	-	-	-	-	-	-	-	-	-
GEL	Gelatinase	+	+	+	+	+	+	+	+	+	+
GLU	Fermentation of glucose	+	+	+	+	+	+	+	+	+	+
MAN	Fermentation of mannose	+	+	+	+	-	+	+	+	+	+
INO	Fermentation of inositol	-	-	-	-	-	-	-	-	-	+
SOR	fermentation of sorbitol	-	-	-	-	-	-	-	-	-	+
RHA	Fermentation of rhamnose	-	-	-	-	-	-	-	-	-	+
SAC	Fermentation of sucrose	+	+	+	+	+	+	+	+	+	+
MEL	Fermentation of melibiose	-	-	-	-	-	-	-	-	-	+
AMY	Fermentation of amygdalin	+	-	-	+	-	+	+	+	-	+
ARA	Fermentation of arabinose	-	-	-	-	-	-	-	-	-	-
OX	Cytochrome-oxidase	+	+	+	+	+	+	+	+	+	+
