# Supplementary material for: Disease Diagnostics and Potential Coinfections by Vibrio coralliilyticus During an Ongoing Coral Disease Outbreak in Florida
Source: Front Microbiol. 2020 Oct 26;11:569354. doi: 10.3389/fmicb.2020.569354 (PMC7649382; doi:10.3389/fmicb.2020.569354)
Supplement: Supplementary file 2 [file Data_Sheet_2.zip › S files2/Supplementary File (S14).docx]

>NODE_1_length_6121_cov_2.441926

CTTCTACCTCCACCTAAATGACGATCTTGCTCTAATGTTTCATTACCAGCAATTCTGATTAGTGGTAGCTGATTATGATCAACAGAGAAGTGCCCCGACCTCCAACTAGCCCCGCCATCACGATAAACAGAGAACGTATAAGCAACACCTTTCTTGATAGATACTCTATAGTCATTATCAGTAATCGATCTGGTATACATTGCTACACGGTAGCTGGCTGTTGGGTGACCAAAACCAATACTGTGATATGTATATCCGTTGGTATCCACTTTTGGTGTAACAACAGTTGCATAAAATGTAGAGTGAGCTTTATAAACCCCATCCGGCATATCTGGTGAGTAAGTGTCGAATTCACTCCAATCAGCGTCTGCCATCAAGTTTTTGCCCAATGGTTGATCATTAGAATAAATGTCGACGTCAACGATATTGACAGGACCAACTAACACCGTACCCGTAACGCTCGTACCTGACGTTGGGTGTGTCGCTGTAACTAAAGTGTAACCTTCTGATAAGCTCGCGAACGATGTCACTCCACTTACAGTTCCTGCAACTGCAACAATCTGATCATCTAATACGTCCCAAGTAATCCCACTTGTATAGCCATCAACAGTCGTTCCATCACTGTAAACAGCTTGTAGTGAAAACGAAGCCATCTCACCTTTCGCTACACTAACAACTTGATCACCTAAGAATCTTAATTCTTCCAATACATTACCTGACACGGACACACTAATAGTCGAAGTCATTGATTCTAGTTGGATGCTGATATCAGCAGTGCCATCTGTTACACCATTAGCCAGTCCATTTGAAACCGTAACGATAGGTTCTTCACTACTATGCCAAGCTACATCTTGCGTAATGTCCCGCCAGTTACCATCACTTAGCAGCCCCATTGCAGAGAGTGCTAAGCTCCCACCAACTGGAATGCTTTCGCTGAGTGACGTTGATTGCTCAACATTGTTGTAGAAGATTCGAATGGCCGTTACCCAAGGATCTACCACTGAGATAGCAATGGCTTCACTTTCAATGCTGTCTTGTGCTGCCTTAAGCGTCTCAACCCCTTTACCTAGGATAACGATGTCTCCCGTACCTTGAGTAATATCGATAACACTATTTCCGTCGGTCAGCCAATTGACTGAATGGGTAATATCCACTTCAGTTGACGCGTCATCCGAGTAGATGCCGTAAGCTCTCACAGAATAGCCAGAACCGGTTACGGCCAGCAGTTGTTCATCCGCTTCAACACGAATCTCTGTCAATGCCGCATCGATAACTTCAATACTCAGCTCATCAGATACCACACCATTCGAGTTAGCCGACACGGTAGCAACACCCAGTGCATTCGCAACCAGCTCTCCATTGCTACCCGCGCTTAACGCGGTTATGTTATCAATTTGCCAAGAGAACTCTGAGGCCTGAAGAACTGACTTGGTACCGTCCGTGTAGAAAGCCGTAACTTCAGGTTTTGTTGAAGAACCAATCGGTAGCTCATCACTCCATGAACCGTCCGTCACAGCAATGTGAGTAACTTCTGCAGCGTCAATTTGAACTGTTAGTGTATTGGATACCTCTCCATCAAACCTCGCTTCAAGAGATACAGTACCTTGAGCTAGCGCCGTCAGTTGACCACGGTCATCGATAGAAACTGAAGACGCGTTGCCTACTGTCACCACCCATTGCGCCTGCATGGTGACATCTTGTGTACTACTGCCATAGTCTGCTTCAGCCGATAGGAGAACTGTAGATCCGACAGCCACATTAGAACTCGAAGCTGAGGCGTTCAAGCTATTGATGGTTAACCCATCATTCACTGAAACATCAACATTGCCTTGCACAACATTACCTTGTACATCAATGTATTCGGCAACCACGTTAGCCACACCACCAGCCTTACCGGTGACTAAACCGCTGCCATCCACATCGGCAATGGCAAGATGTTGACTATCAACTGACCACGTAACTCCGGTTGTCAGTACCGACGTTGACTGATCCGTAAATGTGCCAGTCGCCACCAACTGTAGTCTGGTTCCATTCGCGATCTGTTGCCCATGTGGAGTAACTGCAATACTGGTAAGTTCAGCATCTTTGACAGTGATTGGTAGCCCCAGGCTATCAATGCCCTGCATGTGCACACTAACGCTCTCAGTCCCCACACTTAGGCCACTGACGACACCGCTATTGGTAACATTAACCAGCGCTCGATCAGTACTCGACCAATCCACCATAGACGTAACATTTTTCGTACTCCCATCACTGAATGAACCGTAAGCCTCCAGTTGCTGCTGCATACCTTTCGCCAACTCCCAAGAAGCTTCTACTTGGCTATTAATCCGTATCTCTAAAGACACTAAGTCCTCATCAGTAACTGCAACAGGTAGTGTTGCTGTCTTACCAGATAGGCTCACAGTGAGTTCTGCTGTCCCTTCTGACTGTGCGGTAATCAAACCGTCAATCACAGTAAAGACCGCAGGCTTATCACTATCCCATGTTACTGAGTCTGTAACATCTCGACTGCCATTTGTGTACTGGCCAGTGACCTGAATCTGTTCAGACGTCCCTTCAGCTAGTACCACTTGGTTCTGTGTAAACACCAAGCGTTCCAGCGTTTCGTTAGTCACCGTCACACTGATGGAGTTACTTGATACAGAGTTATGAGTGAATGACAACGTCGTCGAGCCTTGATTATCTGCCACCAGAGTATTACCTGCCATGCTGGCTATGTTCGCCGGACTGAACACCCAGGTGATCTCTGAGTTCAAGAGTTCACGTTCACTGCCATCCGTATAACGCAGCATTGGCGTAAGCTCAGTTGACATTCCCTCGGGTAACGTGACATCACTTTGCTTGATGTAAAGCTGTTGCGCTTGTTTTTCAACAATACTGAGGTCCACTTCATTCGATGTCATCGACTGGAATTGAGCAGAAATAGACACGTCGGCTTGCGCTAATGTCTTCAGTTCTCCCAACCCATTGATATTCAGTGCTTGCTGATCACCAACAATGATCCAGCTGACCTGATGAGTCACATCTTGACGGGAATTGTCACTGAACACAGCTTCCGCGGTTACCTGACTCTGTTGACCAACCGCTAGGGAAGCGTCATTGAGATAGACGATGACATCAGTCAAAGTCGCTTCAGAGACAACCACAGCAGCACTGCCAAGCAGTTGACCAACGTTTGCACGCACATACGTACTGCCTTCAGCTTTCGCAGTCACCAAACCCGTCGAGCTATCAACATCTGCAATGAGTGGGTCAACAACGGACCATTGAACACCCGAAGTCAATTCATCGACTTGACCATCACTGTATGTCCCCCAAGCCTGGAAAACTTGCTGAGTGCCATTAGCAACTACTGGCATTGCTGGCGTCACCGAAATACTCTCCAGCACAGCAGGGTTTACCTTGACAGGGAACATTGGGCTATCTGTTGCACCAAAACGAGCGAACACACTTTCATCACCAGCACCGATACCTCGGATCACACCATTTTGACCAGCGTCGACAATCGTTGTTTCGGTAGAATGCCAAGTAACCAGCGACGTCATATTTTGCTGTGATTGATCACTGAAATACCCTGTGGCGATTAAAGGTTGCTCCGCGCCTATAGGAATCGTCGAGCTTGACTGCAAACCGTCTTCAGTTCGCACTTCGAGTCGATCCAGCTCTGCATCCGTCACCGCAACAGGTAGAGTTTCCGTTTGAGCCAGCAGAGTGACAGAAAGCGTGCCATTACCTTTAGTAAACCCTTCAACCAAGCCTGGCGTCGCTACATTAAATACTCCGGGCTCATCACTTGACCAAGTAACCTGATCCGTTACTTCTCGGCTTCCTGTAGTGAAGTGACCCGTGACGACAATCGGTAATGAGGTCCCTTTCGCCAACACCACTTGATTATGAGAGAATGACAGCCCTTGCAACTCTTCGCCCTCAACCGTCACTAGAATGGTCGAACTACTCTTACCTTGGTAGGTAAAGGACACTCGGCTGTCGCCCACTGACAATGCTGAATACTGGTTATCGGTAAAGCGAATAACCTGATCATCCTCATTCACCCATACTAACTCGTTGGCAGATAATGATCTTTCACTACCATCCGTATAACGTACTACAGGCTCAAGCAATTGAGATGTTCCAAGTGGAAGACTGGTGTCACCCTGTTTCAGGTACACTAAATCCACTGTTTTCACATCCACACGTAATGCTTCTTGGTTTGAGCCGATAGTATTTAGTGACGCACTGATAACCACATCACCTTCGCTAAGGCTTGACACCTCACCTAGGCCATTCACATGAACGGCCTGAGTGTCGCCACTAAGGTTCCATTGAACACTATTTGTCACATTCTGGGTTGTACCGTCACTGAAATTAGCAATGACATCAAGTTGAGCTTTCTCGCCTAACGCTATCAAACGAGAGTTAATAGCAAGGTCAAGGCTATCAAGCGTTGCATCCGTAACGGAAATCGCCGCTGAACCCGTCAGTGCACCCATCGGTGTATCGACTGTCGCTACAACAAAACCTGCACCTTGAGAAAGCCCAGTAACTAAACCATCACTGTCCACATCCGCAAGTAGAGGCTCAGTAAGAGACCAATTCACACCTGTAGTAAGAAGCTGCGAACTTCCATCCGTCATCGTCGCCCAAGCTTGATACTGCTGCGATGTGCCCTTCGCGAACTGACCAATGGCAGGCGTAACAGAAATGCGACTGAGTTCCTCCGCTATTACATTAACGCTCACATTGGAGACCTGAGAATCGAAATACGCAGAAATCGTGGCTGTGCCTTGAGCTAACGCTTGCGTCCCTCCACTCTGGTCAACATCCGCAACATTCACATTATCGGTTTGCCAAACTACCAAGCTTGATAAATCTAGCTGAGTGCCATCGCTATAGTGCCCCATTGCCTGAAGGGAAGTTGTTAACCCTTTAACTAAGGAAACATCTTGAACTTCAGTGTTACCTTGGTGCATTGAAATACGCAATAGATCGGCATCCGTCACCGCTACCGGCAATAGTGCTTGTTTACCGTTCATAGAGACGGTTATGTTACCGGTACCTTCAGCTTGTGCCGACAGTAAACCTGTATTGCTTATCGTAAACGCTGATGGATTAGTGCCTGACCAAGCAACCGAACCTGTCACCTCTCGGCTGCCATTAGTAAAGTGTCCGGTAACCTCCAACTGTTGAGCAGTTCCTTGAGGTAGCACAACCTGAGCACTATCAAAACTGAGCGTCAACAATTGAGCAGGGTCGACACCAATTTGCAGACTGTTACTTGTTTGAGCATCGAACTCAAATGAAACCGTCGTCGCTCCGGTAGCTGTTGCTGTTAATGTATTTGCACCGATATCAACAAACTGACTATCGTATACCCAGGTAACTTCCTGAGCACCTAGCACACGCTCGCTATCATCGCTGTACCTAACGGTTGGATCCAAGCTGAGCTGAGCACCAACTGGCAATATTTGATCACCATGACTAAGGTAGAGCGACTGAACGACCTTAGCTGAAACTTGAATGCCAGCGACGTTGGATACCTGTCCAAGGTACACCGCACTGATTGAAACCTGCCCCTCCGCTAAGCTCGATACTGAACCTAGCTGATCAATAGATATCGCTTGTACATCACCAGATGTGTTCCACTGAACTTGCTTGGTCACATCAACATTTCTGCCATCAGTGAATGTAGCAATGGTGGTTAGCTGGCTCTCTTGACCCAATGCAAGGCCACTCTCAGATACGGTTAGCGTTAAGCGCTCTAATGCTGCGTTTGAAACTGAGACAGCTGCTTGACCTTCGACTGAATTTTGGCGAGCCACTACTTGGCTGGTGCCTTCTTCTTTCGCTTGAACGAGGCCGCTAATATCAACAGCCACCACATCAGGCTGAGTGACAGACCAGTCAATACCAGTGCTCATCAGGTAAGATTTACCATCTGAGTATAGCCCCCAGGCCTCTAGCAATTGGCTCGTTCCTTTGGCAAGTAAACTGTTCACTGGCGTGACAGTTAAGCTCTGGAGTATGGCATCGGCGACTTTATAGTTTAGGTCAGAGCTGCTTATTCCTCCCCAGTGCGCACTCACTTGCTCTTCTCCAGCTTGTAGGCCTGTCACTACCC

>NODE_2_length_6036_cov_2.563182

CAGTTTGTCTTTGCTGCTGATAAAGCCATGCAGAGCGAATTAGATACTCGCCTCAATGATGCCAATATTGAAGACTTGCTGACCTTGATTTACACCTCAGGTACGACGGGTCAGCCAAAAGGGGTAATGCTGGACTATGGCAATGTCGCCGCTCAACTCGAAGGTCATGACGCCCGATTAAGTTTGTCTCAAGATGATGTTTCGCTCTGTTTCCTACCTTTATCACACGTGTTTGAGCGAGCATGGACATTCTACGTATTGTATCGTGGTGCTACCAACTGTTACCTGCAGGATACGATGCAGGTTCGTGAAGCGTTGAGTGAAGTTCAACCGACAGTGATGTGTGCGGTTCCACGTTTTTACGAGAAAATCTTCTCAGCTATTCATGAAAAAGTCTCCAAGGCTCCGATGATTCGCAAGGTGCTGTTCACCTGGGCTGTGAATATGGGCGCTAAAATGGCCGCCTGTGATCAGGAAGGGCGTCAACCCTCTTTAATATTGAAAAAGTCCCATCAGCTGGCTGACAAGTTGGTGCTGTCCAAGCTACGCGCTCTACTGGGTGGTCGAATTAACTTTATGCCATGTGGTGGTGCGAAGTTGGATGAGACGATTGGTCGCTTTTTCCATGCGATGGGAATCAACGTCAAGCTTGGCTATGGCATGACAGAAACGACAGCGACGGTGTCGTGTTGGTCAGACAATAGCTTCAACCCAGATTCCATAGGTATGGCGATGCCTGGTGCACAGGTAAAGATTGGCGAAAACAACGAAATTCTCGTCCGTGGCCCTATGGTGATGCGTGGTTACTACAAGATGCCGGAAGAAACGGCAAAAACGTTTGATGAGCATGGCTTCCTCAAAACTGGGGATGCGGGTCATATTGATGAAGACGGCAACCTGTTTATTACCGATCGCATTAAAGAGTTAATGAAAACGTCTGGTGGTAAGTACATTGCACCGCAGATGATCGAAGGAGCAATCGGTAAAGACCACTTTATCGAGCAGATCGCCGTGATTGCGGATACGCGTAAGTTTGTTTCTGCGCTTATTGTGCCGTGCTACGACAGTTTGGAAGAGTACGCGAAAGAGTTGAACATCAAATACCATGATCGTGTTGAGCTGATTAAGCATCATCAAGTGGTAGAAATGCTCGAAAAACGAGTGAATGACCTGCAGCAAGAGCTGGCGAAGTTTGAGCAGGTTAAGAAGTTCAAATTGTTACCAAAAGCCTTCTCGATGGATGAAGGTGAATTAACCCCGACTCAGAAACTGCGCCGTAAGGTTATTAACGACAAATATCAGGACGAAATCGAAGAAATGTACAGTGATACAGCAATGAAAAAGTAAATCTCTGACAAGTTAAATTTCGAAGTGTTTAAAAATCCCTCAATCGGTTCAAACTTGCCGGTGAGGGATTTTTTATACCTGAATTTTACGACCTTTTACGGTGAAAATATCGTGGAGATACAGTGTTGAATAATATTTTACTCTGAATTTTTTGGGTTAATTTGTCATATTTTCTGATTTTTATGCTTCGGCTGGTGTTCTTTCCTTATATTAGTTTGATAAATAGCATCTTGTTTAAAATGAGAGTTAAGTCGCATTAGATCCTTTGATAAGAAAACGTTACATTTGTTGCGTGAACTTGTTTTCTGTTACGACAGAACAAGACATAGCCGAAAAAGTGGAAGTATTTCGATTTAGGCTACGAATAAGCCCTAGACTATGTAAAACCAGCCCAATTAGTTGACCTTATTAATTGGTAAACTGGAGAGGAGAATAATATGGCAGCAATGTTGTCCGGTGCGGAGATGGTTGTTCAATCTCTGATCGAAGAAGGTGTTGAACAAATTTTTGGGTATCCAGGCGGTTCTGTTTTGGATATCTACGATGCTCTTCATGCTAAGACAGATCAAATCAAGCACGTCCTAGTTCGTCATGAACAAGCCGCTACCCATATGGCAGATGGCTATACTCGTGCAACTGGCAAACCAGGCGTGGTTCTGGTGTGCTCAGGTCCAGGCGCGACCAATACTGTGACAGGTATTGCGACCGCGTATATGGATTCTATTCCTATGATCGTGATTTCAGGTAACGTGCCTAACAACCTGATTGGCAATGACGCTTTCCAAGAGTGTGATATTGTCGGCGTCTCGCGTCCGATTGTTAAACACAGCTTCTTGGTAAAGAAAGCAGAAGATATTCCTGAAATCATGAAGAAAGCTTTCTATATTTCGACAACAGGGCGTCCTGGCCCGGTTGTTATCGATCTGCCCAAAGATGTGATGAATCCGCAGATCAAACTGCCATACGAATATCCTGCCAGTATTTCAATGCGTTCATATAAGCCAACCACGACGGGTCACAAAGGCCAGATCAAAAAGGCCTTGAAAGCACTACTTGCGGCGAAAAAGCCGGTGCTTTACGTCGGTGGCGGCGCCGTAATTTCTGAAGCGGATGAGCAGATTTTGAAGCTGTCAGAGGCATTAAACTTACCTGTCGTTAGTACCTTGATGGGGCTAGGTGCCTTCCCGGGTACGCATAAAAACTCTCTTGGTATGCTGGGGATGCATGGTGTGTATGAGGCCAATATGGCCATGCACAATGCAGACTTAATCTTTGGTATCGGTGTGCGATTTGATGATCGAACCACCAACAATCTGGAAAAGTATTGTCCGAATGCCAAAGTAATGCATATTGATATTGACCCGTCATCTATCTCGAAGAACGTCAAAGCAGACTTGCCTATTGTTGGCTCTGCAGAGAAAGTACTGGCGAGTATGGTGTCACTGCTGGAAGAGCAAGGTGCTACCAATGATGGCGAAGCACTTCAAAGCTGGTGGGATGATATCCAGACTTGGCGTGACCGTGAGTGCCTGTCTTATGAATCCACGCCTGAGCGAATTAAACCGCAACAGGTGATTGAAGTACTACACAAGCTGACGAATGGCGATGCGTACGTTGCTTCTGACGTTGGTCAGCACCAGATGTTTGCAGCACTTTACTATCCGTTTAATAAACCACGTCGCTGGATTAATTCTGGTGGTCTCGGCACGATGGGCTTCGGGCTGCCAGCTGGCCTTGGGGTTAAATTTGCTAAGCCTGATGAAGAGGTGGTAGTGGTGACCGGTGATGGTAGTATCCAGATGAACATTCAGGAACTGTCGACGGCGCTGCAATATGACATTCCAGTCAAGATCATTAACCTGAATAACCGTTTCCTAGGTATGGTGAAACAGTGGCAAGACATTGTTTACCAAGGAAGACATTCTAACTCATATATGAGCTCCGTTCCGGACTTCGTTGCGATTGCAGAAGCGTACGGACATGCGGGGATTCGCATTGAAACTCCAGACCAGCTTGAGTCTGGTTTGAAACAAGCATTAGAAATGAAAGATCGTTTAGTGTTTGTTGATATCAATGTTGATGAAACTGAGCATGTGTACCCAATGCAAATCAAAGGCGAAGGTATGGACAAGATGTGGCTAAGCAAAACGGAGAGAACATAATATGAGACACATTATTTCACTGCTAATGGAAAACCAACCGGGCGCATTATCCCGAGTGGTAGGTCTGTTCTCACAACGTGGTTACAACATTGAGTCGTTAACCGTATCACCGACAGATGATGAAACGCTGTCGCGTTTGAACATCACAACGATTTCGGATGACATGCAGCTTGAACAGATTCAGAAGCAGCTTAATAAGTTGATTGATGTACTGAAAGTTCAGGAAGTCACCGAGCTTGATCATATTGAGCGTGAGCTGATGATGGTTAAAGTGAAAGCCAGTGGTTTTGCTCGTGCAGAAGTAAAGCGTACAGCGGATATCTTCCGTGGACAGATTGTTGATGTCACCGCTTCTCAATACACGGTTCAACTGGCGGGTACCAGTGAAAAGCTGGATGCGTTCATTCAAGCTATTTCAGAAGTAACTGATGTCATTGAAGTTGCGCGAAGCGGTGTGGTGGGGATTGCACGCGGTGAGCGTTCACTGAAGCCTTAACTTGATATTAGTTGTTAAAGAAAAGCCTTAGTCATGGACTAAGGCTTTTTTGTTTATGGGCTATTTGTTGAGAAAGCTAACTGCTTTGTCTGGGAAATCGGTAAACAGGCCATCTACTTTGACCTGATTGTAGAAGATATCCATCATGCCCTCGAAGTTATCCGTATAACTTGGAATACGGCCTGGATCTGCTCGGAAAGTGTATGGATGAACATCTAGGCCTGCTGCTTTTGCCTCTTTCATCAACGGTTTGATAATAATGTTGTCTTTGGTTGATTCATCATCGACCAACATAGGTTTCCAAGGACCAATGCCTTCGGCGTACTTGGCAACTTCCTTCATTGCGCCTGATTCGAACATCCAATCATAGCTGTACGGGGTTGCCGTATCGCCTTTGTAGACCATGGTTTCGTTCCAGTCAGTGTAAGCCATCAGCTGCACTAACTTGAGATCCATATCCATACTAGGCATGAGTTCATCGTTAATGCGTTTGAGCTCATTGGCGTCAAAGCATTGCAGGTATACTTTGTCATCCTGACTGTCATAGCCGTACTGCTTTAATAGCTTAAGCACTGCGGAAGAGATGTCTTTGCCTTCATGACGGTGGAACCAAGGGGCTTTGATTTCAGGGTAGATACCAATATCGTAACCAAGCGTTTTGTTGAGCCCTTGAATCAGCTCAATTTCTTCAGCCAGAGTAGGGACGCTGAAATCGGATTTCCACATCGGAAAACGAGTCGGATAACCCGCAACTTTATTGCCATTGTCGTCAATATTGAACCCTTCTGTCACCTTTAGGGTTTTGATTTCTGCAAGCGTAAAGTCAATTGCATAATAGCGGCCATCGGCGCGAGCACGATCAGGGTAGCGCTCAGCGACATCGGTGACTCGATCTAAATAATGATCGTGGAGAACAACCAACTGATCGTCTTTAGTCATGACAACATCTTGCTCGATGTAATCCGGTTTCATTGCGTAAGCCAAAGCTTTTGCTTCTAGAGTATGTTCTGGTAGATAGCCTGATGCGCCGCGGTGAGCGATAACCAGAGGCTTAGCCAATGTTCCAGCAGATAGGCTAAGAGCCAGAAATGTAGCGCAAACTGACGTTGTTTTCATTCTTCATTCCTTGATTGAAATGAGGCAGCGATGCTGCCTCTTTGATGAGTATTAAGCGAGTGCTGCCTTTTCGGCTTCTTTCTGTTTATGGTGAGCACGTTCGCCAACAAATGCATAAATTAGACAAATAATCGACGCGATACAAGATGCTACGAGAATGATAAATCCACCATCCCAGCCAAAGTGATCCACGGTGTAACCCAGAATCGCGTTCGCAGCCACTGCACCGCCAAGATAACCAAATAGTCCGGTTAGACCAGCAGCGGTACCTGCGGCTTTTTTCGGTGCAAGTTCAAGAGCATACAAACCGATCAACATAACCGGACCGTAAATCAGGAAGCCAATGGCAACCAGCGCCATCATGTCTACGGCAGGGTTACCGGCTGGGTTGAACCAGTAAACCAAGACCGCAACTGTCACTAAGGCCATAAACAGGATACCGGCTGGGGCGCGACGACCTTTGAACAGCTTGTCGGAAATCCAACCACACAATAAGGTGCCTGGAATACCCGCCCATTCGTAAAGGAAGTAAGCCCATGAAGATTTATCAACCGTGAAGTCTTTCGCTTCTTTGAGGTAAACCGGCGCCCAGTCAAGCACACCGTAGCGAATGAGATAGACAAACGCGTTCGCAATGGCGATTGACCAGAGTAGCTTGTTAGAAAATACGTACTTAAAGAAGATTTCTTTGGCGGTCATTTCCTTTTCATGCGATTTATCGTAGTCGTCTGGGTAGTCGTTTTTGTATTCTTCAATGGGTGGTAAACCGCAGGATTGTGGTGTGTCGCGAACTGTCATCCAGATAAATATGGCAACCAGCGTGGCGAAAAAAGCAGGAACATAAAAAGCGGTACGCCAGTCGTCATTAAATGCCCATAGCCCAAGGATGAAGAGAGGACCAATCAGGCCTCCGCC

>NODE_3_length_5393_cov_20.743040

ACGAAGTGACCATTGAATCTTCGGATTCAGCACAGTCAATTCAAACATTACTTATGTAATGTTCAGTATTCATTGAGCCGACAAAATCTTAAATTGAAGAGTTTGATCATGGCTCAGATTGAACGCTGGCGGCAGGCCTAACACATGCAAGTCGAGCGGAAACGAGTTATCTGAACCTTCGGGGAACGATAACGGCGTCGAGCGGCGGACGGGTGAGTAATGCCTGGGAAATTGCCCTGATGTGGGGGATAACCATTGGAAACGATGGCTAATACCGCATAATAGCTTCGGCTCAAAGAGGGGGACCTTCGGGCCTCTCGCGTCAGGATATGCCCAGGTGGGATTAGCTAGTTGGTGAGGTAATGGCTCACCAAGGCGACGATCCCTAGCTGGTCTGAGAGGATGATCAGCCACACTGGAACTGAGACACGGTCCAGACTCCTACGGGAGGCAGCAGTGGGGAATATTGCACAATGGGCGCAAGCCTGATGCAGCCATGCCGCGTGTATGAAGAAGGCCTTCGGGTTGTAAAGTACTTTCAGCAGTGAGGAAGGTGGTAGTGTTAATAGCACTATCATTTGACGTTAGCTGCAGAAGAAGCACCGGCTAACTCCGTGCCAGCAGCCGCGGTAATACGGAGGGTGCGAGCGTTAATCGGAATTACTGGGCGTAAAGCGCATGCAGGTGGTTTGTTAAGTCAGATGTGAAAGCCCGGGGCTCAACCTCGGAATTGCATTTGAAACTGGCAGACTAGAGTACTGTAGAGGGGGGTAGAATTTCAGGTGTAGCGGTGAAATGCGTAGAGATCTGAAGGAATACCGGTGGCGAAGGCGGCCCCCTGGACAGATACTGACACTCAGATGCGAAAGCGTGGGGAGCAAACAGGATTAGATACCCTGGTAGTCCACGCCGTAAACGATGTCTACTTGGAGGTTGTGGCCTTGAGCCGTGGCTTTCGGAGCTAACGCGTTAAGTAGACCGCCTGGGGAGTACGGTCGCAAGATTAAAACTCAAATGAATTGACGGGGGCCCGCACAAGCGGTGGAGCATGTGGTTTAATTCGATGCAACGCGAAGAACCTTACCTACTCTTGACATCCATAGAACTTTTCAGAGATGGATTGGTGCCTTCGGGAACTATGAGACAGGTGCTGCATGGCTGTCGTCAGCTCGTGTTGTGAAATGTTGGGTTAAGTCCCGCAACGAGCGCAACCCTTATCCTTGTTTGCCAGCGAGTCATGTCGGGAACTCCAGGGAGACTGCCGGTGATAAACCGGAGGAAGGTGGGGACGACGTCAAGTCATCATGGCCCTTACGAGTAGGGCTACACACGTGCTACAATGGCGCATACAGAGGGCGGCCAACTTGCGAAAGTGAGCGAATCCCAAAAAGTGCGTCGTAGTCCGGATTGGAGTCTGCAACTCGACTCCATGAAGTCGGAATCGCTAGTAATCGTAGATCAGAATGCTACGGTGAATACGTTCCCGGGCCTTGTACACACCGCCCGTCACACCATGGGAGTGGGCTGCAAAAGAAGTGGGTAGTTTAACCTTCGGGGGGACGCTCACCACTTTGTGGTTCATGACTGGGGTGAAGTCGTAACAAGGTAGCGCTAGGGGAACCTGGCGCTGGATCACCTCCTTATACGATGATTACTCACGATGAGTGTCCACACAGATTGATGGTTTATGTAGTTTAAGAGACGATACTGGGTCTGTAGCTCAGGTGGTTAGAGCGTTCGCCTGATAAGCGAGAGGTCGGTGGTTCAAGTCCACTCAGACCCACCAATCTTCCTCCCAGGAGATTGGCAACAGTATCGACACCTTGATGGGGCTATAGCTCAGCTGGGAGAGCGCCTGCCTTGCACGCAGGAGGTCAGCAGTTCGATCCTGCTTAGCTCCACCATCTTTAAGTGTTCTTGAAAAAGAATCTTTAAAAATGGTTCATTTATGAATCTAGCTCTTTAACAATTTGGAAAGCTGACGAATAACAACAATCCCCATCTCTATGAGATGCGTTGTTATTCATTGAAAAGTTCTCAAATCCTAAATCTTTTGATTTAGGTACCAACACACATTCAAGTGTTCTTGGCTTTTTGTTTTCACTTTTTATAAAAAAAGTAAAGTAAAAAGATATTTGAGTCCGGCAAAATCGAGTCTGCATCATGTTTAAATAATTGCAGACAACTTTGGTTGTTTGACGTAAAGACCCTTTGGGGTTGTATGGTTAAGTGACTAAGCGTACACGGTGGATGCCTTGGCAGTCAGAGGCGATGAAGGACGTATTAACTTGCGATAAGCCCAGATTAGACAGTAAAAGTCATTTGAGTCTGGGATTTCCGAATGGGGAAACCCACCTGCATAAGCAGGTATCATTATCTGAATACATAGGGTAATGAGGCGAACCGGGGGAACTGAAACATCTAAGTACCCCGAGGAAAAGAAATCAACCGAGATTCCGAAAGTAGCGGCGAGCGAAATTGGACTAGCCCTTAAGCCTTTAATGCGTCAGGTGAAAGTTCTGGAAAGTTCTGCGATACAGGGTGATAGCCCCGTAACCGACAGCGCATTTTAGGTGAAATCGAGTAGGGCGGGACACGTGATATCCTGTCTGAATATGGGGGGACCATCCTCCAAGGCTAAATACTACTGACTGACCGATAGTGAACCAGTACCGTGAGGGAAAGGCGAAAAGAACCCCTGTGAGGGGAGTGAAATAGAACCTGAAACCGTGTACGTACAAGCAGTAGGAGCACCTTCGTGGTGTGACTGCGTACCTTTTGTATAATGGGTCAGCGACTTATATTCAGTAGCAAGGTTAACCATCTAGGGGAGCCGTAGAGAAATCGAGTCTTAACTGGGCGTCGAGTTGCTGGATATAGACCCGAAACCAGGTGATCTAGCCATGGGCAGGTTGAAGGTTGAGTAACATCAACTGGAGGACCGAACCGACTAATGTTGAAAAATTAGCGGATGACTTGTGGCTAGGGGTGAAAGGCCAATCAAACCTGGAGATAGCTGGTTCTCCCCGAAATCTATTTAGGTAGAGCCTCGGACGAATACTACTGGGGGTAGAGCACTGTTAAGGCTAGGGGGTCATCCCGACTTACCAACCCTTTGCAAACTCCGAATACCAGTAAGTACTATCCGGGAGACACACGGCGGGTGCTAACGTCCGTCGTGGAGAGGGAAACAACCCAGACCGCCAGCTAAGGTCCCAAATTACAGCTAAGTGGGAAACGATGTGGGAAGGCTTAGACAGCTAGGATGTTGGCTTAGAAGCAGCCATCATTTAAAGAAAGCGTAATAGCTCACTAGTCGAGTCGGCCTGCGCGGAAGATGTAACGGGGCTAAGCTGTAAACCGAAGCTGCGGCAATGCATTTTATGTATTGGGTAGGGGAGCGTTCTGTAAGCCGTTGAAGGTGAGTTGTAAAGCTTGCTGGAGGTATCAGAAGTGCGAATGCTGACATGAGTAACGATAAAGGGGGTGAAAAACCTCCTCGCCGGAAGACCAAGGGTTCCTGTCCAACGTTAATCGGGGCAGGGTAAGTCGACCCCTAAGGCGAGGCCGAAAGGCGTAGTCGATGGGAAACGGGTTAATATTCCCGTACTTCTTACAATTGCGATGGGGGGACGGAGAAGGCTAGGTGGGCCTGGCGACGGTTGTCCAGGTTCAAGTGCGTAGGCTTGAGAGTTAGGTAAATCCGGCTCTCTCTAAGGCTGAGACACGATGTCGAGCACCTACGGGTGTGAAGTCATTGATGCCATGCTTCCAGGAAAAGCCTCTAAGCTTCAGATTGTAAGGAATCGTACCCCAAACCGACACAGGTGGTCGGGTAGAGAATACCAAGGCGCTTGAGAGAACTCGGGTGAAGGAACTAGGCAAAATGGTACCGTAACTTCGGGAGAAGGTACGCTCCTGTCGGTGAAGTCCCTTGCGGATGGAGCTAACGGGAGTCGCAGATACCAGGTGGCTGCAACTGTTTATTAAAAACACAGCACTGTGCAAAATCGTAAGATGACGTATACGGTGTGACGCCTGCCCGGTGCCGGAAGGTTAATTGATGGGGTTAGACGTAAGTCGAAGCTCTTGATCGAAGCCCCGGTAAACGGCGGCCGTAACTATAACGGTCCTAAGGTAGCGAAATTCCTTGTCGGGTAAGTTCCGACCTGCACGAATGGCGTAATGATGGCCACGCTGTCTCCACCCGAGACTCAGTGAAATTGAAATCGCTGTGAAGATGCAGTGTACCCGCGGCTAGACGGAAAGACCCCGTGAACCTTTACTACAGCTTGGCACTGAACATTGACCCTACATGTGTAGGATAGGTGGGAGGCTTTGAAACCGGTACGCCAGTATCGGTGGAGCCGTCCTTGAAATACCACCCTTGTAGTGTTGATGTTCTAACTTAGACCCGTTATCCGGGTTGAGGACAGTGCCTGGTGGGTAGTTTGACTGGGGCGGTCTCCTCCCAAAGAGTAACGGAGGAGCACGAAGGTGGGCTAATCACGGTTGGACATCGTGAGGTTAGTGCAATGGCATAAGCCCGCTTGACTGCGAGAATGACAATTCGAGCAGGTGCGAAAGCAGGTCATAGTGATCCGGTGGTTCTGTATGGAAGGGCCATCGCTCAACGGATAAAAGGTACTCCGGGGATAACAGGCTGATACCGCCCAAGAGTTCATATCGACGGCGGTGTTTGGCACCTCGATGTCGGCTCATCACATCCTGGGGCTGAAGTCGGTCCCAAGGGTATGGCTGTTCGCCATTTAAAGTGGTACGCGAGCTGGGTTTAGAACGTCGTGAGACAGTTCGGTCCCTATCTGCCGTGGGCGTTGGAAGATTGAAGGGGGCTGCTCCTAGTACGAGAGGACCGGAGTGGACGAACCTCTGGTGTTCGGGTTGTCATGCCAATGGCATTGCCCGGTAGCTAAGTTCGGAATCGATAACCGCTGAAAGCATCTAAGCGGGAAGCGAGCCCTGAGATGAGTCTTCCCTGACCCCTTGAGGGTCCTAAAGGGTTGTTCGAGACTAGAACGTTGATAGGCAGGGTGTGTAAGCGTTGTGAGGCGTTGAGCTAACCTGTACTAATTGCCCGTGAGGCTTAACCATACAACACCCAAAGGGTTTTGATGGACTCAAAGCAAGAACTTTGAATGTGTAAATACAGAACTTAAAAACAGCTTTCCGAATTATTCGATTTGCAAGCAAATCTCACCCTAAAGGGCCGCACTAAAGTGCGTTTGAATTTGCGAGCAAATTGATAAAGAATTTGCTTGGCGACCATAGCGTTGTGGACCCACCTGATTCCATGCCGAACTCAGAAGTGAAACGCAATAGCGCCGATGGTAGTGTGGGGCTTCCCCATGTGAGAGTAGGACATCGCCAGGCTT

>NODE_4_length_5207_cov_2.196491

GGATACCCCTGTTGTGGTCTGGCTTAAAATCGATAGTGGCATGCACCGTCTCGGGGTCAGACCGGAGCAATACACGGACTTTGTCTCTCGCCTCAAGGCGTGTCGTAATGTCGCTAAGCCACTGCGTTATATGAGTCATTTTGGTTGTGCTGATGAGTTGGATAAGTCCACTACGACAGAACAAACTGAATTGTTCTTGGCCCTTACCGATGGTTGTGAAGGAGAGCGTTCACTGGCGGCTTCTGCGGGCTTGCTGGCTTGGCAGAACAGTCAACTCGATTGGGTTAGGCCTGGAATTATCATGTATGGTGTGTCGCCTTTTAACGATAAAACGGCACAGCAGATGGGCTATCAACCAGTCATGACGCTAAAGTCGCATCTCATTGCGGTGCGTGACGTAAAAGCTGGTGAGAGCGTCGGCTATGGCGGGATGTGGACCAGCCAGCGTGACACCAAAGTAGGTGTTATTGCTATTGGTTACGGTGATGGCTACCCGAGAACTGCACCTAATGGCACCCCAGTGTTAGTCAATGGTCGCAAAGTACCGATTGCGGGAAGAGTGTCGATGGACATGTTAACGGTTGATCTCGGTCCTGATGCCAATGATAAGGTGGGGGATGAGGCAATCCTTTGGGGACAAAGTCTTCCTGCTGAAGAGGTGGCCTGCCATATTGGCACCATTGCTTACGAATTGGTGACTAAACTGACCTCGCGAGTTGAAATGGAATATTCCAAATAGCTCACATCGAACCTTTCGTTTTAGGCTGACTTATTAAGTCAGCCTTTTTATTATTCGCCCTGAATCGTCGCAACTAGGGTGCGGCTTCCGCCATAATCACGATGCTCTCCAAGGTATATTCCTTGCCATGTCCCTAAAGCTAGTCGTCCGTTGCTAATGGGTATCATCACGCTGCACCCTAAGGTCGACGCTTTTATGTGGGCGGGCATGTCATCATCACCTTCATAGGTATGCTTGTAGTAAGGGGCACGCTCCGGTACAAATTGGTTAAAGTGCTTTTCCATATCATGACGCACCGTTGGGTCGGCGTTTTCGTTAATGGTCAGGCTTGCAGAAGTATGTTGGATAAATAACTGTAATATTCCGACAGAAAAATCCGCTATTTCTGGAATTTGTTGTTCTATTTCATCGGTGATGAGATGAAATCCGCGTTTTCTCGCGCTCAATTGAATACTCTTCTGAGCCCACATATGGCATCCTCTATAATCTATTTGGCAAATTCAACTTAAGGCGGTAAGGTGAGTGCCAATAGCTAGTCATGGGTTTGAGTGTAACTAAATCACTTAACGTGACTTAACTCAATGATGGTGCAGCCACACTGGTTCATAATTGCACCTTGAAAATTTATTACAAAGTTCTGGCTGCTTGTTGCAATGCGAGAACCGAACTATTTTTGCTTAATCGGGGATTCTACTGGCTGTCGTTTATCTTATTATCCAGCCAACCAATGTAGAATAAAACCTACTGTAACATCGGGATAGTAACCATGTTGAAAAATATCAATCCAACGCAAACATCAGCTTGGAACGCGCTGACTGCGCACTTCGAATCGGCTCAAGATATGGATCTTAAAGAGCTGTTTGCTCAAGACGCTGCGCGTTTTGACAAATTCTCTACGCGTTTTGGTAGCGACATTCTTGTTGATTACTCTAAGAACCTAATCAACGAAGAAACTATGAAGCATCTATTCGCATTAGCTGGCGAAACTGAGCTTAAAGCAGCAATCGAAGTGATGTTCAGTGGTGAAGCAATCAACCAAACTGAAAGCCGCGCCGTACTCCACACAGCACTGCGTAACCGTAGTAACACTCCTGTTATGGTTGATGGCGAAGATGTCATGCCTGCTGTAAATGCCGTGCTAGAAAAAATGAAGTCTTTCACTGAACGTGTTATCGGCGGCGAGTGGAAAGGTTACACTGGTAAAGCGATTACTGATGTCGTGAATATTGGTATCGGCGGTTCTGATCTTGGTCCTTACATGGTGACTGAAGCTCTAGCTCCGTACACAAACCACCTAAATCTACATTTCGTTTCGAACGTTGATGGTACACACATCGTTGAGACGCTTAAGAAAGTAAACCCAGAAACGACACTATTCTTAGTCGCGTCAAAGACGTTTACTACACAAGAGACTATGACCAATGCACACAGTGCTCGTGATTGGTTCCTCGAGTCAGCAGGTGATGAAGCACACGTAGCGAAGCACTTCGCAGCGCTTTCTACCAACGCACCAGCAGTGTCTGAGTTTGGTATCGACACCGACAACATGTTCGAGTTCTGGGACTGGGTTGGCGGTCGTTACTCGCTATGGTCAGCTATCGGTCTTTCAATTGCCCTTGCGGTAGGTTACGACAACTTTGTTGAGTTGCTAGATGGCGCTCATGAAATGGATAACCACTTTGCATCTACTGAGCTAGAAAGCAATATCCCTGTGATCCTAGCGCTGGTGGGCCTGTGGTACAACAACTTCCATGGTGCTGAGTCTGAAGCGATTCTTCCATACGATCAGTACATGCATCGTTTTGCAGCTTACTTCCAACAGGGCAACATGGAGTCTAACGGTAAGTACGTTGACCGTGACGGCAATGCAGTGACTTACCAAACAGGCCCAATTATCTGGGGTGAGCCTGGTACTAACGGTCAGCACGCTTTCTACCAGCTGATTCACCAAGGCACTAAGCTGATCCCTTGTGACTTTATTGCACCTGCTATCAGTCATAACCCTGCAGGCGATCACCACCAGAAGCTTATGTCTAACTTCTTTGCCCAAACTGAAGCGCTTGCTTTTGGTAAGTCGGAAGAGACAGTTAAAGCGGAATTTGCCAAAGCAGGTAAGAGTGAAGAAGAAGCAGCGACGCTAGCACCATTCAAAGTGTTTGAAGGCAACCGCCCGACTAACTCGATCCTAGTCAAGCAAATCACACCACGTACGCTAGGTAATCTGATTGCAATGTACGAGCACAAGATCTTCGTCCAAGGTGTTATCTGGAATATCTTCAGCTTCGATCAGTGGGGTGTTGAGTTAGGTAAACAATTGGCTAACCAAATCCTACCTGAGTTAGCTGATGAGTCGGAAATCAGTTCTCACGACAGCTCAACGAATGGTCTGATTAACGCATTCAAAGCTTACAAAGCATAAGCTGATCCGAACTAAGTAAAAACGCCAACCTGTCGGGTTGGCGTTTTTGTTTTGGAGGGATGTCAGATCACATTCGATCTTATTGGTCTACTAATCTGAATTCTTATACTGTGGTATCGATACTAGATTTAGTATCCGTACTCGCGGTGAATTTCTGGCTGAGCAAAGCGACCATCTGATCGTAATACTGCATGTTTGGCTTGTTTCCAAGTAGCTTACACAATTCATTGCCAGTTGGGCTAAAGCGGTAGTAGAGAAGTCGAACCCCTTTGCTGTGCGCTTGAAGTGACAACTGCTTGCCCTGATAGTTAAGCGGTAAAGGGGAATCGAGCTCAATTTCACCTGACTCCAACTCAGTACCATGCATCAGGCCAAGTTCAAATAAGACCAACAGGCTGGAGTAGGGAAGCTGAAAATTACCAACGTTGATATTGCCAGTAATATTACGCTTACCGAAGCTGAAAATCCCCCCTTGTGCCCGATAACCAATCAACAGCTTGCGACTGGTATCGCCACCAAAACTGCACGCTAAAGAGGCGGCACGCTGTAAGGTTTGTGCTTCCTTAGGAGACATATCCTGTAGCACCTTTAGCGCTTTCATAGAGGTCGAGCCTGGGTTAGTGACTTCGCGTTTTAACACTTGTGCCCATAGGCGCTGCATTGAGGAGTTGTGTACTTCCTGAGCCATGTTGAAGAAGCGGTGCAGCCAATCCTGATCGGGGTCGCCCGCAGTTTCATCTTTACAGGACAGATGAGCGAGCTTAAGGATTTGTTCGAGGTTCTTCTGTTTCTGCTCTTTATAACGGCGCTCACGAATGAGGGCGCGTTCTAATGCGGTTTTTTCTGGTGAGTCTTTTTGTAATAAGGCATCCAAACCATGGGTTTGAGCAATGTTCATTACCCGGCTGGCACTATCTTTGATATAGGTTTTCTTTTTCTCATGCCGTGATTCAGCGCTGGACTCGTGCTCTATAACCACTGGCTTTTGCTTTTCCGACATGTGAATTCCTTGCCCATCAAAGTGTTAAACGTATTTATTAAATTTACCTCTTAACCTGATTGGCTGCCAGAGGAACTATGTGAGCAAGCTTTGAAAATTGGCGCTATGCCAAATGATAATAAGTTGTTAAAATTGTTATCGATGTTTTTGAGGGGCTTATGAAGTTTTTAAATGAGAATAAACTGAAAAAGCACCTGTCAATTGCCCTACTATTTCTGGTTTATCTAGCCGTCTTGGTATATCTCTCTAGATACATATAAAGAAAAGCGGATAATTAACAGGTTGCTTTTATGTTAGTCAATATTAGTAGTCATCGTACTCTTCGATAGCGGTGCCTTCTAAAACGTAACCTGTTTCAGCCAGATCATGGTTGATGGTTTCCGTTTTCAGCGTACCGACCACGTAAATAACATCCCACAGCTCTTGCACTGGCGCCCCTTTAGGGAACTTCACGTAGATGATCTGGTTCGGCGGTGGTGGTGGCACGTGAATACATGCACCAAAGTATGGCACCAATAAGAATTCGGTGACTTTGTCCTCATCCCCTTCTAACGGAATGACAAAGCCAGGTATTTTAACCTTACTGCCGTTGAGTTCCTGGCGAACCTTACCCACCAAGGATTGCTTGGCTGCCTGACCAGAGTGATCAGTCACTGGCATACCGTACGAATCGAACTGCTTTCGTTCACTCTCCGGGATCAGATCGATCCAGTCCAGTTTGAGAATATCTTCTTTAGCACTAACCAGAGAGGGCGTCATGAAGCTCATGGCTAGCATGACGACTAAGATTATCTTTTTCATGTTTAAACCCGAATTGTCATCCCATCACTAAGGGATTGTCTGTATGCACGGAACGCTGGAATAAAGCCGATTACAGTACCTGCTAACTGCACAGCTGCAAGCAATATCCACTCATAATGGGATATGGCTACCATCTCAATATTAATTCCATACTGTTGTTGAATTACTGGTGCAACGGTGGCCAATAGCGCATATAAACCTACTACACCCACAGCTAAGCCAACTAAGGTTAAGGCACTGGCTTCGAG

>NODE_5_length_5178_cov_2.513625

TGTCGATATAGCGCCGCTTCTGATACGCCGACGCGTTTCGCTAGCTTGGCGGTGGTGATGCGAGAACCACCATCGTTAGATTCGAGCATTTGTGCGAGTGCTTGTAAGATTTCTTCCCGGCGGTTGCTTTTTCTTGTTCCGGCCATGAACGTGTTCCTTTTGTTACCACTGTTGATGCCCATAGACACAGGCATTCGCCCAACTTAGCAGGCTAAGATGGGCGGAATAGACGAATTTCTGATTGTTAGAATAGCGACTTAATTGTCTAACTTTGCGGCGATAATTGTAAGTATCTGCTTCGCAATTTGTTGTTTTGATGCAAGGGTAAGTGATTGCTCACCATCTTTCCAGTATAGGTTAATCGCATTGTCGTTGCTGTTGAAGCCTTGTCCTTCTACTGAGACATCGTTGGCACAAATCATATCGAGATTTTTCTTGGTCAGCTTAGTTCGAGCGTAATGTTCTACATCCTGAGTTTCTGCGGCAAAACCAACGGTAAATGGGCGCTGTTGAGTCATACCGGCCACTGAAGCGACAATATCAGGGTTTTTGACCATAGTGATGGTCATTTGTTCGCTGTCATCCGTCTTCTTAATTTTCTGATCTGAAACCGCTTGTGGGCGATAATCCGCCACGGCTGCGCAGCTGATGAAAACATCATGCTCACAAGCCTTGTCCATTACGGCGTTATACATATCTTCTGCGCTCGCCACATCAATGCGCTGCGAACCAGCTGGTGTTTTAAGATTCACCGGGCCTGCGACTAACGTGACCGTTGCACCGAGCTGAGCCGCTGCTTCCGCTAATGAGAAGCCCATTTTCCCTGAGCTGTGGTTAGTGATGTAACGTACTGGGTCGATGGCTTCACGGGTTGGCCCAGCAGAAATTAATACTGATTTACCTTTGAGAGGTTTGTCGCCCCAGAACTGTTCGCACAATCCCACCAGTTGCATTGGTTCTAACATTCGGCCAGGGCCGACATCGCCACAGGCTTGTTCACCGGCTGCTGGTCCCCAGATGTGCATGCCACGTCGCTCTAGCGTCGCAATGTTCTCTTGTGTTGCTTGATTGAGATACATCTGTTGGTTCATGGCCGGAGAGACTGCGATTGGAGCATCGGTCGCAAGTACCAGTGTGGTGAGTAAATCATTGCCCATGCCCGCTGCCATGCGTGCAATTAGATCAGCGGTGGCTGGTGCAAGTAAGACCAAATCCGCCCATTTAGCCAGCTCAATATGGCCCATAGAGGCTTCTGCCGCCGGATCCAATAAGCTATCGGAGACTGGACGTCCGGAGACCGCCTGCATTGTCAGTGGTGTAATGAATTCTTTTGCGGCTTTAGTCATAACAATCTGAACTTCAGCGCCACGTTCAATCAGACGACGCGTCAGGTCGGCACATTTATAAGCGGCAATACCACCACCGATACCAAGTAGGATCTTTTTGCCCGCGAGTGTTTGCATGATGATCATCCTTAAAAAATACTGCGAGTAGATTACCACGCAGCCAATTGAGACTCTAGAAATGGAAAGTGTTGTTGCAGATTCCGTGCGCTTTTGCCTTTCTCGTTATTGCTTCTTTATATACCTATCATTTACAGAAAATCTTTCACTGCGACGAAGATAATTCTTTTGCTCGCCAGTGAGTGAATAAGAAAGTTTAAGCTAATACCACCCTATTTATTGTCTTAAGGATAAGTGTTTACATGTTGCCTAGAGAGTCGATGCCAAGAGAAAAACTGCTCAGCCGAGGTCCACAATCGCTCACCGATGCGGAGCTGTTAGCGATTTTTCTTCGCACTGGTACGCAGGGCATGAATGTACTCCAGTTATCAGACAAACTGCTTAAGGATCTCGGGTCGCTACGCAGCTTATTTGCGGCGAGCAAAGAGGAGTTCTGTCAGCACAAAGGGCTGGGAGAAGCCAAATATGTCCAGCTGCAAGCCGTGCTTGAGATGACACAGCGCTACCTAAGCGAAACCTTACAACGTGGCGAAGCGTTAACCAGCCCTCAGCAGACCAAGCTTTATCTTTCTAGCATGTTGCGCGATCGCCAGCGGGAAGCTTTCTATATATTGTTTCTTGATAATCAGCATCGGGTAATCAAGGATGAAGTGATGTTTGAAGGAACCATCGACGCCGCATCTGTATATCCTCGAGAGGTTATGAAGCGGGCATTGGAGCATAATGCAGCGGCTTTGATTCTTGCTCATAATCATCCATCTGGGGTGGCGGAACCGAGTCAGGCTGATCGCAGAATCACCCGACGTATCAGTGATGCGCTGGCTTTGGTCGACATTCGAATTCTCGACCATTTTGTGGTTGGAGATGGCGATGTAATTTCCTTCGCTGAGCGTGGATGGATTTAAATCACATTATTTGTTGTGAGTTGCGCGAATTCTGCTACAATCCCCCGACATTTTTTGAACTATTATCAGCTCTGCCTAAAAAAGATCACGAAAACCTTGAAAAAGGATCTGTTCGGGTCTTGAGCAATGCTAGTCAAGTTAGTATAATGCGCGACCTTTGATAGCCTTGTATGGGTTTCCATAGCGGTATAAGACCTCAAACTTCTATATTTAGAAACTGAGAGGTTCGGCCACCAAGGTTGATATCGAGCTGAAACGATTTTGGAGAAGACATTCATGTCACGAGTATGCCAAGTAACTGGTAAGCGTCCAGTAACGGGTAACAACCGTTCACACGCACGCAATGCTACTAAGCGTCGTTTTCTGCCGAACCTACAAACTCATCGTTTCTGGGTAGAGAGCGAAAAACGTTTTGTTAAACTACGTCTATCTGCTAAAGGTATGCGTATCATCGATAAGAAGGGCATCGATGCTGTTCTTGTTGACATTCGCGCAAACGGCGAAAACGTTTAAGAGGAAATAGGCAATGGCAAAGAAAGGCGTACGTGAGAAAATCCGTCTAGTATCTTCTGCAGGTACTGGTCACTTCTACACTACTGACAAGAACAAACGTAACATGCCAGGCAAATTTGAGATCAAAAAGTTTGATCCAGTTGTTCGCCAGCACGTTATGTACAAAGAAGCTAAAATCAAGTAATTGATTTCTTCTTTGAAGCTTCTTAAACAGAAGTCTGAATTAGAAACCCAGCGTTCTCGCTGGGTTTTTTATTACCTGAAATTCCCAAAAGGCGTCACAGTCTAGCCTACCTCCGCTCATTTCCCTCTTTCCTGTTTTCTGCTACCTTTTCTTATAGTTACCTACAGGGAAATCAAAGTTCATGCGTGTTTCTACTTCACGGCGGCGTCGCTGGAACAACATCATGATATTGGGCATCATCGCTTTCATCGCGATCCTCAATTTGCCGACTATCATCAAAACCTATTTGATTGATGACGAACCGGTCGCTGAAGTTAGTCCTTATCCTTATTTGCTCAATCCGTCGGCGGAGTTGAAAGCACTGCATTTTTCTGACTGGTCACTGGAGCACTTGAACGGTGAGTGGAGTTTGACTAAGCAAAGTAGCCTTTCACCACAGGAGCTAGTTGAACGCTGGTCGGGCTTAGTTGGGACCGAAGTTGATCAGGACACTTTTGATGGATTGAAGCCTCAATTGACCTCACCACAGACGATTGAAGTTTGGTATCGCGATCAAGAAGAGCCTCAAAGGATCACCTTCTATCAAACCCCACAATTCTGGTTGTTGAAAAACTGGCAGGATCAATGGATAGCCGTTTCAGTTGATCCAGAATATCTGCTACCTTAAACGCCCTGAGTTATTAATTGCCATACCACTATGCCTGAATTACCTGAAGTTGAAGTCAGCCGTATGGGGATCACTCCTCATTTAGCTGGACAAACCGTTGCCAAGCTGACTTTTCGCACCCCCAAGCTGCGCTGGGATATCCCACATGAGCTAAAGAAGATGGAAGGTCAGGTGATCCGCAGTATTTCTCGCCGTGCTAAATACTTGCTGATTGAAACCGATGTAGGTAGCGCGATTGTGCATTTGGGTATGTCGGGGTCTTTACGTGTACTGGATGCAGACATGGAGCCGGGAAAACACGATCATGTCGACTTAAAGCTGACCAATGGCAAGGTACTACGTTATAACGATCCCAGACGTTTTGGGGCTTGGTTGTGGACCGAAGATGGTGAACATGATGCCTTGGGCCATATGGGGCCAGAGCCGCTAACGGAAGACTTTGATGCGCAATACATCGCGGATAAGGCAAAGAATAAGCGTGTCGCGGTGAAGCAGTTCATTATGGATAACAAAGTCGTAGTTGGGGTAGGAAATATCTACGCTAATGAGTCATTGTTTAGTGCACGTATCCATCCGACACGATCAGCAGGCACGCTAACGGCGAAAGAGTGGCAACGTTTGGTTAAAGAGATTAAACAGGTGCTCGATACGGCCATTAAGCAGGGCGGAACAACATTAAAAGATTTTGCTCAGGCGGATGGTAAACCGGGCTATTTTGCTCAAGAGCTGCAAATTTATGGCAAAGCTGGTGAAGCCTGTCCTAGCTGTGGCGAAGCGATTGAAGAGCAGAAAATTGGTCAGCGCAATACGTTTTTTTGTCGCCATTGTCAGAAATAGTTAAATATAGAGCTGAGGTTTCATGTCAGCTCTATATCTCTATTAAAATTAGACTTTCGCTTTGTTTTTCAATGCCTCAGCTACCGCTTTAGGCACAAAGTTGGTGACATCCCCTCCATGAATAGCCACTTCACGGACTATGGTCGAGGAGATAAATGCGTGCTCTTCCGCGGGTGTGAGGAAAACGCTTTCTAATCCGGGCATCAGTCTACGATACATATTGGTTAAACCAAATTCGTACTCAAAGTCTACGGTGGTACGTAAACCACGGATAAGAACGTTCGCATTTTCTTGCTTCGCAAAGTCGACCATCAAACCCGAAAATCCCTTCGAGCTTACATTGTCCAAATGCTGAGTCACCTGCTGTGCAAAAGTTACGCGCTCTTCTAGCGTAAACATGGTGTTCTTACTTGGGCTGGCAGCAACAGCAATGATCACTTCATCAAACATATCTGCCGCACGTTCGATTAAATCTAAATGGCCATTGGTAATCGGATCAAAGGTTCCTGGATAGATCACTCGGGAAAGGCGTTTTTTGCTCACAGTCAGTTACTCAAATGATTCTTTTATATCGCTTAATGGTAACAAAAAGGCTCACATTTACCCAAC

>NODE_6_length_5020_cov_2.233664

GCATTGTACTGGCGTACCGCCCTGGTTCGCCGCTGCGTGCTCGTTTCGAACAGCTGGCTGAAGCAATAAAGCATCAGCTTGAAGCTAAGTAAATCTTAGCTAATAAAAAAGGGCGGGTGTCATGACATCCGCCCTTTTTGTTTTGATGCTGGTTAAAACAACTCTTCACTTTCAGCAGAGGTATAGATGCTGTCAGTAATTTCATCTGTGACGTATTCAGTTGGCTCCGTCCCTTCAAGGAAGTACTCAAACATCGAGCTAGAGTCGAACTTGTTGGTCAGTAGCCCTGAATCACGGTCAATTCTTACGCGAACAATATTTGGTGGTATGACTTTCTGCTGAGCAGGTACACCCGCTAGTGCGGTGTGCATAAAGTCAACCCAAGCTGGCTGAGCGGTTTTCGCGCCAGCTTCGGCACCGGAAATTTGCCCTTTGCCTAAATTGCTGTTTGGCTTGGTGCGGCCTAGCTTACGGTTGTGGTCATCAAAACCGACCCAGGATACTGCAACCATGCCTGGTCCATAGCCGTTGTACCACGCATCTTTAGAGTCATTGGTGGTACCTGTTTTGCCGCCCACATCACGACGCTTCAGAGCTTGTGCTCGCCAGCCGGTACCATTCCAACCAGTACCATCTCGCCAGTTACCACCACCCCAAATGTTGCTGTACATCATTTCACGCACGAGGAAGGCGGTCTGCTCAGAGATCACCTGCGGCGCGTATTGAGGTGTTTGATAATCTTGAGGCTCAACGTCTTGTTCTTGGAACTCGTTAGTGACGGTTTCTGTCGCCGCTTGTTCACAGTCTTGACGACAGATGGATTTCGGTGTCGCTTCAAACTCTACATCACCAAATGGGCCTTCTACTTTGCTGATGTAATGTGGTTCGACGTAGTAGCCACCATTAGCAAACACTGAGTAGCCTTGTGCCATCTTCATTGGTGTCAGACTGCCAGCACCAAGAGCGATTGTCTCAGAGCGTGGTACTTGATCAATATCGAAGCCAAATCGAGTCAGATACTGGCGCGTTTCGTCTAAGCCCACTTCACGCAGCACGCGCACTGCCATAACGTTTTTAGATTGTGCTAAGCCGATTCTTAAGCGAGTTGGTCCGATGTAGGTCGGAGGCGAGTTTTTCGGGCGCCATGCTGTCCCTTGGCTCTTATCCCACTGGTTGATTGGCGCATCATTGACCAATGTCGCCAGAGTCAGGCCCTTATCGATTGCCGCTGAGTAAATAAACGGTTTGATGCTGGAGCCAACCTGACGCACGGATTGTGTAGCGCGGTTGAACTTATTGTGAACGAAGTTGAAACCGCCAACCAGAGACAAGACTGCGCCATTTTCTGGATCCATGGCTACAAAAGCTGTATTGGCATTTGGCACCTGGCTTAAGTGCCAAGCAGTTTGTGTTTCGCCATCTTCAACAGATGTCTCTACCTGACGGACCCATATTTGTTCACCTTCTTCCAGAACATCCGTAGCGGCTTTTGGTGCTGGGCCTTGACGGTCATCGGTGAGGAATTTACGCGCCCACTTGATACCATCCCACTCGAGCGTTTTGACACCTTGGTTTTTCACCCAGACAGTCGCTTGTTGGGCTGTGACTTGTGTGACGACCGCAGGGAGAAGGGAGCCGTAAGTTGGCACTCGGTCCAAAGTGTCGTCAATTTTTTCTTCATCGAATGCTGTCTGACCTGCTTTCCACAGGACTTTTTCAGCGCCGCGGTAACCGTGACGTTCATCGTACGCTAGCAGGTTATTGATGGCAGCTTCATAGGCTGCAGACTGCAGCTTAGAATCCACGGTGGTGTAGACATTCATTCCTGAGCTGTAAGCTTCTTCACCGTAACGCGCGACCATCCATGCACGAGCCAGCTCTGCCACATAAGGTGCACTCAGTTCAATCTCTGCGCCATGGTAGCGCGACATGATCTCTTCAGCACGGGCCTGTTGATACTCATCTTCAGTGATGTACTTTTCATCCAGCATACGCTTAAGGACAACGTTGCGACGGCTGGTGGCACGCTGAAGCGAGTAGATAGGGTTCATGGTTGACGGGGCTTTTGGCATGCCTGCCAAGGTGGCAATTTCACTCAACGTTAGGTTCTGTAGATCTTTGCCAAAATAAGTCTGCGCTGCTGCGCCAAAGCCATACGAACGGTAACCTAAGAAAATTTTATTCACGTACAGCTCAAGGATCTCTTCTTTACTCAGCAGTTGTTCAATGTGAATAGCGATAAAGATCTCTTTGATTTTTCGCATGATCTTTTTGTCATTGGACAAGAAGAAGTTACGTGCCAGCTGCTGAGTGATCGTACTCGCACCTTGCTTGGCCGAGCCTGAAAGAGCAACGACAACAGCAGCACGTGCGATACCAATAGGGTCGATACCGGGGTGATCGTAGAAACGGCTGTCTTCTGTCGCGATCAGCGCTTCAATCAGGTGTGGGGGAATTTCATCATAGGTGACCGGAATACGTCGCTTCTCACCAAACTGTGAGATCAGCTTGCCGTCTTGAGTGAATATCTGCATTGGTGTCTGCAGTTTGACATCTTTTAAAGTCGCCACATCGGGCAACTCTGGTTTTACGTAGAAGTAGAAACCAACAATGGTCCCGACCCCAAGTACCGTGCAAACCAATACGAAAATAAACAATCGCTTTATGAACTTCACCGGAGAATCCCTGATTAATTAAGCTTACGTTATGCAAACCTAGGTAGTTTAAGCTAAAACGTAGCTCAAATCTGTTTATGTGCTTACTAAATCAACGATTTAAGCCTAAGCGCTAAGCAAGTTTATGGAGCTGAGCTATATGGGTAAATCACTAATTACAGGCATAGATATCAGCCACCAGAGTATCAAAGCGGTGGTCTTAAAACCTGAAAAAGACACTTTTGCACTTGTTGGTTATCACCAAATGCCAGTCGAAGCGGCCTTTTTTAATTCGAATCACAAAAGGCGCACTCTCAATATTTCACATAAAAAGCACAGCATAATTCGCCCTTGGTAATCTAAAAACCTTCACAGAGTGAGGATCCAAACGTATGATCAGGCGTAAAAATAATAAGCATGTAGTCGTATCATTTACGCTGCTCTTTTCCTCTGTAGTAAGCCATTAAGGAAGCATCATGAAAAAATGGATCACGTTAGCGCTGGCCAGTCTTTTAGCCCTACCCACGTGGGCGGGCCAGTTTAAAACCATCAAAGATGTCGAAGTTCACTACTCCGCATTTAATTCCACATTCCTAACTCCGCAAGTCGCGCGTAGCTATGAGCTGAAACGAAACGGCTACTCAGCAATCATTAATATCAGTGTGTTAGACAATTATCAGGCTGGTAAACCTGCCATAACCGCGAAAGTTAGTGGTAGCGCGAAAAACTTAATCGGCCAAACTAAGACACTTGAGTTTCGCGAAATAAAAGAAGGCTCTGCGATTTATTACCTCGCTGAATTCCCTATTTCGGACGAGGAAAACCTAACATTCAATATCGATGTTAATGCTGGTAATAAAGGCACGGGCAGATTAAAGTTCACCCAGAAATTTTATGTGGAAGAGTAACGACTAAACACCCTTCCCAATATAGGCACAAACAAGATTATGAATGCACAAAAAATTGTCCTAGCGACAGGTAACCAGGGCAAGGTTCGTGAGATGGCCGATCTGCTGGCAGATTTCGGTTTCGATGTCGTGGCACAAAGCGAATTTAATGTCTCAGAAGTTGCTGAAACTGGCACAACATTTATTGAAAACGCCATCATCAAGGCGCGTCATGCAGCGAAAGAAACTGGATTGGCGGCCATTGCCGACGATTCAGGCCTTGAAGTTGACTTCCTAAAAGGAGCGCCGGGCATCTACTCTGCACGTTATGCCGGAGAAGGCGCGAGTGACCAGCAAAATCTAGAGAAGCTGCTCGATGCAATGCAGGGCGTTCCGCAGGAGCAACGTACTGCACGTTTCCATTGCGTATTAGTCCTGATGCGTCACGAATTCGATCCAACGCCGATCGTCTGCCACGGTAAGTGGGAAGGCCGTATTCTGACTGAAGCTCAAGGTGACAATGGTTTTGGTTACGATCCGGTTTTCTTCGTTCCAGAAGATAACTGCGCTTCAGCAGAGCTTGAACCAACACGTAAAAAGCAACTTTCTCACCGTGGTAAAGCGCTTAAGCAGTTGTTTGCGACACTTTCTGAGCAGCAATAATGAGCCAATTGATTCCACCAGCACTGAGTCTTTATGTCCACATCCCTTGGTGTGTACAAAAGTGCCCGTATTGCGACTTTAACTCGCACGCATTAAAAGCCGACATCCCAGAGCAGGATTACATCGCTGCTCTACTGGAGGACCTTGATACTGATATCGAGCGTTATCAGCTTAAAACCAACCCACGGGCACTCCACTCCATCTTCATCGGTGGAGGGACGCCAAGTCTCATCTCAGCGCAAGGAATCGCTGATCTACTTGCTGGCATAGAAAAAAGAATCGCGTTTCAAGACGAGATTGAAATCACCATGGAAGCCAACCCGGGCACGATTGAAGCGGAGCGCTTCGCTGGGTATCGCAAAGCTGGAGTGACGAGAATTTCGATTGGTGTGCAGAGCTTTGAACAGCAGAAGCTCGAACGTCTAGGGCGTATCCATGGTCAGGATGAAGCCGTCAATGCCGCGAAACTGGCTCACAAGATTGGCTTAAACAGCTTTAACCTTGATTTAATGCACGGTCTGCCAGACCAAACCGTAGAGCAAGCCCTCGTGGATCTGGAGAAGGCGATTGAGCTTAACCCGCCACATCTTTCGTGGTATCAGCTCACTATTGAGCCCAATACTATGTTCCACTACAAGCCACCTGTTCTGCCCGACGATGATGACTTGTGGGACATTTTTGAGCTCGGCCACCAAAAGCTGGCTGAAGCGGGCTACGTTCAGTACGAAATCTCAGGCTACAGTAAGCCAGAGTATCAATGCCAACATAACCTCAACTATTGGCGTTTTGGCGACTATCTCGGCATTGGCTGTGGCT

>NODE_7_length_4884_cov_2.844602

TTTTAAAGGAAAGGACACAATCATGCCGTACTTACTGGCGCTGATGCTCTCCATTTTTACCCTCACGGGCTGTCAATCTGCTTATTACTCAGCGATGGAACAAGTAGGCTATCACAAACGCGACATCATGGTAGATCGTGTCGAAGATGCCAAACAGTCTCAGCAAGATGCGCAACAAGAGTTCACAAGTGCACTGGAAGCGCTCTCAGCACTCACTCAGTTTGATGGTGGCGAACTGGAAACCGTCTACAACCAGATTAACGACAAATACCAAGACAGTGAAAAAGCAGCACAAGAAGTCAGTGACCGAATTGCAGCAATTGAAGATGTGTCAGACGCACTCTTCGCCGAATGGCAAAGCGAGTTAGGTCTCTATACCAGCACTAAGCTCAAACGGGCAAGTGAACAGAAACTCAAAGAGACTCAGCGCTCATACAACACTATGTTAAGCGCGATGAAGCGTGCAGAAGATAAGATGACACCTGTACTCAATACATTGCGCGACAATACGCTTTACTTGAAGCACAACCTCAATGCGAGTGCGATTGGTTCACTGCAAGGCGAGTTTGCAAACTTAGAACAAGATATCCAGTACGCAATCAAGGATATGAATGCCGCGATAGCAGAATCAGATAAGTTCCTGCAAAAGCTCAATCAAAAGTAGTGCTTTAAGGCCGCCTCCAAGTGAGGCGGCTTTCGTTTGTGGCCTCAGCGACTATAGCTGTTACCTGATGAACAAAGGCCACGCATTCTTTACGACTGCGTAACAACAAAAAGCCCTGAGTTGATATCAACTCAGGGCTTTAAAGCTTAAATAACGATTAAGTAAAATTACTTAGCGTTACGTGCTTCACGCTTACGATCGCTTTCCGTTAGGAACTTTTTACGGATACGGATACTTTCAGGCGTTACTTCTACTAGCTCGTCGTCATCGATGAACTCAAGTGCTTGCTCAAGCGTCATGATGATTGGCGGAGTAAGAACCTGTGCGTCATCAGTACCTGATGCACGAACGTTGGTTAGCTGCTTACCTTTCAGTACGTTTACTGTTAGGTCGTTGTCACGGCTGTGGATACCAACAACCATGCCTTCGTATACTTCAACACCGTGACCGATGAACATACGGCCACGCTCTTGAAGGTTGAATAGTGCGTTGGTTAGCGCTTTACCCATACCGTTAGAAATCAGTACGCCGTTGATACGTTGACCGATCTCGCCACCTTTGTGCGGACCGTAGTGATCGAATGTGTGGTAAAGAAGACCAGAACCCGACGTTAGCGTCATGAATTCAGTTTGGAAACCAATCAGACCACGAGATGGCATGATGAAGTCCATACGTACACGGCCTTTACCATCTGGAGACATATCAGTCAGCTCACCTTTACGTAGGCCGATGTTCTCCATGATGCCGCCTTGGTGCTCTTCCATCACGTCGATAGTTACCGTTTCGTACGGTTCCATTAGCTGACCATCTTCTTCTTTGATGATAACTTCAGGACGAGATACCGCTAGCTCGAAGCCTTCACGACGCATGTTTTCGATCAGGATAGATAGGTGAAGTTCACCACGGCCTGATACGCGGAATTTGTCTGGATCATCTAGTTGCTCAACGCGCAGTGCAACGTTGTGTACTAGTTCTTTTTCTAGGCGCTCAAGGATGTTACGTGACGTTACGAATTTACCTTCTTTACCAGCAAACGGAGACGTGTTTACTTGGAACGTCATGGTTACCGTTGGCTCATCAACAGAAAGTGCTGGCAACGCTTCAACTTGGTTTTGTGCACAGATAGTATCGGAGATTTTTAGCTCACCAAGACCAGTGATTGCAACGATGTCACCTGCGTTAGCTTGGTCAACTTCGTGACGTTCAAGGCCTAGGTAGCCCATTACAGTGCCGACTTTACCATTGCGAGTCTTACCATCCGCACCGATAACAGTAACCTGTTGGTTAGGCTTAACGCTACCACGAGTTACGCGAGCAACACCGATAACACCAACATAAGAGCTGTAATCTAGCTGAGAAACTTGCATCTGAAGTGGACCGTCTAGGTCAACTTGCGGTGCTGTCACCTCTTCAACGATGGTTTCGAATAGCGGTTCCATATCTTCGCCAGTTTCGCCTTCTTCTAGAGAAGCCCAACCGTTAAGCGCTGATGCGTAAACCACTTTGAAGTCTAGCTGTTCATCAGTTGCACCTAGGTTATCGAATAGGTCGAATACTTGATCCATAACCCAATCAGGACGAGCACCAGGACGGTCAATCTTGTTGATAACAACAATTGGCTTAAGACCGTGAGCAAATGCTTTCTGTGTGACGAAGCGTGTTTGAGGCATAGGGCCATCAACAGCGTCAACAATCAGCAGCACTGAGTCTACCATTGACATAATACGCTCAACTTCACCACCGAAGTCCGCGTGTCCTGGGGTATCTACGATGTTGATGCGGTAGTCATTCCAGTTAATTGCTGTGTTTTTAGCAAGGATGGTAATGCCACGCTCTTTTTCGATGTCGTTCGAGTCCATGACTCGCTCTTCAGCTTCACCGCGAGACTCTAAAGTGCCTGATTGCTGTAGCAGTTTATCAACCAGTGTTGTTTTACCGTGGTCAACGTGCGCGATGATCGCGATATTTCTCAATTTATCAATCTGTGGAGTAGCCATGGATTTGATTCACTCGATAGTAGAAGCTCGCTTCAAACATGGTCGATGAATGAAGCGAGCTTACTTGATTAAAAAAACGGCCATAATGTACCAGATTTTAGCGAAAAACCCAGAAATATGTGATCTGATACGTTGGTTTTCTGAAGAATATGCTTTGATCGAACACAATATCGCTCTGAATATGCAAAATTGTGCATTGACATAGGTAGCGAATGGCGGCTGAATGGAATACGCTTTTGTTTCATATTGGTGCACTTCAAGATATTTGCACCAATAGAGTGCAATTAAAGATCATTTTGGTGCAAACTGGTGCACCAAACTGATACCCAAACCACTAATTCATTGAAATTAATGGAATAATTTTTTTGGCATGGTTTTGGCTTTATTGATATCAGCATCGATTAATACGATTGAAATGAAATTAATCAATGCTGGATTTGTTCATCAATCGAGCTAACACCGCTTTAATAACACTGGAGGTTATCCAAGATGTCAGTTGAAAACGTTCTATCGCTGATCCAAGAAAACGAAGTTAAGTTTGTTGACCTACGCTTTACCGATACAAAAGGTAAAGAGCAGCACATCTCAATTCCTGCTCACCAGATCGATGCAGACTTCTTCGAAGAAGGTAAGATGTTCGATGGTTCTTCAGTTGCTGGCTGGAAAGGCATCAACGAGTCAGACATGGTTATGATGCCAGACGCATCATCCGCTGTACTTGACCCATTCACAGAAGATGCAACACTGAACATCCGTTGTGACATCCTTGAGCCTGCAACTATGCAAGGCTACGATCGCGACCCTCGCTCTATCGCCAAGCGTGCTGAAGATTACATGCGCTCAACAGGTGTTGCAGATACCGTACTTATCGGCCCTGAGCCAGAATTCTTCCTATTCGACGACGTGAAGTTCGCGACTGATATGTCAGGCTCTTTCTTTAAGATCGACGACGTAGAAGCTGCATGGAACACAGGTTCTGATTACGAAGAAGGCAACAAAGGCCACCGTCCAGGCGTGAAAGGTGGTTACTTCCCAGTGGCTCCTGTCGATTCATCTCAAGACATCCGTTCTGCTATGTGTCTAATCATGGAAGAAATGGGTCTAGTTGTAGAAGCTCACCACCACGAAGTAGCGACTGCAGGTCAGAACGAAATCGCGACTCGTTTCAACACACTGACTTCGAAAGCAGACGAAATCCAAATCTACAAATACGTGGTTCATAATGTTGCTCACGCATTTGGTAAGACAGCGACCTTCATGCCTAAACCACTAGTTGGTGACAACGGCTCTGGTATGCACGTTCACCAATCTCTAGCTAAAGATGGCGTTAACCTGTTCGCGGGTGACAAGTACGGCGGCCTGTCTGAAACAGCCCTTTACTATATCGGTGGTATCATCAAACACGCTCGCGCAATCAACGCATTCGCTAACGCAGCGACTAACTCATACAAGCGTCTTGTACCAGGCTTCGAAGCGCCTGTAATGCTGGCTTACTCTGCTCGTAACCGTAGTGCTTCTATTCGTATCCCTGTGGTACCAAGCCCTAAAGCACGTCGTATCGAAGTTCGCTTCGGTGATCCATCAGCTAACCCATACCTATGTTTCGCAGCAATGCTAATGGCTGGTCTTGACGGCATTAAGAACAAGATTCACCCAGGTGAAGCAATGGATAAAGATTTATACGACCTACCAGCGGAAGAAGCAGCTGAAATCCCAACTGTTGCTTACTCACTGAAAGATGCACTAGATTCACTAGATGCAGACCGTGAGTTCCTAACAGCTGGCGGCGTGTTCTCTGACGACTTCATCGATTCTTACATCGGCCTTAAGTCTCAGGACGTTGAACAAGTAAACATGACGACTCACCCACTTGAGTTCGAACTTTACTACTCAGTGTAATCGTTCAATCGATTAACCCTTCCTCCGAGGCTCACCTTTTTGGTGAGCCTTTTTTATGTCTTATATATCAATGAAATTCACTTCAAATCTTTTCTATTCAATATCTTATGCACGGCAAGCTTATTGCATTGTTACAGTTCATAACGGTGCAATTAACGACAACAATACTGAAAAGCCTGTGATATATGGACTTTTCACTTCTTCTGCATGAGTTATTGCGCCATACTTGGAATGTAACCGCACCAAATTGGTGCATTGCACATTTTAAGGTCAAGGATGAGGGACGACAGTGAGCAGCGAACTCGATAACATAATACTCAATCA

>NODE_8_length_4809_cov_2.291209

GAACTATTCGACGGTGGTTTCCACGCAGTTGACTCTTCAGCTATTGCTTACGAAATCGCTGCTAAAGGTGCATTCCGTCAGTCTATGCCAAAAGCTGGTGCACAGCTTCTAGAGCCAATCATGAACGTTGACGTATTCACTCCAGAAGACAACGTTGGTGACGTAATCGGTGACCTTAACCGTCGTCGTGGTATGATCAAAGACCAACAAGCTGGTACTACAGGCGTTCGTATTAAAGCTGACGTTCCTCTATCAGAAATGTTTGGCTACATCGGTCACCTACGTACTATCACTTCTGGTCGTGGTCAGTTCTCTATGGAGTTCGCACACTACGCAGCTTGTCCGGCAAACGTTGCTGAGCAAGTAATCGCAGAAGTTAAAGAGCGTAACGAGAAGAAGTAATTCTTTACGTTAGCTAGATAGCTTAAAAGCCCCTGAAGTGAAAGCTTCAGGGGCTTTCTTTATTGGGGCTCCTTTTTCTGTTTTCTCACCTAGGGCTGCGAGAATCAATGATCTCCAAGTCACTAGGGTGCTCTTTCTGACATTCTTCGACTTGGCTGAAAATCCAGTCACAGAACTGACGGATTTTCTCACGCTTAAAATAGGCGCTAGGGGCACAAAGGAAATAGTTGTAGTCCGTTCTCTGAGCGAGGTTCCCTATGCGGACTAATTTGCCTTCTTCAATGTATCTCTGAGCCAGGGTATGCTTGGCTAAAGCCACTCCTTGTACGGCCAGTGCTCCTTCCAGAACAAAATGCGAACCGTTGTATTTTAACGTCGGTTTCGAGGTCTTATGACCAACGTTTTGTAGCCATATTCCCCAATCGAGATCTCGCCAATAATCTTCGATCAGATCCGCGCCCGTTAAGTCACTAAGATCATAAATACCATGCCTTTCTTGGTAGATTGGGTGGCATACAGGATAGAGCAGCTCTTCAAGCATGAGCTTGGATGTTAGATTCGGGTAGTTTCCCGGCCCATAACGAATGCATAGATCAAGGTTAGATCGTTGAAAGTCCACTAGTTCGCTGGTGGGTTCAATCATGAGTTCTTGTTCTGGGTGTAGAGCTCTGAAATGACCGATTCTCGGTACAAGCCAGTGTTGAGCAAAAGAAGGCAGTGTCGAAATAGACAATAAGTTTGGGTTAGGATCCGAATTGATTTGACGAACTCCATCCTGAATGTGCTCAAAGCCTTTTCGAGCTTGCTGATAAAGAATTTCGCCTTCCGCAGTAAGCTGAACTTTTCGGTGCTGGCGCACAAAGAGTTCTACGCCCAGAAACTCTTCTAGCTGACGTATTTGTTGGCTGATTGCAGCGGCAGTGACAAACAAATGTTCAGCGGCTTTCTTAAAGCTACCTAATTGTGCTGCTGTGTAAAAGTAATACAGTCCTTGTAAAGGAGGGAGGCGATCTTTCACTTAAGTTTTCCTTAACTAAAGGTCAAATTCCATCGTTTGAGAGTGATGCGATATTGTCCGATTATTAAGTCGAATCTTAATAAAGGCAAGGTGAAGTTATGGAAACTACTGCTAAATCTCCAACGTGCTCAATTGATGAACGTCTTTCCTTAAGACAGAAATTCTTAACTAAAATCAAAAGATGGCGACGAAATTACCGTACTCGACGCCAGCTCAATCACTTGTCTGGGCACATGCTACGTGACATTGGTGTTGAGCATGAAGAGGCGGCACGTGAATCTCGCCGGCCATTTTGGGATGACTAGCTCTGTTGATCACTGATTTCACCCTGGAGCCAGTTAACAAACGCTTGGATACGTTTTTTACGCGGAGAATCTTCACTATAGAAAAGGTTGAACTGGATACCCGGTGTCATGCCTATATCAAAAGGTTTGACTAACAGGCCTCGTTCAACGTAATCACTGGCGAGAGAGTCAGAAGCAAGACAGGCTCCATGGCCAGACATCACGGCTTTCATCGCATGGTCAAAGGTACTAACTTCCATCCAACGAACGGAGTTTTTGCTCATGCTGACACCTGCTTGAGCGAACCATGACTCCCATGGATAGCCTCCTGATTCATAGTGAATTAGCCAGCATGCTTGTAGTTGAGATGGCTTTTTTAAATTAATAGACTTAGCAAGGTGAGGAGAGCAAAACGGATAAATCGTTTCTTCGAATAAAAACTCTTTGTGGAAGGATTTGTTGCTTTCTATGTCGAGTTGTTCGCCTTGCCAGATAGCGAGATCCGCATCACCTTGCTTTAAATTTGGTGCATCACAGCTAGTAAGGATGCGAATCGGAATGTTCGGGTGCTGAGTGGAAAACTTCCACAGCCTAGGTAGTAACCATCGGGAAGCAAACGAAGGCGTTGAATTAACGCACAGTAGGCCTTCTATGGGTTCACTTTGGATCTGATTTAACCCTGTGATGATATGTTCGAAGCCATGAGATACGTGCTTGAAGAGTATTTTGCCCTGTTCAGTCAAGCGCATTTCTCGTCCGTCACGGACAAAGAGTTTGCAGCCTAGTCCATCTTCCAATTGACGTATTTTTTGGCTAACCGCAGCTTGACTGATGAACAGCTCTTCTGCTGCGCGGCTATAGCTATTAAGTCTAGCGGCCACTTCGAAATAACGTAGACCTGGTAGATGATGCAACCTTGCATCCATAATAATTCCTTGCGTTGAGCTCCCTTATGGCTAAGCATCATAATCTCAATTGATAAAAAAGCAAAAAGGAGAGCTCTCGCTCTCCTTTTACCTATAGATTTTTAGTCTTCCCAGACGACGAGTTGACCCTTCGGCCAGTTATGACCCACGTCGTGGTATTTTTGCTCAAGGACATGACGTTTGATTTTCAAAGTTGGAGTGAGGATGCCATTTTCAATACTCCAAGGGTCTTTGATCATCAGAACCCCTTTAATCTGTTCATGAGACTCTAACTCTGCATTCATTCTTTCCACTACCTTCTTTGTGGTTCTTTCATATCGCGCACGATCGAAGTTAGGGAAATCGTGAGGGACTACCAATAGGATAGGAGCCGGGAGACCTAGTCCGATCAAACACATCATCTCCACACGGCTGTATTCAAATAGTTTTTTCTCAATTGGTACAGGTGCAACGAATTTACCTTTGGCTGTTTTGAAGGTGTCCTTTTTACGGCCCTGAATGGTTAAGTAGCCACCTGCGTCGATAAAGCCGATATCCCCAGTGTGTAACCAGCCCTCGGAATCAAATGACTCTTGAGTAGCAATATCGTTTTTGTAGTATCCAGAAAACAGACCTTTGCCACGAACCATGATCTCTTCATCTTCTGCGATCTTAAGCTCGATCCCTGGGCCTGCATTACCGACTGAGCCAATTTTATCTGCTCTGAATGGGTAGTTGAGGGTACTGTATGCGAATGACTCGGTCATTCCCCAAGCTTCGGTGATGTTGAGGCCAACGCTATGGTACCAGTTAAGCAAGGCTGGAGAGACGGGAGCAGAACCACAGCCCAAAACACGAGCCTGATCCAGTCCAAGACCGTCAGCGAGTTTCTTCTTAATCAAGGAGTTAACAAAAGGAATCTTGAGCAGGATATTGAGTTTCTTCTGCGGTAGTTTGTCTTGAATGCGTTGCTGGAATAAGGTCCAAAGGCGCGGCACCGAGATAAATAGTGTAGGGCGGTGCATTTTGACGTCTTCGATGAAAGTATCAAGGGATTCAGGGAATGCAGTCGGGACTCCACCCATGATGGAAGAACCAAAGATGTACACGCGCTCAGTGATATGCGCCAGCGGCAAATAAGAGAACAAACGGTCATTTTCCTGAATGCCGATATGATCGATCAGTTGCTGTACTGACCAGCTAAATGCGCCATAGGTGAGCATCGCGCCTTTTGGTAAGCCGGATGTACCTGATGTGTACACCAGCGACATTAACTTATCATCGTAGTGTTCAGGACGAGCATTGCTTGGCTGAGATTTAGCAATTAAGTCGTTAAAGCTGTGCTGACATTTCGGTGCTGTATCGTATGGAAGCGAAATGCTGATTAGATTTGGCATTGCATCCAGTACTTGCTGAGTTGCAGCAGCATCGTCAAGCTTACCGCCAATTAGCGCTTTACTTTCACTATGAGTGACGCAGTACTCAATGGTGTCGGCCCCCGCTGTTGGGAAGATGGGGACGCTGACAAAATCGCCCAGCATCATTGCCAAGTCGCAAATGAACCATTCAGCACAGTTTTTCGAGACCAGAGCCACTTTATCGCGAGGCTGTATACCTAACTCTTCTAAGGCAGACACCAGCTTTAGTGCTTTGTCAGCAACTTCTGCATAGGTGAACTCGACGAATTGACGGTTAATAATTTGTTTTAGATACACTTCATTTGGGCGTTCCTCTGCCCAGCGAAGGATCATTTCATTTGGTGGTGGGAGAGCACAAGTCGTTTTGCTTAACTCGTTAGGCTGAATCATTTTCTAACTCCATGTTATTGGCAAAGTTATGGTATTTTTTATATTGTTAACAGCTTGTTAACTTTAGCACGCGGGGTGAAAAAGAGGGAAGTATTAGAGGGTAGTTCAACAGGAACTTGTGACCGTGATGTGAAAACTAATGTAGCTGGCGGTAGATGGCCGGACTGAAGCCCGTTTTTTTCTTAAAGATGCGTGAGAAGTAAGACACATCGTTGTAGCCACATTGAAAAGCTATAAATGAAATCTTCTCTTTTCTCTTGTCACTAAGTAATTGTTTAGCAAGACTAATTCTTTTAAGGGTGAGGTAGTCTCTGAAACTGACTCCAATCGATTTATGGAAGAATTTTGAGAAGTATGTAACAGAGTAATGGCAATACTCTGCAATATCTTCC

>NODE_9_length_4630_cov_2.421480

CTTTGTCATCTTCCTGTTTGATCTGACACTCAAGATAATGCGCAGCTACTTTATCGACGTAGCAGGCAAAAAATCCGATATTCTGATTTCCTCCAAGCTGTTCAGCAAAGTACTTGGCATTCGAATGGAAGCGAGACCACCATCGGTCGGCGCATTCGCTCGTCACTTACAAGAGTTTGAGTCCATTCGGGAGTTCTTCACTTCAGCCACCATTGGTTCTTTGATTGACTTGCCTTTTGCGCTGCTATTCCTTGTTTTAATCTGGCTGATGGCGGGTAACCTTGTTCTGGTTCCAATTGCAGGGGTGATCATCCTGATCATCTACTCGGCGCTGATTCAGGGGCCTTTGCGACGCACAATTGAAGAGGGTTCACGCCTAGCCTCACAAAAGTACGCTAACCTGATTGAGAGTCTGTCAGGGCTAGAAACCGTCAAGCTGTTTGGTGCTCAAAGCCAATTCCAGTTCCGCTGGGAAGAAGCGGTGGCTCATATGGCAAACTGGAACATCAAGAGTCGTCGCATCACAGATGGCATCCAGAACACCGCAGGCTTCGTTCAGCAAGCATCCAATGTTGGGATGATCATTCTTGGCGTGTACTTGATTGCAGAAGGTGACCTCACCATGGGTGGCCTGATTGCAGCAACCATGTTGAGTGGTCGAGCCATTGGTCCTATGGTTCAACTTTCTTTGCTATCTACGCGATACAACCAAGCCAAATCTTCAATGACTATCATCGAGCAAGTCATGGAAATGCCGGATGAGCAGGAGGAAGGGAAGCGCTATATTCACCGTCCAATTGTTCACGGTAAGATTGAATTGGATAAGGTCACCTTCCACTATCCGGACTCGCCGATAGCTTCTATTCGAGACCTGAGCATTACCATCAACCCTGGTGAGAAAGTCGCTATCATTGGTCGAATTGGCTCAGGTAAAACTACGCTTGAGCGTTTGATCCTTGGATTGTTTAAACCGACGGAAGGCCATGTTCGTATCGATGATACCGATATCGAGCAGCTGCATCACATCGACATCCGACGCAACATTGGCTGCGTGCCACAAGACAGCCAGCTCTTCTATGGCTCCATTCGCGATAACATTACTTTGGGTCGCCCATTGGCCGACGATCGTGATGTGATGGATGCGGCTAACCGCGCTGGTGTGACCGTATTTACTCAGCAGGACCCAGCAGGTCTAGAACGTCAGGTCGGTGAAGGAGGCTTATTGCTCTCCGGTGGTCAACGTCAAGCCGTTACCATTGCACGCGCCTTCCTTGGTCGCCCACCAGTGCTGTTAATGGATGAGCCAACCAGTGCTATGGATAACCGCTCTGAAATGCACATCAAGCAACAGTTAGCGCAACTAAAGCCATCGGAAACGCTGATCTTGATTACGCATAAAACATCCATGCTAGATGTGGTTGATCGAGTCATCGTGATGGAGAAAGGCTGCGTCATCGCTGATGGGCCTAAAGCTGAAGTACTCAACAGTCTTAAGCAAGGTAAAGTCAGAGCCGTAAACTAAGCTGACAAGAACAACAAAGCCGCTCAGTATTGAGCGGCTTTTCTTTATCTACGATAAGAAGAGTTGTTTAGATTAGCAGTTTGCAGGGCCTACTTCTTTCCAAACACCCCACTGACCAGATTGAGTAGGATCATCACCTTGTGTCCACCACTTCGCTTCCCAGACTTTACCATCCCAAGTCACTTGGTCACCGCCATTGTAGATTGCGCCAGAATCCCAAGCGTTGGTACAAGTACCGCCACCAGTATCCTTCTTCGCGACAACAACAGTAGCCGAAGCAGAAGACGTCGCTTGAGAGTCACTGACTGTTACAGTAAATAGCAATGAAGTATCTTGCGTGTACTCAGCAGCAGTGAATGTCACCGTTGCACCACTAATGGTGGCATTGAGTCCAGCAGGCACATCCCAGCTGTACGTTAATATGTCATTATCTGCATCACTAGAACCAGAAGCGTCAACTACCACTACGTCTCCCGCATTCGCTGTCGCAGGCGCTGATACTGCCGCAACTGGCGCAGTGTTTACAGGACCAGTGTCTTTCGCCTGTACCGTCACTACCACAGTGTCGGTACCCGTAGCCCCTTCATTGTCCGTAACTGTCAGTTTGAAAGTAAGCGTTTCAGTTTGCGCAACTTCTGCCACATCGAAACTCGCCTGTGCGGTGTTGGCGCCCGTTAGTGAAACGCTTGTACCTGAGAGTTGTTCCCACGCGTAGCTTGCAACACTGCCATCCGAATCGCTTGAGCTGCTACCATCCAGTACAACCGATGCTGGACCTGTTACTGAGACATCAGCTCCCGCTGCTGCAACTGGGGCTCTATTTGCCGGTGTTCCACCAGCTAGCCCTTCATGCATTGCGTTTAGAATGTCGCCATTATCAGCATCAATTTCCCAAGAGAATAGACCCGCAAGGCCCAAGTTACGGACATATGCACCTTTCGCTTTGACAGAACGATCATCATCAAACGTAATCAGCTGACCTGTAGAACGGTTCCAAACCCAAGGTGCTTCAGCTTGCGCGTCGTACCCGTATTCAAAACCGTTGATGCCGGTATTGTCCGCACCAAGCATAAAGGATTTCACACCTTTATAATCAATCACACCATCTTCCCAGACACCTTGTGCTGTGCTCCCTTTCAGCTTACCAGTACCTGTTCCTGTCATTGGGTCATTAGGATCGGTGAGCGTATCAGGTGTCACGCCTTCCCAACCACGGCCATACATGGCGGTACCCACAACGAGTTTATTCGCAGGTACACCTTGTGCTAGAAGAAGCTGAATACCATTGTCCGTCGTGTAAGCAGGGCCTTTATACGGTTCGCCATTTTCATCTACGCCAGAGCCATCACACTGACCTGGGCGCATAAATGTACCGCAGTAAAGTGCCGTCTGATGACCAGGAACATTGTTCCAACCGCCGTAGAAGTCATACGTCATAGCAAAGATGTAATCCATGTACTGAACTGCATCCCCGTAGTTCACATCTTCAATTTTGTCGTAACCCACACCGATTGCTGACGTAAGCTCATACGTTCTGCCAGTGTCCGCTTCCAGCTGGTCAAGCATAGCGCGAAGCTCTTGCATTAGCGCAATATAAGCTGGGCCATCATTCACAGGGTCTCCTAAATCAGGAGCAGCACCGCCGCCACCTGGGAATTCCCAGTCAATGTCCACACCATCATAGAACTTCCACGTCGTCAGGAAGCGCTTAACAGAAGCGACAAACGTGTCTCGGTTCGCTTTGGTAGTAAAGTCAAAGAATGGGTCTGATAACGTCCAGCCACCAATGGAAGGGATGATTTTCAGGTCTGGATTGCGTTGCTTAAGCGCCATAAGCATCGCGTAGTTGCCTTTGATAGGAGAGCTGTACTCATGGCCCGCTTGAGGGAAGCTCTTCTGATAAGCCGCCCATGGGTCATGAATCACGACTTCATAGTCTGGAACCCCTTTACATGCGGTCATCAGGGCGTTGTAGCTGTTACCACCGACTGATTTCACTGATTCGTTCGGGCCACAAATTGGAATAAAGCCGTATAGAATATGGGTCAGGTTATCGGCAGGAATATTGTCTACCGTAAAGTCACGGCCATAGATACCCCACTCAACAAAGTAAGTACCAACGACCGTGTTAGGGTCCGTGTTGTAACTCTTGTTGTTTGGATCAACATTCATCGCCAGCGGTGCAAGGTGAGCACCATCGGTGTCAGCAACCACAATTTCAGCAGGCGCACTCGTGCTACAACCGCTTGCGTCACACGCTTCAATCGTCATTTGATAACGACCGCCATTTGCATATTTGAAGCTTGCTGTGGTCTGGCTACCGGTAATTGCTCCGGTTGCCACCTGAGTTCCATCAAAGTAAACCTTGTAGGTGTCCCCTGTTGTACCACTCCACTGATTAAACTTAATGGTGATATCCGCTTCATCGAAATATTTCACCATGTCGTTGTAGCCAGAAGTGGTTTCCATTGCTAATTCAATCTTCGAGAACTGAAGATTGTTTGAGCCATAAACATCAATACTAGGGGCTGTTGGTGCAGCCATCGCGGTGCCGGATAGCGCCAAAGCGATACTAGCGGCGCACAAAGTTATACGATTCATACCTATTTCTCTCATTCCTTGTACATTTACCCGAAAGTTGGGAAGTGATACCTATCATCGTAAGCCGCGTTTAACTGCCTGCTGGCCAAGTATTGAGCCGAAAAACTTATCGAGGATCGGTATTCGCTGTTCTACGGATACATCGCGAAGAACATCACTTTCAGTATTTGAGAGACTTTTTTTTTGATAAAAAAAATAAAGGTTGTTTTCGAGGCAAGATCAGAAAATAGTTGTATATACGAGGTCTGTTACATTACAAATTGGATTTCGACCCATAATTTATAAAATCCCAATAAGCCGAAAAACATACTTATTTGTCAATAATTTAAAGAATGAGGTACCAAATATTGGAGGATCACCATTACAAAATACTGCCCCCTAAAAATTTTAGGAGGCAGGTTTCAATCAAGGATTATTTGGACTTAAATCGCGCCGTGAAATGACGCAGTACGGGAGGTTCGTACTCAAACGTTAAGCCTTTAAGTTCATGAGCACGCTCTTTGAGCG

>NODE_10_length_4574_cov_2.359128

TGCTAAAGCACGTAAAGCGCAGCAAGAAGGCAAACAGGAACCCGTCCCCGTTGAGCCTGAAAACGCTGAAGTAGCCGAAGAGGATCCTAAGAAAGCCGCGGTCGCTGCCGCTATTGCGCGTGCTAAAGCACGTAAAGCGCAGCAGGAAGGTAAATCGGAACCAATCACCGCTGAGCCTGAAAGCGCTGAAGTGACAGAAGAGGATCCGAAGAAAGCTGCAGTCGCTGCCGCAATTGCGCGCGCTAAAGCACGTAAAGCGCAGCAAGAAGGCAAACAGGAACCCGTCACCGCTGAGCCTGAAAGCACCGAAGTAGCCGAAGAGGATCCTAAGAAAGCTGCTGTAGCTGCCGCAATCGCGCGCGCTAAAGCACGTAAAGCGCAGCAAGAAGGCAAATCGGAACCCGTCACCACTGAATCTGAAAGCGCTGAAGTTGCCGAAGAGTATCGAAGATGAAACCATCAAGGAATCCGCAATAAACAATATTGAAAGCGTAGATAGCGACATATTGATAACTGCTTTTCAGCTTGTGCCATTAGATAATGTACTCAAGGAACCTGTGCCCTTTATCGAGGAAATTATTAGGGTAATTTCACAACAATTACTCTCACGTGATAGAGAAGACCGTCTTGATTATGCTTTCAGACATGAGTTTTTGAAGCACTATGCTCGTTTTGTCCTTCATCTAGATAATGGCAAAAAAGATAGATTCCTGAAATATTTTACCGATGATTTCACCTTGAGCGAAGGTACGGCAGATTTATTAAATGAATTTGTTAGCGCTGAAGACTCGCTGAATATGCCGGACTCATTTTGGTATGTATGGAACCGATTTAAAGGCTACATCGTTGATTCAATTAGTAGTCAAGGTTGGCGGCATGATAAAGAGCGCACAATCGAATCCTTTTTATTTGCAAGAATCCCTTGGAAGGACGATGCAATGGCTTGGCATACATTTTCGGCTGAGAATAAAGTATTTTTTAATGACCTGAGCGGTAAAATAGGTGGTGAATCTTCTTTTATCTATAGTATCAGCAAACTCCTTTGTGGCATTGGAAGTGAATTTTTAGATGACGGTATTTACTGGATCAGTAATGCAATAAAAACCTTTGACATCGACTTGTCGCAAGACAAATCAGGAAATACACTATTTTACTTGGAAAGATATATGAGAAGATATCTATTCAAAAATCATGATAGAGTGAGACAAACACCTAAATTAAAGGTACAAGCATTGGTAGTGTTGGATTTTCTAGTCGAGCAATACTCCATTACAGGTTACCTCCTTAGAGAGGACATAGCCTAGCTAATAATCATTGTAGCCGAGGTATGAATTCTGGTGGGCTAAGCTAAGTTCACCATAAGACTGTAGCCAAGGTTCCTGTGTCGTATAACAAATGCTTAAGGTTTCAATATACTTTTTCTAATCTGACAGAAATGGATTAGAGCTATGCATCTAAATCATTTCTGTCCAGAAACTTGCTAAAACAATTGGCACATCTTTGTGAGCTTAATTTCTTTAGTAGAGCATCTCTTCACAAACAAAAAAAGCCGCTTAATGCGGCTTTTTTTATCCCGATAACGATGCGTTTAGGCAATATGCGTCATGCGGTTGATTGTGAATGTCTCTACCGCGCCGTCAACCGCTGCGACGTAGTCAGCGATATGCGTGCTGGCGAGATGCTTATCCAGCGCGGCTTCTGACGTCCAGTTTTCATGGAAAACGAAATGCGCTGGGTTGTCGTTGTCTTGATGCAAATCGTAGTTAATGCAGCCATCTTCAGCGCGTGTTTTGTCGATCAACTTAATTAGTTCAGTTTTAACCAACTCAACCTGGCCTTCTTTAGCAATGATATTCGCGATAATCGTTAACTTGCTCATGTGCTTACCTTTTCGTCAATTTCTGATGCTTTACAGTGTCGTGATGACATCTTCTAGAGGCAGACGCGCTTTTGGCAGACCGTGGTTATAATCCTCACCGTCTTTGTGATAACCCAGTGCTAGAGCAAAATCACACACAAAGCCTTCCAGTTCTTGTTTGAACTCTTCACCGATTAGCTCTGGGTCTACCCCTTCCATTGGTGTTGAAGCGATGCCCATGCGCGCCAACGTGTGTAGCGTGTTACCCAGTGCGATATAGGTTTGAGCTTTTGTCCAATTACCGTTGAAACCTGTCTCATCTGTATTGAGTTCCGCAAAGCCGAACGCACCGTTTAACATGTCGTTGTATCGCTCTGCTGGCAGGTGGCCTGAGGTTACTTCTGCATCGACCACTTTTTTGTAGTTGTCTTTGGTAAAGCGAGGGTTGTGAGCAAACAAAATCGTATGAGACGCTTCTTTGGCATGCGGTTGGTTGAACTGGTGCATGTTGGCAAACGTGTTGTGGAAACGTTGTTTTGCTTCGTCACTTTCAAGAACGATGAACTTCCAAGGTTGTGAGTTGATAGAAGAAGCAGATAAACGAATCGCTTCTTTAATGACTTCCATATCTTCAGCAGAGATACGCTTACTTGCGTCGTATTTTTTTGCTGTGTAACGAGTGTTTAGATCTTTGATGATTGGGTGAGTCATGTTGGCTCCTAGTTGAATTTAGATAATTGCATTAGGTCTCACGAACCGCTCGGACGATGTTGAACCCTTATTTCAAGTAAACGAACAAGCTCAAAAACTCGAAGACTTCGATATGAACAATGTCGATGGGCGTAGATTAACGACATTAAAGAAGGGGAAAAATAGCGAAAATTTCCAATCATTATGGAAATAATTGTCCATAATTAAACGGAGTGCTCGACAAAAGGTGGAGCAAGATGTGCGGACGCTTGAATGTAACGGATGATCCACTGGCGAGAGTGGTTTCCCAACTGGTGGGGATCGACTTCTTCCCTCAGATGAACCTTGACTTGAAACCGACTGAAAATGTCTCTGTGGTGGCGTTTGAAGCACAGGCGTATTGTCAGAAAGATATCAAGTGGGGAACTCAGCCTGAATGGGCTAAGCGATTGATTATTAACGCTCAGTCAGAAACGGTGGTCAGTAAGCCTACATTTCGTAACGGATTTGTCCGCAGGCGTGTCATTGTGCCTTGTAACGGTTGGTATGAGTGGTGTGAGAGTGGAGGAAAAAAGAACAAGTTCTTGTTTACCAATTCAGATGGCATGCCTCTGTATATGGCCGGAATTCTGACGGACGATGGCAATTTGGTCACTCTGACGACTAGCCCGAACACACAATGCTTACCATACCATCATCGTATGCCATTTCTTGTCCCTGATATTGGTGTCGAGCAGTGGTTAACGGCAACGCCACAACAGGCACAAAGTTACCTTGAGTTAGGTTGGAGCGATGAGCTTTTAATTACTGCGGTTTAGTGCGTCAATAACGCGCGACCAATGGCATCAACGAGTTCAAGAATGTGGATTTGTGATTCTCGGCTGTATGGCGAGATACCATCGACTTGTTCTGCAAGATCTAAGTTGGTGCTGGCAATCCCCGCGAGCGTTGACCCTGTATGGCTGATCGCGAATACCTTCAAACCTTTGCGTTTCGCATTGTGGGCCATATCCACCACAGACTGCGTTTCTCCCGATTTGCTGATCAACATAACCGTACCACTTTCGGCGCGCGCCAGTTGGTGTATGTCTGTGATCACTAAGTGGGGAACATTCGCCAGTGATAGAAATCGCGAAAGGTAAGAGACGCTGACAATCGAGGCGCCACGGGAATATAGGTACACCACTGGCGACTGATCTAATGACTGGGCGATCTTTTCGATGCGTTGAATCTGGTCATCTTCTTCAATCACAGAGTTTGTCTGTTTTTTCAGATCATCGGCGAGCTTGTACCTGAGTTCCGTGTAACTGGCGTAGCCCATCTTCTTGCACACACGATTGACTGATGTCGTCGTCGTGAGTGCTTTGTTTGCAATGGTCTTCGCAGAAATCTCAGCCAGCTCACTCGCGTGACTGATCAAGTACTCATAGATAACGCGCTCAATGCCCTTAAATGTATTCATCGCATACAATCATTAGTAATGAAATTGTCATTATTAGGCGCTAACTAGCAAATATTTTCCTTGATTGCTATCACAAAAGTATCGTCATACGTAGGTTGTATCAGGAAACTGTACCTTGGTACATGGAGGCTAAATATACTTGGCGGAGTTGATAAGAATACGGTGAACAACCATGAGTAAGAAAAAGCTCGTTGCAGTGACCGCTTGCCCTACCGGCATTGCACACACATTTATGGCAGCGAAAAAAATTCAGGCATGGGCTGAGAAGCAAGGCTACGAAGTCAAAGTTGAAACCCAGGGCAGTGATGGCGTTAAGAACAAGCTGACAGCGCATGATATTGCGACAGCAGACGGCGTGGTTCTGGCAGTCGATGTGCCGATCATGGACATGGAACGTTTTGATAATGCCAACCCGCTAAAAGTTCGCACTCAGGAATTGATCAAACGCGTAGATGAACTTCTGCCAACTGTCTTCCTGCGTGGCAAAGAGAAATCTGATGCCGATATTGAAGTGGCAGAAGAAAAACGCTCCGCGTATCAGGTGGCAATTGGCCACATCATGAC

>NODE_11_length_4484_cov_2.505786

CAAACACACCGGGCGGCATCTGCTTGATTTGAAAGTCAATCATGTGATTGAAGGTATCAAGTAATGGGGTGAAGTCTTGCTCTGGTTGCAGCAAAGTCAGGATATGGTAGCCAGCCTCCACGGTAGACAAGCTGTTGTCACTGGGCGCTTTGCGAATACGGTAATTACCCTGTAGGTTCTCTGGCAAACGCACTAAAGGTAATGCCTGTAAATTCTCAGACAGTTGCCACATTTTGTACGCCTTTTTCCACGTGCCATCGAGCAAAATAATCCGCACTTTGCCTTCGCTGGAGACGGCTTTAGCTACCTCTTCATGGCTGACAGACACTTCTCCCGGATAAAGCACAAAATGTTGATAGCCTTCTTCATCCAACAGCTGATTCAGCTCACTATGTTGAGTAAAATTTTCGCCTTCAAACAGGTAACTATTCTCAAGCGAGAGTCTGAGAATACGCGCTGTGCCCATGGGCCTGTTTGTTTCTGTCGGATGCTGTAAGATGACCAACTCAACATTCGATGTTAGGCTTTGTATCCACTCACAAATACAAGCTTTGAGTGACTTACCACATTGAGAACAATATCGGGACATAGCGTGCGTTTACTTCTTCTGTTAATTGCTATCAGCTTAGTCTGCCTAGGCGTGCAGTTCGAGCCTTTAGCCAGCCTGACATACTGGCATCATCAACTTATTTCTGAAGGCCAGTGGTGGCGAATCCTAACAGGAAATTTCACCCACACCAACTTTGCTCATTTAGGCATGAATCTGGCTGGCTTGTGGGTTATCACTTATCTGTTTAAGCCAAGAGTAACTTCTCTGCTCAGTGTATTGTTCGTGATTAGCTTATCCGTGGGTGTACTCAACCTGACAACAAACATGACTAGCTATGTTGGTCTTTCTGGTGTGCTACATGGCATCTTTGCCTATTATGCGCTTCAGGAATCCTTACAAGGTCGTAAAGGTAGCTGGTTACTTGTCATTGGTGTATTAGCCAAAGTGGGCTGGGAAATGACCATGGGCGCCTCTCAATCGACCAGCGAGTTAATCAATGCCCGTGTAGCGGTGGAATCTCATCTGTTTGGCGCATTGACCGGTCTCGTGCTTGCTTTTGTCACTACTAAGCTAAACTTTCAGACAAGACTTAACCGCTCTCAGCGATAATTTCTATTGATGTTCCTGACCAAAGGGCTCAGAATGGCGCCCTTTTGCTGGTGGTCGATTGCCGCCAGAGCACTTATCTCACCTTAAACAGAGAACAATTTATGGGTTTTACCGCTTTAGGCCTTTCTGCCCCGCTCCTCAAAGCCATCGACGATCAGGGTTATGACAAACCATCTCCAATTCAGGAGCAAGCCATTCCTGCAGTGTTAGCAGGTAAAGATGTCATGGCAGCGGCTCAGACAGGTACGGGTAAAACCGCAGGCTTCACATTACCGATTCTAGAACGATTAGATAATGGCACCCGAGTCAAAGGCAACCATGTAAGAGCACTGGTGCTGACACCGACCCGTGAATTGGCGGCTCAGGTTCAGGAAAACGTGTTCAAGTACAGCCGTTATCAACGTCTGACCTCAAATGTCGTATTCGGCGGAGTGAAGATTAATCCACAAATGATGAAGCTGCGTAAAGGCACAGACGTGCTTGTCGCAACCCCAGGTCGATTACTCGATTTATACCAGCAGAATGCAATTAACTTCTCCCAGTTAGAAGTTTTAGTGTTGGATGAAGCAGATCGCATGTTGGATATGGGCTTTATTCGCGATATCCGTAAAATTCTCGCACTGCTTCCGGAAAAGCGCCAAAACCTACTGTTCTCTGCAACCTTCTCAGCTGAGATTCGCGAACTAGCCAAAGGTCTAGTCAATGATCCGGTCGAAGTCTCTGTTAACCCTGAGAACTCCACTGCGGTCACCATTGAACAATCTATCTACCCAGCAGATAAGCGTAAAAAAGCACCTATGCTGGTGAAGCTCATCAAAGATGGTGATTGGAGACAAGTTCTGGTCTTCAGCCGTACCAAACACGGAGCGAACCGACTTTCTCATTACCTTAATGATCAAGGTATTACGGCAGCGCCTATTCATGGCAACAAAAGCCAAGGTGCGCGCACCAAGGCACTGGCTGATTTTAAATCCGGAGAAGTGCGCGTATTAGTTGCTACAGATATTGCCGCACGTGGTATCGACATTCCTCAACTCCCTCAGGTCGTTAACTTCGAGCTGCCTCACGTGGCAGAAGATTATGTCCACCGAATTGGCCGTACTGGCCGTGCCGGTGAAGTCGGCAAAGCGATCTCTTTAGTTGAAGCAGCGGAAGCCAGTGAACTATTTGGTATTGAACGACTCATCCAGCAAGTATTACCTCGTTTGGAGCTGGAAGGCTATAAGCCCGTCAATGTGCTGCCAGAGTCTAAGTTAGATACTCGCCCAATCAAACCTAAGAAACCGAAAAAGCCCAAGAAGCCAAAAGTCGACCATGCCGATGGTCAGCGCTCAGGTGACAATGCTCGTGGTCATAAGCCAGCGGGGAAAAACAAACGTCATTTTGGCCATAACAAGCCCAACGGTGATGGCTCCAAGAGTAATAATGGTGGCAAACGTAAGGACGGCAGTTCTGGCAATAGCAAAGCCTCAAACAATACTGGCAATAATGGACAGAAACGTCGTAGACCAGCGAACCGCAAGCCAAACCATTCGCAACCTAAAGCACAGTAACCAAATACACAAAGCCCCTCAGTTGAGGGGCTTTTCATTTAATCAACCAAAAAAATCGGCTTACACGATAGGACGCTGACCACGATCAATCAGCTTCATCAGTACGTTATCAGCCGCTTCACCTGCTAATCCAGCAAGCTTTGAGGTCAGCTTTTTCTTCTCTACGTAGTGAATCGCTAAAACTGTTTTATCTTTGCACGCTTCCACAACCAGATCATCAGAGGTTTTGATTTCATCAACCAGACCCAGTGAATGCGCCTGAGTGCCAAACCAGTGTTCACCAGTGGCCACTTTATCTAATTCAAGTTCAGGGCGACGCTCACGAATAAAGTCTTTAAATAGACCATGTGTTTCTTCTAGCTCCTGTTTGAACTTATCACGCGCTTTGTCGGTGTTCTCGCCAAACATGGTTAAGGTACGCTTGTATTCGCCTGCTGTTAGCTGTTCATACTCGATATCGTACTTTTTGAGCACTTTATTGAAGTTAGGTAGCTGGGCAATCACGCCGATGGAGCCTACGATTGCAAATGGTGCTGACACAATCTTGTCAGCAATACATGCCATCATATAACCGCCACTGGCAGCCACTTTATCTACTGCAATCGTCAGTGGCAGCTTAGCCGCCTTAATACGATCCAGTTGAGACGATGCCAAACCGTAGCCATGCACCATACCGCCACCAGACTCTAGGCGCAGTAGTACTTCGTCCCCTTCACGAGCTACCGCGAGAATTGCGGTCACTTCTTCGCGTAGTGACGCGACTTCTTTGGCGTCAATGCTGCCTTTAAAATCCAGAACAAATAGGTGAGGCTCACGCTTGCTGTCCAAATCACCGTCTTTTGAAGCCTGCTTCACTTCTTTTTCACGGGCTTTTGTTTTCTCTTTCTCTTGTTTCTTTTCTGCCTTATCACGCGCTTTGATAAACGCTGAATCATGCAGGTGATGCTCAAGCTGCTCAACGGTGTTTTTATGCTGATCCGTCAAGTTGGTGATCTCCAATTCACCCTTTGCAGCACCACTCTTACCCCCTACGGCTTTGGCTATAACAAGAATCGCAATCACTGCGACTACAACAGTCACAATCTTGGCTAAAAACAGCCCGTAGTCCAACAAAAATTCCAATGTGATATCCCCTAATGTGCGAATTAGTGTATTGTAACCACCATCTTACGGATTCATAAAGCAGAACATTACAATAAGGATGCATGTCGTGGATTATTCCGTTTCCTCAGACGCTTTAAAAGACAAAGTCATTCTCGTGACAGGTGCCGGTGACGGCATCGGTAAACAAGCCGCAATCTCATTTGCTCAGCATGGTGCCACCGTCATTCTGTTAGGTCGTACAGTGAAGAAGCTTGAGCAAACTTACGATGAAATTGAAGCTGCTGGCTACCCTCAACCAGCGATCATTCCTCTGGATATGAAAGGCGCAAGCAAGCAAAACTATATTGATATGGCCGATACCATTGGTAGTCAGTTTGGTCGACTTGATGGAGTGCTACACAATGCCAGTCAACTGGGTGTACTGAGCCCATTTGATCAGATTGGTGAAGACACTTACGACGATATCATGCAGGTTAATGTTAAAGCTCAGTTCCTGATGACTCAGGCAATCCTTCCGCTACTGCATAAATCAGATGATGCTAGTGTTATCTTTACTTCATCAACGGTTGGCCATGCTGGCCGTGCTTTCTGGGGAACTTACGCAATATCTAAGTTCGCAACGGAAGGCATGATGCAGATTCTGGCAGATGAATTGAGCGATACACACA

>NODE_12_length_4414_cov_2.762278

CTAGTACTGCATGTAGAACCGGAAGCTTGACCCGATCCACGAGCGGAGCCCACAGAAAGTTAATAGCGTAAACCGCGAACACACTGCCGAAGTAGCCAATCGCTGCACGTGTTAAGCCAGCGTCTTTTAACCAACCCGACATGTTAGAGCCGATCAAAACCCATGGAAAGCCACTTGAGCAACCGAGCATAAACACCCATAGCAGGCGTTTATCATAATAACTACGAATCGTTTCTAGCCAAGACATTGAGGGGGTGTTGGCAGACATAAAGTCTTCCTTGTTCGTTTTATAGTGAGTGTTAAGTTAGGGAAAATAGGGTGAATTGAAAAGGCTCCAACTGGATAAGTAGGAGCCTTTTTAGATTATGGGAGTAAAGTCACTTTCGTTACGACAATAGGTGTTGCTGGAACATCACGGTAGCGGCCGACTGTTTTGGTGCGCTTTTTCGCCATTTGCTGTACTACGTCGAAACCCTTGGTTACTTTTCCGAACACAGCGTAACCCGGTGGGCGCTGGTCGTAATCAAGAAAATTGTTATCAACATAGTTGATAAAGAATTGACGCGTTGCAGAATTAGGGTTGTTAGTCCTCGCCATAGCAACGGTGGCTGTTTCATTCTTTAGGCCATTAGACGCTTCATTTTTAATAGGCGCGTAAGTTGGTTTTTGCTTCATGTCTTGATCAAAACCACCACCTTGTGCCATAAAGCCCGGAATAACTCGGTGAAACTGGGTGCCGACATAGCTGCCGTCTTTGACATACTTAAGGAAGTTGTCCACTGTAACAGGGGCTTTTTCCTGATTGAGCTCAATCGTAAAAGCACCTAATGTGGTTTCAAATTCAACTTTAGGACCTGCAAACACCAGATTGCTGACTAACGCCAGTGAGGCAATAGCAACTTTCCACATTAGAAGCGCTCCTGCATGTAATTCTTCAACTCACGGTCGCTGGCGACTTCTTTCAGAACCAGGTTAATTACATCGTTAAGTACGATTTCGATTTCTTCGTTTGAAGCACTTAAAGCGCCTGTACGTTTAGCCGTGCCCGTGTAAGTCTTAACTAGCTTACCCTGAGGCGTCTCTGCTGTGACTTCAAGAACGACTTTTCCATCCATCTCGTTTTCCATCACTGAGTGTTTGACGGAAACCAATGCTTCTTGCACTTCAATCTTTACTGAGTTTTCACTGTTAACAGTGCTGCGGAAACCTTGAGATTGGAACTGTTCCAGTAACGCGTTTTCGATCGCGATACGCACATTTTGCTTAGAGTGAATTGGCTCAATGTTTGAGCGGCCACTATCTATTAGGGCTACATATTGAGCTGAACGTACGTCTTTACTTGTCAGAGTGAAACTTGCGTCTTTTACGATGTCGCTATTGCTCAGAACTGGCTGAGGCATAAAGTTGATCTGTTCTTGTTGCGGTGCGGCACACGCAGTAAGTAAGGCCATTGACGCGGCTAGAACCAATTTTCTCATTGTAATTTCCTTTAATCGTAACTCTATACGTATAAGAGACATGATAGTGAACTATTTAGTTGCTTGTAAAATAACAAATTTCTTATTTGAAGCGACAAGTTTTACGTTCGATTTTCCAAATAGCTTAGATAACTTCACATCGTAGCCGAGGTGACGGTTACCGATAACTAATAATTGCCCACCGTTGCTGAGAACATGCTTTGCATCACAGAACATCTGCCATGCGATATGATCGGTAATGGCTTGCTGCTGATGGAAGGGGGGATTACAGACCACAACATTAGCGCTGGCGTTTTCAAATCCGTCCAAGCAGTTGTTGGGAAGGCATTCTATTTTTCTGTCTTCGCCAAGTGTGCGGGTTAAGTTTCTCTTGGACGATTCGACGGCCATAAAACTTTCATCGACGCAAGTGAGCTGTATATTTGGGTTCAACATTCCCATCTTTACGGACAACACACCGTTGCCACATCCAAGGTCCACAACCTTTTCATCGCTGAGATTCTTAGGTAAGTGATCAAGCATAAATCGTGCACCCTGATCCAGCTTTTCGCCCGAATAGACATTAGGTAAGTTGTCGAGCGTGAAATGATGCTCGTCGATATTCCAGCGAGTTATAGGGTCTACAGAAATACTCGTCGAGCTATCGGCCTGAGAGAAAACCAGACGATGCTTTTTCCATGCTAGAGATGTGGTTGTCGTACCTAGGTACTTTTCAAACAGATTTAATGTAGAAGTATGAATCTCTTTAGCTTTGTTTACCGCAATGACAGGTACCGAAACGTCAAGCCTTTGTCTTAGCTGACTCAATTGCCATATTAAGTGGCGGTTGTTTCGCGGGATTTGCATCAAAACGTAATCCACACCTTGAGGAATCTCTTCCATGGTGTGAAGCAAGGTGATATCACGGCAGTGGTTACGCTTGAGGTTGGAGAGTGTTCCTTGATGAGCAATGTAGGAATCGCTCATCATGGTGATCTGATGTTTCTCCGACAACCAACAGCTCAGCGCACCAAAACTGTCATTCAGAATCAAAATGTGTTTGCCAGCGAGGCCAAGTTCTTCTATATGATTGATAAGGTATTCATCGCCAGCATCCCAGGCCTGAAGAGTTTCATTGTTGCGTTGGGGGAATCGATGCAAAGTTAAGGTGCGCTCATGGAGCACAAGTTCAGTTTTCATATTTGGAACGCTTGGGCAAATTCATGCGTAAATTGTCGCAGAAGGTAGGGCGTGTGGAAACTAAAACCTTTTGCTTTGCGTACAAGACGATTATTATCTGTTCTTTCATGCTGTTCATATTTAGATGGTGACACACAATGCTACAAGAAGTGATTGAAACTAAACTCCAAGAGCACTTCGAACCTGAATTTCTTAACGTGCTAAACGAAAGTTACATGCACAATGTTCCAGCGGGTTCGGAAAGTCATTTTAAAGTGGTGGTGGTCAGTGACAAATTTGAAGGTCAACGTCTGATTGGGCGCCATCGCCAGGTTAATCAAGTGCTGGCTGATGAACTCGCTAACCATATTCATGCTTTGTCGATGCACACATACACGCCATCGGAGTGGAAAGAGCAGAACGAGTTGGCCCCAGATAGCCCTATGTGTTTAGGCGGCTCTAAATAGTTGAAAGAAAAGAGGTGGCGTTTGCTGCCTTTTTTATTGGCTGTGATTTAAGTAAGCGTTAACAATGTAGAAACAAATAAACAGCTGAATTAGTTTTAACCAGAAATGTTTCACAATGTGAATAATTAAAAACAGCAAAGCCCGTGTGGTTATTGGCGATTGTGCGATGGTTCAAACTTATGACCAATACTTAGCCTTTTAAACTCAAATCCTTCGAAATATCTATCTGATTCCCTTCATTGGTCATGGCATAGACTTCTAAAAAGATGATAGACTATCGCACCTGATAAATCTCGACTAAATTTGTAGCGAGGTTTTTAATAGGATGTTGCGATTTTGGTGTTTTAAACTGCGCCAAAACCGGACACTAGTGTCGTGTCTAAAAGTTACGCAATCAAAGAATCTTTTTCCCGATAAAAGTAGTGCAAGTGCGTATGATTACAATAAAGAAGGGTCTGGACCTTCCTATCGCAGGAACTCCTACCCAGGTGATTAATGATGGTAAGACCATCAAGAAAGTCGCCTTGCTTGGCGAAGAGTACGTTGGCATGCGTCCTACTATGCATGTTCGCGTAGGCGATGAGGTAAAGAAAGCCCAAGTTCTTTTTGAAGACAAAAAGAACCCAGGTGTGAAATTTACTTCACCAGCAAGCGGTAAAGTGATCGAGGTTAACCGTGGCGCTAAACGTGTCCTTCAATCTGTAGTGATTGAAGTGGCAGGTGAAGAGCAGGTAACGTTCGATAAGTTTGAAGCCGGCCAACTAGCAGGTCTTGACCGTACCGCGGTTAAAACTCAGCTTGTTGATGCAGGTCTTTGGACAGCTTTACGTACTCGTCCGTTTAGCAAGGTTCCAGCAATTGAGTCTTCTACACAGGCTATTTTTGTAACTGCAATGGATACTAATCCGTTGGCAGCTCAGCCTGAGTTGGTCATCAATGAACAGCAAGATGCGTTCGTTGCTGGTTTGGATGTGCTTTCAACTCTGACAGACGGTAAGGTTTACGTGTGTAAATCTGGTACCAGTCTACCTCGTTCTTCTCAGTCAAATGTTGAAGAGCATGTTTTTGATGGCCCTCACCCAGCAGGTCTAGCTGGCACCCACATGCATTTCCTATACCCAGTAAATGCAAAAAACGTGGCGTGGAGCATCAACTACCAAGACGTTATTGCGTTCGGTAAGTTGTTCCTAACTGGTGAACTTTACACGGATCGTGTTGTATCTCTGGCTGGCCCAGTAGTGAACAACCCTCGTCTAGTTCGTACAACTCTAGGTGCAAACCTAGAAGATTTGACTGACAGCGAGTTAATGC

>NODE_13_length_4345_cov_2.005623

CCATTGAACTAGCAGCACAATACCAATAGCTGCCGCCATGAAACACAAACCGTAAAAGGCACTGATCAACGGCATACGCACAAAGTCGTGCAAGCCGAGTGACAGCCAATGAAACGGCGCGGATAAGCTGACATGATTGCACGGTATAGTACGTGCGTATTCGTTATCTGGGACTTCCTTTCGTTTTTCGTTGAAGTCTTGTGGATTCACCGTACGAGGCATACATCCTCCTTGATCGACTCTGTAATAAGTCACCCATAGGTTCATGGGTGGCGTTTTGGAACTCGATGCCCGGCTTTGCGACTGCCATAATAAGCTGCGCATCTGACTTGGTCTGTTAAAAAGGAATTTTTGGCGAAGAATTATCCTTTTAACATATTGTTAACTATTGACCCCATGACAAAAAAATACAAATTTGTTGTTCGATTTTTGCGCACAAAAACAAAGGACTTCTGGTTACCCAGAAGCCCTTGAATAGCAAATTAGTGCGGTAATTTAAATTACTTAAGACCAGCGAAGTCGCGTAGAAGTGCAGCTTTATCTGTCGCTTACCATGGGAACTCTTCACGACCAAAGTGGCCGTATGCCGCTGTTTTCTTGTAGATTGGTTGAAGTAAGTTCAGCATTTCTTGTAGGCCGTATGGGCGAAGATCGAAGTGCTGGCGTACTGCTTCAACAATGATGTCTTGAGATACTTTTTCAGTACCAAATGTTTCCACCATGATAGATGTTGGATCAGCAACGCCGATTGCGTAAGACAGCTGAATCTCACAACGGTCAGCAAGGCCAGCCGCAACGATGTTTTTCGCAACGTAACGAGCAGCGTAAGCCGCGGAGCGGTCAACTTTTGATGGATCTTTACCTGAGAATGCCCCCCCACCGTGACGAGCAGCACCACCGTATGTATCAACGATGATCTTACGACCTGTTAGACCACAGTCACCCATTGGGCCACCGATAACAAAACGGCCAGTTGGGTTGATGAAGAAGTTAGTGTCTTTGTTTAGCCACTCTGATGGTAGAACTGGCTTGATGATCTCTTCCATAACCGCTTCGCGTAGGTCTGGCGTAGAGATTGAGTCACAATGCTGAGTCGAAAGAACGACAGCGTCGATACCAACGATTTTTCCTTGGTCGTACTGGAACGTTACCTGTGATTTTGCATCAGGACGTAGCCAAGGTAGTGTGCCGTTTTTACGTACTTCTGCTTGTTTTTGAACAAGTAGGTGAGAATACGTAATTGGAGCTGGCATTAGTACGTCTGTCTCGTTAGTCGCGTAGCCGAACATGATGCCTTGGTCACCAGCACCTTGCTCTTTTGGATCCGCTTTATCAACGCCTTGGTTGATATCTGGAGACTGTTTACCAATGGTGTTCAGTACCGCACAAGAATCGGCATCAAAACCCATGTCAGAGTGAACGTAACCGATATCACGAACGGTTTGGCGAGTTACCTCTTCGATATCTACCCATGCTGATGTAGTGATTTCACCACCACCCATCACCATGCCAGTTTTTACGTACGTTTCACACGCAACACGCGCTTTTGGATCTTGCTCGATGATTGCGTCTAGAACTGCATCAGAAATTTGGTCTGCGATTTTGTCTGGATGACCTTCTGATACCGACTCAGAAGTAAACAGGTGTTTAGCCATGAGAGCTCCACTTTTAATTCAAGGAAGTGCTCCATTATTAACGGCGGAACACTAAGAAATAATACTGATATCTGTAGGTGTTTCTACATCTAGACGTCTATTCTAATTTTCATAGCTCGAATTACAAGCTCTTTTTTAATTTAATACATATCCAAACGTTTGCTTCTAAAGAGTTAAATATTACTAACTTGGATAGAAATGGCAGAAAATAAGCGAGAATCCCCTGTATTCAGACTGTAAAACGTTTGCACAGCTTAGGCGCCTTTGAGAGAATAATCGCCCATCAAAATTAGTTTTCGCTTAACGCACTCAGGAGCAGACATGTCTTCCCGCAAACATCTAGCTAATGCTATCCGTGCCCTTAGCATGGACGGCGTACAACAAGCCAACTCTGGTCACCCAGGCGCACCTATGGGTATGGCTGACATCGCTGAAGTTCTTTGGCGCTCTCACCTAAACCACAACCCAGCTAACCCAGAATGGGCTGACCGCGATCGTTTCGTACTGTCAAACGGCCATGGCTCAATGCTGATTTACTCTCTGCTTCACCTTGCGGGTTACGAGCTGTCTATTGATGACCTGAAAAACTTCCGTCAACTGCACTCTAAGACACCAGGTCACCCAGAGTACGGTTACGCACCGGGTATCGAAACGACGACTGGCCCACTAGGTCAAGGTATCACTAACGCGGTAGGTATGGCGCTGGCTGAGAAAACTCTAGCAGCACAATTCAACAAAGAAGGCCACGACATCGTTGACCACTTCACTTATGCATTCATGGGTGACGGCTGTCTGATGGAAGGTATTTCTCATGAAGCCTGTTCTCTAGCGGGTACGCTAGGCCTTGGTAAGCTAATCGCATTCTGGGATGACAACGGCATCTCTATCGATGGTGAAGTGGAAGGTTGGTTCTCTGACGATACACCTAAGCGTTTTGAAGCGTACGGCTGGCACGTAATTCCAGCAGTAGACGGTCACGATCCAGAAGCGATCAACGCAGCGATTGAAGCTGCGAAAGCGGACCCACGCCCTACGCTAATCTGTACTAAGACCATCATCGGTTTTGGTTCTCCAAACAAATCTGGCTCACACGACTGTCACGGTGCTCCACTAGGCGCTGACGAAATTGCAGCAACTCGTAAAGCGCTAGGTTGGGAACACGGTCCTTTTGAAATCCCAGCTGACGTTTACGCTGAGTGGGATGCAAAAGAAGCAGGTGCAGCGAAAGAAGCGGCGTGGAATGCTAAATTTGACGCATACGCTGCAGCTTACCCTGCAGAAGCCGCAGAGCTTAAGCGTCGTCTAAACGGTGAATTGCCAGCTGAGTGGGAAGAGAAAGCAACACAAATCATTGCTGACCTTCAAGCAAACCCTGCAAACATCGCATCACGTAAAGCGTCTCAAAACGCACTAGAAGCGTTTGGTCAAATGCTACCTGAATTCTTAGGCGGCTCTGCTGACCTTGCCCCTTCTAACCTAACTATGTGGTCTGGTTCTAAGTCGGTATCGGCTGAAGATGCGTCGGGCAACTACATCCACTACGGTGTTCGTGAGTTCGGTATGACGGCTATCATGAACGGTATCGCACTACACGGTGGTTTCGTACCATACGGTGCAACTTTCCTAATGTTCATGGAGTACGCACGTAATGCAATGCGTATGGCGGCTCTGATGAAAGTGCAGAACATCCAAGTTTACACGCACGACTCTATCGGCCTTGGCGAAGATGGTCCAACTCACCAACCAGTTGAGCAAATGGCTTCTCTACGTCTGACGCCAAACATGAGCACATGGCGCCCATGTGACCAAGTGGAATCAGCAGTGGCTTGGAAACTGGCTATCGAACGTAAAGATGCACCGACTTCACTTATCTTCTCTCGTCAGAACCTTGCACAGCAAGAGCGTGACGCTGAGCAAGTAGCGAACATTGCTAAGGGTGGTTACATCCTGAAAGATTGCGCAGGTAAGCCAGAGCTTATCCTTATCGCAACCGGCTCTGAAGTTGAGCTAGCAGTAAACGCTGCGGCTGAACTGACTGCTGAAGGCAAGCAAGTACGCGTTGTTTCAATGCCATCAACGGATGCATTCGACAAGCAAGATGCAGCTTACCGTGAAGCGGTTCTGCCATCTGACGTGACGGCTCGTATCGCGGTAGAAGCGGGTATTGCTGACTTCTGGTACAAGTACGTTGGTTTCGACGGCCGTATCATCGGTATGACCACCTTCGGTGAATCTGCACCAGCAGGTGAGCTATTCAAGATGTTTGGTTTCACAACTGAAAACGTTGTGAACACAGCGAAAGAGCTTTTAGCTTAATCACTGAACCTTGATATGAAAACGCCCCGCTACACTAGCGGGGCGTTTTTTATTGAATCTCTTCTACATTCACTTCAGTACTGAACTTTACTGTGCGAGTAGAGACCGAAGTCTGAAATTTGCGAATGTTCTTATCCTGATGCAATACGCGCTCAATAAATTTTTCATATGCTTTGATATCTGGAACGCTCACCACCAACATGAAATCGTAGCCTCCCGTGATCTGATAACATTGAGTGACTTCCGGGGCTTTTGTTATCGATTGGCGAAACACGCTGTATATATCAGGTCTATCTCGCTCCATTTCGACTGAAACGATCAAGTTCATCTTATTACCGGCCAGTTCGGGATTAATGATTGAGACATCACCCGTA

>NODE_14_length_4335_cov_2.464068

CCGCCGACTTTTGCTAATGGAGTATCGAGAATGACATCGCCTTTCGTCACCATAGTTACAAGCTCCACTTCTAAACCAGGGTGTGCTGCTTGAAGGGCATCTCGTACGTAGTAGGCTTGCCAAAGAGCAAGAGGGCTTTTGCGTGTTGCAATTCGAATAGGAGTCGATTGTGTCATGATGGTCTTTGAATAACAGTTCAGATGGGCTAATCCTACCATTCTCCAAGAAAAAGTCTTACTGCTAGATCATTTCGATGAATGTCAGACGTCGGGCTAGCAGAAAATATTCAAAATAGTGTGATGAGCATCTCGTTTGTAAAATGGGCTATTATTACAAAAGGCGGATACGTACCACACTATGTCGCGAGCATATTGATTAACAAAACAATGTCGATTCGATCGTACATTTCTTTACCAATCGAAATAAAAGTGTTAAATTGATCACGTTTTGTTAGTGCTTTAGAACAATTTGTCGTCAGGGCACAGTTAGGAATCAACCAAGGAATCACCCTTGCAGGCTTATACAAAGACGCTTATTCAGAGACTTGATAACCTAAACCAGCAGCGTATTGAGCGCGCGCTGGCTCTTATGGATATGCAAAGTCAGCGTGTTTTCCATCTGATTCCTACCCTTCTTCACTTCAACCACCCAGTCATCCCCGGCTATTACGATTCTCAAGTTCCTTTTGGTGTATACGGTCTCGAATTCAATGAGGTGCAACAGCAGTTTGTTGAAGATACTGAACTGACGATAGGTCAGTCGCTTCGGACCAGTGCAGAGCCCGCTATTCTCGGCTTGTATACCATGGGTAGTACTTCGTCGATTGGACAGAGTACCTCAAGCGATCTTGATATCTGGGTGTGTGTCTCACCATCAATGAGCTGTGAAGAAAGAGAGTGCCTGACCAATAAGTGTTTGTTGATTACCGATTGGGCTCAGACTCAAGGTGTTGAGGCGAATTTCTTCCTTATGGATGAAGAGCGTTTTCGTACCAATCGCTCAGAAGAAATGACCGGCGACAACTGTGGCTCGTCCCAACACTTACTACTATTAGATGAATTCTATCGCTCTGCTGTTCGTCTTGCTGGTCAGCGGTTGCTGTGGCAAATCGTGCCGCCTGAGATGGAAGAGTGCTACGACGAATACGTTCGCGATTTATGCAGCCAAGGTTACATTGATTGCTCGCAGTGGATTGACTTTGGTCAGCTGAACCGTATTCCAGCCGAAGAGTATTTTGGCTCGAACTTGTGGCAGTTGTATAAGAGTATCGATTCACCTTACAAGTCGGTGCTAAAAGCTATCCTACTTGAAGCGTATTCGTGGGAGTACCCACATACCCAACTGCTTAGCATTGATACTAAGCGTCGATTTTTTGCCCATGAGCCTGATCTCTATGGCATGGATGCATACTACTTGATGCTAGAAAAGGTAACACGTTACCTTGAGCGTATTGGCGATTATACCCGTCTTGATCTGGTGCGCCGTTGTTTCTATCTCAAAACACACGAGAAGTTATCACGTGAGCCTGGTATTGGCTCTGTTGCTTGGCGCCGTGAGGCGATGAGTGACATGATCCAGAAATGGAACTGGGACCACTCAGTGCTGGTTGAGCTAGACGACCGCCGTAACTGGAAGGTTGAGCAGGTTAAAGTGGTTCACCATGCTTTGCTTGATGCGTTGATGCTGAGCTATCGTAATCTGATTCAGTTTGCTCGTCGTAACGACATTACTTCTGCAATCAGTCCGCAGGACATCAGTATCTTGGCGCGTAAGCTCTACGCGGCGTTTGAGGTGTTGCCGGGTAAGGTGACTTTGCTTAATCCGCAGATTTCCCCAGACTTGCATGAATCCGACTTGAGCTTTATTGAAGTGAGACCAGGTCGTACAAACCGAGCCGGCTGGTACCTCTATAAGCAACCTCTTCAGCCAGATCGCATTCTTGGTCAGCCATTCTTAGAGCACAATGAATACCTCAGTAAGTTGGTCGCTTGGTCATTCTTCAATGGTCTAATTACAGAATCGACGCGTCTGCACTCGGTAGTTCGTGATGCTCATCTGGATATCGATAAGTTCTATCAGATGGTCAGCGATTTGCGTAATACCTTCTCATTACGCAAGCGCCGCCCAACCATGCAGGCATTAGCGAGCCCTTGTGAAATCAGCCAACTGGCGATGTTCATCAACTTTGAAGATGATCCAACGGCTGAGCTGAGTGGCCGCGCGCTGAAAGTGGATCTGAAAACTACCGATATTTTTAGTTTTGGCTCAAAGCAGACTTGTCTGGTTGGCAGTGTGGATTTGGTTTATCGCAATTCTTGGCATGAAGTCCGTACGCTGCATTTCCGAGGCGAAACGGCGATGCTGGATGCACTGAAAACCATTCTGGGTAAGATGCACCAAGATGCGTTGCCGCCTGAGTCGGTAGATGTGTTCTGCTACAGTAAAAACCTGCGTGGTGTGATGCGTAATATGGTGTATCAGCTTTTAGCGGAATGCATTGATTTGCGCCTTAAGCCTATTGAGCAAGAGAAACGTCGCCGCTTTAAAGCGCTGCGTATTGCTCAACAAACGTATGGCTTGTTCTTTGAGCGCCGCGGTGTGTCTGTGCAGATGCTGGAAAACTCGGTCGATTTCTACCGCAGCATCTCGACCAGTAAACTCAAAGGTTCACCATTGCTGATGCTGGACAAGGAGCAAGATTACCAGCTGCCAGAAATAGTCGACAGCTTTGCCAGTGAAGGTCTGATCCAGTTCTTTTTTGAAGATTCAGATCAAGGCTTCAATATCTATGTATTGGATGAAGCAAACCGTGTTGAGGTGTACCACCAGTTCAGTGGCATGAAAGATGAGATGATTGCCAGCGTGAATAGCTTCTACACGTCGGTAAAAGATGATAATCAAATGTCGACTCAGTTTATTAACTTCAATTTGCCTCAGTACTATCAAATCGTTCACCCAGCAGAAGGTGACTCTTACATCGTGCCATATCGTAATGATGGCGCTAACTATTCCAAGCCAAGTCGGGCCGTGAATGCGTAAGTCGAAGCAATAAAAAAGAGCACTTAAGGTGCTCTTTTTTGTATCCGAGTTTTACGGGGCACTACACCCATTCAATCTCTTCATCCGAATGCTTTTCGCACTCCTGTTTAACCATCGCAATCAGCTCAATACCCGTTTTTGAACAGGTCCACTGATCCTCAACCATAGCGAAGTGGAAACCGCCAGACTTAGATGCCAGCCAGATTTCCTTCATCGGCTCCTGACGGTTAATAATGATTTGGCTACGGTCTTCAAACTCTAGGGTCATGACATTACCGGAAGTTTCGTAATCAATGTCCGCGCCTGAATCATCAATCATTTCTTCGATGATTTGCATTTGAACATCTGCCAGCTGATGAAATTCAGTCTCGTTCATCCTGTTATCCTATTGCTTTTCCTGAGTGTGGTGCGATTATAGGGGGCATTGAAAGATTAATCACGACACAACCCATGAAAAAATCACTAATCGCTCTGTTTCTTTTATCCGTTCTTGGTTTGGCTGGCTGCGGTCAGACAGGTCCTCTCTACATGCCTGAAGATGCATCTCAGAATGAGCAGCCTTCACAACAATAAATAATGACATAAGGGATAGCACTTTGGATTACTTCAACTATCAGGATGATGGCCAACTTTGGGCCGAAGATGTTCCACTGACAGACTTAGCAGAGCAATTTGGAACGCCACTTTACGTATATTCCCGCGCGACGTTTGAGCGCCATTGGAATGCTTTCGATAAATCCGTTGGTGAGCACCCACACTTGGTGTGTTATGCCGTAAAAGCGAACTCGAATCTGGGTGTGCTTAATACACTGGCTCGCCTTGGGTCTGGCTTCGACATTGTTTCTGGTGGTGAACTGGAGCGTGTTATTGCAGCAGGCGGTGAAGCGAGCAAAGTCGTTTTCTCGGGTGTTGGCAAAACGGCGCCTGAAATGAAACGTGCCCTTGAACTGGGTATCAAGTGTTTCAATGTGGAATCTGAGCCAGAGCTAGAGCGCCTTAATAAGGTCGCGGGTGAGTTAGGCGTTAAAGCGCCAATTTCACTGCGTATCAATCCAGACGTTGATGCCAATACGCACCCTTACATCTCAACAGGTCTGCGTGATAACAAGTTTGGTATTGCGTTTGACCGTGCGCCTGCGGTGTACCAGTTTGCGCAAGGTCTTGAAAACTTAACGATCAAAGGCATCGACTGTCACATTGGTTCTCAGCTGACGGACATCGAACCGTTTATTGATGCTACTGACCGGTTGCTGGCACTGATTGATGATTTAAAAGCGCAAGGGATCAACATTGAAC

>NODE_15_length_4301_cov_2.753788

GCTTTTGCGAGCAGTACCCATCCATCTCATTGTGTTGTGATACGAGCATCAGCCCTAAAGAAGATTTGTTCGATGGGTTTGATATTATCTTGAAATTCCATCGCGGACGTCTTGAAGATTCAAATCTGGTTGCGAGAGAGATTAAGCGCTGGCCGAGTGTTGTTGTCGGTTCTCCTAAACTGCTAGAGCGGTACCCGACACCTTTTCAGACGACTGATTTACAACACATTCCATGCATCAGCAGCCTGACCGCAATAAACGGAACTCCGTGGGTTTTCAAAAAAGATTGCGGCGAACTTAGTACTCAGAAAGTACGATCTTCGTTTAAGGTCAACAGTGGTCATTTGGCGAAAACGGGCGCGATATCTGGGCTAGGGCTTGCGATACTACCAATTGAAACGAGCCAGGCTGAAATCAGCTCCGGTGCTTTAAAGATCATAGAAATGGAGTACCAACCCGATGACTTGGTTCTTTATGCCTTTTACGCATCAAGAAAACACGTAGCCAATAAGATTCCTATGTTTATTGAGCATATTAAGCAGCATGCGAATTTTGGTAGCTGAACAGCGTTCTAATTCGCATGATGTATTCAAATTTGGCTGGCTGAAAGTAGTCTGGTCATGAGTGTAAGCAAGGGAACCCCGCAAATCTTAAGTTATCCAAACATTGGCGTTCCCAAGCTGAGATACTAAGAAGAGCAGGGGGACACACCTTCAGTCCGCCAAACCTTGTTCATATCATTTTGCCTCACGTAACGAAGTTAAATTAGAATTCGTAGAACAGACGTACACGAGCACCGAAATCATCTTCTTCAGCAAAGACAGATGCGTCCGATTGCTGATCCAACTTGTTGTCGACGGTTGTGTAGTATGTACCTAGAAGAATCGAAAAGTCTTGTACCTCCAGTACATCACGGAAAGCGTAAGATGCATACCATGTTGATACGTTTACGTCGCCTTCAGCCCACGAAGAAAACTCTTTATCTTCATACTCATTTATCGAGTGAATGTAACCCAATCCAAAGTTATTCCACAGTGCATTAACGCCTGCTGATAAGTTATTTTCGTCAACGGCATCTAAGTACGCAACGTTGGCATTTACTGTCCAATCACCGCTCGTCCAGTTACCTGTTAAGCCATAACCTGTACGATCCGAGATATCCACGCCATCAGCGACAACTGCGTTAGAAACGAGGTTGGTTTCAACCGAAGCAGCTACTTTGAAATCACCCATTTGGTAAGCCACAACTGGACGAACTAAAAACGAATCTTTGCTCGATTCGATCTCTTTACCGTGGTACTTGCCGTCAAGGCCGTCATCGAACAAATTAGAACGATCACCAAGCATAGCGCCAACTTCAATGTAGAGATCACCAAATGTTTGGCTATACATGATTTGTCCGTCAGCGCCTCGGCCACGGGCTTCTTTTGCTTGGTATGAATATGCAGCTCCATCTGAATACAGATCATTCGCTGTATCACCTGAGTATTCTAGGAATACATCAAGGCCAACAGGGAACATGTCGTAAGCTTCAAATCGCCCCGCTTTAATTGCCCAGCCATCTTTTTTACCAAACTCAAAGTACGCATCATCCAATGCAATATTGCCTGTGCTCTCAAACAGCGGCTGCGCTTTCACACCTACATAGTGTCCCTTACTTGTGTATTTTTCGCCTGCAAACTCAATTAGAACGCGGCCGTCTTGGTTAAACTCACTGTCGTCACTAATGTGTGATTCACGATCTTGGTAGTTGAAGTTCAGCTCCACGTTACCACCAACAGAGAAGTTGCCTTCTTCAGTATCAACAATGCTGATTCCAGCGTTCGCTGACATTGCAACCATTACTGCAGCCGCAGCAAGCGACATTTTTAATGTATTTTTCATGTTAAACCCTATATTTTGGCAATAAGAGACCCTCCCCGACGAGGGCCTAATTTTTTGTTTTGGAATAATTTACTGGTGGCTAGCGAGTAGCCTGTGTCGCAGACATGTAATGCAGTGTGCCACTTGCCTGATTGAGAGTGACTAGAGACAACTCATCAATGAAGATTCGAGATGTGAACACTGTCTTGCCGTTGTCAGCAAAAATTTCAATCGCTGAGTTATCGACAAAGACGCGAATGGTTTGCGCTTGCTCTAGACGATGAGCGTAGCGAACCGTGCCGTATGCTTCTGCATGTAGGTGAGATACTTGAGTACGGTCTAGAATTAACTCATCTTGATCGCCTTGGAACACCATCTGCTCACCTTTCTGGTTAGCCAGAGTCAAAACGAAAGACTCATCTACACTCAGCTCCAACTCGAATGCTCGTGACGTTAGCTCTAGAGGCTCAGAGTCTTCGACATCAATTGAACTTTGGCGCAGTGCTTCAAGCTCTTTCAATGGTTTTTGAATTAGGTAGTCGCCATCGATGATCAACTCACGTGGTAGCGTAAGCATATGCGCCCAGCCGTTTTTATCCGTCGGATAGTCGCTCTTTGAGTTACCCAACCAGCCAATAAGGATGCGACGTTGCTGGTTGTCCAGATACGTTTGTGGTGCGTAGAAATCAAAGCCTTTGTCTAGCTCTCGATAGCCTTGGTTTTTGAATACTTTCGATTCAAAATCAATAGGTTCACCCACCATATAAACCACAGAAAATACGTTTTTAAGGTCATATTTATTCTCTGAGCTCACGCCCTGTGGTGAGAAAATATAAACCCCATTTTCATCCGTCTCGTAGTAGTTCGGGCACTCCCACATGTAGCCAAAATCTTCTAACCCCACATCGATATCACCTTGGTGATGGTAGTCATCAATGTCGCTACCGCGGTAGTATGCCAACGTGCCCTTTTCGTTATGACCTTGAGCGCCAATCAGCATGTGATAATCGTTGTCACGACACACCAACACAGGGTCACGAATCTCTGCGGTATATGCGCTCGGATCGAAGTCAACCACGATACCTTTCTTTTCCACATTACCTTGCTTGTCCATAATCGCGAGAACTTGAGTCGGATGACGTTGGTCATTCTCCTTTTTCAAGTTCGCTGTGTAGAAAATGTACAGCTGGTCATCGTGTGGTACACCAACACCGGTATAACAACCATGATGGTCGTAATCTTGGTCTGGGTACATCGCGATACCGCGGTCAGTAAAGTTCACGAAATCTTTGGTCGATACGTGGTACCAGTGCTTTAACCCGTGCACTGGGCCAAGCGGGAACCACTGGTAGAAAATGTGGTGCTCGCCATTGAAGTAACTCAAACCATTCGGGTCATTCAACAAGCCGTGCTTTGGTGCAATGTGGTAGCTCGGATAAAACGGGTCAGTTTGTGTTTTGTGCTCAATATCCGCCAAATACTCCTCGCTCACCTCATGAATTCGGACAAAGCGATTTTGACGATTAGAAAAATCCATAACAACTCCAGAAAATACTATTCACTCAAATTAGTGGTGAAAAATTATTTAGGCTCTTTGTAAAGCAGCTTTGTTAGCGCGAAAGCAGAGGCTGCACCGACTAGCATCACAAGAACGTACATTCCTAGACCGCCGCCAAGATAAAGTAGGACACCCGGTAGCATGGTTGCGCCCGTACCCGCTGAGCCGATACCCGCAATTGCGCCCAAACAGCCACCTAACGCACCACCAACACAGCCGTATAAGAAAGGTTTTACGCGAGGTAAGGTAATCGCGAACATGGCTGGCTCAGTAATGCCAAATAGAGCTGGCAGTGCGGCACCAAACATTCCCGTGCGTTTAACCTTGTCTGTCTGCATTGACATTACAGCCATCGCAGCACCGAATTGACCTGCAATAGCTGCCGTACCCAATGGGTTGATGTAATCAAAGCCTGTATCCGTCACCATGGCGATGGTGATCGGCACGATGGTATGGTGAATCCCTTTTACCGCCATAAACTGGATAGCGCCACCGTAAATCAAGCCGCCAATGCCGTATGGGATTTGTAAGAAGTAGAGCACGGCTTCAGTAATCAGTTTCTCAATTCCGAGTAGGATTGGACCAACGAACACCAAACCTGCAATCAAAGAGACCAACAGAGTGATAAATGGCGTTAGAATGAGATCCAATACTTCAGGGATGTATTTGCGTGATGTACGCTCAACTAGAGCAGCGAACCAACCCATAAACACCGCTGGAAGAATGGAACTCTGTAAACCAGTTACCGCAATCTCAAAGCCCATAAACGGAATCATCATCGCTTCTGCGTTACCGAAAGCGACATCCCATTTACTAGCTAGTTGCGGAGCTACCAGCATCAAACCCAAAACAATACCGAGCACTGGATTACCACCGAACA

>NODE_16_length_4288_cov_2.865115

GTCTTGAGGGGGGACTGTCAGGTAACCTCAATGTTGCTCAGCGTGATAAAGGGCCATTTGTAACCGGAGAAGTGAACATTGTTGACGGTTCGTACCGTTCCTTCGGTCAGGACCTTTTGATTAAGCAAGGTAAGATCCTGATGAACGGCCCGGTTGACCAACCTTATGTCCAGATCACCGCGATCCGAAATCCGGACAATACTCAGGATGAAGTGACGGCTGGTGTCAGGGTGACGGGGCCTGCTGATGAGCCAAGTGTGACCATTTTCTCTGAACCTGCGATGCCGCAGGCTAATGCACTGTCTTACTTACTACGTGGGCAGAATATTGATGGTGAATCTGGTGGCAACTCAATGACGACCACGCTGATAGGATTGAGTCTGGCACAAAGTGGTCGTGTCGTGGGTCAAATTGGTGAAGCGTTTGGTGTTCAGGACTTGCAAGTTGACACCGCGGGCTCTGGTGATGACTCGCAAGTGACGGTCAGTGGTTATGTTCTACCGGGTTTGCAGGTTAAATATGGTGTTGGCATCTTTGACTCAGTGGGTGAGTTCACGGTGCGTTATCGTCTGATGCAGGATCTGTACGTGGAGGCGATGTCAGGTCTGGACAGTGCTGTCGATCTGCTGTATCAGTTTGAGTTCAATTAATCATTAAGTTAGGTGGCAGGATGCAACATTTACTCTTTGTGTATGGAACCCTACGTCAGGGCGAAAGTAACCATAATTTTTTGGCTGATAGCCAATGCTTGGGGCATTTTGAAACACCGCCGCACTACGCTTTGTATGATTTGGGCACCTATCCAGCAGTAATCGAAGGTCATGATACGATTCTAGGTGAAGTGTACCTGATTGATGATGAGACTCTGGCTAGGGTGGATAAACTCGAAGACGTGCCCGTCGAATATCGACGCGAGCAGATTGAGACACCTTTTGGTGAAGCTTGGATATACTTGTATCAAGACGGTAGCATGCTCGATACCATCATTTCATCAGGGGATTGGTGTCAGAGGGTTTAGCTGCTATAGCCATGGAAAAAGAGCCAGCATTGCTGGCTCTTTTTATTATTTCTGTGCGCGTTCGTAAGACTCTAGAATCTCTTTGCGAGCTGACTCTACGTCTTCCCAACCGTCGACTTTTACCCACTTGCCAGACTCAAGCTCTTTGTAACGCTCAAAGAAGTGCGTGATCTGTGCTTTTAGCAGCTCAGGCAGGTCGTTTACGTCTTTGATGTGATCGTATTCTTTTGACAGCTTAGTGTGTGGTACCGCCACTACTTTCGCATCTTCACCAGACTCATCCGTCATTTTTAGAACACCGACTGGACGGCAGCGGATTACTGAACCAGGCATCAGAGGGTGTGGTGTTGGAACCAAAACGTCTACCGGATCACCGTCTAGAGACAGAGTGTGGTTTACGTAACCATAGTTACATGGGTAGAACATAGGTGCAGACATAAAGCGGTCGACGAACACAGCACCTGAATCTTTGTCTACTTCATATTTGATTGGATCAGCGTTAGCTGGGATTTCAATCACTACATAGAGGTCATCAGGAAGAGACTTACCTGCTGGTACGTTGTTTAAACTCATTAAGAAAGTTCCTTTTTTAGTTTGTACCTCAATGAGGCACACGGGCTGAATATAAGACCGGAATACCTTACTGCTAAACTCGGTTTTATGTAAACCAACTAAGGCGAGATTTTGCTCAAGAAAATGTGAAAAAGGCGCTCGAAAGCGCCTTTATATTTAATCTTCTGGATGGCGTTCTAAGAACTCTTCCACTTTTTTGACCATGTTTTTTGAGCCAACAAAGAAAGGCACACGTTGGTGCAGTTCGGTTGGTTTGAGGTCCATGATACGAGTTTTACCATCAGACGCTACACCGCCAGCTTGCTCAATGATATAAGCCATTGGATTGCATTCGTATAGCAAACGCAGCTTACCGTTTGGATGGCTTTGCGTACTTGGGTAGAGGTAGATACCACCCTTAAGCAGGTTGCGGTGGAAATCGGCAACCAGTGAACCGATATAGCGCGATGTATATGGGCGATGCTCACTTGGCTCGTTTTCCTGACAGTATTTGATGTACTTCTTCACACCCATCGGGAAGCGAATGTAGTTACCTTCGTTGATCGAGTAGATGGTACCGTCTTCAGGAATCATCATGTTTTCATGAGACAGGCAGAATGTGCCCAGAGATGGATCGTAGGTAAAGCCATTGACGCCTTTACCAGTTGTGTACACCAACATCGTTGAAGAGCCGTAGATTACGTAACCGGCAGCGACCTGCTTGTGGCCTGGCTGAAGGAAATCTTCTTCTGTCGGTGGGGTGCCAATAGGTGACACACGACGGTAAATCGAGAAGATGGTACCGACTGACACGTTGACATCGATGTTTGAAGACCCATCTAGCGGATCCATCAGGACCACGTATTTCGCGTTTTTGTTCAGCTCTTTATTGAACGCAACGGCTTCGTCTTCTTCTTCACTGGCAACACCACAAACTTGATCACGGGCTTCTAGCGCTGCTTTAAACTTTTCATTCGCGTAAACATCCAGCTTCTGCTGGTCTTCACCCTGTACGTTCTCAGTACCGACTGCACCGGTAATGTCACCAAGACCAGCTGCGTTGATTTCGCGGTTAACGATTTTTGCAGCAAGTCGGATAGAAGAAAGTAGGGATGATAGATCACCGCTAGCGTGGGGGAAGTCCGCTTGTTTCTCAACAATGAACTCGCCAAGGGTGCGCATTCCAGACATGATTTTTCCTTTAAGTCGCTTCAATATAGGGGGATTGGCGTTGGTGATCTCTGACGGATCTTAGATTTCGCCTAAGCCTAGTCTAAGAGTTAGATCTTTTTATTGGTAATGAACTAACTGACTGAGATCTCGTTTTATTTGTCGACTTGATAACGCTTTTCATCTGCTTGTTTGAAGCCATACGAATTGAATAACGCGTATAACACGCAATCATTTGCGTTCGTGTGACGTAAAAACAGTGAGTAAAGTAGTTCGCTTTTCTGCGTATAATCGCCATAATGTCAGCTCAGTTTTCGCCTTGGAGAAGGTCGTTTATGCATATTCATATCTTGGGAATTTGTGGCACATTTATGGGAGGTGCGGCAATTTTAGCTCGTCAATTAGGTCATAAAGTGACGGGCAGTGACGCCAACGTTTATCCACCAATGAGCACATTGTTGGAGTCTCAAGGTATTGAAATCATTGAAGGCTTTGACCCTTCTCAGCTTAACCCAGCACCTGATCTGGTTGTGATCGGAAATGCGATGAGTCGAGGCAACCCATGTGTCGAGCACGTACTCAACAGTAACCTGAGATACACCTCAGGCCCACAGTGGCTGCAGGAGTTTCTTCTCCATGACCGCTGGGTGCTGGCTGTCTCAGGAACCCATGGTAAAACCACTACTTCCAGCATGCTGGCGTGGATTTTGGAAGACTGTGGCTACCAACCTGGCTTCCTTGTTGGTGGGGTGCTGGGCAACTTTGGGGTTTCAGCGCGTTTGGGCGAAAGTATGTTTTTTGTCGTTGAAGCTGATGAATACGACAGTGCTTTTTTCGATAAGCGTTCTAAGTTCGTGCATTACCATCCTCGCACGTTAGTTATGAACAATTTGGAGTTTGATCATGCGGATATCTTCGATGATCTGGAAGCGATCAAGCGCCAATTCCATCACTTAGTCAGAACCGTGCCGGGTAATGGTAGAATCCTTGCTCCGCAAGGCGATTCAGCAATTGCCGATGTATTACAGCGTGGTTGCTGGAGCGAAACGGAGTTCAGCGGCGAACAAGGTGACTGGCGAGCGGAGAAACAAGTCGCCGATGGTTCAATATTCACAGTTTTCTTCCAAGGGGAAGCCGTTGGGACTGTGAAGTGGGATCTGGTGGGCGATCATAACGTCGATAACGCCTTGATGGCCATCGCAGCGGCTCGTCACGTGGGTGTTACTCCGGATTTAGCCTGTCAGTCGCTGGCGAAATTCATCAATACCAAACGTCGACTTGAGCTAAAAGGTGAAGTGAATCAGATTACGGTTTACGATGACTTCGCTCATCACCCTACGGCGATTGAACTTACTGTGGGTGGATTGCGTAATAAGGTTGGCAAACAACGTATCTTAGCTGTACTTGAACCTCGTAGTGCGACGATGAAACGTGGCGTGCACAAAGAAACATTGGCGGCGTCCCTTGCTCAGGCCGACGTCGTCTTCCTTTATCAACCAGATAATATCGAATGGTCTGTCGAGGACATTGCCCAGCAATGTCAGCAGT

>NODE_17_length_4180_cov_2.603461

CCTCTAGTAGAGGGCGGCAAAGTAGTTGCTGAAGTTGTACAACACGGTCGTGGCGACAAAGTTAAAGTCGTTAAGTTCCGTCGTCGTAAGCACTCTCGTAAGCAACAGGGTCACCGTCAGTGGTTCACTGAAGTGAAGATCACTGGTATCAACGCTTAATTTATTAGGAGAGTTTAACAATGGCACACAAAAAAGCTGGTGGTTCTACTCGTAACGGCCGCGATTCAGAAAGTAAACGTCTGGGTGTTAAGCGTTTCGGTGGTGAATCTGTTCTTGCAGGTAACATCATCGTTCGTCAACGTGGTACTAAGTTCCATGCTGGTACTAACGTTGGTATCGGTAAAGACCACACTCTATTCGCTCTTACTGAAGGTAAAGTGAAGTTCGAAGTGAAAGGTCCTAAGAACCGTAAATTCGTAAGCATCGAAGCTGAGTAATTCTCGCTGAGATTAAGAATTTAGGCTTCTAGCTGAATTCAAAAGCCCTGCCGATTCGGCGGGGTTTTTTATTTGTAGCGAGATGGAATCGATACTAAGGCACTTTGTAAAAAGTCCCTCATTATATATTCCTGAGTAGCAGAACGATCTGGGATTTTAGGTGATCGATCTGAAATTGGGATCTGCTAGAATTTATATCATTCATTTGCCTAAATACGGTTAGTGAGATGATATTTGCAACGCAGCTACGTGATGTAGCAATTGGGCGGAGTAAGAGATGAAATTCGTTGATGAAGCGGTAGTTAAAGTTCAGGCCGGAGACGGCGGTAACGGTGTAGTGAGTTTCTGGCGAGAGAAATTCGTTGCGAAAGGCGGTCCGGACGGTGGTGACGGTGGTGATGGCGGCGATGTTTACATCCAAGCTGACGAAAACCTTAACACGTTAATTGATTATCGCTTCCAGCGCTTCTATGAAGCAGAACGTGGTGAAAATGGCCGTGGCGGTAACTGTACTGGTAAACGTGGTAAAGACAAAGTTCTACGTGTGCCTGTTGGTACTCGCGCTGTGGATATTCACACCAATGAAATCGTGGCGGAAGTTGCTGAGCATGGCAAAAAGATCATGGTCGCAAAAGGTGGTTGGCACGGTTTAGGTAACACGCGTTTTAAATCGTCAGTCAACCGTGCACCGCGTCAAAAGACGTTAGGTACTAAAGGTGAAATTCGCGAAGTTCGTTTAGAGCTGTTGCTACTTGCTGATGTGGGTATGCTTGGTTTGCCGAATGCAGGTAAATCGACATTTATTCGTTCTGTATCAGCAGCTAAGCCAAAAGTAGCTGACTACCCATTTACGACTTTGATCCCTAGCTTAGGTGTAGTCAGTGTTGTACCTGAGAAGAGCTTTGTTGTCGCGGATATTCCTGGATTGATCGAAGGCGCTGCGGATGGCGCTGGCCTTGGTATTCGCTTCTTGAAACATTTAGAGCGTTGTCGAGTTCTACTGCATATGATCGACATTATGCCAATTGATCAAAGTGATCCTGTACAGAATGCCTTAACCATCATTGATGAGCTTGAGCAGTACAGTGAGAAGTTAGCAGACAAGCCGCGTTGGCTTATCTTCAACAAGGTTGATTTGATGCCTGAAGAAGAAGCGAACGAAGTGATCCAAAATATTCTCGATGCTCTAGGTTGGGAAGAGGACTACTACAAGATCTCCGCGGTGAATAAGCAAGGAACCAAAGAGCTTTGTTACAAGTTGGCTGACTTTATGGAGAATCTACCTCGTGAAGAAGAAGAGATCTCTGAAGAAGAGAAAGTTGACTTCATGTGGGATGATTACCATAAAGACGCAATGTCAGGCAAAGACGTCATTACTGAAGACGACGATGACTGGGATGATTGGGACGATGAAGAAGACGACGGGCATGTCGTTTATGTCCGCGAATAATCCTTAAGTTGATTGAAAGCCGCAATGTTTAAAGCATTGCGGCTTTTTTGTATTTAAATCAAATCAATCGTCGGCGTGATTCGCCACAATAGCAGGATTCTAAAGGAGTAGGATTGATTTATGGTTTCAAAACAACGCAGTGTTTCTCGCCTTATCGCTCAAGCCGGGCAAATGCTGCTTGCGCATGGTGCAGAGAGCACATTGGTAGGGGATATTACTCGACGCATCGGACTTGCGAGTGGCATGGATGAGGTGGAAGTTTCTTTGTCTGCAAGTTCGTTAGTAGTGACGACTGTGTATCAGGAGCACTGTATTACCACTGCACGCCGCAGCCCTGATCGCGGAATCAACATGAGGGTCATCACCCAGATACAACGGATTTGTATCATGCTCGAAAGGGGAATCATAGATCATGCCTTGGCTCAGCATAAACTGGAAAGAATTAGTCCCGAACGTTATAACAGATGGCTAGTTGTAGGTATGATCGGCTTATCTTGCGCTGCATTTAGTCGACTAGCGGGAGGGGATTGGGTGGTCTTTGCTATGACATTCATCGCTTCTTCGATCGGAATGATAGTCAGGCAAGAAATCGGCCATCGCCACTTTAACCCCTTGATGAACTTTGCCGCGACAGCGTTTGTTACAACTGTTGTGTCAGCGCAAGCGGTAATTTATGGGCTAGGTAATTCGCCGTTTTTGGTTATGGCCTCTTCGGTACTTATGCTGGTGCCAGGGTTCCCGCTAATTAATGCTGTCGCTGATATGCTCAAAGGCTGTATCAATATGGGCATTGCTCGCTTTGTTATGGCAAGCCTGCTTACCTTGGCAACCTGCCTTGGTATCGTCGCGGCCATGAGCCTAGTTGGTGTCTGGGGGTGGGTGTTGTGATGTGGTTTGATTTATTGGTTGCTTTGCTCAACGACATGCTGTTTGCCGCGATTCCTGCTGTGGGTTTTGCTTTAGTCTTTAATGTCCCCCAAAAAGCCTTGGTCTATTGTGCTATCGGGGGGGCAATTGGTCATGGTTCACGTTATTTGATGATGCATTTTGGTATCCCTATCGAATGGGCCACCTTTTTTGCTGCGATGTTGGTTAGTATTGTTGGTATCCATTGGTCTCATCGCTTTCTCGCTCATCCGAAAGTGTTCACCGTAGCGGCGTTGATTCCTATGGTTCCAGGTGTATTTGCGTTTAAGGCAATGATTGCCTTAGTGGAGCTTAATCATAGAGGGTACAGCCATGAGTTGGTCGCGATGTTGGCAGAAAACTTCCTTAAAGCAATGTTTATAATTGCGGGATTAGCGGTTGGCTTAGCGATGCCGGGATTGCTATTCTATCGCCGGAAACCGATAATCTGAGACGTTTTAAAGGAGCATCAGCAATGATCATCAGTATGATCGCCGCCATGGCAAAAGATCGAGTAATTGGTAAAGACAATCAAATGCCTTGGCATCTACCCGCAGATTTTGCTTGGTTCAAACGTTGTACTATGGGCAAGCCTGTTGTTATGGGGCGTAAGACGTACGAGTCGATTGGTCGACCACTTCCAGGTCGTCAGAATATTGTGATCAGTCGTGATGCTAACTTAGCCATTGAAGACGTCACGACAGTGACCTCTATTGACGACGCCATAAAAGCTGCTGGTGACGTGGAAGAAGTGATGATCATTGGTGGTGGTGCGATTTATAAAGCCTGCCTTCCTCAAGCGGATAAGCTCTACGTGACTTATATCGATGCCCAAATAGAAGGCGATACCCAATTTCCGTCTTGGGGAGATATGTTTAAAGAAAGCCATTCTGAAAACTACGCAGCGGATGAGAAAAATGCGTACGATATGCGATTTGTTGTTTTAGAGCGACAGGGCTAGCAGATTAGTTAACAGTTTATGATCTGGAGTAGCTGATTGTTGTCGGCTCTCTAGCTCTCTAGCGAATTTTGTGTAAAAAACGTCTTATCTTCCCATCTCAACATCGTCAAGCTGCCACCCCATACGCAGCCGGTATCTAATCCAATGACATCCTTGCCGGTATAACCTTCCAATGCAGCCCAGTGGCCAAACAACACTGTCTTATTCAGCGGAATACGATTGTTAAGTTCAAACCATGGCGTTAAATGACTATCTTGGACGTCTTTTGGTGGACGCTTACAAGCCATGTCTAACCTTCCGTCGGAAAAGCAGAAGCGCATTCGCGTGAAGGCGTTAATGATGTAACGGTAGCGCTCAATGCCTTCGAGCTTGTCAGACCATGAGTCGGGTTGATTATCGTACATGTTTTTGATCAGCCAATGCCAT

>NODE_18_length_4169_cov_2.515396

GTCGTTGACGTTCGCTTCATGAATCATAGGGGTCGATAAGTTTGATTTCCATAATATATTAAACAGTGTTCAATTAAACCCCTAAAACGAAAAAAGCTCCCAATTTGGGAGCTTTTCTTACAAATAAGAGCTAGGTTTATTTGTTACGACGCGCTTCTACCGCATCGGCTAACTGACGAAGAACCTTTTCAGTGTCTTCCCAACCAATACATGCATCAGTGATAGACTGACCGTAAGTCGGTGCTTGACCATCAACAAGATCTTGACGACCTTCAACTAGGTGTGACTCAATCATTACACCGAAAATAGCATCTTCACCTGCAGAGATTTGACCCGCTACATCTTCCGAAACCAACATTTGACGCTGGTACTGCTTCGAGCTATTCGCATGGCTAAAGTCGATCATTACCTTCTGAGGCAGACCTGACGCTTCAAGTTCAGATTTAATTGCACCAACATGCTCAGCACTGTAGTTAGGCTCTTTACCACCACGTAGGATGATGTGGCAGTCTGGGTTACCAGCAGTTTCAACGATTGCTGAGTGACCGTACTTAGTGACTGACAAGAAGTGGTGAGAAGCACTCGCACTGCGGATTGCATCTGATGCAATTTTGATGTTGCCATCAGTGCCGTTTTTGAAGCCGACTGGGCAAGAGATACCTGAAGCCAGTTCACGGTGTACCTGAGACTCAGTAGTACGTGCGCCGATAGCACCCCAACTGATTAAGTCTGCAACGTACTGCGGAGTGATCATATCAAGGAATTCACTCGCCGTTGGTAGGCCAAGATCAGTTAGATCCAGCAGCAACTTACGACCCATGCGCAGACCATCGTTGATCTTAAACGTGTCGTTCAGGTACGGGTCGTTAATCAGACCTTTCCAACCCACAGTTGTACGTGGCTTCTCAAAGTAAACTCGCATCACGACTTCTAGACGGTCACCTAGCTCATCACGCAGTACTTTCAAACGCTTACCGTATTCGATTGCTGCTTCAGGGTCGTGAATAGAACAAGGACCAACGATAACCAGCAGGCGGTCATCATCGCCGTTTAAAATCTTAGAGATTGCTTCACGAGAGCGAAAAGTCGTTGAAGACGCAGTTTCAGTCGCCGGAAACTTCTCTAAAACAGCTACTGGCGGTAATAATTCTTTTACTTTGCTAATTCGTACATCATCAGTCTGGAACATTGCTTACGTCTTCCTATTTCTCTTGGCTTGAACACCGCACCAAAAATTAGTGGTGCATAAATGCTCTATTTTGTTTTTCATCTTCCCTGTAACTTATCTATCAAAAACGATGCTTGCAACCACTTTTTGAACCAAAAGGCAATATTTCTTCTATTTTTACATTTTACACCGTATGTAAAATTTAAATTACATACGATAAAGAGTGAGCAATATCAGGCTTTCTAGCTCTATTTGAACCGCATGAGATGCAATATCTCTTTGATATTGTTAAGGTAGAAATACAAATAAATGCATATAAAGAACCGGCATGAGCGCCATCGATTTTACACACGTCAGCAAGTGGTACGGCCATTTTCAGGCACTGAAGAATATTAGCCTCTCAGTACAAAAAGGAGAGAGGTTAGTCGTTTGTGGCCCCTCTGGTTCTGGAAAATCGACCTTAATTCGTACAGTCAACGGGTTGGAAACCATCTCAAAAGGCAATATTCAGGTGCTCGGCCAGCCTTTGTCCTCTGTCTCTCCGGGAAAAGTCGGCATGGTTTTTCAGCATTTTAATCTTTTTCCTCACTTAACCGTACTAGAGAACCTGACGCTGGCACCGATGCGCACATTAAAGCAATCAAAACAACAAGCCATCGATACAGCCATGCACTTTCTAGATCGCGTCAACATCACGGAACAGGCAAACAAATATCCCGTTCAATTGTCTGGCGGACAGCAGCAGCGTGTCGCGATTGCACGCTCTTTGTGTATGAAGCCCGAGATTTTGTTGTTTGACGAGCCAACCTCCGCTTTAGATCCAGAGACCATCAAAGAAGTTTTGGAAGTCATGACAGATTTAGCCACAGACGGCATGACAATGATATGCGTAACACATGAAATGGGCTTTGCCCGAAAGGTGGCCGATAGAGTGATATTTATGGATCAGGGAGAAATACTCGAAATGGCTACTCCTGAGCAATTATTTAGTGCGCCGCAACACCCTAGAACACAACAGTTTCTGGAGCAAATCCTGAGCCACCAATGATTAGCCGAATTTTAAAGCCCGTTTTGTCCGCCTTCGCTCAAATCCTAATACTAGGTTTAGGGCTGTGGTGGCTGCTGGATTCGGGTGCGAAAGCAATAGATTACCAGTGGCAATGGTACCGAGTACCTGACTATCTCGCCTTCTATGAAGATGGTGAATGGTGGCCTGCTGAACTGCTCGAAGGCTTGTGGGTCACTATCAAGATATCGGCATGGAGCCTATTGTTTACTCTGCTGATTGGAATGCTGACGGCTTTGCTTAAGCTTTCTCAATCTGCTGTAGGTCGAACCATCGCAAATACTTACATTGAGCTGATTCGCAATACGCCGCTTCTGGTTCAGATATACCTTTTGTATTTCGTCTTTGGTCCAGTGATCGGCCTCGATAGATTTGCCACTGCGATTCTAGCGCTCTCACTTTTCCAGGGTGCCTACACCGCTGAGATTTTTCGTGGAGGCCTAAACAGCATCCCTAAAGGTCAGTTTGAGGCTGCTCGCTCCCTTGGGCTAAGCCCTTTCTCTACCTACTACGACGTTATCTTCCCGCAGTTACTGCAAAGAACGTTACCGCCTTTGACCAATGAAGTGGTTTCTCTGGTTAAGAATTCATCAATTGTCAGCGTAATGGCCATATTCGACCTGACGACAGAAGGAAGAAACATTGTTTCGGAAACCGCTATGCCATTTGAAATTTGGTTTAGCGTGGCAGCGATATATCTTTTGCTCACACTGTCACTCTCGGGAATCTCCGCTTGGCTAGAACACAAACTTGGCGCCCAATGGCGTGCTCAATAATAAGGAAATCAAATGAAACACTTAGGACAAAAGATTCGTTCGTTGAGAGCTTTCAAGCAAGCGATTACCGCTTTGCTTGGGCTAGCAGTAGCGCTACCAACGCTCGCCAGTGAAACCCCAAATTTGGATAAGATTAACGAGCGCGGCACCTTACGCGTCGGAATGTCGACCTTTGTCCCATGGGCAATGCGCGACAAGCAAGGGGATTTAATCGGCTTCGAAATCGATGTAGCTGAACGTCTTGCCGCGGATTCTGGCTGGAAAGTCGAATTTGTACCCACCGCTTGGGATGGCATTATTCCAGCGCTTCTGGCGAAAAAATTTGATGTGATTATTGGCGGCATGAGCGTGACTCCCGAACGCTCTAAGAGTGTCTTATTTACCACTCCTTATTCACACTCTGGTGTTCAGGTGGCTGCCAATAAAGAACTGGCTTCAGGCTTTTCTGAAATGAGCGATTTCAATTCTCGACGAGTTAAAATCGCTGCACGCCGTGGCGCCTTCACGGTACAAGTCGCTCGAGAAGCTTTTCCAAAAGCTAAAATTCTTCAGTTCGACGACGATGCTCAAGCATTTCAGGAAGTCCTCAATGGTAACGCTCACGCTGTGATTGCTTCTAGCCCTAAACCTGAACATGAAACTGTGAAGAATGCCGACAAACTGTTTATCCCGTTTAATGAGCGCCTGTCTAAGGGCAACGAGGCTTTCGCCGTTCGTCTTGGTGAAAGCGACAAACAAGCCTTCTTCGACCAGTGGATACAAGCTCGTACCGAGGATGGCTGGCTTGAGCAAAGATACGAGTACTGGTTCGCGACGCTCGACTGGCAGCAGCAAGTTGCAACCGGTCAATAACCACGTTTCTTAAACCTAGGATGATTGATAAGACATAAAGATTAGCAGGACGTTAGGTGAATAAATCGAATTTACTGACCGCCAGTCAGGACAGAAAACCCAGCTTAAGCTTCAATAAGCTAGATTTGGTTTTATTGCTTGCTTTAGCTGTGTTTGGATTCTGGCTTTACCAACGCGCTTCTATCGGAGTGAATTACACTTGGCGATGGAGCGAAGCATTCACGCTGATCTTTACTTCACGCTCTGATGGGTCGTTGCCCTACTTCATTCAGGGTGTGATCTCGACATTACGGCTCAGTGTTTGGGG

>NODE_19_length_4150_cov_2.073410

CTCTTAACCCGGCAATTGATATTGGCCAGGTCGAAGGTGGCTTCCTGCAAGGCGTGGGCTGGCTGACCACAGAAGAGCTGATCTGGAACGAACAGGGTCGTCTGATGACTAATGGGCCAGCCAGCTATAAGATTCCGGCGATCGCCGATATGCCTATCGAGTTCCACACTCACCTTCTGGAAAACCGAGCCAACCCAGAAGATACCGTGTTTAACTCCAAGGCTGTCGGTGAGCCGCCATTCATGCTCGGCATGTCAGTGTGGAGTGCACTGAAAGATGCCATTGCCTCAGTTGCCGTTGAAGGTGCTATACCTAAATTAGATACACCAGCAACACCAGAACGTGTGTTGATGGCAATCAATGAGGTAACGCAAACGCAACAACAAGTGGCAAAACAGAACACGCAAGAGGTTTAAGCGGTTATTCAAGGGTTTACCGTTCAAGGAGAGACGTATGTTTAAGGACAATTGGATTCATGAACTGGCACAGCTAGAGAAAAACAGTGAACCTTGCGTGATGGTGACCGTATTGGAAGACCGTGGCTCAGTGCCACGGGATGCGGGCACAAAAATGTTGGTCACTCGCGACCGTTTGATTGCCACCATTGGTGGTGGTCATCTGGAGCACATCGCCACCAAAATGGCGCGTGAAATGCTGCTTGCGGGTGAACGTTCACTGAAAGTGGAGCGCTTTAATCTCGGGGCTCGTTTAGGTCAGTGTTGTGGCGGAATGGCAACACTGAGCTTCGAACCAATTGGTACCGCGCAAAAACATCTAGTGGTATTCGGTGCCGGCCATGTAGCCAAAGCCCTTTTGCATATCGTCTCGACTCTCCCTTTCCGTGTCACTTGGATTGATGAGCGTGAAGACGTATTCCCTGACGAACTCCCTCGCAACGTAACCAAACTGGTGAGCGATGATCCGGTCGCTGAAGTGCGCGACATGTCACCAAACAGCTACTATCTGGTCATGACACACAATCATCAACTCGATTTTGAGTTGGCCAAGGCCATCATCGATCGCGACGACAGTGTGTATTTCGGCATGATAGGTTCACTAACCAAGCGCAAAAAGTTCGACATGCGTTTGGCGCAGCGAGGCTACAACCAGCAACAAATAAATACGATGATTTGTCCAATTGGAATCAGCATGGTGAATGGAAAACATCCTGCTGAAATTGCGGTATCGGTTGCGGGTGAATTAATCGCACACTATCAGGGAATTTCGCTTGAAGAAAAACGCCCTACGCCACACAGTAAAGAAGAACAACTGAGCGATTCATCATTGGAAGAGAAAATCGCCTAGCCCCAAATAGGGCCTTTAAAGGAAGTAAACATGACAACTCAACGTAAAGCTTATCGTGCAAGCCTTCTGCACAGTATTGCTGACCCAAAAGACGTCGGACTGGAAAACTCGTATCAGTTTTTCGACGACGGTCTTCTCGTAGTAGAAGACGGCCACGTCGTCGATATCGGTGAGACAGAAGAGGTGCTTAAAAGGCAACCAAAGAACCTCAACATCACCGAATATGAAGACAAGCTGATCACATCAGGTTTTATTGACACCCATATTCATTATCCGCAAACAGGCATGATCGCCTCCTACGGTGAGCAACTGCTCGACTGGTTAGAAAACTACTCCTTTCCAGAAGAAAGGCGTTTCAAAAATCCGGTTTACGCGCATAAAGTCGCAAAACTGTTCTTCAATGAGCTAGCGAGCAACGGTACCACCACAGCATTGGTGTTTGGCACAGTACATAAAGAGTCGGTCGACGTGTTCTTTGAAGAGGCAGAGCGCCGCAACTTGCGTATGATCGCAGGTAAAGTCCTAATGGACCGCAACGCACCAGACTACTTAACCGACACACCAGAATCCGGCTATGAAGCGTCTAAAGAGCTGATTGAAAAATGGCATAACAAAGGTCGACTGCTTTATGCGGTTACACCTCGTTTCGCTCCAACCAGCACACCGGAACAGCTGTCGACTGTAGGTAAACTTCTGGAAGAATATCCGGATGTGTACATGCATACCCACCTTTCAGAGAACAAGAAAGAGATTGAATGGGTACTTGAGCTGTTCCCAGAGCGCGACTCTTATCTGGATGTCTACGACCATTACGGACTGCTGCATAAGCGCTCTGTGTTTGCTCACGGCATTCATCTCTCTGATTGTGAATGCCAGCGTTTGGCAGAAACTGAATCGGCAATTGCCTTCTGTCCAACATCGAATCTTTTCCTCGGTTCTGGTCTCTTCAAACTACCTAAACTGGAAGAACATGGGGTTCGCGTCGGTATGGGCACCGATGTCGGCGCCGGCACCAGCTTCTCGATTCTTGAGACAATGAGTGAAGCGTATAAGATCATGCAGCTTCAACAGGAGAAACTGCACCCATTGAAGTCCCTGTTCTTAGCAACCCTGGGCGGCGCACGTTCACTCCACCTTGAAGACAAAATTGGTAACCTTGAAGTGGGTAAAGAAGCGGACTTTGTCGTGCTTGATTTACACGCAACTCAACTGATGCGTTTCCGCATGGAACAGGCCAAAACCTTGGAAGAGAAGCTGTTTGTGCTGATGAGCCTTGGCGATGACCGCACCGTTTCTAAGACGTACATCTATGGCGAGAAAGCCTATGACGCAGAAGGACGTATCGACGCAAAAAAGTTTGCTAGTTAACTCGGTCTTTTAGTGAAAATGCCCTTGCTTCGCAAGGGCGTTTTTTGTTTCTCGTATCTTATTGAATATCAAAATCAATCTCAAAATAATAACGTCTGCAATAGTCAAAATAAACACATATAAAACAGCGACTTAAATACAAAAAATATCGGCACAATAACTGCTAAGTTTCATCATAACAATGGAGGTGTTGAATATGAGTGAACAAGCTAAGTATCTTGGCCAGAATAAGATTGTTTTATCCAAGGGAGACATTATCGACAAGGAGCTGTTTCACCTTGTGTCAGATATTATTGAACGCTACATCCCAAACCCTAACCAAGCACATTTTCTCGATGTCGGTGGAGGCAACGGTAGCTACGCAGACAAACTCATCGGCCGATATCCAGGCTGCCAGGTCACTATCGTTGACCCCGACGAGAATGCATTGAACGAGAACAACCCCAATAGTCGCAAAAACCTGATCCAAGCCCATTACCAAAGCTTCGACTGCTTCTTCAAATACGACTTAATCCAGTTCAACTGGGTACTCAACCATTTTGTCGCGCCAACCTACTCCAAGACGCAGGATTTACAGCTGAAGGGGTTAAAAGATGCTTACGACATGCTAGCACCAGAGGGCATCGTTGTGATTTTTGAGAATCTGTATGAAGGCACGGACCACTCCAATATTCCAGGCAAAAGAATTTATCAGCTCACCTCCTCGACTCTGATTAAAACGCTTGTAGCCAAGTTAGGTTCAAATAATGCCGGAGTTGGAGTGTGTTTTAATTCAGAACTTTTCTGGCGAGAACAACTGATCAAAGCAGGCTTTAGCAGCGTTTTCTCAGCCCACTGCTACGACTTTGGTAACTTAAATCCCATCAAAAAATGGCTGCTAGGTATCGAGAACCAAAGAGTGGGAATGATCGTGGGTGTGAAATCTGATTAGAACTACAAACTCATGCGTGTTTCTCTAACATCCCTTTCAACCACTCTAACTTAAATGGTTTAGGAAGCACGTTATCAAAGCCTTGCTCTAGGTAGTTCTGTACATCACTTTCAAACGTGTTGGCGGTCAGTGCAATGATTGCGGTATCTGGTGCAAGGTTGTCACGCTTTATCTGTTTGAGTGCTTCTAACCCATCCATGACCGGCATCTGGATATCCATAAAGATAATATCGAAATGCTCATGATTAAGTAAGTCGACACATTCCTGCCCGTTGTTCACGGTGGTGACCTCTGCCTCCAAGGTTGCCAGCATTTTCTGAGCGACGATTTGGTTCATACGAATGTCTTCAACCAACATAACGGAGACCCCAGAAACGTCGTCCCCACCCACAGTGCGCTCTTGTTTTGAATCCGTTTTAGCCATCGCTTGTAAACAATCGAGCAAACTAAATAAGTCGTACGGCTTACTCAATAATGTCATGTTTTCGCTCGGCGGAAGTGTCGTATTGATATTCCCATACACAATGAGTGTTTGGTCATCACGGC

>NODE_20_length_4149_cov_2.328094

GTGCTAGAGCGATAGGGCATATCTTTGAATACTGTTGGCGTATTGCGAAAAGTGATAGCAAGTAGGGTGTCTAGAGAGTGTTTATCGCGGCGATAGCCGTAGTTTAGCTGAATACCTTGTTCGAATTCTGAAGAACTGAAAATGCCATGTTTGGTGTCGTTGTAGTAAAGAGATGCTCCGACTGAAAGCCCGTGATGCTTTTTATTGATAAATTGGTATTGCATGTAACTGGTGATCGCGTGACTTAGGCTTTCTCCACGGAAGTCTTCCAACTCTATGCCATAGTCAGGCATATTTATCACAAAGCGATCGTTGTCCACATCCTCTCGGCCATTTTGGTCAATGCCAACCCAATCGTGAAACGCAATGGTTAATCGATCTAGGTGGTTATTACCAGCGTAATTCCAGCGATAGCTAAGCTCTAGTTGCCATTCTGAAGAGAATTGCCATTTCCCACCAACGGCGATTTGATTTTGGTAGTAGTCTAACTCGTAGTCTGGAGTGACGGCCCAAATACTTGAAATGGTACCCGTACCATAGACCTCGATATCGTCTTCATCCATTGAGAATCCAGAGCGGAGCTGAGGTGACAAACTGTTGGTATGGAAGGGCGATTGTGTATAGCTTTGTACAGGTCCGTAGTCGATTTGTGCCCACGCAGCGTTAACGATTACCAGAGGAATGAGAGAGAGGAAAAACTGCCTGCAAAGGATCATAGATTCATTTCTTATTCTTATGTCACTGATAAAATGATAGTTTATGATTTTATCGCGTGCCGAATTTATGATCGGATATGTGCTGAGGGACGTCTGGCTAACCCTCAGCAGTCGGGTTGGGTAAGCTTTACAGTGGATACACTTTTTCGAATCGTTCTAGTACCAATTCGGACTGTAAATCGAATTTGGCGCCAATCTCACCAGCGTACTGAGTAAAGAATTTAATGTGCGCCTCACGCCACCATTCATAGCTCAGGTCACCTTCTCCTTCTGAGCGGGCAAACTCTTCAGTTACTTGATCGAAAGGGCAGATAGAAACTTCTGTTAGGCGAATGATACAAACCGGCTCTTGCTGCCAGTTAAGCACCACTGTAATGCGACCTACCTGAGGAAGAGGCTCTTGCTCAATGTCGTAGCCTTGTTTAAGGCTGCAAGACGCTCTCTTTGTACCTTGATTAATCAACCTTGCACATTCGTTGGCATTAAACTCGTCAGCACAAAAATATTCAGCGATGGTGTCTGGTATTGATTCCCGCTGTTGAGGTGTGAGTGTGGTGAGGTATTCGTTTAGATACGCTTGCTGCTGTGGGGTCATCATCTTCCTTGAAAAACAACCCCTTCATGAGAAGGGGCTGTTAGCATTTAGTTAGGCCTAGGCGGCACTTAGATGGGTAGTAATATAAGGCTGCCATGCTTCTTGGTAAAGCGCTTTCGACTGAGCTCGCATTGACTTAATCTGGGCGAATTCAAACTCACTGATATCTCGGCTTTCTGAAGCCGCTTGACGTTCGATTTTCTGAATACGTTCGTACAAGTCATGCTCTGGACCACTTTTTACATCAGCAATGGCTTTAAGGTGTAGCTGGACTTCAGCAATCAAGTTGGTCTTTGGAAGCTGGACCAATAGATTTAGGTCACGATAGCCTGAAGCCCCAGGTTTCTTAAAGCGGTTTTTTACTTTTACCACAGTGGTTTCACGATTCAACACTTCGTAAGCTGACATCAAGCCTTCGATGTCATCAGCAACAATCGTCGCACGGGCAAGGTCAGTAATACGCTCAGCATCACCATCAAGTTCATAGGCGATCTTTTGCTTTGCTCGCTCTGCAGATTTCACACCTGCAAAGTGGGCGTCTGTTGAAGTTAGCATTGCAGTGCTCTTACAGATCATTTCCAGTTCAGCCTGAGCTTGGTGTGCTTTGCTGTATAGAATGTCGAAGTCAGAGTAGGGTTGAAGTGGAGTTGTTGGTGTGGATTGGATGCCATACAGGCCACTCAGGTTGTGTTTGAATGCTTTGGAACAGACTTCGTTCTGGTTAGTCGAACGATTTTCCTCACTACCGGGTGCTGTTGGAATAGCGGCGAAAGCTGGCGCGCGGCTGAGCATTAAAAGCATCAGGGCCGTAGTTCGCAAAAATAGGCTCATTCACTCTCCTTAGATTCGTAGCGTCTTGTGTAAACGGTGTAAGAAAAGGCTCAACAACCAAAACGTACCAATCTCTAATATCTATATGGGGGCTAGCTGGCGAATTACAATGTAGGAATTACCCTATGGTGTAATTGTTTGAGCTAACTCTCAATTTGTTTGAGTGCCCGATTTCCTTATGGTTCATTCTCAAGTTGTAAATAAGTATAAACACCTTACCTGAACCTAATCTGAACGGTTTATACAGCTTGAGGGATACCACTAAGAAACGCTTTCGTATAGGCTATGAAGCAGTCACGTCAGGTAGGGAATTGAAATGAAAGATCCAGAATTTTGGCACAGTAAATGGGCATCAAACCAAATTGGTTTTCACTTAGAAGACGTAAACCCGCTGCTGGTTGAGTATTGGTCCGAAACCAAGCCAAGTAGAGAAGATAAAGTGTTTGTCCCACTATGTGGCAAAACAGAAGATCTTGTTTGGCTAGCGACCAAACACGAGGACGTTCAGGGTGTCGAACTAAGCAAAATCGCGGTGCGCTCATTTTTTTCTGAACATTTTTATACGCCTACGGTGATCCCTGTAAACGGTCAGCATGAATTGTATCAGTTTGATGAGCTATCTATTTATACTGGCGACATCTTTACCGCGCCCGTTCAACCAGTAGAAATTGTGTATGACCGTGCCGCTCTGGTCGCTTTACCGGAAGAAATGCGCGCAGATTACGTCCAACGCGTTAAATCGCTGCTGAAGCCGGGCGGAAGAATTCTGTTAGTTACGTTAGATTATCCCCAAGCTGAAATGGCTGGTCCTCCGTTTTCAGTCACCGAAAGTGAAGTTCGCCAGCTGTTCTCAGAATATAAAATTTCAAAACTGTATCGTGATGACGCAGATGAAAATCATCCTAAGATAGCGAAGAAAGGTTTATCCAGCTTCGCCGAAGAAGTGTATTTGATTGAAAGTTAATGATGAAAGCCCCTGAATAAGCTCAGGGGCTTTTGTTTTAGTAAACGATTTTTATCTTATCTGCGCTGGACACGGCGCTTTCTATCGCTTGCTCAATGGTCTCTCTACGAGCAATGCTCACTCCTAGGCGTCTGCGTCCATTGATATCAGGTTTGCCAAATAGACGTAGCTGAGTTTGTGGGGCTGCTAGAGCGTCTGTCATTCCCTCAAACTGAAGGTTATCGGATTGGCCTTGCCCTAAAATCACAGCTGAAGCGGTAGGTCCATATTGGGTGATACCTGAAATAGGGAGGCCAGTGAATGCGCGTACATGAAGTGCGAATTCAGAACTGTCTTGAGACATGAGAGTGACCAATCCGGTGTCGTGCGGGCGAGGGGAAACTTCATTGAAAATAACGGTGTTGCCCTTAACGAATAGTTCTACGCCGAAGATACCACGGCCTCCTAATGCATTTACAACCTTGCCGGCGGCGTCTTGGGCGGCAGATAGCGCCGCATCGGACATCGCTTGAGGTTGCCATGACTCGCGATAATCACCGTCTTCTTGGCGATGTCCAATCGGAGCGCAGAAGTGGATACCATCCACAGCTTTGACTGTCAGTAAGGTAATTTCGTAATCAAAATCGATAAAACCTTCAACAATGACGCGTCCAGCACCAGTGCGGCCACCTTCTTGGGCGTACTGCCACGCTGTTTCTACATCTTCTGCCGATTTAATAACACTTTGACCTTTCCCAGAAGAGCTCATAATTGGTTTACATACGCACGGGATGCCAACGGATTCAATGGCTTGAGTGAACTCTTCATAGGTGCTTGCAAAATGGTAGGGTGAAGTGGTGAGGCCAAGCTCTTCTGCAGCCAACCGGCGAATGCCTTCGCGATTCATTGTTAGGCGAGTTGCATTGGCTGTTGGCACAACATTGAGCCCATTAGCTTCAAGCTCAACAAGCTTGTCTGTGGCGATGGCTTCGATTTCAGGCACTACGTAATTAGGTTGTTCTTTGGCGATGATCTGCTCTAGTGCTTCACCATCAAGCATATCC

>NODE_21_length_4137_cov_2.442365

GTCTACCACATCGCCAACAGGGGTAACATCAATGGTGAATTGCCCTTGATGTGCAGTAGGCTCTTGTAACAGTTGCTCTTGCGTAAACACAGTAATTCCAATATTGGCAGTGCCACTGAAATGCTCTGCTGGGCGAATCGAAATCGCTGACAGATCGATTTCTGTTACGCTCGGGTCAGTCAATTGAATAACCCATTCACCACCACCACTGTTCTTAACTACAAAGTCAGACGAAGTACTGGTTACCGTAAAGTCATCAGGCACACCCGTTAGTTTGGCTGAAAGGAATTGCTCTGAGCCATCCGTGTCATTCAATGCGATCGACAATCCAGAACCACCTGAAGTGAGCGAAATGTCGGTATCTTCATCACCAACCACAACGATATTGTCCGCTGCGGTTGGCATAGTGACAGGATCCAGGACTGGCGTGATATCAATGTCCACGTTCTGCGTGAAGGTGCGCTCACTACTCGTATTGGAAACTTGCGTCGTACCTGTGGTATCAAAAGTGGTTTGGTCAACCACCGTGCCTTTCACTGTAAAGGAGATTGAATTACCACTGCTATCCGTAGGATAGTTTTCTCTAGGTACAAAGTACAACCCATCCAATGCAGCCTGAATAACCGCAGGATCGTTAGAATCAACGACAAACACACTGCTGTCTGGTTGGATCGGGTCACCATTCACATCAGCGAAATAACCCAAGCTAGAGTCAGGTAACGTGATTTCAACTCTCGTCAGTGTTTCTTGGCCTTGGTGACTATCGTTGCGAACATCTGCCAAATCAATATCAAGATCTAACTTGATAAGATTATCTTCCAGTGCATTAGCGTCCAGCTGAGTACCCGTGCCTTTTTCAACTGAAGTCGCACTCAGGCTTATTTCAGGAGTGACATCGCTGTCGAGAGGCTGACCCTGCTCGCCCGCTCCCGGCGCTATATCTACTACCGGTGTCACAGCGACAGGGATATTAAGCTCAACACGATTCTCATCACCTGATGCTTTATCCGTGGTGACAAAGGTAATAGGCAAATTAAAGTCACCTGCATAATCTTCAGGCAGTTCTAGTTTGAGGCCATCAGGAATAGAAATACTGCCATCAGGGTTGATAGTCGCTTCAAATAGATAAAGATTATTGGCAAAATCGTACTCAGCCCCAATGACCTTCAGACCACTTACTTCTGCCGGAATATCATTCGGGTCAATCACAATGGTGAACTGATCCGCGACATGATCCGCAGTGCTCACATTGCTACCATCGACACCAATTTGGACAACGTTGCTTATCTGACTACTAAGGTCAACGTAGTTGTCTTCAACTCCTGTGATAATGGCGTCTGGAGTTGTCACTTGCTCTATAACCGCAGCTTCGCTGTTTTGCTCAATAACATCAGTCGGGAAAGTAAGATCCACAGTGGTCGATTTCTGACTCACTGGGCCATTCTCGTTTTCATCACCTTTATCGTAAACCTGAGCGACAAACTCAACGGTCAAGGTACTGCTCGGTAACCCATTACTATCGGTGTAATTTAGGCCATCAGGGGCGACAATGCTGAAGTTTTCTTCGTCAGTGACTGTCCAAGAGCCATCACCATTATTCACAGCACCTGTGACGAACAGTTGATCCAAATCATCGCCTGAAATACCGACAAAACGAACCACCACTTCTTTGACTAGTTCATCCGAACTCGGATCAAGATCTTCAAAGACAATAATATTCGCATCACCATTTTGATCGTTGATAGTGAAGTCTATACGACCATCATCATCTGCATCGTCAATCGTGGTAATAACAGCATTATTAGATTTAACCTGAAGATCACCATCACTCTCAACAATTGGCCTAACCACAACGGTGAGTGAACCCGTCACTTCTTTAGGGGCAACACTGTCCCCATTATTATCGGCGTCATGCTCTTCGATGACCAATTTGCTCTGTAAGTTCATGTCAACGCTAGAGTCTTCTGGCGGCTTAACACGCACGACAGGTTGGCTGGAGTTTTCGTCCAACCCAGTGATCGTCAATGTATTGGTAACGTCATCATAAATCGCGGTGCCGCCTGTGTAGTTAGCCACTGGAACGCCATCGATCGTAATCTCAACATCTTCAGGGAAACCTGAAATAGTAATGGATGAGACGAACTCATAATCACGATCATTGGTATCTGGTGCTCTGTCAGGATTCTCTGCATTTTGCAGATTCCAAGGGACGACGATAAAGCTGTCTTCGTTACCTGTAGAAGTCCTACTGTAAGCGTCATCACTACCACCGGCATCAATAACAGGAACAACTTTAACCAGAATACTGCCGTCAACTTCTCTGATGTGGCCGTCATTCTCCGTAACGATAACTTTCGTCGTTATGTTAATGTCTTCCGTCGAATGTTTTGGCGGTTCAACCGTAATACCTGAACTGTATTGCTCTTGAGTAATATTGGCCTGATAAATAGGGCCATTAGCATCACTACCTACATATACCAAGTCAATTGCACCACCATTACTGTCGTACAATTGAACGCCATCAGGAATATCCGAGAGAAGTACCGTTATCGATTCAGAGTTATCATTGACACCATCTTTTTCTTCACCCGACAGGACGGCAAAACTCAGCTCAATACTGGAGTTTTCATTAATGACAACCTGCGCACCTGCATTGCCATTACCGTCTGTGTAGCTGTACCAAGTATCAAGCTCAGCACCAGTGTCAGGAACATCTGGTACATCGGGAATGTAAGGGATGTCAGCGACACCTTTCACCGACACATGAACGGTTTTACCTGCACCCATTTCGACCACATCAGTGACCTGAGAACCGTCGCCAAGCGTTGCGGTATCTTTCACCACACCTGTCGCCTCAAAGGTAAAGTTCTCGTTGGAATGCAGAGGTGGCTGAATCTCGACTTTGTCTAAGTCAGATTGTGCAATCTCATAGTAGCTACCACTAGCATCAGTAATCAGGGGTAGTGGATCACCTGAACCCACCCAACGCACTTCAAAACCAGTTAGCGGCTGACCATTTTCGTCAGTGAAATCAGATAAACGGACATACAGGACTTCAGAAGTATCTCCCGGAGTATCGATGTCAGCCGTTGAATTGAGCGTGATGACTTTATCGAGTGTAAACGGTTCATGATCGGTATCGGGATCGACTGTGTTCTGACTCGCTGCGTTATCCTCAAAAATGGAAATACGATTGACCGAGAAACTACTGTCATCCGCTACTGGCGTGACATTGAATACCAGCTCTTGCACAACGGACTCGGCAGTTTCTTTGCCAGAAACAGCATTGGAGCTTTCTTCGGTCAATGCTTTTGCGTCAAACTTAATCGCTCCAGAGAAGTTATCCCTCGGATTTAGCTGAACACTGTTGATGTCATCAGAGCTGATCAGATAAACGCCTGGAGATGACTCTGCAATGACGTTTCCTGATGCATCCAGTAACTCAAACTCGCCTTGACCTTGAGTCACTTCAAGACGGTACGTAATCGTCTCTGGATTACTGGTATCTTGTGTCACCGCACTCAAGTTGAGTGTCTGATGAGCACCATCCTCTGTTAGCGTGTATTCGTAGGTGCTATTGGCATCATCCCAGGTGGCAATATCGGCGACACTTTCCACTTCAATTCTGAAGCTCGAATTCAGCTTGTGATCCAACGTACCATCGTTGTCAATTTCAAGCTGGTACTTGATCTGGATACCTGATTCATCGGTCGAGTAGTTTCTGTCCGGAACAAAATACAGGTTGGTGATCGTGGTGATTTGATCGCCATCACCATTCACACTCGATGTCTGATCAATCAAAGAGCCATCAATGTAGATTTTGCCTCTGTTGTTTGGCTCAAGTTCGACGTATTGGCTGCCGTCAAAATAATAGAAGGTACCGTGGTGATCTCCCGCCTTGATCGTCATGCCGCCGATAGATTCGTTGTTATCCGCATCATACAAATTCGCTTGAAGCAAAATCTGTGTTGGAGAGTCAAGACCAGTGATACCGCTCTGGTTATCCTGAACGTTAGCGGTATCAACGGTATTCGGATTATTTGGATCGTAGTCAATCGACGGATCACGACCAGATTCTTCGAAAGTCGTAACTTTGAGAGCGATTGCTGACGCTTTCCTATCGACGATGGTCAGATC

>NODE_22_length_4108_cov_2.453734

TGACTGGAATCTGGCGACGTTTTAGCGTACCCAGTTCCTGAACGTTCATCATGAAAGAGCCGTCACCTGTAATAAGGATAGACTGATCATCCGGACGTGCGACCGCGGCACCCATAGCGGCAGGAAGGCCAAAGCCCATGGTGCCTAAGCCTGCGGATGTGATGAAGTTTTGTGGGTCACGTGGCTGAATATGCTGCGCTGCCCACATCTGGTGCTGACCTACATCGGTTGAGACCATTGAACTATCAGGCATCATGTCGGACAACTGTTTGAGCATACCCGGAGCAAAAATCAGATCACCCGGGTGGTCGTAACGCCACTTAAAGCCACTACGCAGACTTTCACTGTGCTTGACCCATGGCGTGATGTCTTGAGTCAGCTCGAGTTGAGGAAGAAGGGAATTGATATCACCGCGCAGTGGCGCATGGGCGTGACGCAGCTTGTTGATCTCAGCGGCATCGATGTCGATATGGATAACGCGAGCGTGAGGGGCGAACGTTTCCAATTTACCTGTTACTCGGTCATCGAATCGCGCGCCAACAGCAATCAACAGGTCACATTCTTGAACGACTAGGTTCGCGGCTTTGGTGCCGTGCATACCCAGCATGCCAAGGTAATGTGGGTAGTGACGTTCGATAGTACCTAAGCCTTTCAGGGTGCTGACCGAAGGCATTGGGTTTAAACGTAAGAATTCGCGAACGGCTTTTGTGGCTTTACCCAGTTGTACACCGCCACCAACATAAAATACTGGGCGGGTACTTTGTGAAAGAAGCTCCTGAGCGGCAGAAACGGCTTCTGCTTGAGGAACGGGAAGCGCTGGTGGTGTGAATGGAGGCAAAACGTCAACCGGTGCCTGAGCCAGCTGAACATCTTTAGCGATGTCGACAATCACTGGTCCTGGTCGGCCTGCTTTAGCGACTTCAAACGCTTCGGCTAAAGTTGGTGCAAGCTCTTCAATCTCAGTCACGAGGTAGCTGTGCTTGGTACAGGATAGTGACATTCCTATCACATCCATTTCCTGGAAAGCGTCTGTCCCGATGTGGGAACTGGCGACCTGACCCGTGATTGCCACCATTGGGATCGAATCCATAAACGCATCGGCCAAACCGGTTACTAGGTTAGTGGCACCGGGACCAGATGTTGCCATACAGACTGCGACATCTTGAGTCGAGCGTGCCATACCGATAGCGGCCATCGCTGCACCTTGTTCGTGACGGCAGAGGATGTGCTCGACGCCACCGTCATACAGCGCGTCATAAATTGGCATGATGGCACCGCCTGGATAGCCGAATACGGTTTTGATGCCCTGTTGTTTTAATGCGGCTACGACTAATTCAGCACCTGTCATCGTAAGCCTCCTTGACTACCTAAGTAGTAATGTTGTGTTGTTGTGCACGTCATGTGCGCTCCCATTCTTCCTGCCAGCTCGCGGCTGAGCTCTTTATTTTTCTGTCTGCCTGACTAAGTCAGTGTTTATTTTTTCGCTGATTTTCAGCGTTTAAGTTGAAAAAAACCCCCGGACTTTTCAGTGCGGGGGTTTTTTTGAATTTGGTGTTTTATTTTTCGCCCACCAGCCCCCGCGCGGAATCAATAATGACCACGATAATCAGGACAATCAGTGCGTTAATGCGGGCGTTGAAATTCATATTCTTTTCTAATGTTGTGCTTACGTAATATGGTACGAAATTGTTTGCGTCACTAGTGGTAACATAGAATTCCGTTACATGACAAGCAAAAAGTCATTCTTTTTTCACATTCTCTCATCATCTCACCACGCTATATAACAAGATTGTTAGAAAAGTGCGCAAGTTATCAAACAGATAAATAGATAGCAGAGAGTGTAGGAAAAATTATGGGCTTAGCCATTATTTACAGTCGGGCATCGGTGGGGGTTGAAGCGCCACCTGTGACGGTAGAGGTGCATATCAGTAATGGAATGCCGGGGTTCACTCTGGTGGGATTACCGGAAACCACGGTCAAAGAATCACGCGACCGAGTGCGCAGTGCGATCATCAACTCTCGATTTGAGTTCCCAGCCAAGCGAATCACGGTCAATCTTGCACCCGCCGATCTGCCCAAGGAGGGAGGGCGCTTTGATCTGCCGATTGCACTAGGTATTCTCGCCGCTTCAGAGCAGATCCCAGTCAGCGGCTTGGCAGAAAAAGAGTTCGTTGGTGAACTGGCTTTGTCGGGGAAACTGCGAGGAGTGAAAGGAGTATTGCCTGCAGCGCTGGCAGCCAACAAAGCACAGCGAAGCTTGGTTGTACCTCATTACAACGGAGATCAAGCCGCGTTAGTGGGTGAAGAACAGCATAAATCTGCCCAAAGCTTACTAGAAGTGTGCGCTGATCTTTGTGGCCAACAAAAACTTAGCCTGCATCAAACACAAAATCTGCACCGAGCGGTGGATAATGGCCGCGACTTACAAGACATCATTGGTCAGCAGCAAGGTAAACGTGCTTTGGAAATTGCTGCGGCAGGCAACCATAACTTGTTATTTTTAGGCCCGCCTGGGACGGGAAAAACCATGCTTGCCTCGCGTTTGTGCGATTTGCTGCCGGAGATGAGTGACGATGAAGCAATGGAAACGGCGTCTGTTGCGTCACTAACTCAGCAGGAAATCAATGCGCTTAACTGGAAGCGGCGCCCCTTTCGTGCACCGCACCATTCAAGCTCAATGGCAGCGCTCGTCGGCGGAGGCACAATCCCAAGGCCGGGTGAAATTTCTTTGGCGCACAATGGGTTGCTGTTTCTTGATGAAATGCCAGAGTTTGAACGTAAGGTGCTTGACTCTTTGCGTGAGCCACTGGAATCCGGGGAGATTATTATCTCGCGTGCTCAGGGCAAAACTCGTTTCCCTGCACGCTTTCAGCTGGTGGGCGCCCTTAACCCGAGTCCGACGGGGTATTACGAAGGTAATCAGTCGAGAGTTAATCCACAAACCATTTTACGTTATCTGAGTCGGTTATCTGGCCCTCTACTGGATCGTTTTGATATGTCACTTGAGATCCCAGCGTTACCTAAAGGTACTTTGGCCGAAGGGGGCGATCGTGGTGAACCTACAGAAGTAGTTAAGCAAAGGGTTCTCAATGCCAGAGTCTGTATGCTGGAGCGCTCCAATAAGGTTAATGCACTGTTGGGCAGTCGCGAAATAGAGGAGTACTGTCCATTGAAAAAAGAAGATGCGCAGTTTTTGGAGAGTGCGCTGCACCGACTTGGGTTGTCAATTCGGGCTTATCACCGAATCATTAAGGTCGCGCGCACTATTGCTGATTTAGATGGTCAGTCGCAGATTGCTCGCAATCACCTGGCTGAAGCGTTGGGGTATCGGGCAATGGACCGATTGCTTAAGCAATTGACCGCTCAGGCGGTGTAAAAAAGCCCGCACTGGGCGGGCTGTACTTCAACTTAGAATTGGTAGTTCATACCGACCGAGAGCAAATACTGGTCTTCATCGTAGAAGTCGATGTTGGAGTCGGTTTTGGAGTAGCCAGCAAATGAAACCAGCGACCAGTCTTGCCAACCCATAAACTCTTGATATTCATACGCGGCAAAGAAGCTCAGTTCATCTTCGTCACGTGTCTTACCATAAATTGCATTGATGGCGTCGTAAGAACGGTTAGTATACCCAGCGGTCAGTGCGAACTGGTGGCGGTTCATGATCTTAAAGTAACTTAGCTCACCGCCAACTGAATCGAGTGAGTTGGCATCTCCGTCGGCATCAGTTTTGATGTAGGTGAGTGAAGGCTGCAAGAACGAAGTGCGAGTCAACGGTAGACGATAGTCACCTTTTAGATAGATCGATGAAGAGTCACGCTGAAGAGCATTTCGCTCTGCGGTTGTTAATGATGTATCACTGACACCAGATTTTTCATCGTCAATATCGCGGGTTGCATACGCGGTGTCTAGGCTGAAGTTAGAGCCCATAATATTGTTGAACTTCAGACGGAATGCATTGCCTGTTTCGTCTGTGGTTTGACGCTCTTGGCCCGTCAGGAATGGGTCAGACCAAGTTTCGCCCGACATCACTGTCGGTAGGAAAGAGGCATCAATCACCATACCTGACGCAAGTTGTTGCTTATATCCTAGCTCCAGCGCTAATGTACCG

>NODE_23_length_4047_cov_2.689421

GCAAATTCTACCCGTATGAACGCGGAAGTGAAAGCAGGACGCAGAGCACCTCACATAAACTACTTCTTAGCACTAAAAAGAAGCGCCAGTCTTTCGACCAGCGCTTTTAAAATCCTAATACCTAAATAGTTGGTGTTGCGCAGTTAATAACTGCTACAGCGCCAAATCTGACCGTATTTACATTGTGTAAGTGTATGGTGCTTGAATACCTAGCGGAATGCCTAACATCCAGTAAGCCACTAGGAAGATTGACCAACCGATTAGCATAGCAATCGAGAATGGCATCATTAATGACGCTAGCGTACCAATACCAGTCGACTTCACGTAGCGCTGGCAGTAAACAACCACTAGAGGGAAGAATACCATCAGAGGTGAGATGATGTTCGAAACAGAGTCACCCACACGGTATGCCGCTTGTGATAGTTCTGGAGAAATACCAACAGCCATTAGCATAGGCACCAGAATTGGACCAATCAGTGCCCATTTCGCTGATGCAGAACCGATTAGAAGGTTTACAGAAGCCGTTAGAAGAATCATACCAATGATTGTTGCTTCACCTGGTAGGTTCATCGCTTTCAGACCTTCTGCACCGTATAGCGCTAGCATAGTACCGATGTTTGATTGCGCGAATGCAGATAGGAATTGTGCACAGAAGAACGACATTACGATGTATGCGCCCATCGTAGACATGGTGTCCGCCATCGCTTTGATGATGTCATTACTTGTCTTGAACGTGCCAGAAACGCGACCGTAAACGTAACCCGGAATGATAAACAGGATGAAGATCAACGGAACGATAGACTTCATGATTGGCGCAGAGAACGCTGTGATCTCACCTTCCGGTGAACGCAGTGCAGAATTTTCTGGGATAAGCGCCACAACAAGAAGTGCGATGCCCGCCATCATAGCCCAACCCGCGTAACGAAACGCTTTAGACTCAAGCTCAGTGAAAGAACCTAGATCTGGTGCTTTCTCTGCGTCTTCGTCGATTGGAGTGTTCTTTAGACGTGGCTCGATGATTCTTTCAGTTACATACCAACCGATAGCCACAATGATTACTGAAGATAGACCAGTAAAAAAGATATTCGCTAGAGGGTTAACCACGTACTCAGGATCGAGAACCTGCGCGGCTGTTTGAGTAAAGCCAGCCAATAGAGGGTCAATACCTGAAGGGATGAAGTTTGCAGAGAAACCACCTGATACACCTGCGAACGCTGCCGCGATACCCGCTAGAGGGTGACGACCTGCAGCGTGGAAGATGATACCACCAAGAGGAATAACCAGAACGTAGCCCGCATCAGCAGCAGTGTGCGATACGATTGCTACAAGAATTAGCATTGGAGTAAGAAGTTTTGCTGGCGTGAAGTTCAGCATCTTCTTAAGGCCAGTGGTGATGAAGCCAGAAGAATCGGCAACACCTACACCCAACATCGCTACTAGAACGATACCAAGTGGTGCAAAGCCAGTGAACGTTGTCACCATGTTAGCAAGGAAACTTGCTAGTGCTTCACCTGTCAGAAGGTTAGTGATGGTAAGAGCTTCGCCAGTTCGAGGGTTTAGAAGGTCGAAAGAAACATTCGACAAAAGTGCAGACGCAGCCCAAGTAATGATCAGTGCCCAGAAGAACAGAATGGCCGGATCCGGGATTTTATTACCAGCTCGTTCTATAAAATTAAGAAAGCGATCCATGCCATTCGGCTTGGACGATGGTGCTTTATTTACTGCTTGGTTACTCATGGTAACTCCATATGTGATGTTGTTGCCTTAGGCCTGTAATAATTGTTGTGACGACCTATTTTTCAGCTACACATTTTATTAAATTGCCCCCTCAAAAGCACAAGATTCCAGAGGTTTTTCCATATTTGGAGACATATTTTGCAAAATTGCCATTACTACGCAACAAAGTAAAAACACTAGCCACCCATATAATGAAACAGATTTGAAACCATTTTGAAAAATATAGAGAACGCTTTTAAGTAAAATATCAATAATTTATTTTGAATATAAAATAGTCTACATACTAAATTCATTGAATTTAGAAACTGATGTCATTAGCTCAATGGATTGATGTTTTCCATGACGATCGAGCCAACACACCTCCACGGTAATGTACTTAACTTCAACCCCATTTTGTAACGAGGTGCGAGCCTCGCATCGTATCTGGAAATATTCAGAGGTTGACGGCTGACACTGAGTTTGCGAATAACAGGCTTCTTGTTCTATATCGCCGAATTGATAACTTTCCTCCTTCGCCGTTGATCCATGCGTGGTGAACTGCTCAAGCTGAGATTCTGCCAAATAGAGCGCTTCAATAGCCCGTTCAGCCCAATCGGATTTCATTTCGATATAGGTATGTAATTTGACTAAACCGACAGCACTACCGCTGACAAGCAAGAGAGCCAATAGCACCTCAAACAAGCTGACACCTTTCTGGTTAGAAATCATGCCAACTACCATTGCTCCATTTGACCTTATGAAAATGTCGAGCAGCCGAGGCCGTGACATCTTTATTGAACTGCATGTATAGATCATTGTTGATCAAGGCATAGCTATCAGGAGCATCCAACACTAAACCACCTAACGGATAAACATTACCACTCTGAAAATAACTCACTTTTTGTTGCTCTACTGTGTTCAGCAAAGCATGCTCGTTCAAAGATGCAATCAGTGCCGAAAGCAATGGATTGTTGTTTGATTGTCCCCAATCAACAAACCCAGCAGACGGACTCTCAGAAACAAAGTGATAAATCATGGCCTTCAATGCTTGGTTTCCTGAAATAGAAGCTATGCCATTGTGGAGCACCAGTATCAGACCACCATTCAAATGAGTATCAATGGCAAGGTTTATGGATTGCAGGTCATCTGCGTTAAGTTCACAGCTACCGTATACCCAAATAAGCTCATGATATGCTTTGATAAGTTCTGTAATCTGCTGGCCGCAATTTTCTATGTACTGCGCACTGGGTAACTCACTCGGTAAACGTGGCATGACACTTTTAAGCTCTTCTGGTACATAACCGAACAAGCTGGTGTCCGACATGACCTCAAACCACTGGCTACGTGGCACATTAAAAACTTCCTGAAATACATCAAGTTGCTCCACATGCTCGAAGTCTTTGTGAGTTAAAGAGAGCCGCCAACTGAAATCAGTGTAGTGAGTTTGGTTACATTGATGTGAGTTGGTTCCCTGACTCTCAGGAAAATAACGAGAGATATCGGTTGCTTGATACGGATGCGTGGTGTCGTAGCTATGCGCATAGAAAATATTCTGATAGCGAGCCGAGATGCACGCCCAGCGGAACTCGCTAACGCGTTGATTCACGTGCGGCGCTATGTTGAGCCCACCATTGACCAATAAGTGAGAGATTGCTGTGATTGGGCTTTTTATTTGAAGTTCAGGCAGAAGAAAAGCTTTGCTTATGGTTAAGAAGCCACTGGTTGCACGAACAATATGTGGTGTTCCAGTTTCAATCTCAAGCGCATCCAAACTCAGTTGCTGCTGACAGTCACTCAGCTCTTCTGGTATGGCTTGTTTTCCAACAACAGAGGAAATCGCACACTCTAACCCGCCCTCTGCGCGCCAATGTGCATGGCGACTTTCCACTTCATTTTGACTGCGTTTAATTTGGTAGAATAAGGCTTTATAACTGCCCATGACAATCATGAGCACGCCAGCTAGCAAAAGGCTTGTGATTAAAAGGGTTGCAGTACCTCGCTGAAATGTCATGTCACCAGTTCCTCTGTTTAATGGAAAAGGTGAGCGTCTTCGATACCTCTGGGGCAAAGCGTAAATGGGTACTGAGTTGAGCATTGAGTAATGAAGAAGTATGGGTAGCACCGGCTAAAGGCTCGGAATCCAGTTCAAACTCGGTCACAACAATACGATTACTATCGAACACTGTGTTACAGGTATTACCAAAATGTGTGGTGAAGTTTTCTGCTTCTAAAGCAGACATAGCTCTTGGTAGCTTCTTCGCGCAGACTCTCAAAACCTCTTTTGATTCGCTCTGCCGCTGATATACCACATTCGTGTATGCTGTCTCAGAT

>NODE_24_length_4020_cov_3.316510

ATACTTTCAATGATTTTGTAACTTATCAGGTTTTCGTTCCAATCTACATCTGGACAACCTCTGATAATGTCATCTGCTGACTCTTGAATGCGTTTTTCAATGCTATATGGAGTACTTCTAGGATCAAAGTCCTCCCTATCATCGACTTCTCTCTCCAATTCAATATCAAACTCTGAATAATCGATCTTTGCCATCAAGTTGCCTCATTGCAGATTTTCCATTGATACATAACGCCGCATTAAGGGGTGAACAACGCAGCCACCCGACTTAACATATTGTGCCATAAACACTTAAATTGAAGTAGAAGCTGAAATGCCAAGCGTTGAGAATCCCTCTTAAATGCTTGGTTAGGTGTGGAGCCTTTATTTACGCTGGCAAAGCTTGTACTCGTTAGGCATTGTGCGTTCTAGTAAAAGTTTATTAGGATGACCTTTGGGTGCTTTGTGAAGACCATGATATACGATCCACTTGCCTATCTCTCGATGCCTAATTTCATGGCACTTTCTCCAAAAGCTAGGAGTAAGCTCTATCTCAACTAATTCATCATCTATTAGCAACCTTATGTTTGGCTGCGATTGAGAAAAGTTGGAATCTCGATCCACCCGAGCAATACGAACTCCTAAACCTGCACCTGTCTCATAATTGGGCTTACCACTCGTCCAAACTTTGACTTCCATTGTATTGGCCCCTTATGTAAAAACACCTAACGCCGCGTTAAGTGGTGAGCAACGCTACCACCTGACCTAAACCATTGTGCCGTAAACACTAAAGCTGAATCAAACCGAAAATACCAAGCGTTGGGAATCCGTCTTAAACGCTTTGTTAAATTCGTTCTGATGGAGTCCCACAGACTTCAACATGCTTTGGTACCCACCATTCTTTACAAAGACGCGGTTTCAATGAAAAAAAGTAGTCACCATCGACTGGTATTACTTGTTTTTCGCAGTATGTTTTATTTTTCAGAGTTTTATGAAGTTCTTTCCAACACTTTGAGCCTTTCATATTGGTCTGCATCCATTTATCCGAACGAGGCGATATAACTGGAACTTGTATATCTAACGCGCATAGAGAGTATAAAAACTGATCATTTGGTAACTTTATAATTTCTTGCGAATTACTTCTGGGGTTAACAAAATTCCACCCTAGCAGCCAATAAGGAAACGGTTTATCTTTTTTCGACCAAACTTCAGATAAGAAACTAGCCGTCACTTCAATGATCTTTGAGCCAGTTAATGCTTCAAGTGTTTCATATTCATCTGTTTGAGTTAACTTAGGATGTATTAAGCCACAGCGAACATCATAAAAGAGGTTGATGTGCTTCATGACTCCCGGCGAATACTTCAAACTACTATCACTTCCTAAAGAATCTTTAGGCCAATTTTGTAGCTTTTGGCTAAATTTCGTACTCTTTATCAAGCGTAATATTTCGCTATCCTCTGTATGTGACTCACGCAACTCTTCTGTTTTCAAGTGATTGAGGAAAGCTTCAATACACGCAATTGAAAAATACACAGCAGATGTCACATGGTGAAGCATACTTTCTTCATGAGGAGCCCTGTCACTCTCAGTAGCCTCTCTATACATCGAACAATAATTTTTAAATACAGGATCAAGATTCCAAGGATTCATTTAACTCTCCCCGTCCGTAAAAGAATTTAACGCCCAATTAAGGGGTGAACAACGCTACCACCCAACCTAAAAGATTGCGTCATAAACACTAAATTTGAAGTAAAAGCAAAAGTGCCAAGCGTTGTGAATCCCTCTTAAATTGCTTGTTATGACGATTTTCTCGCATTCATAATATTGCCGAGACCAATACTAACAATAGAAATAAATAAGCCACCGAGCATACTTACACCCCATGTTTTCATATCAGATAACTGGGACTCTAGAACTTTTATATGAAGCTCGTACTCTTTGACTTTCTCAACTTTCCCATCGAGCTCGCTGATACTTTGCTCTAGCTTGTTGACCTCTTTACTCAATACACCAAGATCCTTAGCAAGTAATGGCAACTTCAAGACCTTTTCTGGTGTTGCAACTAGTGCCGTTTCTATTAACTCTATACGGTTGTCCATTATATCTAGCTCAAGGTTTTCGGCTATGCTTTCTTCGACTCGCTCCTGTACAACTTCTATTAAATCTGTTTTTGATTCACTATTTAACTTCTGACTAGTGAACAATAAAAGTGTATTTTGCTGTTGCTCTGATAAACTGGCAAGCTGCTTTTCTGATTGATTAATTGTAGAGTCAACTTGCGCTTTAAAATCGACAAAGTCATCAGTTAATTTGCTTAAATCGCCCGCTAGTTCACTATATTGCTGACCATCATTAACTAGTTCAAATTTTACTCTATCTTCCTTAACTTGAGAATCCGTATATAATGTGGCGGAAAACGCTATGATTGCAGTTACCACTGCCGTTGATAGCCCTACGCCAGTAAATAAATTGTCGCTGATTTTAATACTCATATATTAGTTAAATTCCTAAGAAACCTATCGTGCTGATCGTCATAACGCCGCGTTAATGGGCAAGCAACGCTTCCACCAACTTAAGCCATTGTGCCATAAACACAAAAACTAAACTGTGAGCCAAAACTGCCAAGCGTTGGTTGTCCGATTAAACGCTTGGTTATGGGAGTTAGAGACAACCAATACACTCACAACAAAGCGTGAGCTCTTTGCGGCCACGAAACCCCACAGACCAATACGCAGCAACCAGACGAAGCCCAAGAAGAAAACTTACAACAACGATACAGCCAACTAAAACAAAGAAGGGCCGCCCCACTGAAGCCTAGGTTCAGCTAATTGCCGCAACACCCAACCTACTTGGGAGCCGAATTCAAACGGCAGATTGGCCGTCATGGAAAACTCAAAGCGGCTTTTCACCAGAGATAAAGATTTTGAACCTAATCGACATGTAAGAATGACCATTAAGCTCTCAGCCAACGCTGAACCAACAAGCCAAATAAGAACTATATCTTGCTGCGCCATAATGCCGCGTTAATGGGCAAGCAACGTTGCCACAAAACTTAAGCCATTGTGCCATAAACACAAAAACCAAACCGTGAGCCAAAACTGCCAAGCGTTGGTTGTCCGATTAAACGCTTGGTTATGGAAGCCTAAGACAACCAATACAATCACTACAAAGCATGGGCTCATTGTGACCACCAAGGACAACAAACCAGTATGCAGCAACCCAATAAAGCCCAAAGAAGAAACCTAACAACAACGATACAGCCAACTAAAACAAAGAAGGGCCGCTTCACTGAAGCTAAGTACCAGCTAATTGCCGCAACACCAAACCTACTTGGGAGCCGGATTCAAACGGCAGATTAGCCTTCACGAAAGGCTCAAAGCGGCTTTTCACCAGAGATATAACTATGAACCTAGTGGACATGTAAGGGTGGCAATGAAACTCGCAGCGAATGCTGAACCAACAAACCAAACAAGAACTAAATCTTGCTGCACCATAACGCCGCGTTAATGGGCAAACAACGCTGCCACCAACTTAAACTATTATGCCATAAACACAAAAACTAAACTGTGAGCCAAAACTGCCAAGCGTTGGTTGTCCGATTAAACGCTTGGTTATGGAAACCTGAGACAACCACTACACTCACCGTACAATACGAGCGCTTTGCAACCACAAAACACAACAAACTTGTATGCAGCAACCCAATGAGTCCAAAGAAGAAACCTAACAACAACGATACAGCGAACTAAAACAAAGAAGGGCCGCCCCGCTGAAGCCAAGTTTCAGCCAATTGCCGCAATACCGATATCACTTGGAAGCAGAACGCAAACGGCAGATTAGCCTTAATGAAAAACTCAAAGCGGCTTTTCACCAGAGATAAAATTTGGAACCTAGTGGACATGTGAGAATGGCTATGAAACTCACCTCTAACGCCAAACCACCAAGACAAATAACAACTAAATCTTGCTGCACCATAACGCCAGCTTAAACCGCAGACAACGCACAAAACAAGCTGCTGCATTACGCCTTAAACACTAAATTCAA

>NODE_25_length_3957_cov_1.863402

CCCTTAGGCAACCTTTGAACACTGGATTCTGCAGTAGATGGGCTCTGTAGCATCTTGTCCACCAAATTTTGGTCAAGATCCGAAGCGCTCGTGAACTGATGCTTTTGTACTACGTATTTCTTCAATGCTGCTTCCAGCCTTGGGAATACAACAGGCTTAAGTAGATAGTCGACAACACCACCACGCATCGCCTGTTGCAGTGTATCGACATCACGAGCTGCGGTAATAAGAATGACATCGCAGCTTTGGTTGCGGGCACGCAGTTCCTGTAAAAGCTCCAACCCTGTTCCATCCGGAAGATACACATCAAGTAACAGGAGATCTGGGTTCAACACATCAAGCTGTATCTCAGCTTCGACTTTCGTAGTGGCAATCCCGATAACCTCATAGCCTTCCATCTGTTCCAGATAACGTTGATGTAATTGAGCAATGTTGAGATCGTCTTCAATAATCATCACTCGTGTTACGGATGTCATACCTGCTCTTCCTTCGGTAAATACACTGTCATTCTAGCTCCATGCTGTTCGTTATTCGTGATTTCCAGCGCGCCGTGATAGCGCACTGCTAACTCATTCACCAAGTAGAGGCCCACTCCTCTGGTCGACTTGTTTTTGGTTGACACACCCCGTTCAACCAAATGCTCTACTGAAAGATCTTCGGGTAAACCACAACCTTGATCCGTGACTTCGAGAATGATTTCATTCCCGAAATCACCAATGGAAACTTCAATTTGTCTTCGTACTGGGGCAAACATGGCATCCAAAGTAATCGCGCTGCGCGTGGCGTCAAAAGCGTTATCAATAAGGTTGCCCAAAATAGTCACAAGATCTTCTGCATTAATCCGCTCAGGCAGAGGCTCAAGTCGGGAGCCCTCTTCGACGATAAGTTCAAGCCCCAGCTCGCGAGCACGCTCCGTCTTGCCCAGCAGCATGCCTGCGATCAATGGCTCTTTGACGGTTTCACGTAAGAACTCAATCAAAGACTGGTAATGTGCGGTTTCCTGGCCAATCAGATGCTGAACCGCATCCAGCTCACCCATTTGAACCATACCGCTAATAGTGTTCAGCTTATTGCGATGTTCATGAGTCTGAGAGCGAAGCATCTCTGCGTACTCTTTGGTTTTCGCTAGCTGCTCCGATAGCTCATTAAGCTCATCCCTCAAGCGAAAACTCGATACGGCCCCCACCACTTTTCCTTCAACAATTATCGGGCTACGGTTTGCAATTAATCTTTGTTTGTTGAGATAAAGGTTTATGTCATGATCGGTGCGGGCTGTCGTAAGAACAGTGTGAAGGTCACTCTCAGGCAATACTTCGTTTAACGGCTTATTCAGGCATTGAGACTTTTCCAAACCCAATATATAACACGCACTCTTGTTAATTGAACGCAGTTTACCCGACTGATCGATGCTCAAAATGCCTTCTTTGAGCGTTTCCATCGTCACGTCTAACTCAACGTACAAACGACCGATTTCTTCAGGTTCAAAGCCAAGGATCGCTTTCTGGAAACGCCGAGATGCATAACTTGAGACAATAGCATTCGCTGCAACCACCAATAACGCCATTAAGATCAAAAATATCAGAAAAGGTTCAAGTCGGTCCTGAAGGCGGTCAATGAGATAGCCAACCGATACCACACCTATGATGTTGCCATCCGAATCAAAAACGGCCGCTTTCCCTCGAACGGAGTAACCCAACGAGCCTTGGGCGAAAGAAACGTATTTCTTACCTTCAATCGGAGCACGATCGTTATCGCCCCCTTTCATTGGTTTACCAATACGTTCATCAATAGGATGGATGATGCGAATACCGTCCTTATCGCCAATAACAATAAACGCAGCGCCAATCAACTCGGTAAGATTACGATAACGAAGCTGATATTCTGAGCCACCACCATCTTCTATCATTTTTACGACGAAGGGAGAGTTTGCCAAAAATGAGGCGACACCAAGCGCTTGCTTCCCCATCTTTTCTTCCTGCTCTTGCTTAACGTAGAAGAAACCTGCGGCAGCAAGCAACAACAATTCAATCAAGCCAGAAAGCGTCATGATAATCAGCATTCGCTTGCGAAAGCTGATACTACTCCATTTCATAAACTCTCCCATTTATCCTATCGAGGCTAACATCCAATTAACATTTATACGAGTTACTGGGCACTATTTTGTGAGGCCTATTATCTTAACCATTCCCTCCTGTTATTGAGCTACTCCCAACAATTCGCCTTATGTGTTGTTTGATAGTTTTAAACACACTTTTATATAAAACCAGTAGAATAATTGATTTTGATCACAAGAGTTTTAGAACATTTTTTGTACTACTTACTTATTTAGACTGTATAATTATTGAGCATGAAAAATGTAGGAACGCTAGAAGTGGGCAGTGAGTACTAATAAAAGCACGAAAAACAACCGTCCACAATACAATGTCAGAGGGAAAGACGATGAAACGGGATATGTTTGGTATCTGCCTGTCAAAATCTATGCTGTCACGTAATCTGTCCAGCACCTTTACTCACGTAAGGGCCTATGAAAAGTCCGACTTAGATAACGACGTGAAGGTTCTGCATGCCTTTCCGCAATTATCCGGTCAGGAGCTGCTGACGACGATTCAATCCTCCAAGCAATTACTATGGCGAGCGGAATATGTCTGCTTAAATCAAAAGTAAGCGACAACAAAAAGGGAAGCAAATTGCTTCCCTTTTTTAAATCTACTACCCTGTCGCCTCTTGAGCGGCAGCCCTGAGTTGAGACAAGTATTTAATCCAACTCTTCGCCTGACTTGATGGTTCGATATTGTTGGCTCTTTTCGCTTGCACTAACGCTTTCTCAAGCAGATTCAATTTGTACAAGGCACGCGTTTTGGCTAGCGCCACGTCTGCTTCACGGCCTTTCACTTTATCTAGCACCGACAAAGAATCTTTGTAATAGCCCTGCTGAACCATGAGCTGAGCGACACTCCAGTTATACTTAGCGTCTTTCTTGGCTGCTAACTTCCAAATAGATAAAGATTTATCCCACTCTTTTGCCAACTGCCAGTAAGTGGCCTGCTCCGATAACAACTGAACATCCGTTTGCGCGTCATCCAGTTCACTTATTTCAATCGCGGCCCTTTCTGGGATACCACGCTTGGCATACATCTGGGCCAGCATGCGTCTGTCACTCTGGCTCAACGGCAACCCATTGATTTTGGCCAGAGCTAAGGTATCAAGCGCCTCTTTGTCCTTGCCGACTTTCATTTGCAAGCCAACCAGTTGGCGCCACCAGCTCAGTTTCTCCGGCTGGATACCGATGAGTAACTCAAGGGTTGGGATAGCTGCGCTCCAGCGTTTCAGCTCCAGTTGAGCACCCAGCTTGAGTGATAGCGGTTGAAGAATCTCTTTTTTCTCGACCTTTAGGTACTTATCGATCGCAGGTACTACTTGCTCTAGTTGATAGTGCGACTGTGCAATTCGCAACCACAAATCCTGAGCTTTTTGGGTTTCAGGAACCGACTTAACTAACTGGTAATAGTGCTTAAGTGCCTTAGCAAACTGCTGGTCATTAAGGTACAGATCAGCCAGCATACGTTGGCTGATCCACGCCTGTTCATCACGCAGTAGACCACTTTCAACCGCATACTCCATTTTCGAAATGGCTTGCTTTGTGTTGCCATCTTGCCAGTAGAATACCGCCAACATACGAGCGACAAATGCCTTGTCGTAGTCGCGCGAGGTGTCGGTTTCTTTAAGCAAAGTAATCGCCTCTTTCAGCTGTTCTTCCTGAGCGAGTTCATTGGCTTTCTGAACACGAACCGCAGTGTATTGAGTCAGCTCCTGAGCCGAGGTTAATGGCGAAGCCAGAAGCAAAGCGATAAGGGCTAATCGTTTCATCATTTGGCTAACTTAAACTCCAGTCTTACAGTTTGGTTTTCCTGAGATACAGCGGTCCCATCCACGACTTTAGGCTGATACTTCCACTTC

>NODE_26_length_3836_cov_2.292897

GCGTTCTTCGGGCAAAGCCCAAAAGAACGATCACCACCTTCTAAAGAAGGTGGTTTAGGGCCAGAAAATAAAAAAGCCTGCAGTTGCAGGCTTTATGTAGAATTTGTTTTTTTAGCTAGTCAGGTCATCGAAGAACTTCTTCACGCCATTGAAAAAGCCTTCCGCTTTTGGCTTGTGCTTCGTTGCCGCTTCACCACCACAAGACTCTTCAAACTCTTTCAATAGATCTTTCTGACGAGTACTTAGATTTACCGGAGTTTCTACAACCAGCTTAACGATAAGGTCGCCAACGCCACCGCCGCGAACACCCTTCACGCCTTTACCGCGCATACGGAACATGCGACCCGTTTGAGTTTCGGTTGGTACTTTCAGGTTCACACGACCATCCAGTGTTGGTACTTCGACTTCACCACCCAGTGCAGCCATTGCAAAGCTGACAGGCACTTCACAATAAAGGTTGTTGCCATCACGCTCAAAGATTTGGTGCTCTTTCACATGAACCTGTACGTATAGGTCACCCGCTGGAGCGCCCATTTCTCCTGCTTCACCTTCACCAGAAAGACGAATACGATCACCAGTATCAACACCAGCAGGAATCTTAACGTTAAGCGTTTTAGTCTTCTGCTTGCGGCCTTGACCATGACATGAGTTACAAGGATCTTTGATGATCTTGCCCTTACCATGACAGGTAGGACAGGTTTGCTGAACCGCAAAGAAGCCCTGACGCATCTGAACCTGACCATGACCGTGACAGGTTCCACACGTTTCAGGGGACGAGCCTTTCTTCGCGCCACTGCCATCACAAACGTCACAGTTAACTAGCGTTGGAACTTCAATTTCTTTTGAAACACCACGAACCGCTTCTTCTAGCGACAGTTCCATGTTGTAGCGCAGGTCTGCACCGCGTTGCGCGCGCTGCTGGCCACCACCACGTCGGCCACCACCAAAGATATCACCAAACACGTCACCAAAAATGTCACCGAAATCAGCACCAGCACCACCGCCAAAGCCACCACCGCCCATGCCGCCTTGTTCGAAGGCTGCGTGACCATATTGGTCATAGGCTGCTTTTTTCTGAGGATCCAGTAGGATTTCGTACGCTTCTTTTACTTCTTTAAACTTCTCTGCAGCAGACTCATCACCCTGGTTACGGTCTGGGTGGAATTTCATTGCAAGGCGCTTGTACGCCTTTTTAATATCACGTTCTGATGCATCTCGGCTTACGCCTAATACTTCGTAAAAATCACGTTTTGACATGTTCTTCGTCACCAATTTATTACTGCAAACGACCGATTGTCGCTTGGTGTTTGTATCGTTCTAGATAAAGGCTTTGTGCAAAAGTGCTTATCTGGAAAGACAGCGTATTTACAAACAGGCGGGCGTGTGAGTTCCCTCAAACGCCCGCATATTACTTTAAGTTAAGTCAGAATCGAGAATTATTTTTTCTCGTCTTTAACTTCTTCAAACTCAGCGTCAACAACGTCTTCTTCTTGCTTAGGCTGTTCACCAGCTTCTGCACCTGCTTGTTGAGCCTGAGCTTGCTGCTGAGCGATTTCCATCAGCTTCTGAGCTGCCGTCATAAGCGCTTGAACTTTCGCATCGATAGCTTCTTTATCGTCGCCACCTTTTGCTTCTTCAAGTTCAGTAATTGCAGCTTCGATCTTCTCTTTTTCTTCAGCTGGAAGTGCGTCACCCGCTTCTTCAACTTGCTTGCGAGTACCGTGGATCATTTGGTCAGCTTGGTTACGTGCAGCTGCTAGCTCTTCGAACTTCTTGTCCGCTTCTTTGTTAGCTTCTGCTTCTTGAACCATTTTCTCGATTTCGTCGTCGCTTAGACCGCCTGAAGCTTGGATAGTGATCTTCTGCTCTTTACCTGTCTGTTTGTCTTTCGCAGATACGTGCAGGATACCATCCGCATCAAGGTCGAAAGTTACTTCGATTTGAGGCATACCACGAGGTGCTGGCTGAATGCCTTCTAGGTTAAATTGACCTAGAGACTTGTTGTACATCGCTTGCTTACGCTCACCTTGAAGAACGTGAATAGTTACCGCGTTCTGGTTGTCTTCTGCTGTAGAGAAAACTTGGTTCGCTTTCGTTGGGATAGTTGTGTTCTTCTCAACTAGCTTAGTCATTACGCCGCCCATAGTCTCGATACCTAGAGACAGAGGAGTAACGTCTAGTAGAAGTACGTCTTTCACATCACCAGCAAGTACACCACCTTGAACTGCAGCACCCATTGCTACTGCTTCGTCAGGGTTCACGTCTTTACGAGCTTCTTTACCGAAGAACTCAGCCACTTTCTCTTGAACCATAGGCATACGAGTCTGACCACCTACTAGGATTACGTCAGTGATGTCGCCTACAGAAAGGTCTGCGTCAGCTAGAGCAACTTTTAGTGGCTCAAGAGAGCGTTGTACTAGGTCTTCAACTAGAGATTCTAGTTTCGCACGCGTTACTTTAACGTTCATGTGCTTAGGACCAGTCGCATCTGCAGTTACGTAAGGTAGGTTTACGTCAGTTTGAGAAGTAGAAGAAAGCTCGATCTTCGCTTTTTCTGCTGCTTCTTTAACACGCTGCATTGCTAGAGGATCATTCTTAAGATCGATACCTTGCTCTTTCTTGAACTCTTCAACTAGGTAGTTGATCATGCGGTTATCGAAGTCTTCACCACCTAAGTGAGTGTCACCGTTAGTTGCTAGTACTTCGAACGTTTTCTCGCCTTCAACTTCATCAATCTCGATGATAGAGATATCAAATGTACCACCACCAAGGTCGTATACAGCGATAGTGCGGTCACCGCCTTGCTTGTCTAGACCGTATGCTAGAGCAGCAGCTGTTGGTTCGTTGATGATACGCTTAACTTCTAGACCTGCGATACGGCCAGCATCTTTAGTTGCTTGACGCTGAGCATCGTTAAAGTAAGCAGGTACTGTGATAACTGCGCCAGTTACTTCCTCACCTAGGAAGTCTTCTGCAGTTTTCTTCATTTTCTTCAGTACTTCAGCAGAAACCTGAGGAGCCGCCATTTTCTGGCCTTGTGCCTCAACCCAAGCATCACCGTTGTCAGCCTTAACAATGTTGAAAGGCATGATTTCGATGTCACGCTGAACTTCTTCGTCTTCGAAACGACGACCGATTAGACGCTTAATTGCAAATAGCGTGTTAGTTGGGTTTGTAACCGCTTGACGTTTAGCTGGCTGACCTACTAGCGTCTCACCATCTGTGTATGCAATAACTGATGCTGTTGTACGCTCGCCTTCCGCGTTTTCGATTACGCGTGGCTTGTCACCGTCTAGAACAGCAACACAAGAGTTAGTAGTACCTAAGTCAATACCAATGATTTTACCCATCAGGCTATCTCCGAATAAATTCTATTTTCGTTTTGCTATATCCCCTATGTGGGGATGGAGAATAGGGTTTCAACCCTTACTCACAAACTAAAATAATTTTGTTTGCTTCACTTATGCCCAATAGATAAGGGCTGTAAATAGCTTTTCAAGGGAAGAAGTGAAAAAAAGTGAAAAAAGATTTGAGGATAAGCGAATAGACCGGATTCCGAAGTCATAATTTCAATAAGTGGTGCTCCAACACTTACATCTTCGTTCTCTCGAGTGGTAATGCTCAAAGCCCAATTAATGTCATTACTACATGCTAAAAACAAAAAGCGAGGCACTTGGCCTCGCTTTCAAATTCCGACACTTTATAGATTAAGCATTCGCTTGTGCAGGTTGTGCTTCCACTTCTGTTTCTTCTTTGCCGATTTCGCCTTCTGATTTAGCAACGATAGTATTTACAGTTGTGTCGCCTACTACGTTAGAAGATGTACAGAACATATC

>NODE_27_length_3819_cov_2.323624

CTTGTACATGCCACCAGGAACCGTTGCAGTACGGTAGTAGCTGGCATCAGACAGAAGCTTGTCGACTTGCGGACTTGCTACCTGAACGATATTACTTTCACATGACGTGGTGGCTTCCTGAATTGCACCACTTGGGTGGCCTACCACATAAACCATCGCGTCAATTTTGTTATCGCACAATGCTTTCGACTGTTCAGCTGCTTTCAATTCTGAAGCCAGCTTAAAGTCCTTAGTTGTCCAACCATACTGGTCCATCAGAACTTCCATTGTGCCGCGTTGCCCCGAACCTGGGTTACCGATGTTGACACGTTTACCTTTCAGGTCTTCAAAGCTCTTGATGCCCGAATCGGCGCGAGCAACAACGGTGAACGGTTCTGGGTGAACAGAGAACACAGCGCGTAGCTCTTTGAATGGGCCCGCTTCAGAGAACTTGCTTGTTCCGTTGTAGCCGTGATATTGCCAGTCAGACTGTGCGATACCTAAGTCCAGTTCACCCGCACGAATCGTGTTGATGTTGTAGATGGAACCACCGGTAGACTCAACAGAACAACGAATGCCGTGTTCTTTTTTCGTCTTGTTTACCAAACGGCAAATAGCACCGCCTGTTGGGTAGTAAACGCCTGTTACACCACCAGTACCAATGGTTACAAATCTGTCAGCAGCCGAAGCGTAAAAAGTGGCGCCTAAAAGTGCAGTTGCTGAGGCGGCAGTCAGTACGTTTTTTAGCAATGTCATAATAATTCCTTTTTATTGCGTGTAACGATCCTATCTTACTTACCAGAGGCACGAATCAGTTTTTGTTTAGTGCCTTTCGGAAACTATAAGCATCATGAATGCAAATGTTTAATAATTTGTTAATTTTTTTGAGGTTTCTCACTTGTTCCGCATTATTAATGGCTGGATTTCCTTGTGGATCAAGGTGATTGTTCTCAAACCCAACTCGTACATCGAGGCCGAGTAGCATGGCACTGATTAGGCAGTCCTGCTCACGATCTCCAAACGCACATACTGCGAGGTGGACGTTTTGTTGAACAAACCGACTCAAGTCGAGTTGTTTTAAATCCCACGGACTGGATTGCTGTTCGTCATGGTATCGGCCAAGAACGATGAGAGCGTGTTGATTCATTGTCGGCAAGATTCCCTGAGATCGAAACCGGAAGTAGCGGTCAATATCATCTTGATCGTAGAGAATGATTTGGCTGATGATTCCGTGTTCCGCGACCCAGTGGAAAAAGGCCTTTCCAGACTCAATGTGGTTGTCATCGGGTATCAATTCACGCAGTGCGAATGAGGCGGCTTCGGGCTTGACCGCTTTAGTCAGTGCTATTTGCTGCTCTGCACTATAAATCCCTACGGCTTCGGTTGTCAGCTGGACGATAATGCTGTCACCGACGGCCTGTTTCACCTTGTCATACAGTTGGAGATTGGGTTCGATCTCTAGGCTGTGCTGACCATGTTCATCTCGACAGTGAAGATGAATCATTGCAGCGCCGGCATCACGACATGCAATGACCTCAGAGATAATTTCTTCCTCGGTCATTGGAAGATTGGGATGATCTCGCTTGGTTTTACGCGCTCCATTTGGTGCCACGATAATTGCGCTGGTTTGCTGGTGGCCAGCATCAACTGTTGCTAAATCCATGCTCCGGACACCTCAGTCAATGTGGTTTTCAGTTTAGCCACAATCTCTTCGATGTGGCTTGGTTCGATGATGAAAGGTGGCGCTAACAGAATGTGATGACCTTGTTTTCCGTCAATCGTCCCGGACATTGGGTAACACATCAGGCAATTTTTCATTGCTTGAGACTTAACCGCAGTGTGGGCTTTTGTGGCGATATTGAGCGGAGCTTTGCTTGCTTTATCCTTGACGAGTTCAATGCCAAGGAAGAGTCCTTTGCCTCGAATATCGCCCACGTAAGGCAAGTTGCCCAACTCACTCGACAGGGCTTGTTTAAGCTCTGCTCCGTGAGATTGCACCGCAGACAACAAAGATTGTTGTTGAATAGCTTCGACTGTGGCCAGTGCAGAGGCACATGCCATCGGGTGCGCCATAAAGGTGTGACCATGCTGGAAGAAGCCGCTGCCATCGGCAATGGCTTGGTAAATCCGGTCATTGGCGATGGTGGCACCGATAGGTTGATAACCCGCTCCCAGTCCTTTCGCAATACAGACCAAATCGGGTTCTATCTGCTCTTGCTCGAATGCAAAGAAACTGCCGCTGCGACCGACTCCGCACATGACTTCATCCAAAATGAGCAACACTTCATATTGATCGCAAATCTCTCGAATTCGTTTGAAGTAGCCTTCTGTCGCAGGCACGGCGCCAGCAGTCGCGCCAACAACAGGTTCAGCGACAAACGCCATGACGTTTTCTGCGCCTAATTCGAGGATTTTTTCTTCAAGCTGATTGGCACTGCGAAGTGAATAGTCCAGCTCTGACTCTTGTGGTTGTTGTAAACGGTAGGCGTAGCAAGGATCGATATGGTGACTAGTTGTGAGAAGTGGCTGAAATGGCTCACGACGCCATTCATTTCCACCGACAGCAAGTGCTCCTAGCGTGTTTCCGTGGTAACTCTGTTTACGCGCAATGAACTGAGTCTTGCTTGGTTTTCCTAGCTCAACAAAATACTGTCGAGCCATTTTTAGCGCCGACTCCACGGCTTCAGAACCTCCGCTGACAAAGTAAGCGTGATTGAATTGCGAAGGCATCAAAGCACACAGTGCATCGGCCAATTGTTCACAGGGCTCGTTGGTAAAAAAACCGGTGTGAGCAAAAGGAATACGCTTAAGTTGTTCAGCGATGGCTTGTTTGACCATCGGATGGCTATGGCCAAGATTAGAAACTGCAGCGCCGCCACAAGCATCTAGATAAGCTTTTCCATGTTTATCATAAAGATAAACACCTTCGCCGTGGCTGATAGTAGGTAGTTGTGTATGACAGTGTCGGTGGAATACGTGAGACATAACAGGTCACTCGGTGGTCGCATTAGCCTTATCCTGTACAACAAAGCTTAGACGCGCAATCGATTAAAAGTTGACCGCTATGAGTTTTTGGAATGAGGTTGAGAATACAGAGATGAAAAAAAGCAGCCAACAGGCTGCTTTTAAAACAATTAGATATCGCGATTTTCGGCAAATTTTTCCAAGCCGAGGACCAGCGCAATGGCACACAGCATGAGAACGATGGCGATGCCTATCTGAGCAGGTTGTGAGGTGATGGCTTCAAAATCAAACGGTGATAGGTTGCGTTCAATCAACGGGACTTGTTCACCACTTGAGTTAGTGCGCCACGTCAACGTTTCTTTCCAAGGCCAGATCTTTGGTAATGTGCCAATCATTAGGCCAGTCAGGAAGATAAGAGTGATGTCTCTGAATCGTTTTAGGAGCCAGGATAAGACGTGGGAAAAGCTCAGTAAACCAATGACACAGCCCGTGAGGAATAACAGCAGAATGGTGATATCCAATGATTTGACTGCGCCAAGTACAGGGCTGTACATACCAATGAGCAGCAGGATAAAACTGCCAGAGATACCGGGTAAGATCATCGCACAAATGGCGATCGCTCCAGCAAACATGACATTAATGCTGGTCGGATCCATTTGAAGGGGTTTGAGGATGGTAATGCTGTAAGCAAACCCGACCCCAAGTAATAGGAAGATAAGGCGACTGAAGGTTCTTTTTTCTATTTGTTTCAGTATATGGAAAACGGAAACCAGAATCAGACCAAAGAAAAAAGACCATAACGGAATAGGGTGTGTCACTAGCAACCACGAAATCAGCTTAGCTAGTGTTGCAATGCTGGTGAGAACGCCCCCGAAT

>NODE_28_length_3774_cov_2.029213

GAGCAACAACAGGTACCATAACAACCTCTACAATATACTTTCTATGTAGTTATAGGTAATGACTAATCCCAATACCAGTGTTTACCTATAACTACAGATTTTATTTTGTTCTATCCAATAAAACGGCTCCCAACTGTTACCAGCCAGGAGCCGAATGTGAATTATATCACACGATTACTTGGTAATGCTCAGTTTCTTTACATCGCCATTACGTGTTACTGCAATGTAGCCATCTGGCACATTAATTGGCGCAACTGCGAAACCGCTGCTGTTGATTTGCTGCTGTGCAACAAATTCGCCAGTATCTCGGTCAAGCCAATGCAGATAACCTTCGCTATCGCCCACAACCACATAGCCATTAATGAGTGCCGGTGCGGTCAATAGACGGTGTTCAAGTTGCTCGTTACTCCAAAGCTCTGTGCCACTGCGTGCATCAACGGCAACCAGATAATCTTTATCTGTCACAACAAACAGACGACCACCATCGCTGGCAAGATCAGTAGATGACGAGTATTTACGCTTCCAGACTGACTTACCCGAGCGCAGATCAATAGCGGTCAACTGACCGTTATAACCAACAATATACAGTGTGCTACCAAGAACCAGTGGAGAAGCATCAACGTCGACTAGACGATCGATTTCTGTTGCTCCTTGTGGCGTACCGATAGGCTGTTGCCAAATCAGCTGACCACGCTCTGCAATCGCAGCCGCTAGACGACCATTCGCTGTTCCCCAGAATACACCGCCAGATACCGTCACAGGAGCGCTGTTACCGCGCAGAGTCAGACTTGGCACTTCTGTGCTGATGGTCCACTTGATATCACCGGTTGCCTGATCCAGAGCAATCAACATACCCGTTGCAGTGTGAACAATAACTAAGTTGCTTTCCGTCGCAGGTGCCGCTAGCACTTCACCTTCAACATCTACACGCCAGTTCACTTCACCGGTTTCTTCGTTCAGGGCAATCACCTGACCGTTTTCACTACCAATAAAAATTTGGCTGTAACCTGCAGTGATGCCACCAGATAAGCGAGCGGGAACATTCTGTTCAAGATCTTGTTGCCACAAGGTACTGCCATTTTCAGGGCTCAGTGCCTTCACAATACCTTCACGGCTTGCAACAAAGACTTTGTCATAAGCATATTCAGGAGACAATTTAGAGAAGTAGTTTGATACGCCATCACCAACACTGGCACTCCATTCACTCTTCGGAGTGAATTCACTGTCAACCTGAGGCACTGGCGCCATGATAATGGTATCTTCTTCACTGGCACAGCCAGCAAGAACACCCAAAATGGCCGCCGCAGTGAGGGCTTTTTTCAGCATTTTATTCATGCACAGCACCATTACTTCGCCAGGTCGTCCAGTTTCATTTGTATAGTCTGACTTGCATCATCCGCTTGCTGTGCTTCGGTATAAGCTGCGTACGCACCTTCCGCATCACCTTTACGAAGAAGGATATCCCCCTTCAGCTCAGCAACACGTCCAGCCCAGCTTTCATCATTAATTGCAGCTAGTTCAGAAACAGCCGCATCAAATTCACCCTGTTCTGCTTTCAAGCGAGCAAGACGGAAAGAAATAACAGAAATTAGTGCTTGGTCTTCAGTCGCTGTTTTTGCCCATTCAAGCTGCGCAAGTGCTTCATCTAAGCTGCCCGCTTCAACCTGCACTTTCGCTAGTTGAAGAGCTGCAAGGACTGCATACTGAGTCTCTTTGTTAGCATCAATAAAGCTCTGAACCGCACCTTCGCCTTCAGCACCTTTGGCCGCCAGTTCCTGAACAGCCTTGGTATAACTTTCAGACGCAGCTTCCTGAGCTGCTGTCACTGAATCTTGGTAGTAGCGCCAACCAAATAGGCCACCCAGACCGATAACCGCGCCGAAAATGACCGCTTTACCGTTTTCTTTCCACCAATCTTTGATCGCTTCTACTTGTTGTTCTTCAGTATCGTAGAGTTCCACTTCCTGTCCTCTTGAATCTAAAAAGTGGTGACTAGAATCACCACTTCAAATTTGAATGCGTTTAAACCAACTCTGCCAGCTTAGCTGCAACGTCTGCTTGGCCGTAAGTTTCTTGAGTGCCGCCAATCAGGTCTTTCAGTACCACAGTGTTATCTGCTACTTCGTTTTCACCAAGAACCAGCGCCACTGCTGCACCTACCTTGTCGGCACGCTTAAATTGTTTCTTAAAGTTACCACCACCGAAATGGTTCATCACACGAATGCCTGGAACTTGCTCACGTAGTAACTCAGCCAGCTTCATGCCCGCCATCATAGTGCCTTCGCCCGCAGTAACCATGTAGACATCAACAGAGCGGCGAACGTCTGTCAGTTCTAGTGTTTCTAGCATGAGAACCAGACGCTCAAGACCCATTGCAAAACCGACAGCGACTTTTTCAGAGTACTTGTGAGAGATATAAAAGTTAGGAATAACGGCATCATTCGCGAAATCATCGGAAGATGCATTAGATGTAGGAAGTAAAGGGCTTAAAGACTTTGAGCCATCCACACTACCACTAACATCAACATTAGGATTTACATAGATAGCACCGACAGAAATCTGAGTACCTTCGAGATAGGTTAGCATTGCTGGGTTGCGCCACTGTGCGCTCGCGTTGTCGGCCATAGCTGCTTCACCAGCGTATGCACGACCTAAACCTGTTGCTGAATATTCAGCGAGCTGGAATCCTGCTGCGTTTACTGATGCAGATGCTGAAAGAAGGCTACCTGCCACTGCAAGAGATAAGAGAGTTTTGTTTGTTTTCATTTTTATGTTCGCTGAACGTATTGATAATTTGTCGTGCGATCATAAGTTTAGAGCTATTACTTTAAAAATTAAAAACAGTGTTAACTCTATATTTGGCATAAAATATTGTAATGTGGTCATATCCCTTTCTAATTGACTAAATGTTAATGGCTTGTTTTCGATGTTGAGCGTACATACGTACTATGAAAGGGGTATTTTTGAGTACATGTGTAAGCTGAAAATAGAGTAGAAACAGTGGGTTGAGTAGAGTGTAAGACTTGCTGTAAAAGAAAAGGGTCAGTTTCCTGACCCTTTCAAGAGATAAATTATGCTAACTTTCTAACTTAGAAGCTACGGCTGTACTGTAGGCCTAACAAGATAGCATCCGCACGAGTTGTACCAGTTACTTCTGAAAGAAGTAGGGCAGAGTTGGTTGGGCTATATGTTGTTTCTTCGACTTTAACATCCTCACCCATGAGATAAGTGAAGCCAAAATCGATGTCAGATTTTTCATCTAGGTGGTAAGTAAATCCAGCGGACAGCCAGTTACGGTCTGAGTCCGGAACAGAGATAGAAGTCACTTGATCTTGGGCACTTGTGTCATGCATATAACCAGCACGCAATGTCCAGTCTTGGTTCAAGTAATATGTACCACCGATAGCATAGTGCATACCATTTTGCCATTTGTAGTCATTAATATCACCTGCTTTGTCCGCGACTAACTTATCGAATTCACTCCAGTTTATCCACTGTACGCTATAATGCACAGCGTATTTGTCATTAAGGCGATGGTAACCTGAAAATTCAGCCATATCTGGTAGTGGCATATGTAGCGTATCGGAGGTGTAAGTAGTACCCAGCTTGGTAATATCACCTTCTGCTTCTAAATCAGGGCTATAGCGGTAAGAAAATCCGAAACGGTTTTTATCATCCATTTCATAAACTGTACCGACATTAAAACCCACAGCCCAACCGTCTGCTTTAGTATCAAGTGCTGTAAATGAAGTTGATGCTCCGCTAGTTAAAACG

>NODE_29_length_3734_cov_2.537599

GGCATTACTGAATGTACAAGTTTCGCTATTTCCATAACGGTATGGTCGGCAGTCCCAATTCTGTTTACTGGCTTCCGAACCAAACTTAACGTAAAGATCCACATCACCAGAGCCACCGGAGGTCTTAATATCAAGTGTTTTAGAAGAAGTCAGTTCGAAACTGTACGCGACCTCGCTGCCAGTTGCTCCCGATAAGCCAGTCACTGGCACACCATCCTGAAGGACGTTATCAGTAGGAGGAGGTGTTGTTCCACCAGATGCATTGCCCATCTCGACTGCGTAAGCAAGACCAAGCTTAGTAAATTTGGTTGCGTGAGCACCGGTGGGATCCGAATTCTCTAAGGTGTCCTGTGAAGTGTGAATCTTAGGGTTGTAGTCATTAAAGTTTGACTCAAAAGGCATCGCAGCAGAATACCCAGCGTTGTGCCAAGAAGCGTGGTCTGAACACGCATAGCCACAACGATCATAGCCATAACTCAACGCTGGCAGATATTCGTCAAGCAAGTTAGCTAAGAACTGAGTCAGGCTGCTGTCAGTGTAATCGGTAATGAAGACGATATCTTGAGCAGAGCCTTGATAGTTGGTCATATCCAACTGAAGGGCAGACACGACATTTTTACCCTGAGATTTATAGGAGTTGGCGATATCCTGAGAGCCACGCAGCCCTACTTCTTCTGCTGCGTAAGCCATAAAGGCCATCGAGCGTTTAGGCTTGAAATTGTTCTCAGCCAGAACACGAATTATCTCCGTCACACTTGCGATGCCCGACGCATCATCGTCAGCACCGGGGGCAATTGTGTGTTCATCCGTTCTTGAGCCAATAGTGGAATCTAGATGACCACCAATCACAACCCATTCATCAGGGCTTTCTGAACCCTGAATCGTCAAGATCACAGATTTCTGGTTGTAACCTGAGTGAGAGAACTGCTCAACACTGGCATTAGACAACCCTGAAATCAAGGAGCGCCACTCGCTGGCAATCCAATCAGACGCTTGCGCACCAGAGGTGGTAGTGTAAAAACGGTTGTTGAAGCTCGTCAGCGAAGTGATTGTGCCAGTGATCTGCGCCGAATCCACTTGCGGCAGCCATGCATTAACGGTTGTTTGTTGAGAAATATCAGGGATTGCAAAAGTATTGAGGGCCAGTGGCATCGCGCTTGCCGCCATTGCACTCTGAGCGGAAGGGTGAACCATGTAGCCCCCACAACGATGATGCTCTTCATGCATGTTGTGAGACAATTCTGCTAGTTCCGTTTCATCAACCTGCCCGACCCAAACACTCCCACTGCTAGCCAGTGAATTCGGCAGCACAGCTTGCGCCCCTGATTTCGCCACTGTTTTGGATGCATCAGCACCGATTGATATCCATACTTTGTCTTCTGCGTGTGCATTCATGAAAGCCGTGGAAAGCGCCATAGCCAAAAGCGTTTTAGCGTAGTTCATTATCTCCTCCATAGAGTTTTATAAAATCACCAAAACTTAGGATATTTATTTACATTTTGAGAATTATTCGATTTACATCAGGCCGTAACAATATGTTGTCACTTTTATATCAATACAGAGAATGGTTAATAAGGGCACCGAAGTGCCCCAGGCTTGACTCAGACTATTTTGCCGTTACGGACAAGGTTACGCCTGAAAAATTGCTGTACGCATTGAGCATAATGTAAAGATCTTCACTCGCATTCACTGCATCACATGACTCCGTATTACCAGAGCGATATGGTCTGCAGTCGTAGGCACTCCTAGTTGGTTGAAGCCCTGAACGAACATACAGATCGGCATCGCCCGATCCAGATGCAATAGACACTGTGACGTCAGAAGCACGTTTATCTGAGCGAAGGACATACATTTTCTGCTCACCTTTCGCTAAAGCAAGACCAGACAACGGCGTATCTAGGCTCAGCTCAGTGATATCACTTCCTGGTTGTTCAACCATGCCATCGGGCAGAGTGTTGTTAAAATGGCTCGCCACTTTTGGCCAGATTTTACTGATTTTCACCCCTTGGTTTTCGCAGCCACCAAAGTGATGCAGCGCAATCGCTTTGTTACTGCTGGTTTTGAGCACTGGTGAACCAGAAGAGCCACCAATAGTGTCACAGAAGTAACCTGTATCGGTGTTCACTCCACGGCCATTGGCCGAAGCAACATCAATTTGACACAGTCCGGAACCATTCTGGTCGCTTTCAATCGCGAGCTCTTTCGGGTTACCTGAACCGTGTTGCGGAATATAAATACCGTCACCATAAGTCGGCTCGGAAGCATCCAGACCCAGGTAACCAAACGAGGCGATCTTGGCGAAATCATCAACGGTAAACAGCGTGTAGTCCAGCTCATAGTCTGTGCTGAGCATTTGATCACCCATCACTTTGACGGTCGTCGACATCGAACCATTACAGGTCGAGCGCTGATAGTTAAACCAGACTTCGGTATTTTTCAGTTCCGTTTCACTCTCAACACAGTGGTTGTTGGTAAACATACGGTTATCCGGGCCTACGCGCCATGCGGTACATAAACTACGACCGTTAATCAATAACCTCGCGACTGGCGTTGTCCAAGCGACTTTCTCTGGGTGTGAGTCTTCCCAACACGCCACATCTTTCCGCTCATTAACCCCACAGGTGGAAAGTGTCGACATCTCTTGCTCTGCCGTATCTTTACCTGCCATGTAGTAATCGAGCTCAAACTGTCCTTGCTGACCTTTAGCATTCGCTATCAGTTCAATAACAAGTGCATCGCCTGAGATAGATTGTGCAAACCAATCTAGCTGGCTGTCAGTGTACTCAACCATCTCATCCGTAGTAGCGGATTTAACACGTAAAACCGCCCCTTTTGGTAGTGAAAGCTGTTTGAAGTGCAACTTAATGAACGCAGCCCCTTCATGCTCCACTATCTGGCTCAAGTGCTGACCTGAAACACCGGTCAATTCTGCATCTGACATAGTGAATTTCACTTCATCAGCAATGACAGGCAACACTGTATCTGCCATCGCGACTGGAGTGATAAAACATGCAATAGCCAACCCAACGTTGCATAAATTTGTCTTCATTGTTTTTTCCTTTTTTGTTTATTTGTGTTGTCCCAAAAACAAACATATGCGGAAAAAACAAATAAGCAATCAATTGCATATTTATTTTTGAGTAAAGAAAATTGAACAGGTCGTTTTATTGGGGTGAATAAAAAGGCACTCAAAGAGTGCCTTAATCAAGATATAATTTATTGAAATATCAGAGCTTAAATGTCGAAACCTTGGCCATTAATTCGCCAGAAAGTCCCTTCACTGTACCCGCTTCCTGCGAGCTTTCCTGAGCACTATGAACTAACTGATCAGCGACATCTTTAATTGAAGTAATATTCTGCGTAATCTCATTCGTTACATGGGTTTGCTCTTCAGCCGCTGTCGCGATTTGTGTCGCCATGTCACTAATGGTTTGAACGGCAGTATTAATCTCTTCCAATGCCACACTCGCCTTGTCCGCATCTTCCACAGAATGCGTCGCCAGAGCGGAACTATTTTCCATCAACTTAACGGCATTACCGGTAGTAACTTGCAGCGTATCGATCGTCGCTTTTATTTCTTCAGTTGAATCATGAGTACGCTGAGACAGCACTCGAACTTCATCAGCGACAACCGCGAAGCCTCGTCCTTGTTCACCTGCACGCGCAGCCTCAATCGCTGCATTCAGTGCCAGCAAGTTTGTTTGCTCAGCAATACCCTGAATCGTCGCCAGAAC

>NODE_30_length_3724_cov_2.603236

CTGCCATCCTGCTCACCCTCTGGCTGAGCTGGATACGGTCACGATCACTGATGCCATCAAATACCGTCAGTTGTTACCGCGTGGTGACCAAGGCGCTACCCTACAGCAATTTCCCGTGCTGGCGTCCAAGGTGTGGTGGTCAAACAGCTTTAGCTCGATCAAGCACACTATTCTGCATGGCAACTTAGGTTGGTCCTATCTGCCTGCTCACATGGTGGATCCTGCTATTGAGTCAGGCGCCTTGGTTGAGTTGCATGTTTCGATTGATCACAAAACTTGGAGCCCACCTGTGGATGTGGCGACCTTGAAAAGTTCAGGTAAGGGGCCGGCACTCTTATGGTTGGAAGAGCAACTCAAGCAACTGCTTGACTGATCCTTATCACGCTTTGGTTGCCAATAAGGCGCCGAAAGAGACAAGCGCAATGCCGCAGGCTTGTTCTAGCCTTTTCTTCAATTTCAGCAAGCGCGTTTGAAGTGTTGGCGAAGTCAACAGGACGGCCACTAAACCAAACCATAGCCCGTGGAGCAGAATCATATAGCCACCGTAAAACAGCGCTAGAGTCATTTCTTCGCCGTTTGGAGAAATGACTTGGCTAAAGATACTGACAAAAAACAACATGGTTTTTGGGTTCAATGCGTTACATAGCCAGCCTTGCAATGCATAGCGCCATTGAGCTTGATGGCTGTCCATGCCTGCTTGTTGCTCTAATTCGTGGCTGCTGGATAAAAATCCTTTTATACCTAGATAAATCAGATACGCAGCGCCGAGATATTTCAGACATTGGAAAAGAAAAAGGTTTTGTGAAATTAAGTAACCAATACCTAGAATAGAGTAGGCGATGTGAACCGTAATCGCGGTACTAATGCCAATGGCTGTCCAAACACCTGCCGCTCTTCCTTGTTGAACACTGTTCCTCAACACTAAGACAAAATCCGCACCGGGACTAATGACAATAAAGAAACCGAGCACGGCTAAACTAACAAATTCCATTGTGAACCTTTGTATATTGTTTATAAGATGCAGCTATTGTAGGAAGCTTTCCTCCTTTCTATAATCGAAACTATCGCCAAAGACTTGTGAGATAATTTCACATATGCGTCACCTTAAAGCGTTTCACGCATTCCACATCGCCGCATCGTCCAGCAGTTATACTCAGGCAGCAGATCAGCTCAATTTGACGCATGGGGCCGTCAGTAAGCAAATCAAGACATTAGAGCAGTATTTGGATAAGCGCCTGTTTCACAAACAAGGCCGCAATGTGGTGCTAACGGAAGAAGGGCAACTGCTCAAGAGCTATACCGAGCAGGCGTTTGGCGCTTTATCGGCAGGTGTTGCCAAGCTAAACAGCCTCGATGCTCACTCTTTGGAAGTGTCTTGTGAGCCCACTCTTACCATGCGCTGGCTGATGCCCAAGCTGGACGAATTCTATTCTCAAACAGGGACAGATGTCCGCTTATCCACGGCAGGCGGCCCAATCAATCTCGTCAATGCGGGGCTGGATGTCGCGATTCGTCGTGATGATTTCCCGATCCCGTCGCACTATCAGATAACTCCTTTGGTGGAAGAGTGGGCGGGGCCAGTGTGTACCAAAGAATACTGGGAGAGCATTCAAGGTGATATACAAAATGCCAAGGTGCTGCATTCTCAAACGCGTTTAACAGCGTGGCAAGAGTGGTTAAACGCTTCGTCTCTCTCAGGAGTCACAACAAAATCCCAGCAAAGCTTTGCTCATTTCTATTTTTGTTTGCAGGCGGCAAGTGACGGGCTTGGGATCGCTATAGGTTCTTACCCACTGGTACACCATGACATTCAGCAAGGGCGGTTAATTGCGCCTTTGGGGTTTTCTCTTACTGGGCACAACTATGTTGCCATCCAGGCTGCCGATCATCATTCGCCACTGAGTGCGCCATTCGTCAACTGGCTTAAATCGGAAATGATTGACTGCTTACCCTGTCACTGCGCAGCATAGCTCCTACGTTATTGACCAGACATTAAAGCTCGGCAATAGAGTGAAAGCGGCCCTGTCATCATGTTTTCATCGAGTTGGTCGGTGTTAGGGTTGCAGTTAAGGGCTAAAGCAAATCCGTGTAGGTAGTCCGCCAAAAGATCAATGGCATTACGCTCTTCCGCTTCGCTTAAAGACAATGGCGCGATAATAGATTTGAAACGCTCAATGAAGACCATCGCTGGGCTGACAGATTCTATGCTGAGCAACGTGGTCAATAATCCTGGATAGCTCTTCAACAACTGGACATAGCTGGTTGCTAACTGCGTGAGCTCATGCTGCCAGTTTTCGTTAGTTTTAGGCAGATAGATGTCTTCAATCAAGGAAACTGTGATGGCTTCCAGCAGCGCATCTTTGTTTTTGAAATAGTAATAGATGGCCATCGCGTCGACATCTAACTCGCTGGCAAGTGCCCGTATGCTGGGAATTTTTCCTTGCTCTTTCATCATTGATTTTGCAACCGAAAGAATACGTTCCTCACCCAATTGAGCTTTTTCTCCTGCTGGTCTTCCGCGTTTCTTCTTGACAGTCATTTTGTTATCTCGTTATAGTATTTTCATTAATTCTACATTGTAGAATTAATGGTGTCGAGTTCGTTTTACCTATCACTATCGGAGCTGTTTTATGTCTAACATCGTGTTTATTGCTACTAGTCTAGACGGGTATATCGCGGATAAAGAAGGTGGCCTTGATTGGCTGCATTCTGTCCCGAACCCAGACAATCATGACATGGGGTTCTCCGCAATCATGGAACGCGTCGATGCACTCGTGATGGGGCGAAATACATTGGATGTGGTTTTAGGCTTTGGCAGCGAGTGGCCTTATTCCAAACCTGTTTTTGTATTGAGCAATACAATGGACAAGGTGCCGGAAGGTTATGAAGACAAGGTCTTTCTGGTCAAAGGTGAGCTGAAGGATGTATTGAATGAATTGAATGCTCAGGGCTACAAAGATCTCTACATTGATGGTGGAAAAACAGTACAAAGCTTCCTTCAGCAAGACCTGATTGATGAGATGGTCCTAACTACTATTCCAGTATTACTTGGTGGTGGCGCGCCGCTGTTTGGTAGCTTAGTGAAACCATTGAATTTCAAGTTGCATAAGAGTGAAACCCATCTAGGTGCTATTGTTCAAAGTCACTATTTAAGGGCCTGAAAAAGCGCAGATACATCAATGTTTTTAACTAGAAATAATTGATATATATAATATTTTAACAACTACTCTCTTAGTCTTTCATCAGCTAAATGTAGTGGTTTTCTATCGCCAGTCACACATCCGAATTTAGCGTTCGATTTATTGTTGTAATTGCTTGTTGAAAAGGGCATTTAGACGTCGATAAATCGGACGTAAAGGATTGAACATGCCACTTTCTTATGATGATACTCAAAGACTTAGGGAGTTTTACAAAGTTTGCCTCGACAGCCAACATCAGATGTTGGGATATCCAGTTGCGACAGATTACGACTATCAGGACCTATCGTCTTTCTTCCAGTTTTCCATTAACAATGTTGGTGACTGGGCGGAAACGTCGAACTACCCAATGAATACCTTTCAGTTTGAGCAGGATGTTGTTGAGTACTTTTGTCAGCTGTTTCATACCAGCACAGAAAAAGCTTGGGGCTATGTCACCAATGGTGGCACTGAAGGCAATATGTACGGATGCTACTTGGCACGTGAACGCTTCCCTGACGGTGTAGTGTACTTCTCTAAGGATACTC

>NODE_31_length_3686_cov_1.920477

GGGTACTCCCAGTCTAAATCAATCTGAGAAAAGAAAGAGAAGCGCTTGAAGATCTCCACAACACTTGCGATAAAGATGGCGCGATTGTCCGGATCGGCAGCCATGCGTGAAAACGGCTCAGATAGAGACCAGCCTCCGACACTAAAGGCGACGGCTAAATCTGGATTCTGCCCCTTAAGCTTTTTCATTGCCCCTAAGAAGCCTGCAACTTTGTCTTCTCCCTGGTACCAACGCTGGAGCAAAACATCTTCCTCTTCCCAGTTTGGGTTACCTGCATCGGCAATTTCGTCATCCGTTAAGACACCGGCACCATCGGTTGCCATATCCGCCCAAGCATCGGTGGTCAGGACCTCAAAATCCTTACCATGCTCAGGGTTAGCATCTTTGGCAAAACCACAAAGCCTCTCTAGTGATTCAACCGCGGACGTATTCGAGCTATCACCTATTTCGGATCCACACATTCCAAAGAAGGAGAGTATTATCTTGTCGTAACCCTCTGAAAATTTGGATAGATCAACCCCACGAGCATAAAAGCCCCAGTTAGATAAATAAGCCGAGATTTTAGGCAGTTCAGAGTTATCTTCGTACTCATTGACATAAAAGGGGCTACCTGATGCAACAGGCCCTTTCGGTGCCAATGGATCTGTCCGTTCCGCCCACTCTTCCGGAGAGATGTATCTCTCATCTTCACGAGTAAAATCACCCGCAGTGGCAGTAGACGCTATGAGTACTAAAATTGACGTTGTTAATAAATTTAGCTTCATTTCTTAGATTCCAAATTGTTTTGTGCGCATACGCTTAAATGACACAAATTTAATAAAAACCAATTTCATATTGGCGAATTCAATTTAGAACGAAAGATATAAAAAATAGGCAGGAAGAGGTGAATCATTCTAAAAACTTGGATACATAAAATGAGACTGGCATTAATGCAGAGATTTTTTAATACCACTCTCCCACCTCCAGGAGAGAAGCTTCCTTTGCTCTCAGCGTCATACTCAAGCACAATAAACCTATGCATCTCCACCAACATGTCAGGCGGCGTCGTTAAAGCGCTATATTATTCACGCAGTTAAAGGCCAAAATTTATATGGGTTCAAACAGTAAACTGTTATGGGAACAACGCCACGATGATGAACATAAGATTCGTCGAGACGATCACCGTAGTCCTTACCAACGTGACCGAGCGAGAATTCTTCACTCCGCAGCGTTTCGACGTTTGCAGGCAAAAACTCAGGTTCACGGCAACAGTTTTGACGATTTTCACCGTACTCGGCTTACCCACTCTCTCGAAGCGGCTCAGTTAGGGACGGGAATCGTTGCGCAGATAAAGAAGAAACAGGAAGAATTTCGCGACCTCTTGCCTAGCGACAGCTTAATAGACTCGCTTTGCTTAGCCCATGATATTGGCCATCCACCCTATGGGCATGGTGGTGAAGTCGCCCTTAATTACATGATGCGTGAGCATGGCGGATTTGAGGGAAACGCACAGACATTTCGCATCGTGACCAAGCTTGAGCCATATACCGAACATTTTGGTATGAACCTGGCGCGACGAACTTTGCTTGGCCTGATCAAATACCCAGCGCTAATCAGTAAAACATGCGCTGATGTGACTCCAGACAGTGTCTCTCACCAGCGTAAGCTCAAAGCAAAAGAGTGGTCACCAGCCAAAGGTTTATATGACTGTGACCAGTCACTATTTGATTGGGTACTTGAACCGCTCAGTGAGTCAGATAAGCAACTTTTCAGCCAGATGCGCGATGAGCAATTAACTGAGCGTCAGCATAAAAAGACCCGCTTTAAGTCCATCGACTGCTCTATCATGGAGCTGGCCGATGACATTGCCTATGGGGTGCATGATCTCGAAGATGCGGTGGTGCTTGGCATGGTGACAAGACACCAGTGGATAGAAGGCGCGGCGAGTTTGCTTTCAGAATGTGGTGAGCCTTGGTTTGAAGCTCATATCGACTCTATTACAGACATGCTGTTTTCCGGTACTCACCACCAACGTAAAGATGCCATCGGTGGCATGGTGAACGCTTTACTCACCAGTATTTCCATCAAGCCCGTTGACGCGCCTTTCGAAAGTGAGCTGCTGGCGTTTAACGCTTATTTAGAGCCAGAAATGGCTCAAGCGTTGGAAGTCCTTAAGCGCTTTGTCAGTGAGTATGTGATTCAGGTACCTCATGTTCAAGTTGTAGAGTACAAGGGGCAACAGATCATTATGGATATCTTCGAAGCGTTTAGTGCTGATCCTGAAAGATTGTTGCCGGCTCAAATTCGTCATGAATGGCAGCAGGCAACATGTGAAAGCGAAGGTTATCGTGTGATTGCAGATTATATTTCTTCGATGACTGACGGCCATGCTCAGCGGCTGCACCAGCAGTTGTTCTCATCTCATTAACAGAAAGCGGAGCTTATGCTCCGCTTGCTTTTAACACCTGTCGACAAATTCATCGAGTCGGCTCATTAGACGTTGCTTTTCACTGTCACTGATAAAGCTCGCTTCAATCGCATTACGACTAAATTGTGCCAACTCTTCTTGTGTCAACATATGACTGTCTGCAACTGCAAGAAAGTTGTCGGTCATGTAGCCACCAAAATAGGCCGGGTCATCTGAGTTGATCGTCACACACAGCCCTTGCCTCAATAAATCGACAACATTGTGTTGCTTCATTTCCTCAAATACTTTGAGCTTAGTATTGGACAGTGGGCAAACTGTCAGCGGCATACGCGACTGAGCCAGTGACACCACCAGATTCGGATCATCTGCACAGCGCACGCCATGGTCAATGCGCGACACTTTCAATAATTCAAGGCTGTTATAAATGTTGGCAACAGGCCCCTCTTCTCCTGCATGGGCGACCGTGAGAAAACCAGCCTCGAGTGCTTTGGCAAAAACACGAGCAAACTTCTCTGGCGGGTGACCCATTTCCGATGAATCCAGACCAACACCAATGATTTTATCTTTGTGTGCCAGTGCTTGCTCTAAGGTGGCAAAAGCACTTTCCTCATCCAGATGACGTAAAAAGCACATGATGATCCGACTACTGATATTCAATTGCGTCTTCGCATCTCGCAAAGCGCGGTGAATACCGTTGATCACTGTGTCAAACGCGATACCACGCTCAGTATGAGTTTGTGGGTCGAAGAAGATCTCGGTGTGAATGACATTGTCAGCCTTACAGCGCAACAAATAAGCCCAGGTTAAATCATAAAAATCCTGTTCATGAATCAACACGTTGGCACCTTGATAGTAGATATCAAGGAACGATTGAAGGTTAGTAAACTCATACGCGGCTTTGACCTCTTCAGGCGTGGTAAATGGCAGCGCGATTTGATTGCGTAATGCGAGTTCAAACATCATCTCAGGTTCAAGTGAGCCTTCAATATGCAGATGCAGCTCAACTTTCGGTAACTGGCGAATAAATTCGGTTTGGTTCACGCTTGTCTCCCCATTCAATTCGATTGGTATTCAACAAACGCTTTTCTCTTGTGAAGATAATCAGGCATACAAAGAGAAAAGCGCAATGCACTTCACTCTTCGTAATCTGAAATTTGCGGTTTCAGGTAGAGACTCCCACACCCTATCAGTGGGATTATACGAAGCATCGTTTATGACGTAACAGCCATAACTAAACGATTGCGCGTCATTATATTCTGACAAAGATTCACTGTACAAC

>NODE_32_length_3623_cov_2.411450

GTAGTGAGGTTGTCGGCAATCGGAGAAATAAAGAAAGCCAGAATACCGGTTAACCAAAATAACGACTTGTAATTAAAGCCTTTCCCAACCATCCACGCTTGCAGTGCATCGAACAATCGCCGCTCTTCCATTGCGCTGATATAGGTCATAGCGACTAGTAAGAACAGCAATAATTCTGCGTATTCCAGCAGGTTGTGTTCTAGCGCTGACTTGGCAACTTCAATTTGACCATGTTGCGAATAGACGTAACCAATCATGGCCCAGATGAGGCCCGCCGCGAGCAGGACAGGTTTGGACTTTCTCAGCGACAGGTACTCTTCCAGCATCACCAACGCATACGCACAGGTGAAAATCAGAATGGCCACATAGCCGATGGTAGATTGAGTTAAAGGTAAGGTTTCTTGGGGGCTGTTGGCTGCAAAAGTCGAAAAGGGAAGTAATAAAAGGGCGACAAAAGCGGACCACTGCAATTTCATACTTTCACTCCTTGAAAGAAGATTGTTGTTATGGTGGAGCCATTCTAAAGAGGTTTTGGCGATGGGTATGTGAGATAGGTTTGATCTGATTGAATTGTCGCCAGTTTTTATCAATAAATGAACGATTTATGAATCATGTTGATATGCATTGTTGAATTTCATGTGCAGATAAAAGCGCTGATTATGAACATAATCACAAAATAAGATAATAGATCTCTGGTCAGAGTTGCCCCTTCTAAGTAATTGATACGGATCATCACTATAGCGTACGATTTAAAACAAGATCCTATGTGTCCTGTTCCACATAAAACATAGGTGATATATGTCCGATCCAAACATAAAGCAGCGCAAAGTAACCTGGGGATGCTACGTTGCTCTCGCATTTGCTGTCATATTCTTTTCTGGCTTGATGAAATCCGGTGAATGGTATGGTGTGCTTGATTTTACAACTCTCAATGGCTCGTTCGGGCAGGTTGCGTATAACGTGACTGAAACGGCGGATGGCGTTGAAGCTGCAACAACGTCACTGCGTGGTAAAGGCGGTAGTGGTGCACGTGATGGCTTTATCTTCGCATTAACTTTGATTCCAACCGTCATGTTCGCACTGGGTGTGATCAATGTCCTTGAGCATTACGGTGCACTAGATGCTGCTCGTAAATTGCTTACTCCTTTATTACGACCTTTGATGGGAATACCGGGTAGCTCCGGCCTTGCATTGATTGCGTCACTGCAAAGCACTGATGCCGGAGCTGCCATGACGCGCCAACTCAAGGATGAAGGTCGTCTGACCAAACGTGAAACCGATGTATTTACCATGTTTCAGTTCACTGCGGGGGCGACTATTGTTAATTTCTTCTCTTCTGGAGCTGTGTTGTTCACGCTGACTTTGGCTGATGGGTCGCTGGCCGTGACATCGTCAATTGGTCTCGCTGTAGCCATTATGTTTGTCTTTAAGTTTGTTGGCGCCAATCTGTTTCGCATCTACCTCAACATCACTGAAGGTAAACAAGATAAAGATACGCAACGTTTACAACAGGAGGCTGCACAATGAGTGAAGTGAAAACGAGAAAACCCATGGTGACGGATATTTTTGTCGAAGGAGCAAAGAAAGGTTGGGTGATTGCAACGACTTCAACGGTGCCAAATGTTCTGATGGCGTTTGTAATCATTAAAGCACTGCAAATCACAGGAGCGTTAGACATGATGGGGACGGTCTTTGCACCCGTTATGGCGATATTTGGTTTGCCCGGTGAAGCTGCTGCCGTATTGATTGGAGCGTGGATGTCGATGGGAGGTGCTGTTGGTGTAGTCATAACCTTGTTTGATCAGGGCATTCTTAACGGCGCACACATTGCGATTTTGGCCCCAGCGATCTACCTGATGGGTTCACAAGTCCAGTATATGGGACGTATTATGGGGCCAATTGGTACCGAAGGCCGCTATATTCCAGTCATGATCATTATTTCAGTGCTTAATGCATTTGGTGCGATGCTGGTTATGAATTTATTCGTTTGAGTGTACTGAACAAATTAAAGTATCTAAGCCAAAGTGTTATGACGCTTTGGCTTTTTCATTTCAGTAATATCATCCAAGTTAGTCAGATCGCGTAATCATTGAATTAAGAAAGTCACGTCGATCTTTAGGGATGACAGCCCAATGACAATTTTGAATTGCGCCTTCTAAATGTAAGACGAGCTCTTTCGATATTTTTGGAAATCGATCTTTGAGTTTTTTATAAGAGTTTACCGAGCCCCTATTTGCTAGCTGATGTACGCTTTCAATGTTGATGCTTAGCAGCATGCGCTCAGTAGACACAGAGATATTGGGTAAATCTTTTAGCCTTTCTATGGGCGCTTTATCTGCGTATTCCCTTTGGCGGTTTGCCGCATTAATGGAACGTTCCGCATGTTCAAGTAGTCGTTTCTCATCTCGCTCATATTCAGAGATGAAGTAGTTTGTTTTTACAAGGTGCCCTCGTTTTCGATAGCAGTAACGATTTTTACTGATTGGCTCGGCACCAAAGTCGGGTGAAGAACGAAAGTAAATTCGATTCTTATAATGTAATGCGAATATTACACCATCACAGAATATGCCAAACGTACCAAACATTGATTTAATCGTAATGGTTCCTAAACTGCTAAAAAATAGTTGTGTATCTTTAATATTCATATCAAGTTAGCGTTTTTGTTTATCACATCATAAGCCTAGACATCTTGATAACTATAAATGAGATAATCCTTTATATTTTTGGATATAAGGTTTGTTTAGTTGCATACGGATGTGGTTGAATTGTGACAACTATATTTGAATTATCAATAACATGACTAAGCATGTCTTTCTTATCTCTCTGATTAAGTTTACTAATGATTTTACAATGTAATTATTATTAAATGTAAGTTGTTGAAACTTAATGATAATCCTATTTTTATATAAATTATCCTTAAATATGTTCAACTCAATGTGCTTGATATGTTAAGTATTTCTTAGTGTTTGCTGTATCAAAAAATATAAATAATAATTAAAGGAGAAATAGCCATGGATACTAGGCTAAAACTAAGACATGACACTAGAATCACGTTAACAATAGGAAATGGAATTCTTAAAGTTTCACTAAATGAGGAAGTGATAAAGTCGACAAACCTATCCAATATTGAAGATAGGATCTTGCATATTCTTATTCATAAAAGTAATGGGTCTAATCCGGTATCGAATCGAGAGCTTGATAAAATATGTAGAGAAGATTATAAGCTAAATATACAATTCAACACCATAAAAAATAATCTGGCTTCTTTAAGAAAAAAAATAAGAGAACTCTTATCAACCCCCCAGAATATTGAATTATCTACATGCACTTTAGTTGATAATAGACATGGTAAAGGCTACTTAATATCAGACTACTGGATACTTGAACGAGATGAAGAGGTCATAAACTTTTGTAAAGAAAAAGATATTGAAGGAATCTCAGATCTGTATCTAAGCCTTATAAAAACGTCAATATATAAAAGTAAAAAATTGGTATTTATTTTAAGTGGATTATCGATATCGTTAGTAATGTCTCTAGCTTTTATACAAATATCTTATGTAATAGGTAATTATGAATTTTATAGCTCTAATACAACAACGATCACGCGCGATATATTAAGAACC

>NODE_33_length_3613_cov_2.203054

GTTATGGATTTTCCACCTGGTCACTATATTTGGAGCAAAGATGGTGATACTCCACAACAATACAATCATTACTCTTGGAAAGAGTATGACTCAGTCAAAAATAACGTTTCCAACTTGAAAGTTATTCACGACTCATTATGTACATCTATCGAAAAGCACATGATGTGTGATGTTCCATTCGGCCTACTATTATCTGGAGGGCTAGATTCTTCAATCATTGCATCTGTCGCTCAGAAAATAAGGCGTTCGAAGGGTCTTAATCCTTTGCATTCATTCGCTGTTGGTCTAAAAAACTCTCCTGACTTAAAAGCAGCTAGAGAGGTGGCAAATAGTATTGGAACCATTCACCATGAAGTGATTTACAATATTGAAGATGGCATTAATGCAATTAAAGATGTTATTTACCATCTAGAAACTTACGATATAACTACTATTCGAGCTGCAACACCCATGTACTTAATGGCTAGGCAGATTAAGGCTATGGGATTGAAGATGGTTTTATCTGGAGAAGGAAGTGATGAGCTCTTCGGTGGTTACTTGTACTTCCACAAGGCTCCAAATGCTAAAGAATTTCATGATGAAACCGTTCTTAAACTAGAACAACTTCATAAATACGATTGCCTTAGATCAAACAAAGCTATGGCAGCCTGGGGAGTTGAGAGCCGCGTTCCTTTCTTAGATAAAGAGTTTGTGGAATGTGCGATGACTCTAAACCCCAATTCAAAGATGTGTGGTAACGGTGTGATTGAAAAGAAAGTTCTTCGTGAAGCCTTCATCGACTACTTGCCTGATGAGATTCTATGGCGACAAAAAGAACAATTTTCTGATGGGGTTGGATATTCTTGGATTGATAAAATCCATTCTTATGCTGAAACTAAAGTCTCCACACCAGAGATGGAAAGAGCTAGTTTAAAGTACCCAACAAACACACCGTTAACGAAAGAAGGTTATTTATACCGGAGTATCTTTGAAAGCTTTTTCGGAGGCAGCTCAGCAGTTCTTTGTGTTCCTCATGGCAAAACAATAGCTTGTTCGACTCCTAATGCTATCGCTTGGGATGAATCCTTCCAGTCTTGTGCAGATCCGTCAGGCCGTGCAGTGAAGAATATTCATCGCAATAGTGAATCAGCCTAAGTAATCTGATTTGGGCATCTGAACTTGATGCCCAAATTATCACTTCTGTGCCTTACTGATAGCTTAACTGGTTACCTTTCTCGAAGCGATACAGCACTGCTAGCCCCGGTAATGTCGCGATCGCAACATAAACACCCACATAGGCGGAGCTAAGCAGCATGGCTTCTGTCACGATCAGTGGTGTTAGGCCACCAAAGATAGACAATGCAATGTTATAACCAATACCTAAGCTGGTGGTTTGGTTAGCAGATTGCTGCCATAACAGCGCGGCCAAGTTACCTAAAATAATCGCTGCCAACGTACTGATACCCAACACTGCTAACGCTTGCCAGATGAATTCGCTGGAACCAAGTAGCGCAAACAAGGGGACCGCTGCAAACAGCATAAACAGCGCTCCGACTTTAAATGCTTGTTGTGGTGAGGAACTTTTGTCGACGACAAACGAAAATACGAACAGCAATACCACCAGTACTGACGAGTTGATCAGTGCGAGGTTCTCAATCCCCATCGCTTTGCCCAAGATACCGCTCGCTAGGTTCTGAACATAGAACACCACCGCACCAAGAATGGAGATACAAAACACTTTGGCAATGATCTGACGGCTCGACTTACTTGCTACCGCTTCTGCTTTCGGCAATTCAGGTAGAGAAAGACGCAAATATAAGCTTACGATAATGTTCGCGACACCCACTAGCAGCGGCACTCGCCAGCCCCAAGCACTCATTTCTTCTGCCGTTAAGTAATGCTCTAAGCCTGCAACGATAAGGAGAGAAATGATGACACCGACAATAGAACTCGCAACAATCAAGCTGCTCACACGTGAGCGTTCTGATTTCTTCGCATCGTAGAGAAGGTAATTGATTAGGGTTGGGTATTCGCCCCCAAAGCTAAACGCTTGAATCATTTGGAAGAACAAGAACACAACCACCACGTACTGTCCGACCGTGTCGACTGGTACAAATGCCATCGATACCGTAGCTAAGCCAGTCAGAAGGTTACAAAAAACCAGTGCTCGTTTACGCCCGTATTTGTCGGCGTAGCGGCCAATCACAACGCCGCCAAAAGGTCGGATCAAAAATCTCAGGGCAAATACGCCCCACACCACCAGCTCTGAGTGCTCAATGCCTTGGGTGGCAAATAGCTGGGAAATGTACATAGATATCGATGCGAATACAGCGATATCATAAAACTCCATGGCATTACCAAGGACAGCACTGGTGCGCTGTTTCCAGGTGAGTGACATTCTGTTTTACTCCTCTATAAGTTCTAATTGATTTCCATCGGGGTCTGAACAGAAAAAGTAACGGAAACTACCTAGCCTAGCGTTGTAAATAGTAGTGATGTTGGTGACTTCCGATAATTTTCTATGAAAGGCTTCAAGATCATTCACACGTAAAGCGAAATGGGTGATGCCCACTCTTTTTAACGCGATCAATGATTCAGAAACTGGGGCTGAAACGTCCCCATAAAAATGAAAAAGCTCGATTCTCGAGCCTTGGTTTTCCAACATATGAATGGTGACCTGCTCATCCCGATAACTTCGTTCAAGCTGGTAACCAAACAATGCATAGAAATTTTGGCTGGCGACTAAATCGCTGACCGTTAGCGATACGTGATGAAACTGTCCAGACATGCGACTGTCACTCCATGTTCCACCAGTTTTCTAAAGAAGACCGAATCAGGCTGAGGTTCACAGCCCAGTTTGCATTTGATGATATAGAGTCGCCGCGCTTCTTCCGCCTCTTGGCAACCTCGAAGCAGAGCCACCTCTTGAAAGGTTTTCATCTCAAGAAAGTAACGCACACTGGTGAGCTCATCGTGTGTCAGCGTCAGATTGCCGAACACATAAGGTTTCTCTTCGTCATAATTCGACTGAACTGAATTGGCGGCTGACAAGCTATCAATAGAGATTTGTATCTCTTTATCACTCCAGCATTCAAGCGCAAGGTGATTGACCTTAGGACGGAACGCAATCGCCTGACTGACCACATCGCAAGGTAACGGCGTGCTTGGCAAAGAGATGGAGAGGATGTCGTACTGTGAGCCCTGATTGGTCACGATATCGACGCGGTTATGGCTCGCTTTCGCTGACTCACGGAAATTGTGGGCCCCTTGACTGTTCCACAGGTTAATGCCCGGCTTGAGACGCGAATGGAGGTTTTTATACAGCTCGTCGCCCCAATAAGCCAGATGCCATGAATAGTTAGTCGACACGGTTTTTACTTGCTCAAGTTGTGTATCCACTCGAGAGAAAGACACCGTCTGATCGACTCCCATGTACTCAACATAGCGTTTGAATACATGGCACATCGCTTCCATGTCAGCGTTACGTACATCCATGACTTGTTAGTATTATCAGCAAAAAACAATATGCGTATCATACCTTATGCATTTTGTTTTACTACAAAAAGTAAGGCCGAATTAAACGGGTTATCAATAATTGAGGTAAAAGCAGAAAATCCCCTTTAAGTGCCACAAA

>NODE_34_length_3609_cov_2.489807

GATTGAACCAGTAGTTTTGACTGCCAACGACAGCAACCAGTTCGAAGTTCATGCCGCGACGTTTTTCCAGCTCGGCTTTTTGCTCAGCAAAAAGTTCAAGCCAGCTTGAGCCAATATTACCTTTGCCACACAAAGCGATACCAACGCGCTTTTGAGCCTGGAACAGTTGGCTGTGGATCGATTTGACCAGAACAGAAGTATCTGTACTGCGCAACACTGCGACCAAGCTTAATCCTGACTGAGATTCTGAAATAAACTCCACAGGAGAAGACTTAAGCTTCTGGTAGAAACCGTAGCAGTGGTTGGCATTTTTAGTTACACCTGCACCGACTGCTGCAATCAACGAATAGCCCTCTTTGAGCTTAATTTCAGCTTCTATCGCGGCGTCTTGGAGGTATTCGAGTGCGCCGCCTGCAATCTCAGCGGTATACGCTAAGCGCAGCGTTTGTTGGTCTTCCTGTGTTTCATAGGTCAGTGGCTCAAGTTGAGCGCGTTTCAGCCCTGCAAGTGTTTCTTGTTCAAGGCGACTGAAATCGTGGCCAGAGGCAAAGTTGAGTTGGATTAGCAGCACTTCTTTCAATGAAGAAATGATTTTAGCGCCGCGACCTGAGGCCAGCACGCGTTCAATGCGTGTGGAGCCATCTTCTGGCTCATAGCTGCAACGCAGGTGTAAATCCATCGCACTTTGTGCGACCGGCTGAAGGGTGCGGCTGTGCAGGACTGGTGCCGCAAGTCGGGCTAATTCGCTGGCTTCGTCTAGGCGAAGCAATGGCAAAAGACACGCATCAGAGACTAAACGCGGGTCTGCGCTGTAGACACCCGCAACGTCACTCCAGATGGTCACGCGTTTTACTTCGGCTAGCGCACCAATGACGGTGGCGGAGTAATCTGAACCATTACGACCAAGCAGAACGGTATGGCCTTGCTCATCCTGTGCCATAAATCCTGTGATCACAACACGGTGATGGGCGTGTTGAGCGAGCACTTCTTTGATCAGTGGATAGGAGGTGCCCCGGTCAACTTCAGGTTGTGTGCCTGCTTCGGCACGTAGGAAAGCTCTTGCATCCTGAGCGACAGCCTGTAGATCATTTTGATTCAGTAATGCCGCCAGCAATCGAGAAGACCAAACCTCTCCGTGACCTAAAACGGCTGCGCGCTCGGCCGTCGTTAAGGGAGCCGTTAGCTCACCCAGTGTGCTGAATTCGTCGTTGAGTGTGGCAAGAAGCTGCTGTTGGCTTTCACCTTGTAACAGGTCTTCGACCAGCTGTACCTGAAACTGACGTAATGACTGCAAAGCTTCGTGGGCGAGGCGGCCATCTTTGTTCAGTGAGTCTAAAAACTCAATCAAACGATTGGTGGTCTTGCCTGCCGCTGAGACAACAATTAAATCTTCTTGATTAGAATATTCTTTGAGAATACTAACTACGCGGCGGTAACACTCTGGATCCGCGAGGCTACTGCCGCCAAATTTATGCAATTGACGCTGCTGGCTCATGACAACGCCTCCTGAGTTTTACTGAATGCTTGATCTAGATCGGCTATCAGGTCATCAGCGTCTTCGAGGCCGACAGAAAGGCGGAGAAGCTGTTGTGAGACTCCGGCTTCTGCTAATGCGGCTTCACCCATTGCTCTATGTGTCATTGACGCTGGGTGGCAAATCAGGCTCTCCACGCCGCCAAGTGATTCGGCGAGTGAAAACAGTTCTAGCTCACCAACAAAGTGTTTGAGCTGTTCAAAGCTGCCTGCAAACTCAAAGCTGAGCATAGAGCCGAATCCAGACTGCTGCTTTTTCGCAATCTCATGGCCCGGGTGTTCAGGCAAGCTTGGGTGATAGATGTTGCCAACCAGAGACTGAGATTGCAGGTAGTTGAGAATGTGTGCTGAGCTTTCTTCATGCACTTTCATGCGCGCACCCAAGGTGCGAATACCGCGTAATGTCATGTAGCTGTCGAATGGTGTGCCGGTAGCGCCGATACAGTTACCCCACCAAGCAAGATCTTCAGCATGCTGCTCAGTTTTGGTGATTACCACGCCACCTATGACATCAGAATGACCGTTAATGTACTTGGTTGTCGAGTGAATGACGAAGTCAGCGCCGAGTTCAAGAGGCTTTTGGAACACAGGTGTCAAAAAGGTATTATCGACGCCGACCAGAGCACCAACCTGGCGGGCTTTTTCACATACTGCAGCAATGTCGACTACTCGAACCAAAGGATTAGACGGTGTTTCCAACAGAATCAGTTTAGGTTTCTTTGCTATAGCGTCGTTCAGGGCTTTTTGATCTGATTGATCGACAAACTGAACCTTGAAGTCACCTTTCTGAGCGCGGGTGTTAAATAAACGGTACGTACCGCCGTAGCAATCGTGTGGCGCGATGATCAGGTCATCTGGGCCAAGAAATGCAGAGACCCATAAGTTTAACGCTGAGGTTCCACAGTTGGTCACTACCGCACCTTTACCGGACTCCAGCTCATACAGAGCTTTTTCTAATAAGCCACGGTTTGGGTTACCCGAGCGAGTGTAGTCATATTTTGGCACTTCGCCGAAGGCGGGAAATCCGTAGTTAGTCGAGAGGTAAATAGGTGGCACAACGGCGTGATGCTGAGTGTCTGACTCGATACCAGTACGTACCGCGATTGTGGCTGGTTTACGGGCGCTCATAAGTGTTTCCTTTCGAACTGTAGTGACTTGGCTGGAGATTACACTATACCTGTTTGCCGGATGTTAGATATATGTTATTCCAATCGTATATCTCTTCACTTTACTTCTCATTTTTTAAGACGTCAACACTTCTAGACGTCTATATGTCTTTGCTTGTGGCCATAAATCCCGCTAAAATTACGCTTTCTAATTTTATACATAATGAATCATAAAGGTGCGCAATGGCAGATTGGAACGGCGAATACATTAGTCCGTATGCTGAACATGGAAAGAAAAGCGAGCAAGTAAAGAAGATCACGGTTTCTATTCCATTGAAAGTATTGAAGGTTTTAACTGATGAGCGAACGCGTCGTCAGATTAATAACCTGCGCCATGCGACGAACAGTGAGCTGCTTTGTGAAGCGTTTCTGCACGCTTATACAGGCCAGCCATTACCTACCGATGAAGATTTGCGTAAAGACCGCCCAGATGATATTCCTGCGGAAGTGAAAAAACTCATGACTGAGATGGGCATTGAATTCGAAGCGTATGATGAAGAGTAACAACTGGCAGCGCGCCTGGTTTTTATTTTGCTGAAAACTAAAAAACCGCTGAATGTTCAGCGGTTTTTTAGTTGCCGAGAGGAACGCTTACGCTTCCATGTAATTCTCTGGCATTTCAATGCGAGCCACACCTGACTCTACTGCTGCTAGAGCAACTGCTTTTGCAACACGTGGCAATAGGCGAGAGTCCATCGGTTTAGGAATGATGTAACCAGGACCAAATTCCAGCTTATCCACGCCAGCGGCTTTCAATACTTCCGCTGGAACTTCTTCTTTCGCCAGTTCACGAATGGCATCTACCGCAGCGAGTTTCATTTCATCGTTGATTTCGCTAGCACGAACGTCAAGAGCACCACGGAAGATGAATGGGAAACACAGTACGTTGTTTACTTGGTTCGGGTAGTCG

>NODE_35_length_3607_cov_2.207365

GCCATCGCCAATTTTGACTCCTGCCATGATCATGGCCTCAGAGCCAATCCACACGTCATTGCCGATCACAGTGTCACCTGCCGGACGAAAGCCATCTTTTGCTTGAGCAAAATTTGAATCGTCCTGATAAAAGAAAGGAAAAGTACTTGCCCAGTCGGTACGATGCCCTTGATTGCCAGCCATCATAAACACTGCACCGGAGCCGATAGAGCAAAAGCTGCCAATGATCAGTTTATCGACATCGTCACGCTCAGCAGAAAGATATCGAGCACAGTCATCGAAACTGTGCTGATGGTAGTAGCCTGAATAGTAACTGTGTCTGCCAACGACAATGTTAGGGTTAGTAATTTGTTCAGCAATCGGTTTACCCGCGAAAGGCGAGTCAAAATAGTTTTGCATTATGGTGGTACCTGATTGTTTTTCGGACAATTTACACCAACACTCTATTGCCCCCAAGCATTCTTTCTCGTCTGAACCAGATATAACGCAACGCTGACAAGACATAGACCAAGCATCACCCAATAAGCGCCCGCTAGGCCAAGCCATTTCGCTGACAGGCCTGCACTCAAATATGTCACCAACCACCAAAAATGAGTCAACGAGAAATGCGCGGCAAAATATTCACCACTCTCTTTACCTTCGACTACGGCATTAATAATGAGCCCAGATGTCGTTTGAATGCATGACATTCCGATACCCAGCGCAAAGCACATCAAGCCAAAACCAATCCAGCCTGGTGTCCAGGTTCCGGCCAAAAACGCCAGAGCAATAAAGACTAAACCGCGTAAATGGTAGCGCTGTGGTTGATGATGTTTAAGGAGTCGAGGAAGAAAAATCGCTACCAGCATGGACCCTAATCCAACCACTAACATGGCCATCGCCGTTTGGGTCTCGCTCCCATGCAGAATGTCGTGTACATAGACCACAGTATTCACCAAAACCATGGCACTAGCAGACGCAGCTGCCAAATAGGCATACCAAAGTGCACGCAGACTCGGCTGCTCTAAATACGCTGAAATTCCACTTAACACTGAGCGAAAAGAGAGTTTTTTTGGCTGAACATTCAGTTCTTTCCTTTTGGGTAAGGTACATAGAATGATCAGTACGCCAGACAATAAGAAAGTCATCGAATCAAGCATAAACAGCTGACGAAAACTGATCACACTCAGCAACAATGCAGTGAGCATTGGGCTAAAAATCTGTTCTAAATCATAAGCTAATCGGCTGTATGAGAGCGCTCGAGCATAATCATCTTTATCCGGTAAAACCTGTGGCAAAGTGGACTGAAACATTGGTGTGAACCCTGCCGAACATGCATTGATAACAAACATCAACACGTACACTTCCCACACTTCAGTGACGAAAGGGAGGCAAGCAAACATAAGGGCACGGATGATATCCAGAGCGACCAGCGTCTGGCGCTTGGGCAACTTGTGAGCCAAAACACCAAATACTGGTGCTAAGCCAATATAGGCCAGCATCTTGAGGGCGAAAGCAATGCTCAGTACTGCTCCTGCACTTTCTTCCGCTAAATCATAAGCGAGTAAAGCTAAGCATACCGAGCTCACTCCTGTGCCAATCAGGGATATCACCTGCGCAGACAGTAAGCGAACATAATTCACGTTCTGCCACAAGGTAGAAGGCTTCAAAGCAACGGTCATTGCTCTACCTCCCAAAACGCTTTAATGCCTGACACCAAAGCACGTAGCCAGAGTTGGAGGGTATCCATCAGCCGACTGGTAAATGAGCCTTGGTCCACCCCGACCTGACTTACGGCGGGAGCAGTAAACACTTGCTCACCGTTGAGACTCCAGATGACCTGCCCCAGAGAGTCTCCCGCTGCAATTGGTGCTTGTAACTGGGATTCGACGACCACCTCCTGTTTGAGCGCATCTTTCATGGCTCGTGGAAAGGTCAGATACACATTTTCTGCCACCGTTACAGGGACGTTTTCCTCAGCGCCCTTCCACACTCGGCTTTGCATAATGACTTCGTCTTTTTTCGCCACTTGGACGGTATCAAAAAAGCGAAAGCCATAACCAAGTAAGTTACGTGAAGCGCTGGTTCTCGCCTGCTGACTTGGTGTACCCATCACCACAGAAATTAAACGCATCCGCCCCTCTGTCGCAGACGAGACAAGGCTGTACCCCGCGTTCTCGGTATAACCTGTTTTACCACCATCGACATTCAGTGAGCGATCCCATAACAGCTTATTTCGGTTGTACTGTTCGATGCCATTCCAAGTAAATTTCTTCTCCTGATATAGCTTATAGACCTCAGGCACGTCCTTGATGATCGCGCGCATCAGGTTCGCCATATCCTTTGGTGTGGTTTGAATATTGTCGCCATCTAAGCCATGCGCATTGATAAACTGTGTGTTTTGCATACCAAGCGTCTGCGTCCAACCATTCATCAAGATTACAAAAGCACTTTCACTGCCTGCTACGTGCTCAGCTAGCGCAACCGAAGCGTCGTTTCCCGATTGAACAATCACGCCCAACATCAGGTTACGAATGCTGATTTCGTCACCCGGTTTGATGAACATTTTGGATGACTCCGGAAAATTCACCGACCATGCATTACGGCTGATTGTCGCTTTGTCATCCCAGCTAAGGCGACCAGCGTTTATCTCTTGTCCAACAACGTACGCCGTCATAAGTTTGGTTAAACTCGCAGGAGCCAAAGGCGTATCTGGGTTTTGTGACGCAACTACCTGACCGGAGTGGTAATCCATTACGACATAACCTTTAGCATTAATCTGTGGTGCATCAGGTGTAATAACAGCCCCTGCGGAGACCGTGACCAGAGTACAGAGCAAGGCACAAAGTTGAGTCCATTTGATCATTTTGCCACCTCACTACTCAGCGCCGCGAGATCACCGATATATTGTTGTCTGTCTCCTTTGCCATTTGAAATGTACTGGTGAGTTTCACTGCCTTTTCCACACACCACGATCAAATCACCGGCCACACTCAATTGCTTCGCAAATTCAATCGCTTCAGCTCTGTCACTGATTTCAAAATGCGGAACTTTGGACACTTGCGATGTGAGTGCTCGAAATATGTCTTCCTGATCGACACCCATGGGGTTATCTGAGGTAAAAACTGCCGTATCTGCCAGTTGCGAGCACAGAACGCCAATGCCACTCGCGTCCTGAATCCGATCTCCCGTTACTCCGGTCACTACGATTAAACGCCCCTTGGTATAAGGTCGAACGTCTTCCAGCAGCTTACTGAGTCCATCAGCATTGTGTGCATAGTCCACCAATCCGGTCGCTCCATTGTTAAGCGACAAGAACTGAGCACGGCCATCGGGCGCTACTATGGTTTGCAATGCGGCCGCTATCCGACTAGATTGCCAACCGAGTGAAAGTAAACTCGCTGCAATCGCCAAAGCATTTTCGACATTGAACGTGAAAGGCATTTGAGAGTTCACATCAAAACACTCGCCGTCAATTCGGAGTTCAAAACACGAGCCAAAACGAATAGTACGTACATTGGTGGCGAAAACATGCGCTTGAGCATTCTCAATACCAAAAGTCACCACGTTCACTCGCCCACTCGCTCGCTCAATCGCCGCTGCTGCATGCGGGTCATCAAGGTTGATC

>NODE_36_length_3559_cov_2.466111

CGTTAACGATTCTTGATGAAGGGCATATCAAGCCTGAGGACATGAAGGGCTCATGGGCAGGGGCGATGGGCCAGTGCCAGTTTATGCCGAGCTCTTTTCTTTCTTATGCCGCGGATGGCAGCGGTGACGGCAAGAAAGACATCTGGGATAACGAAGCCGATGTATTTGCTTCCGCAGCCAATTACTTGAGCCAGTCTGGCTGGACCGATTCCTATACTTGGGGGCGTCAGGTTAAGCTGCCACACGGTTTTGATACCAGCATTCAGGGTCGCAGTGAAGAGAAAGGGAAATACCTTCAGGAATGGAGCAAAATGGGGGTCACTCGCTATGACGGACGCCCACTGCCTCAATTGGATGAAGATATTAAAGCTTGGTTGATTGCCCCTGATGACGCCAATGGCCGCGTTTATCTGGTATACAACAACTATAATGTGTTGATGAAGTGGAATCGCTCTTATTATTTCGCTTTAGCCGTCAGCCATCTGGCCGATAGAATCGCACTTTAATTGTTTTGATTAGGAGCTCTTCGGAGCTCCTTTTAGTTTGTGGAGTCATTGTGCTGTCTGATCGCGCGTCTCAAATGGTGGTGTTTGCCGCCTTACTTACTCATAAAAACTTTACTCAGGCCGCCAAGAGTCTGGGTGTCTCTGTTTCACATGTCAGTAAGCAACTGGCTCTGTTGGAAGAGTCGCTAGGGGTTAAGTTAGTCCAACGAACCACAAGGACTTTTACGCCGACTGAGGCAGGAGAACAGTTTTTCTATCATTGCCAAAAAGTAGTGCGAAGTGTCGAAGAAGCGCATCTGGAAGTTGAAAGCCAAAAAGATGAAGTAGCGGGCTTAATCCGAATTGGTTTGTCGCAGTCGTTTGGTACCTTACATATTCTTCCTGCGATTGATGAGTTGCGGTTGCAGTACCCTAATCTACAAGTAGAGATGCATTTGTTTGACTACAAGGTCGACATGTTGCAAGAGGGGCTTGATCTCTGGATTACTAACAACGAGATGCTGCCGGAAGGATACGTCGCACAGCGGTTAGCGGACAGCCAGTTCGTAGTCGCGGCTTCGCCAGAGTATCTGATGAAATATGACACCCCTCATCACCCGAATGATTTGGTTGATCATAATTGTTTGATTTATCGAAGTTGGGAGCGCGATTATACCAGTTGGGCATTTTCCAATCGTGACGATCAAATAAATGTAAAGGTGTCTGGAAATTACTCGGTCGATCTGGCCGAAGCAGTTAGAGATGCGGCTGTCGCTGGATGGGGAGTCGCTTACCTTGCCACTTATCTGATTCGTGACGAGTTTAAACAAGGTCAGCTGATTCAGCTTTTACCTGAGTGGTTACCTAGTCAGAACATGCCATTTTATGCGGTTTACCCTAGCCGTCGTTACCTGCCGACTAAGACAACAGCAGTAATTGAGTTCATCAAGAAGAAAATTGGTTCACCTTGCTACTGGGACAAACGATTGTCTCCCTATATCCACCGACCTAAAGCCTGATTTTCGCCCTGAGATTATTTCTGTTTTTGGAAACATTTTTTCTTTATGGAAATAATAAGAAAAAGCAAACGTTTATCTTGATTTAAAACAGCAGCTTAATGCGTTGATTAAAGTTTGAGTTGTGGAAAGTAAACCGGATTTTAACAACTTGGATTTGCATCTCATTTGTAACGGAATATTATCCGACGCCATTCTTAATGCCCCTTTTAGTCGGATATTTCCATGAACTCGATCCTCGGAATCATCGCCATTCTTGCACTGGCATGGCTACTTTCTACCAACAGAAAAAATATTAACCTGAAAACGGTATCTTTAGCATTTGCTCTGCAGATTACCTTTGCGCTGTTAGTCCTCTATGTACCGGCAGGTAAGGAAGTGCTGAACAATGTCACCGGAGCGGTGTCGAATCTGATCAACTATGGTCAGGAAGGGATTGCCTTTTTGTTTGGTGGGTTGGCAACCGGTGGCTTTACCTTTGCCATCAACGTGTTGGGTATTATCGTCTTCTTCTCTGCGTTAATCTCCGGTCTGTATCATATTGGGCTAATGCCGAAAGTGATCAATGTGATCGGCGGCGGATTACAAAAACTGCTGGGAATAGGCCGTGCAGAATCTCTGTCTGCGACGGCAAACATTTTCGTCGGTATGATTGAAGCGCCTCTAGTAGTAAAGCCTTATCTGCGCCAGATGACCGATTCACAGTTTTTTGCTGTGATGACAGGCGGTCTTGCATCTGTGGCTGGTGGTACACTGGTCGGATACGCGTCGCTGGGCGTGGACCTCAACTTCCTTATCGCGGCAGCATTTATGTCAGCCCCTGCAGGTTTGCTGATGGCAAAAATCTTGGTTCCTGAAACGGAAAAAATAGACGCTCAGGTTGAAATGGACAATGTCGATATGCCACGCGCGACCAATGTTGTAGAGGCAATGGCGGATGGTGCGATGTCCGGCTTACGAATTGCTGTAGCCGTTGGTGCCACCTTGCTAGCATTCATCAGTGTGATTGCCATGCTTAACGGTATGCTGAGCTGGATTGGTGGTCTGGTAGATATGGAGCTAAGTTTTGAGCTCATCTTAGGCTACTTGTTTGCACCTGTTGCTTGGCTGCTGGGTGTGCCTTGGAGTGAAGCGATTACGGCAGGCTCTCTGATTGGCAACAAAGTGGTGGTTAATGAATTTGTTGCCTTCATTCAGCTAATGGAAGTGAAGTCTCAGCTTAGTGAGCATTCACAGGCGATTGTGACGTTCGCTTTGTGTGGTTTCGCGAATATTTCGACAATGGCAATGCTGATTGGTGGACTGGGTAGCTTGGTACCTGAGCGTCGCAGCTTTATTTCCCAGTATGGGTTCCGTGCCATTGCAGCAGGGGTAATGGCCAACTTAATGAGTGCCTCAATCGCTGGTGTGATACTCAGCCTCTAAAACGAAAAAGCCGCTTTATGATAAAGCGGCTTTTTTATGTCAGCTTTTCAAGATCAGTAATGTTGTCGATCGCTTGCTGGTTCGTACTACCACAGACGACAGGACAGGGTTTAAATTCGTGGCACACTTTCGGTCTTTCTGGTTTACCAAACAACTTACACAAATTATCTTCATTGAGCTGAATACATCTTGTCCCAGCCGGCTTACCTTTCGGCATACCCGGAATCGGGGAAGAAATACTTGGCGCGATACAACACGCTCCGCAGCCTAATCGGCATTCCATATTGACGGTCTCATCAAAAACAGGGGCGCGATAGTAGCAAAAAAGATTGCCTATAGCAGTATAAAGTTCATTCGACACATTACATGTTTACTTAATGTCATTCATGACTTGGTCGCCAATATAACTGTGGTGTGAGTAGAAGCAGTTCCGCTCTAATTTATATTTTTGTTAGTTTTGAGCGTGTTCCACTAGACTTAAAGGTTAGGGGAAACGCTTTTTAGGCGTTTTGAAATGAAAAATACAATCGGGAGTGTCTCGTCATATGAGCGATAAAAGCTTTTGGACAAAACATTCCAGTAAAGTCGCGACGGTCTTTGGCATAGCTGGCGCAGTTATGGTCGGTGTCGGCA

>NODE_37_length_3559_cov_2.285755

GGCGGGAATCAGTGGCATCGTCAACGGTGTGCTCTCACCAAAACGTAATGATCTTTTCTGGCGTCTGTTGTTTCTTATCGGCATGGTTTCGGGTGGGGCTCTAAGTGTAATGGTGCTTGGTATCGAAATACCAAGCACCGAATCTATTTCCACTTTTGTTCTGGTAATAGCAGGTTTGCTGGTTGGGTTTGGGACTCGACTAAGCAACGGTTGCACCAGCGGGCACGGCATATGTGGTGTTGGTCGATTCTCCTTTCGTTCAATCGTCGCGACAGCCATTTTTATGGTAGTTGCGGCTCTGACTGTCTATATTCGCTAAGGCAAACGCTATGGGTAACGCTACTCTTCGAATTTCTGCATTAGTAAGTGGTCTGCTTTTTGGTATTGGTATGGCGCTGTCAGGTATGACAAATCCAGAGAATGTGATTGGTTTTTTAGATGTTACTGGAGCATGGAATCCAAGCTTAGTATTTGTAATGGGGGGCGCGTTACTCATATTTGCTCCGGCCTATCACTTTTTGATAAAACCTCAAGCCAAAGCACTGTCCGGCGATGTGTTGTCTCTGCCTACGAGAGTGCAGCTGGACAGTAAGCTCATCTATGGGGCAGCTTTGTTTGGAGTTGGCTGGGGACTCGCAGGTATATGCCCAGGCCCTGCTATTACGAGTTTAGCGTTTGGTAATCTTGATATCGCTATCTTCATCGCGTCTATGGTGTCTGGGTCTTTGCTCGCTAAATATGTGGCAGCACCGAGCCCCCAAATGGTTTCGCAAAACTGATAATAGGGCGCTCATAAGAGCGCCTTGTTAGTTTAGTTGGGTGCTGATAGGGCAGTGGTCACTCAAATGATGTTTGAGAACCAATGAAGTGGGAAAAACATCTTGATTGGTTTGAGCGGTGGTCAACCCAGGGCTCACAATAATATGGTCGATGAGGGAACGAAATTGGTGCGTCTTATTTGGGTTCTTGCGCGAACGCACCTTACATGCTGCCGGCGTGCCTTTTGTCGCCAGTACGGCTTGTGAAGAGTGAGAGATTACTTCCCATAACCAATCTCCGTTGTAACTGAGGTTGTGGTTGAAGTCGCCCAGTATGGCGTAGTTCTCTCCTTGTCGCTCTCGTTCACTGATCCATTGGTTCAATGCCTTGCCCTGCGATTTGAGTATTTTGCAGTCACGGCTGTTTTTATAGGCGCCACTACAGCGAGCTTTTAGATGGACGGACAGGGCATGTATGGGCTGTGAGCCATTTGGGTTGAGTACAATGTAAGTGCCAAATCGGAGTTTACTGTTTTTTCTTTGATCGAGCTGAATGTCTGGCTTATCCGCCACCTCAATATCTTCTTTGACTGCAAATCCCGTATATTGATTGATATCGTCAAACTGTTGCTTCATATACGCGTCTGATGCACGGTCTGAGAAATAAACGTGATAGCCATCTCCAATCACTTTACGCAAAGCCTGTTGGTCGTTCACTTCTTGGAAAGCAAGTACATCACTATCGATTGTCGAAAAGTGCCCAGCTAGTGCCTGAAAATCTTGTTTGCTTCGCTGGGATTCTGAAAATTTGTCTGAAGGGTGAGATGTCAGCCCTCCAAATTCCATGCTGTGACTGTAAGGGAGGAGGAGGCAAAAGAAGCGGTTGAGGTCGTTATCAACAGGATAGCCAAAATCGCTTTGAATTTAGTGTTAGTCATACTCTATAAACTCTGTTCTCAGGTACAGCCTTTATTCTATAAGTCCTCAAAAAAAAGCAAGTTAAAATCCAATAAAAATCACTTTTTGCATACATTTCTTTCAAGCTGAGATCACTGCCACTAAGATCGATTTGATCTTAATCTAGGGCCTAATTTTATTGATCGAAATCAATACACGAGCTTGGTTCATCTCGTTAAATACGCCACAACATATTGTGTCGGTAGAATAAAAAACAACCCTATATAGTGTATTTGTGGATAAGTCTGTGAGTATGTAAAGGGTAAGGAGAGAAGGTGAAACCAATCGTAATCAAGCGTGACGGCTCTAGAGCTCCGTTTAGCAGGGATCGCATTCAGGCTGCTGTTGAAGCAGCGGCAGAGCATGTAGATAAAGAAATCGCTATATACGCGCTGAATGTCGCACTAGCGGTGGAGCTGCAGCTTAAAGATCACGATGAAGTTCATATAACGGAAGTTCAGACGTTGGTGGAAAATGAGTTAATGCAAGGGCCTTATAAGTCACTCGCACGCTCGTACATCGAGTACCGCCATGATCGCGATATTGCACGAGAGAAGCAAAGCGCTTTGACTCGCGAGATCGAAGGGTTAATTGAAGAAAGCAATGTCGATCTGATTAACGAAAACGCGAATAAAGATGGCAAGGTTATCCCAACTCAGCGTGATCTACTGGCCGGGATTGTTGCTAAACATTACGCTAAAACGCACATCCTTCCCCGCGACATTGTTCAGGCACATGAGCAAGGCGATATTCATTATCACGATTTGGACTACGCTCCGTTCTTTCCGATGTTTAACTGCATGTTAATCGATTTAAAAGGCATGCTGACCCATGGTTTCAAAATGGGCAATGCGGAGATAGATACGCCAAAATCGATTTCTACAGCGACCGCCGTTACCGCACAAATCATCGCTCAAGTTGCCAGCCATATATACGGCGGCACGACGATCAACCGTATTGATGAAGTGCTTGAGCCTTATGTGATGGCCAGCTACGAAAAGCACCTTAGCATTGCTCAGGAATGGGACATTCATGAACCCAAAGCGTTTGCACGCGTTCGCACTGAAAAAGAGTGCTATGACGCTTTTCAGTCTCTGGAATACGAAGTGAATACGCTGCATACCGCCAATGGTCAGACACCTTTTGTCACCTTCGGGTTTGGCCTTGGTACTAGTTGGGCCTCGCGATTGATCCAACAATCGATTCTCAAGAATCGTATCGCTGGTCTAGGCAAAAACCGTAAAACGGCCGTATTCCCTAAGCTTGTCTTCGGGATCAAAGATGGTCTGAATCATAAAGCTGACGACCCTAACTATGACATCAAGAAGCTAGCACTTGAGTGTGCGTCTAAACGTATGTATCCAGACATTCTTAACTACGAAAAAGTGGTTGAGGTAACGGGTTCATTCAAAACGCCAATGGGATGTCGTAGTTTCCTAGGAACCTACGAGGAAAATGGTGAGTTGATCCATGAAGGTCGCAACAATCTAGGCGTTGTTAGTCTAAACCTTCCTCGAATTGCGATTGAAGCAAAAGGTGACGAGGGTAAGTTTTATGAGATTTTGAATACGAAGTTAGAACTCGCACGCCGCGCTTTAGAGACACGCATTGCTAGGTTAGAGAATGTGAAAGCACGTGTTGCACCAATTCTCTATATGGAAGGCGCTTGTGGCGTTCGCCTAAAGGCTGATGAACCTATTGCTAACATTTTTAAACACGGCCGCGCGTCTATTTCTTTGGGTTACATAGGTGTTCATGAAACCATTAATGCTTTGTATGGCAATCAGACACACGTTTATGACAACTCAGAGCTTCGTCAGAAAGCGCTCGATATCATCAAGCAC

>NODE_38_length_3547_cov_2.394524

TCACGGTGGCCGTAGCCGTCAAGAATGACTAGAGCCAAAGGCTTCTTAGCTGACATAGTGATGTCCTCGTCAAATTTCAAAGTAACTTAGAAAACAAATTAGCGTAATTTTACTACACTTTTTAACCAAAACTGTAGGATAAGATCAAATATTGTTAGTGAAATATCGTTGTTATCTTTCACCTTATTCCTAGCGTGGTGCTCGATTCTGGCCTGTTTTCCTGTCTTGTTAATATTATTTTCACTCATTAAGAGCAAACATAAAGTGGATTATAACTATCGTTCTAATAGCCGCTAGCTGACTAATAACCCCGTGCCAACAACGGCTAGAATGGCGCCAAACCAGGCGAATCGATTTGGGCGCTTCTTGGTGTAAATCCAAAGTATCGGCAACAGCATGATAGGGGTAGTAGAAGAAAGTAGGGCAACCATGCCGACGTTTCCTTCTTGCAATGCGTAAAGGATAAGTGTCATGCCCACAGCCATCGCGAGGAAGCCGTTAACCGCCGTGATGGCAAACAGACGCAGATTCATTGGTAGCGTTGCTTTCGCGAGTTTCGCGCCACTAAGGAAAAAGACACAGTGAGCTGCAAAGGCCGTCATCATCCGCATAGCGGAAGCCGCGACAGGATCAATAGCCGTCTGCATCACCGGTTTTGCTATAATGCCGCCGAGTGCTTGGCACAGCGCAGCAATTAACCCCAGGCCAATCCCCAACCAGATATTGCCTTTTATGGATTCTAAGTGATTATTGCTTTGCCCTTTGCGACCAAAAAAGATAGCGGTTAGTACGCCGGAAAACACCAGAGCGGAGCCGATAAGTTCGGTACTCGTCATGGTTTCGCTAAACAGGAAGTAGCCGAGTATGGCGGAAAATACTGCATGACAGGAAAATAGCAAGCCAGCTTGGCGCGGGCCCATGCGGTTCAAACAGGCGAACAGTGCGGTGTCACCGATAAAAATACCAATCAGCCCCGACAGCGCCATGGCTGAGATGGCACTGGTTTCAACCGTTGTCCATCCTCCGGTGAGAGCAGCCATTGAGGCGAGAATGACGGAAGTACTGCCCATTCTCCAGCGGCTGTACGCAAAGGTCCCAAGGTGTTGAGCGGGGCCAACAGAAAGCAGGCTGGCAACCGCCCAGAGAAAAGCGGCAGCCAGTGCCAGCCATTCGAATCCCATATGTTGTTCGTCCTGTGAACAAAGAAATCAATAGGCTGACAATATGCATTAAATGGTTTTAGAAACAAAGCCTAAACTTTAAAACGCTCCACTTCAGTTCGGACAGTATTGGCAGAGTTAAACATGTTGGTCGCACTTTCGCTGCTGCGCATAGCCTGTTCTGAGAGAATATCTGACGCGTCCTTAATCCCCTGAGTATTGCGGCTGATGTCTTCACTAACGGCACGTTGCTCTTCTGCGGCGCTGGCAATTTGCAGTGCCATGTCATTGATTTCTGTAATCGCAGAAGTGATGGTATTGAGACTTTCATACGCTTGCTGAGCGTAATCAACACTGGTACCGGCTAAGGCGGTGCTGCTCTCCATACTGTTGACGGCCAGTTTGGTATTGTTTTGCAACGTCTCAATCATGTTGCGAATTTCTTCGGTCGAACCATGTGTGCGTTGGCTCAATACTCTGACTTCGTCCGCTACGACGGCAAAACCACGGCCTTGCTCACCGGCTCGCGCAGCTTCGATTGCAGCGTTGAGTGCCAACAAATTGGTCTGTTCGGCGATTTCTCGAATGGTGGAGAGGATTTGATTGATGCTCTGTGTATTACTCTCCAGCTCAGAAATAATGCCGGATGCCGCTGAAACCTGATCGGCCAGCTCTACAATAGCCTGGCGGTTTTTCTCAATAACTTGTTGACCATCTTCACAAGCAGAAGCGGAGCCTTGAGAGGCGTTAGCAGTCAGTTCCGCATGGCTGGCGACTTCGGCTGCGGTAGCTGACATCTCATGGATGGCGGTGGCGATCTGATTGACGTCGTTTTGCTGTGTTTCCACTTGCTGTGACGATTGTTGTGACAGTTGATTGGCCTGTTCAGCCTGAACGGCTAGTTCTTGGCTGTGATGAATAATGCCACTGAGCATGGATTGCATCTGACTCAGGAATTGGTTGACGTGCTGAGCCAATTCGCCAATCTCGTCCATACGACGAACTTCGATTCTCTGAGTTAAGTCACCTTCACCCTGAGCCAATTGTGACAGGGCTGCTGAGAGCGTTTGCAATGGCTTGAGCAACCGAGTGATAACGTAGGTGCTGATGGATGCGATCAGAATGTAAAGGAAGATTGAAGCAATCGCAGTGAACTGGATTTGCTCGGTCACTGACGCAAACGCCATATCCTTGTCCACGACGATGCCGAGTGACCAATCAGTATGAGGAACATTGGCAACAAATATCAGTTTATCGCCCTGTTCAGGCCAGCTGACGGTGGAAATAGAAGAGGTGTTGACCAACTGCTGGACTTTATCCGCTGACAGTAGTGGGTTGAGTTCGGTCAACGGTTGCTGGCTCAGCTTTTCATCTTGATAAGCAACGATGTTGTTATTGCCATCGACCAAGAATGAGAAGCCATCGTTGTCCAAACTCACATTAAGGACTTCTTCGATGATACTTGTGACGGTTAAATCAGCGGCCAGAACACCATTCTTGCTTCCACTGAAGGCTTTAGCAAAGCTGACAACGATGCTGCCATCAAAGTCCTGATAGGGTTCGGTGACAATCAATCCATTGGTTGCCATAGCATCTTTATACCAAGGGCGGGTGCGGGGGTCGTAATTGACTGGCCAGTCTTCAGTTTTGTCACCATAAGCGATAGAACCGTCCTCAAAGCCAGCATAAACCGATAGGAACCCACCGGCCCGCTTAGTGATCAGCATCTCTCTATCGGCATTGTCCGAGTTGAGAATGCTCGTTTCATTCGCCAGCATCATGTCGCTGCGAATGGAGAGCCAGTCGCTGATATAGCGAGAAGTCGCGTGACTGACGTTTGTCATTTCACTATTCAGTGCAGAAGTAGTTTCTTGTTTTAATTGACTGACGGATATCCAAGCTTGGACACCGGAGACAATCGCAATAATGATCGCGATAGCGATCTGGATCTTTAGCTTGATTGTGTTTCTCATTGCCTTTCCTTAATACTGAGACAACCTATCGAAAATCAGAGTGTGTTGGTATGAATTAGTGATTACACCCTGTTAGTGCGAAAATTCACGTAGGAACTGCTGAAATAGCTATGAATGTTGGTTTAACAGGCTGATGTTATAGAAGTTATTAAAATATAGATTTGATCTATATGGATATTCAGAGAGTGGAATATCAATGAAGTGGTACCTTTTTACCGAATTGTGACTTCTTGCCCATTAATTGAGCAAGAAGTCCGAGGTCACTCTTAAGTCAGCAATAAGGCGGCAATATAGACGATGATTAAACCAATAACACCAATGATCACGCCCGTTTTAGCCATGTCTTTACTCTCAATAAGCCCCGTCGAATAAGCTAGAGAGTTTGGTGGGGTAGAGACAGGTAGAATCATACCG

>NODE_39_length_3505_cov_2.745333

GTGTTCTATTACATGTCCACCCAGCGGTGATTGACGTAACGGAAGAAAATAAAGAGATCACTAATGGCACGTCTACAGTCTCTTTGCCACTAGCGACCAGCTCCATTCGTGAATCGGACTCTGTTATTCGTGCACAAGATGGCGATGTGGTGGTTATTGGTGGCTTAATGAAATCCAACACATTGGATAGAACATCCAAAGTCCCGTTTTTAGGTGATGTCCCCGCTCTGGGGCATCTGTTCCGCAACACTACGCAATATACTGAAAAAACAGAATTAGTCATTCTATTGAAGCCGACAGTTGTGGGTGTAAATACTTGGCAGAAAGAGATTGAACGTTCACGAGATTTACTTCAAGAATGGTTTCCTGATGAGCAGTAGCACATGTATTTGGAGCATTTTGGTTTAACTCAATTTCCTTTTCATCTGACCCCAAATACGGAGCTTTTTTTGGGGTTGGAGCCTCACTATGAGGCCATTCAAACCGTTCAGTCGGCAATTGAAATGGGGGAAGGGCTGATAAAGGTGACGGGTGAAGTCGGCACAGGAAAGACCATGGTATGTCGCATTCTAGTCAATCAACTTGAGCAAGATGTGCAGCTGGTTTATCTGCCAAATCCTGCCCTTAATGGTGATGAGTTAAGACGAGCCATCGCGACTGAGTTGGCCGTTGATTCAGTAGACTCGACCAACTTAGTTGAAGAGATTCAAGCTAAACTTATTGAATTAGCTCGGGGTGGATTGCCTGTTGTGCTGTTTGTCGATGAAGCTCAGGCGCTGAGTGATGAAGCTCTAGAAGTACTGCGTTTGTTTGGCAACCTAGAAACCGAATACAACAAGCTAATACAGATCGTTTTGTTTGGCCAACCGGAACTGGATGAGAGATTAGAGCAACATCATCTTCGCCAGTTCCGTCAACGGATAACTTTCAGTGCACGACTAAGAACGCTAACAATTGCTGAGGTGGTCGCCTACATTGACAACCGATTAGAGAAGTCGGGTGGGGAGGATGGAATCTTTACTGTCAGCCAGAAAAAAGCGATTTGGAAAGCCAGCCACGGGATTCCAAGACTGGTGAATCAAATCTGTCACAAGTGCCTAGTATTAGCGTGTAGCCAACGTAGCTTGAGCGTCAATAATGAACAGCTGTTTAGCGCCATTCACGATACGTATGACAGCTGTAAACCGAAATTTAAAAGCCCGTTAATTTGGGGCTGGAGTTTATCATGAGTGCGATTAACAATGCGCTGTCTGAGCTGGCTAGTAACCATTCGCATAAGCCAGAGCAGGTTGTCAAAGCGCAAGTCAAACCTGTTAAACAGCTCAAAGTGTTGCCCTGGGTGATCGGCAGCTTTGGGTTAAGTCTTGCTGTTGGTGGGTGGGCTCTTTCTACGCAGTCCCCAGAATTGCCGCAACAACCAGTCTCTGTCACTGTCGCTGAGCCCACAATCCAAATAGCCTCTCCGACGTCTAAACCTTCTGAGAGCAGTGGCGTTATTTATAACCCTCAATCTCGTACCGTGAATAAGCCTACCAAACCTGCGCCTGTCATCGAGGAAGAAGCTCAGGCTCATCTACCTCAGCCTTTGCCAATAGCCGTTGCCAGTGTTTCACAGAAACCCACCCAAGTTGCACAAGGTGAAGTTGTTATTCAGCAAATTGAACTGTCACCAGCTCAACTGGCAGAAAAAGCGGAAACAAGAGCCAAGAAAGCGCTTGATGACAACAACCTGAAAGAAGCACTTGGCCATTATCAGGAAGCGCTTCGCTACGCGCCCAATAGCGTTAAGATTCGCCAGAAACTATCGGCGTTATATTACGGTAAAGGTGAAGCAAGAAAGGCGGTGGATATTCTGCGTAAAGGTATTCAACTCGACAAAGATAATACTGCGCTACGAATGTCTCTAGCGAAATTGCTGATGAAAGAAAAGCAAAATGAAGCGGCACTAACGGCTTTAGTTCATCTTCCACAGCAAGTACCAGTTGAGTATTTGTCGTTGAGAGCCGCGTTAGCACAGAAATCCAAGCAAGACGAAATTGCGCTCAGTAGTTACCAGAATTTAGTTTCGATGGAGCCAGACAGTGGCCGTTGGTGGCTTGGATTAGGTATCCAGCAAGAGCGTGCTTTAAATTTACCTGAAGCCGAAAAAGCCTATCAGCAGGCACTCACAAAATTGGGTTTATCCAGCCAATCGCAACAATTTATTCGCGATCGTCTATCGCTAATTAGTCGATTAGAGGAGCAACCAGATGCAAATTAAACTGAGAAAGCGTCTCGGTGACTTGCTCGTCGAAGAAGGCATTATTACCGACTCTCAGGTCGAAGAGGCACTAGCCGCGCAAAAAAGCACGGGTCGAAAACTGGGTGCAACTCTGATTGAGTTAGGTTTTTTAACCGAACACCAGATGCTGACCTTCCTATCTCAGCAGCTTGATGTTCCATTGATTGATCTTAGCCGAGCGAACGTTGACGTTGACGCTGTGCAGCTCTTGCCTGAAGTTCATGCTCGTCGTCTGAGAACCTTAGTGATCGGTCGTCAAGGTGACACATTGAGGGTGGCAATGAGCGACCCTGCAGACTTGTTTGCTCAGGAAGCACTGCTTGGACAATTGCCTCAATACGGTATTGAATTTGTCATTGCGCCTGAGAAACAGCTGGTTGATGGCTTTGATCGCTATTATCGACGTACCAAGGAAATCGCCTCATTTGCAGAGCAGTTGCAGGCAGAGCATCAGGTTACTGAAGCATTTGATTTTAATATTGAAGATGAAGACAGCGATGAAGTTACCGTTGTTAAGCTAATTAACTCATTGTTTGAAGATGCTATTCAAGTTGGTGCTTCAGATATCCATATTGAGCCTGATGCCAATGTATTGCGCTTACGTCAACGTATCGATGGTGTCCTGCATGAAACCCTATTAAATGAAGTCAATGTCGCTCCTGCCTTGGTGCTGCGCTTAAAGTTAATGGCGAATCTGGATATTTCGGAAAAACGTTTGCCACAGGACGGTCGTTTTAACATTCGCGCTAAGGGTCAATCAGTCGACATTCGTATGTCGACGATGCCAGTCCAGCATGGCGAATCCGTCGTTATGCGTCTATTGAATCAGTCAACGGGTGTACGAAAATTGGAACATTCCGGGATTCCTGAGCACCTATTGCTTCGCCTTAGGCGTCAACTTCGCCGCCCTCACGGCATGATACTAGTCACCGGTCCTACAGGTTCGGGGAAAACCACCACGCTTTATGGCGCATTGAGTGAACTTAATGAACCCGGTAAGAAAATCATCACTGCAGAAGATCCGGTGGAATACCGTTTACCCCGTGTTAACCAAGTCCAAGTGAATTCGAAAATTGACCTCGATTTCTCAACCATTCTCAGAACCTTTTTGCGTCAAGACCCAGATATTATTCTGGTTGGTGAGATGCGTGACCAAGAAACGGTAGAAATTGGTCTGCGTGCTGCACTCACGGGTCACTTAGTTTTGAGCACATTGCACACTAA

>NODE_40_length_3486_cov_2.560282

ACTCTGGGTTGCCTGGGTCTTCTTCAATTCTGGCCTAACTAAAATTGCTTCTTGGGACAGTACTCTGTACCTATTTGAGTACGAATATCAGGTGCCAATTTTACCTTGGGAGCTGGCCGCTTACATCGGTACCGCCGCCGAGCTTATTCTGCCTGCTTTCTTAGCATTCGGCCTGTTGACCCGCCCAATGGCGGCCATCTTGTTCGTATTCAATATCATTGCCGTCGTTTCCTACCCGCTGCTGTGGGAGAAAGGCTTCTACGACCATCAGCTCTGGGGCCTGATGATTCTCAACGTCGTAGTCTGGGGACCGGGCTTGGTTTCTCTGGATAAGCTGCTTAAGAGTAAGCTACAAGGTTAAACCCAATACAAAAACGCCCTCCTAATTGGAGGGCGTTTTTAGTTTCAAAGCTCAGTGCTTAAGCACGAATCCCTGAATGACGCAGCAAGGCATCGATTTGCGGCTCACGACCACGGAATCGCTTGAACAACTCCATTGGCTCTTCACTGCCACCCATTTCGAGAATGTTATTTAGGAAGCTCTTACCGGTATCGGTATTAAAAATGCCTTCTTCTTCAAAGCGTGAGAAAGCATCTGAAGAAAGTACTTCCGCCCACAGGTAGCTGTAATAGCCTGCACTGTAACCACCAGCAAAGATATGGCTAAAGCTGTGCGAGAATCGGTTCCACTCCAGACTTGGCAAGACAGCGACCTTAGATTTCACATCCGCCAGTGTTTCCAGCACGCGAGGACCGATTTCAGGGTCAAATTCAGTGTGCAGCGTGAAATCAAACAAACCAAACTCGAGTTGACGAAGAATAAACATCGCCGACTGGAAGTTCTTTGCGGCCAGCATCTTATCCAGCATTTCTTTTGGCAGTGGTTCGCCCGTTTCGAAGTGACCAGAGATAAACGCCAGCGCCTCTTCTTCCCAACACCAGTTCTCTAGGAACTGACTTGGCAACTCTACCGCATCCCACGGTACACCGTTGATGCCCGAAACTGCGCCAGTATCGACCTGAGTCAGCATGTGGTGAATACCGTGGCCAAACTCGTGGAATAAAGTCACGACTTCATCGTGAGTAAACAGCGCAGGCTTGTCACCAACCGGTTTGTTAAAGTTACAGGTCAGATACGCCACTGGTGATTGCAGCTCACCTTTCGCATTGATACGACGTACACGGCATTCGTCCATCCAGGCACCGCCGCGCTTGTGTTCACGCGCATACAGGTCAAGGTAGAAGCTGCCTCGAAGCTGATCGTTGGCATCAAAAATATCAAAGAATCGCACTGACTCATGCCAAACATCAACGCCCTCTCGCTCTTTGACATTCATACCAAACACACGCTTCAGAACTTCAAACAAGCCACTTACCGCTCTTTGTTCTGGGAAGTAAGGACGTAGCTCTTCGTCAGAAATCTGGAACAAGTGCTGCTTCTGCTTTTCGCTGTAGTAAGCAATGTCCCAAAGGTTGAGTTCTTCAACACCAAACTCGCTCTTAGCAAACTGGCGCAACTCTTCAACTTCACGCTCGCCTTGCGGCTTGGCTTTGCTGGCAAGATCATTGAGGAAGCCCAACACTTGCGATGGGTTTTCTGCCATTTTAGTCGCCAGAGACTTCTCACTGAAGGTATTGAAACCTAACATACGGGCGATTTCATAACGCAGCTTCAACTGCTCGTTAATGATTTCACTGTTGTCCCACTCGCCCGCTTTCGGGCCACGATCCGAGGCACGAGTGACGTAAGCTTCATAGAGCTCTTTACGTAATTCCTGATTGTCACAGTAAGTCATCACCGGAAGGTAAGATGGGATATCCAGCGTTAGCAGGTAACCATCCAGCTCTTTGGACTCTGCCGCTGCTTTGGCTGCCGCCAGTGCAGACTCCGGCATACCTGCCAATTGTTTTTCATCTTCAATGTGTTTGGCCCACCCCATGGTCGCGTCCAACACATTGTTAGAGAACTTAGAACCCAACTCAGACATGCGCTTACTGATTTCACCGTAGCGGTGCTGTTCGTCGGCTGGCAGACCAATGCCTGACAACTCAAAATCACGCAGGGAGTCTGTGATGGTTTTCTGCTGTGCCTGAGTCAGCCCAGCGAATGCCTCACTCGCTTTAATTGCCTTATATGCCTCAAACAACCCTTTGTGCTGCCCTACCCAAGTGCCGTATTCGGACAAAAGAGGTAAGCAGCTTTCATACGCTTCACGCAATTCATCACTGTTCATCACCGAGTTCATATGGCTCACAGGTGACCAAAGGCGGCTTAGGTGATCATCCACTTCTTCAATCGGCGCAACGACATTTTCCCAACTTGGGTCGCTGTTTCCTTCCAGTACCAGATCGATTTTCGCCCGGCAGTCTGCAATCGCCTGTTCAACGGCTGGTTTCACATGTTCAGGTTTGATTTCAGAAAACGGGGGCAAATCGGTAAAGCTCAGAAGAGGATTAGACATAAAGCATTCCTTTTATTGGCAAAAAGCGAACTGACGGGTTCGTCTTTGTTATTTCTCGTTCGTCTTAAATAGTAAATATAGGTAGGTTCAGCAATTTTCAATAGTGACGTATACTACAGCTATTAAATTACCCGGTGTTGTCACCAGTTAGAGAGTGTCATTTTGTTAAGTTATCGCCACAGTTTCCACGCAGGCAACCATGCTGACGTGGTTAAGCATATTGTTCAAAGCCTTATCCTCAACGCGCTCAAGCAAAAAGATAAGCCATTTGTTTACCATGATACCCACTCAGGAGTGGGTCGCTATGACTTAACCCACGAATGGTCAGAAAAGACAGGTGAATACAAACACGGTATCGCGCGTGTCTGGCAACAAACTGAGATTCCAGAAGAAATCCAAAGCTACCTTGAATCGATTCAGACACTGAACAACGGTGACAAACTGCGTTACTACCCAGGCTCGCCGCGTGTCGCTCGCGCGCATCTGCGTCCACAGGACCGCATGGTATTAACGGAACTTCACCCAAGTGACTACCCGTTATTAGAACAAGAGTTCCATCGCGACCGTCAGGTCAGCATCTATAAGGAAGATGGCTTCAAGCGTTTGAAGGCGAGCTTACCGCCGCAGGAGCGTCGTGGTCTGGTACTGATTGACCCACCTTACGAGCTGGCGAAGGAATACCGTGATGTCGTCCAAGCGATTTATCAGAGCTATAAACGCTGGGCCACGGGGATCTATGCTATCTGGTATCCGGTCGTTAATCGCTGCGACATTGAAGACATGATTGAAGGTCTTGAAGGCCTTGGCATCCGTAAGATCTTACAAATTGAATTAGGTGTTGCGGCGGACAGCAATGAACACGGGATGACCGCCTCAGGCATGATTGTGATTAATCCTCCATGGAAGCTGGAAAGCCAGATGAACGCCATTCTGCCTTTCCTCAAAGAAGCAATTGCGCCTGCAACCGGTCATTTCAAAGTAGACTGGATTGTACCGGAATAACGGACACTCGCGCTCAATGTAC

>NODE_41_length_3473_cov_2.320377

CCATTCGCCATAGCCTTCTTGATCGCCTAAGCCGAACAATGCGACGGTCTTGCCATTTAGCGATACGCCATCGATTTGATCCCATATCTCATTCCAGTCTTCTTGAATTTCGCCGAAATCCCAAGTTGAAATGCCTAAAATCAGAAAGTCATAATCAGCCATCTCTGCCAGAGGCGTTTCTTTGACATTGCGGATATCCACCAGTTCTTCACCTATGATGGCACGCATTTTCTCCGCTGCCATTTCTGTGTAGCAAGTCGTAGAACCGTAAAACAGACCAATTTTCATCACTTTCTTTTTCATTATTTTGAATCAATAGCGAATTCTAAACACAAAATGGCGAGCTTTGCAGCGAATATCCATATCGAATGGCAATTTTTATGGCTTTATTGCTGCGTGGCACTTACATTGAGCGCATATCATAAAGTTGAGGATTACTGTGTCAGAAGTGATGTCGCCCGATCAGGGGATGGTGGAGCAGTTTCTGGATGCGATGTGGTTGGAGCGAGGGTTGTCGGAAAATACGCTGGCATCTTATCGCAATGATCTATCCAAACTACTCCAGTGGATGACTGAAAATAACTACAGGCTCGATTTTATTAGCCTGTCTGGTTTACAGGACTACCAAAGTTGGTTAATGGATCAGGGATACAAGCAGACCTCACGAGCCCGTATGCTGGCAGCAATTCGTCGCTTATTTCAGTATCTGCATCGAGAAAAAGTGCGAGCGGATGATCCTAGTGCTTTATTGGTCAGCGCTAAGTTACCTAAGAGGCTACCCAAAGACCTGAGCGAAGAGCAGGTTGAAGCACTACTGAATGCGCCGGATCCAAACGATGCGATGGAACTGCGTGATAAAGCCATGTTGGAATTACTCTATGCGACAGGCTTGCGTGTGACCGAATTGGTCAGCTTGACGATGGAAAACGTCAGCCTTCGTCAGGGCGTTGTTCGTGTTACAGGTAAGGGCGGTAAAGAGCGTTTGGTACCAATGGGTGAAAATGCAGTTGATTGGATTGAAACTTTTATCCAACAAGGCAGGTCTGAGTTACTTGGGGAAACTACATCTGATGTCGTCTTTCCGAGTAAACGAGCACGCCAGATGACTCGACAGACATTCTGGTACCGTATTAAGCACTATGCGGTTTTGGCTGGTATCGATACCGAAATGTTATCGCCTCACGTATTGCGCCATGCTTTTGCGACGCACTTGCTCAACTACGGCGCCGACCTGCGCGTAGTGCAGATGTTACTCGGGCACAGCGACTTGTCGACGACACAAATTTATACTCATGTAGCGACAGAACGGCTAAAACAGCTACACAGTGAACATCATCCAAGAGCATAAGGGATTATCTCTTATCATATTAAAAATAAGGTGAATTGAATGAGCGTACTACGCCGAATGACTCTACTTACGTTACCACTGCTTGTGGCAGCACAGGCAGTATCTGCAGCGGAAGTAAAATTTGATAAAGCTCAGCTTGAAGAGCGTTTTGGCAAGCTAGGACTAGAAGTGAAACAAGTTGTTCCTGCAGAGATCGATGGCTTAGTCGAAGTGCAGACTAGTGGCGGAATTCTTTTCGCTTCGCCAGACGGTGAGTACTTTTTAGCGGGTACACTTTATAAGCTTGATGGCAATGGTCAGTATGAAGATGTACTGGCGAAACGTCAGGCACCGATCAATGCGGCTAAAATTGAAACCTTCAAAGACAGCATGATCGAGTTTAAGGCCAAAGACGAGAAATACGTTATCACTGTCTTCACGGATATTACTTGTGGTTACTGTGTACGTCTTCATAGCCAAATGAAAGACTACAACGACTTGGGTATTACTATTCGTTACATGGCTTACCCACGTCAGGGCGCAACGGGGTCTGTTGCGGATCAAATGGCAACGATTTGGGGTTCAGATGATCCTCAAGCTGCCATGCATAATGGTAAGGTAAAACGTGAATTCCCTGAGAAGAGCAAAGACTTCGCCAAGTATCAGGAAATCATCAAAGAGCATTATGCGCTTGGACGTGAGTTAGGTATCAGCGGTACGCCAGCGATTTTCCTACCAAACGGTGAAATGGTTGGTGGCTACTTACCACCAGAGCAGATGCTGCAACGCCTGCAACAAACACAAAAATCGTAACTCAATCTTCAGAGTTACGTTTTGAGCGCCTGACAAGCGAGCTTGTGATGAGAATCCTATGTCATGAATTAGGTTGATATAAGATTCTCATCTGTTAACTTGTTCGCTAATCAGGCGTCCTGCCCAAGAACAAGGCTCAATCCAATCTATGATCGAAATCCAACGCCGGCCTGAGGTTGATCTCAGTCTGCTTTCCACCGACATTGCTCCACTTCTTAAGCGCATTTATATCTCACGCGGTATCACCAGTGCCCAGCAGTTAGAAACGACGGCGCGGGCATTGCACTCTTATCAAAAACTGCACGGTATTGACGCCGCAGTCGAGCTGCTATTTGAAGCTATTCAGGGTAATAAACGCATTATCGTTGTCGGAGATTTCGACGCCGATGGGGCAACCAGTTCGGCCTTATCGGTGATTGCATTACGAATGCTAGGTTCCAATAACGTCGATTATTTGGTGCCGAATCGTTTTGAAGATGGTTATGGCCTTAGCCCAGAAGTCGTTGATCAGGCGATTGAGATTGGCGCTGAAGTGATTATGACGGTCGATAACGGCGTCTCTTCGATTGATGGCGTGCGTTACGCCGAAGAGCAAGGGCTTAAAGTATTAGTTACTGACCACCACCTGCCGGGCCATGAGTTACCTAACGTCGATGCGATGGTCAACCCAAATCTCGAAGAGTGCGCTTTTCCTTCGAAAGCGTTGGCTGGCGTGGGAGTGGCGTTTTATCTCATGATGGCGTTGTGTGTTCATATGAGAAAGCACGGATGGTTTGCCGAGCAGGGCATGGCTGAACCAAAACTGATGGAGCTAATTGATTTGGTTGCTTTGGGTACTGTTGCTGACGTTGTGCCACTAGATGAAAACAATCGTATTCTGGTTCACCAAGGCCTGCAGCGTATTCGTGCCGGCAAAGCACGACCTGGCATTCAAGCATTGATTGAAGTCGCGAAGCGAGATGCGCGTCGATTGGTTGCTGCCGATTTCGGTTTTGCACTAGGTCCGCGTATCAACGCAGCTGGTCGCTTAGATGATATGTCGTTTGGTGTTGAGCTTTTGATGGCTAACAACATTCATGCTGCACGTCGTATGGCGAGTGAGCTGGATGGTCTGAACCAAACGCGTAAAGAAATTGAAGAAGGTATGAAGCAGGAAGCAATGGCTTTTTGTGAGCGCCTTCAGTTTGGTGACAAGGATATGCCTCATGGACTGGTGCTTTTTCAGCGTGACTGGCACCAAGGTGTGATTGGCATTTTAGCATCTAGAATCAAAGAGAAGTTCCACCGACCTGTGATTGCCTTCGCTGATGGTGGCGAAGGGAGCATTAAGGGC

>NODE_42_length_3463_cov_2.299468

AATCACTGAACAGCTACCAGTTGTTCAGTGATTTGCGCGATCTCTGTACCTGGGTGAAGTACTTTGGCTGGGTAAATTTGATCCCACCAGAAACCAGGGCGGAAGAAAGTAAACGTCAGAACCAAGAGCAGAACCGTTTCCCACCACTTGTTCTTCGTAAACCACCAGCCTTGTGTTGCTGCAGAGAATATCAGCATTGCGATGACGGAAGATATGATCGTGAGCAACAAATGCCACCAACTATCAATACCCATCAAAAGAAGCTGAGTATTGAAGATGAACATAAATGGCAAGATGGCAGTACGAATATCGTAGGTGAAGCCTTGAATACCTGTTCGAATCGGATCCGACTTCGCGATCGCCGCTGCTGCAAAGGCAGCCAAACCAACCGGAGGTGTATCGTCTGCCAGAATACCGAAGTAGAACACAAACAAGTGAACGGCGATTAGAGGGATAATCAGGCCGCTTTGTGCACCTAGCGTTACAATAACGGGTGCCATCAGCGTAGATACCACGATGTAGTTTGCTGTTGTTGGCAGGCCCATACCCAAGATAAGACTGATGACTGCGGTAAACAGCAACATCAGGATGATGTTACCGCCAGAGATAAACTCAACAAAGTCCGTCATAACCAGACCGATACCCGTTAGAGTCACCACACCGACAACCGTCCCCGCGGCTGCAGTCGCAACACCGATACCGATCATATTACGAGCACCAGACACTAGGCTCTCAAGTAAGTCAATAAAGCCTTCTTTGGTTTGCTCAGCAATCGATCCTTGTTTAGAAAGTACGGCCATGAGAGGACGTTGAGTAATCAGGATAAAGATCATGAATACTGTTGCCCAGAACGCAGATAAACCAGGAGAAAAACGTTCGACGGTTAAACACCACACCAGCACAACGATTGGTAGCAGGTAGTGTAAGCCAGACTTAATTGTTGGGCCTGGATCTGGTACTTCAGTCAGTTCGGCATCAATCTCAATCGCCCCTTCTTTGGCATATTTGGCCGAGATATTGACTAAACCGACGTAAGATAGAAGTAGCGCAACGGTGACGATTGGTGTAGCAGCATCACCGAAGACGTCTTTCGTCCAACCAATACCGTAGTACACCGCGGCACTGATGACACACAAGCCGAGGATGGTGCCAGTAAATGACAGCAAGCTCGCGATTAGCGGCGGATTGTGACGACGCGGAAGGCCAGTCATACCCGCTTTACACGCTTCTAGGTGAACAATGTAGATCAAAGCGATGTATGAGATTAGAGCTGGAAGAATCGCGGCTTTAATCACTTCTACATATGAGATACCGACATATTCAACCATAAGGAAGGCTGCTGCGCCCATGATAGGTGGCGTTAACTGACCGTTGGTTGAAGCGGCAACTTCTACTGCACCTGCTTTTGTTCCTGGGAAACCGACACGTTTCATTAGTGGGATAGTGAAGGTACCAGTGGTCACAACGTTAGCAATTGAAGAGCCTGAAACTAGACCTGACAAACCTGATGCCACAACAGCCGCTTTCGCTGGGCCGCCTTTCATATGGCCGAGTAGAGAGAAAGCGACTTTAATGAAGTACGCGCCTGCACCAGCACGTTCTAGCATTGCACCAAACAGTACAAACAGGAATACGAATGATGTCGAAACGCCTAATGCAACACCAAATACACCTTCCGTTGTTAGCCATAGGTGCGACATGGCTTTGTTCAGACTTGCTCCTTTGTGCGCGATAACATCCGGCATGTACGGACCGCCAAAGGTGTACAGCAAGAAGACTGCAGCAACAACCATCAGTGGTGGACCCAGTGCACGACGTGTAGCTTCCAGTAGCAGCACCATTCCTATGACTGCAACCACAATGTCAAATGTTGTTGGAGCACCTGAGCGGCCAGCCAGCTCGGTGTAGAAAATATAGATATAAGCAGCGGAGAAACTGCCGGCTAACGCCAGTAGCCAATCCAGAGCAGGGATTCGATCACGAGGAGAAGTTTTCAGAGCAGGGTAAGCGGTAAACGCTAGGAACACGGCAAACATCAAATGTATCGAACGTGCTTCAGTGTCGTTGAGTACGCCAAAATTAAATATGAACGGCAGAGGGGATGCGTACCAAAGTTGGAATAATGACCAACATAGCGGCACAAACCATAAGATGCGGCCAGGAATCCCCAACGGGGCACGGGCACCTGTGTCGGCTTGAGCTACCATTTCTTGCACGTCTTGAGACGGAGATGTTGTCTTCGACATGTACTTAATCCTTATTATAAATGGCCTGTCTTTTTATGATTGTCAGAACCTGATTTTGCATCTTTCAGTTTAGTGAAAAGATGAAATATTGCTCGGTTGTAAGTTTTTGATAGATAGAAATAAAGAGGAGTTTCAAAAATGTGAAACTCCAAATAATACGATATTGCTCAATACCTTAGAGCTTGCTGCACCAAGGAGGTAGTGACCTCCCTGGTGAACAAAGAGAGGTTACTTAAGTAGACCTACTTCTTTGTAGTATTTCTCAGCACCTGGGTGTAGAGGGATAGAAAGACCAGCTTTCACCATGTCTTCTTTCTTAAGGTTTGCAAACGCAGGGTGCAGACGCTTAAAGGTATCGAAGTTCTCGAATACAGATTTTGCAACGTTGTAAGCAACTTCGTCAGAAACGTTTGTTGTAGTTACCATAGTAGCGGCAACACCAAAGCTGTTTACGTCTTTATCTGTACCACGGTACATGCCCGCTGGAACTGTACTGAATGCGTAGTATGGGTTGTCGGCAACGATTTCATCGATTTTCGCGCCAGTCGCTGATACTAGCTTCGCATCACATGAAGTGGTTGCTTCTTTGATTGATCCGTTTGGATGGCCAACAACATAAACGAACGCGTCGATTTTGTTGTCACACAAGGCTTGTGAACGCTCAGAACCTTTTAGCTCAGATGCAAGCTTGAAGCTGTCGTTAGTCCAGCCCATTGCTTCCATTACTACACCCATAGTCGCACGGTCACCAGAACCTGGGTTACCGATGTTTACGCGCTTGCCTTTCAGGTCTGCAACGTTTTCGATACCAGAGTCAGAACGAGCAATGATGTTGAATGGTTCTGTGTGTAGAGAGAACATTGCACGTAGCTCTTTGTACGGGCCTTGTTCAGCAAACTTACTTGTACCGTTGTAACCATGGTACTGCCAGTCAGACTGAACGATACCGAAGTCCAGCTCGCCCGCACGGATAGTGTTGACGTTGTAGATCGAACCACCTGTCGATTCTACAGAACAACGAATGTTGTGATCTTTACGGCCTTTGTTAACTAGCTTACAAATTGCACCACCAGTTGGGTAGTAAACACCAGTTACAGAACCTGTACCAATAGTGATGAATTCTTGAGCGTTAACCGCGCCAGCACCCATTACAGCAGCTGCAATAGCACCAACTTTAATAAGTTTGTTCAATGCCATGAATTTCCCTTCCTTTATTC

>NODE_43_length_3439_cov_2.575550

CAAAGTGGAGAGTATGATGCCACTGCGGTATGGATGTTTGATGAAGATCGGACGGAGCATTTCTATTACAGTGAACCAGTCTCGCAAGAGCAATTTGTCTTCTTTCATCACAAAGACTTAGCGTTTGATTGGTCTAAGATAGACGATCTCAAGGGATACAAGATGGGTGGAGGGTTAGGTTACAGCTATGGGGAAGCTTTGGATGCCTTAATCGATGCAAAAGAGGTCAGCATAAGTCGAGTCAACAACCCTAGCCAGAATCTGCTGCGCCTTAATCATAAACGTATCCAAGTCTATCCAGAAGAACGTCAAATCGGCATGTATCATCTCAGCCAGCAACCAGAAGAGATTCGAAATAACATTACCTATCACCCGAAACCCTTTCTTAATAACGACGGTTTCGTGATGTTCTCCAAACAAAGCCCTAGAGGAAAAGAGCTATTAGAGATCTTTAACCAAGGGCTTATCAAGTGGCACCAACAACAATCTAAAGGCCACTAAACGACACGGTTTTGTTCCTGCCCTTGATGAAAGGCAGTGATGTTATCCATCAGGATGTTAGCTAGATTCTGAATCGATGAATCACTGCCCCACGCAACATGTGGAGTCAGGAGCAAATTAGGCAGATTCATATTAGCGAGCAACGGGTTGCTGTCGTCTGCTGGCTCTTGGCTAAATACATCGACACCAGCACCAGCAATCGTGCCTCGTTTTAAGGCCTCAACCAGAGCCGCTTCATCAACCAACCCACCTCGACCAGTATTGATCAGCACACTGCCAGGCTTCATTTGAGCCAGTTCATGACACCCAATCAAATGATGAGTCTGTTCATTCAACGGACAATGTAGCGTCAAGACATCAGCCATCATCAACACCTGCTCAAATGGCAGGTAACCTTTTCGGCAATGATGCGCACCTTTATGTTCTGCATAAATGACTTGCATACCAATCGCCTTCGCCAGCGCGGCAGTAGCCTGCCCCAAAGCTCCAGAGCCGACAACGCCCAGCGTCGAGCCTGCTACGTCACCAATTGGGTGAGTAAAGAAACAAAACTGTTTATTGCGTTGCCATTCCCCTTTGGCAATATCATTGTGGTAGCCCATCAAATTGCGCTTAAGAGCGAATATCATCCCAATCACATGCTCAGGCACCGATTGAGTGGCGTAGCCCTGCACGTTTGTGACGGCGATATTTTGCTCACGGCAGTAATCAATGTCGATGTTGTTGAAGCCAGTAGCCGAGACAGCGATCAGTTTGAGATCCGGTAGTTGGCGCAACACTGCCGCATCTAATACGACTTTATTCGTGATGACGATATGAGCGTTTTTCAGCCGTTCTACCACCAGCTCAGGGCTAGTGAAATCATATTCAATCCATTGGTGATCAAAAGGAAGGTGAGGTAATTCGATATGAGTTGGGATCGTCGCACGATCCAAGAACACAACATTAGGCAGCGACATGGTTTCTCCTTTCGCTGCCTATAATGAAATCATGGACGCGGCAGCGTTTTATAGTCCGGCAATTCTCGCACAGGATCGAAATAGTTAAAGATCAACGCCTCCGCTTCAGGATAGAAATCACAAGGAAGAAAAATACTGCGTAGACGTTCATCTTGAGGGTGGTAAAAACTCAATTCAGCCGCATGCAGGTCAAGACGGTTGGAGAATTCAAAGGCTTCTCCCTGTGAATAGAACTCATCTCCGACAATCGGATGCCCTAACGCCTGCATATGCACACGCAGCTGATGTGAACGCCCAGTAATAGGCAGTAACCTGACGATGGTGGTCTGCTCTTCCCGCTTCGCCACCTCAAATAAGGTTTTTGATGGCTTACCCTGTTCAAAACAGACTTTCTGCTTCGGCCGATTTGGCCAATCACAAATCAGTGGAAGATCCACTTCTCCCTCTCGCTGTTCGACGTGTCCCCATACACGAGCATAGTAGACCTTATGCGTGAGGCGAAACTGAAACTGTTTTTTTAACGCCGATTCTGTGGGCTTGTTTTTGGCAAAAAGCATTAAACCTGAAGTTGACATATCAAGACGATGAACCGGTTGGATATCAGGATAGATGTCTTTCAAGCGGCTCCACATACTGTCGTAATGCTCTTCAGCCCTGCCTGGCACAGACAAAAGACCCGACGGTTTATTCACCACAAGGATGCCTTCATCTTCAAATACTCGCTCAATCCATGGCTCGGTGGGAGGATGGTATTGCTGCATAGCCATAAAACATCTCAATCAGATTTATAACTCAGCCAGTATATCAGAGTCATGCCTGTGACGGCGTTTCCACATCCTAGTATGTTTTAGCTTTTTTCATGTAAATAATATTTAACGCCTTGGTTGCATATGATATTCATACCCGCCATTGACAAGACACAATCACTAACAACAAAAACATCAAGGAACACAAATGGAAACGATCTCCGCTTCAGAGAGCCAACCCAAACACTCTCTGTCACAACTCGTCCGTTCGCTAGGCCCAGGGATCATGATGGCCGCTGCCGCGGTAGGGGGCTCCCATCTGGTGGCTTCAACAAAAGCCGGGGCGATTTACGGATGGCAGCTAGCCGCACTCATTTTGTTAGTTAACTTTTTTAAATATCCATTTTTCCGCGCCAGTATCCAATACACAATGGGAACCGGAGAAAGTCTGGTCGAAGGTTATGCAAAGCTTGGCCGACCTTACCTATGGCTGTTTGTCGCACTCAGCGTTATTTCCGGTATCGTCAATACAGCCGCCTTACTGCTCTTCAGTGCTAGCTTGCTCGGTTACTTCATTCCCTTCGACCTCTCGACCACAAGCTTGTGCTTAATCATCTTAGCCACCTGTTTAACAATTCTATTTGCAGGTCACTACAAAGCCCTCGACTCCTTATCCAAAATCATTATGGCGGTACTGACCATTGCAACCCTGACTGCGGTCGCTATTGCCGCAGGTAACCCTGTCGAAGCGGCTCCTGCTTTTGAAGCGCCCTCACCTTGGTCCATTGCCGCCATCGGCTTTATTGTTGTAACCATGGGCTGGATGCCAGCACCGATTGAAATTTCCAGCCTGACTTCAATGTGGCTTAAAAGTCAGCAACGCCACCAAAAGGTAACGGCTCGGTCCGCTTTGTTTGATTTCAATGTGGGCTACATAGGTACGGCGATTCTGGCGATTGTCTTCCTTGCACTGGGCACTTTGGTCTTACACGGGAGCAATATTGAATTATCTCAATCGGGTGTAGGATTTTCTCATCAGCTGGTTGGACTCTATGCATCAACCATTGGCGAATGGTCACGTTACCTGATTGCAGCCGTAGCGTTCTTCTGTATTTTTGGCAGTACGATTACGGTGATTGATGGCTACGCTCGAGTGATTTCAGAATCTCAACGCCTACTGCGTAAGCAAAAAAAAATGAAGCCTAAAGTGACTCAGGGTTGGATGTTACTTAT

>NODE_44_length_3397_cov_2.602108

TTATTAAAAATAAATGAGTAAAATAATATTTATAAGCACGCAAATAGCACACTGCATTCAAAATATAAACCACACAACAATAATGATTATTGAATAAATCGAATTCGGCTGAATAATAAGATTAGTGCGACCAAGAGTTGAAGTATAAAGCATGAAACGAACACCGCTTGCTCACCAAAGCTAGCAACCATATACCCTGACGCTGCTAAACCAAACGGCATGAGCAACTTAAACAACGAACCCGTAATTCCCGTCAGCCTGCCGAGGCATTGCTCATCGAACGCTTCCTGGCGGTAACTCCAGATGCAGATCGAGCTGTATAAGCCAATGGCAGAGATCCACAAGAAGGCAAAGACCAGACTCCACACTCCGGGAAGCACCAGAGGAATGAGGAAACCAAACGCCTCCAACGCAATTGAGGCAATCAATAACTGGCCTAAACCAAACTTGGTACGTATTTTATCTGCGGTAAATGAACCCAATAAGCCACCCACACCCGAAGCGGCAATCAAGTAGCTCACTTCAACTGAATTCAGACTGAGAGTGGATTTCGCATAGAAAATCGACTGAATCCAGAATATTGCACCTGTAGTGTTAATTACCATCACGGCCAAGGTGATGTACCACATATTGGTTTCAGCACGCAGTATCGCCCACCCTTCCGCCAATGCTTTTAGCACGGGGGGATGCGATTTCGGCTCTGGTTTCACTAAAGCTAACTTTTCCAGTTGCCAATAGGCCAGCACCAGCATCGCTGCCACTCCAAGAAAGACATTATGAATCGCTGACAAGAGCATCAACGCACCAGAAAGTACCGGTCCAACCGTTTCCATAAAACTACTCAGCGAACTCATGCGCGCAATAGCAGTATTTTGCAGTTCATGAGGAATACCATTTTTCATCATCGCCATGCGTGCATTGTGATATCCGTAATTACACGCCATCATGAAAAACGCCGACGGAAAAAGAACCCAGAGCGGGTTATCCAACCATTCCACCGCAAAATAACAACTCAATACCATCAGCAACTGGCCTAACAACATCATTTGAGACCAGCGCTTTTTGTCGACTCTATCGACCCATACACCAATAAACAGCGCCAGCAATAAATTAGGTAGGAATTCAACTGCTCGCATCCAGCCCATCATCTGAGACGACTGAGTCAATTCATACACCAACAAAGGCAATGCCAAATTATAAATCTGGGCACTAAACGCCACGAACAATGCGCTGGAAAAGAGCACCACAAACGATCTATTTTTCCACACTGAATTTTGAGGGTGAGATAACGTCTCCATCGAATAATCCTTTCTTCCTGATAAGAATCCACTATTATCCGAATCAAAAGATGAAGAAAAATAGAAATAAGAAAAAGGAATTTCACCTTTATGCGTTACTGGAAATCCCTCGCGTTCTGCCGTGATAATCTGCAGTTTGAGCAGTGGCGGCCAATGTCCTTGGACAATTTCAGCCAATCACTGGATTGCACAAGACGCAATGCCCAATTACTCATTAAACGATTGATTAAAGAACGCTTCCTGGAGTGGCAGCCGGGGGTAGGCCGAGGTAATTTACCCAATGCAAAGCTATTGAAGTGCCCAAACGAACGATTGGAGCAACAAGCTTTTCGTCTATTAGAGGAAGGAAAAGTCGCACAGGCTCTGGTACTGATCAAGGACGCACATCGCGATCTATTTCTAGAAAACTATCTCGCTCAGTTTCAGCCGAAAGATGAGTCAAAACATATACTAAAGATCCCTTTCTACCGCGGCACTCATTGCCTAGATCCCGTCTTTATCACGCGGCGAACAGAAATCCACCTTGCACGCTATCTTTATGCCAATCTGCTTACCATCAATACCGATAATCTCGGCATACAAGCTGATCTGGCACACTCATGGAAGCTGAGCGGCCACACACTTGAAATAACATTACGCAAAGGGCTTAAATTTCAGGACGGCAGTCCTTTGCTATCTCAGGATATCAAAGCTCACGTTGGACGATTGATGAGCTCAGACGCTGGCTGCAAAGTTCTGTTTGAATTTATTGATGACGTCGTCGTAATCGATGACCTATCTATTCACTTTGTCAGCCATTCAATGCCGAACCTACTGCCTAAACTGCTGGCACACTCTGCTATGGGCATCACCAAAACAATCAAAGGTGAAATCGTCGGAACTGGCCCATTCTGCTTAGAAGAGCAATCTGAGTGGCGAACACTAATGACCATTAACCCGCACTATCACGGATTCAGACCTTGGATAGACGGTATTGAGATCTGGAACATTGGTGATAAAGCTAAAAACTTTGACCTAAACAGTCATGTCGTACATGGCAGCCACATACGTGAAGGACACGCATCTGGCTTCCGTGCACAAAATCAATGGGAGGAAGGTTGTGTCTACGCAACATTCAACGTGAATCGTAATGGCTGGATGACCAAACGAGCTCACCGGGCTGCGCTGCAAGAGGTTTTATCTCATATGGGCGCGCCGCAATCGCAACAGTGCGAAGCGCTCGCTGGAGCGAGCGGCATGATGTCTTCTCCCGAGTCACTCAGACCATCAGCGCTAGATTTGATTGCAGAGCATATTCGTTCGCTCCCTAAGCCGGAAGCGCCTCTCTCTGTCGTCACCTACCAACTTGCAACCCATGTAGAAACCGCTCAGCTAGTTGTCGAATCGCTAGAAAAGTTAGGCATAGATGCCAAACTGACCGTTCATGAATACCCCGTGTTTGACCAACTTAGCACCTTATCTCAAGCGGATGTCATCATTTGTGGCGAAGTATTCGGTGAAGATACTGAAATGTCTTGGTTAGGCTGGTTATTTAACACCAGTGCTCTTACCGCCTGCCTGACCAATAAAGAGCAACATTGGATCAAGCAGAAATGTACTGAAGCAATGGCACAACCTAAGCTAGCTGCGAGACTCAAACAGTTTGAAAAGATTGAGAAAAGCTTAATCAGAAATGGCATTTATCAACCACTCTTTCATGTAGAGCAGGACCTCAATATCTCAGACACCGTGACAGCGCCTGCACTGCTTGCCAACGGTTGGATAGACTTCAATCAGGTGGTGATGAAATAGGTGGATCGACTAACGTCACTTTACCCTGTTTGTCGATTCGATAGCGCAGTAAGTCTTGTGCAAGGAATAAGTTGCCAGAATATACCTGCTCAGCCTCTGAGCGAATAATCTCGACTGACAGCTCTGCTTCAGGGTGAAGCTGATAACGTGGGCTGAAATGGGTGAGAATCAAGTTAGGGATACCAGCGGCTTCAGCAAACTCCGCAACCAACTTTCCGTAGCTGTGGCCGAACTCTTTTGCCTGCTTGGCGAAAGTTTGGGCATAAGTCGACTCATGCACCAGCAAATGGCACTCTTCACACAATTGT

>NODE_45_length_3388_cov_2.289641

GACTAACACCGGAGCCAATACAAACGCAGGGATGACCACTCCTGTCATTGAAAGTGACATAAGTAAATAGTCAACTTTACCATTGCGGTTTAATGCGGCAATGGTGCCAAGTAACATACCTAATGGCACAGAGATACAAAACGAGATAACACCAAGGATCGCCGACACTGGCCAAGATTGAGCAACAAGCTGGGTGACGGTGAAATCCTGGTAGACAAAGCTTGGGCCGAGATCGCCTTGAAGGAAGTTGGTGATGTAAATGAAAAACTGAACCAGAAAGGGTTCGTTCAAGTGAAATTTGGCTTCGATATTTGCCCGTACCACTTCTGGCATTGGGCGTTCCATATCAAATGGGCCGCCGGGCGCGATATGCATTAACCAAAACGACACCAGTGCAATAAACAGCAATGTCGGTATGGCAATCAGGCAGCGGCGCAATATGTACAACAACATGACATTTCCTTGCGTAGAAAAAAGCCCGTAGCCTGTGCTAGGGATAAGTGACAGGTACGGGCAAACTCGGGTTAATGTGCTTTTACATAGACATTTTTGCGGTAGTAGGAGGATTCCGGATTGGTCCGCTCATAACCGCCAATGTAATCCTTGATCAGGTATTGATCGTTGCCATTGCGGTACATAGGGATGATCGCCATGTCCTGAATCAGAAGCTTTTCCGCAGCAATGTATTCCTGTGACGGGTCACTGAGGCTCTTCGATTCAGCCATCAGGCGATCATATTCTGGGTTGTTGTACTTTGAGTCATTGTATTCGCCGGTCGAGACAAAGATGTCCAGCCAGGTTGAGGCTTCGTTGTAGTCAGCCGTCCAGCCACCACGGTTAATGTTGCCCGGATCTTTCAGCGCATAGAATACCTTTGGCTCAAGTTGCTTAATTTCGATTTGCGCGCCTAACACGCTTTTCCACATGCCCGCCATCGCGGTGGCCATTTTGACGTCTTTAGAAAAAGTCGGCACGGTGAAAGTGAGCTTAAGGGGATTGCTCTTGCTGTAACCCGCCTCTTTAATCAGCGCTTTAGCCTTGGCGTTACGTTGTTGCTGAGTCCAAGTCGCGAATTCCGGTGTGTGAGATTTGAAACCATCGGTTTGAGGGGGAACCAGAGTAAACATTGGAATGCCGCCATTTTTGGCGATGGCTTTTGTGATGATGTTTCGGTCGATAGCATAAGAGAGTGCCTGACGGACACGAAGGTCTTTGGTCGGGCCTTTACTAGTGTTGAGATAGTAGAACGTCGAGCCAAGTGAGGCACTGGTGTCCGCTACCTGCTCAGGATATTGTTTTAGAAGCTTGTTCTTTTGAGCCGCGGGAATCGACAGAGCTTGGTCAATTTCGCCGGCTAAGTAGCGGTTTAGTGCAACATTGGCATCTCCGATAGGTAGCCAAGTGATTTGGTCAAGTATGACGTTGTCAGCATCCCAGTATTTCTCATTCTTTGTCATCACCATTTTTTCGTTTACTTTCCACTCAGACACAGTGAAAGCGCCATTGGTGACGATGTTTTTTGGCTGAGTCCACTCGCTGGCGTATTTTTCTACCGTGGCTTGGTGAACTGGTGCAAGGACAGGGTGGCTCAGAAGTTTGATGAAGAAAGGCACTGGCTTTTCTAATGTCACTTCAAAGGTGTGATCATCAATGGCGCGCACGCCTAAGGTGTTAGCTGGTGCTTCACCTTTCATAATCTTGGCGGAGTTTTTGATGCCGGGAATCGCGGCAAACCAAGCATAAGGTGACGCGGTTTTCGGGTCGATAAGGCGCTGCCAACTATAAACAAAATCGTGTGCCGTTAATGGGTCACCATTCGACCACTGAGACTGACGCAACGTAAAGGTATAGACAGTGTTGTTGTCATTCGCTTGCCAGCTTTCAGCGATGCCGGGAATGGTTTTGCCTTGCTTATCCTGTACTGTCAGACCTTCAAACAAGTCTCGCCCTAAATTGAATGCTGGCTCTGCGGCCTGTTTTGCCGGATCAATTGAAGTGACCTCAGCTCCGTTGTTTAAAACAATCTCTTGTTTTTCAGCGAGCTGCGTTCCTGATGGGTATGGTTTGGCGAGAGCATTGCTGGCAAATAATGCCGTTGCAATAGCACTCAGTGCGATCAGTTGTTTTGCTTTCATAGCTTCCTCGTAATGTCCTTATCGGATGAAATCCCTATCATCTGAAGGGAGGGAAATCAGACACTCAAAACGACCGGATTGGCGAGTACTGCACAGAGTGAAAGGGATAGAGAAAATGTATATTGTATAAAATTTTTCAACAATCGATATTTAACATTGTTGTAACAAGGTGGCAGTTTCAGAGACTTGTGGTCTTATAAAATGAGACTGAGCGAAAAATGAACAAATGAAAGTCTGAGAATGAATCCAGAGTCACAGAAAGTTGATGAAAATGATACGTCAAACTCACTCTGGCCCTAGCGTCTGCGGAATTTTGCGGAAACCTGTTTAAGCAATTCCCCATCTATCTCTGGTAATCCGTCCGAGAAGTAGATCTGCTGGCCGGGCGTCATTCGAAACAGCACATTGGTGAACTCACCCTCTTCGTTAGCGCAATTCGCCGCGTGCATTTCACTGACTTTACGATCCACTTCCTGTTCGAGTTTGAGCTTTTGATCTTGGGTCAAACGCACTCTGGCCTGACAAACTTGAGAAAGGGTTGAGTCTTCCATCGAGATCGTTTCAAAACGGCGCTTGAATAAGGTATCGAGTGCAGTGCCTTCTTCCATGATAAATCGCCTGCCAGAGCGATACACGATCTTGTCGCCGTGCGCTTCCATATAGCCAAGGATTTCAGCAATGCTCAGGTAGAATCTCAGGCTGGTATCTGAGAGTTGATACTGATCGGCAATTTGCTGCGGTGTTTGGTTACGTGAGGTGATGAGGTAGATAAAGTCGAGAAGGTAAGGGTGTTCAACAAACAGAGCGTTTTGCTCATCAGAGAGGAACTGCTCATTGTCTCGTTGGAGCTGCACCGCCAATTGCGTCAGTTCCATCAGATCACTATTGAGGTGATTGGTGTACATCAGGATCTTGTCCAGACCCAATGAAGGATTATGCAAATGCCGCTTTATGGTCGAAAGCGGCACGCCGGTTTTGTCAGACAATTCGCTATAGCACAAACCTTCGTTTTTGATTTTCTGGCGGATGGCAGCCAGCAAAAATTCTGGTCCTATATGCATGGGTTTCAGCCCTGATTGTTTTGGCGCTCGATAAGCAAGTTGCGAATATCAGCACCAGCTTCGAGGATGTGTTTCTTCAACATGTATTTCGCTCCTTCAATATCACGGTTCTGGCACATTTCCAGCAGGGCAACATGCTCCTTGTCGGCTTTGACGATGCCTGATGTAAACAGAAGCTGCATACGAATATAG

>NODE_46_length_3376_cov_2.035465

CCTTAACACCTACTTTTAGTAAGTGTATAAAAGAAGTGCCATTCAAGTCTTGATGTTGCTCGAAAATTGGGCAATTAGCCTTGGCAAGAGCTAAATACTAACCTGATCTGGAAGGTGCCAATCGATCGTTGTTTTACCTTGGGCGTTAAGAATTTGATTTGCTTGCGAAAAATGTCCACAGCCGAAGAATCCACGATGCGCTGACAACGGAGAAGGGTGAGGCGCAGCTAATACATGGTGCTTATTGCGGTCGATGAAACGCCCCTTCTTTTGCGCATGAGAGCCCCAAAGCAAAAACACCACGCCAGACTGATGCTGATTAACTGCTTCAATGACTTTGTCGGTAAAGGTTTCCCAACCGGTTTTAGCATGCGAATGGGCTTTTCCCTGTTCGACCGTAAGAACAGTGTTGAGTAACAGAACACCTTGCTCAGCCCAGCTTTGAAGGTAACCATGCGCCGGAATCTGAAAACCATCAATATCCTGTGCTAACTCTTTATACATATTGACTAAAGAGGGAGGAGTCTTGATACCTGGCAAAACAGAGAAACACAGACCATGAGCCTGTCGAGGGCCGTGGTATGGATCCTGTCCCAGAATAACGACTTTCACATCATTGAACTCAGTCGCCCGAAAGGCATTAAACACATCTTTTGCCGGAGGAAAGATCGCTTTACCTGCTGCCCTTTCCGATTCAACAAAATTCAACGTCTGCTGAAAATAGTCCTGCTGCTTTTCAGCCCCAATGACGTCTTGCCATGTCAGTGACAGGTTCATGTTTTGTTCCTTCTACTATTTACGCTCAAGCAATTGTCGCAAAAAGTCATGAAGGAACAAAGCGATAAAGTGAACTAGCTTTTTTGTGGGATCCGATAATGCCCCTGCCCCGTCACATGGCGATTAGGAACAGGAAGACGTGTCGAACGTACGGTAGTATAAAAAGAAGTGTAAGGCATAATGGCATCCTTATTTGTCTGAGTACCTACACTCACTATGAAAAATTAATGCCACTTTTAAATAGTTAGACCATTTCCTTGAGCTGAGTAACATGATGCTGACGTATAAACTCAATTTCGTCAGGCCAACTTTCCGGTTCTATTGTTTCTAGAATCAATGGGATATGGTCAAATCGAGGATCTTTGGCGATATACTCAAAACACGACCAACCAATTTCTCCTTTGCCTAATGCGTGGTGACGGTCGACCTTCCCACCTAGGGCGATCTTTGAATCATTAATGTGCATTGCCCTTAAGTAAGGCATACCAACAACACGCTCAAATTCAGCAAAAGTAGCCTCACAAGCATCAAAGCTGCTCAAGTCATACCCTGCTGCGAAGGTATGGCAGGTATCAATGCATACACCAACTCGAGATTTATCCTTAACTTGTTGAATGATTTCAGCTAAATGTTCAAATCGCCAACCTAGATTGGTACCCTGACCCGCGGTGTTTTCGATCACTGCGATCACGTTTGGTACCGCTTGGTGGGCAATATTGATCGATTCCGCGATTCGAGCTAAGCACTCCTTCTCAGAAATTTTCTTTAAATGACTCCCAGGATGAAAATTGAGCAAGGTTAGTCCAAGTTGATGGCAACGATGCATTTCATCGATAAATGCAGCGCGAGATTTCTCAAGCTTTTCTTCTTCCGGAGCGCCAAGATTAATCAAATAAGAATCGTGTGGGAGTATCTTATCAGCGGTGAAGCCGAGCTTTATACAATTAGACTTAAATGCGGCGATGGTTGTTTCATCAAGCGGTTTGGCACTCCACTGGCGCTGATTTTTAGTAAATAGGGCAAACGCATTTGCTCCTATCTCTTTTGCCCTCAATGGAGCCTGTTCAACACCACCTGCTGCGGAAACATGAGCACCAATAAACTTGTTACCGTACGACTTTTCTAACATGTTGCTTTATCACTTTTTACTTTCAATATATGGCACGAATTACAGCAAGCTATTGGACTCATCTCAACACTCACACCACTTTTGTAGTAATATTACTACAAAACAAAAAATCCGAATCTATCGCTAAGACCACAAACCATTGCTATATATAATGTTATTGATTCAAATCAACATATTAACCACTAACTAAATAATGATACTGGTAGTTATTTGACTTAAATCAAAACCATCAAAGTAATTTCGGGTTATATAATACGCAGCTCGACGAGATTCGGAAATTAACACCAATAAAACCACTAATGTGGATAGGAGACTGTGATGATTCAAGGCATTCAAATTACTAAAGCGGCAAACGATGCACTACTGAACTCTATTTGGTTACTTGATAGTGAAAAAAATGAAGCACGCTGCGTGGCTGCAAACTCAGGTTTTGAAACTGACCAAGTTGTAGCAATCAACGAACTTGGCGAATACGAAAGCCGCGAAGTTGCGATCGAGAAAGCACCTCGCATCGAAGGTGGTCAACACCTGAACGTTAACGTTCTTAAGCGTGAAACTCTAGAAGATGCGGTTGCAAACCCTGAGAAATACCCTCAGCTAACTATCCGCGTTTCTGGCTACGCTGTTCGTTTCAACTCACTAACGACTGAGCAACAACGAGATGTAATTGCGCGTACATTTACTGAGTCTCTATAATTCAGTGAGTGGTACATTGAACATGATGACGGCGCCTTAATGGCGTCGTTTTTTATGACTTTAGAAAGAAGCAGTGCAAGCAAGCTTTTTTTCCGTTACATTAATTGACCCATTCCTAGTTTTCATCAGTAGCTATGTTACTTGAAGGCATCGAAACTTTATTAGTTCTTAGTAAAGCGAAAACCATGAGCCGAACTGGCAGCTTACTGTATATCAGTCAATCAGCAGTCAGTAAACGAATCGCCAATCTGGAAAAAAAACTCGGTAAGAAATTGATCGAACCTTCTGGCCGTTCAATCAAACTAACACCCGATGGGCAGGCTCTGATTGACAACATAGGTCCTTCTTTTAATGAGCTGCGAGGTCTAATCTATGAACAACAAGAGTTGGAAGATACCTCCCTCATCAAACTCGATAGCTCAAAGTCTTTGATTGCAGGGTACCTAGGCAAGACCATTGGCAACTACATTAAAAGCGATCAGTACATTACTATCACGACCAACCACACACCTCGTATTGTAGAACATGTTCAATCAGGTAAAGCAACGCTGGGCCTCTGCGCGGGTTTACTACCAGCCCACCATGGCTTGATGACCTTTCACTTATTTGATGAACCTTTCGCTGTCGTAAACGATGCACCTTTAAAGCAATTACCCGATAAGATCATCACCAACGATTTGACCAATCCCGCCAACAGCTATCAACTCATCACACTAGAAAAGCTCGGTATCAAGCCAATCATGGAAATGGATGCTTACACCGCTGCAGCCCAGC

>NODE_47_length_3375_cov_2.027896

TCCATGAGTTGATCTTGAGTGCGTTTAAATTTCTCGATGAGTGTTTGGATGGAGTGCTCACTGGTTTCAATTTGCATGGCTTGATTTTCAATTTCGCCTTCCACTTCATCAATCAGCGAGCGTAAACTGACAATCATTGAATGAAGTGAGTTCGCCATTACGTCTTTATCACTACTCGGAACAATATCCACATCATCAAGGTTACCTTTTGCTACGTGGTGTAACACATCGATATTGGCCTCTAGCCTATCCATTAAATGATTGAAAGAAATGGCAACGTCACCCGCTTCCGTACCGATTTCCACCGGCACGCGTTTTGAGTAATCACCGTCTCGACTGATGCCTTCAATCACTCTAAGGGTCTCAAGCCAACTCATCTGGACACCATGCTCTGTGACATTCAAACCAAGCTCTTCATCCTGAGGAGACACCCTGTCGAGATGAAAACACTTCAATAGTTGAAAACATGCATAAGAGTTAATGCCAGACCAAAACAAGATCACCAGAATACCAACAGCTTGCACCATCACGTCCCAGACCAACTCACCATTTTGCGTCAAGCTGAGTGAATAGCCGGTGTATATAACGCCTAAAATACCGCAGAACAAATGAACTGGGATGGCACCAACAGGGTCATCTATCTTAAGTGCTACTAGGATTCTTTCGCCGTAGTACACCGTAATGGATGCCAGAAAACCCAGCAGAGCAACCTCAAATGTATTAAAGATATTGGCGCCCGCTGTCACGATAACTAACCCACCCAGAACACTATTGAGCGTGACAAACACTGGCACGTGTTTGGACCGATGGACAAGAATCAGAGTGACAAATCCTGACACGGCACCACAAAGGACAGTGTTAAACAAAACGACAGGAACACGTGCATCGAATTGGTAGAAACTCCCACCATTAAAACCGAGCCAAGCAAACACCATTAAAAACACGCCGATCGTAACCAGAGTGTGATTATAGGAGGGGATTTCATTTACTTGTTGTGTGCCAATGAATCGGTCTTTGCGTGGCCCTATGATCATCGTACCGACTAAACCAATCATGCCACCTACAACATGAACCGTCGCACCTCCAGCAAAATCTTGAAAACCAAGTATGTAGAGCCAAGAATTAGGGTTCCAAGTCCAGTAGGACACCACTGGGTACGTCAGTATACCAATGAAAGCCGACGTATAAATATAACCGCGAAAACTCATTCGCTCGGCAACACAACCTGAGACAATAGTCGCGGCGGTCGCAACAAACATCAGGTTAAACATCACAACGGGAAAGTCTTCACTTGTTAGAGGGAAAGTGTCGAATGAGAAAAGCGAGCTGCCCTGTGCTAAATGGAAACCAACGAAAACATAGACGACAGACACCACTAGAAAGTCTGCCATGTTCTTCATCGCGACATTGACAGAATTCTTCGCGCGAACACAACCGGTTTCGATTAGGGTAAAGCCAGCTTGCATGAAGAATGCAATACAAGTGCAAACCAGCAACCATAGAAGAGACACAAAGTCCGTCCCAGCCATACTTACCTTTAGAGTTATACTTCCCTACTTATCAGTAAGCGTAGTCGATGACTACGAACTCTGATACATATCAGTTATAAATGTTAAAACGCATATTTAAGCCGTGACTGCTCTTTACCAAAAATTGACCAATAAAGTGTGGCAGCCTGTTTAAGAATTCACTAAGATAGTCGTTGTTGTGGGCACGCATCTCATCCCACATTTCCTATTTCGAAGCGCCTAACTCTGTTTTCTTACCTTTGAAATAGGCTCTCTCATAACTAGAAGCAATTTGTGTATTCATATGGACCAAGATAAGTTTACTCATATCTATCGCTTACCGACTGCGACACAGATACGTATAGCCAAATGGCAGCAGACATTTAATGGCACCTCTGATCTGGTCATTCATCAGGCGATTGAAACCCGGAATAAGCAATATCGTAAGCCTAACTTCTTTCCATCTGGTTGGAGCGTAACGCTATTCGACAAAGAGGATATCTCGATCACCAATCATGGTAAGTACATACAGACTGCAATGCGTACCATGCTTGATCGAAAAGTGTCTTATAAACGGATCTACTTGACTCGCCTGCCGCTAGAGGAAGCCGAGCCCGCTTTGAACAACTTTAAGTTGGAGTGGATCTCAAAGCACAATCGTGTTGCACGGAAGTACAACCAAATCAAGAAGAAAGAGTTTCTTCGCTACGCCCGTGAAGAAGAAGAAACTTTGTACCCTTCTATCCCCAAAGGTGAGTTTGACCGAGCCTTGTGGAACAGACTGGTTATTTCAGAGCTGGGGCCACAAAAGAAATTCGATAATCCTTATTTCGTCAAAAAGGCCAAAGTTTAAATACAAAAGCAGCCATTTGGCTGCTTTTTATCGCTCGTATTTGTCCATAATTTCCTGATATAAAGACTTTCCAGACTGACCTTTGGTGTTCTTGAGCATATCAACACCCTTTTGCAGCTTAGCAAGTACTTCCGGTTTAACATCAACGTTAAAAGCGTAATAGATCAGACCTTCCCTCACAACATAAACAGATTCAAACAAACTCGGATCGACCTCAGCTTGCTTAGACCACCAAATTGCTGCGCGATGGGCGTAAACCAAAAGGTCAATACGCTTTTTCATTAGCTGCTCCGCCAACGTTGTCACGTAATTGGCTTCCTGCATTGAGTTCCTTGGCAAACCCATCGCTAGCAGGGTTTGCTCACCGATATCATCTCTGATTACCCCGATACGGTAATTGGCCATATCCATTGGCTTTTCAATTGTAATGTCCGCATCTTTTCGCGCCAAAACGACAATTTTTATATCAGCGATAGGGCCAACCCAATTAAACAGCGTTTCTCTGTGTTCTGAACGTGTCGTAGAAAACAACACCGAATTATCATGAGTTAACACCGTTCGATATGAACGAGCCCAAGGTTGTAAAGTCACCTGCGACATATCAATCTCTTCCCCGACCATCTTACTTGCGGCAAGGAGGATTTCCACGGCATAACCTGTAATTTTATCGCCTTCCATATAATTGGCAGGGGGATAAGACTCAGTAAAAAATGACAGCTCACGAAGGTCATCGGAGTATCCGGCTACAGATACCGAAGAGAAAGAAGCAGACAACGCAATGAGTAGTGACAGAAGAGAATACATTCGCTTCATGACAAACTCCCAGTTAGTGAGTACTTTATAAAGTTAGCCCACACAACGGGAGTTCTAAAGAAAAATAGCGGATTTATACCAAGCCGTTGCGCTGTAATCCCCTACGAACTCCATTGCACATCTTACCAAAGCGGCGAATTTCATCGAATTGAGGCGTAATACCCCGCTCCATCGCCGATTCCCAATAGGTCAGTTCAGAAT

>NODE_48_length_3345_cov_2.652387

GTCCCCATGGTTGCACCAGCACCCGGCAGTGCACCAAAAAGGCCAGAGATCATATTCGCCAAGCCTTGACCACGCAGTTCTTTATCCGAGTTATGCTCTTTACGCGTCAGTGAATCGCCGATAACGGCGGTCAACAGAGTATCAATACACCCAAGTGTTCCCAGCACTAGCGCATCAATAACCATCTCGGTAAACATCGAAGCATCGATATGCGGAATCACTAGTGACGGCAAACCTGCAGGTATCTCGCCAATGCGGCGGATCGCCTCAGTATCAAAGATAATAACGGATAGCAAAGTAACAGTAATTAATGCCACTAGCTGAGCCGGAACGTATTTACGGTATTTTTTCGGGAAGAAGAACAAGATCCCTAAGGTCAGCAGGCCAAGAAATAGCTCGCTAAACTTCATGTTGGCAACGGTGTCAGGGAGAGCGGATAAGGTTCCTAATACACCACCTGAAGGAGCAGCATGACCAAGTAGTGGCGATAGCTGTAGGATAATCAAGATGACGCCGATTCCTGACATAAAGCCAGAAATCACACTATACGGCATCAAGGTAACATACTTTCCGAGCTTCAATGTTCCAAGTAATATCTGAAACGCACCGGCCATCATCACAACCGTGAACGTCATCGCCATCCCTGTCTCAGGATATTTGGCCATCATACTGGTAAGGACGGCTGTCATTATCACCGTCATTGGACCTGTAGGCTCGGAAATCAGCGAATTGGAACCACCAAATAAAGAGGCAAACAATCCGACCAAGATAGCGCCCCATAAGCCAGCTTCAGCGCCAGCACCAGAGGCAACACCAAACGCCAATGCCAATGGCAGAGAAATGATCGCGGTGGTGACACCACCAAAGACATCCCCTTTTATATTGATATCTTCAAAACGACTTCCAAACACAATTCAGCCCCACTGCTTAATTAATGCTACTTTGATACTCGGTAATGAGAGCTACTCATAAAATAGACGGCCTTCCGAATAACCCCATCAAAAGTGGTTAATTTGGGCAAACATTGACGCCATCTTCTGAGAAACACCATCGAAAATTAAATTCTAGCAAATACCCTAGGTCAAAGTTAGTGCCATTCTAGTTCCAACAGGCACACGTTATAACTCTTTGTTAGGTTCGCTACTAAGTTTATGGTTTTTAACGATAGAACACGTCAGATGAGTACCTTTTTACACAGTTTTGACAGGAACGAAATTGTAACATTGTGTATAGTAGCGAAAGAATTGTTAAGGATTGATTGTAAGATGCCGGAAATAAAACAGCTTTTTGAAAACAACTCAAAGTGGTCTGAATCCATTAAGGCTGAACGACCAGAGTACTTTGCAAAACTTGAAGAAGGCCAGAACCCTGGATTCCTTTGGATCGGTTGCTCTGACAGCCGAGTGCCTGCTGAGCGCCTCACCGGCCTGTATTCAGGCGAGCTCTTCGTTCACCGAAATGTCGCAAACCAAGTTGTCCATACGGATCTGAACTGTTTATCTGTTGTTCAGTATGCAGTGGATGTACTGAAAGTAAAACACATCATCATCTGCGGTCACTATGGCTGTGGAGGAGTGAACGCAGCAATCGACAACCCTCAGTTAGGGCTGATCAATAACTGGCTGCTGCACATTCGTGATAACTACCTCAAATACAAAACAGAAATCGAGTCGTTGCCACGTGAGCAGTGGGGCAACAAACTGTGCGAAATTAACGTTGCTGAACAGGTATACAACCTTGGCAACTCAACGATTTTGCAAGGAGCATGGGAACGCGGCCAGGAGATTCAAATCCACGGGGTTGTTTACGGTATCGGCAATGGCAAGCTGCAAGATTTAGGTGTACGCTGCTCAAGTAGAGAAACGTTAGAAAGCAGTCATCAGGAAGCCTTAGATAAAATCCTATCTTCACCTGTACTAGGTTAGATAACAAAAGACCCGCACAAGGCGGGTCTTTTTACATTCGTATTAGATGGCTTCCATTTATTCCTGAGGAACCACTTTACCAATGTAAGGTAGGTGACGGTATTTCTGCGCGTAATCAATACCCACACCAACAACAAACTCATCTGGGATAGCAAAGCCAACCCAATCGACTGGAACTTCCACCTCACGACGAGACGGCTTATCAAGCAGAGTACAAATCGCAATTGACTTAGGTTCACGCAGAGTCAGGATCTCTTTGATTTTATTTAACGTGTTGCCCGTATCAATGATGTCTTCAATGATCAGCACATCTTTGCCTTTGATGTCATCATCCAGATCTTTCAGAATGCGCACATCTCGCGAACTTTCCATCGTATTGCCGTAACTAGAAGCAGTCATGAAATCAACTTGGTGAGTCAATTCAATCTTTCGAGCCAAGTCTGCCATAAAGACAAATGAGCCACGTAGCAGGCCAACCAGCACCAAATCTTCGCTGCCTTGGTAGTGAGCGGTAATCTCTTTACCTAATTCACTTACGCGTTCCTGAACTTCTTGCTCAGAAATCATTACTTCTACTGTATGTTTCATACCAATCTCATTTTCTGCATTAGCGAATACTCACTAAATACTAGTTTATATACCGCCAGATGATTTTAAGCGCGTAGTTTAACACCACTATGCAGCAGGAGAAACGTTTGCTTGATTAAAGGCTGCAATAAACATATGCAACTACATGATCATTAGCACGGTATTAATAAAAATGATTATTAAATGAACTGAAATCGTTGACCTAATTCATATGCACCATTACACTCATTACTGCGAAAGCAATAATAAAATACTAATTAGGGCAAAAGCCCACATATACAACTCAATTGGCAAGGAAATATAACTATGGATTCTATAGCTAAGAGACCGCGTACGCGTCTTTCACCACAAAAACGCAAACAACAACTGATGGAAATCGCATTAGAGGTGTTTGCTCGTCGTGGTATTGGTCGCGGTGGTCATGCTGACATTGCAGAAATCGCTCAGGTTTCTGTTGCTACTGTTTTTAACTACTTCCCAACCCGCGAAGACTTGGTTGATGATGTACTTAACTACGTAGTTCGTCAGTTCTCAAACTTCCTATCAGATAATATCGATCTTGATATTCATGCAAAAGAAAACCTACGCAACCTAACTTCGGCAATGGTTGACCTTGTTGTCAACGACTGCCACTGGCTTAAAGTTTGGTTCGAGTGGAGTGCATCGACACGTGATGAAGTATGGCCACTGTTTGTTACGACAAACCGTACCAACCAGCTTCTAGTTCAGAACATGTTTATCAAAGCAATCGAGCGCGGTGAAGTATGTGATGATCACGACCCAGCAGACCTAGCGACACTATTCCACGGCATCTGCTACTCACTGTTTGTGC

>NODE_49_length_3335_cov_2.066605

CGTATCAGCCTGAGTGGCAATAGATTGCACTTGTGACGCAATGGCCTCCAGAGCTGAGCCTGCCTCTTGGGCTTTATCCAATACCTGATTCGCATTGCTTTGGCTCTGAGTCATAAGATTCACGGCGTTACCTGTAGAGGTTTTCAGCTTCTCGATGATCGATACGACATCCTGAACAGACGTCTGAGTGCGATGCGCTAACGTACGGACTTCATCAGCTACAACAGCAAAACCACGGCCTTGTTCTCCTGCACGTGCTGCTTCAATTGCAGCATTCAGAGCAAGCAGATTGGTTTGTTCGGCGATTTCATCAATCATCTTTGTGACAGTTTGGATAGCTTCACTGTCTTTGTTTACCTGTTCAATGGCTTGCGCGGAATTATCTAGCTGATCATTCAACTGCGCGACATCAATACAAACTCCTTCAACTTTTTCTAGCCCCGACTGGCTGTCTTCGTTGGTGAGACGGACGTTTTCAGCAATAGCTTCTACTTGCTCGGCTACTGATTGTGCGGAAGAGGCCATTTCTTCAATAGCAGTTGCGACTTGCTCAACCTGTTGCTGCTGCAGGTCTGATTGGCTGAGATTCTGGTTGGCATCCTGAGATACACTACGCGACGCTTCCTGAACTGCGTCACTCGTAGAGCGAATCTCGCTGACCAGGGCATTCAGTTGTCCTGCCATATTGGCGACGCCGTTATTCAGGTTAATGATTTCATTCTTAGATGCTTTGCCTGTCGCTGGTATATCTAAACTTACTTCACCTTTTGCTAGGCGAGACATATACCCGTTCAGTACCGTTAGTGGTTTGAGGTTACGGTTCAGGAAAATAGTCAAAATGATGAAGGTGGCAATCGCCACTACCGTAGCAATCGTAATGATCAGTTTGAGCAAGGTGTCACTGCCTTTGGTGACTTCTTTGATGAACGTCCCCCCAAGCAGCTTCCAGTCCCAACCAGGTACTTCTGTGTAGACTAGATATTTCTCTCCAACCGTACCCTGATACTCGTATGGGTAACGGATCAGACCACTTTTCTGTTCGAAAATCTGATGAAAAGGTTTATTGCCATTGTAGTCGGCGACATCGATGATGGGTGGAGCGTTTTCATCATGAATTGGGTGCAATAGGTAGCGACCCAAATTGTCCTCTCGGTTATCTACTACAATGCTATATCCTGTATCGCCCCACTTTATTGAACGTAATGATTCAAACAGATCTTGGGTCGCTTGTTCGACTGGTAGTCCGATGAAGGTGATGCCACTCACGTTGCCCTGATAGTCTTTGAGGGGGGCGTAATAGGTAATGTAGTTTTCGCCAAAAAGCTTAACTTGAGCGTAGTATGGTTGGCCGCTCATTAGCTTGTTATAACCCGGATGGTTTTTGCCTAATGTTGTACCAACCACGCGATTACCTGATGGGTTTTTGAGTGAGGTTGAAACTCGAATAAAGTCACCATCTAACGGAGCAAATAGTGTGGCTACTGCTCCTGTATCACGGGTAAAGCTATCAACCAGTTTCTCATCATTGATCAGGCTCTCACCATACTGAGTGACATTTAGAACCTGTTTACCATTGAAGTCGACGGTGTAGTCTTCAACATAGACGCCTGCCAGATAACCGTTTCTAAAGGTGGATTCCAAAACTTTTGCGGTATGAAGGTAAGCGTTAAATTGGCCAGCAATGGTCATTGCCATTGCTTCGACCTCTGATTGGTGGCCTTTCAGTGTGGTATCAAGGAGCACTTTGGAGGCATTACGGTAAACGAGGGTAGCGGTCCCTACAAAAGAGACCAACAAGCAAAGCAGTATCACCAACTTTAGTTGAAAGCCAACACTTTGGTTTTTGAATTTCTCGAACATTTTTTAATTATCCTGTCTTTGGAGACGTGTCTTATCGCTGAAAAATAGAGCTCTTATTGTAAATTAAGAATAATAATAACTACTTATCCATTTGTTAAAATTGATTCCTATTAATTATTCAAACAATAAGCAAGTCAAAAAATGCGTTTGTACAGCGGGTGTAGAGTAAAAGACCTGAGCGGGCATAGAATAATTTCGGGAGAGAGGTACTGCAGGCTAATAGCCTGCAGAAAAAGAGTTATTGAGCGGAAATAGAGGTGATAAGTTGATGTGACTCACCAGGTTGAAGTGTTTTTCCTAGTTCAATGCTTGGTGCGTGAAGAGTAGATTCGACACACATCATTGTTAAATACCCGTCATTCGTCATATCTGACATAGATTCAGCACCTTGTGCCCATGGGTTCCATAATACAGCCGAGTTGTGGCCTTGGTTCTCCACGCTTAGTGTTCGATCCAACACTGGATCTTTCACTAAAATTTGCGCTTCTGGCTGAGTATAAACGCGGTCGATAGTATCAGTAAGTTGAAGAACTTTGTCCCCTTGGCAGATTTCACCTGCTTTTAGGCTATCGATGTATTCAGGACCCATCCCAGTAGTTTGTGCTTGTTTGATGTTGCCTACGTTAAGATACGTGTGCAATGCCCCTGAAAATGCCCAAGGTGTCGTATCAGTATTGGTGACGTCGAGCGTCACTTTAAGCTCGTTGCTGACTTCTATTATTAAACGCGCATCAAATTTATGTGGCCAGATTGATAGTGTTTGCTCTGAAGGTTTCAGTCCGAGCTCAATCACAACTCCTTGTTCATTTTCACGATGTTGTAGCAGTGACCACTCACTGGTACGAGCAAAGCCATGTGCAGGCGCGGCAATGCGGCCAAACCAAGGCCAGCAAATTGGAATGCCACCACGAAGCGCTGCCTTACCATCAAAAATGGCGCTGTCGCTCATCCAAATCAGATCTTCTTGGCCTGCTGGTGTGAACGATACGACATGGCCACCGTGTAGCGCGATGCCTGCCGTTGCTTTTTCATGAATAACGCGGACGACTTTAACCTGATCAACTTCAACGATAGTGACGTTGTCAGATAACACGGTCAGGGCTGGGAGAGTTTTTAAATCCATGTGTAGTTTCCTATCGCTGGGGAGAGCTTTTCGACATACTTCGCGCATTATTTGCAATGTCGAGTTTGTATAACCTCAAAGTCCAGATACAAAAAAGGCGACTCTAGAGCCGCCTTTTCTTTAACGAAGTTATTCGCTAATTGCTAACTATCTAATTACTTAGAGATGTGAGCGATTAGGTCTAGAACTTTGTTTGAGTAACCGATTTCGTTGTCGTACCAAGATACAACTTTAACGAAGTTGTCAGTTAGAGCGATACCAGCTTTAGCATCGAATACTGAAGTTTGAACTTCGCCGATGAAGTCTTGAGAAACAACTTGGTCTTCAGTGTAACCTAGAACACCTTTAAGCT

>NODE_50_length_3329_cov_2.060886

GCCAGAAAGCGACATAGAACTAATGGAAGAGATTGGTCGTCGAAGAGGCGCGCTACGTTCTGGCGGACGAGTCGATCTGCATAAATGTTCGGAAATCTTACTTCACGAACTACGTAATGGTACCTTAGGTCAAATAACACTTGAGTTGCCTGAAATGATCACCAAAGAGTTGATCGAAGTAGAGATCGAAGCGACACGCAAAGCTGAAGAGAAGGCCAAGAAAAAAGAAGAGCGTCGTAAACGCTACTTAAAGAACAAACGTTAATCACAACAACGGCGGTATCTACCGCCGTTTTTTATTCTAGCTTCCACGCTAACCTTTGATATAGTCAAAGCATTGTTATCGACGAGTTTTGCACAATGAAAATCCCAACCAATATCATTACTGGTTTTCTGGGTACCGGAAAAACCACGACAATCCTAAACCTACTGAAAGAAAAACCTGCTGACGAAAATTGGGCAGTTCTGGTCAACGAATTTGGTGAAATTGGTATTGATGGCGCAATGCTGACCGAACAAGGTGCGATGATCAAAGAAGTACCCGGTGGCTGCATGTGTTGTGCTGCTGGAGTCCCAACTTCCGTTGCTCTGACTGCTTTGCTTCGCACGCAACCCGATCGCCTGATTGTTGAACCTACCGGCTTGGGTCACGCCCACAAGATTCTCGCAACCTTAACCTCTGCTCAGTTTCGAGATTACATCGACCTGAGAGCCACCATCGGCTTAGTCGACCCACGTAACTTATCTAGCCCTAAGTATCTGGAGAATCAGAATTTTAACGACCAACTCAACATCGCAGATATTGTGATTGGTAATAAAGTAGACGAATGTCATGTCAGTGACGTCGATGCGTTTAACGACTGGGTTTCTGACCAAAATCCACCCAAGATTTACAGTCAACTCGTCAAGCAAGGGCGTTTCCCTATCGAGCTTCTGGACAACCAGAGAAGGGATCCATCGGTTTCAACGGATATTGCACAGCACTATCATGAACATGCTGAGGCAGAACCTCAATTTGCGCTAGCGCCAGATCAGATAATGGTTCGCAAAGAAAACAAAGGCCAAGGTTACTTTAGTTGCGGCTGGTTATTTGGTGCAGAAGTCACGTTCAATTTTGATGACCTATTTTCTATGTTATCTGACTTAACTGCCGAACGCGTCAAAGGCGTGATGAACACCGACAGAGGCTGCTATGCGTTCAATGTAAGCCACGGTGTTGTCTCTGTTAATGAGATGTCATTAGAAGGCTTTGAAAGCCGTATGGAAGTCATCGATAGTCAACTTCTACCATGGGATCAATTGGAAAAGGTACTTAAGCAAATCGCTTCAATTGACTAGCACCCTATAAAAGACAAAAGGCGCACCATACAGTGCGCCCTATCCCTCGTTACTCAATCGTGAGTCTGCGAATCAAAACCGTCTGTTGATGCGTCCTGATCTTAATACGAGGATCTCGAAATGATTTGTTGAATCCATCCAAATTGTAGGTAAGGCAGAAAATCCTCCATGCGGGTCGGTTCAGATTGCCTTACCATTTAAATATGGTTCAAAACTCCCAAAGTGCAAGGGAATAAACGTGATCTCTTGCCAATTCTCAGGCTATTTGTCGATATCTCTGAATCTCCATATTTGAAAATCAATATTTCAATCTTGACGCTATAGTTAAGCCTATACCAGCGTATAATTCTTGCTAGAGACATCACCATTTCTAATAAGAAAATAGATAAAACGAGGTTGATCTTTTTATGACCAAAAGAGAGAAAATTAAGCAGTCCCTTTTAGCCAGTATGCCAAAGGATGCTATTAACCAGTTTCTCTCTAAAGATAAAACGCCCGTCTCTGTTCTCATGCTTTCTTTACTTGTCGGTATTCTTGCGGGCATCGTTGGAACCTTCTTCGAAATTGCGGTTCATTTTGTCTCTGAGACTCGGACTGACTGGCTCAAACAAGCGATTGGTAGCGCATTACCGCTGTGGCTCGCTGCATTTTTAATCAGTGCCGCATTGGCATTTGTTGGCTATTACCTCGTTCACCGTTTTGCTCCTGAAGCTGCGGGATCTGGCATTCCAGAAATAGAAGGCGCGATGGATGGCATACGCCCTGTTCGTTGGTGGCGTGTTCTACCTGTAAAGTTCTTCGGTGGTATGGGTGCACTAGGCTCAGGTATGGTTCTTGGACGTGAAGGGCCAACGGTTCAAATGGGGGGAGCCATTGGCCGCATGGTGACGGATCTCTTTCAGGTAAAAAATGATGATACCAAACACTCACTGCTTGCATCGGGTGCGGCAGGAGGTTTAGCTGCAGCGTTTAACGCTCCGTTAGCCGGCATCATGTTTGTAGTTGAAGAAATGCGCCCCCAATTTCGATACTCACTCATTTCTATTCGTGCGGTTATCATTTCAGCTATCTCCGCCAACATTGTCTTTCGTTACATTAATGGTCAAGACGCGGTTATCACTATGCCGCAATATCACCCGCCAGAGCTTGCCGCCTTATGGCTTTTCTTACTGTTAGGTGCGATGTTTGGCGTGTTTGGAGTCGTGTTTAATCGCCTAGTTACCTTGTCTCAGGACTTGTTTGTCAAACTGCATAAGAATGACCGTAGACGCTACCTGATCACCGGAAGCTGTATTGGCGGTTTTTTTGGTTTGATGCTGCTTTATCTACCAGAAATAACCGGCGGCGGCATTGGCTTAATTCCTACCATTACTAACGGTGGCTACGGAGCCACAGTGTTGCTGCTTTTGTTTGTTGCAAGAATTCTCACCACACTGATTTGTTTTGGCTCTGGAGCGCCTGGTGGTATCTTCGCACCAATGTTGGCTTTAGGTACACTGTTTGGATATGCGTTTGGATTAATCGCCCATGGTTTGATGCCTGAACTGATGATTTCTCCGGGCATGTTCGCCATTGCAGGAATGGGGGCATTGTTCGCGGCTACCGTTCGCGCTCCAATTACGGGCATATTATTGGTGATCGAAATGACCAACAATTACTACCTGATATTGCCACTGATCATTACCTGTCTCGGCGCGGTTATTTTCGCTCAATTACTTGGAGGACAGCCAATCTATAGCCAACTGTTACATCGAACATTAAAAAATGAGAAGTTGAGACAAGAAGACCTTCCTTGCGAGTCAAAATGACAATTGCACAGCAACCGATGAGCTTGATATGATCCCTCGCCCTAAATGATCGGTAGCACGGATTTGCTTACTCATTTAGTCCCCAATCATTTGTTTCAAGCTAGTCAGGATTAAGCCGTTGAACTGGAGACGCTTTACACAATTTCAGAGTGTACCACCATCACAAGCAGCATTAGCGCTAGG

>NODE_51_length_3328_cov_2.566595

GCTCATATCCATATCGGCAAGCGCTTCGTCTAGCTCGCCATCGTCCCCGATGCCCGCGAATGGGTCTTCGCCATCTTCACCTTCAAGATTGAAATCGAGATCGTTATCTTCTAAGTCGGCAAAGACATCTACTTCGCCATCTTCTTTACCTTCATCTAAACCAGAATCATCCGAGAACAACTCTTCTTCATCGTCGGTTGATTCACCAAACAAGTCATCATCAAGCAGTAAATCATCATCCACTTCTTCATTAAGAGAATCCGGAGTGATTGGCGCAACAGGTTCAGCGGTTTGTTCGGTCGCGGTTTGTGCTTCTGAGTCTTCTTTCTTACGGCGACCCAGCAACATCATGATCAGCAAGCCAATCAGCAAGCCTGGGATGATAGCCAGCGCAGCGACAAGCCAAGTATTAGAGAGCAGTTCATCGAGCGGAGAGGGTTGCATTTTCTGCTCTTCGGCAAGTCGCAGACGTTCCGCTTCGAGTAATTTTTCTACCTCATTGCGAATGCGAGACTCATCACCCAGCTCATCTTTCAGCACATCCACTTCGGACTGAACATCAGCCAACATCAAGCGCAACTTATGATTTTTCTCTTCCAGAGCCATCAACTCGCCTTCTGAACTTTCGAGTTGTTTCTCCAAAGTCGAAACTTGCGCTTTTTGCTCTTGCTTGGCTATTTTTGCTAAATCCGCATTTGGCTGCATTGCCGGTTTGGTATCATCCATCATTGGCTTTTCAGCGTCAGATGGTTTCATTTCTTCAGGTTGTTGCTGAGGCGAGGTTTGCGGGGTTGGGCGAGAAACCGCAGGAATATCCGTCAAGCGTGCTTGATGGGCCGCCATAACATTGACAGCTTCTTGTGTCGAAACGCTTTGAACTTGAGCCAACGAAGGAATTCGTAGCGTACTGCCTGGAATAAGCTCGTGGATGTTTTGATTTTCAAACGCTTGAGGGTTGAGCTGATACACAGCAAGTAACGTTTGTTGTACAGAAACGCTAGCTGATGGACGCAAACGGCTGGCAATAGCCCAAAGTGTTTCTTGAGAAGTAGTTGGTCCATAAAAGCGAGAAGGCTCGTTGTTACGCAGATCTTGGGGGACAAAATCAGAATATTGCGGCGATGCTTGGATCTCGCCGTCAGGTCCCTTAAGGCGAATAGTTTCTGCACTAACAATCGATGTCTGAGTCACGGCTACTAATGCAACAGGCAGCAGCAGACGCTTAAAAATTCGAAGCATAGAAGGCTCAGCTATGTGCTCAATAAATTTTAATGTAATGATCTGTTAAATATATCGGATAAACACGCTAAAACTATAGGTGCATAGCTAAAAAATGTGTGGCTGCCGCCATATTCGCACTAATCTCACACTAAATTTAGGAATCTGATTAAAAAAGCCTCACACGATGGTGAGGCTTTGCTATAAGTGTTAATAATACGCTCAGTAGTAATCGCGAATCAGAACTTCTGCAATCTGAACCGCATTGGTTGCTGCGCCTTTGCGAACGTTATCTGCTACAACCCACAAGTTGATACCACTATGGTGGCTGATATCGTTGCGAATACGACCAACCATCACGTGATCTTTACCACCCGCATCACGAACCTGTGTCGGGAAATCTTCACCATGGAACACTTCAACACCCTCGGTCTGCTCTAATAAGTTAGCCACTTCCTGAGCATCAATAGGTGCACGAGTTTCGATATGAAGTGCTTCCGCATGACCATAGAAAACAGGCACTCTCACACAAGTCGGGTTGACCGTGATTGACGAGTCATTAAAGATTTTCTGCGTTTCCCACACCATTTTCATCTCTTCTTTGGTGTAGCCATTTTCCATAAACTGGTCAATTTGCGGCAGACAGTTAAAAGCAATTTGCTGTGAGAACTGCTCATTTTCAGCCGGCATGCCATTAAGCAGCTTAGCAGTTTGGCCTGCCAACTCATCGATACCAGATTTACCAGCGCCAGAAACGGATTGGTAAGTCGAGACATTAATACGATCAATACCCACGGCATCATGAATCGGCTTAAGTGCTACCAACATTTGAATCGTCGAGCAGTTCGGGTTAGCGATGATATTGCGGTTACGGAACTCGGCAATCGCTTCAGGATTAACCTCAGGCACAACCAAAGGAATGTCATACTCATAACGGAAGTTAGAGGTATTATCGATGACAATCACCCCTTCATCGGCGGCGATTGGCGCCCACTTAGCCGATAGTTCTCCTCCAGCGGAAAACAGGCCGATGTGTACCTGAGACCAATCAAAGTTCTCAACGTTTTCGACTTTGATGGTTTTGCCGTTAAAACGGTAAGTCTTGCCTTCACTGCGTTCACTGGCTAGCAGAAAAAGGTCACCAACAGGAAACTTACGTTCCTGAAGTACTTCGAGAATTGTTTCACCGACTGCGCCGGTCGCACCTAAAATGGCAACGTTATATTGCTGGCTCATTGACTTACCTCAATTTGAAAACCTAATTTCGCTAGTGGTTGCAGGTTAGCCTCTGCATTTCCTCTTAGCGTGACTGCACTGTATTCACGGCGATCCCAGTAGTTCTTACGCATCAAGTCAAACGCACCGGGTTGACTGATTTCACGACGGAACAATGCATCGTCTTTACGCACATCATAGACAAGTTGCGTCAGATTGTGCAGCGTGGCTTCTTCCCAAGCTCTGTCCAATACTAACTCCGGCACTGGAGCTGTAGGCAGAAGTTCACTGGCATGTGCGCGTAAATCGTTATTCAGAAACTCGCAGAAGCTATTAAAAATCATCGTTGTTCCACGAGCTTTCCCCTCTAAGCCATAACCGGCGACATGGGGGGTTGCGAATGCTAGCAGAGGCAGAAGCTCCATATCAACCTCTGGCTCGAATTCGAACACATCCAATGCCGCAATAAAGCCATCTTGTTTTCTCAGACGAGCTTTCAACGCAGCGTTGTCAACGACTGGCCCGCGCGCCGCATTGATTAAAATTTGGTCACCACGTAACTTATTCAACACAGTTTCATTGATTAGATGATGAGTCGGAAAATCACCATCACGGGTGATTGGAGTATGAGTCGTAATGATATCCGCTTGCTCAAGCAATGCATCTAACGGGGTGAAATTACGCTCATCTCCCGCCGCTTGCTTAGGTGGATCATTGATAAGCACCTTAATACCAATGCCTTCCAAACATTGCTGTAGATAACTACCCACCTGACCTGCGCCAATAATTCCAACGGTTTTATCAAATACTGAAAAGCCTTGTTGCTGCGCAAGTACCATCATAACGCTGAACGCATACTCAGCTACACCGACTTTGTTGCACCCAGGTGCCGCGG

>NODE_52_length_3322_cov_2.488136

GTTATGGGAGTCAAGAACAATCAAACGGTACCCCAATTGATTAGCTCGTTGCTCAGCCCCCTCTTTCATCGTGACAAAAAACGGATTGTTGAGACTCGAAATCACCAACGCTAACGTCTCTTGAGCCTGAGCAAATGACGCAATAAGTCCAGCTAAAACGCCAACGGTTCTAATCACATAATGTTTCATTTAGTTCTCCTTTAGTTAAACGACAAAGCCTGAAACGGCGTCCTTTTGCGCAAGTGTCAAAGACATCTGACAGGTCATTCTCTCATGCGTTTGTGAGGCGGACTCCGATACCCCCTGTGACAACACCTTAATATCATTGGTGTTTGAGCTAACTTCCAATGCCACTGTCTTTTGCTGGTCTGCCGCCATTTCTGTGTCGTTGATCAAGTCCGAAATCGTCAGAACAGACATTTCAACTTCCTCGAGCATCTGGTGTGACGCCTCACCCCGTTTGACGGTGGTATCTGCCGTGATCAAACTCTGCTTCATCACATGACTGGCAGCCATCGCACTGTCTCGCAGTTGGATGACGGATTGCTGGATCTGCCGTGTGGAGTCCTGAGTTCGTTTGGCCAAGGAACGGACTTCGTCGGCGACTACCGCAAACCCGCGACCAGATTCGCCTGCTCTAGCAGCTTCAATAGCGGCGTTCAGTGCCAAAAGGTTGGTTTGTTCTGCGATATCCTCGATCACCGACATCACGTGCTCAATGTCGTTTGCATTCGCTTGAACCCGTTCAATAACATTGGCCGCATCTTTGATTTCCCCCGACATCGATGCAATCGCTCGACGTGTTTCATGCACAACGGTACGTCCTTGTGAAACAGATGTTTGCGCTAACTTTGCCGACTCCGCCGCTTGGTATGCAATATCAGCCACAGAGTGAGAAGAGGCGGAGAGCTGCTCCATGGCCACTGACAACTGTTCCAATTTGTCAAGCTGGCGCTTTGCGACATCGGACACACCATTGGCATCACTAGAGGTTTGCTGTGCGCCTTCATGCAATTGCTCATTGAGGCTCTTTACTGTGTTTATTGTGCCGTGCAATTTGTCCGCAAATTGGTTGACATTAAGTGCAATTTCAGCGAATTCGGTCTCCTTAGGTAACGTCAGTCGACGAGTTAAATCACTGCTCCCCGAAGCCAAGTCCGTAATCATGCTGGCCAGACTCTCTAAAGGTCGACATAGGTAGCTCAATAGCCACACCATAAAACCTGTCCCTAAGATCACTATACCCACCGTCAAAGCTCCCATATCACGAGTCATCTGGGTAAGACTCGCGAACGCCACACTGTTAGGAGTAGCTACACTCAGAGTCCACACGTTCGCTTGGGAAATAGCTACCGTTTGTTGGTACACCGAAGTCAAAACGCCTTCAATGACCTGCTCTCTTTTCTGTAGCTCTCCAGATGCGATATGGCTGACGTCACTCGTGAATAGCGGTGTACCCTGCAGAGGTAATGAACTGTAAAGTACGTCACCGTGATTATTCTGCAGATAGATATCTACATCATGTTGCTCCGCTTTTTCGATGAAAGAATGCAACTCATTGAGTGGCTTGGTTGCTTTCAATACACCGCGATGCTTGCCTTCATAGTAAAATGGCACCGATACTTGTAACGCGGTCTCGTTGCTGCTCAATGTCATTGCTTTCGCGCGAGATGCACCTTGGTACCACGCTTCTTTGCGTGGGTCGTAAAAATACACATTACCGATCCCAGTGTCAGAGTGATAAGAGCGCCCGTCTTGGAAAGCGACTAGCACTTCGCTGGCTCGGGTCGCTTTTTGTATTTGCTCCACCATCAATTGGATTTGAAGATCACGACGATCGATTGAAAAGTCCGGCGCCACTTGTTCGACAGCGTGTGTGAACGACGACGCCCATTGCGCAATACCATCAGTCAGGATCTGCAGTTTGAGGTCGGAAATTGTCTCTATGTGCTTTTTGGCGTCTGAAGAGACGAGAAAGTAGGAAAGCGATGTCAGGATAATAAGACAAGCAAGCAAGAGCGACGAGGTCGCGACCAAGATTTTCGATTTAAAAGATCGTATGACTACCACTCATCCTTCTTAGCAATAAGATATGCATAACGTTGAATTTATATACTCAGTAAATCAGACTTTCCCTCTTCGAACATGCCAAAGCTTACGCTTTTTGAGACAATCCTTGCCCTATCTTGAAATCAGAAGCGTGAAGGGAACAGTCGGAATCCTAATGTAATGCGGGTGTTACGAGTTCGATAATCCAGCAATGAGTCCCCATATCCTGAGCGATACTGGGCAAACACCGCCAGAGTATGGGTTAGCGGATGCGTCACTGAAGTCTCAATAAAACCACGCGATGCAGGTCCTCCATAGGCACCAGTAACAGCCCACTCCCCCCTCCCAATGGGGACGTTCACATCCAACTGCCACGGAGGAATATAGTGGGAAATATCAGGATTATCACTCAGTGCGAACGCATGCCACCCTTGAAGAGTCATCCCCCAAGAGCTCGTATCGAAGTGCATGGCCAAAAAGTAACGCTCCCAACTCCGAGAGAGTGCACCCCCTCGTCCATTCGACTGATGACGATAGCCTTGAGATACGCGTAGCCTGCGGCTCACATGATGCAGGCTAAAGAGTTCTGGACGGTAATTGCTGTCTCTAAAAGGAGAAGATCGGTCTGTGTTTCCTAATTGCCAAAAAGACGTCTGCGTGAATGCCCCCATAATGGCCCTATCTCGCTGTTCAAAGACAGGGAATTTCAGACTGAATTGAAATTTTGCTTCAGTGTTTTTCATTCCTTGATACTCCCCATGCTCCCCTTCAACTGCGACGCTAGTATTGACCTGGTCAACATAGCCAAAAGTGAAATAATTTGGTTCATGAAGCGTAATTTTACCTTGTGCCCCTGACGCTAACAGAGCACTTGCGACTGCCATTATCACCTTTGTAATCCAATTCATCTTTCCTGCTCTTCACATGGACGTAAGCGTCCTACTTTAGACGGAGCGTTTCGCTAAAATCAGACAAAACAGCGCAGTTTTCAGCCAAGCGTTCAGGACGACATAAATTGCCTCCTCTTTGTCCGTTATTGAAGAGAGGTTTTAGCGACACTGGAGGACAATGACACGCCGCTTGTGACCTTTATTTGGAGACCACATGATGAAAATTCTAGAAATGACCTTTGACGGCTACTGGTTGTATCGCCATCGCCACCGACTGCCAAAAATTGGCGGACTGTATCTTGTTTATACCTGTGAGTTTGCCGTTGACCGTAACACGCTCAGGCTGCTCAACTTAATTTATGTCGGCTGTAGCACTGACATTC

>NODE_53_length_3319_cov_2.279457

GGCCAGTGGCGACTCAAAGTCCCAATGCAAATAGACGCAGTATGGGCTGAGATGGAAGTGTGTATTGGCGCCACCGAAACACAAGCGATTGTTGCGGAGGAGTTTGTTCCTTTTCAACGTGAAGTGTCACTTGTCGGTGCACGCGGTAAAAATGGCGAAGTCGCGGTCTACCCACTTGCAGAAAACGTCCACACCAACGGGGTACTGAGCGTCTCAACAGCCATAGCCGATACTGAGTTGCAACAACAGGCACAAGAGATGTTCACTGCGGTAGCTAACGAGCTCAACTATGTAGGCGTTCTGGCCCTCGAGTTCTTTGATTTGAATGGCACCTTATTGGTCAACGAAATCGCACCTCGTGTCCATAATTCTGGCCACTGGACACAACAAGGCGCAGAAACCTGTCAGTTTGAGAATCACTTACGAGCAGTGTGTGGCATGCCACTGGGTAGCACCAAGCTGATCCGCGAAACGTCGATGATCAATATCCTCGGTGAAGATACCCTGCCAGAAAGCGTACTCGCTATGCCGGGATGTCACGTTCACTGGTATGGGAAAGACAAACGCGCAGGACGTAAGATGGGCCACATTAACGTGTGTGGTGATTACAGTGGTGAACTGCAACGTCAATTATCTGCTCTGGCAGAAGTCTTAGACGCGGATGCTTTCGCGGCGGTTCATGAGATCGCGCGTCACATTAAGCACTAGTCATCGCAAACTTAATAAAAACAAAAACGGCGCTGAAACAGCGCCGTTTTTATTGTTCATTCTGGGTGTGATGGCATTTTCGATCGGCACACTGATGTTTAATCCCACTAGCGAGCTTCTTCTCGATCAATAAAGGAAAACCACATTTTTCGCACTTACCCTTAACTGGCTGCTGATTGACCGCAAACTTGCATTTAGGGTAGTTATCGCAGGCAAAGAAGGCTTTGCCAAAACGGTTCTTCCTCTCGACCAAATGACCTTTGCCACACTCCGGGCACGCATGTTCTGCTTGAACCTCTTGTTCAGTCGGTTGCTCAAGCGACTCGATATGGTCACAGTGCGGATAATGACTGCAACCAATAAACATACCGTAACGCCCTTGGCGGAGCACCAATTCATTAGAGCACTCCGGACAAGGTACGCCCAGCTCTTTAATGATATGGCCGTCATTCTGATGTAAAGGTTGGATAAAGTCGCAGGTTGGATACTGATTACAGCCCAAAAACGGGCCATGCTTGCCGTGACGAAGTTGTAGCTCGCCCTCGCGCTGCTCAGACTGGCATTTAGGGCACGCTTTATGTTCTAAAGCGTGCTCATGGGCCGAAAACAACTGATGATCTATTTTACCGCTCATAGCCACTCATGGAGTTTAGTGAAGAATACCTTGCTCTTTGGTATACAACAGCTCTTCCATAAGCGTGTAGGCGTTTTCATTACCTGGAACATTGAACAGAACCATCAGCACAATCCATTTCAGATCATCCAGTTCAAACTCGTCTGTTTCAAGCCCCATGATACGGTCAATCACCATTTCACGCGTCTCAGTCGTCAGCACATTGACCTGCTCAAGGAAAGTCAGAAAACCTCGCGATTCAAGGTTTAAGCGCTCACACTCTTTCGCGGTATAGATACGCGTTGAGGTAGATGTGCTGCAAATGGAAATCGCGGAATGCGTTTCGCTTTGCTGCAATGCAGCCAACTCTTCCAGCCAGACAAGGGCTTTATAGATGTCTTTTTGATGAAAGCCAGCACGAAGTAATTCTTCTTCTAGCTCATCTTGATTGACCTGTAACTCCGCATCGCTATGGATGTAAGTTTCAAACAGATACATAAGGATATCCATCATCATAGCTAGCCCCTCCCCTTTCGAATATAGCCACCGGAAACCGCAACAACATGCCCTGAGAGCTCAAGCTCCAAGAGCTGCATCATGACTTCATGCACAGGTATATTGGTCCTGCTTGCAAGAATATCAACTGGTGTAGCCTTACTTCCTACGTTAGCTAACAGCTGAGGAAATGGCAATTCTTCTTTGCAATCAATTTGTTCAAATAGATCGCTTTGCACTGGCGGCTTATTAACATCAGACCAGCGAATCAGACTGTCAATTTCATCGGTTATGTCAGAAACCTGCTGAACCAGACACGCACCCTGTTTTATCAACAGGTTACAACCTCTCGCATGGGGATGATGAATCGAGCCGGGAATCGCAAACACTTCGCGGCCTTGCTCCATCGCATAGCGAGCGGTGATCAGAGAGCCACTTTTCTCTGCCGCTTCTATGACTAAAACGCCCAATGACAAACCACTAATGATTCGATTTCGGCGTGGAAAATTATCGGCTTTTGGCTTCACATTAGGATGGAACTCAGACACCAACGCCCCTTGTTGAACGATTCGTTGAGCTAACTTCTTATGCCGTGCCGGATACACAGAATTTAAGCCACAACCGAGCACGGCGATGGTTTGGCCATTACCATTGAGTGCCCCATCATGAGCATAACCATCAACGCCTAGCGCCAAGCCGATGGTCACGACCATGTCTTGATTCACCAACTGGGTTGCAAAGCTGCGTGCGGTGTTAAGCCCATCAATACTGGCATTGCGACTGCCCACAATCGCCATTTGTGGCCGAGATAGACTCTCTAGCTGACCTTCAACAAACAGTATTGCTGGCGCAGCGTCAGCCTGTTTCAGTAGTGCCGGATAACGCTGATCACTCAAAGGTAGAATGGTTCTGGCACTTGATTCTGCTTGCCACTCGAAACACGCCTCAATATCCTCGGCAGCTCGGTGTTGGAGATAATTCAGTTGCGAGTCGGAAAAGCCTAAACGGCGTAATGTCTCGGGTGAAGAGTCAATGATGTTAACTGGAGAGTCTACCGCTACCAGACGCTGAAACGTTTTGGGCCCAATCCGCGGCGTAAAGTACAAGCTCAGCCAAGCCGTCAGTTCTTCTTTCGTCATAGGAAAATTTAATCCGGCCTAACACCTTGTTACACGACACTGATGAGCTAGAGAGAGTCAGCCTAAACCTCTTATTCCAAACAAAGCTTGGCTACTCAGTGTTCGCCAAATTTGACATGATTCACAGTGCAATTTAACTCGGGGAAAATACGCAAAAGTGTAATGAGGCTGTCATTTGAGTGGCAAAATGTCTAGAATTGAGCCAACAAGGTTTATCCTGATTCGGCTCAGTTCAACATTTCGAGTGTATATGTCTGTATTACAAGTATTAACATTCCCAGATGAGCGCCTTCGCACTGTTGCAAAACCAGTAGAGAAGGTGACCCCTGAGATTCAAAAGTTCGTCGACGACATGATTGAAACC

>NODE_54_length_3304_cov_2.168578

GACCATCGCCTTTTGCTGCATTTGCTGATAAGCCTTCTCGGAGGATTCAAATAGAATGGTACCGTCCTCAGACAGAGTGGCTCTGAGCCCAAGAGGAATCAGACTCCGGTCTAGCATTTTAACGGTTTGCTTGCCCTCAGACTGACCATCAAATTGAACCATCACTGAGTTGCCTTGCGGGAAATCCAGGCGAATTTGCTCAGCTTTGTCACTCAAGCGATGGATGTTAAGTCCAGGGATTGAGAAACGACGCATATCCGCTTTGTCGATCTTGAGCTTCAACTCGTTGTCGATCACTTTGTTACCGTCAAAACGTGCGCTGTCTAGAATGCGCTCTATGTTAGCTTTGGATCCTGTTAAGCCTTCTTTTAAACCAGCAACATTACCCGCACCAAGGTTCATTGCCCGAGTGAGACCTTGCTTTATCTTGGCTAATTCACCGCCAATGGCTTGCAAGGAGCGTGTCGCAATCTGAACTGAAGTCGCATTTTGCTGCCCCTGAGTAAGCAAAACACCAGACACCGAATACGATGCTGGTGTTTGGTTTTGGGCAGCTAGTTTACGCTGCCCTATGCCACCATGAGACTCCGAAGAACGAGGAGGAGAAATAGAAGCTCTATCGTGCCCAGTCAATCTGACATTGTGTGGACGAACCTCAACTGTGCTCATTACCATATTTCAATTCCTGCTTATCAGATGCGGTCGAATAAGTTGAGATCGTTAATCTTAACGTAGCTGGCTTGAGTCGCTTGCAATGCGGCCATGTAGTTACTCAAACGAACTGAAGCTTCACCGTAATCTAGTGCGGAGAGATCGCTGCTGACCTTATCGACAAACAACTTATTCTCACCGTGGGCACTATCCATCAAATCCAGGTTGTTGTGACGACCACCCATTTCAGTCATCGCAGCTAACACGTTGCTGGATGTTGCGTCGATAGAGCTGAGCGTCGATTCAACCGTAGTTCGAAAAGCTGGCGTGGGTGATTGGAACTCTGCAATCAAAGCATCAAGATCGTTAAGAACGTTATTGCCACCTAGATCCAGAATTTCCTGTGCAGTCATATTGGATTCCATCGTCACACCTTTCGCTACTGTCACAACGCGTTTATCGCTGTTGCCTTGGATTACGTAGCTCCCACTCGCGTTAGACAAAGCTGCCGTGTCTGTTTTAGTTCCGGAGAACAAGTAACGGCCTTCTTCATCCTGAGCGTTAAACGATGAAACGACAGAAGAACGTAGACTTTCAAGTTCGCTGATAATGCCAGTACGATCGCTGTCGGTTAACGTACCATTAGCTCCCCATAGAACCAGTTCGTTCATCTTCTTCAGGCTTTCATTCACTGAGTCAAGTTGAACTTCCTGGTTGGATAATGCGGTCTTCACATTGGAAATGTTGGTTTTGTACTGGGTAATGGCTGAGCCTTCACGCTCTAAGTTAAGCAATTGAACTGATGCCATTGGATCATCAGACAATTTGGTCAGACGTTCACCGGTAGACATTTGCTGCATAACTTGCCCAAGTCCAGCGTTATTAATCTGCAAGCTTTGCAGCATCATCTGACTGAATTGAGTATCGCTAATTCTCATCTGATTTCCCTGTAATTAGAATAACTGAAGAACGGAATCAAACAGCTGATTTGCTGTGCTGATCACTTTCATGTTGGCGTTGTGAGCGTTGGCAAACGTCATTAAGTTTGCCGCTTCTTCATCACTATTTACTGCGCTGACGTTATCTCGGGCTGCCATTGCTTGTTCGCTCATCGACAGCTTTGCCTGATAATCCGCATCCGCCTGACGTGCTTTGATGGCCGTTTCTCCCACCATGGCAGCAAACGCATCGTTTAATGTGACTGAACCAAAACCACTGATTGACACGGCTTTATTGCTGACTGCAATTAAGCTGTCCAGCACATCGGCATTGCCAGGATTACCGTCACCAGACAAAGCAAGTTCCTCTGGTTTGAGGTCGGTAATCGTCAGGCTTGCCGCTGGGTTAGCAGGGTCGTAACTGAACAAAGGCTTACCAGCATTGCCATTGAGGTCTTGGCCGGTTACCAGTAATGCGTTGAATTCATCAGCAAGTGCCTTAGCCATATCGTCAATCGCAGAACGGTATGGTTTTAGCACCTCCGTCTGATAGTCGGTCAGTGCGCCAACTTGCCCACCCGGTGTATCAGTAATCGCAAAACTCTGAGTACCAAACTCAACATGCAGATCGGCAAGATATGGATCGCTTGGATCAGGCACGGCCTTAAGCACAGCGGCATCTCCACCCATGACCAGTGGCTGTCCAGAAGAAAGCGTAACCTGCAAGCTGCCATCGGCCTGTGACGTCGTTTTCACTTCCACAACTTTAGACAGCTCACCAATTAACGCATCACGAGTGTCCATTAACTGAGCAGGGTTACCCCCTGAGCCTTCCATCTCAACAATCTGCTTGTTCACATCAGCGATGTTACTCATCAAGCTGTTGGCATGAGAAATAGCCGCATTGCGCTGGTCATTGACGTCTTTATGCTGGTGAGAAAGCGATTCGGTGAGGGTATTAAAACGACGCGCGAGGGCTTCTGATTCATTAATGATTTGCTGGCGGTAAGGAACCGATTCTGGTTTTACCGTTGCATCATTCAGGGCTGAAAATAGCGTATCCAGACCAGCTGAGAGGCTGAAACCGTCAGCGCCGAGTGTATTTTCCAGTTGGCTCATCGTGCTGCTGTATCGAGTAGCGTAAGCAGAAAGACTGTTTGTCGCCCAAGTTTGCTTGACTAGAAACTCATCCGTTACTCGACGGATGCTGGTCACTTCAACACCCAGCCCTGGGCTTAGCTTGTCGTGTTTCGTTCCACCAACAGAAGCCATCAAGGCTTGTTGACGGCTGTATCCCGGTGTATTGATGTTGGAGACGTTCTGCGCTGTGACATCCAGTGCCACGCGATTCGCATTGAGGCCGGAAAGGGCGATATTGACGAGGTTCATTTACAGCTCCATTTTTCGAATCAATGGTTGAGCGAAGGCTGTGGTCACGCTGACTTCGCCCAACATTTGTCTAACTGAGGCGACCGTCTCCTGAGTGGAATGAAGCGCCTGGTCCGAATTAGATGTTACAGATGCTTCAGCGTGTGAGCTTGCTTCCACCTCATTGAGATTGTCATTCGCCGATAAGGTTGACGCACTGGTTTCAAATTCCACTTCCGAAATCGAAGGACTGAGCTGCTTAATCAACATCTCTGAAATGCCAGAATGCTGTTTTTTAGACATTTCAATCGCTAGCTGACCATCGTACATATCGCGAAAAACACCCT

>NODE_55_length_3281_cov_2.312422

GAGAAACACTATGACACGAAGATTGATGTACTTGCTTGTCCTCACATACAGCTCTTTTTCGTTTGCTCAGGATTGCATTGGCGTGGTCCCAGCAGGCTCAGTGCATGATTTCTGGAAGTCGGTCAAAGCAGGTAGTGAAAAAGCAGGCCAGGAACTAGGCATTAAAATTTACTTTCGTGGTCCCCACGATGAATCTGATGTCAGTGCTCAGCGCTTCATCATCGATCAAGTCATGGAGAATGCCTGTATTGGCTTAGTCTTAGCACCGAATACCAGAGATAGAGCCTATGATGTCGCCCGCTTGAAAGAAGCGGGTGTACCCACAGTCTACTTTGATCGAGATACTGGCTCTCCTGATGTGGTCAGCGTGATTGCCACTAACAATTATCAAGCAGGCGTCCTCGCGGGAAAAGAAATGGCTAAAAAGCTAGGTAACAAAGGAAGGGTCGCGTTACTCAGAATGAAGAAAGGCGTGATTTCTACCGATGCTCGTGAAAAGGGCTTTGTCGATGGCGCAACTTCATCTGGGCTCGTCGTGGCTGTCGATGGTTATCTTGATACTAAAATTGGAGAAGCTCGTAAAAATGCGGAGCGAATTCTTCAAAGTCAGATTGCTGAATTGGACGGCATATTTACACCTAATGAATCCACTACGTTAGGTGTACTTATGGCTCTGGACACCGTCTCTGCCCCAGAGGGACTGATCCACATCGGCTTTGACTCCAATGACTCTCTGGTGAATGCACTCGAAGACGGTTCTATTTATGGGCTTATCATCCAGCAACCTTTCGAAATGGGCTACAAAAGTGTCTATACCGTATACCAAGCCTTTCTGGGCCAAGAGTATTCCAAAAACATCGCTACCGAGACCATTTTCGTCACTAAAGAAAACATGCTTTTGCCTAGCGTCAATCAGGCACTGTTAATGAAATACGATTAACTCAGACTGTGGTTAATTACGCCGATAAAAACAAAAGGCCGCTGACAAGTCAGCGGCCTTTTTCATACAACACATTTTGCCTTATGGCATTTCGTCAAACTCTGCGCCTTCTTTCTCAACTTGAGGCGGCATCAGATGCTCTTTAGTAATACCCAGTTTCAATGCCAGTGCCGAAGCCACATAGATTGAGGAGTATGTACCTACAGTGATACCCAGTAGAAGTGCAGTGGCGAAGCCATGAATCATAGCGCCGCCTTGCGTGAACAGAGCAATGACTACGAATAAGGTGGTACCGGATGTAATCAAGGTACGGCTCAATGTTTGTGTGATTGAGCTGTTCATGATTTCAGCTGGTTCGCCTTTACGCATCTTGCGGAAGTTTTCTCGAATACGGTCAAATACAACAATGGTATCGTTGAGCGAGTACCCCACTACCGTCAGCAAGGCTGCAACGATTGTCAGGTCAACCTCGATTTGCATCAAGGAGAACACGCCAAGCGTAATGATAACGTCGTGTGCCAGAGCAAGTACCGCACCAGCCGCTAAACGCCATTCGAATCGCACCGAAACGTAAATCAGGATACAGATAAGAGAAACGATGATCGCCAGACCACCTGCTTCTGTTAGCTCATCACCTACGTTTGGCCCTACAAACTCGATACGGCGCATTTCAACGCTTTCACCAGTGCCTTCCTTGATGGCAGAAAGAATTTGGTTACCCAAAGTCTCACCTGCGACACCATCACGAGGGCGCAAACGAACCATCACATCACGAGCACTACCAAAGTTTTGAACCGTTGCGTCACCAAAACCTTTTGCTTCCAGCGCAGTACGAATCTCTTCCAAATCAGCAGGCTGCTCAAAGCCAACTTCAATCAGCGTACCGCCAGTGAAGTCTAGGCCCCAGTTCAACCACTTAGTAGAAAGAGTAAAAATCGACGCGCCAATCATCAGGATCGACAGCACGAAGGCGAATTTCGACCAACGCATAAAGTCGATCAATTTGTCTGCTTTTAGAATCTGAAACATAATAATTCCTAGCCTTAGATCGACAGTTTGTTGATGCGCTTACCACCGTACATCAGGTTCACGATACAACGTGTACCAATGATGGCAGTAAACATAGACGTTAAGATACCGATAGACAGTGTCACCGCGAAGCCTTTGATTGCGCCTGTACCTACAGCAAACAGGATTATTGCGGTAATCAAAGTGGTGATGTTGGCATCGGCAATGGTACTGAATGCGTTCGCATAGCCTTGATGGATCGCCTGCTGAGGGTTTCTTCCGTCACGTAACTCTTCACGTATCCGTTCGAAAATCAAGACGTTAGCATGCCATACCGACTGTAAGAACAATACCGGCAATACCAGGCAACGTCATCGTTGCTCCAGGAATCATCGACATCACACCAATAATCAGTACGAGGTTTACCATAAGAGCAGTATTGGCAATCAGACCAAACTTACGGTAGTAAAGCAGCGTGAATAGCATAACTGCCGCCATACCCCAGATACACGCCATGATACCCATGTCGATGTTCTGCTGACCCATCGATGGACCAATCGTACGTTCTTCAACAATAGAAATTGGTGCAATCAGAGCACCTGCACGAAGCAGTAGCGCTAGGTTGTGCGCTTCAGCCGCCGAGTCGATACCTGTGATGCGGAAGTTACGGCCAAGCGCTGATTGAATCGTTGCTTGGTTGATCACTTCTTCATGCTTAGTCAGGATAACGCGACCATCTGCCGTCTTACGTCCACTGTCTTTGTATTCTGCAAACACGGTTGCCATCAGCTTGCCAATGTTCTTCTTAGAGAACGCTGACATCTTGTTACCACCTTCGCTATCTAGCGAGATGTTAACTTGCGGGCGGCCATACTCATCCACGCTTGAGCTCGCGTCAGTGATGCTAGAACCACCTAGGATAACGCGTTTCTTAAGCACAACCGGACGACCATCACGGTCTTGTTTGATTTCACTGCCCGCTGGCGCTCGGCCACTGGCTGCAGCAGCGAGGTCTGCTTTGTCGTCGACTTCGCGGAATTCAAGCGTTGCCGTCGCACCCAGAATTTCCTTCGCACGAGCGGTATCCTGAACACCTGGTAGTTCAACAACAATGCGGCTTGCGCCCTGACGCTGTACAAGTGGTTCAGCAACACCCAGTTCGTTAACACGGTTACGCAGAATAGTAATGTTCTGCTCAACAGCGTAGTTACGAATTTCGGTCAGACGTTGCTCAGTAAAAGTGGCAGTCAGAGTGAAACGACCATCAGAGTCTGAGTCAACAAAGGTCATATCAGGATGATTTTGCTGCAGTAAGCGTTTGGCATCCGCAAGC

>NODE_56_length_3272_cov_2.603756

GGATAGCACCATAAACAATTACACCCTCCCAGTTATTATCAATCGCTAGGATCGCCAATTGATCACCAAGCAAGGCCTTTTGACAGGAACCGTGTCCATCGACGATCAGCACTTTTCCCTTGCCGTCCTGACTCAAGGTTTCTCTCACTTTGGAGTTATCGTGGTAGCAGCGAACCGTGACAATTTCTCCCCAGAACGCACTTTTGTTACCAAAAGTGTGTAAGGGCAAATCCAACGACGTTACTTTATCTTCATGTTTATCACATATATCCGGTGTTATATCTTTCATTTTTCCTCCATGAATATGGCTTTAATTTCTTATCTGCATCGATACTTCCACTCACCTAAACTGACTTTCGCTTTGTCAGCCAAACAGGAGAGTAAAAACAGCTCATTACCATCAACATAGTAAATCGCGTTCTGCTGATATTTACGCCCTAGCTCTAGTGCCTGTAGCTCATCAATATCGACGGCGAAACTCTCTTCAGCCCAACCGAAACTTTCATCTCCCACTAACACTTCAACAAAGTAAGTATGGCTAAATTCTTGAACTAAGTAGCGATTATTTCGTTCATTTTGTTGTTTGGACTGCCACTGACTAGCGGGGTTCCACGCCGTGATAATGGCAAACTGATGACTGCTCGGCGAGCGGATAAATTTGAAGTAAGGGTCTGAATATTCTTGCCAAAGTTGTGCATCAACCATCATATAAACCACAGTTTTACATTTGTTTTAGACTATTAATATTTGTTACATTCGAACTCTTGATATAAATCAACAAAGTGCATGGAATTAACATTCTGACGTTGTTAGCATGCTGTTAACATTATAGAATCCGCCTCAAAGACTAATAGAACGTGTGAGGAAAAGGGACCTCAAGAAGCGAATTGGGACCAAGGAAGGCGGGCAACCAAAGAAACAATGTATTTTTACAGTCTTTGGTTTATTATCAACATCACATTACCTGTATGTTAGGTTTTTGCCTAGAATTTATTCTATTAGCACAATTTTTTCACAAGTTTAGGTACCGCCAATGCAAACCCCGCACATTCTTATTGTTGAAGATGAGCAAGTAACTCGTAACACTCTAAAGAGTATTTTTGAAGCAGAGGGATACGCTGTTTTCGAGGCCAGTGACGGTGAAGAGATGCACCAGGTGCTGTCTGACAACCAGGTAAATCTGGTTATTATGGACATCAACCTTCCAGGTAAAAATGGCCTTCTGCTTGCACGTGAACTGCGTGAGCAAGCAAATGTGGCATTAATGTTCCTGACTGGTCGTGACAACGAAGTCGACAAGATCCTTGGTCTTGAGATCGGTGCAGACGACTACATTACTAAGCCGTTCAATCCACGCGAACTCACTATCCGCGCTCGTAACCTGCTAAACCGCTCTATGAGCACCAGTGTAGTAACGGAAGAGAAACGTAGCGTTGAGAAATACGAATTCAACGGCTGGGTATTGGATATCAATAGTCGCTCACTGGTTAGCCCAAGTGGCGATGGCTACAAGCTACCACGTTCTGAGTTCCGTGCCCTGCTTCACTTCTGTGAAAACCCAGGCAAGATCCAGACACGTGCTGACCTGCTTAAGAAGATGACTGGTCGTGAGCTTAAGCCACATGACCGTACTGTCGATGTGACTATCCGTCGTATCCGTAAGCATTTTGAGTCAGTCTCTGGTACGCCAGAAATCATCGCGACGATTCACGGTGAAGGCTACCGATTCTGCGGTGATTTAGAAGAGTAATCACCAGAATTAAGAAAGAAAAATGGAGCCAATTGGCTCCATTTTTTGTTTTTTCTTTGTCAGCTGTGACTAAAATGGTAACAACTTATGAGCAAACTTTGATTCAGGCAGAGCTTAATAACACTCTGCCGAATGTGCCTTTAGCCTTTGCCACCTTTGATGGCATCAATGATCTCAGTCGTTGAGCAACCATCTTCAAAGTTAAGTACTTTAACTTCACCTCCATTGGCAATCACTTCTTTACCACCCGCAATCTCTTCAGGCTTGTAATCACCGCCTTTCACAAGCATGTCTGGCAATACTTCTGAAATCAGGCGTTGAGGCGTTTCTTCGCCAAACGGGACAACCCAATCAACCGCACCAAGACCAGCCAGAACCGCCATACGGCGATCTGTTGGGTTAACCGGACGACCAGGGCCTTTCAAACGCTTAACAGATTCATCCGTGTTCACAGCGACAATCAGGCGGTCACCCAATTCAGCAGCATGGTTCAGGTAAGAAACGTGTCCAGCGTGCAGAATGTCGAAGCAACCATTGGTCATCACTACCTTCTCGCCCTTCGATTGTGCGTTCTTGACCGCTTCAATCAGAGCCTGTTCAGTGATGACACCAAAATCAGTGTCTTGACTACCATGAACCGCTTCCGCCAATTCAATGGTTGATACCGTTGATGTACCCAGCTTACCAACCACGACACCTGCGGCTGCATTTGCCAATGCACAAGCTTCATCAAGCGGTTTACCCGCAGCAACTGACGCAGCAAGGACAGAAATCACAGTATCACCGGCACCTGTAACGTCATAGACCTCTTTGGCAAGAGTTGGCAGGTGGAACGGCTCCTGACCGCGGCGTAGTAATGTCATACCGTGTTCACTGCGCGTCACCAACAGTGCCTCAAAGTCGAACTCTTCAATCAGCGCTAAGCCTTTCTCGACCAGTTCTTCTTCAGATTTTACCTTACCCACAACCAGCTCAAATTCTGCCATGTTTGGAGTCAATAATGTCGCACCACGGTAGCGTTCAAAGTCTGCGCCTTTAGGGTCAATAAACACAGGAACCTTGGCAGCACGTGCTTTCTGAATAAACTGCTGAACGTGCTCCAATGCACCTTTCGCATAATCAGACAGAACAACCGACTTCACTTTTGGCAATGCCTGTTCCATACGGTCTAAAATCAGCTCGGCATCGGTATTTTCAAATTTGTCTTCGAAATCTAGGCGAATCAGCTGCTGACCACGGCTCAAAACACGAAGTTTAGTAATAGTTGGATAGTTTGGCAGAGCCACAAAGTCACACTTTACTTTCAGTGCAGACAGCGTCTCATTGAGCACTTTGGCAGGCTCATCAATCCCAGTTAAACCTACGATATGCGCATGGCCGCCCAAAGAAGCAATGTTCATTGCTACGTTAGCTGCACCGCCTGGACGCTCCTCGTTATTTTCTACTTTTACTACAGGAACTGGGGCTTCTGGGGAAATGCGTCCAGTAGGGCCATACCAGTAACGATCAAGCATGACGTCAC

>NODE_57_length_3257_cov_2.702201

GGCCAAGTGAAATCGGTCACGGTGACATGCTGACTGCTTTTGTCGTATTCACTCCAAAGTTGCTCGTATGTCTTTGTTGTTAGTGGATGAGTTTCGTGTGGAATAATGTCTGCTAGCGGGCGAAGCACAAACGCGTTTTCGGTGAGCTACCTAACTGGGTTATTGCACTGGTTGCCGCGGGTGGTTTAGCCGCTGCTCTGTCTACCGCTGCCGGCCTGCTACTCGCGATATCTTCGGCAATATCTCATGATTTAATCAAAGGCGTGATAAATCCGAATATCTCTGAGAAGAAAGAGCTACTGGCGAGCCGGATATCCATGGCGGTCGCGATTGCTGTAGCCGGCTATCTGGGGCTAAATCCGCCTGGCTTTGCCGCTGGTACGGTGGCACTGGCCTTCGGTCTGGCAGCCTCATCCATCTTCCCAGCCTTGATGATGGGTATCTTCAGCAAGAGCATCAACAAGGAAGGCGCGATTGCCGGTATGATCGCAGGTATCACCATCACTCTGTTCTACGTATTCCAGCACAAAGGCATCTTGTTCATCGCTGACTGGAAATACCTAGAAAGCTGGGGCAGCAACTGGTTCCTAGGTATCGAACCTAATGCCTTCGGTGCTATCGGTGCACTGTTCAACTTCCTAGTGGCATTCGCGGTATCGAGAGTCACGGCAGAAACACCACAAGAAGTGAAAGATTTGGTCGAGCACGTTCGTGTTCCAGCAGGCGCTGGTGATGCGGTAGATCACTAATCTACACTTAACTATCAAAGCCCCTCACGGGACGACCCGTCCGGGGCTTTTTTATTGCTCTTAATTCAGTTACCGTAGTTTCGAAATGTTGTCGCAAGGAAGCCAAGATGTTCAAAAATAAGCACTTTATTGTCGCTCTTTTGATTGCACCGATCCTGTCCATTATTGCCTATTTCGGCACTGATATGGCAGTCAGCGAGAAGCCCCATGCCGCTAAAGAAGGCCAAAGCTACAAGCTGGTTAGCAAATCAAACTGTCGCTATACCAGTGGACTGTGCGATATGGAAAATGGCGACTTTAAGGTTAAGTTTCGTTCTGAAAAGCTAACAGAAAACAGTCTGGAGCTATCGCTACACGCCGCCTACCCGTTAGAAGGAATTAAGCTTTCTTTGGTCGACAGCCAAGATCAGAACACGCAGCCAATCAATATGCAGCCAGCCGATCAAGCCGGGCAAAACTGGTATATCACGCTGCCTAAACCCACATCAGCAGACAGCTGGTTGAGAGTGGCGATTCAATCTGACGGCACGCTGTATTACGGGGAAACACAAACCGCATTTGTGAAATACGAAACACTGTTTACTGAGTAAACCGAAATTAGAATAGCTAAAAGGGTTGACGTGAAGCGTCAACCCTTTTTGTTTAAACGCACTGAGTCATATTCAAACGCACGACAGGATGCTTGTAAACCTAGACCCGATTCAGAATCGCTCTAAGTTTGAGCGGTTTAACTGGCTTAGGAATAAAGCTAAATCCGTTAGATTTAATGCCATCCATCATGTCGTTGGTTCGATCAGCACTGATGATCACCCCTTCAAAGCTATCTCCCAAGCGCAGGCGACACTGTTGGAGAACTTCAAGCCCTGTACGGCCGTTATCGAGACGATAGTCGGAGAGAACCACGTCCGGCAGCCACTCTTCGTCTAGACACTTCAGACTGGTGACAATATCGGTTGCCGTGCGCACGTTACATCCCCAACGCGCAAGCAAGTTTTCCATCCCGACTAAGATTTCTGGCTCATTGTCGACACAGAGTACATTAAGGTGGCTGAGATCAGAGACGGCAGCAGAAGGCGCAACTTGCGGCTGGACCAAAGCTTGTGCACTGCGATTGAGCGTGATTGAGAACACGCTGCCTTTACCCGGCCATGAACGCATGGATATTTGATGACCGAGAACGTGGGCGATGCCTTTCGAAATCGCTAAACCAAGCCCAAGCCCTTGATCTGAACGAACCTGACTACCGCGAGTAAACTCTTCAAAAATTTCCTGCTGTTTGTCTTCATCGATACCGGTGCCGTTATCCCATACTTCAATCCTCGCCTGTTGACCTACCCGTCTTACCCCAAGCACCACCTTGCCTTTGGGATTGTAACGGAAAGCATTGGTCAGGAAGTTCTGCACCACCCTGCGCAGTAGTTTCGGGTCAGATTGAACAATCAGCTGACTTGGCACCATCTCAAACTCAATCCCCTGCTGTTTTGCCAATGCACTGAATTCAGCGTTTAGATTGGAGAGAACATCATTAATTGCAAAGCTATGCACATTAATATCAAGCTTGCCAGACTCCAACCGAGAAATATCAAGCAAGTCACCGATCAGATCTTCAGCAGCCTCCAAAGCACTTTCAATATGCTTGGAGAGACGCTTGGTTTCCTCTTCTTGCGCCACCTCAGACAGTGACGAAGCAAACAAGCGGGCGGCATTGAGTGGCTGCATCAAATCATGACTGACCGCCGCAAGAAAGCGCGATTTCGACTGAGATTCATGCTCGGAGCGTTGAGTGGCAGAGACCAGCTGTTTGTTGAGTTGCTCCAGCTCTTGAGTCCGTTCATGGACCCGCTCTTCCAAACTTTCGTTAGCGTCTTTCAGTGCCTGTTCCGCATCACGGAACACCGTGATGTCAGTAAAACTCATCACAAAGCCACCGCCGGGCATCGGGTTGCCCTGCACTTCAATAACCCGACCATCAGGTCGAACTCGTGAAGAAGTGTGGCGGGTACCTTGTTCAAGGTGATAGATACGGCGACGGACATGATCTTCTGGATCACCAGGGCCACATAGCCCCTGCTCAGCATTATGACGAATCACATCCGCGATCGGCCTGCCCACCTGAATCAACCCTTGAGGAAATACAAACAGTTCCAGATATCGTTGGTTCCATGCCACCAGACGAAGCTGCTTATCCACCACCGCAATGCCTTGTCCAATATGCTCAATCGCCCCTTGGAGAAGCCCGCGGCTAAAGTCATACAACTCAGAGGCTTCGTCAACAATGGTCGCGACTTCTTCCAGTTGCATGTTGCGCCCTTGTAGGGCAGAGGTCAGGACCAACTTTGCTGAAGAGGCGCCAAAGACGCCAGCCAACACCCGTTCCGTGTGGCGGATTAAACTGGCGGGGGCTTGCTGATTTGGCCGCATAGTTTCGTGTTGCTGGGCCCAGTATTGCTCAAACGCAATGCGCACCCTTTGTCGACCAACAAATCGCGCAGCTAGCATTTCCAGCTC

>NODE_58_length_3249_cov_2.665511

AGTTGAATTAGGGCATCAGGATCTTTGGCTGGTGATACCTCAGGTATCACAAGGCAAGCCCCGCAGTGAAGGGTTGAGAACAGATCAAACACCGACAGATCAAAATTGAGCGCGGAGATTGCCAGTACTTTGTCGTCACTATTGAGAGTGAGACGATTTTCTACGTCCACAAGCGTATTGATCACCGCTTGATGATCCATCATCACGCCTTTGGGCTGCCCGGTAGAGCCGGACGTAAAGATTACATAGGCTAGATCAGATGGTTTGAGCGCCTTAGGTTTGAATGTGTTGTTTTCCGTGTGGCTGATTGATGGGGTGAGGACACGATAAGGTTCCAGAGCGTCTGTTAACGCCTGTTGAGCAACAACGGTTGTGACTTGTCCCTGATTAAGCAATGCATGGATTCTTGCTTGAGGATAGTCGGCGTCTATAGGCAGGTAAGCTTTACCAGCCATCAAGATTCCTATCACTGCAACTACTTGTTCCCAACTTTTGTTCATCACAATGGCGACCAAAGGTGCATCATCGTCTTTGGCCGCAATGTCTGCCGCGAGTACACTCGCTGCATTCCAAACGTCTTGATACGTTAGGAAGAGTTCACCTTGTTGAATTGCGATTCTTTCCGGATATGCCTCGGTGCCCTGCTTGATCATTTCCAGTAAGGTCTGAGGTCTTGAACCGCTTGGCTCTTCGATGAGGTTAGGTTGAGTGTTTGGCTGATGCCCCCATTGATTGTCGGATAAGCTCAGAGTCAGTACATCATGCCAGTTGCTGCTCGTCAGCGATTCTACAGCCTTGATATAGGCATGGAACATAGCCTCGAAGACGCCTGACTTGAGGTGATCGTGTTGTACGGTCCATTTGATGTCTATGCCGCCGCAAGGTGAGGAAATTAACATCGCATCTAAGTAAACATGCGGCGTTTGCGCTCCGAAATCGCGAAGCGTTCCCATAGTCATAGAGCTCTGCTGGCCTACCGATACGGTATCGTTAAAGACCACTGGCATACCAGCGCTGAAGTTACGATTGAGCCTGTTCTTTTCGGTTAAGACGTCCTGACCGTCAAACTCGGCATGGGCAAGATCGTCTGACATTTGCTGTTGAACTTGCTGGATAAGAGCAAGTAAGGCTGGTTCCTCACTGATGTCTACTTCCAGCATGGATGTCGTCGATAGATTCCCCACCAAATTGGCTAAATCAGGTTCGTTAGCGGGTCTATTGCTGTGCAAGACATTTAACGAAAAGTGGTTTGTATCACTCCAACGACGCAACACGAGGCAGAACGTTGTTAGCATTGCCATAGAAGGTAACACATTGTGACCAAACGCCTGACGCTGAAGCAGGTTCCAGTTTTGTGCAGATACACGGTGCGTCAGCACGCGTTGATCCAAACTGTCACCTCTTGAATCCGCTAAGGGAAGTGCAGGAGCATCCGGCATAGTCGGCAAGCGATCGTTCCAGTATTGGCGAGCGGCTTGTTTCGAACCATTGGTACTTTGTTCTGCTTTGTTTTCCAGATACTGAGCGATGGTCATTAAGGGTTTGTCGAGAGCCACTGATGGGTTGTCGTATAAGTGTTGCCACTGCTGGAATAGCAGGTTGAGGCTTCTCCCGTCTGCCACGACCAAATCAACCACAAGGTGAAGCAGGCGAGTGTCTTGATCTGGTTTCACAATGCTGAGGTCGAACAAAGGCCAGCAATCCGTCGCTACCCCTTGCGCGGCAATGCGAGATCGTGCTTCGGTTAAATATTGTGCTTTGTCCGACTCAGCGCTCTGTGTCAGATCAACATAGTGAGGCTCATAATGGGGCACTTCTTGAAGAATGCAGTAATGCCCTTCATGGATTTCTCCACGCAACTGGTCATGGTGATTTATCAGTGTATTCCATGCGTCGACAAGTTTTGAGCGGTTCAGATCTTTAATTTCTAACTCAGCGTAAAAGTGCGCAATTCCCTGACCTAAAGAGAATAGGCTGCTCTCCCCCAGCCAATACGCGTTCTGCAACGGAGTCAATGGGTAGTCCCGATTAGCTTTTGGTGCATTGACCGTTGCTTCAGCATCCGGCTTGAATAAATGCTCACAGAATTGAGCGAAGACTGGCTGCTCAAAGATATGCTGCAGCGTCACCGCTGCAAATCCCGCCTGTTGCAGTTGAACGACCAGTTTGGTCGCCATCAGACTGTCGCCACCACACTGGAAAAAGTCTGTCTCCGAAGTGATGTCCTGGCTAAGGAGTGCCTGCCAGATTTGCTGAACCTTCTGTTCTTCAGGGTTAGGCTCGGGTGGCTCATTGCTCGCTTGCGGAGCGAACGTGCTGTCCAGCGCCTGAACGAATTGTTCGAGCGTTGGTTGTTCAAATATCTGCTGCAAGCTAGCGTGAGCGATCTCCTGTTTGTTGATTTCCACAATCATCTTAGTGGCCATTAAACTGTCGCCACCGCACTGGAAGAAGTCTGTCTGCGGAGTCACTTCCTGCCCGAGTAACAATGTCCATATTTGAGCGATGCGTGACATGAGCACATCTTGTATTGGCGTAGCCTGAGGTTCTACGACCACCTTTTGTGTTTCGTTTTCATCGTAGGACGGGTAAGCAGAAGGTTTTTCCACTGGTGATTTACTCTGTGTCGTTCGTGCTAAGAGCAAATGTTGGCGCAACACTGATTGCTCAGAGTCCGGGAAACGACATTCGGTGATGAAACCTTGCTCGCCAAGTACTCGCTGCCAGTCACTGGCGTTGAGCATAGCGCTGCCTGTATGTTGACGGAAATCGGTAAAGGCGTTGATCCCTTCGATAAACCCAACAGTGGCGAGCTGCATCGGGCTGAATTGATCGGTGGCTTCGATCAGCAGTATATGTCCATTGGTCGCAAGTATGCCTTTAAGTCGTTCAAGCGTATCAGGTAGGTCCGTTGCATCATGAAGAACGTTGACTGCAATGATGACGTCGTAACCGCCTGTTACCTGCATTTCTGGTTCGATATCGAGGTTGATGTCTAACCATCCAAAATCAACTTGGTTGTATTGTGCTAAAGACTGCCTCGCCTCGTTCAAAAAAGCATGCGATACGTCAGTGAAGCGATAGTGCTCAATGTATCGGGCTGCATTGCTAAGAACAGCAGTAGACGTTGCTGCTGTACCTGCCCCCACTTCTAGGATTTTTAGTGGCGCTCCTTTTTCCTCTCCCAGAGCCGAAATGAGATTGGCAGCGCTTTGGTTGAGTATCCTTAGAGAAGGATTAGTTCTGTAT

>NODE_59_length_3228_cov_2.618534

GCTGTACCGCGATTGTTGTGATCGTCATATAAGAAGTCATTGGCGAATGCGATTGGTAAATCCGCAACACCGGCGATATCGTAGTATGGAGACCACTGAGTACCGACTACAAATCGACCGAACTGCTCATGAGTCGCACCAATGTAACCCAAACGAGTAGTGAAAGAAGTGTCACCACCATCAAGGTAGTTTAGAGCCCATTCCCCTTTCGCATCGACAGTAAAGCCGTTACCGATATCTTGTGTTCCAGCGATGTTGATTCGAGGAGACACATCATGCACCTCCACTTCATCTGCCGAGTCATACTCACCGATACCCACATCTACGTAGCCACCGATAGAAATTGTTGAACCTTCGCTATTGTAGATTTCTGCAGCGAAAGCGTTAGTACCACATGCTGTTGCCGCAATCGCAGCGGCTAATAATTTAATTTTCATAACTTAACGTCCGTTTAGGTTTGTAACTTCTGTCCGTGTCTGGCCTGTAGGGCCAAAGCATCACGAACAATTAAAACGTAGTACGAAATATTTAAAAATGAAGTGCAACCAAGTGCTTAGCTTGAAGATTTATTAGAAATTGCTCAAATAACAAAAATGTGAACAATTTTTAACCACATGAAAGTATTAAAGTTTTTAGTGAGAGAAGAGGCCTTTTCTTACCTCTCGATCAAGTTCACACTTTTTGAAATTTAAACGGAACTTTTTTGAAAAAAGCAAAAAGCCCTCTATCAATAGAGGGCTTTTAAAGCTAAATCCAGTCAAATTAACAGGATAGTAATTAGGCAAAGTGCAATGGTGGAACAACCTAGTTGGTTACTCTACATCCGCTATCTGAAGATCGTATTTGCCCAGCGAGCAAGCCCTGCCGTTACCGAACCAAAATAGTTCCCGCTTATCACAGGTGTGTCAGGAAGTACGGATTTCACTGCCCCAGTGAGGATAGGTGAACGGGCTGAGCCCCCTGTCATGTATATCACATCAGGCTTGATACTACTTTGCTTAATAGCTTCGATAACCAGCGCTTTGATTTTCTCAGTTGGATTGACAATGGCTTCTTCCATTTGTTGGCGTGTTACAGATAATGAAACCACTTCAGAAAGTAGATCCAGATTTGCGTTGTAAGCCGCCGCGTCAGCCAACGCTATCTTAGTTTGCTCTGCTTCTCGAATCACCGAGTAACCAAGGGTTTCTTGATGCAATTGGACTAGTCTAGCCAGTTTTTCAGGTTCACGAGCTTCCTTTAGCAATAGTCGTAGCTGCTTGAGGTTTTCATGTGCGTAGAACTTGCGCTGGGCCTGAACATCGTTGATTGCAATGGGGTTCCAAAATTGAGTGGTTGGGATGTCTAGGCCAGACTGGCTAGTTGACCCCATACCAAACTCCTGCATAAAGCATTTAAAGGCAGTGAAAATATCTAGGTCATTACCGCCAATTCGTTGCCCAGAGTGAGCAATTAAAGACTCACTTCTGTCCTGTTTTCCACACCAACTTGGACCCATCTGAACAAATGAGCAATCTGTTGTACCACCACCAATATCAACAACCAGCACATTTTTATTCTGTTCAAGGCTCGCTTCATAATCTAAACCTGCCGCCAGAGGCTCAAACTGAAACTCGATATCTTTAAAGCCTGCTCGGCGGGCAGCACGGCGCAAAATACCTTCTGCCTGTAGGTTAGAATCATTCCCACCCCGACCAAGGAAGTTCACTGGACGACCAATAACCGTCTGTGAAATCTCTTTTTGAAGTGAGCATTCTGCCTGAGTTTTGATATTAGCCATCATGGCGCAGACCAAGTCCTCAAAGAAGGCCAACTGCATATCACGTAAGCCTGTTACCCCAAGAAACGATTTGGGGGATTTTACGTAATAAACATCCTTGGGATCTTCCAGATAGAGCTCTAAGGCTTGCTGACCAAATCGAATATCGTCAGAGCGTACTTCCAGCCCTTCCTCGCGATTAAGCTTAATCGCTCGACGCAATACGCTTTCACTGATTTCGCTACCAGGCTCAATGTCTAAACAGCGAAACAAATATTCAGAGACAACTTCCGCTGTTGGCGCACATAAAGTAGATGGAATATAGTAGCTATCTCCAACCAGAGGGATCTGCCTGATCTCATCATTGACTAATTCCGCTACCGAACAGTTAGCCGTTCCATAATCGAATCCAATCGCCATCAATCACCTCTTTATCATCTACCAAAAATCGGGCCGCACAGTAAAGCCTACACTTATAGACAGGTCAACCCAGTTAGGTTAATTCTTTTTAACTGCATCCATTTGTTTTAAAGTGACCGCTATCCGTTCATGTACTTAGGGAAGCGAGGAAGTGAATCTACGCCAGATAGAAGTGTTTTATGCCGTCATGCACGCAGGCACAGTATCAGGGGCAGCCAAGCAGCTTCATGTTTCACAGCCCAATGTCACCCGAGTACTAGCTCATACTGAGCAGCAATTAGGGTTCACGCTGTTTGATCGAATAAAAGGTCGGCTGGTTCCAACTCAGGAAGCCCAATCTCTGATTACAGAGGCGGAAAAGATCTACCAACAGCTTGGTCAATTTCGAAGTTTAACCAGTAAGCTCAAGCATGGTCAGCATCACCTCCGCATTGGTTCACCGCCAATACTTGCCTCTGGTTTCCTCGCACCGGTCATAGCAGAGGTTTGCAATAACACGGACTATACCTTAGAAGTGTGCACTGCAAATCGAGATGAACTGATTGATGGTCTAGTAAAAAACGAATTAGACCTCGCTGTATGCTTTGGGGAAGAGAGCGCTTCTTCCATTGTTCAAGACACGTTACTCAACACAGAAATGAAGGTGCTGACCCCCACAGAGACGATCGCTCAGCACTCAGTCACACTTCAGGAACTACTAGAGAGTTCGTTGCCACTGGTTGGACTCGACACGCGAGATCCACTTGGGTTTCAGCTGCACCAATCCATTCATGCCGTCGCCCCAAACTTTCACCCAAAGGTCAGTGTTAGAAGTTATAATGTTGCCGCGGAAATGGTCGTGCATGGTGCGGCAAACGCCATTATCGATCCTTGGACCGCCAAGCATTATCAATCGTATCCGCAGATCCAATGTGCTTCATTAATGCCAGCGATACCAGTAAACGTCTCCCTGATTTATTGCCAGCATTACCCGCTTTCTGTTTCAGCCAAGTGGTTCGCTGAGCGCCTTCAAGAAGCCGCGCGACAAGGTGTATAGCTTAACGTTATAC

>NODE_60_length_3196_cov_2.335043

GTCACCTAAAGTTCGTCTGTTGATTGATTTTCTGGCCGAAAAGCTAGGTGGGAGTACTGGAATCTAAGTCTGCATCTTGAATGGATTGCTCGACCATTTCGGCTATTTCCTCGGCCTGTTCAGGAGCTGTTTCAGATACTGCATTGACCCATTCTTCAACCGATTCCGTCAGGTTAGCGTCGTCACGGTCAGCGGGTCTCAAATTGCTGCTGTTTTCTACCATGTTGGCCACCAGCTCCTGACTCACTTCGACTAGTTTGTCAGGGTCATGCTCTGCGAGCGTTTCAATTACCTTGGTTGGGTCTGATTCCATTGATTCGGTAATGGCGGCGATGATCGACATGGCATTTTCCGGTTCTTTCTCAACCAGCCATTCTACGAGCTCTTCTATCTGGTTGTCGTCAGACTCAGCCAGGCGCTTCGCTATATCCACTGAAAGCTCTGGGTTGGTCAGAGTCATGGCTCTGAAAAGCAGGACGGTGTCTATACTTTCGATCTGTGCTGCACGCTCTGCAATTGTCACAGCCCATTCGGGTTGCTTGTTAGCAAGGCTGCGAATAAAGGTAATTGCATTGGAAGGGTGTGATGCTGCTAGTTCTTCTACCTGTTCTAAAGCCTCTTCTACATACAACGAGTGTTCCTGATACTCAGTTCGAGGGTCTAGTTCACCATGAATAGAGGCGGGTGTATGCATGACAAAGCTTTCTTGTTCTTCCACTGGCAGTGCCTCGCGAACTGACATCCGATAAATAGTGAATACGATAAGGCCTAAGTCCACCATGATTAGGAACGTAAATAGTGCACTGTTATCGAGTCGCTCCATTAATATGGAGGCAGTGTAGGGGCCAGCAATGGAGCCGATCGCATAGATACAAAGTAAACCTGACAATACTGGCACAAGCTGCGCTTTCGTAGCTCGGTCAAAGGTTTCGGAAATACTCAAAGGATAGAGACAGGCAATGAGGCCCATGGTCAGCGCGACCAAAATCAGTGGAGTGATCGACCATTGCCATTCAATCGAAACAGGCAGCGCAAAACTGACAATCGCGAGTACAAAGCAGCTGCCAAGAATAACTTTGCGACGCTCAAATCGATCAGATAGATATCCCATTGGCAGTTGTAATAAGATCCCGCCGGCTGTCGCTGCGCCCATAAAGATGGAAAGCTGGAAGCCAGTGATACCAATGCCGTCTGCGTAAATCGGCAGCATGTTGGCAATGGTGGAGTATAAGATGCCACACACGAAACAGGTGACAAAGCCCAGCGGAGAAAGAATGTAAATGGACTTGAGGGGGATGGAGTCCGATTGCTCGATCTGTGGCTCGAACTGGGAAATGAAGACCACTGGGGTGACTGAAATACTGAAAAGCACACCGCATACAATAAACAGGGTGGTTTCATTTGGCGGTGCAATGGCGAGGCCAAACTGGCCGAGTGTAATTGCGCTGAGAATAACCACCTGATTGACCGCAAGAATTTTCCCGCGGTTGGATTCGGTCGACACACTATTGAACCAAGTGTCTAAAGTGGTCGTTGCACAAGCGATACAAAAGCCCATCACAGCCCGCATTATTGCCCACATCCAGACATCGGAATTTAGCCCCATGATAAGAATCGCACTGGCTCCTAGGCTGCCGCACATAGCAAAAGTCCGCACTAAGCCTATGTTACGTAGAATACGTTTTCCTAGGATTGCACCGAGCAGAAATCCGACGGAATACATTGAGAGGACGAAACCTATCGACTGGATGCTGATATTCGCTTCCGCAAGTGAAACCGGGAGTAGGATACCGCTCAAGCCATGACTACTCATCAGCAAGAATGAGCTGATGAAAAGTAGTGCTAGTGGCCGGAGTGTCGCTGCGATAGTCATAACAATTGATTTAAAAGCAAAAAAGAGCCATTTACAAGAGGGTTGTAAATGGATTGTTATGGTTTGGTTTCTTTATTGGTTCGATGGTTGTATGACTAATATCGCATATCAAGATTGCATTACTTCGCTTTTTTACATCATCTTGTTTAATTATCATGCCAATTAACATTGACAATCGCCTGTCTCGCTTCTGACATTGAGTGTTTATTGCCTCATTATCTTATGCGCAATGCGATTGGTTCCTACTAGACAGAAAAGGTACAGATGAAACTTAAAGTAACGGCTTTTTGTATTGCCACTGTTGTTGCCCAGCCAAATGCCTTTGCCGCCGAATGACAGAAGAGCTTAATAGCCAGACTGTTTACATCGATGAAAGTGACGATTGGGTGAAATTGAAATCCGGTGAAGTGCTAAAAGGTGAATTGACTGGAACTGTTAAAAAGGAATCTAACTCTTACGATCAGGAAATCGAATTCGACAGCGATGACCTTGGCGACCAGGAAATTGAACTTGAAGATGTCTCTGTCCTTGAGACAGCAAGTTATTTCACTATCCGCACGGCTAGCGGAGAGATCCATGACGGCTATTTATCAATCCGAGATAACAAGCTCTATCTGAAAAAAGGCGATCAGGAACAATCATTCCCGGTTACTCAGGTGGTTTCTATTTACCGAGGCTCTGAGAAAGATTCGGATTATTGGACTGCGGATTTATTCTTCGGTTTAGATATCAGCAAAGGGAACACTGATGAATTTTCGATGCTGGGTGAAGTCGAAGCTGAGCGAAATACGGTGGAATCTAGAACCAAGCTTAATGCACGCCATGAAGTGTCTGAATCCAACAAAGAGAAAACGGCTCAGAAGAGTCAGTTTGACGGCTCTTACGATATTTATATCAACAATCGACTGTTTTTCAGGCCGATCAAGTTCTCAGCGTTGAGTGACGAATTTCAGAACTTGGATTATCAGGTAAACGCTTCGATGCAGGTGGGTTACTTCTTTATCGCCAACTCTGATACAGAGTGGGATGTATCGATTGGTCCGGGTATGCAGTACAGTGAGTTTTCAACAGTAGAAGAGGGCGAAGACTCAAGTGCCAGCAGTACGATTTTGACCTTAGAATCAAACTTCGAATACGAGTTGACCAAGGACATCGACTTTAGCTATACCTATAACCTAGATTGGGCGAGTGATGACGCGGGTGGTATGCGCCATAAAAATGATCTTGGATTTGATATCGACTTAGTCGGCGATCTGGAATTCAGTATCAAAGCCACTTGGGATCACGTCTCGCAAACCAAGGCCGATTCGGATGGTGTCGTAC

>NODE_61_length_3174_cov_2.002583

ACATTTAAGGGCGAGGAAATCACTTTCACTCGTCGTGGCGGTGGCTCGCGTTATGTTACTTACATGCCGGTTTATGACCAGAAATTACTGGATGACATGATCAACCAGAATGTGAAAGTGCAAGGTACTCCGCCAGAAGAGCAGAGCTTACTAGGTACTATCTTCATTTCTTGGTTCCCAATGATCTTACTGATTGGTGTATGGATTTTCTTCATGCGTCAAATGCAAGGCGGCGGCGGTAAAGGCGCAATGTCTTTTGGTAAGAGCAAAGCTCGTATGATGAGCGAAGAACAAATCAAAACCACATTCGGTGATGTTGCTGGGTGTGATGAAGCAAAAGAAGACGTAAAAGAACTGGTTGATTACCTGCGTGATCCAAGCCGCTTCCAGAAACTGGGCGGTAAAATCCCGACTGGTGTTCTGATGGTTGGTCCTCCTGGTACTGGTAAAACACTGCTTGCTAAAGCGATAGCTGGTGAAGCGAAGGTACCCTTCTTTACTATTTCAGGTTCTGACTTCGTTGAAATGTTCGTTGGTGTGGGTGCATCTCGTGTCCGTGACATGTTCGAGCAAGCAAAGAAAGCCGCACCTTGTATTATCTTTATCGATGAGATTGATGCAGTAGGTCGTCAACGTGGTGCAGGTGTTGGTGGTGGTCACGATGAACGTGAACAAACACTGAACCAAATGCTCGTTGAGATGGATGGTTTCGAAGGTAACGAAGGCATCATCGTGATTGCGGCAACTAACCGCCCAGACGTTCTTGACCCAGCGCTACTTCGTCCTGGTCGTTTTGACCGTCAGGTTGTGGTTGGTCTACCAGACGTTCGTGGTCGTGAGCAGATTCTTAAAGTTCATATGCGTAAAGTGCCACTAGCCGGTGATGTAGAACCATCTCTAATTGCTCGTGGTACACCAGGCTTCTCTGGTGCTGATTTAGCTAACCTAGTAAACGAAGCGGCATTGTTTGCAGCACGTGGTAACAAGCGCAATGTATCTATGGTTGAGTTTGAATTAGCCAAAGATAAAATCATGATGGGTGCTGAGCGTCGTTCTATGGTGATGTCTGAAGAGACTAAAGAGTCAACAGCTTACCATGAAGCAGGTCATGCGATCGTTGGTCGTCTGGTTCCTGAACATGATCCAGTTTACAAGGTTTCTATCATTCCACGTGGCCGCGCGTTGGGTGTGACGATGTACTTGCCAGAGCAAGACCGTGTGAGCATGTCGCGTCAGCATCTCGAGTCTATGGTCTCGAGCCTGTACGGCGGCCGTCTTGCTGAAGAGCTTATCTACGGTGCAGACAAAGTATCAACTGGTGCTTCTAACGATATCGAACGTGCAACTGATATTGCGCGTAAGATGGTGACTCAATGGGGCTTCTCAGAGAAACTAGGGCCACTTCTTTATGCAGAAGAAGAAGGTGAAGTATTCTTGGGCCGTAGTGTAACTCAGACTAAACACATGTCTGACGATACCGCCAAGCTGATTGATGACGAAGTGCGTAAGATCATTGACCGCAACTACGCGCGTGCAAAACAGATCCTTGAAGATAACATGGATATCATGCACGCAATGAAAGATGCGTTGATGAAGTATGAGACCATCGATGCAGGTCAAATTGATGACCTGATGGATCGCAAGTCGGATATTCGTGAGCCAGCGGGTTGGGGTGATAACTCCGACAGCAAGCCGGAAGCTAAAGCAGAGCAACCAGAAGCGAAAGCTGAGCCTGTGAAAGAAGAATCCGCTTCTTCTTCTGAGTCTACAGAAGAGCAGACGACAGAAAAGAAAGATTCTGAATAACGCAAACCAATAAAATAAACCCCGAGGAAACTCGGGGTTTTTGTATTTTTTATGATTATTACAGCAAATAATAAATCCCTCGACTTATCAACGCTTCAAGTTATGGGTATTCTCAACGTCACGCCTGACTCGTTCTCTGATGGTGGAAAATACAATTCACTAGAGAATGCGATGATTCAGGCAGAAAAGATGATTAACGCAGGCGTTAGTATTATCGACATCGGCGGCGAGTCGACTCGCCCTGGTGCACCTGATGTGGCTTTGGAAGAAGAACTTAGTCGGGTGATCCCGGTGATCAAAGCCATTCGTGAACGCTTTGACGTATGGATTTCAATTGATACCAGCAAAGCAGAAGTCATGCGTCAGGCTGTTAAAGCAGGGGCGGACATTATTAATGATGTTCGGGCACTGCAAGAGCCAGGTGCGCTTGAGGTTGCTGCAGAATCGGGGTTACCGGTGTGTCTGATGCACATGCAAGGACAGCCAAGAACCATGCAGGCAAATCCTCATTACGACGATTTACTTAAAGAAGTTGGTGAATTCCTGCAAGAAAGAGTACAAGCATGTGAAGCCAAAGGGATGAAAAAAAGTCAATTGATCCTTGATCCGGGTTTTGGTTTTGGAAAAACGCTTGAACACAACTATCACATGTTGGCTCACCTTGAGCAATTTCATCAATTTGGCTTACCGCTTCTGGCTGGTATGTCTCGTAAATCGATGTTGTTTAAGTTGCTGGATAAAGCGCCTGCCGAGTGTGTTGCTGCCAGTGTCAGCTGCGCGACAATTGCTGCAATGAAAGGTGCTCAGATCATCCGAGTTCATGACTTTGAACAAACGCTTGATGCTGTAAAGATTGTCTCTATGGTGCAAAGTAACCACTGAGAAATAGATTATAAAAAGGAATAATTATGTCTGATAAAAGACGTTATTTTGGTACCGACGGTGTACGAGGAAAAGTGGGTCAGTACCCAATTACACCTGATTTTGTACTTAAGCTTGGTTGGGCAGCAGGACGTGTTCTTGCAAAGCAGGGCACCAAAAAAGTAATTATCGGTAAAGATACTCGTATTTCTGGCTACATGCTGGAGTCTGCACTTGAGGCAGGCCTTGCTGCTGCTGGCTTGAAAGCTACCTTTACTGGTCCAATGCCAACGCCAGCAGTAGCTTACCTGACCCAGACATTCCGCGCAGAAGCTGGTATTGTGATTTCGGCTTCTCATAACCCTTACTACGATAACGGTATTAAGTTCTTTTCTTCGGAAGGTACTAAGCTTCCCGACGATATTGAACTCGCGATTGAAGCGGAACTTGATAAAGACATTGAATGTGTCGAGTCTTCATTGTTGGGTAAAGCCAGCCGTCTTGATG

>NODE_62_length_3168_cov_2.384018

GTGTTGAGGAAGATTTGACCTACTGCCGTTGCATTCTCAAAGTCAGAAACCATCAACTGAGAGCCACCGTTGAAACCAAACCAACCGAACCAAAGGATAAAGGTACCCAGTGTTGCAAGAGGCATGTTCGAACCCGGAATTGGGTAAATCTCACCATTTTTACCGTATTTTCCTTTACGAGCACCTAGTAGTAATACGCCTGCTAATGCAGCGGCTGCACCCGCCATATGTACAATGCCTGAGCCTGCAAAGTCACTGAAGCCCGCTTCTGACAGGAAGCCTCCACCCCAAGTCCAGTAACCTTCCATTGGGTAGATAAATGCCGTAAGTACCATTGAGAAAATAAGGAATGACCACAGTTTCATCCGCTCTGCGACTGCACCAGACACAACAGACATAGCAGTTGCAACAAACACCACCTGGAAGAAGAAGTCAGATTCTAATGAGTGGTCTGCACCTTCCCCTTGTGTTCCAATCAGTGCACCAAATGAAGGTAGCCAGCCGCCTTCGCCATTGTCGACATACATGATGTTGTAACCAACCACCAAGTACATGGTACATGCGATCGCGTATAGACAAACGTTTTTAGTTAGGATTTCAGTGGTGTTCTTTGAACGAACCAATCCCGCCTCAAGCATGGCAAAACCGGCTGCCATCCACATTACCAACGCACCTGAAATGAGGAAGAAAAAAGTGTCCAGTGCGTAACGTAATTCCGTTACTGTAGTTGTTAATTCCATTTTAATGTCTCCAGTCCTTTGAATTCTTAAAGTGCCTCAGCGTCCATTTCGCCAGTACGAATACGTACTGCCTGGCTCAGGTCGTAGACAAAGATTTTTCCGTCACCGATTTTTCCGGTGTGCGCCGCTTTAGATTCAGCTTCAATCACGCGGTCTACATTTTCAGCTTGTGTCGCGATTTCTAGTTTTACCTTGGGTAAGAAATCCACTTGGTACTCTGCACCACGATAAAGCTCGGTGTGGCCTTTCTGACGCCCAAACCCTTTCACTTCTGATACCGTCATACCTTCGATACCGACATCAGCAAGCGCCTCTCGAACATCGTCTAATTTGAATGGTTTGATAATCGCATTAATAAGTTTCATCGCATCCTCTCTGAAGCTGTCATCCTTTTTATCTTTATAATCCAAGTGACATGCCAACTTATTAAGACACTGATAAATAATGATTTATCAGTCAAAAGGAGCCAGAAACGCAAAAAGGCGCACCGTTATGGTGCGCCTTTATCACAATAATGAGGCAATGATTTCATTCTTCACTATATCAGTGCAGATCTATTTGGTGGTCAGAAACTCTTTCCATTGATCAATTAACAGCTCAAAGTCATCTAAGCCACAACAGGCCGTACTCTCACTGTCATAAAAATCAAACTCGCTGTCTTCTGCCATCGCTTCTCCATGCGCCAACACATTCTCCTGAATGGTGACTTCATCACCATGAATAGAGATGGAAATCTCAGTACCCAGCATAGTGTGTTCCTGAAGTGGAGACTGGTGTACAGCGACTAGCATAGACTCGACCTGCTCTATCTTCTGCCAATCCTTACCGATCTCTTCCTGCAACCAGCGACCAACAATTTCATGGCCCATACTCGACTTAACGTAGTATTCGCCCATCAATGTATTGCGGATAAATTCAAATTCCATCGTGCTGCCTCATGAAAACGGAGCGCAGAGTATATAGAGACAATCCTAAAAGCACAAAAAAAGCGCCTGCCGAGGCAGACGCTTTCATCATCCTTTCTAAGCGCTTATGCAGGCTCTTGGAAAATCACTGAATCCGCTTTCTTGGTGTACTGATCCATCTTATGGAAGTTCAAGTAACGGTATGTGTCTGCTGCTGTCGCATCGATTTGCTTAGCGTACTCTAGATACTCTTCCTTGGTTGGAATCTTACCTAGAATCGCACCAACTGCCGAAAGCTCAGCAGAAGCTAGATAAACGTTAGCACCAGTACCTAAACGGTTCGGGAAGTTACGTGTCGAAGTCGACATTACTGTCGCCTTATCTGCCACACGTGCCTGGTTACCCATACATAGTGAACACCCTGGCGTTTCAATACGCACGCCAGCACGGCCGTAAATACCGTAGTAACCTTCTTCAGTTAGCTGATCGCGGTCCATCTTCGTTGGTGGCGCAACCCAGAGGCGAGTATCTAACTGACCACCGAATTGCTCTAGCAGCTTACCTGCAGCACGGAAGTGACCGATGTTAGTCATACAAGAACCGATGAAGACTTCATCGATGTTTGTACCCTGAACTTCAGAGAGCAGACGGGCATCATCCGGATCGTTTGGAGCACATAGAATTGGCTGATCAATTTCTGCTAGGTCAATTTCAATGATGTGAGCGTATTCCGCATCTGCGTCTGCTTCCATCAACTCAGGATTCGCCAACCACTCTTCCATCGCCGTGATACGACGCTCAATAGTACGACGGTCACCGTAACCTTCAGAAATCATCCATTTAAGCATGGTGATGTTCGAGCTTAGGTACTCTTCAATCGACTCTTGAGACAGCTTAACTGTACAACCTGCCGCTGAACGCTCTGCAGAAGCATCTGAAAGCTCAAATGCCTGCTCTACGGTTAGGTGTTCAACACCTTCGATTTCAAGTACACGGCCAGAGAATTCGTTGATCTTACCTGCTTTCTCAACCGTCAATAGACCTTGCTGAATTGCGTAGTAAGGAATCGCATGAACCAAGTCACGTAGTGTGATACCTGGCTGCATTTCACCTTTAAAGCGAACCAAGATTGATTCTGGCATATCCAGAGGCATAACACCTGTCGCTGCAGCAAACGCAACTAGACCAGAACCAGCTGGGAATGAAATACCTAGAGGGAAACGAGTATGTGAGTCACCACCTGTACCAACAGTATCAGGAAGAAGCATACGGTTTAGCCATGAGTGGATAACGCCATCGCCTGGACGAAGAGAAACACCACCACGGTTCATGATGAAATCAGGCAGAGTATGGTGCGTGTTTACGTCGACTGGTTTTGGATACGCAGAAGTATGACAGAAAGACTGCATCACTAGATCAGCCGAGAAGCCAAGACACGCAAGGTCTTTTAGCTCATCACGCGTCATAGGGCCTGTTGTATCCTGAGAACCTACGGTCGTCATCTTAGGCTCACAGTATTGACC

>NODE_63_length_3155_cov_2.511371

GGAATATAGCGGCTGTATAGCTCACCTTTTTGAGGTCTTGGGCGAAGAGGATGGTAGCCATTAGAAGCAAGAATCTGCTTATGTGGAATAGCGCGACGCTGCTGTTTTAGCCACAGTTCGGGCAACTGCAGTAAAGCTTCTCGCCAAACCAGACGAATACCTTCACCTGTGACATGAGTGATGTCGTTTAACGATGAGAAAGTAAACTCGCCGAGATCAATACAACGAATATCCGTACGATGCTCAAGCATAAGCACAAAAGCTTGAACCACTTCCTTATCGTTGATGGCACTGCTGGTTACACGCCAATAACCACCACTTTCCTTGCTAAGTGTTAACTGACCGCTTGGCATCTCAACGCAACATGTATCCCCTTGTTGCTGAACACTCTGCCCAAAGGGCAGAGTGTTGAGGGTATCACTGTTACCAGTCATAGCTCAGGCTCGCGATAACATTGCGGCGATCACCGTAGAAACAGTTGCTACTGGTACAAGTAGCGATATATTCCTTGTCAAAGATGTTCTTTGCTGCGACTTGGAATTTATAGTCATCAAATCGGTAACTAACTGTTGCATCGTACAGTGTGTAAGATGGCACTGTATTGGTATTGTTGTTACCAGCGTATGTATCATCTGTATAGCGGAAGCCTCCACCGATACTCATACCGTCAAATGTGCCACCGAAGAATTGATACTTCGCCCAAGCGGACGCCAAACTGTCTGCCACTTGTTGAGGTTGATTACCTATAATAGAAGTATCCGTATCATCTTTGATTTCAGAGTCCACATAAGACAGGTTACCAATTAACGTTAAGGCCTGGGTTACATTGGCAACCGCTTCTAGTTCCATGCCTCGATTGCGAATCTCACCTACTTGCTTGACTGCAGCACCATCAATTTGAACAACGTCTTTCTTAGTGGCTTCATAAACAGATACGTTAAAGTAGCCGTCAAAGCCTCGCGGCTGAAACTTAACACCCGCTTCGAAAGCTTCACCACGCTCTGGTTTCGAGGCTTGATTGTCGACACTTTTAACCTGAGGTTCAAAGGATTGAGAGTAGTTTGCATATGGAGTAAAGCCGTTACTCATTACATAAGCAACACCTACGTTAGATGTCCACTCTTCATAATCTGCTTGGTACGCATTGCCTGTCGTTTGATTACTTTGCTTATTCGAAGTGTCATCGTAACGAACACCAGCTTGTACTACCCAGTCATTGAATCGCACTTGATCTTGTAGGTAAAAACCAAGCTGTCGATTTTTGGTAACCGTAGTTTGAAGGTCAGCGTCTGTTAATTGTTGGAATGACGTAGGATGAAGCAGGACCACATTGCTGCTATAACTTGGATTAAAGATATTGAACCTTGGCTCTGCAATCGTGCCAACTAACGGGATGGTGAAAGAACCATTGCCATCGGCTACAGTAGGATCAGCTGCATAATCCTGACTATCTATTTCAATTTGTTGATAATCAAAACCCGCTAGTAACGTGTGCTCAACATCTCCAGTTTGGAATTGATACACTAAGCGGTTGTCCACATTAAATGCATCAGACTGCCCTTTATCAGTGGATGCCCTTCTCACAATTGTTTGTCTTGAGCCAGTAGGATCTAGCAGCGGCCCCAACTGATCCACATCAGCCGCATACGCCGCGAAATACATCTGACGAAGTTCAATATCCATACGGCTATAACGAGCAGATTGACCAAAGCTCAAAGCGCTATTGAACTGATGCTCAAACTCGTAACCAAAGGAAAGTTGCTCGCGTTCAAACTTTTCCCAATCAGTATTACCAACTGCTACATCATCACTAATCTGGCCGTTAGGATTGCTGGTCAATGTACCTTCGACAGGCAAAAACTGTAGATAAGGGTTTGAATCGTCTTTTTGGTAACTTGTCAGAAATGTAATACTGTTCTTATCGTTAATATTCCACGCTAATGATGGTGCAACTAGAATACGCTCGGCATCAGCATTGTCGACTTTCGTGCCATTCTTACGCCCCATAGCGACAAGACGGAAAGCAACATCTTCATTAAGTTCACGATTGACATCTAGGCTAATCTGAGTTCTGTCATATGAACCATACTCAACTGATACTTGACCTGAACCACCATCAAACTGAGGACGTTTGCTTACCGTATTGATAACGCCGCCCGGTGGGTTCTGTCCGTATAAAACTGATGCAGGACCACGAAGGATTTCTACACGTTCTAAACCAAACGGGTCAATCTGCCAGCTGTAGAAGCCTGCCGAATATAGGCGCGTACCATCTTGATAAAGCCCGTTATTAGCTTGCTGAAATCCGCGTATAACAAACCAGTCTTGCTTATTGTCATCGCCGTAGAAGTTGGTCTGAATACCTGGGGTGTACTGAAGTGCGTCTGAGATGCTGATTGACGCGCGGTCGTCCATTTGTTCACGAGTAACAACAGAAACAGCTCGTGGCGTTTCATGGATTGCTAAATCTGTTTTGGTTGCTGTACGACTTTTTTGTCCTAAATAACTGAAATCTGGGCCTACGGCACTATCGTGCTCTTGACCTACTACCACTACTGTTTCGTCTTCCGCTGCGTTAACTACGCCAGCAGACAGAGCGAACGCTACTGCCAAACAAGCCGGGCTCAGCATAAACTGCCGATTCATGTCTTTCTCCTTAACTCTCGGTTTTACCGAATATTATATTAATAATTATGATAACCATTATCATTAGCATTTCGAATGCGAATGGAGTGTAACGAGTTTTATAGAAAAATGGAATCTATAAATTAGTCGTTATGGATCTATAATGTATTGATTATTAATAAGTGGCGTAATTTCTCGATTAGCGTACTGTCTTGCACTATTCCTAAATGATCCGTATCCAGCACTTCACTGCTTAACTGAGCGTTAACTTTCGACAGTTCCGAACTCAGCAGTTTGTGTCGACCTTCAGGTTGCCCTGCCCAAAATGCGGTTGGTTTGGTATTGATATTAGGTAACTGATACTCATGACACAGCGCTCTCATGTGCTGCTCTACCGCCCAGCTGTAAAGCATTTGCTCTGCACTTTCCGACAGGTAGTGATCGAAGTTTTGTGACGCTAACCATGATGAGAACACGTTTGGCCATTTATCCGGTGATGATTGGTCAAACCCTGCAAGACAGCTTTGCTT

>NODE_64_length_3149_cov_2.675130

CCCACCATTGGCGTTTCACCCGTCACAGCTCCTAACAATGTTGACTTACCGGCGCCATTAGAGCCAATAACCGTCAAAAATTGATGTTCAGGGACTTCCAGTGAAACACCTTTCAGTGCTGGATTTTCAAGAATGGTTCCAGGATTAAAAGTGACTTGAATGTTCTCAAGCTGAATCATGCTGCATCTCCTGATTTTTCTACCTGCTTAGATATGACTTTGTTCTGAGCAGGTGAACTAGGCTTTTTTGATTTGAAGTTTCCTTTTAGCTTCGGCGCAATCAGCGCAATTGCTACCAAAATCGCTGTAACCAAGTTAAGATCAGAGGCTTGTAGACCAAACATGCCTGTACTTAATGCAAATGCTACAGCCAGTCGATATAGCACAGAGCCTACAATAACAGCCAACACAGCCACCCATATCTTTCTTCCAGGAATGAGCGTCTGTCCCAAGATAACTGCAGCAAGACCAACAACAATAGTCCCCACTCCAGAAGTCACATCTGCAAAGCTGTTAGTCTGGGCAAATAGAGCACCAGCAAAACCAACAAAGCCATTTGATAGAGCAAGACCAAAATAAGTGTAAAAGGACGTGCTGCCACCTTGTGCGGATACCATACGCGCATTAACACCCGTTGCACGTAAACCTAAGCCAAAATCACTGTTAAGGAGCCTAACAACAAACCATGCCGAAACGAGCACGAGTATCCCAACCAACAATGGCCGCATTAGCATCGGGTCACCAATTGCTTCAAATGGAGTCAGAATCGTTTCTTCCCCCAATAAGGCCATATTCGGGCGACCCATAATGCGAATGTTGATCGAGAATGCAGCGATCATGGTAAGAATGGAGGCGAGAAGATGAAGGATCCCACACCTAACCGCTAGAAAAGCCGTCACCCAGCCGGTTGCCGCTCCCGCTAAGATAGCCATTGCGGTTGCAACCCAAGGGTTGATACCCGCAACGATTGCCGTCGCAGCCACAGCGGCTCCCATAGGGAAACTGCCGTCAACACTGAGGTCGGGGAAATCCAATACCCGAAAAGTCAGGTAAACGCCCAATGCCACTAACCCGTAAAGCAGGCCAATCTCAAGCGCACCAAAAAAAGCAAAAGCAGACATAACAACTCCCTTTTCTGCCTTAAAAAAAAGAACGCATAGCGCGTTCTCTTTTTGGGTTACTTAACGCTAGTTGCGCGATCAAGTACTGATGCTGGAATCGTCACACCCAGCTTTTTCGCCGCCGTCTCATTAACGACTAAATCAGACCCTTTGGCAACTTTAACGTCTAATGAACCTGGCGCCGAACCTTCAAGAATCGCCGCAACATAGTCTGCTGTCTGAACGCCAACTTGATAGTAATCAAAACCTAAGCTAGCAATCGCGCCTTTTTCAACATATGAAGTCGCACCACCCAGAACAGGTGTCTTGGCCTGATTCGCCGCTACAATCATACCTTCAATCGCACTGGCAACAGTATTATCTGTCGGTGCATAAATCACATCAGACTTCTCCGCAATTGCCTGAGTCGCAGACTGGACATCAGCACTCTTTAGCGCCGTAGCTTCTACAATTTCAATACCCTGCGCTTTTGCACTCTCTTTGAGTAGTTCCACTAAAGTGACAGCATTCGCTTCTCCAGGGTTAAACACAACACCGATGGATTTCACATCTGGCATGATTTCCTTAATCAGTTCGACATGTTGACTCACTGGGGAAAGATCAGATAAGCCCGTGACGTTCTTTCCTGGTTTCTCCATGCTTTTGACTAATTTAGCGCCTACTGGGTCCGTAACGGCCGTGAATACAACAGGAATTGAGCGCGTTGCTGATACTAATGCTTGTGCGGTCGGAGTAGCGATACCAACTAAAACGTCAGGCTTTTCACCGACAAACTGACGAGCAATCTGTACGGCAATCGCAGGGTTGCCTTGGGCCGTTTTGTAGTCAAACTCGAGATTTTTTCCTTGCTCATAGCCCTTTGATTTCAAGCCATCAATAAGCCCCTGGCGAGTGGCATCCAGTGCCGGGTGTTCAACGATCTGTGATACAGCTACTTTGGCTGTCTTCGCCATGATGCTTGTTGATGACAGTAGTGCTGCTCCTGCCAATACAGCGGTAGCAATAACTTTTCCTGCTTTCATCTCGACTCCTTGATTACAGCATTATTGATGTGATGTTGTGTGCGGTTAGTGTTTATATAATTATTGATTGAGTTGCACATTTTCTGTGCACATCATTCATTATTACCAATAACTTAGAAAAATTGGAAGGGAGTTTTAACAATTAATCAACAAACTTAGAGGTGTGTATACGGCTAATTTTTAATCGCCTGAATCAGGATATTTCTCGGCGTAATTTTTTTAGCACAAAAAGCTTCAACAGATACTTGGTAACCATGCTCTGCAAGATACAAAGCCTTATCTAGTACCAACCACATTTCCAGACTGCGACGAAACTGTTGTTGAACAAGGCTGAGCCTCTCCATTTTCCAATAACGTTGTTCACCTTTCGCTTCAAATTCCGCGAACGACACATTCGTTGGCAAATCCAGCTGTTTCTGATCGGCCGCCCATTGGCAAAAAGCGCCGAATCCATCGGCCAACAAGGACTTTTTAACACTTGGTACTGGTATGTAATCTTGATGACCACCGACTTCTTTCAATAGCAGGTCCAAACCGAGCCGGTAGCTCATTTCGAGAAAGCGATGCCGTTTAACTCGCTCACCACCTGTAACGGTTTCCTGAAGTGGTATACGCAGCTCTGGCTTTGTTAGCTTAAGTGCTGACGAACAAGCAAGCTTAGACAAAGGGCAATACTTATCCTGTTGGATAAGGTGGTAACAACAAGGCGAGATGCTTAATGCTGGCAATCCAACCGAAACTGCCTTTTCAATCAAAGAAACATGCAAATCGCCGCATGCATGTAATGCAACCGCATGCTGTTTAGGGTTAAGTGCTTGCTTTGCGTCAGCGGAAAGCGCATCTCCTTGGACAAACCTCATGGGTAAGTTCTGTTTATCCGCTTCTTGCTGACCCGAATCACACAGCATTTGTTGAAATTCGAAGCTGGTCACTGGCTGCTGACTTTGACTGGCAAGTATACGGCCCAAGAAGCCTTTACCTGAGCACCATTCAAGCCATTCACTGCC

>NODE_65_length_3136_cov_2.277542

CACCTGAACCATCGTAGTTGTAGGTGTAGCTGTTGCTGAGTGGCAAATCACGTTGATCGATTCGATCTAATCCCCAAACTGGGTTAGTCTGAGTCGCGTTGGCGGAGACGACAGGATCAAGTGAGATGATTTGGTCTTGCTCAATGAAGTCAACTTGTTGATCTGCACGAAGTGCTTTCAGTTGTTCAGCCGAAAGAGTCGCTACAAAACCACTTAACGAACGGTCATAAACTTTATCGATCTTAAAGGCGTGTTGGCTAGCTAAACCAGAAACGGTTTGCTGCGTAAACTGCTGAAGGGCTTGTTGGTCGTTACTCATCGTTAATGGCTGTTTTAGCACAACGATGTATTGATTTTTAATCGCAGATTCTGCCGACGCCGTCATTAAAGGCGCAAGCATTTGATCCTCAGATGCAATGGCTCCTCCTTCGTTCTGTTGCGCAAGCCCCGAGGTTGAGATCACTGAAAGAGTCGAGGCGATACAACAACTTAATAATTTCTTAAACATACTGTCTTCCTTTGAATAGTAAAAGAGATTGTTGAACATGGCCTACTACAAACATCAAGCATGTCAGGTGGGTTCTCGTGGTTCAAAGAGAACCTGACTACAGAATTCACACTATGCATATATCCAATATGTTGCATATTGTTGTTTTAGTTAGTCTATAAATCTGCGCGTGGTTTATAAGATTATTGAAATTGACTTATTACTATTTCTTGAATCGTGGGATAAGAATCATAAATGAGAGCTTTTGCTCTATGTGGTGGAATCTACGTGCTAAGGCAATAGTGATTTATTTTGGATGACGGGTTTGCAGAAGGCCCCTGTTAGACAAGAGGCCTTACTGAGGCTTAAATCAGTTGCTCAGTGTTTATCAACGGAACACGTGTAGAGATGGCATGAAGGGCGATATTATGAATCATTTCATCTGTGGTGGTGCAACAATCGGCCAGCACAGATACTTCATATTGCTCTGCTGCTTTGGAAATCGCAGTATGAGTCACGCAGTTTTGCGTCATCATGCCGCAAACAATCAATTCAGTTACCCCATGCTTTTTGAGCACATTTTCTAGCTCTGTTTGTTCAAAGCTGTCTGCGAAAGATTTAACGACAATATCCCCTTCCGGTGCGCTTGCGAGGATCTCTGAGTGGATATCTGCACCTGTACTACCTTTGTTGAAAAAGGGGGCGATACCCATAGCAGGGTCTGCAATATGCTGAATGTGAATCACAGGAATCTGTTTCTGTTGGGCTTTTTTAATCGCTGCTTTGATATTGGTTAACGTGTTCTCTGTGTTCCAAAGAGGGAATTTCCCGGTTGGAAAGTAGTCGTTTTGTAGATCGATGACGAGTAGTGCTTTCTGGCTCATTTTGTCTCTCCTGTTTGGTATCGTTCAAAGTAGAGTCATTATGGCGCTTTATTTTTTGGCTTTTTAGAGTCTAAAATGACACAAATAATGCTAAAAACGACATTGTATGCCTAAGATTAATATTGCCGTATTTAACTATCCTTTTGCTCTCAAATCGGCCGTATTTGGTTTTGAAGAGCTTTTTCAGTTAGCCAACCGAGCATGCCGTGAGAGTGAACTTGAGACGGAGTTCAAAGCGCACATTATTGACGCGTGTAATATGCCTCCCAAGCAATTTGATGTCGTGCTGCTCCCGCCCAGCATTGACGATCAGTTCTACCGAAAACCAGAAGCACCAGTACTCGAATGGCTGGTTGAGCAACACCGACGTGGCAGTGTGATTGCGTCGGCGTGTGCTGGCGCGTTTATCTTGGCGGCGTCAGGGATTGGTGGACAAAGGACTCTGACTACCCATTGGGGGCTAGCTGAGACCTTTAAGTCACAGTACCCATCGCAGCCCATGGAGATAAATCAAATTTTAATTGATCATGGCGATGTGATTACAGCTGGTGGCATGATGTCTTGGTTGGATCTCGGACTGGAGTTGGTATCCAGGTATGCTTCACCACAAATCATGCGACAACTGGGTAAGATGTTGGTCATCGATACTGCGCCAAGAGAACAGAGGTTTTATCAGCAATTCACGCCATCATTCTCTCATGGTGATATTGCAATTGTTGAAATTCAGCACAGAATCAGTGCCTGCTTTTCTCGGACAGTGTCGGTAAAACAATTGGCTGTTGAAGCAAACTTGACCGAAAGAACTTTTCAGCGTCGATTTATGAAAGCCACAGGCATGAATCCAAACCAGTATTTGCAGCGAGTACGGGTGCAGAAGGCTTGTGACTACCTGGAAGGTTCAACTCATTCTTTTGAGTGGATAAGCCATCAGGTAGGGTATGAAAACGCGAGTGCATGTCGCAAGGTATTCAGTCAGATAATGGGGCTGACCCCGGGGGAGTTTCGTCGGCGCTTTAGACGATAAATAAAAAGCCCGCAATGATTGCGGGCTTCTGGGAGTTGGAGGCTTACACCTTAAATCTGTCTACCAATTCGTACAGCTCATTGGCGCGTTGATTCAGCTCTTGGCTGCCTGAACGGTTTTCGTTGGCCAGTTCAGCCGATTGAGAGCTCTGGTCAGAAATCACCACTATGCGCTGTGAAATTTCCTGGCCTACCAAGCTTTGCTCTTCGGTCGCGGTTGCGATTAGGGAGTTCATATCCATGATGGTGCCGATGGATTCCTGAATCTGAATCAGCGCCTGAGCAGCAGCATTGGCTTCTGCTACCGTGCTGGCCCCACGCTTCTGGCTTGATTCCATGGCTCTTACCGCGTCGTCCGATGCAGCTTTTAATTGTTCAATCATCTGGTGAATCTCACCAGTACTGTCTTGGGTACGACTCGCCAGTTTACGAACTTCATCGGCAACAACTGCAAATCCACGACCCTGCTCACCAGCACGAGCGGCCTCAATGGCTGCGTTCAGTGCCAGCAGATTAGTTTGTTCTGCGATGTCTTGAATCACTTCCAAAGAGGAAGAAATGTTTTGCACATCACCTTCAAGACGAGAAATAACGCCATTCGCCTGAGAGACTTCTTCGGCAAGCGCTTCCACCGATTGGGCGGCAGAATTGACGATGCCTTGTGCTTCTTTGGCATTGTCATCAGCTTCTTTGGCAGAATGCGCCGCTTGGCTGGCATTGTTCGAGATTTCAGATGCTGTTG

>NODE_66_length_3130_cov_2.157222

TCACTGTATTTGAGAGAAAACAAATGTATTCTGTAGAGCCGATTGGTTTTATTGAAAGTCCTTATAAAGAGAAGTTTGCCGTACCAAGGCAGCCTAGACTGGTGCCTGAGGCAAAAGCGAGACTTCGTTTAGTGGGCGATGCAAATTGTGCAGAAGCGATCAGAGGCATCGAGCAGTTTAGCCACCTATGGCTGCTGTTTATGTTCGATCAGAATTTGCAGGCCGGATGGAAGCCGACAGTACGCCCACCAAGATTAGGCGGAAACGAACGCATTGGTGTGTTCGCATCACGCTCGACATTCAGACCAAACGGGATTGGCATGTCTGCGGTGGAACTCAAAGGCATGTCACAGTGTGGTGACCAATTTTATATCGAGCTTGGAAGTGTTGACTTAGTTGATGGTACCCCTGTTATCGACGTCAAACCTTACATCGAATACTCAGATGCTATTACTGATAGCAGTAACGGCTATGCCGACGAAGCGCCGTCCACCTCAGAAGTCGTATTCACCGATAGCGCGTTAGCGACACTCAGCCTCCTTCCTGACAGCCAACACGTCACTTTGGTTATTGAACAAGTGCTGGCTCAAGATCCTCGTCCCGCTTATAAGAAGAATAAGCCCGACAATAAGGAATACGCGGTAAATCTATTCGATCTGAACGTGAAATTTCGTGTTGAAGGCAACTTAGTGACGGTTACGACCATTGAACGCTTTTGACAAAGCCAATTGGCTGATATTATAGGCGGCTAACTATTTTCCCTTGATGGGATCTCTTTACTCGAGCTCGTTCTGGCTAGAGTATCTAACTTAAATAAACGGATAAATTTAATGCGTACCAGTAATTATCTTCTTTCTACTCTGAAGGAGACTCCAAACGACGCAGAAGTAGTGAGCCACCAGCTCATGCTACGTGCAGGTATGATCCGTAAGCTGGCTTCAGGTCTATACACCTGGCTACCTACCGGTCTACGTGTACTGCGTAAAGTCGAAAACATCGTTCGTCAGGAAATCGACAATGCAGGTGCAGTCGAAACTTTGATGCCCGTTGTTCAGCCGTTTGAACTATGGGAAGAGACAGGCCGCAGCGAAAAGATGGGTCCTGAGCTACTTCGCTTTACTGACCGTCATACTCGTCCGTTTGTTCTTAGCCCTACCGCTGAAGAAGTCATCACTAGCCTTGTGCGCAATGAAGTAAGCTCTTACAAGCAGTTACCGCTAAACCTGTACCAAATTCAAACTAAGTTCCGTGATGAGCGCCGCCCACGTTTCGGCGTAATGCGTGCACGTGAATTCTGCATGATGGATGCGTACAGCTTTGATATCGATAAAGAAGGTCTGCAGAAGTCTTACGATGCAATGCACGATGCTTACTGTAAAGCGTTTGACCGCATGGGTCTTGAGTACCGTCCGGTACTGGCTGACTCTGGCGCAATCGGCGGCAGCGGTTCTCAAGAGTTCCACGTACTAGCAGAAAGCGGTGAAGATCTGATCGCATTCTCTACTGAATCGGATTACGCAGCGAACATCGAAAAAGCTGAAGCGCTAGCTCTGGCTGACGAAGCTGCTGCTCCAACTCAAGAGATGGAACTTGTTGATACACCAAACGCGAAAACCATCGCAGAGTTAGTCGAGCAACACGGTCTAGCAATTGAGAAAACCGTTAAGACTCTATTTGTTAAGGCGTCTGATGAAGTTGAAGCTGACCTAATTGCGCTTATCATCCGTGGCGACCACGAACTGAACGAAGTGAAAGCGGAAAACCTTCCTCAAGTTGCAGCTCCTCTAGAGATGGCAACAGAAGAAGAGATCCGTGGTCTAATCGGTGCTGGCCCTGGCTCACTAGGCCCTGTGGGTCTTGAACTGCCATTCATCGTTGATCGCTCTGTAGCAGTCATGAGTGACTTTGGCGCGGGCGCTAACATTGACGGCAAACACTACTTCGGTATCAACTGGGGTCGTGATGTTGAGCTAAGCCAAGTTGAAGATTTGCGTAACGTAGTTGAAGGCGATCCAAGCCCTTGTGGTAAAGGTACTATCGCACTAAAACGTGGTATCGAAGTGGGTCACATCTTCCAGCTAGGTAACGTTTACTCACAAGCCATGAACTGTGGAGTTCTTGGTCCAGACGGTAAGAACGTTATTCTAGAGATGGGTTGTTACGGCATTGGTGTTTCCCGTGTTGTTGCTTCTGCGATTGAGCAAAATCATGACAAGTACGGCATCATATGGCCAGACGCACTAGCACCTTTCCAAGTGGCTATCGTTCCTATGAACATGCACAAGTCAGAAGAAGTAAAAGAAGCGGCTGAGAAGCTGTATGCTGAACTGACGGCGATGGGCATCGAAGTACTATTCGATGACCGTAAAGAGCGCCCGGGTGTTATGTTCTCTGATATGGAACTTATCGGTATCCCTCACACTATCGTGATCGGCGATCGTTCGATGAAAGAAGGAAACTTTGAGTACAAGAACCGCCGCACTGGTGACAAGACACCTGTTGCGATGGCTGACATCGTTGAACACATTAAAGCTCAACTGGCTTAAGTTGATTAACGCTTAAAAACAGAAAGGCTGATGCATTGCATCAGCCTTTTTTACTTGGAGTTTCCATCAAGCCCAATCTGTTGTCTCACTCCAACGGCTCAGTTGGCGCCTGACTTTCGACCTTTTTCGGAGCAAATGGCACGTCAATGAGCTCATCGGCATTGCCATCGATAATTTCACCAAAATGTAAAATGCCAAATTCCCCAGGCTTCATTCGATGCCACTCTTCATTATCAGTTAGCGGCTGCGTCGCGATAACTGAAACCACGTCATTGGGTGTCGTTTCTTCCTGAAAATTAATCTCGACGTCTTCATCAATTAAAGAAGCTTTACCAAATGGAGCACGACGAGTAATCCAGTAGAGATGGTTGGTACAATACGTCATGACATATTCACCATCTGACAACAGCATGTTGAACACCCCTTTACTTTTCAGGGTTTCACAACATTTCGCCACAAAGCGGAACACACCAATCATATCCTGAGGCGGCTCTGGAAATCGGTCTTCCATCTGCTTAAGCAACCAACAGAAAGCTAATTCACTGTCTGTCTGACCCACAGGTCGATGACGCCCCGTATGCAGA

>NODE_67_length_3124_cov_2.427634

GTACTGATCTACCACCTCAAAATGCGCAACAGCAGCAAAGATGAAATGATTCCGGGGCTGAAAAAAGATCAGGAAGACGATTTTAAAACCGCGATTCTGCGGGCGCGTGGCATCATCAAATAATCAGGCATAGGCCAGAGCGACCTTAGGGTCGCTTTTTTTATATCCAAATCATTTAGACGGGCAAAACTGTTAGGCAATTTATTTATAAAACTGTACTGATCTTTGTTGTTAAAGGTTGATAGCCTTAAAGAAATAAAAAAATGATTGCGTATATAATCGCCGTCCATAAGAAAATACTAAAATATTAAAGAGCAAAGTCGCAGAGCGGGATCGCTAAGAAATCTCGCCTTTAGCCCAAGAAGGAAGCGTAATGAGTCAGGACAAAATTGAGATAAAAGACGTGACTCCCAAGACCTTTAACCCCAAGACGCACAAAAACACTGGGGACAGGTTTAACCCGAGCAACCGCATCTACGTGCGTGAAAGTAAGGGCACATTTCAACAGTTACGTCGCTACGGAGGCTGGTTGCTACTGGTTCTCTTCGCTATGGTGCCATGGATTCCTTACGGGGAACGTCAGGCAGTTCTATTAGACCTTGGTCATCAGCAGTTTAACTTCTTTGGTACCACACTTTACCCGCAAGATCTGACCTTGCTGGCATTGCTGTTCATGATTTCCGCGTTCGGTCTGTTCTTCCTCACCACCTTCTTAGGCCGAGTATGGTGTGGCTATTTGTGTCCTCAAACCGTCTGGACCTTCATGTACATCTGGTTTGAAGAGAAGCTAGAAGGCAGCGCCAACAAACGCCGCAAACAAGACTCAGGCAAGCTGACCGCTAATCTTGCAATGCGTAAAGGACTTAAGCACCTAGCATGGTGGGCCATTGCACTGGCAACAGGTTTCACCTTTACCGGCTACTTTGTTCCGATCAAAGATCTTGTACTCGGGTTCTTCACCTTTAACGCTGATTTCTGGCCTGTGTTCTGGGTGCTATTCTTTGCAGGCTGTACCTACGCTAATGCAGGTTGGATGCGCTCGATCATGTGTGTCCACATGTGTCCGTATGCGCGCTTCCAGTCAGCCATGTTTGATAAAGATACCTTCATCGTTGGCTACGATACAAAACGAGGCGAATCTCGTGGTGCTCGCTCACGTAAAGCCGATCCAAAAGCTCTGGGTTTAGGCGACTGTATCGACTGTAACCTGTGTGTGCAGGTGTGTCCGACCGGTATCGATATTCGCGATGGCCTGCAATATGAGTGCATCAACTGTGGCGCTTGTATTGATGCCTGTGACAATACGATGGATCGCATGGGCTATGAAAAAGGCCTGATCAGCTACACCACAGAGCATCGTTTGTCAGGCAAAAATACCAAAGTCATGCGCCCTAAATTACTTGGCTATGGCGCGGTACTGTTAGTGATGATCGGTTTGTTCTTTGCTCAAGTGGCTGCCGTTGATCCAGCAGGTCTAAGCGTGCTGCGTGACCGTAATCAGTTATTCCGCGTCAACAGCTCAGGCGAAGTCGAAAACACCTACACACTGAAGATCATCAACAAAACTCAGCAAATTCAAGAGTATAAGTTGGATGTGTTAGGGCTCACTGATGTCTCTTGGTATGGCAAACAAACTGTCCACGTTTCACCGGGTGAAGTGTACAACCTGCCAATGAGCTTAGGCGTCAATCCGGACAATCTCAGCTCTCCTGTTGCGACAATTCAGTTTATACTGTCCGATAGCGAAGACTTTACGATTGAAGTAGAAAGCCGCTTTATTAAAAAGCTTTAGCGGTTACCTCTACCTAAAACATGGAAAAGGGCTCAGCAATGAGCCCTTTTTATTTATGACACAACAAGCCTTTAACTTTGATGCTCTGACACCCGACTTTATGTGGTACGCCTTGGAAAGCATTGGCGTTCGCGCCGAATCCGGCTTTCTACCACTCAACAGCTATGAAAACCGTGTTTATCAGTTTACCGATGAAGAGCGTCAACGTTATGTGGTGAAGTTTTATCGCCCCGAGCGCTGGAGTTCAGAACAGATACAAGAAGAGCACGACTTTACTCTGGAGCTGATTGAATCCGATATCCCGGTTGCCCCACCGATAAAAATCCAAGGGCAAACTCTGCATCAATATCAGGGGTATCTGTTTGCACTGTTTGCCAGTGTTGGCGGACGCCAGTTTGAAGTGGATAACCTCGAACAATTGGAAGGCGTAGGTCGTTTCCTTGGTCGAATCCATAAAGTCGGTGCTCGAATGCCCTTTAAGCACAGGCCCACCATCAGCCTCGAAGAATACCTGCATCAACCACGTAAGATTCTGGAGCAATCACAGTTTATTCCATTTCATCTGGAGAATGCCTTCTTCAATGACTTAGACTTGTTGATTCAATCGCTGCAACATCAATGGGATGACTCAGCCAACATCATTCGCCTTCACGGTGATTGCCATCCGGGGAACATTCTCTGGCGCGACGGCCCAATGTTTGTCGATCTTGATGACTCTCGTAATGGACCGGCAGTACAAGACTTGTGGATGATGCTTAATGGCGAGCGTGCCGATAAACTGATGCAATTAGATATTCTGCTTGAGGCGTATCAGGAGTTTAACGACTTTAATACCAACGAATTGAAACTAATTGAACCTTTACGCGGTCTACGTATGGTGCACTATATGGCATGGTTGGCAAAAAGATGGCAGGATCCTGCTTTTCCTATCGCCTTCCCGTGGTTTGATGATCCAAAATACTGGGAAAGCCAGGTATTAGCTTTCAAAGAACAAATTTCGGCATTGAGTGAGCCACCACTCTCACTGATGCCTCAGTGGTAAGAAGCCAAAATGACAAACATGGAGTCAATAATGAAAAAGCTGTTTGCACTTGTCGCAACTGTTATGTTGAGCCTTTCTGCTCACGCCGCTCAATTCAAAGAAGGTGAACATTATCAAGTTCTTGATATTCCTTCAGCTTCTCTCAGCCAGGCTTCACAAAAGCCAACCGTGACCGAGTTTTTCTCTTTCTACTGCCCACACTGTAATACGTTTGAGCCAATCATCGAACAGCTAAAAGCTCAACTTCCAGAAGGGGCTAAATTCCAGAAAAATCACGTTTCCTTCATGGG

>NODE_68_length_3116_cov_2.188549

GCATGGGCTTGGGGCGCTCGGGTAATTCGTTCACAAACTCTAGCTCTACGTGAGAAAGAGTTCGTTAAAGCCGCAGAAGTACTCGGAGAATCGTCTTGGCGCATCATCTTTGTCGAGATCCTACCTAACCTAATCCCAATTGTGGGCGCAAGTTTCATTGGTTCAGTGATGTACGCCATCATGATGGAAGCAACGATTTCCTTCCTTGGATTAGGTGACCCTAACACCGTCAGCTGGGGCATCATGCTCTATAACGTTCAGACCTCCTCCTCAATGCTAATTGGCGCGTGGTGGGAACTGTTTGCACCATGTGCGGCCCTTATCCTACTCGTTACTGGATTGGCATTACTTAACTTCGCTGTAGACGAAATTGCTAACCCGCAGCTGCGCTCGCACAAAGGGATGAAGCGTTGGAAAAAGCTAGCCCAGCAAGACAAAAAAGAACGTGAGCCCGAACTGGCACCACAAAATGCACTTTGGAGCGGAGATAAATAATCATGACAGCACCACTCATTTCTATCCGTAACTTATGTGTTGATTACATCACAGCAGCAGGCGACGTTCGCGCCTGTAACAATGTCAGCTTTGATATCGCTCCCGGTGAAGTCTTTGGCTTGGCGGGAGAATCTGGCTGCGGAAAATCAACGGTCGCGTTCTCGTTAATGCGACTGCACAAGCCGCCAGCATTTATTACCGGTGGAGAAGTCATCTTCAATGGCGAGAACATCCTCAATTACAGCGATGAAAGAATGCAAGCCTTTCGCTGGAGTGAGATGTCAATGGTGTTTCAGAGTGCGATGAATGCGCTGAACCCAGTGTTGCCAATGGAAGAGCAATTTTGCGACGTGATTATGCGTCATACCAACATAACGCGTGAGCAAGCACGTAAACGCGCAGAAGGGTTACTGGAGATAGTAGACATTCATCCAAGCCGTTTGACTGACTACCCTCACCAGTTTTCCGGTGGTATGCGCCAGCGCTTGGTCATCGCCATTGCCCTCGCACTGAACCCTAAAATGATCATTATGGACGAGCCAACCACTGCTTTGGATGTTGTGGTACAACGCGAGATTCTTCAGAAGATCTACGCTCTGAAAGAAGAATTTGGTTTCTCTATTTTGTTCATTACTCACGATCTCTCACTGATGGTCGAATTCTCAGATCGCATCGGCATTATGTACTCAGGTGAACTGATCGAAGTCGCGCCATCGAAGCAAATTTTAGAAAGCCCTTACCACCCGTACACCAAAGGGCTGGGAAGCTCTTTCCCTCCACTCACTGGTCCAAAAACGAAGTTGACTGGTATCCCTGGCAACCCACTCAACCTGCTGGAAATTCCACAAGGTTGTCGATTCCAAGCACGATGCGATCGTGTCCACGAAGCCTGTACCAAGATACCAACTCAACTGAAACAAATAGAGCCAAGTCGTTTCTCAAACTGCCACCTATATGGTGAGCCAATAGCCCAAGCCAAACTCTAGCGCTGGAAGACAAAAACATAACGATTACTGGAGACAATGATGAGCAAAGAATTTGGTGAACTACTGGTGGAAGGGAAAAATCTAGTTAAAGACTTCCCGATTAACAGTAATGCGCTGCAGCAACCCATGATGCGCGCGATAAACGACGTATCTTTTAAAATGTACAAAAGCCGTGGCCTGTCAGTGGTTGGTGAATCAGGCTCAGGCAAATCGACCACTGCCAAGATGATCGCAAAGATGTATGCCCCAACCAGCGGCACCATTGAATACAAAGGGCGTGATATCCAGACTATTGGCTCTCGCTCTGATCTGATGGCCTATCGCGAAGGCGTGCAAATGGTCTGGCAAGACCCATTTGGCTCTCTTAACCCAACCCATAATATTTTCCATCATATTGCCCGCCCATTACTAATCCATAAAAAGGTCAAACCGGGAAATAAGAAGGAATTGGAAGAGCGTGTCTACGACTTGCTGGAACAGGTGGGGCTTATTCCTCCTAAGGAAACCGCGCAAAAATTCCCGCATCAACTTTCCGGCGGCCAGCGCCAAAGGGTTAACCTAGCGCGTAATATTGCCGTGGGTGCTGAGGTCGTGCTCGCTGATGAGCCGACATCCATGTTGGATGTCTCGATTCGAGCTGGCGTTCTCAACCTGATGGAAGAGATGAAATTTGAAAAGCAGATGTCACTGCTCTACATCACTCATGACATCGCAACTGCGCGCTACATCGCTGAAGATCTTGCCGTAATGTACGTCGGTCATATGGTTGAGTGGGGAGACACAGAAGAGATCATTCACAACCCTCAACATCCATACACTCAATTACTGGTTTCCGCTGTACCGGATCCAAGCAAATCGATCCATGAGCAGCTAAAAGGCAATAAAGGTGAGATCCCTCTTTGGACACCTAATTCCTTGGGCTGCCCATTTGCTGGACGTTGTCAGCATGCAACTAACCAATGCCGTGAACAATTGCCAGAAGTCACTCAACTGTCAGATAACCACTTTGTGCGTTGTTACCTATATCAAAGCTAAAACCATATTGAAGCCAGCCTAAGAAGCTGGCTCATCAGATTCACTCGGAGATTTTAATGCTGCTACTTACTAACCACATTGGTTATGAATCCCTGGGACCAAAGCAGGCTATTCTGATGGCAGATGTACCCAACCTACCATCTTCCACTGCCCTTTTGGTGTGTGCAGATAGTCATCAGACTGTTGCGTCACTGGATGTCGAGACTGGCACTCGAGTCGCAAATTGGCACCAGGGATATTTTTTTCGCATTGATTTTTCCAACGTGAAAACACTAGGGCGCTACTACCTCAAATACAATAGTTGTCAGTCCTCTATTTTTTTGATCGATCAAGATCTGCTCTTCACCTCCACACTGTCCGACGTACTGCACTATTTTAAATCCCAACGCAGCAGCGGCATCTACGATCGTCAGGATCAAAACATACCGCTATTAAACAGCAGTCAAACTGTCGACGTCCGTGGCGGATGGTACGATGCCTCGGGTGATGTCAGCAAATATTTAAGTCACCTCTCTTATGCCAACTACTTGAACCCACAACAAATTCCAATGGTGGTGTGGAATATACTCAAAGGTCTTGAGCTATGTGAGACCAGCTCAA

>NODE_69_length_3114_cov_2.823510

CTCCAGTCGCGCCCGATTTTTCGACTGGTCCCTCAACAATCCTAATATTTTGCTGCTGTAAGTGAGTAATCACATCTTCCAGTGACCAGTGTGTGATTAAGCACAGATCACCTGAGCCGACCTGAGCATGATTACGGGGTTCCTGGCCTAAGAGCTGAAGATTGATTTTCTGATTACCGAACCTCAATGCTTTACGACCACCAGCAAAAGTTACCTCTTCCATTTTTAATACACGGCGATAAAACTCAACCGAAGCTTTAATATCCGAAACGGTAAGAACAATGTGATCAATATGGCTAATCATGAAACCCTCCTTATCTAGTTGTACCCTAACTCAATACAATGGCTGTGCTATACCAATGAACGGTATACACAAACCTCACGTTTTTCGCCGTGTACCTCAACCGTCCGGTCTTCCTGATACTCAATCCCCAGTTTTTCAATTACTGACTTTGAACGCTGGTTATGCACCAAATGCTGAACTTCAATCTCGACCAGATTCAGGTGTTGTTTGGCATAGTCAATTAAGGCTTGAGATGCTTCAAACGCGATACCTTTCCCCCAATAGGGTGTCCCTAACCAATAACCAAGAATGCCAACACCTTCTTTCAGTGTTGGAAGGCTGACGGCGCCGATGATGTCGTCACAACCTTTAATTGTGATGGCAAAAACAACCGCCGTTTTCTCAGTAAAATGATGAATATGTGTCTTGATCCACTCCACAGCCATATTCGATTCGTACGGGTGAGGGATATTCGCCGTCATCTCCGCGATAACCTTTTCTCCCGCTAAATGGGCCACTCTCTCGCTGTCTGATAAACGAAAGGGTCTCAGGACTAATCTGTTTGTTTCTATATTGGGTTGCATTGCATACCTTGGTTTACAGTGTAAAAGTGACAAGCTCTGAAGCCTTAATGGCAAAGCTCTGCCTCAGTATTGATAGACATTGTGAGTTCTGAAATAGACTCATCATCAACTCGATAATAATCTGTCATTTCCCCAACAATTCGAAATCCATGTTTGAGATAAAGATTTTTAGCGGCAAGATTGACTGATAACACATTTAAGTCGAGCCACACGATCTGCTCATTGCGTTGACAAAACTCGATCACGGTTTGGATTAGCCGACTGCCTAATCCCATTTTCCGGCACTCCCGCTCTACGCCCATCCCTAGCCAAACGCGATGAGAGCAATAATCTTCCATATGATGTCGAAGGTCAATGTGGCCACAAATCTTACCCTGTACGTCTTTCGCTAACCAGAGTTGTCTCCATGCAACGTCAGAAAACTCAACGCTCACCCCGTCTATAAACTTTTGCTGTGTTTGAGTCGGAATGGCCCTGTTTGTACGTGATAGAGGCTGAAAAAGTGGTGTGCCATCGACACCATTTTCCGCAAGTTGATTTCCAAGGTAAACAAAAAACTGAGGTAAATCACTCTGCTCAGCTTGAACAATAGATATCTGCATCTGAGTATTCTTCAAAAGTAAATCGATTACAGCTTCAGTTTGAAGCCCTCATGGCTAGGTATAAAGCCCAGTTTTTCGTAAAAACGCAGCGCATCGGGTCTCTGTTTATCGCTAGTCAGTTGCACCATAGAGCAACCTCTTTGCTTCGCAAGTTCGATGGCATGCTGAAACAAACACTCACCTAGTCCCTGACCGCGAAAGTTTTCATCCACTCGTACCCCTTCAATCAAGCAACGCCAGCTTCCCTGATGCGTTAAGTACGGAATGTATGTCAGTTGTAGCATTCCAACCACCCGTTGCTCAAACACGGCAACGATCAGTTCATTGTTTGGGTCTCGGTTAATCTCAGCGAAAGCCGTCTGGTATTTATCGAATTGCGTCTTATCTTCACGCTGCTGACCTAATGGATCGTTGGCCAGCATACCAACGAGAGCGCCTAAATCATCCTCAGTCGCCGATCTGAATATCAACTCCATGTGTTTCCTTATTTTTCTATTTCTTCAAGCAATTGTTTCGCAATGCGTTCTATCTTCTCCAACGAGTGGTCACTCTTGTCTAACAAGAGCAAATCCGCCAATTCTGGATTCTCCCATTGTTTACCTTTAATAAAACAGCGCTCCGACAACTTATCCAACTTAAGGTCTTTCAACTCGAAGCTCAGCTCAGTCCAGAGCAATGACAGCTCTCCTTCCGCATCGATATCACGAATAGGGTTCTGCTTTTGCTTTCTGATGTAAATGGCGGTTTTTCGACTCGAAATCACTACCTTACGCAGCGCAGCGGTCGATTCCATCTTTCGGGATTGCTTCGCGCGCTTCAGGTTAGACACCCAGGTTCCGGCATGCTTCACTAACTCCCAAAGCGACACACTGGTGACCAGTTCGATCAAATCATCTTCCTCTCAACATGGATTAACTGACACGCTCAAGTTAAGCGACATTATCAGACACTATGCAACAACACGAGTTTGTCGAATTAAAAACGTGCCTCAGGTAGGAAGTCTTAACGATAAAAATGGGCACCCTAAGGTGCCCGACAGGAGCAGTACGTTCTTGGGGGAAGAACTGAACTAATTATTTGCGAGGAGTGAGCATGCCTTGTGTGACAATAAAGTCGATGATGGTATTCAGACCATGGCTCTCTTTGAGGTTAGTGAATACATAAGGCTTGGTTGGGCGCATGCGTTGCGTATCTTGCTCCATCACTTCCAATGAAGCACCGACATAAGGTGCGAGGTCGATTTTATTGATCACCAATAAATCCGAACGCGTAATGCCCGGGCCACCTTTACGTGGAATTTTCTCGCCTTCGGCAACATCAATCACGTAGATAGTTAAGTCTGCCAATTCAGGGCTAAACGTCGCACTGAGGTTATCGCCACCACTTTCGACAAACACGACATCCAGATTCTTGTGTCGTTTTGCCAGCTCTTCTACCGCTGCCAGATTCATCGAAGCGTCTTCCCTAATTGCCGTATGCGGGCAGCCTCCGGTCTCAACACCAATAATTCGATCCGGTTCGAGTGCTTCCGCGCGGGTTAGAATCTTAGCGTCTTCCTGTGTGTAGATATCATTGGTAACCACGGCGATATTAAGCTTGTCGCGAATCGCTTTACATAGCACTTCCAATAGCGCCGTTTTACCCG

>NODE_70_length_3113_cov_2.326746

TCCTTAAGCATAATAAACTCGTTCGGCTCAGACAATGATTCAGGCCTGAAAAGGTGACGCCTGGACAGCAATACAGAGCAATAGTTAAAAGGCACAGAGCAAAGCATGGCGCGTTTCTTCCAACCGAAAAAGAAAACTCAACTCAACACTAAGCATCAATCGTTGAAAATCGAAAAACTCGATCATCACGGTGCAGGCATAGCCTATCAAAATAAGAAGCCAATTTTTATTGAAGGGGCACTGCCGGGAGAGCAAGTGTTAGCTCAGTTGACCGAAAGTAAGAGTAAGTTCTCACGAGCGACGCTGATTAAGGTGCAAACGCCGAGTGAACAACGAGTTGAGCCTTTCTGCCCACATTATCAGCAATGTGGGGGCTGTAATATGCAGCACCTGAGTAGTGAGCACCAGCAGATGCACAAACAGCAAACGCTGAGCCAATTGATGAGCAAGTTTGCTGGGCAGAGCCTCAACTTAGAGGCCCCGATAGTGGGCGAATCGGAAGGCTACCGTCGCCGCGCTCGCATCAGTGTCATGGTGGATAAAAAAACGCGTCAACTGAAATTTGGTTTCCGTAAAAAGCAGAGTAAAGACATCGTCACTCTGACTCACTGCCCTGTTTTGGCGCCGGAATTGGATGAGTTATTGCCTGATTTATATGCGCTGCTATCACGCTTTTCTCAACAAGAGAACATTGGCCATGTCGAGTTAGTGAAAGCAGATAACACGCGCATTCTGGTTTTGCGCCACCTCAAGCCACTGAACAATAAAGATCATGATGCACTGCTTGAATTTGCCAAGCTCAATCAGATTACGCTTTATCTGATGCCAAACAATGAACAGTTGATGCTTGAAACAGGTGAAGCGGGTGTTTATCAGGAAACTGGCGTCAACATCCCGTTTGAGCCAAATAATTTCATTCAAGTTAATCAACAAGTGAATGCGCAGATGGTTACTCAGGCGTTGGATTGGCTGGCGTTATCTGAGCAAGATCGAGTGTTAGATTTGTTTTGTGGGTTAGGGAACTTCAGCCTCCCTATCGCTAAGCAGGTAAAAAGCGTTATCGGTGTTGAAGGGGTCGATGAGATGGTGAGTAAAGCTACTCATAATGCTCAAGTTAACCAGATTGATAATGCTCAGTTTTATCAGGCAAATTTGGAACAAGATATGTCTGGTCAGCTCTGGGCGAGAGAAAAGTTTGATAAAATACTGCTCGATCCAGCACGAGCTGGCGCTAGTGGAATTGTCGAGCAAGTTTCTGCGCTAGGTGCGACAAGAGTCGTTTATGTTTCTTGCAATCCTGCTACTCTAGCCCGAGATTCTCAGAGCTTGCTAAGTCAGGGATTCGAGTTAAAGAAACTGGGTATGTTAGATATGTTCCCCCATACCAGTCACTTAGAATCAATGGCGCTTTTTGTGAAGGATTAATGCGAACTTAGCCCGTGGTGAGCAGTCTCAACACGGGGTAGGTCGAAGATAAAAAGTATTACAGGACGAAGACGATGGTTGCGGTAAGAAGCGCACATTTGAATCAAGACGAACAGTTTGAACTAGATAATTGGATTGCGAGCCTAGAGCAAGACAATAAAGTGTCGAAACGCCTTAAAGAGGTATATCGACATTGTGAAACCATTCTACAAGACAACGATCAAGGCCCACTGCTGTTATGGCGTGGGCGTGAAATGATCGAAATTCTCATTACTCTCTCCATGGACAAAGCCACCTTAGTGGCTGCTTTGTTGTTTCCAGTGGTGTCCAGTGGGGCTTATGAACGTGAAGCACTGGAAGAGGATTTCGGCAAAGAAACGATCAAATTGATTGATGGCGTTGAGGAAATGGCGGCACTGGGACAGCTCAATGTCACTATGGAAGGCAGCGCTGCCTCGGCTCAAGTAGATAATGTTCGCCGTATGCTATTAGCGATGGTCGATGACTTCCGCTGCGTGGTCATCAAGCTTGCTGAACGCATCTGTAATCTGATCGAGGTAAAGAAAGCCCCGGACGAAGTGCGCCAAGCTGCGGCGAAAGAGTGTTCAAACATCTACGCGCCTCTGGCAAACCGTCTTGGCATTGGTCAGTTGAAATGGGAAATTGAAGATTACGCTTTCCGTTATCAACAGCCTGATACCTATAAACAGATCGCGAAACAGCTTTCTGAGCGTCGCATCGTTCGTGAGCAGTACATTACGGATTTTGTGGATGACCTCTCGAATGAAATTACCTCTTCAGGCATCAATGCAGAAGTAAGTGGGCGTCCTAAGCACATTTACAGTATCTGGCGCAAAATGCAGAAGAAGAGTCTGGCATTTGACGAACTGTTTGATGTTCGCGCTGTTCGTATTATCGCCGACAAACTTCAGGATTGTTATGCAGCACTAGGCGCTGTTCATACCAAATACAAACACCTACCAAGTGAATTTGATGATTACGTTGCGAACCCAAAACCGAACGGGTATCAGTCAATCCACACTGTGATTCTTGGCCCTGAAGGGAAAACCATCGAGATCCAGATTCGTACTAAAGACATGCACGAAGACTCGGAGCTTGGCGTTGCAGCGCACTGGAAATACAAAGAAGGTGGCGGTGGCCGAAGTGGCTACGACGAGAAAATTACTTGGCTGCGTAAACTGCTTGATTGGCAGGAAGAAATGTCAGATTCCGGCGAAATGCTTGATGAAGTACGTAGTCAGGTATTTGATGATCGCGTTTACGCGTTTACGCCTCGTGGCGATGTGGTCGACCTGCCTATGGGCGCGACGCCACTTGATTTTGCTTACCACATTCACTCAGAAGTCGGGCACCGTTGTATCGGCGCCAAAGTTGCAGGGCGTATAGTACCGTTTACCCATAAACTGACGATGGGCGATCAGGTCGAAATCATCACGGCTAAAGAGCCGAATCCATCTCGTGACTGGCTGAATCCATCAATGGGATTTGTCACATCTGGTCGAGCGCGCGCTAAGATCAATGCTTGGTTCCGTAAACAATCACGTGAAAAGAACCTAGAAGCGGGCCGAGAGATTCTTGAAGCCGAGCTGGCCAAAATCAACGCGACACTGAAAGACGCCGAGCAGTATGCGTTGAAGCGCTTTAACGTCAACAGTGCCGACGAATTG

>NODE_71_length_3102_cov_2.721653

GTCAGTATACCAGCGCTGAAACCATACAAGATGAGAGCTAGGCCCGTAAGCTTGAATGGCAGTGAGGCGCCATGGCCTTCTTTTTCCAGTACGTTGGCGTAGCTCATCATGGCAAAACCGGATAAAGCACCGGCGCCTAGACCGAATACCCAGCGTATTTGGTTCGCAGTATTGTTGAGATAAACAGCAAATTCGTTGTGACCGTAGCGATAAAACAGGCTGAACACAAACAGAGTTACGACCGCAAGCGTGCCCCCTTTGAGTACGCGAGTATAGCGCCAGCGGGTAAGATCGAGCATTTTCCAAGCAAACGCCCCCAATGCGATGTAGGAAACCCAAAGTTTAAACACCATGAAGATGTGTAAATCGCGGGTGAGGGGAAAATCGTGCTCATAAAGGACTAGGTACAGTTCAGACCATTCGTGAAAACCATGAATTAGACCAAACAACGCCAGCGTGGGGAGTGCGGAGGCAATGGCAATACGGCTTTTTTTAAGATTGCGAAACAGGATCGCAAAGAAGATGGCAAAAAACGCCAACCCGTACAGCAGGTAGATCTTGAAGATGAGTAGCATGTCCATCGGTTAATCTTAATTGAGCATGAACTCTTCATCAATGCTTAATCTAAGCGTTTTATAAATAGCATTGAGTATATATTTTATTACACTTTGAATTAGCCTAAGAATATTCTGTGAAGTTTTTTTATCAAATTATATTTGGTTTCTATAATTGTTATTCACGACTAGTTGGTTGACATCGTTTTTTAATAAATCTGTCACATTGTATTTCTAATCTCCGCTTTGTTGATTGGTCAGCATCGATCGACTGTTGAAGTTATTTCAATATCTTTAGTTTTACTTGAACACTAAAACTAATACGGGCTAATTAGATTTCACTTTAATTAGCCCTACCATTTGCTTATTTATATCGGAGATATATTGTGAAATTCATCAAATCAGCTCTAGTGACTTCAGTACTGGCGGCAGCATCCTTTGGCGTAGCTGCCAACACGACGATTAACGGCGCTGGTGCGACATTCCCTCACCCTATCTACGCTAAGTGGGCTGAGCAGTACCAAAAGGAAACGGGCGTTCAGATCAACTATCAGGCGATTGGTTCAGGCGGCGGTATCCGTCAGATCACAGCAAAAACCGTCGATTTTGGTGCGACGGATGCACCTCTGACTATCGAAGAGCTGAAAAAAGAAGGAATGATTCAATTTCCAATGGTAATGGGGGCTATCGTTCCTGTTGTTAACATCCCTGGCATCGATGCCGGTGAAGTTAAGCTAACAGGTAAAGTGCTGGCAGACATCTACCTAGGTAACATCAAGAACTGGAATGACCCAGCGATTGCAGCCATCAATAAAGGCGTTGAGCTACCAAGCCAACCTATCTATGTGGTTCACCGTTCAGACGGTTCAGGCACAACCTTCAACTTTACTGAGTACCTTGACCAAGTTAGCCCTGAGTGGCACGAGCAAATTGGAGTTGGTAAAGACATTACATGGCCAAGTAAAGCGACCACTATCGGCGGCAACGGTAATGCAGGGGTTGCGAACTTCGTAAACCGTACGCGTGGTGCAATTGGTTACGTTGAGTATGCGTTTGCCAAGCAAAACAATCTTGCTTACACCCAGATGCAAGCGCACGACGGTGATTTCCTGATGCCAACGATGGAAAACTTCCAATCAGCGGCTGCAAACGCGGATTGGGATAATGCTCCGGGCTACCACTTGCTATTGAACAACCAGCCGGGTGCACACTCATGGCCAATGACTGCTGCGACTTTCATCCTAATGCACAAAAATCAGGCTGATGCAGGCAAAGCGAAAGAAATCGTTAAGTTCTTTGAATGGAGCTACACACAAGGTAAAGCTGCGGAAGAACTCGATTACATCCCGATGCCAACCAAGGTTGTGAACATGGTGAACGACACTTGGAAGCAAGGTCTGAAATCTGACGGTAAAACGATCATTCACTAATGAACCTATGCCTCACCAGCTCGGCTGGTGGGGCTGATTAAATTGTTATGACAGCATTAACGTTAAAAAATAAAGTCGATGGCGACTCAATTTTCAGCAAACTGAGTTTTGCCAGCGCTTTATTGATTTTTATTACTCTGACGGGGATTGTTCTTTCTTTAATTGAAGGTGGTTGGCCTGCATTTAAAGAATTTGGTCCGGGATTTGTTTTCAGAGATATTTGGGACCCTATTGGGGGTCAATTCGGTGCAGCGTCGGCGATTTACGGCACGCTGGTCACTTCATTCATCGCTATTGTTATCGCAACGCCTATTGCACTAGGTACTGCAATCTTTCTTGCCGAGCTGGCACCAAAATGGGTAAGCCAACCAGTCAGCAAAGCTATTGAACTACTCGCAGCTGTACCAAGTATTATCTACGGTATGTGGGGCTTGTTCGCGTTTGTTCCTTGGTTCTCTGAGGGTTTCCAGCTTTGGGCTGGCACGCATCTGGTGGACATTCCATTGATTGGTCAATGGTTTAATGGACCACCGATTGGTATTGGTCTTTTAGCGGCGGGGATCATTCTGTCGTTTATGATCTTACCAATCATGACGTCTCTGACGCGAGATGCATTGAGATCGATTCCGGATGTTCTGCGCGAAGCCGCCTACGGAACGGGTGCAACACCGTTCGAGGTGATTACTAAAGTGCTTATCCCGAAAGTGAAGAATGCAACGGTCAGTGCAGGCATTCTTGGCTTAGGCCGAGCGCTGGGTGAGACCATGGCGGTCGCGTTTGTCATTGGTGGTGCGAGCCGTATTGAGTTCTCGTTCTTCATGCCAGCAAGTTCAATCTCATCAACGATTGCTCAGCAATTCAATGAAGCAACAGACCCGATTCATATTGCATCATTGATCGGTCTTGGTGTCGTTCTGTTCATCATTACCTTTTTTGTTATGGGCTTTGCTCGTCGTCTTCTGAGGAACTCATAATGAATTTGAGAAAACTCAAGAATCAACTGTTTCTGGTGTTCTGTGCATTGTCGGCAGCGTTTGGCGTCTTCGTTTTAGCCAGCATTCTTTACACACTCATTGGAGAAGGTGTTAAAGGGCTAAACATCATGACGTTTACGGAGC

>NODE_72_length_3094_cov_2.201193

TTTCTCAAGCGAACGGTTGTGGTGGAAGACATCAATCACCACCCTAACTGGGCGCCTTTTCTCTCTATTACTGAAAAAGCTAACCTTCGCGCTTGCTGGTCGGTACCTATATTAGCGAATGATCAGACTGTGATGGGTACATTTGCGATATACAGCTCGCAACCTTCTAAGCCAAGCGAAGAAGAGCTGGAAGTTCTAGAAATGTTGGCTTCTCTTTACGCTGTCGCGTGGGAAAAATATCAGCTTGAACAGCAGCTTCACTATCACGCTAGTTATGATTGCTTAACAGGTTGTCTGAATCGACGAGCGTTGCTTCAGCGAGCTTCTGATTGTCTCAACACCAATTTAACTTACGTCGCATGCTTCTTTGCCGATATTGATAAGTTCAAGCAGATCAATGACAAGAATGGACATGAAGCAGGGGATGTTGTGTTAACAGCAGTTGGCCACGCCTTCAAACACTGTTTTTCAGAGTCAGGCATTAGTGGACGCTATGGTGGCGATGAGTTTGTCGCGTTTGCGTTTTCAGATGATGAAGAAGCTCTCCGGACCCTGCATACCGAGCTCAGAGAAAGTTTGGCAGAGATTACTCCACTGCATACGATTGAAGTCAATGTGAGTGTCGGGTTGTCTATTGTGAATATTGAACGCCTAGACTCTATTGAGTGCTTGATTAAACAAGCGGATAAGCAGATGTATCAGGTTAAGCATCACCACTGAGAGTGACTTAGATCATAGTATCTGGAGTGCCTCAGAATATAAGCTCTCAGTAGAAGCATCGATTAAGAGAATTCAATATGAACCCGTGTCCAGTATGTCAGAAAGAGCTAGAGTGGCAAGGTCATTACCACTGCAATGACTGTGCAAGTGATTACATCAAGGTTGGCTACTGCCCTGAGTGTGAAGACAAGCTAGAAAAATTACAGGCTTGTGGTTCCGCCAGCTATTTTTGTCACACTTGTAACGAACTTAAGTCGAAGTCCAGAGTTGGCTTTCAGTTCATTAAACAGGCTTAAACGACGGTAGAATGTATCTCTCCGTCAGATAAACGGGTGACCAGAGTATCACCCGTTTTTGTCTCGGCCGCTTTGGTCAGCACTTTACCTGATGCATCTTGAGTAATGGAGTAGCCACGTTTTAACGTCGCGAGCGGGCTGACAGTATCCAGTTTTTCAGTAGCCAGTGCGAGTTGGTGGCGAGAGTTAAGTAGCTTTCTGTCCATCGCGTCCATCAGAGTTTGCTCAAGTCTTTGTAACGACAGCTTCTGTTCATTAAGTCGCGCCACTGGAGAGTGGAGTTGTAGCTTATATTGCTGGCGTTCTACGTTCTGACTGCGGGTAGTCAGATAGCGTTCCATTGAGCGTTGCAGACGAACAGACAACTCGTCGAGCTGTTGGCTCTGTCTCTGAAGTTGATGAGACGGGTGTTGACGTTCCAGTCGGTGCTGTAGCGCCGACGTGTTTTGTTTATGCTTGGTCAGATAATGACGAATAGCACTATGAAGTTTGTGCTGACGAGTATTGATGGCTTGCGCTTTATGGCTGTTATCTCGGCTGACTAATTCCGCTGCGGCTGACGGCGTAGGTGCACGCATATCGGCAACAAAGTCGGCGATAGTCACATCAATTTCATGTCCAACAGCACTGATAATAGGTATCTGACTGGCTGCAATTGTACGTGCAACAACTTCGTTGTTGAAACACCAAAGATCTTCCAAAGAGCCGCCACCACGGCCAACAATCAACACGTCACATTCGTTGCGACTATTCGCACAACCTATCGCCTGTGCGATCTGAATGGCCGCATCTTCTCCTTGAACCAATGTAGGGTAAATCACGACAGGTAACGAAGGATCACGCCTCTTAAGGACATCAAGAATATCGAAGAGGGCAGCGCCGGTTTTGGAGGTCACTACGCCAACGCGTTTTGGGTGCTCCGGCAATGTTTGTTTACTGGTTTGGGCAAACAAACCTTCACCGGCCAGCTTCATTTTCAGTTCTTCAAACTGCTGCTGAAGTCGGCCATCACCCTCAGGCTGCATACTTTCGATAATAAGTTGGTAGTCACCACGAGGCTCATACAATGACAGACGAGCTTTGACTAAAACCTGATTGCCATTCTGAGGTTTAAAAGTCACTCGGCGATTGTTGCCACGGAACATGGCGCACTTAACCTGAGCACGGGAGTCTTTGAGGGTAAAATACCAGTGGCCTGAAACGGGCGCAGAGAAGTTTGAGATTTCGCCGACCAACCACACAATACCCATTTCATTTTCTAGTAATAGGCGAACTTCAGAATTAAGACGAGATACAGTGAAGATGTTTTGATTAGTCAGAGATGACACAGCTAATCTCATAGGCGTGAGTATGGAAATTAGCGGCAATATAATACATAGCAAGGGGGTAATTGCAAGAAAAAAATTAAAAAAACTTGTGGTCAAGCGATTGCGTTAGCCGTATAATCCGTCCGCAATATCTAATCCAAATGCAGCCTAGCTTCTCCGCTAACTGCCCTCATTATGCGAAACGATGAGACCGGATTGTTCTTTTACTCCTCTAATTGTGAGATATTGCAAATGCTAAGAATTGCCAAAGAAGCGCTGACATTCGACGATGTTCTACTAGTGCCAGCACACTCCACCGTTCTCCCAAACACAGCTGATCTTCGCACTCAGCTGACGAAAAATATTACCCTTAACATTCCGATGATTTCTGCCTCTATGGATACCGTGACGGAAGCTCGTCTGGCTATCGCTCTAGCGCAGGAAGGTGGCATTGGCTTTATCCACAAAAACATGTCGATTGAGCAGCAAGCTGAAATGGTTCATCAGGTTAAGATTTACGAAGCTGGCGTGGTTTCACATCCGGTTACTGTAAACCCTGATGCAACAATTGCAGACGTTGTGGCTCTAACAGAAAAGCATGGTTTTGCAGGTTTCCCTGTCGTAACTGACAACAATGAGTTAGTTGGTATCATCACTGGCCGTGACGTTCGTTTCGTAACTGACCTTTCTAAGAAAGTTGAAGTCGTTATGACACCTAAGGAGCGTCTGGCGGCAGTAAAAGAAGGTGCAACTCGTGAAGAAGTGCAAGAG

>NODE_73_length_3089_cov_2.259296

CCGGACCAAGAATGATCGCATCCGCTTGTTCTATCGCAAGTAGAACAAGCGGATGCGATCATTCTTGGTCCGGGCAGTTTTCTAACCAGTATTATGCCACCATTGCTGTTATCTGAAATTGGCCGAGCAATCGAAAACAACCAAAAGGCTCAGCTTGTCTTCGTTGAAAACCTATCTCCTGAATACGGCCCTGCGGGTAAGATGACTCTGAAAGAAAAACTGGAATGGTGCGAGCGTGCCTGCCGCGGTAGAAAAATTGATGTGGTTTTGGGTGAAGAACCTCACCCTGAATTAAGCGATTGGAATTGCATAACAACACCTCTCGCTTCACCAAATCGAGACTGGCGCCACGACAGAAGCAAATTGCAGTCTGCCGTTGAGTCGTTATTGTGCGAGTAACGCTTTATAGCGCTGTTGGGTTTCACTCAGCAGCGCAACCATCTGTTGCTGTGTCGCTGAGATATCGAGCGTCTGACCTGATTTAGCTTTCGCGTCCACTTCCACCGCATACGCACATAGAGATTCAGCCCCAAAACTTGCAGCACTGCTTTTCAGAGCATGACTAATTTCTTTGAGATAAGCATCTTTATCCAGTCCTTCGTCGGTACTGAGCTTTTCGATGTACTGCGCTAGTTCCCCAAGAAAGATATCAAGCAAAATCGGTACATTATCAGCACCAATTTCACTTGCCAGTCGGTCTATTTTAGTTTGATTTAACACTTCCATGATCGATTACTGTGTTTCCTTCGTGTTCCAGTTTTGCAGCTTGCGATAAAGCGTTGATGGACTGACATCTAAATAGCCCGCGGCTTTAGGAATATTCCCTTCACAGGCCTGAATCGCTTGCTCAATGGCTTTCTTCTCTGTTAACCACAGAGGGAAAATGTCATGCACAGAAAAACTGTCTTTGTCCTGCAAGTCTACCCTGATCTGATTTTCGACTGGCTGATTCAATGGAGGCGGCAACATATCCATGGAGATGTTTTCACCGTTGTTAAGAACCACGACGTTTCTCAGTACGTTTTGCAATTGACGAACATTACCCGGCCACTCGTATTGAGCGAAGCGGCTCACAACTTCTGGTGCCAGCCGGACAAAGTCTTTTCCTTCCTCTTTAGACATAAAGCCTAGCAGAGAATAAGCAATTTCTATGACATCATCGCCACGCTCCCTCAAAGGAGGTAGGTGCAAAGGAATTACGTATAAACGATAATATAAGTCTTCACGGAAGCGACCTTCCTGCACCTCTTTCCAAGGGTCTCGGTTGGTTGCACAGACGAAACGCACGTCGACGCTTTTCATTTTCGAAGAGCCGACTTTCTGGAAGGTACCCGTCTGAATAAAACGTAGTAGCTTAGTTTGCAGATCCAGATCCATCTCACACAGTTCGTCAAGGAACAGCGTGCCTCCATCAGCCAACTCAGCCGCGCCTTGTCGATCAGTGGCAGCGCCGGTAAACGCGCCTTTCACATGACCAAACAATTCACTTTCAATCAGATCTTTTGGAATAGCCGCACAGTTGATCGCGATGAAGGGCTTGTCACCACGCTTACTTGCCGCATGAATGGCCTCTGCACACACCTCTTTACCCGTACCACTTTCACCAGTGATGAAAATACTGGCTTTACTGGATGCGGCGGAGTCGATGGTACGATACACAGCCTGCATAGTGTGGCTACTACCGATAAAGCCTTGATAATTCTGGTTACCCGGATTATCAGCTTCATTTTTCAACTTGGTCGCTTTGCGAATAGCGTTGTTAACCGTGACACGGAGTCGGTCCGCTTCACACGGCTTGATAAGGAAATCCTGAGCACCATGGCGCATCGCTTCTACAGCGGTATCAATCGAGCCGTGGGCAGTCATGAATATCACTGGAACATCAGGCATTTTCTGTTTCACTGCATGCAAAACATCCATACCTGTCATGTCGGGAAGACGAAGGTCCAATAGGATTAAATCAGGAGTGCGAAAGTTGAGGCTTTCAATGGCATCACGCCCGGTACCAACGATGTTGATATCGATACCCAAAGGGGTGAGATAAGAACGGTACAACGCCGCGACCGACGCAGTATCTTCAACCATCAAAAGATATTTGGACTTTTGTGTTTGCGTATTGTGTTGCATAACCTAGCCGAATTTTTCTTCGCAATTTGTATAAATGATTGCATTGCGCTTTGCATTTTGCAAATTTAATGCTTAAAAAACGAGAAACGCCCTAAAATTGGCGTAAAAAAATTGGCACGTTAAATGCTGTTATAACATTGACCCTTATGGGTCACCTAGCCAACTGACGTTGTTAGTGAACTTAGTATTCACAAATTACAGCCAATAGAATTTATTTCTACTGGCTATTTTTTTATCTGTCAGTCGGAGGCCATAACACGAAATCAGCTATTCACAATGAACTGCTGACGCAATTTTTCAATTTGATCTCGTTTCTCAGCCGCCGCTTCAAACTCCAGATCTTGAGCATGCTGATACATCTGTGCTTCCAACTTGCTAATCTCTTTTTCAAGCTGTTGCGGTGTCATCACATTGTAAGACTGGGACGGCTCTGCGACCTTGGACAACGGAACCTGCTTATTAGTTCGTTGTTTTTTGCTCTTAGCAATATCGCCAAGTTCCATAATATCTTTAATATTACGTTTCAGTGCCTGCGGCTCGATGCCCATCTTATCATTATAGGCTTGTTGTTTCTCTCTACGACGGTTGGTTTCTCCCATCGCCTTGTGCATAGATTTCGTTACACTATCGGCATAAAGAATAGCTTTACCGCTAATATTACGTGCCGCACGACCAATAGTCTGAATCAGCGAACGCTCGGAACGTAGGAAACCTTCTTTGTCAGCGTCCAGAATAGCGACCAACGACACTTCCGGCATATCCAAACCTTCTCGCAGCAAGTTGATCCCGACCAAAACGTCAAATTCACCCAGTCTCAGATCTCGAATAATTTCCACACGTTCGACCGTGTCGATATCAGAGTGGAGATAACGAACACGTACGTCATGTTCCGTCAGGTACTCAGTTAAATCTTCTGCCATACGTTTTGTTAGCGTGGTGACCAACACACGTTCGTCCTTAGC

>NODE_74_length_3080_cov_2.034965

GTTGGATACGTTTACGCTGCAAGCTCAATCAATTCCGACATTGACAGATAAGGTTTACCAATTAGAAGGCATTAAAGTTAAGTTAAACGAAAAGTTGGAGTTGGATAAAAGCCTGAAAGATTCTGTCACCAAGAAACAAGAGTTCAACGCGACCTTAATCAAATATCAGGCGCATAAAGAAAAGCTATTACAAGAAGCATCTAAAGCTCAACAAGAACTCGAGAATGCGAAGCTAGCCATTGCAGGTAAAGCAACGGTAGAGTCTGAACTTGCACGCTTACAGCGTCTTACTGCCGATCTCAATAAACTTCAGTTGTTGTCTAAGGAATTGGCTACTTTAGAGCAAGGTCTTCAGCCCATTCAAGCTCGTGTACAACAGTTGAAGGTCGATTGGCAAGAGCATAGCAAGCAAGCGGATGAATATGAAATGCGCTGGCATAATGCTCAGGCAGCGGTTCTTGCTAGTAAGCTTCAACAAGGTATGCCTTGTCCGGTGTGCGGCAGTGATGAGCACCCGAACCCTGCTTCGTTTGATGGTCAAGAAGTGACTAAAGAGCAAGTGGAGTTTTATCGTAACCAAGAGCGTCAGTCCCTGAACTCTTTAAACGAACAAACTCGCATTGAGGAACAACAGAATGCCCTTATCTCTCGTCAAAAAACTCAAATTGAAGCGTTTCAGGAAGAGTTGGGGGAACATAGAGACCTCTCTTTAGATCTCGTACAGAACCAGTACTCTGAATGTCAGGCTAAGTTAGCTGGATTGGTTTCAATTAACCTTGAGCAGATGGAGTCGGAGGTTGCAACGCTCAATCAACGCTGTGAAAAAGGTGACAATGAAATTAATCGCCTGAGAGAGGAGATGTCTGCTCATGATGCAACAATTCAGGCGCTTCAACAGCGCTTAGACATGCTCGTTCAGTCCATTGGTGAACAACATCAATCTGTGGAATCTGTCGACAATGAATATAAGCAAACCAAAGCGTCAATCGATACTTTGCAAAAGCAACTAGAACACGCCAATGAACAGCAACAGCGACTTGCTAAGCATCATGCTGAGCTTGAAGGGCAAAGACAGAGCAACACACAGCTTTTAGCGGAAGCAAAACGTAACTTAGATAAAGCAGAAACGAAATGGCATGTCTCAATTGAAGAAGCACAGTTTGCTGATGAGCAGGCTTACCTCAACAGTAAGGCGACGGATGAACAAATAGAGCGTTGGAAAGCAGCGGTCGAAGCTCACGCGAAAACCGAAGTTAAACTGAAACAGACGTTAGAGGATTTGAGACATACGCTTAAGGACCAGCAAGTGCCAGATTTGGTTGAGCTAGGAAAACTTGTTGAATTGAGCAGCCAAGAGTACGTTGAAGCGAGAAATGCACTAGATACTACTCGTTCGAAGTTTGAACGATTTGAGAAAGTTCGTCGTGATATTGTCTTACTGCACGAAAAGAATGCGAAGTTGGAAGCTGAGTACAGGGTGTATGGAACCCTGTATGACGTCGCTAGTGGTAAGACTGGAAGTCGGGTTAGCTTGCATCGATTTGTCCTTGGCGTATTGCTGGATGATGTTCTAATCCAAGCATCTCAGAGGCTCAGCCTGATGAGTAAGGGGCGCTACATTCTTGCGCGTAAAACCGAAGGCTTCAAAGGTGCGGCAGGACGAGGCTTGGATCTGATGGTAGAAGATAGTTACACAGGCAAAAATAGGGACGTTGCAACCTTATCAGGGGGGGAATCTTTCATGGCAGCGCTGTCTTTAGCTCTTGGTTTATCTGATGTTGTTCAATCGTACAGCGGCGGTATCAGGTTGGATACGCTGTTTATCGATGAAGGTTTTGGCAGCTTAGATCCAGAGTCATTAGATCTCGCCATCCAGACGCTAATTGACCTGCAGCAAAGTGGTAGAACCATCGGTTTAATTTCCCATGTTAGCGAGCTTAAAGAACAGATGTCATTGCGTATTGATGTGAAGTCCAGCCGAGTGGGAAGCGAATTGAAAGTCAATGTCGCATAAAGTAGTTAATAGGGCCGATATTCTGGTCGGAAGTGGCTAAATGTGCTAGTTATTAGTTAAAAGCTATAAAAGTCGAGCGGCCTTTAAAACAGCTCTTTTAGTTGGCATCAGGATCATTCATGACAGCAGTTCTGGAATCAGTTCGCAAAGTATACCAGTATGCAGAACCAAACCTCACGCTCGTAGGTTGGATGGGGTTGCTTGGCTTTCCAACTTACTACTACGTCTGGTCCTACTTATTCCCTCAGCCATACGAAAGCCTGACGCTCAGGCTCTTCTGTTCATTCCTATTCGCAATAATTGCTTTTCGGCATGCGCTCCCTAAATACATACAGCGTTATATGCCGCAATATTACCTCATCTCGATAGCGATCTGCCTGCCGTACTTCTTTAGCTATATGATGTTTATGAACGAATGGTCGACGATATGGGCGATGTCGTTTATGGCTTCAGTTTTTCTCCATGTTCTGCTTGTTCATCAAACACGAATTATGCTTCTGCAAGCCGCGATTTCGTTGTTGTTTGCTTTTATTACCGTCTACGGATTTAACTTTAGTTTGGCCATGGAGAAGATTGTGTGGCCATATTTGCCGATTTTTCTCTTCACATATGTGTTTGGGAACCTGTTTTACTTCCGTAATCAGGTTGAACATGAGAGTAAAGTATCTATCGCTAAGTCCTTTGGCGCAGGAATCGCTCATGAAATGCGTAACCCGCTAAGTGCACTGAAAGCATCGTTTGACGTGCTAAGTTCCTTGTTACCTGATGAAAAGTCCAAAACTGCTGAATTTTACTCAATGTCTCATCAGGAATTAACCATTGCGAGAGAAGTACTCAGTGATGCGGATGAAGTCATTCAGAATGGTAATGAAACCATTGATCTTCTGTTAACTTCTATTGATGAAAACCGTGTATCAACGTCGACATTCAGAAAGCATTCTTTGAAACGTGTGACGGAAGATTCTCTATCCAGCTTCGCTTACAAAAGCACAAAGGACAAACAGGCTGTTCAGTTCAAATGTGAACAGGATGCAGAAGTTTTTGGTAGTGATACGCTCATCAAGTA

>NODE_75_length_3046_cov_2.743348

CTGTAAATGTGGGACAAGTTAGTCAGCTTATCTGATGATAATCAACAGGTTATTGCCAAGTTGCGACCAGATATGAATATCGATGGTCGCTTTGACAGTAAAGGTGTTGAGGAAGCGATTGCGGGCATTGGTGCGGCAGATTTTTGCGTATTAGAGGAAGAAATTACTCGATTTGTTAACTGCGCAAAGGAAGGAAAAAGTGATGCGCTATTAGGATTCCCTATTGCTGAAATCAGAGATGCTGTGGTTGAAGTTGTTTTATCGGATAATGATATGTTAGCCAGTATGGTAGTCACTGGTGCTTATCGCGGCTCGCCGCTCAAAGGCCCTCAGATTGTACACGCATTAGCCCAAGCTCATGTCACTAAAGGCATCAACAAATTAGCACTGAAAAAAGTGTTAATGATGAGCCATCAACTCAAACCGGGTGAGACCTTTACCCAACCTGTCGCCAAAGGCAAAAACCCTGTTCAGGGACAAGATGCAAAGTTTATCCCACTGGTCAAAGACATCACTAAGCAAGTTCTGGCTCCTTCACAAGACGCAGATGGCAAAGTCGATATGTTGGATCTCGGTGAAACGGTCACCGTAGAAGCGAAAGCTCGGTTGATGAAGCGCATCCCCGCGACTAAAGGGACACCTGGTATCACAGTGCAGGGGAAGCCTATTCCACCCAAGCCTGGCAATGATGCGATGCTGAAAGAATCGAAAGGTTCAGAAATCTCACCTGAGGATCCCAATCTCCTTGTCGCTGCAGTTTCTGGTATGCCGATGATCAAAGAGCGTACTGTCGAAGTGGAAGACGCGCTTTGCTTGCCTGCCATTGGTGTGGCAACTGGGCATGTAAAATTCAAAGGCAATGTAATCGTCACTGGAAACATTGAGTCCGATATGATGGTTCGTGCAACCGGAAACTTAACAGTTGGTGGTTTTATTGAATCGGCAGATGTACAGGCACAAGGCGACATAGATGTGGCTAAAGGTATCATCGGTCATAACGTTTCTGAAGACCAAGCGAAAAGTTGTCATGTAAAATCGGGTGGCTCTATTACAGCAAATTACGCGCAATTCTCAGAACTTCAGGCTGCTGAAAATATCAATCTTTCAGTCCACTGTATGAACAACGAAATTCGCTGTGGTAAAGATTTAACCGTCTCTGATGCCGCCGAGAAGCAGGGAACTTTAAGTGGTGGTCACGCAAAAGTAGGTGGTAAGGTGACGTGCGTTAACCTAGGTGTTGAAGGTGATACTGCAACTTACATCAATGCGTTTGCGCGTTATCAGAACTTCAAAGAGCGCCAGCAAAAATACAAAGAGCAATATAAGCTTGCCCAAGAAGCGACTATGGATGTGGTTCGTCGGGAGCTGGAATTTAAGAAAACACCGAAAGCAGAACGCAGCGAAGAAGAAGCAGAAAACATTGAGAAACTTAAACTTGAATCCAATGCGCGTCTAGAAAAAGTGAAAATGGCGCGAGATAAACATGAGTTAGAGTTTGAACAAGCGCTTGAAGACAACATCGTAGATGTAAAAAACAAAGTTTATACCCATGTTACGGTTCAATACGGTGAAGAAAAAGTTATCACCAAGCGAGTGCATGGTACGAGTACGTTTTCCTTTAACCAGTACGAAATTAAATGTGCTGCCGCTATGGATGAAGAGGCGCTTGAGCAAGAGTAACTCAGGCGGATTTGATAGCAAAACAGCCAGCATTATGCTGGCTGTTTTTATTTTCTGAGTGCAGCGAATAGGTTCTGGTTACGATGCTTGGTAGCGTGGGTTACTAGAGCGATGACTGCACAAGACATTAAGACCTTTCCAAGATCTTGTATTTGCCCAGCGATAGCCAGACCAATACCTATCTGTAGGTAGTAGATAAGCCCGAGAATCGCGCCGCCGCTGCCAACATGTTGCTTGTAATCTTTTAGTGCTGCACTGATTACATTGGGAATCGCGATACCAAATGCCATTACGACCAGCATCATTGCGAGGACAAAGTAGATACTGTTCAGAGTCAGATAGATACTTATGCTCCCTACGCATAGCAATGAAAGGGCTAGGCCAATCAGAGTTGATGGCTGCGTATGCTTGTTTAGCAGGTATTTATTACAGTAGCTGCCAATCATTGTACCAATCCCTAAAGCGATGCCGCTGTAGCCAAATTGGGCAGAGCTATAGCCAAGCTTAGCAAAGGCAAATGCGCCCAGTTGGTAATAGGAAAACAGTGCGATATTGAAGACTGCAATCAAGATGGTGGAGTACCATATATGGCCATCTTTGAGCATCCTCCAGCCTAGCTGGTGGAGATTAAGTGGCGTCTTCGATTGCTGTGTTTCTGGTAACCTGAAGAGGTTGTAGAACAGTAGTAGGATCGCCATGCTAAACAGAGCAGAAAATACGTAGGTGTGTCCACCAGCTTGAGCAAGCTGTCCACCTATGGCCATTCCTATTACGGGACTGATCGCGATTCCCATACCCATCAGCCCAAACACTTTAGCCAGAGCTTCTCCGTCGAATACATCTCTCAATATCGTTTGAGTGACCACCGAGCCAACGGCAATGCCAAATGCGCTGGCAATTCGCGCAGCAATCAGATACTCAAACTGGTCGGTAAGCATTGCGATTAAAGCGGATAAGCCGTAAACCGCAATACCTGCAAGCATTGCTGGCCGTCGGCCCCATTTATCCGCAGCTATCCCCCAAAATACAACGCCCAGTGCAAACGCGATGAAATAGACAGATAAAGTCTGCGAAGCTTGCGAATCAGTGACTGAAAAGGCCTCGGCAATCGAGTGGAGTGCTGGGCTGTAAATCGTTTCCACGATCTGCGGAAACATGAGCATTACGAACATCAGCCAGAGTGAAGGCTTACGTTTCATGATACGTCCTCAATAAATTTAACTTGAGACGCAGTATAAGGAGCACTACTATTTTTCTATTAACGATAATAATACAAAAAACATCAAAATAAGGACAATGGTGTGCTGATCGATGAACATTCTGTGATAGATCCCGACAGTGTTAATTCTAAAGTGGTGGGCATTGC

>NODE_76_length_3036_cov_2.359919

GTTGAGCCCAATAAACCACTCAAACAAGGGGACATTCTCTTCATGATTGACCCTATCCCATACGAAGCTGAAGTTGAAAAGCTTGAAGCACAAATTAAAGAAGCCAGCCAAGGCGCACTTGGACTGGAGTCTGATGTGCAAGAAGCGCAAGCCGCTCAAGCTCAAGCCATCGCAGATAGAGATAAGGCACAACGTGAATACGCTCGTTATCAACGTGGCTACGATAAAGGCGCCTTCACCGAGCAAGAACTGGACACCCGACGCCAAGAATACAAAGCCACTGAGGCTGCGGTCAAAGTTGCGGAAGCCAGAGTGGATCAAGCCAAGATAGCACTCAACTCTGAAATTGGTGGAGAAAACACAACAGTGGCGCGTTTGCTAGCGGAGCTGCGTCAAGCTGAGTTCGATCTTGAACAAACCATCGTCAGAGCTCCCACCGATGGTTTTGCAACGCAGTTAGCTCTGCGACCTGGGGTTATGGCAGTTCCACTGCCGTTAGCACCTGTGATGACCTTTGTTCATACAGAGGAAACGCTTTATGCAGCCGCGTTCAGGCAAAATTCCTTACAACGCTTACAGCCGGGTTATGAAGCAGAATTTCTCTTCAGAGCGCTTCCAGGCAAGGTCTTTAAAGGGGAAGTAGTAGATGTGTTGCCCGCCATTGGCGAAAGCCAATTTCAGGCCCGTGGCGCCCTGCTTGGCACGGAGGCATTGCGCACCAGTGGGCGAGTCTTTGTTACTCTGAAGATCACTGATGACTTATCTGACTACCACTTACCCATGGGGACAGCGGTCGAAGTTGCGGTTTACTCCGATCAATTCACTCATGTATCCATTATGCGTAAAGTACTCATTAGAATGAAAAGCTGGCAGAACTATCTCTACCTAGACCATTAAAATCCGTCAGAAAGCAAAAAGGCCTCTTAAAGAGGCCTTTTCTTTTATATATGAACTTATTAGCCGATAAAGGAGATATACCCTTGAAGGATAATTAAGTTAACAATGTCGATAAAGAAAGCACCAACAATCGGCACCACCATAAATGCTTGCGGTGAAGGGCCAAACTTATTCACCAGAGAACCCATATTCATAACAGCTGTCGGTGTTGCCCCAAGGCCAAAACCGCAGTGACCACCCGCAATAACAGCTGCATCGTAGTTTGAGCCCATCACTTTGAACGTCACAAAGTACGCAAAAATACCTAAAATGACAGACTGAACCGACAGAATGATTAGGAACGGAATCGCCAAGTCAAAGATGTTCCATAGTTTCAGGCTCATCAGTGCCATAGCCAAGAACAAAGATAGCGAGACCGTACCCAAAATATCAACGGTTTCTGCGTCTACCTTGTGTGTCTTAGTAACTTCCATAACGTTGGTGATGAAAACACCGATGAACAAAGCGTAAACAAAGTCTGGAATCATCAGCCAACTGATTTCCAAGCTACTCACCCACTGTTCAAGGTATTTAGCACCAGTTACACAGATAAGAAGAATAAACAGGATTTCAATAACCTTTTTCGCCGTCACCTTATCTTCTTCGTATTCGTTATACGTCACGACTTCAGGGAACTTTTCATGGGTTTGTGTTCCGCGTCCGTATTCAGATTCGAAGTTATGTTTAGTAACCAATTTCTGAGCAACCGGGCTACCGATAATACCGCCAATGATCAGACCAAAAGTCGCAGATGCCATTGCAATTTCAAGTGTATTACTCAAACCGTAAGTGTCAGTAAAAGTCTGAGACCATGCTGCACCCGTACCATGCCCACCCGACAATGTGATGGAGCCAGCAATTAACCCCATTAGAGGATCCAAACCAAGCGCTGTCGCCATTGACACACCAACACCGTTTTGAATGATGATGTAAAAAGACGCAACGGCTAGGAATAGAAAGACTTTAGCGCCGCCTTTCATCAGTTGAGTGTAATTTGCAGCCAGACCAACAGTGCTGAAGAACATCAACATAAAGGTGTTTTGCAACGGTAATGAGAACTCTAAGCTAACCCCATTGAAATGCATTCCCGTGATGATCATCGCAACGATCAAACCACCAACAATAGGCTCTGGAATATTAAACTTCTTCAGAATAGGGAGCTTTGAGTTTACAAAGTGACCAAGAAACAACACGCTGATTGCGATTAAGAAAGATTCTAGTGGTCCAACCGAAATTATCTGATTCATATAACCTCTTTATTGTCTGTCACTCATCTTCCCTAACGAGTTAAGAGCATACTGCTCTGGGCCATTTTGCTACCAAGGTCTCAGGTAACAATTGCAGGCACTGATTGAGATATTCTCTATTGAAAAACGTCCTCTCTACAGCGGGTCTCCCCACTCCATTTCAACAGCGAAACCCTACGGTGTACTTTAGGGCTATAAAAAGAAAGCGGACATTATTAATAAACTCGCAGGCGTTTGTCGAGTATAGACAGAAAATTCGTGTCATGAATCCGTGTAAATTGACCAAGAGTTAACCAAAGGACAAAAAAAAGCCAACCGCAATGAAGGCGATTGGCATATATATAGTGTGAACAAATTCATTCACTAACAACGTCAGTTGGCTAGGTGACCCTCGGCTTAATAAGGGTCAATCACATTAATGCATGTAGCGTGCCAACTTTAAAACCCATAAAACCACAATATTGATCCAACTTTTAGCCAATTTATTATATTGAATGTGCAAAATGAAAATTGCATTTTGCAAAAAAGTGCAATTCGATATTAAACAAGTATCTTATCAACTAGTTTATCAATGCACTATTTTCATACTTTTTTTACGTTATTGTCGATATACTCCCACTACCTGAACTGAATCGCTGTATCGAATGAATTATCTATCCACTTTCTGTAAAGGCTTAGCCATGGGCGCGGCCGACGTTGTACCAGGAGTTTCTGGTGGCACCATCGCCTTCATCACAGGCATCTATGACACATTGTTGGAAAGCATCCGACGCATTAATCCAAGCCTGATTGGTGTATTGAAACGCGATGGCGTTAAAGCTGCGTTTAACCATATCAACGGCTTCTTTCTTAT

>NODE_77_length_3036_cov_1.946604

CCCTCGACGCCTTGGCAGCGGTGCTGCAGGTCAAACAACCCCGCGTTTTGCCAGCGAACCATCCACGCGTTAGGGTCTTGCCTGTCTACTTCACTGACGCCAATGTCATTGTCTAACGGCGCGCGGAAAAAATTATCTTGCAACGGTGATAGCAGCATTTCACGGTTAGATTTTTTCCAACTTTCAATAACACCTGTCGTTGAGTCGATAAACCATTCATTCTCCGCAGCGGTTACTAGCAAACCTTGATTTTGCTGTGTGAACATTGCAGGCGTGAACGTAACATGTGTTATTGATTCAACGTGTAGTCGCTTGCCAATAGCCATCTGTTCTTGGGCGACAATATGGCCTGCGTCTGCCCAGGACGTCGCCTGTTTATGACGAATTGCAACATTTAGGTGGCAATTAGACGTAGGAAGTTGGAAGCTGGAGTCAAGCTCTAGCACACACGTTTCTTTTGCTTCAAGTGAGAGTTCCAGCTCCCCACTGTGAACAGTTTCGCCACAATCGCCAATAACTACCCAGTCCAAACTGCAATTTGAAGTGGCAGCAAACAATAACTCACTTGTTACTTTCACTGCTAATGGGCATGTTGATACTAACTCAAACGTGTAAGGCTGCTGAGCCCGTTTAGCCTCTAATAATGTTGGATGAGGTGTTCTGTCTGGAAAAACAAGGCCGTTGATACAGAACTGACGATCATTGATGGCATCTCCAAAGTCACCACCGTAAGCCCAATAGTGCGTACCATCATCAGAGTACTTGTCGAGTCCTTGGTCTACCCAATCCCAAATAAAACCGCCTTGTAATCGAGGATGCCTCCTAAACGCTTGCCAATATTCAGCGAATCCGCCCAAACTGTTGCCCATCGCGTGCGCGTATTCACACAAAATGATTGGACGATTTTCATCCCCCATACCAAGCCACTTTTTGAGTGCTAACTTGGGCTCGTCTGTGTGCCCTTGTTGCTGATCAGCATCGGTACGAGCATACATTGGACAAACAATATCCGTCGCGGCAGTATTGGAGCCTCCCCCTTCGTACTGGACGACTCGTGATGGATCAACCCTTTTAGTCCACTGATACATAGCATCGTGATTGGAGCCATAACCAGACTCATTGCCCAATGACCAGATGATGATACTCGGATGGTTAAAATCGCGTGCAACCATACGCGTCATACGCTCTAGAAAAGCGCTTGCCCACATTGGGTCGTCTGCAATGCGTCCCATTGGCGTCATTCCGTGTGTTTCAATGTTGGCTTCATCGACGACATAAAGACCAAGACGATCACAGAGCTTGTAGAAACCTGGCTGATTTGGATAGTGTGAGCAACGCACCGCATTGAAGTTATGCTGCTTCATCAGCTTCAGGTCTTGTTCGACACGTTCAAGGCTCTCTGCATGGCCTGTCGCTGGATCATGCTCATGTTTGTTAACACCCCGAATAATCAAAGCTTCGCCATTTACGCAAAGTTGGCCATCAATTACCTCGATTTTGCGGAAACCAACGTCATACGCCTCACTTTCAATGACGTTGTTGTCATTGCTTAGCAAGCTCACAACCAATCGATATAAGTGGGGCGTTTCTGCGCTCCATTTCTTTGGCTGGGAAACGGGAAGCGAAACGTGGCAGCGATCATCATAGCCTCCCTTTTCGTCAATAGGTCTTGTTCCAACAGGAGTGACTTGGGCAACCACTTCATTTTGGTCATCGAACAACACCGTCCTGACTTGCAAACCTCGGTCGCGAGTGGTCTGTACTTCGATTTCTAGCTCGCCCTCTTGATAACTGTGCTCAAGATTCGCGGTCACCCTTACATCTTTAATATGCTCTAAGGGCTTGTTCATCAGTCTTACAGAGCGGAAGATGCCGCTCATCCACCACATATCCTGACCTTCCATGTAGCTTCCGTCACACCAGCGCAATACCATAACGGACAAGGTATTGGTGCCTGTTGTCAAATGAGGGGTCAGGTCAAATTCAGCGGCTAGACGGCTGTCCTGAGAATACCCTACCCAAACGCCATTACACCATACGTAAAAAGCGCTGTTTACGCCTTCAAAGACAACCCGGGTAAACGACGCCTGATCCCAGCTTTGTGGCAGTTGAAAGGATGTCTGATAACAGCCGGTAGGGTTATCTTTTGGCACAAATGGGGGATTACATGGAAACGGGTATTTCACATTCGTATAGATAGGATAATCAAACCCTTGAAGTTGCCAGTTACCTGGAACGGTAATCGTCTCACCATCTAGGTTTTCTTTTGCCCAGTCAGCGGCCACATGCTCGGGTGAAGGATATAAAGCAAACTGCCAATCTCCATCCAAACTCATAATGGATGAGCTCACACGATCATAGCGCGCGTCACACTCATCTCTCCAACTGAACAGCGGAGAATGCGCCTGTAGGCGATTGTATGAAGTAAATTCTGGTGTTTCCCAATTACGAGGGCTCATTGTGGGCATGTTTCTAACCTAAGCGTTTGTTCAACTGCTTATGATCCTAACGACAGAGGCGTTTCAGCACTGTGACAACTCTCACATCAATGTAAACGTTTCCAGAAATGAAAAATTGCAGATAATTAGAATGAAACCCATCTATCACTTTGAAGGATTTTTTTGAAAATCTTTAGCGAAACCTTATAAATTATTTATTTTACAAGCACTTAAACAATACCCAGTTAAAACCACATGCACAATTAAATTTATTACACATCAAAAATAGATATCATTAGTGAATCAATTACATGTTCCAAACCACCTTGTTACAGACGTGTTACATAAACAATTAGCAAGTTACATTTATATAGAGAATAATTAAATATCATTTAATTTCAGAGATTTAGAAGGCGGTCACACTAATTAAACGTAAAAAGCCCAAGTACAGCTGTACTCGGGCTTGAGTGAAGCGTGCAACTCAAGGTTTATGTATGAACTTACTGGAAGTTGGCGAGTAGGTTTTGGAAATTAGTATCAATGATAAAATATGTTGCAGCGTACATGATGGAAAATACGATGGCTGAAACACCTAAGG

>NODE_78_length_3024_cov_2.230743

GCGTATTGTTGTTGTTCCTCGTTCAAATCGTATCGATTGCGATCAGTTGATGAACCACCTGTTTGCTTCGACAGATCTGGAGAAAAACTTCCGCGTTAACCTCAACATGATCGGTCTCGATAACCGTCCACAGGTGAAAGGTCTGGTTCAGATCTTATCAGAGTGGATTGAGTTCCGTCGCGCAACTGTGCGCCGTCGCTTGCAGCATCGCTTGGATAAAGTCCTTGCACGTCTGCATATTCTTGAAGGTTTGTTGGTTGCTTACCTCAACTTGGATGAAGTGATTGAGATCATCCGCACTGAAGACGATCCAAAAGCCGTACTGATGGCTCGTTTCGGCATTACGGATATTCAGGCTGACGCGATTCTTGACACCAAACTTCGTCATCTTGCCAAGTTGGAAGAGATGAAAATCCGTGGTGAGCAGGAAGAGCTAGAAAAAGAGCGTGAAAAGCTTGAACAGTTACTGGGCTCTGAGCGACGTCTGAATACGCTATTGAAGAAAGAAATCAAAGCGGATGCAGAGAAATACGGTGATGATCGCCGCTCTCCTCTTGTTGAGCGTGCTGAAGCGAAAGCCTTGACTGAACGAGACCTTGTCCCGAGTGAACCAATTACCGTTGTGCTTTCAGACAAAGGCTGGATTCGTCACGCTAAAGGTCATGATGTCGACGCCGAAGGACTTAACTATAAGTCTGGTGATAAATATCTGGCCCATGCTCGTGGTAAGAGTAATCAGCAAGCAGTATTCCTCGGCAGTGATGGTCGAAGTTACTCGCTTGAGTCGCATTCTCTGCCGTCTGCGCGTAGTCAGGGGGAGCCGATCACTGGACGTCTGAACATTAATGCAGGGACTAACATTCGTCATGTGGTAATGGGCGAAGAAGATCAATTATGGCTAGTGGGTTCTGACGCGGGCTATGGTTTCGTTTGTAAAGGCAGTGATCTTTTATCGAAAAACCGCAGTGGTAAAGCATTGGTCACACTCCCACAAAATTCGGAAGTGATGACTCCTGACACCATTAGTAACCTAGATAATGATGAAATCTTAGCGATTACCAACCAAGGCCGAATGCTGCTGTTCCCGATTAAGGATCTTCCTCAATTGGGTAAAGGTAAGGGTAATAAGATTATCAATATCCCAGCAGCCAAAGCGAAAGAACGCGAAGAAATGGTATCACATCTGATGTCGTTGCCTCAGGGGGCTTCAGTAACCCTTTATGCAGGTAAGCGTAAGCTAGGTTTGAAACCGGCAGATCTGGACAATTTCCGTGGTGAGCGTGGCCGTCGAGGCAGTTTACTACCGAGAGGGTTGCAGCGAGTGACGCGTATAGAAATAGAAAGCTCTGCGACAGAGCAAGAAAGCGAATAAAACAAAAAATCCCATGCCAAAGAGCATGGGATTTTTTTATCATTCAGTGGTTAAATTTCTCGGGTATCTTCTGCGATCGTGACGCTAAGGGTTTGTTCTTTGCCCTTACGTAGGATGAGCACATCGACGGTTGTTCCCGGCCTCAATTCAGTTACAATATCCATTACACTTTGACGACCATTGATTTTTTGCCCATCAATTTTAAGAATAATATCCTGGGCCTGAAATCCTGCTTCAGCACCCGGTCCGTTAGGATCAACGCCCAGTACCACAATGCCGCCTAAATGCTCATTACCCAATAAACGAGAAGTTACCGAATTAATATCTTGGCCGTCGATACCTATATAGCCTCGGATAACACGACCGTCGGCAATGATCTTCTGCATGATTTTATTGGCGAGAGTGTAAGGGATCGCGAAAGAAATACCGTAAGTTTCAAGCTCTGTCGCTTGCTGGAAAGACGCAGTGTTGATACCAACCAGTTCACCTCGTGTATTCACCAATGCTCCGCCAGAGTTTCCTTCATTGATGGCAGCATCTGTCTGAATAAAGGCTTGTCGGCCGCCGGCACTGATTGAAGAGCGGCCTGTTGCGGACACGATACCGAAAGTGGTTGTTTGACCTAAGTTGTATGGGTTGCCGATCGCCAGTACTACATCACCGACAGTTGGTGAATAGTTCGGATTGAGTGGGATAACAGGAAGGTTGTTGCCTTCAACACGCAGAATGGCGATATCCGTACGTCTGTCTGTCCCAACTAATTGCGCTGCGGCGACGCGACCATCCTGCAGTGCGACGACAATTTGATCGGCCTGAGCGACAACGTGGTAGTTGGTAATGATATAGCCTTTATCACTGACGATGACACCTGAGCCTAATCCTTGAGTCAGTAGCTTTGAACGGTCATCTTCTTTGTACTTTCGGCTGTAAATGTTCACTACCGCAGGGGCGGCTTTACGGACGGCACTATTAAATGAGATTTCTTGTGCGGAAGTAATGTTGATTTCATCGCGGAAATTCTGAGGAAGAATAGTGGAACGCAGAGAGGGGATAGCTAATAGCAACAACGCAGCTGTCGCCAACCCTAAACCTATTGAACGCAATAAGAATTGCAGCATATCTTCCTAACCACTTCAAAATTCAGACAAGTGGCGAAAGAATAGCATTAGATGAGAAAATAACAAAAGGACGGCAGGAGATCCTACCATCCTTTGACATTGACCTCGCGTTTTTAACGCACGACTAGATAAATGGTACGTTCACCACGTTGAATATTCAGAGCCAGTACGCCAGGTTGTTTTTCTAGCACTTTTCTAAACTCTGCAAGGTTCTTGACTCGCTTGCGGTTCACACCAATGATGATATCGCCTTTCGCCAGTTGATAAGCTTCGGCTTTTGACCCTTTCTCAACGGAAGCGACTTTCACACCTTGCACGGAATCATTTGAGGTGGTATTGGTCAGCTCTGCACCCGATAGACCTTCATGCAGTTTATCTGCTGACGCTTTAACGTTTTGCTGCTCACCCAAAGTGACATCAAAGCTCTCTTTTTTACCATCACGGATAATGCCTAGTGAGACCTTCTTGCCAGCACCGAGTGTCGCAACCTTGGCACGTAGCTCACTGAACGAGTGAATATCTTTGCCGTTGACT

>NODE_79_length_3018_cov_2.430466

GCTATCAGCAACACTCGACTAAACTTTGCGTCGTTGACCCAGAAAATTCGGTCTTCCATGATTTCTTCAAAACAGGCAACCTTGAACTCAAAGGAAACTGCGGAAGTAAGATCGAAGGTATTGGTCGACCTCGCGTAGAACCAAGTTTCATTCCGGGTGTGATTGACGAGATGCGCACTATTCCAGATGCCGCTTCAGTCGCCACTGCGCACTGGCTTGAAAAGATCCTTGGCCGTAAAGTGGGCGCATCAACAGGGACTAACCTGTACGGTGCACTACAGATAGCAAGTGAAATGAAAGCACGTGGTGAAACAGGCTCGATCGTGACACTTTTGTGTGATAGTGGTGAACGTTACCTAGACACCTACTACAATAGTGATTGGGTAAAACAAAACATCGGCGATTTGCAACCTTACCTAACGAATTTAGAGACGTTTGAAGCAACTGGCCAAATCTAAATAACAATAACAACGTCAGTTGGAAACTCAAACGCCACTCATCTGAGTGGCGTTTTCATATTAAGCCAGAAGCTGCAAGCTCTGATTAGCTCTTTTTCTGGCGTGCTTCGAAAGCGGCGAGTTGTTCAGGTGTCGCTTTCGGCTGATGGTTCTGCTTCCACTCATCGTAAGTCATGCCATAAACACGCTCACGAGCGTCATCAATGTCCAGCTCTAAGCCCTGAGTTTCAGCTTCAGCCTTATACCATTTACTGAAACAGTTACGGCAGAACCCCGCAAGGATCATCAGATCAATATTTTGCACGTCTTTATTCTCATCAAGGTGAGAGAGCAAACGGCGAAATACCGCTGCATCGAGTTTGTCCTGCTCTTCTTGAGAAAGTTTTTTATATTTAAATTCAGCCACTTTACTTACCTATTGTTGTTCTGTATCGAATTATCTTATCAAGTCTTGACCCAATACAAAAACGCCCAAGCACTTGGCTTGGGCGTTTATTACAAAATTGTGCTTAACCTTGAATACCGACAATCAACCATGGAGCGTTAGGTACCGTTAGGTCACGCTCAAGGTGCCAGATGTCTTCAATATTCTCTTCAACATTCTCTACAGCATCACGGTAGCGGCCGCTAAACTGTAAGCTAAGCTGAGCTTTACTTGCGTCATAGTCTGCACGAACGATTTCTGCATCAACGTACATAACATCAGTGTGTTGCTCACCTTCCAGTTTCGCACGCTCAGCTTTCAGGTCTTCAAACAGACTTGGAGAAACATATTCTTCAATCGTATCAAGCTGGTTGTGGTTCCATGCGCCTTGAAGAGTTCGGTAATGCTCACGAGAGCCATTAATAAAGGCCACTTTATCAAAACCCGGTGGATAGTTGTGTGGAATATCAGATTGCGCACCAAAGCCAAAACCACCTTGGCTACCAGACTGAGGCTGTTGCTCGAAGTTATGAACATTCGGTTGCTCAAACTTAGGTGCAGAACCACCAAACGCAGGCTGCTGTCTTTGCTGATTCATACTGCCCTGCTTCGAGCCAAGCAAACCGCGCATCAATTTGAAGATGATAAACGCCACTAAGCCCATGATCAGGATATCCATGAACTGGATTCCTTCAAACGCGCCACCGAAGAATGCTGCTAAAAGACCGCCTGCTAAAAGGCCGCCAAGCAATCCGCCCATTAGGCCTTTTTTACTAGACCCTTGCGTTTTTGCGCCCTGGTCTTTACCAATCGTGTTTGTGTTTTGCTGTTGTTGCTTTGGTGCAGGAGCTGTTTTAAAGCTCTTACCAAATGACTTACCGCCACCAAACTTCTTCGCTTCTGCGATAGGAGTAATAGCAACAGATACCATTAGCAAAGCAACGAGGGAAAAAAGTCGTTTCATATTTTTCCTTAGGGTTTCCTGTATAAGGTAAGAATTATACTTATGGGGCAATAGTAGCCTTTGTATCTAGAGAATGAAATATGCCAAAGTCGAATACATGTATCAAAGTATTGCAAATTAGCGCTTCGCCAGTTGCGCTTAGCCAAGACCCTTATTAGAATATTGAACGTTGTTCATTTTTGAAAATTTATCATGGCCCGTAGAAACGATCATACGCGTGAAGAGCTCATCGCTCTCACTCTCGACACTGTCAAAGACTTCCTCAGCGAAAACTCCTACCACGAGTTAAGCCTACGTAAAGTCGCTAATATGATCGGTTATGTGCCGAGCACGCTAGTCAATGTGTTTGGCAATTACAACTTACTGCTACTCCATGCTGTTGCCCAGACTCTCGATGAACTGGCTGAAGAAGCTAAGGTCGTCGTCTCTCAGAGCCAAGATCCTAAAACCGCGCTCTACGAGTTAGCCTACTGCTATCATGATTTTGCTCAGCGCCACCCCCATCGCTGGCAGCTGATTTTTGAGCACAACATGAACGGCGAAACCCTCCCTGAGTGGCAAGCTGAACGCATTGACAACATGACTGGCATGCTTGAACAGCTGCTGCAGGTTCTTGCACCTCATCGCTCATCGAAAGAAGTGTTGCAAGCAAGCCGCGTTCTGTGGTCAGGTGTTCATGGAATTACGCTACTTAGTGTCGATGATAAATTCTTCGCAGCTGAGCCAGTTGACGGCAAAGAGCTCATCAACAACTTGCTATCTCACTATATAGCGAACTGGTAATCGCATCCTAAAGGAACAGGGATAATCATGCCAAACAGCCAAACCTCTCTTTTAGGGCAGAGACGATTTCTGCCCTATTTCGTGACCCAATTTTTTGGTGCTTTCAACGACAATATCTTCAAAAACGTTTTACTGTTGTTTGTTGCTTTTGCTGGTGTTGGAGCGCTACCTATCTCTAGCGACTTGTTTATTAACCTAGCGGCGGGACTATTCATTCTTCCCTTCTTCCTTTTTTCCGCGTCCGCTGGTGTTTTAGCCGATAAATACGAAAAGTCTTGGTTCATTCGCAAAGTAAAGCTGGCTGAAATCGGCATCATGTGCCTTGGCGCAATCGGCTTCATTACTGAAAGCTACATCATTCTTCTCATTCTACTGTTTTTGATGGGGAC

>NODE_80_length_2995_cov_2.512680

GAACGAAACGACCGCCATTGCCGCGCCAATGTAGAATACGGCTGACGGGGACACTAACCAGATAAGGCCAAAGGATACCGGGATAAATACCGCTGCAATATGATTGATGGTGAATGACACACCCGCTGTCGAAGCCATGTCTGCAGGATCGGCAATTTTTTGTAGATAGGTTTTGATCGCTAACGCTAAAGCGAAGAATAGATGATCAATAACGTACAAGGCTGCCGCCCATTCCGCACTTTGCACCAAGCCGTAACCAATAAAGACAAATATCAAACCTGTGTATTCAAACAACAAAGCTTTACGTTCACCCACTTTACCGATGAACTTGCCAATTCTTTTCGCGAACAGGAAGTTAAACAGGTAGTTGATCAGAAATAGCATGGTGATATCTGCCGCCGAGTAACCAAACTTCTCCACCATCAGGAAGCCTGCGAAAACAGTAAAGATTTGTCGACGTGCACCACTCATAAACGTCAGAGCGTAGTACAACCAATAACGCTTGCGCAGCACCAGTTTTTTATTCTGCTGGACTTGGGTTTGGAACTGAGGAAATGCAAACGCCATCCAAACCACCAGTAGAAAACCAATGCCACCAAAAGTCAGATACACCCACTTGAAGTCCATTTGAAACACTTCCAGACAGACCCATAGCGCACCATAAGTGATTAGCGATGCCAAAGCGCCCACAGAAATAAACTTACCCAGCATCTCTGGCGCTTCGTCTTTTGAAAGCCATTGCAAAGAGAGAGACTGCTTTAGGGTTTCAAAATAGTGAAAACCAGTAGACATTAACAAAGTCGTTAATAGCAATCCAAACAATGAAGGGAAAAAGCCAGTAATTGCGGTACCCAGAGTAAGCGCCCCAAGCGCGAGTAGCATGAACTTCTGTTCGCGAATGAACATCAGAACAAATACGGCGGTGAACGCTAAGAAACCCGGAATTTCACGGACACTTTGAAGAAGACCAATATCAGCACCATCAAAGTTCGCTTTTTCAATAACAAAGTTGTTAAGCAGCGCCATCCAACTAGAGAAAGCGATGGGCACCACAATAGAAATGATGATCAAAAAATTCTGAGGAGTTTTCCAGCCAGCCGACGTGTTCATTTTTTATTCCTTTTATGAGCTCTCAAACACCTTAAGTCTTCGCCAACAAATAAACAAACTTATTTACAATTTATACGTCTTCGCCAACGTTCAAGCTTCAAACGTGTCAGAATTAAATTTAGACCTATTAAAACAAGTGAAAACAATGCTTTATCTTTGGTTACTTGCTCGTTATGGTTGAGTCATGTTTAACCAGTCGAGAAGGTAATAATATGGAAATAATAATTCGCCCTACTCTAGTGAGCGATGCAGCCGCAATGTGTGAAATTTTTTCACAACCTAAGGCGCAGCGTGAAACCCTCCAACTCCCTAAGCCATCTGTGGCAATGTGGACACAACGACTGGAAAACATGCCTACTGGAGTCTATAGCTTTGTCGCGGAACTAGCCGGCAAAGTAGTAGGGAACATCGGTTTTGAGCACTCTCAGCGACCGCGTGCCGCTCACCGCGGCACTTTTGGGTTAGCAGTTCATGATGATTATCATGGAATGGGCATCGCCAGTAAGCTGATCGAAACTGTCCTTGATTTAGCGGATAACTGGCTGCAAATAAAGCGCGTTCATCTCGAAGTTAATACCGATAATGGCGCGGCCATCGCGTGCTATAAGAAATTTGGCTTTGAAATCGAAGGTGAAGCGAAGAATGCGGTATTCAGAGAGGGCGAATACCAAAGCACTTATTATATGGCAAGGATGAAACCAGAGTAATAAGGGTAATAGCGAGCTATCGAAAAGGCTTACCTCATGGGTAAGCCTTTAACAACTCAAGCGCCTTGTCAGCCAACGCTTCTGTGTAATCATTGATTTTCCAATGGCCGGGACTCCAACCTTTGACAAATCGATGAAAATCCGCCCATGCAATCGCAAATAACGGCCGCCAGCTTTGCTCGACATCGTTGGCATCAATGTCAGGCTTTAATTTGTTGAGTGCCCGTTTAAGGTGGGAAAAATAGGTATCCAGAATCCAGTTTTCCATTTCATCACACTGTTCCGGCTCAACCGCACTGCTCATGAACAGAGCGACATCTTTCATCGCACATCCCTGCCCAACATACTGAAAATCGACCGCTGCGGCTTCACTGCCATCAGAAGTAAAACAGAAATTAGCCAGTTTGGCATCACCATGAACCAGTGTCTGATATGGTGCTTGACGAAGCATTCGGTCAATTCGAACCGCAGCTTGTTTCAAAGGGGTATCATCTAGCGCTTCCAGCTCATCAGGCCTTGTATCTAGGTGCCAATAAGTACCGATCTCCCACAAGCCGTCCCCTCGCTCTCCAATATGCGATGCATGAAACCAAGCAAGCCACTTTAGCGAGGCTTCCATTTGCAAAGACCCCGCACGTTGATGCGTTTCGGTGTAACCCAGTTGATGTAAGTCTTCCATCACCAGAAGGAGTTCCGTCCCTTGCTGTTCAACGACTATAGGCTTAGGTTGCGGACAATAACAATGGGCCACCTGAGCAAAGCTCTGATACCAATGCAGCTCAACCTGATAGGATTTCAGTTTACGTTGATGGGAGAGATCGCTATTCCAGCCTCTCGGATGATGGGAAGGTTTAGGCAATTGCACATGCTTGATAACAATACTGGTTTCATCTACATGGAGCCTGACCAATTCGCCATAGCCACTCCACAACGATTGCAAAACTTCAGTGCCTTTAAGTTCTGTATAACCTAAACGTTGACTCAAAGCTTGGTAGTCATAGGAGGTGCTCATGGCTGTTCCGCAAGCAAGTGGGACCATTCTGGATGACGGGACATATAATGCACAACGTAGCTACAGACAGGTTTGATTGTGACTCCAAGACGCTCAAGCTCTGGCAAGATCGTTTCCATCATCACTTTCCCATAGCCTTTGCCTTGCAAAGCTTCAGGTACTTTAG

>NODE_81_length_2975_cov_2.347481

CTACAAGGATGTTACAACGCGCAATCGGCTCACGAATTTCTTCGATATCATTCACTGCTGGTAGCTTAGCTGGGCTATCAACGTAAAGAATTTCGCCATCCGTTTGCTCTACTTCGTACACTACCGTTGGCGCAGTGGTAATCAGGTCGAGATCGTACTCACGCTCCAGACGCTCTTGGATGATTTCCATATGCAGCATGCCGAGGAAACCACAGCGGAAACCAAAGCCTAGAGCAGCTGAGTTTTCTGGTTCATAGAACAGAGACGCATCGTTTAGACTCAGTTTACCTAGTGCATCACGGAAGTTTTCATAGTCATCCGAAGAGACTGGGAATAATCCGGCGTATACCTGAGGCTTCACTTTCTTAAACCCTGGTAGCGGATCTTCACAACCGTGCTTAGCTAGCGTTAGCGTATCACCAACAGGCGCACCTAGGATATCTTTGATACCACAGACAACCCAACCTACTTCACCCGTTTGCAGTTCCGTCGTGTCTTCCTGCTTAGGTGTGAAGATACCAAGACGATCCACACCCCAAACTTGGCCAGTGCTCATCACCTTGATCTTATCGTTCTTCTTCAGGACACCATTTTTAATACGGACCAGAGAGACAACACCTAGGTAGTTATCAAACCAAGAGTCAATGATCAAAGCTTGCAGTGGCGCTTCTGGATCACCTTCTGGTGCCGGAATCGCAGAGACGATGTTTTCTAGTACGTCTTCAATACCTAGGCCCGTCTTAGCCGAACAGCGCGTGGCTTCCATCGCATCGATGCCAACAATTTCTTCGATCTCTTCTGCTACTCGCTCAGGATCCGCAGCAGGTAGATCGATCTTGTTCAGAATTGGTACCACTTCCAGATCCATTTCGATCGCGGTATAGCAGTTAGCCAACGTCTGAGCTTCAACACCCTGACCAGCATCAACCACCAGCAACGCACCTTCACATGCCGCTAGAGAGCGAGAAACTTCGTAGGCGAAGTCAACGTGCCCTGGAGTATCGATGAAGTTCAATTGGTAGGTTTCACCGTCTTTAGCGGTATAGTTGAGTGTCACACTTTGAGATTTAATAGTGATGCCACGCTCACGTTCAAGGTCCATGGAGTCCAGAACCTGAGCTGCCATTTCACGGTCACTCAATCCACCACAGTCTTGGATCAAACGGTCTGATAGGGTCGACTTACCATGGTCGATGTGGGCGATAATCGAAAAGTTACGAATGTGCTTCATGATTTGGTGTGACTAAACTCTTTAAAATAGGGACATAAGAAAGCCGTCTTGTGACGGCGTTTCAATCAAGTTGGCAGATTCTACCCAATTTCTGTGCACTTCGCACTAGCTAATTGGATCTCCCAAAACCCGAAGTAATATGACTTCTCGCAGCGACTGGCGTTCAAAATTAACGGCCAGACTTTTTGCGCCATAAATGCCTGCCACAGTAAAGATAGCAGATGTCAGGATAGCAATACCTTCGCCAAGACCAAACGCAGGCGCTAATAGCCACTGTCCAACCGCAGCGCCTAGAATCATGGCAAACAACGGCACTAGATAAACCACTGCGGCCGATTGCAGCAGGCTTTTTTCCGGCAAGCCGATTTCCACCACCTGACCTTCTTTAACCCGTTTGCTGGTCACTAGGTGCCAGTGCAGAGACTTGTTTCCCACCGCTTTTGAGACAATACCGGTACCACAGCTTTTTTGCGACGAGCAGCTGCTGCAGCTGGTTTGCTGTTCACAACTCAGCTCCACATCATAGCCCTTAGGCCCTGATTTCACTGCAGATACTGTCGCTAAAGCGGTCATCATTGCGTGGCACTCGCTTGTTTATTGATGGTGACCGATTGAGCAATTCGCTTAGCGGTTGCTGGCGGAATATCTCCGATCACAGAGATCTCTTTGTCACCTTTTACGAAGCTATGAAGTGTGCGACGCCCTTGACGAACCAGTTGGCCTTTAAGTGAGTAATTGTCACTCTCAGACACATAGATGGAAAAACTGAACAGACCATCAGTGTACATCTGAGTTTCGACCATGCGGTTAGTATTCGCCATTCGATAACGGTTCAACTCTTTGGATTCAAACCCTTTCGGAATCCAACCGACCGTCCAAAAGCTTTTCTCTAGCTCGCCTTTTGGCAGGGACAATACGTCGGGCAATTTAACGGTATTCAAACCGCTAAGAATGTCAGAAATCTGATCATTGACGCTATAGGAAATGGTGCGGAACTGTTCCAGCACTTCGCCATCACGATCAACCAGATCAGCTCTTAGTGGAAGCTTAGTTTGCTCATCGATCCACAGGATATAGGAATAGCGTAACCCGTCTTTCGGAGCAATTCTCAATACTTGGCAAGCCGCACCCGCTTCACGCGCTCGACCGACTTGAATAAAGTCGTAAATCTCACTCAGCTCATCAACATTAGAATTTAGAAGCGGGATAGTCGGGGCAACCATGTTACCGGACTCAATGGTAAACGGCTCAACACCAGGCTCAATGTAACTGACCTCATCACCTCGGCGAATCACTTCACGTACAGGTCCACTTAGATAAACAAGGTGAGCGAGTTGCTGACCTTCGCTGCGAGCGTGGCGATACAGCAAAGGTTCGATGCTGTTTTTCTTAATCAGGATATAAGACAGTTCATAATTGAGATGCTGACTGGCTTCGTTCATTTGATGCAACAAAGCCTCTGCAGGTTTTTCTTCTGCAAAGGCTAGTGGCGAACTCAAACTGAACAGTGTCAGCGCACTGATCAGAAATTTTCTCATTCAACTACCGTTTCTGGGTTGTCATTATGCTCTACAGCGTTATCGCTATTGAGGCGAAGCTGTAATTCATAGTCTTGAAGCATGGCGTTAATTCGACGGCGCTGCTCTTGCACGTTAGTTTCACTTGACGCTGTTGGCTGTTCCACCGAAGAACGTGTCAAGCTCACTGGTTCAGCACTGCCTGCAAATGGAATCGTCTGCAGAAC

>NODE_82_length_2965_cov_2.216066

TGATTTTTCCATGCGCAGGTAGGCCAAGCTCACAGCGTGCCTGTGCCTGAGATCCCGGCTTGAATCTTTCACAGTCTATGCCGTTTTTTATTACAGTGATATTGGCATAGTTGAGTTTCTCATCTAATTGCTCTTTTACTAGCCCAGCATCTGCCACCAAATTAGGACGGGATAGCTTTAAAGCAAGGCTTTGCAACAGAAGATGTTTTTGATTGTTAAGATGCCAAGCGTCGTGTTCTGTGTGGACTATGTGTTGAACACCTGCCAGTTTAGCTGCAGTCGCTCCGTAAATTAGAGGCCCAATGTGGTGAGTGTGAACGACTCTAGGTTTAATTTGTTTGAATAGTTTACGCAGTTTAGATATCGCCTCAAAATCAAAACCGGGCTTCTTGGTTAGAAAGATAAGTTTATCTTCAACGTTCTTTAAGCTAGGCCAGTTTGCCAATGTTTCGCAGCGACTTCCTTCGAGACTGATCAGGAAAACGTTCTCGTCTGGTTTAGCAAACTTGAGCATGTTGATGGCTAATGTTTCTAATCCACCTGGTGCCAAATGCTGAACAACATGTATGGTTGTTTTCGGTTGACTCGTCATGCGATTTCCTTGGTGTGCGATGGTTTAAATTAGAAACTGCATAACCTGTGCCAAAAAAAAATCCCTATATAACAGTGTTTTAAGTTTTCATGTGTAAGGTGTTTTATTTTGAGATTCTTTATGATTGAAACGGTTTTATTTTAGGAATTGCAGTGATCACCTGTTTGCCTGTTAGAGACTGCAACTCACTCGCGCTTCGAACCGAAGTGTCAAAAAGCTCAATGAGCGTAGCGATACCGCAGCCTAGGCCAATGCCTGCGACGAACCCTGCGATAATAAATATGACTATGGGTAGGTTAGCTGGTGAACTTGGCGTATAAGGTAAATCAATGATCTTGACTCGCTTGTTTTGCTCAAAGATACCTAGTGAGCCAGTAAGTTGAGCCATCTCATATCTTTGAATCAACTCATCGTAGAGTTGCCTTTTGATTTGAACATCCCTCTGCAACTGGAACATGTCTTTTGCGTTGTCGCCGAAGCGGTTCGCTTGTTGCTCAAGATCTTCAATCATTTTACGCAAGCTTTTGGTTTCTTCACTCAGGGATTCAAACCTTCCCCGCACTAGCTGAAGGCTGTGTAACTGGGTAACGAGCAAAGGTTGAATTTGGCTGATGTCAGACAAAGTGTTGCTACTCGCGATATCCCAAAGTTGGTCGCTGCTGATATTTGGCTGCTCAACTTTAAGAAGTTCCGAACGTTCGTTTTCAAGTCGACGAAGTTCCCGCTGTTTGCCGAGTACAGCGCTATGACTATCAGTATATTTAGCTCTTAGTAAAGTTAGCTCACTGCGAATTTCTATGATTTGATCTTCAATTTTACCAACGACGGGGTTGGTTTTAGACAGTTGTTGATCGAGTGAGCCCAGACTCCGCTCAACCCCAGCAAGTTCCGCCTCTTTCTCGGCTAAGTTTTGCTTCAGGCTAGCAAGCCTCGCAAGACTCTGACTTTGCATTTCAGGTGTAACGGAGGCGTTTTTGTTCTTAAAATCAGCAAGCGCTGTTTCTGCTAAGTCGAGATCTTCTCTTCGCTTATCAATATGCATGGTGAGAAACTCACTTGAATCTTTGATTGAAGAGCGTTCTGGGGCCAGCATTTGCTCTATAAAGTGCTCGCTGACAGATTCTAATAGTTCTTTCATGCCTTGAGGTTGAGTAGAAGAAAGCTGGATTTTAAGGAAATCCTTTCCAGGCTGGGTAACCGTTAAGCTTGCTGCGAGCTCTGCGATGACTTGATCCACTTGAAAAGGGGACATTGAATCGTCAATCAGTCCACGCTCTTTGGCAACGGATCTTAAAATATGTCGACTCTTGAGCAATGTACTCAGAGCTGCCAAACGCTCCTTGAGCATGGTTGAAACTGCGATATCTTCCAGAAACGGGTTCATTTTTGCAGTTTCCTGAATCAGCATACTGGTATGGGAGACGTATTTCTCCGGAGCCATTTTCCCGACGAAGTATCCAGCAAAAGGCATGATCAGCATTGGTAGAACAATTACGTACCTCTGCCTCCAAGCTGCATTAAGGATAATTATAAAGCGCAGTTTCAGTTCATTCATAGTGACTCCAGAAGCTGATTGACAACTCGACTTCGAGCATCCCAGCTGTCGCGACGAACAATCTCGGTATCAGTGTTTTCTATACTGAGAACCTGATTGAGTGATTGGCTAAACTGTCTCGCATCGTTGGCAATACTGACGTGTGATCGATAAGGAGCAAGTGCTGGAAACGGCGTTGTAACAACAGGTGTTCCGGCTGCTAGATACTCCATCAGTTTTAAAGGGCTACAGGCTTGAATCTGATCATTGAACAAGAAAGGCAGCATGCTCGCATCCCAGTGTTGACTGTATCGAGGCAACTCTCGGTGTGGTTTGGGGCCTAAGTAAAATACATTTGGAAAATTCGGCAAAGGATTATGATCTAGTTCCAATGGCCCGATAAAAACGAAGTTCCAGTGAGGATTATCCAAACAGACTTGAGCTATCAATTTATAGTCCAGCCATTGGGATATGCTGCCGTAAAAACCAGCAATAGGCTTACCGTCTAAGGGAAGGTCTCGAGCACGGCTTATTGAGGTGTTAAACAATTCATAGTCTACGCCATGAGGGATATGCGCGGTTTTTAATCCGTCAAACTTTTTGCAGATTTCCTCGCTTGCACCCAAGATAAGGTGAGCTTTTTCAGCTAGCTTTCTCTCATGATTACTCACTGTTTCATGATCAACACCAGCAAGCGAGTTGAAATCGTCTCCACAGTAATAAACAACCGCAGCTTCATCTAGGTTGCCACATAAATCCGCAGCAGTCGGAAGAGAAGTCCACAAAATCGGTTTTGTTAGCCCCAGCTTTTCGATGATGGGCTTGAGTTGAGCTAATAG

>NODE_83_length_2946_cov_2.355176

ACACTGTATAGCCTTGCCGGGTGAGAAGGCAGTGTTTCTCAATCCATATACATCTCGAAAGCAAATTGATGAGTATTGCCGCTTATTCGTATAAGGACCCATAGAACAGCTGTTTTAATCACTACGTGGTAACGGATGAACTAATCTTTATATATGAAGATTTGTTCGTCTGTTGCTATGAAGATAAACGCTGTATCGATTTTCTGCGGCTTAACCTACTGTTGTTCATCTAGTGTTGGTGCTGAAACCGTCCGGCTGCTTGAGCTTGACTGGTCAAGTCAGCGTGTCCTCACACACGTGTTGGCAGAGATATTACAGTCAAAGCACGTCGATACTGAAGTCATCACCATTCCTTCAACACCACAATGGATGTACTTGAGTACAGGGAAAGCGGATATTCAGGTAGAGGTTTGGGAAGGGTCAATGGGGCCACAATACCAAGAATTGCTGGACAAATCTTTGATTGAAGAGGGAACAACCCATCAAGCAAAGACCCGAGAGGAATGGTGGTATCCAGACTATGTTGAATCTTTGTGCCCCGGTCTACCTGATTGGGAGGCGCTGGAGTCGTGTTTTTCTGTATTTTCTGAAGAGGGTGAGCAAGTCGGTACTTTTTATACGGGGCCTTGGGAGAAACCCGACAACGCTAGAATACGTGCCCTTAATCTCAACTTTAAAGTCGTTGTTCTACCAACCGGGCAAGCAATTAATCAGAAAATCCATCAATATGTTAAAGACAAAAAACCTCTGCTTATCTTTAATTGGACGCCCAACTGGGTTGAGTCTGTGTATTCGGGAAGCTTTATTGAGTTCCCTACCTATACACATGAGTGTGAAAAAGATCCTTCTTGGGGATCAAATCCTAAGTATTTGTGGGATTGTGGTAATCCATCGGGTGGTTGGCTGAAGATAGCGATTTCCAATCAGCTTAAAAGCCGTTCTCAATGCGCTTACGACGTAGTTCAACGCTTCCAACTTAACAATAGAGATATCGCGTTGGCGGCTACATTAGTCGATGTCGAACATTTTGATGTTAAAGGGGCTGCGAAGACTTGGATAGAACGTAACTCAGAGTACGTTGAACAGCTATTGATGTCGGTTAATTGCCAATAAACATTTCTTTCCGTCAATCTTGATAATGGAACGTAATACACTTGTTTCGCCCACTCTGCTTCGCTTGGTATAGGGCTTTATCTGCTTTTCTAAAAACACGATCCCAATCCGTTTCAATGTCTTGGTAATGGCACAGCTGAGTTCCGACTGAAATGGTTACAGCAAGGCTATCTTCATCGATATGAATCTTATGACTTTCAACGCTCTTTCTAAGCCTTTCTAAGGTTTCTTCAACTGTATGTACGTTATCGATAGGAATGATCATGACAAACTCTTCACCGCCCCAGCGAGCGATAATGTCATGGCTGCGAGTATTGTTCTTTAACTGATTCGCCGTTGCTTTGAGTACAGTATCACCCACGTCATGGCCGTGAGTGTCGTTGAATTGCTTGAAGTGATCAATGTCGATCACACTCAGAGCATAAGACTTATGTTGTTGCACATTATCAGACTGCGACAACCAGTTTTCGATATAGCGACGGTTAGCTAAGCCAGTGAGTGGATCCGTGGTGTTTTGACTCTCTAGCTCTTTAGTAATTCGCTTCATCTTCCACAGGCGTCGAATGACAAGATATGCTAATGCGAAAAACAGGGTAATAAATAGAATCTCACGGGTACGTTGAGACCGCAGCTTGGTTTGCTGCAATTCATTTTGAGCCTCAAGCAACTCTATTTCCCGTTTATTTAATTGCCTTTCCAGAGCATTTTTCTCACTCAGAATCTCGTCGTCGTATGTCTGCCGATAGAAGTTTTGACGTACCTGTGATTGTTGTTTCATGTAAGCCAAAGCTTGTTGATAATCTCCTTGCGCCTCATAAGCTTTGACCAATGTCTCCAGAGTTTGAATTTCCCAAACCACTTCGTTGCTTTCTGAGGTATGCAGTACTTCGATGACTTGTTTGGCGTAGTCGATGCTTTTTTGATAGTCACGAGCGACAAAACTGGCCTCGGCATAGGCTTCATAACATGTGGCTAAGGTGATGTTTGACTTGGTATCGAGAACCTGGCTTTCTTCAATACATTTTCTGGCGAGTTTTAAAGCTTCTTGTGAGTTTCCAACGTCACTGTAACGCTGTGACATATTTGATAATGTTGAAAACTTAATCTTGTTTGACCCAGTTTTGTCGGCCTCCTTGAGTGCTTTCAACATGTATTGTTCCGAGCGCTCCTCTTTGCCCAACGCAAAATAGATGGCACTGGCATTTGAGTACATAATGGCTTCAAACAAGCTATTGTCATAACCGTGTTTTTGATAAAGATCCAATGCACGCTTGATGTATTTATTAGCACGTTTCCATTCTTCAGACTGCATGTAGACAGTGGCAATATTATTGAGCGTCATTGTGGTCTGAAAGTTGTCTCCAATCTCTGTCATCACCTCATATGCGGTTTTGTAATAACTGAGACTGGACTTAAGATTCGAACGCTGAATCTTAACGTTACCCAACCAACGATAGGTACGACCCAATAGCCGCTTATAACCAATGTCTCTCGCAATCGGGACGACTTCATGCAGTAAGAATTCCCCTTCCAAAAGTAGATCAGACTCGATTAAGTGAATAGCTTTGTTGAGTGTTGCTTCTGCACTGACCCATTCAATACGACTGTCGTCGCCGAGTGAAAGCAAATCGGGTATGCGAGGCGTTTGTAATCCTTCATATTTACGAAGATTTTTGAGGCCAAAGTAATAAAGAGCCTGCTCAGACGCAGACGCGTTTGGCGGCAATTCAATATGTGAAAGGTCGACTTGTCCTGGTTTACAAAGTGAGTCGTAGTAAAAGGTCAGCATTGCCTTTTGCTTGGCGTTCGTATGTGAGTAGAGATAATCGCTAT

>NODE_84_length_2946_cov_1.950505

AAATAAGGCAATTCAGAGGCTTTTTGTTCATTAACCGCTTCAGTATCTTTCCCTGAAACTTCGGTCGCTCTCTCTGATTTCTTCATTTCAGGGATTTCTGTATCTGCACTAGAAGAAGCAGCACCGAGAGAATTTATTGCAGAAAGATCGACCCCCATTTGCCGAGCAAGCTGTTCTTGTTGGGCTTTAGGGAGAGATTGGAACTGATTGATCTGATCTTGAGTTGGCGTTGCTGCGTTGACACTCGCACTTAGGAGTATTGAGGCAGCCAACATATGTATATTGAACTTCAAAGTTGTGATACTCATTAAATTATATAATCTACTGTTCTCTGGTGTTAGCGACACAATCAAAACCTAAATTCATACCTAGAACCAAATACCATCTCACTGTCATTGGTGTCTTTATTGATATAATCTGCGCTAAGCTTAATTCTTCCTTGGAATGCAGGAAGCTGGTAAGCCAGTTGATATTGTCTCTGCCTGTCATTTTTGTCATTCAATAATTTTTGCGTCGAAACAATCGCAGAAACCTGATGATCATTGACTAGTTGAAGATAACTACTCAACGACCAACTACTTTCATTCAGGTAACTAGCCTGCAAGTAATTATCAATTCCTTCATCCACAATTAATTTGCGCTCTTTCGAAGTCATTGACTGATATTCGACCGCTACACGAAATGAACTGTTGAATATAGGCTTATGTCCACTCCAACCTATCAGGTAAGCACCTAAACTATTTTCCTTTTGAGCTGAGGCTACTTCAGCATACAGCCCATGCTGCACATTATATACTTTTGGTAAAGTTAGCTTTACATCGACAGCGGATTGCTTCTGATTTTTTGCTACGTAATTCGTATTTGCTTCAAACCAATAATGCTGAGTAAAACCGAGCTCTAACCAAGAATTCAGTTTACCAGCATATCTCATAGAGGTTCTGTATTCATAATCGAGATCCTCTAACTTTGAGGTCACAATATTTACACTCCACACTCCAAGAAAATGATTGTCTCCGATCCAGTTCAGACCAGCATCTAAATCATTTCCTGACGTTGCTAAAATGAGACTGTGATTCCAACCGTGCCCCCACCATCTAGGTAGCATACTTATATCAAGTAACGCGCGGCCAGCATTCAGTGCAATGTAACTGTCTTTAAAAGAAAACTCTTGATCATTAGCATGCTCTAGGTAATAAGAAGAGTTGAGCCTATACGCATAACTGTTAGCTAGGTATTCATAGCTCGCTTCTACAGCCCATTTATCACGCTTTATATCCGCAAACCAACCATTGTCTAACTCTTTATTACCACCTGATAATGCGAAGCGACGATTACCACGATTAAGTTTAGCCGTCTGAAGAGCGTAATTAAAGTGAGAAAATTCGACATTTAAGCCACTAGATATATCGACGAAACTTTTATCTCCTGCTGCAGATGACCAACGTAATGGAAATGTGCTAGTTACGCCGTTAAGCACTGCTCCATCACTTAGCGCGACCATAGATGACCTTAAAAAAGGATCACTTGCTTCGATCCAAGGAGAAGAATAGGCATAATTACTAATTAACATAATAGATGTAAATGGTACAAATAACCTAAATATTTCAATCCTTGGCATGTATCCTACTTATGTATGTCTCTGAATCGATAAACAATACGAGCAAATTCAGAACCGATTTCCTTAGTTTTAGCTCATTATAAAGGGCGATATAGATTAAGCAATCCACCATATTGTGATCACCTTGCATTAAAGAAATTTAAATTTTTACAGATATAAAAAAGTGAAATAGAATCAAAACTGGAACAAAATAATTTGTTCCTACATCGATGTTAAATTGTAAATTTACAAGGAACACTCAATGAAGAAAATTGCTCTAACGATGTTAGTTGCGTTATGCGCGTCTGCTGGTGCTTTCGCTGAGGAAAGTACCGCCGCTAGCACTGCTGGCGGTTCAACTGCTGGTGGTACTGCTGCAGGTTCTACTGCTGCAGCCTCAACAGCAGCGGCAGGTGTAGCGACAACAACAGCAGTAGCAGTTGGCGCAGCTGTTACTGTTGCCGCTGTTGCCGTTGCTGCATCAGATAATGATGGAACAGACGGTACAACTAATTAATTTAGTTTAACCAACAAACACCCTTAGCTCCGCTAAGGGTGTTTTTTTGCGATATTTATTAGTTCGAGCTTTCATCATGACAAATAAACTACTTGCCCTATCTATAGTTGCTCTTTCTGCCTTTTTAACTGGGTGCACACAACGTTTTTCAGACACTAATGACACACTAAAAGAAGCGTTCTGGGGCTTTGAGGACGTAATTTTAAATAAAGAAACAATCGAAAAAATACCCTATGCTAGCAGCTATTTTAAGATTAATGATGGGCCACAAATTTTTATGGTGCTTGCATTCGTTGAAAAAAATCCACAATCTGGAAAAACTCAGCATAAATGGCTCTCCAGTGACAAAGCGATGATTGTTACCGAAGAAGGTCGTATCGTCAGGACTTATAACCTCCCAGAGGCAAACCTAGCTGGCAAAATATCGCCCAATAGCATCATACATTTTAACACTCCTCAGTATCACTGGACTTCCGTATACGATTGGCAACCTGAATACCAATATAATCACCAAGCAAAAGTCACCACAGAGTCTGTGGCAACAATTAAATTAGACTCTGTGATGTGGTCTCAAAAGGTCAAAATCTGGCATGAGTATATTTCATTTGAAAGATTGGGTGAAACCATGCAAAGCACTTTTTGGGTGAATGACAATGGAGATGTATTGAAATCAGCCCAATGGGTCATTCCAGACCAACTTTTCATCGAGCAAGAAATACTCAAGCCATTTAAGGGGTGATCTAGATGACTCGTAGCTTTTTATTTCTTTTCTCTCTAGGTGTTTTCAGTGACGCTATTGCTAACACCGTAAAATTAGAGCTTCCAAGTGAGC

>NODE_85_length_2941_cov_2.329609

CGATAGAATTAACCATACGATCCAGGCTCTCATTCACATTCTCCGTCGTCTCAGCAGTTTCCTGACAACGCTGCTTAGTGTCGTCCATCGCTTTAACAACCGTTCTGGTACCCTCGTTAAGGTTATCCAGCATATTGTTGATCTCAGAGGTGCTTTGTTGAGTTCGGGCTGCCAAAGCGCGAACCTCGTCCGCAACCACTGCGAAGCCACGCCCTTGCTCACCTGCTCGCGCCGCCTCAATCGCCGCGTTCAGTGCCAACAGATTGGTTTGATCAGCTATCTCACCGATCACCATCAACACAGAGCCTATCTTCTGCGAGTCTTCGTTCATAGCCTGAATACTTTTCGCCATCGACTCAACTTCTTCGATCAGGTCAGCAACACTGCGAACGGCACTGGCAACGACCAGTTTCGACTGCTCGGCTTCATCATTGGTTACACGAGTAAACTCCGCGGTTTGAGATGCACTTTGTGCAACGCTTTCGGCTGTTGAACTCATTTCATTCATCGCCGTTACCACTTGTGTGGTTTCCATCGCATGAGATGTCAGCACTTGAGCATTGCTGTCGGTCTGCGATTTTAATAGATCAATGTTGCCTGAAATTTGATTGGACGATTTCGACACTTCCAGCATCATTGATTGCAGGTTACCAATGAAGGTATTCACTGCCTGAGCTATCTGGCCTAAGTCATCGTCAGTTTTGACTTCAAGACGACGTGTTAAGTCGCCATTGCCCTGAGACAGATCGAGGACAGTACTCTTTAACGCTAGAATTGGACGATAGGCATAATTGAGAATGACGAACAAGATGATAAAAGCGACAACGAGTAACACCAGTGCTGTATAGAGTGAATCTTTCAGTGCTTCATCAACTTCGGCATAAGCAACGGATTTATTCACCCCGACCAACAAATGCCAGCTGTTGTTCTCATCCAATTGAATTGTCTGGAAGAAAGCGACCTTCTCGATACCTCCTAAACTATAATCCTTCATCCCTGTCGGCTGAGACAGCAAAGCGTCGGTCAAAGGATTTAATTCAGCAAAGTCATACAATTTCGTTTCACCGGGAACATCCACTTCGCCGTTTGAGGCAATTGTCAGACTGTTGTGGTCATATAGACCCATCATCGCACCCGGGAAAGGCGCTTTTCTGACCAGCTCATCCAATAAATTCAATTCGATATCGGCTAGCAAGACCCCTTTTAGCTGGCTTTGCTCGTAAAAAGGTTCAGCAACACTTACCATCAAACTTTTGGTCAAAGCATCACTGTAGATTTCCGTGACGATCGTGCCACGCTTTTGCTTGGCAAGTTGGTACCAGTCGCGTTTACGTGGGTCATACTTGGCTAAGTCCCGCTTACCGCTGTTATTGCCATAAGAGCGGCCATCTTCGTAACCAACCAGAATATCCGATGCAGGAGAGGTGTTTGCGATCTGTCTGATCATCAATAAGATTTGGTCATCGTTGTAGTCTGGAACAAAATCAGGCGCGTTCGCCACCAGCCCAGACTTAATTGAGTCAATCCAAGTTAAGATCGTATCGCTGGTTGTACTGATTTTTAGCATCGAGTATTCATTAACCCGCTCGTGAATCGACGAAGAAATTTGTCTGTAAGAAAGGAAGCTAGAAACCGTAAGTGATGCTGTCAGTAAGAGAATGACAACAGCAACGATCCTGCCCTTAAAACTGTTTAAAACACTCATATCCTTTACCACTGTTTCATAAGTGAGACGAATGTATCATCGCAATTGCATAATGCTCCTTGTTTTCATCTAAATTTTTACGCCGATCACACTATTAATAAAGTTAACAACATCTTTCTAAGTCATTGTAAGTATTGATAGTTAATAGAAGATTAATCGTATTCGAGCCCGAAAAAATACCGTTTATCGTTCCTCGATGTTTCATGACAACTGAAGCCAGCTAAATAAGGCTTCAGCGCTTTTAGTCACCAATATGTTCAACAAACGATCTCAACGATACATAGAACCTTTAGCTATTGAATATGCAGCAATTAGCTTTGCGTAAGTTGCCGTTGATCACTACTATCAATTTGAACGATCGTTCAACAGGGTATATGATTCACCAGAACAATAATTAATCCAAACAGAGGTTGCAGATGTATCTAGCTCAGTTTTTTAAAACCGCAGGCGAACCCGCGATACTAAAACGCTCGTTAAAGGTTTCTCTTATAGTTGGCACTATTCTTATGTTCATTAATCACGGCGACAAATTGCTCTCCAGCAGCATCGACGCAACACTGGTTATCAAGATCTTAATGACTTACTGCGTTCCTTTTTGTGTATCTACCCAAGCAAGCGTTTCTGCAACTCTACAATCACGAAACAAGGCCGCCTAATCATGAAATTTTCCTTTAAAAAACAGTCTCCAGATATTGTTGAGGTCAAAACCAGTCACCTCGAAGAATTAGAGCAAATCTCTGATAAGTACTCGCTATTCAAACAGAATGATCCCACCAACTATGCCAAGAAAATTCACGTCAATGCGGCCAATGTCCATAAAGCGTCACGAAGTCGCGCTGAATCTATCGAGCAAAGCTACGAACTGGTCAATCACTTTATCGAGCAATCGGAAAGCATTAATAAACTGTCTTGTGAGAGTCACCAATATTCCAGCAAAACGGCTGAAACTGCAGCCAATACCATCGATAAGCTGGAACTTTTGGAAGAACAGGTGCTGGTTTCTAAGGAAAAGATCTGTCAGTTCAGTGACCTTCTTGAATCATTAGATGAGATAAACAAGAACGTCACCCAATTGGTGGATTCCATCAAAGGCATTGCGGCACAGACAAACTTGTTAGCGCTTAATGCCGCGATTGAAGCGGCTCGAGCTGGAGAGCACGGTCGCGGTTTTGCGGTAGTCGCAGACGAAGTCCGTCAGTTAGCGAACACAGCGAACCAGTCAGCAGAGAGTATCGA

>NODE_86_length_2938_cov_2.728766

CGATTCGCCAAGTTGTACTCTTCAGCCTGCGTATCGTCAGCAAGGCCAAGCACTGACTCCACATCATAAACTCGCTTGCCTAGGGTATGGATTTGATCGCGCTTTTCTTCCAGCTCTTGAGACAAAGTATGAGTTTCATTGACCTGCTGTTGGTATTGTTGCTCGGCTTGCTCAAGTTGGTTGCGCAGTTGAACGACTTGAGATTGAGAAGAGTTCTGCTGCTGCCAGCTGTAATAGACGACAGTCGCGCTGGAGGCAATCGCACTGATTGAACCAGCAATAGTCAGAAGAAGGGCTTTTCTTGAAACGTGAAAGTGTTGCTCCCCAGATGAAGAGGGGATAGAAATTATTATCTTTTTGGACATACTGCTTAGTTAGGTGTATTCACCTAAATCTGACAGGTGCTCAATGTCTCTGTGTAGGAGTTCATCGTCACCGACATTCAATTCAATTAACCGCTTCAGATGTCCGATACTTTCAATGTCAATATGATCAATACTCAGACGGATCACATTGTTATTGAGGCCCACAATTTTGCCGACCAGCTCAATCGTGATATCGCTGTCTTGAAGCGAAAACTCTACATCTACCATACTGTCGACATCAAGCGAGTCTGCGTCATCTGACGTCAGCAGCAGTCCATGCAGGGATAAATCACACAAAGAGGATTTCACCACTTTGTTTTCTTGAGATAGCAACGCTTGTGTCTGGTAAACAATGCGCGAAAAGCGACGGCGTTCGATCATAGTTGTCTCTCTTAATGGTATCCAGTAAGGCTATCAAATCAGAAGTGACTGCTATACGCGTCGGTTCGCATTATTGATTCAAAACACGGTAACAACCATAATTATTGTGAACCAATATACAATAAAGGCCGCTAATATTAGCGGCCTTTGCACAAATTTCAAGCAAGATTACTTAGTAATACGCTTGTATTTGATTCGGTGCGGTTCCGCTGCTTCTGGACCTAAGGTCTTCTTCAGCCACTCCATATACTCAGTATAGTTACCTTCGTAGAAGTTCACCTGACCTTCATCACGGTAGTCGATGATGTGCGTTGCGATACGGTCTAGGAACCAACGGTCGTGCGAGATAACCATGGCACAACCAGGGAACTCCAGTAGTGCTTCTTCTAGTGCACGCAACGTTTCAACATCAAGGTCGTTGGTTGGTTCATCGAGTAGCAGTACGTTGCCGCCCGCTTTAAGCAGTTTAGCAAGGTGCACACGGTTGCGTTCACCACCTGATAGTTCACCGATGATCTTTTGCTGGTCTGAGCCTTTGAAGTTAAAGCGAGAACAATATGCACGTGCAGGAATTTCAAAGTTGTTGATCTTGATGATATCTGCACCTTCAGAGATTTCCTGGAAGACAGTCTTGCTGTCATCCATGCTGTCACGGAACTGGTCGACAGACGCCAGTTTGACCGTATCACCCATCTCAATGGAGCCCGAATCTGGCTGTTCTGTGCCACTCAGCATCTTGAAGAGGGTAGATTTACCTGCACCGTTGGCACCAATGATACCGACGATGGCGCCTTTTGGCATACTGAATGAAAGATCATCAATAAGAACACGGCCGTCGAACGATTTAGTCAGGTTATTCACTTCAATAACCTTATCGCCTAGACGCTCACCTGGCGGAATGAACAGCTCGTTAGTCTCATTACGTTTCTGGTGATCACCGCTTTGAAGTTCTTCAAAACGTGCCATACGTGCTTTTGATTTCGCCTGACGACCTTTCGGATTCTGACGAACCCATTCCAGTTCTTTCTCAATGGTTTTCTGACGAGCTTTTTCCTGAGACGCTTCTTGTTGTAGACGCGCGTCTTTTTGCTCTAGCCATGAGGTGTAGTTACCTTGCCATGGGATACCTTCACCACGGTCAAGTTCTAGAATCCAACCTGCAGCATTGTCTAGGAAGTAACGGTCGTGCGTGATAGCAACGACAGTACCAGTATAGTCAACCAGGAATCGCTCAAGCCAAGCGACTGATTCCGCATCCAAGTGGTTGGTTGGCTCGTCGAGAAGCAACATATCTGGCTTCTCAAGTAGCAGACGACAGATAGCCACACGGCGACGCTCACCACCAGACAGGTGCTCGATCTTTTGATCCCACTCAGGAAGACGCAGTGCATCTGCCGCACGCTCAAGCGCGTTATCTAGGTTGTGGCCATCTTTAGCTTGAATCAGTGCTTCCAGCTCGCCTTGTTCTTTTGCAAGCGCATCGAAATCAGCATCTGGTTCTGCGTACGCAGCGTATACTTCGTCAAGACGCTTCATTGCACCCGCAACATCGGATACCGCTTCTTCAACGATTTCACGTACGGTCTTAGACTCGTCTAATACAGGCTCCTGCGGTAGGTAACCGACATTCAGACCTGGTTGTGGACGAGCTTCACCATCGATATCAGTGTCAATACCCGCCATGATACGCAGTAGGGTCGATTTACCCGCACCGTTCAGACCAAGAACACCGATCTTGGCACCAGGGAAAAAGCTTAGCGAGATGTCTTTCAGAATTTGACGCTTAGGTGGCACGATTTTGCTCACCCGCGACATGGTATATACGTATTCAGCCATTGCCGATCGTTCCTAAAATTTCAATTCACGTAGAGAGGCATTTTATACCAAGATGGGGGGCTTTGTTACCTTCGAAATGAGGATGATGAGGAACTAATTGGCCTTATTGATGGATAAGTGAACAATTCATTTATAGCGATGCCACTTATGTCACAGTTGTGTGAATTTATTATTATTTACATCTATACGCTTTACATGGCTGCTTACAATAAGCATAAATTAATAAACACACTAATTATTCTCAACAGGACGAGAATTTATGATGCTGACGTTTTCAAAGTCGCTGCATCGCATTCCTTCATCGATTCTGACCTGGGTCTCGATGGTTTGCAGCGCAAGCTTAGCCACTTCAGCAA

>NODE_87_length_2932_cov_2.190893

CTTCATTACTGCATGAAATAGGCAGGCTGAGAGAGAATAACTTTCGCAAAGTGGGTGAAGGTACAGGCCTCGCTTTAGACATCGACGAATTTGATCGTTACTACAAGCATTTGTTTATCTGGGACAGAGATAATGAATGCCTAGTTGGCGCCTATCGTCTTGGGTTAATCGATAAGCTTATGCAGAACAAGGGGTTGTCCGGCCTTTATTCGAGAACCCTTTTTCATTATGGCGAAGACTTTCTCAACACCATGGGCAAATCGATTGAAATGGGTCGCTCGGTGATTGATGAACGTTATCAAAAAAGTATGGGCGCTCTGCTATTGCTCTGGAAAGGGATCGCCACTTTCGTCCACCGCAACCCAAGCTATACGCATTTGTTTGGCCCTGTCAGTATCAGTAACGATTACAGTGAGCAAGCAAGACAACTGCTTGCCGATACCATGACTCTCCATCATTACGACTCAGAGAAAGCCGAATATGTGACACCATCGAACCCTCTGCCAGTTCAACCTAAAAATTGGAATACTAGCATGCTGACCGCGTTGGGCGATCTCCAGCTATTGTCGCGAGTGATTGCTCGTATTGATGACGGCAAAGGGGTTCCCGTATTGTTGCGTCAATATTTGGGCCTGAATGGTAAATTAGTGTCGTTCAATGTCGATCCTTCTTTTAACAATGCATTAGATGGGTTGATCATGGTTGATCTTCAGCACGTACCAGTCAAAACACTAGCCCGTTACATGGGCACTTCTCAAGCCCAGCAGTACCTAGCGCATCACACCCCTTAACTTGGTTGAACAACAACGAAAATGCTGCGTGACTTACGCAGCACTTTTGCTATCCAGAGAATTCACTTGATTTAAAATAAATTTCAAAGCGATCTTTCACACCGTAACTCAGCAACAAAGCAACAATCTTTCATAGCTGCTAAAAAGCCGACTTTTATATCGATTGACCAAGATTATGCCAATCAAAAATCCATTGACGGAACTATCATCTAACTGAAATCTAGGGGTAACGATAATCACAATAACAAGGAAGTAACCTCCATCATGATGCCATCTCATAAAACCATTCGCTCTACAGTTACTGGCGCCATCGCGTTGCTATTAGGTTTAGCGTCTGTCACCAGTTTCGCCGCAGAAAAAGTCTATCGACTCAAGCTAGCTGAAACTTGGGGACCAAATACTCCAATTCTAGGTGATGCCACGAAGAACATGGCTCAGTTAGCAGAGGAGATGTCTAACGGACGCCTTAAGATTCGCATAGACTCTGCCAATAAACACAAAGCGCCATTGGGCATTTTCGATATGGTCAAGTCAGGTCAGTACGACCTTGGGCACTCCAGTTCGTATTATTGGAAAGGTAAAGTACCTAACACACTGTATTTCTCTTCCATGCCCTTCGGGATGATCACGACCGAGCAATACGCTTGGTTTTATCGTGGTGGTGGTATGGAGTTGATGGAAAAAGTCTATGCACCGCATAACTTACTTTCCTTCCCTGGTGGCAACTCCGATATTCAGATGGGCGGCTGGTTTAAAAAAGAGATCAATTCCGTTGAAGACCTAAAAGGTTTAAAAATTCGCATTCCTGGATTTGCCGGTGAAGTGATGGCCAAGGTTGGAGCAAAACCAACCAACATAGCTCCTGGCGAACTGTATACCTCACTTGAACGAGGCACAATTGATGCGCTTGAATGGGTAGGCCCTGCTTTCGATTTGCGTATGGGCTTTCAAAAAATCGCCCCCTACTATTACACAGCGTGGCACGAGCCTGGCTCTGAAACTCAATTCCTAGTCAATAAAAAAACGTGGGACAGACTACCTAAAGATCTTCAATCGATCCTAGAAACAGCATTCAGGGTAGCGGCATTTGATATGTACACACAGGCACTCGATGCTAATGCCAACAGCTGGGCGAGCATGAGCGCTGAATATCCCAACATTAAAGTTCGAGATTTTCCACCGGAAGTGCTCAGCGCAATGCAAACCGCGACTCAAGAACTGTTAGAAGAGCTAGCGAACAAAGATGAACTAGCAAAGGAGATCATCAGCTCTCAGAAACAGTACCTGACTAAGGTGCGTGCCTGGACCAATATTTCTTCCAAAGCTTATCTAGATAGCAATTAAGCCTTTTGTGGGTGGCAAAATGTCCTTGCCACCCATTTGAAAGCACATCAATAACCTGCCGTTTTCCTCTAGAACTTACTGTTGCTTTATTTCACCAAAGCAAAAATGAGATATTTATCACACCAAAAGTAAAGATCATCCACCCTATCAATCAGGCAAAGATCACAAAATTATAATATTCGATAATTCAACGCCAATTATTAGTGATTTAAATCACAAAATATCAACCAATTAAAAACAAGGAAATGTGAGTATGATCATGTGATTTTTATTGACATATGAGTTGTTGACAATTTGTATGAAAATTACACGCCAATTACTTGATAGAGATCGCTATAAGAAAGCGTTATTGGCAGGATTATTTGCATGACGTCACAAAAAACCCGTCGTCAGTTCCAAAATGAATTTGTGTGGTAAATTGATTGGGTACTAAGCAATCAAGCGGTTTTGGCGAACCGTACTACTACAATATTCATCAGAACATCGAACGGGGAACTGTTATGATATCACCAGACGCTAAGATCAAGATACAAAATTTTGGTCGTTTTCTGTCTAATATGGTAATGCCCAATATCGGCGCGTTTATCGCGTGGGGTTTTATTACTGCTCTATTCATTCCAACAGGCTGGCTACCCAACGAGACGTTAGCGTCTATGGTCGGCCCAATGATTACCTACCTATTACCACTGTTAATCGGTTATACCGGCGGTAAACTTGTCGGTGGCGAACGCGGTGCGGTTGTCGGTGCAATCACCACTATGGGTGTGATTGTGGGTACCGATATACCTATGTTCATG

>NODE_88_length_2929_cov_2.547686

GTGTTGGATCCGCATGATGTCCATGACTCTCTACCATTTGGCGGCTAAGGCGATCTAAACACAAATAGTTTGCTCGGCCTTTAAGTAACGCCACTTGCCCGTAAAAGCCCAAGGCATCGGTCATTAAAGGTAAATCACGATGGAACAACTGTTCCTGCAGGTTTTTCGAGCCCGTGCTGATGATGGTTTTTTTGTCGCTCAGCAAAGCTGGAACCAAATAAGCGAAGGTTTTCCCCGTTCCGGTTCCTGCTTCAATGACCACTTGCTGGTCACTTTTTATCGCTTTAACCACAGATTCAGCCATATCTAGCTGAGCCTGTCGTGGTTGGAACCCAGGAATCGCTTTACCTAGAGCACCATCTGCTGAGAACGTTTTAGAGATCATCAAAGGCTGTAATTTGAGTTGAACAAGATTAGGAACGAGGGATTATGGCAGGTTTAAAGACGTGGCGCGAATGCAAATCTGATCCGCGCCGATAGGTCACTTTATTGATGAGTAAATTGTGTTAACGACGGTTGAGATAACGCGATAGCCAAGCGGGACCAGCAAACTCATTCTGACTATCCATCATGGCTCGAGCTGCTTCACTGGGCGACGCTTTGTTTTCCCACATTTCATTGAGAAGCTCTTCATTGATTTCGTGGTCACCTAGTAGCGCACTGACTCTTTCGGAATACAACTTACGTGCTAGTAGATCTTTATCCATACGTACCACCTGTCGGGCTAAAATCTGACAATTTTTAAACAAATTCGCCAAGCTAATCGGTTAACTCGGATTAATTGTTGAAACCATCTTGATATTAGTTTAATTGTAGCTTCCATATTGTGGGGCGCTTCACCGTTCAACGCCCGACAAAAATTTCAGAATACGAAAAAAATAATTCACGGAAGTTTTAACCCAATGGAAAAGAAAACACTGCTCACTCATTGTACAGATGCACCTGGCTTGATTTCTAAGATCACCAATATCTGTTACAAGCACCAACTCAACATCATCCACAATAATGAATTTGTCGATAATACCAGCGGCCATTTCTTCATGCGTACTGAGCTGGAAGGTTACTTCAATGATCAAACCTTCCTTGCCGATCTCGACCAGGCTCTGCCTCAAGGCGCTAAACGAAAGTTGATTGGCCCAAACCGTAAACGCATTGTCATTCTCGTCACCAAAGAAGCGCACTGTATTGGTGATATCCTAATGAAAAACTACGACGGCAGTCTGGATGTCGATATTGCGGCAGTGGTGGGCAACTATGATACGCTACAGAGCCTGACTGAAAAGTTTGATATTCCATATCATCATGTGTCTCACGAAGGGCTAAACCGTGAAGAACATGAACAGAAGATGCTTGAAGTCATCGATCAGTACAATGCGGACTATCTGGTTCTGGCTAAATACATGCGCGTACTAACACCATCATTCGTCGAAAAGTATCACCATAAGATCATTAATATCCACCATAGCTTCCTGCCCGCTTTCATTGGGGCGAAGCCTTACCAGCAAGCTTACGAGCGAGGCGTTAAGATCATTGGTGCAACAGCGCACTTCGTCACTAATGACCTCGACGAAGGGCCAATCATCAAGCAAGACGTGATTCCGGTCGATCATAACTTTAGCGCGAAAGATATGGCACAAGCAGGACGAGATGTTGAGAAGAATGTACTCAGCAAAGCACTCAATAAGGTGATAAACGACCACGTATTTGTCTATGGCAACAAGACTGTGATTCTTTAATCTTGTATCTAAATGCAAAAAGACCTCCCATTGGAGGTCTTTTTAATTTCAGGACTTTATTCGAACCTACTTAAAGCTTCGCAGCCAGTTCAACACCTTGTCTGATAGCACGAACAGCATCCAACTCACCGGCATGATCGGCGCCTCCTATGATATGAAGCTTGTCACCAAACTGCTGCCACTGGTCCTCAAAAGGTCTCACCGACTCCTGACCCGCACAGATGATTACTTTATCCACTTCAAGTAACTCAGATTTGCCATCATGGCTGATGTGCAGACCAGTATCATCAATCTTGTCGTAGCTAACACCACCCACCAGATTCACGCCACGCTTCTCAAGCGTACGTTTATGAATCCAACCCGTGGTTTTACCCGGCCCTTTACCAACTTTGCCCTTGCGGCGCTGCAGAACCCAAACATCTTTTTCGCTTAGGGATTCAGGATAAGGGTACAAACCGCCCGGATGCGCGATCTCTTTATCAATTCCCCACTCATGCAGCCAGTCGTCTAAATTGTGACCAACTGGCTCTGTCAGCATGGTTGCTACATCCACACCAATGCCACCCGCACCAACAATCGCAACTTTCTCACCAACGTAAGTTTTCTCTTTAATTAGCGTCTGATAATCAATCACTTTTTCTGGGTTGTCGATCCCGTCAATCTTGACTTTACGCGGCTCCACGCCCGATGCCATGACTACTTCATCATACTCGACCAACAGATCAAAGTTGGCATCCGTTTCAAGATGTAGCTTCACGCCTGTTTCATCAATCTGATTGGCAAAATAGCGAATGGTCTCGCGGAATTCCTCTTTACCCGGAATCTGCATGGCTAAGCGGAACTGCCCACCAATTCGGTCATTGCGCTCAAACAGATCAACATGGTGGCCTCGTTTCGCCAGAGTCGTCGCACATGAAAGGCCAGCTGGACCAGCTCCAACTACAGCGATATTCTTCGGCTTATCGGTAGGTGTAATTTTCAGTTCATCTTCACGACAGGCAAGCGGGTTGACCAGACAGCTAGCCCGTTTGCCTTTAAAGACATTATCCAGACACGCTTGGTTACAACCGATACAAGTATTAATCAAATGCGACTGTTTATTTTCAGCCTTAATGACAAAATGCGGATCCGCTAGGAAAGGTCTTGCCATGGACACCATGTCAGCGTGACCACCTGAGAGAATACGCTCAGCT

>NODE_89_length_2927_cov_2.080351

GTCACCGGTAGCAAGCAAAAGTACACATACAAAGAGCTCAGAGACCAGGTCGCTAAGATCGCAGGGATGTTAGCAGAGCAAGGGGTGGAGAAAGGCGATCGAGTTGTTATCTACATGCCAATGATTCCAGAAGCCGCAATGGCGATGTTGGCATGTGCACGATTAGGGGCCATTCATTCTGTGGTATTTGGGGGCTTTGCCCCAAACGAGCTTGCGGTACGGATAGAAGATGCTGAGCCAAAAGTTATCATGACAGCGTCTTGTGGTATCGAGATCAATAAAGTCATCCCTTATAAACCTATGGTTGATAAAGCAGTGATGGATAGCCGTTGGAAACCAGAAAAGGTTTTGGTATTACAACGCCTACAGTGTGAGGCTGAATTAAGTCAGGCGCGAGACGTGGATTGGATGCAAGCGTATAACAATGCTTTACCTCACGCGTGCGTACCGGTTCTGGCCACTGACCCTCTCTATATTCTTTACACCTCAGGAACAACCGGAAAACCGAAAGGAGTCGTGCGTGACAACGGAGGTCACGCTGTCGCGATGAAGTACTCAATGAGTGCTATTTATGATGTTCCTCAGGATGGTGTTTATTGGGCTGCTTCTGATGTGGGCTGGGTAGTCGGGCATTCTTATATCGTCTATGCGCCGCTTATTCATGGCTGCACAACGATTTTGTTTGAAGGCAAGCCTGTTAGAACGCCAAACCCAGGCGCCTTTTGGCGCGTGTGTGAAGAGTACAAGGTCGATGTATTGTTCTCCGCGCCAACTGCTTTCCGTGCCATCAAGAAAGAAGACCCAGAAGGTGAGTACCTCAAGCAATATGACCTGTCGAATCTAAAAACCATCTTTATGGCGGGGGAACGTTTAGACCCTCCAACCTTGGAGTGGGTAGAAAGTAAAACCGACAAACCGGTTGTAGACCATTGGTGGCAAACGGAAACTGGCTGGGCAATTGCGGGTAACCCTACAGGGATTGAGTTGATGCCTGTTAAAGCAGGCTCAGCGACTAAACCCATTCCCGGTTATGAGGTTGAAATATTGGATGAGTTGGGTGAGCCAGTGGCACCGAATAAGCAAGGTTTTGTTGCGTTGAAACGCCCATTACCACCCAGCTGTTTACCTACCGTGTGGCGCAACCACGACCGATTCGAATCGGGATATCTGAGCCAGTTCCCAGGCTACTATGTGTCAGGTGATGGTGGTTACCTAGACGAAGACGGCTACCTGTTTATTATGGGCCGTATCGATGATGTGATTAATGTCGCCGGACATCGATTGTCGACCGGCGAAATGGAAGAAATTGTTGGTGGCCATCCGGCTATCGCGGAATGTGCGGTTGTTGGTGTGCATGATGACTTAAAAGGTCAGCTACCATTAGGGTTTGTGGTACTAAAAGACGGCGTCAAAGTTGATGACTTGCAGCTTGAAGGTGAGCTTGTGGGTAAGGTACGCGATGAAATAGGCGCAGTTGCCTGCTTTAAGCACGCGCTGGTCGTTGAGCGTCTACCGAAAACGCGTTCAGGTAAAATCCTTCGCAAGACCATTCGACAAATAGCTGATGGTGAAAAGTATACTGTTCCTTCAACCATCGATGATCCGACGAGTCTGGAAGAAATTCAGAAAGCCCTCGAATCATAGTTGAGACGGCGTTCCATAATCACTAATTAGCCCCTTTTTTAAGGGGCTTTTTTGTTGATAAATTTTTATGTTCGACTCACACTGTGGTTTATTTGCGACATCAAAATAAATATGCACAGATTAGAAATTAGAGTCGCTCAAGCGCAGGACTTAGAATCGCTCAATGACATGATGTTCAGGCTGCACGATGAACATCATCAGCAATGCTCTGAGCACTTTAAAACGGCTGAAGATATAGAGCAGGAAAAGAGCATCGCCCGATACTTAGATGACCCAGAATGTATTGTACTAATTGCTTTTGAAGAACAACGGATTGTTGGTTTTGTATCTGGCCATTTTTGCGAACTGATTTCCAGTGTGAGTAAGCCTGTGCAAATGGGAAGTATTGATGAACTTTATGTCCTTCCAGAGTGTCGTAAGTCGGGCATTGCAAAACAATTATGCAAGTCGATTGAACAGCGTTTTGATGACTATGGCGTAAAGCAGATATTTGTTGAAGTGTGGAATTTCAACAGCAATGCGCTGGATTTTTATAAAGAACTGGGTTTCGAACATCACATCCATTGGCTCCGCAAACCAGTGGGTTAAACAAGAAGTAATAAATGACCATTTTTAAGCAGTTGGATCGAGTGTTTCTATTTTTTACACTCACATTTTTCTCATCTTTGCTTTTCGCTGAGCAAAAGCAGCCAGAAATAGAAGGCGCCGCCTGTGTTATCAGAGCGGATGATAAGCTGGTGCTTGTTAATGAAATTTTGACTGGCAAGTCATGGTTGCCTGCAGGCAATGTGAAACGAGGTGAAAAGCCAGAATCAGCCGCACAGCGCGAAACTTGGGAAGAAACTGGGCTGGTAGTCTCAATCAATAAAGTTCTGGGTCAGAGAAACAACACGATTTATTACGATTGTATCTCTGATTCTGAAATTGTGGCGTTTCACATTGATGATTCATTTGATGCACACCAGTTACCAATATGGTTTGCACCGCATTATGGCGTTGAAATTGCATCGGCAATGTTAATTTCGCCAGAGATGATCAATGCTCAGGAATATCGATTCCCAGAACAACTCAAACGGATTGCAGGATATTTTGAGCAAGCAACTCCTCAACCTGTTCGATATGTGGATAACTTGGTTGAATCTGCGCCTACATTTAATCAAATGGAGTTGGCTTGGTTAAATGAGTTCCGTTTGTTGCTAGATAAGCTACCAACTCATGCCGAAGCCATTATTGAAGAGCTGTTTAAGTTAAGCCTTCAACTTAATCATCCCATTATTCTGTTG

>NODE_90_length_2913_cov_2.020451

GAACATGCAGAGCGATTAAATATTCATCAGGGTCAGATTGAAGACCCAAGCCTCGCTCGATTACTGGATGGCGTTGCCCTCCTCAATGCGACGGTAGAAAAACAGTTATCGGAACAATTACCAGAAGTAGTCGAAGGCCTATTAGGTGTGCTCTACCCGAGCTATATTCAAACTATTCCGAGTGTCGCATACCTACAGTTGGCGCAAGAAGAAGATGTGCCGGAGTCAGTTTTAATACCTAAAGGCTCACACTTTACTTCAGAAGCTAAGGGCCGATTGTGTCAGTTTACGACTGTAGACGATCTCAAAACTGCGCCTTTTACATTGACCGATACAACAGCCTCCAGCGCACCGTTTAAATTCAATCGTCCCGCTGGTACCGAGCAGTCTACCGCAGTGATACAGCTGTCGTTTTCCACTGGGGATCCAGATACTCATTTTTCTCAGCTCGGTCACCAAGACCTAGATCTATTTGTTCAGGGTTTCGAGAATAATGCCGATTCTCTGGTGGAGCTCTTGTTGGGGCAAACCAAAGCGATTTCGATATCGGATGCCAGTTGCGAGAAGCACGTTGTCTTATTAGCAGAAAAACTTAAAAGTCGGATAAGTGACCTGAATTTCCTGTTCCTTCCTCAAAAAGGAAATCAATTTTCAGGCTTTCAACTTATTAGCGAATTTTTCTTCTTCAAAGAAAAGCGCCAGTTTTTCCGTCTGAAAGATTTCGGACAAGCTATAAAGCAGTTTGATTGCTCCGAAATCAAACTGAACCTGTTTATGAATTCAATTCCTACAGAATTCATGCGCTTATTCGATAATCAAGTGTTTAAGCTGGATATAATCCCAGCGGTTAACTTGTTTGAACAAACAGGTGAACCAACAAATTATGACCAACGCTCTCTGACTATTCCTGTCAATGCAGATGCTCACAGTGAAAGTGACATTGAAGTTGTCGAAATAAAAGAAGTCTTTGAGATCACCCCTCAAGGTGAAAAACCTCTGGTTCCACTATTCAGGGATAGCTATAAATCCAACCCAAATGCCGATTATTGGCAGAGTTCAAGGGATATTAATGGTCAATTCCAACTTGCCATCAGCCTAAAGCCAAACCATGATTTAGAGTTTAACAAGCTCTATGGGACACATTTGCTTTGTACCAATGGAAAACAAGCATGTGGTATTAATGGAGAGATGGAGTGTCTTGAAAGCATTGACCTTCCTGGCGTATTTCGTGCTATTTACCCACCTACGGCTCCGATTGAACGAGAACAGAACCTCAATCTTCACTGGCAGTTTGTTGGCTTATTGAATGGTAACTTTTCTTCACTGTTACATGCGGATAACCCGACAGAGAAGCTAAAGCAAATGCTGCAGCTATGCAGCCGAGAGCAAGTATCCGCGGAAGAAATACAAACGATTCACAATGTATCCATCAAATCTCAGGTGTCTGCAATTCGAATCATGGGTAAAAACGTCTTTTCTCCAGGTACAGAGATTGAACTCACACTCGATACTCAAGGGGGCTTTCTGGTCTTTAGCGATGTTCTTAATCGTTTCTTCCAACAGTTTTGCAGTTTTGATCGCTATATCCAGCTGTCTATTAAGATATACGGGCGAGATGGCATCGCAAAACGCTACCCTAAAATCCATGGGAGCCAGCTAGCAATGTAGTTGCTGGCAGGAAAGCGATTATGTCATTCATTAACGACCTAACTCAGCACCCGCAGCAATACGACTTCGCTCAAGCGATGAGATTGTTGACGCATTGGATTAATCAGACTCCGAACCGAGTTAAGCTTGTGCTGAAAGCAGAAGCGATGCCAACAGGTGATGCCAGCGAAATTCAGTACTTCTCTCTGAAGAATAATCGACTCAAGTTGAGATTGGCAAAACAAGCTTTATCAGGTGTAAAAGGGGTTATACCTAACTACATATACGAAGAGCTGTTAGCGGCTTTGCACAACGATGACCATGCTTTAAAAGACTTTCTTGATGTCTTCAATCAGCGATATTTCGAAATTGTCAGCCAACTGGAAACGGGCTCCTGGCTTGTCGTACAAAATGAGCTCTACCCTGCAAAGACCGCGTTGTTGAACCATATCGCGACATTAAAAAAGAATCATGGCAAGTATTTCCAGTATTCGATGTTGTTAAGCCAAGGGCAGCGCAGTTTAAGTACATTGAGCCAAATGCTAAATGACTACTTCCCTTTCGCTATTCAAATTTCCACCAAAACACATGAAAGACGCCAATTGCCTTCTGACTCTCTAACTCGCCTGGGGCAAAAAGAGCATTTTAATAGCCGAGTGGGTCAAGGCTTTTTAATCGGCAAAACATGTCTGACACATTTTAGTCACCTAATTGTCTCAATCACTCCAAAAACAGAAAACGAGTTGATAGCGATTCAATCAGACCCATTGTTGGCGAGCAATATGAAAGAGCTCATCCAACATTACTTGAAGGACAACACCCCGACTTCCGTTTATCTAAATGTGAAACGGGAGTACCTAAATCGACCACGACTATCAAGCAATAAATATAACGCGGTGAAATTGGGCGAAGTTGATTGCCTTGCGCCGGAAAGGAAACCTCAACAAATCGTAAGTATTCTTCTGAAGTAATCTGACTTCAGGACCAAACAAAGAGTTAGGTATTAAAGACGTATTGATGCCTCTTGAGATACAAGGAAAACGGTATGACGCAAGTTACACTAACAAATTTAGTCGCTAAGCTGTCACCAGAGCTGAAACAAGCACTTGAAGTTTCAGCTGGTGCCGCAATGAATCAAAATGTGCCTTCTATGGAGACAGAACACTGGTTGCTTCAGCTTCTTTCTCAGCAAGACAAACATTTAAATAGCCTGATTCAATCACAGAACTTATCCCAAGATAACTTGGTCAACGAACTGTCTTCAAA

>NODE_91_length_2912_cov_2.598942

CAGCAAGGACATCGGACAATCATCTTGAGAGGCTTTCGCCCATTCTTTTTCGAATATCTCCTCAAAGTAACGGCGATTGAAGAGCCCCGTTAACGGATCGCGCATCGCCTGCGAGCGCAGTTTCTCTTGAAGGCTCAAGTTAGCTAATGCCAATCCGAGATGCTCAGCAATCGTGAAGGCGAGTTGTTTGATGTCTTCGGATATTTCTTCAGATTGTTCATCAAAATAGAGATGGAACATGCCGACAGTATTACCATGAGCGGTTAGCGGAACGCACAACGTGAGCCCTTCAATGGAAGAACTACTCATATGGGTGCAAGAGAGGTTATGGTGTTTTTCTTTCGATAGGTGGTATTTACCTTTGCGTAATGCCCAGCAGTCATTGGGGGCGTAGAGTTTACTCGCGGGCCATTCTCCTCCCCAGTCGAGTTTAACTTCGAGTTGATTGCGTGAGTCACGCATGATGGAAACACAACCGTTCACTTTGCCTAGCACTCTTGGAATGATGTCTGCGACGACAACTTGTGCCTCTTTGACGCTGTTACAAGCGGCGAGCATATTTGCGAGTTGGTGGAGTAGCTCAATCTCTTGAGTACTTTTTTGAATACGGTACTCCTGTTGATCTCTTTCTTCTTTAACAAGCTTGTAGATTTTTCGGTTACTCAACCAGGTTAAGAGAATCAAAATGGCCAGCGAAAATGCCATTAGCGCGTGCTCAATTAATTTGAGGTCGGCGGACATGGCATAAAGCGAAGTCATTGGCATCGCGAGTCTGAGAACATAAGTTGAGTCGTGGCCATGGTCGTCTTCAGGAAGTTCGAGCTTCTTAGCGACATAAAGTAACTCTTGGTTTAAGGACTGGCTAAATCGTTTCGATAAACCAGTTTCACCGGACAGAGCAGCGCTTATCTCGGGTCTGCTACTGTGATCTTCCAACTGGGAAATCTCCCTATTGGTAAGTTGTGAGTCTCCCAGCACGGCTCCCGAGCTGTGTATGATGGTAAATCTTTGTTCAAAGCTGGATTGCGAAAGGTTATCGAGGAAGTCGTCTATTTCAGGTTGGCTCATGTCATAGAGGTCGAGCTCTTTTTCTTGAATCTGATGCGAGACGTCGTCAAGCAGATTAGACATGTAGTTACTGACGATATCAGTTACCCAATTGTCTAGGGTTTTGTCGTAATAGTAACCACCGACTACCATAGAGAGAGCAAGGGCGAGAATTGGGAAATATTGAAAACGACTTAAAGAAGAGCGAACCATAGAATCCTCGTGTTAACTGCTACAAAAACTGATGATTTACGTAAAAAGTCAAACGATATTGTCCAGTGATATTAATATTAACGATATGTAAGTGTAGAAGGTCTTAAGTGTTATGCAATTGCGTTATTGCTCTATGGTTGCATAAAACTTTCTCATTTAATCATTGGCTTATGTGAAGGAGACAGGATGAAGTGGTTGCGTTTAGGAACAATCATTCTTGCTCTAGTGGCATTAAAACCTTGTGCTGCAAGGCCGGTTGATTTGGTCACACTTGAATACCCGCCCTATATAGAAATGCAGGGACAGCAGGTTTCAGGGGTTGCTGTAGAGCTAGTGAGGTATGTGTTCCATCAGTTGGAAACACCAGTCAACATCACCGTATTGCCTTGGGCCAGAGCCCTTACACTGGTAAAGCGTGGTCGTGCGGATGGCATTTTCACCGCCTTTAAAAATCCTGAGCGAGAAAAATTTGCAGATTTTAGTCAAAATGTGCTCTTCGTGCAAAATATTAGTTTGATGGCGCTTAAAGGTAGTGACTACAAACCTGAGGAAATTTTAGCGGGTGATGTATCCAAAATCGCTTTGTGTGTCGTCAATGGTGTGAGTTATGGAAAACGAATGGATGCTAAGATAGAAGAGGGTCGCTTTCGGCTTATTTTCCAGAGAAACAGCACTGTCGAATGCGCTCATCTCGTCCGTACAAAACGCGCTGATATTTGGGTAAACAATGAATTTGGTGCCCGAAGCATTTTGGTCAGAGAAGGACTAGATAGCGACATTGATATTTTATCGCCTCCTATTGAAGCAACGCCCAGCTACATTGCATTTTCCAAAGACCGCAGCCATGCCGAATTGCTCCAGCGCTTTGATCAAGTATTATTCGATATGAAGCAAGATGGACGTTACGAAGCACTCATATATGATTATTTTGAGAACATGAGAGTTAAATGAGAGGTTCTCTTTTCATGTAGACAAAAGCGTTATCTTCGAACGTGAATTGGAACCCGAAGCGTTGATAAAGACTGCCCACACGGTTTCCTTGCAAATAACACAGTTGGATCGGCTTACGTTGGGCGTCGGCTAGCTCAATACAGTATTGCAGTATTTTGCTTCCCATCCCTTTACCGTGGTATCGGGCCAGAAGGAAGAAACGACAAAAATACAGATGCTCGCCTTTATCTTGCAGCAAAAAGCTACCAGCGGGCTGGCCGCCAACAGTCACGATGGTAGGCTGGTCTTCCTGCCATTCTTCATGATGAATACGCTGCTGTACTTCTTCATCCCAACCAAATATGGCTTTTATCGCTTCGTATTCTGCGTTCTTTTTGAGAGAGAATAAGAAGTCGAAATCGCCCGGTGTGGCCGGGCGAAAATCAAAGTCGATCATGCATCAACCAGCTCATAAGTAATCAGGAATAGCTCGTCGGTGTTGGGGTAGATTTCACGAATCAGCTGCTTAAGCTTTGGCAGTTCAATCGCTTCTTGCTCAGCGTGAAACTCGTTGATGTCTTCAAAGTGAAGGGGCTCTACCGCTAGGATCTTCACCTCACACACTTTTTCGTCTTTCTCCAAAGTGAATACTTCAACCTGAGTACCCGGCACGTAATGAGATTCTGACTCATCACGAATGGTAATGGTTTTCTTACCAGAAG

>NODE_92_length_2907_cov_2.507420

TACTTGCACAGCAGATGGTAGTAAGGGATCACGACCGCATCGAACAGCTGGCTGAGCCATGACTCGATAGACACACCCGATGCGCGAATGTACTCGCTAATCAAAGATGCACCTTGGCAGTCCGTCTCCATCAAGGCTGCCATTAGTATCGCGCTTTCGCCTTGTTTGAGCTTAGAGATTGCCGACTCTCGCCAGATCACACCAAGCAGCTCATGATAACGATATGGCGCGTGTTGCAACTGCGCATAATCATGTTGAGCGGCAAATGCCGCTGCGGGCTCTTGCAATACTTCGGCTTGCTTTTCCAGCAATATTGGATCGCTTTGGAAAACAGAATGGATCCAGTCAGAAGCGCTAGGGCCTGCTAGAATATAACGCCCCGGAATACCGCGATAGCAGGAAGTATTCATCACCGTTAGCGGCAGTTTAATATCGAAATTCTGCGGACGGGTGGCATTACTCAGCGTACGTAAGGAAAGCTGAGGAAGAAACAGATCACCAAACTCACCCAGATAAATCAACTGCTTGCAGGCCTGTTCACGAGTAAATAGCAAAGCAAGTTTGTTGTGCCATTGCCAAGGGTGGACAGGCACAAAATGGTAATCAGCAACATCTTGATCCAATCCGGAAATCACCGATTGCATTTGGTCAATGTCAGCCTTGTTGACTGCGCTTTCCACCAGTTGGCCCCAATTGACTTCTTGATTGGTCGATACTTGAAGAATATCGTTGTGTACAGCGACCCAACCCAACTGAAAAGCTTGCTGAGATTCAGGCGCATAGCGCAGCAAGTCCTCACTCCCCCAGCCTCTTCTACCTTTGTTAAATGCAAATTTTGGGTGACCTTCGAAATAAGATTGCTGCTCTTCACACGACATTAGACTCAAATCTTTAGCCGTTATCGATGCCTTGCGCGCCATTAACTGACAATCACTCCACAAAGTTGCATTGAGGTCTTCAAGGTGCTCTGCCATGGCTTCATCTGGCATCTCAAGCAAAGCCTGAATGTCGCGCATAAACAGTGCTGCACTAAGCGGTTCTTGTTCACCATTTCGTGATGTGCGAGCCAGTGATTGCGGCTCAATCACGATTTGGCCCCAGATGTTTTCCTTTCCATCAAATTGATAGGTAGTGCCGTTATCAAGCTCAAGCACGAAACCTTGCTCTACACGGTGGATCGAAAACGCCTGCTCGTAACCAAATTCACTGATGAGCTTGGCAACCATTTTGAGGTTCGCAATGTGCCAATACTTGTGAAGTGCGGATTGTGTCATCACCATTGCTCCTCAAAGAATCGATCACGATTGAGCATTAACAACGCGGAACGTTTGTGAGGAAAATTGAATTCCATACATTTGTCATAGCCAAGCGGCAAAATGCGCTTGAATAGCCTCTGATTGTCGTGGCGAGGTTCAAGAACAATACGCTGAGTTCTGGCATCATCAATAAAGAGATAGTGACTGATTGCTCTCATCCAGCAATCAAAATACTTAGGCCCACGAAAACTTTCTTCACCGACCAACAAGTGGATACCGCGGTCAAAATTTTCGACCGGATAATAAGGGCTCAATCGATCTTCAGCGACCCAGTAAGCTTCGATGTAACCAAAAGGATGGCCGTTGAACTCCGCTATCAAGGGGATAATATGCGGATCAGCTAGGCGCTGCTGAGCGAACTCAGCCAGTTTCTCTCGGCTCCATGCTTGCTCCCAAAATTCGGCCACACGCGGATCGTTCATCCAAATCGTAAAGTTATCGAGGTCTTTCTCCAACGTAAAAACACGAAAACTCACTGTTAATTCAGACTTAGCGTCATAACGCTGGTAAACCAAACCTTCCGGCATCGGTGGCCTCTGCGGGTGATGGCGATATCGGCTATCCGTCTGGACCTGAAGCAATGGATAGCGATCGGAAGGCTTGTGCTTTAGCCAGTTAAATTGGCTCTGATAGAAGGCGGCGCGCTTTAACGTACGGTCAGTAAAGATAGGCAACTTCTGCTTAACTTTGTGCCAGACATTGATGTCCTGACATTCGATCTCAACAGCCAAAAGCTCAACATGCTGGCTAAACAAAGCATCCAGTTCATCCACCATCAAAGACAATAAATCAGCTTGGTTTAAGTCACTATCTTGTATAGCGATAGTGACTTTAGGCAGGTTATCAGGCTGATAGGTTACGGTGCGAAACTCATGCATGGCTGGTCTCCTTAACGCGAACTAACGGGTTAGGTAAATCGAAATAGATCACCGCAGGATCGGGAATAGAGTTTTCATTGAAATTCTGCAGGTAACAGAAGAAGTTGCCCTTACAGCATAAGGACTGGCTTTCCAGTACATAGTGCAAACACTGATCATCTCGAACCCCAGTATCTAACAACCGACGGAGATGTTTATGTAAGATAGCCACGAGTTCCAACTCCTCAACTCCACATTGGGCGGCTAAGTTGCTGATGACGTTGTACGAGGAATTAATGATCAGATAGTAGGCGAAATAGCGTCTTACCTTGTCCTGATCCCAATAATTCTCAACATCTCGCTTGGCTTCTTTTAGCTGCTCACCAAAAAGTTCAAAAGCAAGATCGGTATAGCCTGTCCCCTGACAGTCTCGGTAGTACATTCCGATTGGAAAACCATTCTCCAACTGCATGACTATGTTCTGCTGATGAGCGAGAAACACGACACCAAAGTTAGCTTGCAAATGAAACAGAGGCACCACTGTGTGTCGGCAATAAGCGTCAAACCAATTTGTTGCCGCATGTTTCACGTCAACGCTTTGTGTGCGGGCATATTCACTAACGCGGTTCGCCAAAAGACTTGCCCCACCGTACGGGCTTTGTTGGGTCAAGGTCGCCAGTACGACGGCTTCTTGATCGGGCTGGATGGCTAATGGGTTATCCCGCAATGCGACA

>NODE_93_length_2907_cov_1.881272

ATAACCCTCAGAATAGCTTAATTACGCTTGAGCTCGGCCTCAATGATTTCATGAACTATGACCGTGAAGTCGCAGATGTTAAAGCGGACTTAAGTAGTGCAATGATCCGATTAACAGAATCTGGCGCAAACAACATCCTATTGTTTACCCTACCTGATGCCACTAAAGCGCCGCAGTTTAAGTATTCAACACAAGACGAGGTTGATACGGTAAGAGCCAAGATCGTTGAGTTCAATGAGTTTATCAAAGAACAGGCGACACTCTATCAAACGAAAGGGCTTAACGTCGTGCTTTACGATGCACATGATATATTCGATCAAATCACATCAAACCCTAAACAGCATGGCTTTGAAAACTCAACGGATGCATGTTTGAACATCAATCGTAGCTCGTCAGTCGACTACCTCTACAGCCATGAGCTTACCAATGACTGTGCTTACCACAGTTCGGACAAGTATGTTTTCTGGGGGGTTACCCACCCTACAACTGCAACACATAAGTACATTGCAGACGAAATCATTCAGACAAAGCTAAACCAGTTCAATTTCTAGCTAGGTCGGAAATCAAAAAAGGAGGCAGTATGATTGCCTCCTTTTTCCATTTTAATGCCTAGCTAAGCAGTCACACTTTTAGCCCTGAATACGTTAATGCCGAATAACGCGATAACAGCAAGCACAAACGGTAGCACATAAATATTTAAGTTCTCCCACCCTACCGTTGCTTCCAGCCAACCAGATAACAAAGCAGTAATTGTCACACAGCTAAAGACAAAAAATTCGTTAAAGGCTTGGGCTTTAGATTTGTTGTGCGCGGCATACGATTGACTGAACAGCCCCGTTGCGGCGATAAACATAAAGTTCCAACCAACACCGAGTAAAACCAGAGCGAGCGAAAAATGCCAAATCGACTGACCATGGATATTAATCGCCACGCAAAGCACGAATAAAATACCTCCCGTCAGGATCATTTGTCTCGCGCCAAACTTTTCAATCAATTTACCGGTAAAAAATGCCGGTACGAACATCCCAAGCACGTGCCACTCGATGACGCCTGCGGCTTTCTCGAAGTCGAAGCCACAACCTATCATCGCAAGTGGCGTGGCTGTCATGAGAATGTTCATCACGGCATACGCCACCATGGCAGCAAACACCGCCAACATGAAATTAGGCGCTTTGATGATTTGAAGCAGTGGCTCAGGGTTATCCTTAGCAGTTCCCGTATGATGCGCTGGGAACTGAATGGTCTGAAGTAAACACAATGCAATAATATTTAGCACAATCAATGACATGAAAGCACCTACGTATAGTGAATCACCTGTCATTTGTTGCGAGTATACGGCTAGATTAGGTCCAAGAACCGCAGCTAAAACGCCACCAGCCATAGAGATAGATATCGCACGATGTCGTGCGCTTTCTTCACACACTTCAATTGCTGCAAAACGATACAAGGTACCAAACCCGATACCAATACCAAGTAAGAAAGTCCCGACACAGAACAGATAAAACTGCTCATTAGACAAAGCATAGGTCGCTACTGACGCACCGGTAATGCCAACCAAGTTACCTAAACTAAAGCCTTTTCTTCGACCAAATTTCCCCATGATCAGAGAAGCAGGAATCGTTGCGCTCATTAGCCCGAGGAATTGCAATGCGACGGGCAAGGTGATCATACTTTCACTTGGCGCAATTTCCTTACCCACTAACCCAATCACAGATATCAGCAGGATATTGCCCGTCATCAGTAGTGCCTGACAGAGTGACAGAATCCAAACATTCCTATTCACTTTAACGCCTCGCTAACCGCAGAGTTTGCGTGTATTTCAGTGGTGTCGAACAATGGAATATCCGTATCTTGCTCTGAAATCAATAAGCCTATTTCGGTACAACCTAGAATCACGCCTTGTGCGCCGTCCGCGGAAAGATCCTCAATAATGGTTTTAAAATCTTGTTTCGATCTAGCGTTTATCTCACCAAGACAAAGCTCATTGTAAATAACATCATGGACAAGCCTTCGCTGAACACCATTTGGCACAATCACTTCTATTCCAAATTTATCGGTTAGGCGTTGTTTGTAGAATGACTGCTCCATTGTGAAAGCCGTGCCAAGCAAACCAACTTTATTGATTCCTTGCTCTGCCAACTGCTGACCTGTCGCATCGGCAATATGGAGTAACGGTATTGAGACGGCTTCTTCGACCTGACTCGCCACTTTGTGCATCGTATTGGTCGCAATTAGAAGAAAGTCAGCACCAGCCTTTTCAAGTGATTGAGCAGCTTCACTAAGAATAATTGCGGTTTTATCCCATTCACCTTTGTGTTGCAGGGCTTCTATCTCAGCAAAGTCGACGCTGTTCAGGATGAGCTTAGCTGAGTCCAATCCGCCTTTTTCGGCTTTAACTCTCTGATTTATTTGCTTGTAGTAACTTGCTGTTGATTCCCAGCTCATTCCACCAATAATACCGATTGTTTTCATCACGACTCCCTTCACGTAGATATTCCACTAATTAATCAAATGCATAACAAAATTACAAACAAAATAAAATGAGTCAGGATTATTACTCTCCGCCCTCACCCTAAAATTCTCACCCCATAAAAAAACCCTGAGCTGTTCACTCAGGGTTTTTGGCTTTTCAGTAACATCAGGCTAGCTAGACACTTCTTCTTTTACGGGAAGTACTTGCTTCACATAGTCACCCGGTGCAGGCCCTAGTACAGGGAACTGTTCGTTACCTGTACTGAACGGCTCAACTTGCTCTTCAGGGCTAAAGCCCAACAACCATTGATGCCAATGTGTCCACCAAGAGCCTTCAGTATGAGTGGCATTACTTAGCCATTCATCAGCACTCTCGTCCAAGCTATCGTTGGTCCAAAAACCGTACTTGTTCTTCGATGGGTGGTTAACGATACC

>NODE_94_length_2899_cov_1.920978

CACCATATGCGCTTCATCGCCCTGACCAAAGTAAGAGATATTCTCTTTATGCGGCACATTGGTTTCTGTAATCAGCAAAGAGCCCGGCATAACGTCATCGAGAACAATGCGCCATAGCTTGATGATTTCATGGGCTTCCTCAAGGTGAATACAGGTTGTGTTAAGCACTTTCCAGATAAAGCCGATGGCATCGAGACGAATAGAACGACCACCGTTCGCTGCGTACATCAGCAAAATATCGATGCTTTCCAGCAGCACTTTCGGGCTTCTAAAGTTAATGTCGATCTGATCGTCTGAGAAGCTTGTCCAGACATGTGTCGTCTCACCCGAAGTCTTGGTAAACGGCGTCAGGAGCGGCAGGGCGCGGGGGCGCGTAACGCTCGAATAATCAAGGCTCGGGTCAGACTCTACAAAATAATCCTGATAAGCCTCATCGCCCGCAAGATAGCGCTGGAACCAGTCGCTGCTCTTAGAGATGTGGTTAATCACGCAGTCGTACATCAGGTCAAAATGTTCCGCCAGCTCATTCAACTCTTTCCAGTCACCCAAGTTCGGGTCAACCTTGCGGTAATCCACTACGCTGAAGCCGTCATCCGAAGTGTATGGGAACATAGGTAGAATATGGATATTGCTGATCGCGCCACGCAAGTACTTATCGGCAAAGTACTTCATGGTATTCAGCGTCGGCTCTCCCTCGCGGCGAATACTGTCGCCGTATGTGATTAGGTACGCCGTCGATTCGTCGACCCAGTCCGGATAAGACGGCGCATGACCACGCCACTTATCAACCAGTGCCAAAATGTCCTGTGTCAGTGATGGTGCCAGCTCTTCACCGTACAGCTTGGCAACTTTGTTAAAAATCTTCTCTTTCATTGTGATTCCAACCTGTTAAATCTTGAATCTAACGCGCAGGTCAATGGCATTGTTTGGTTTGACTGTCCAATTCTGCTCGACACTCTGCTCTCGGTGATTGACACGCTCAACATCAGCGAAATGAGCGAACGCGGTGACATCGAGTTGCTGTTCACTGTCCGTCGCATTAAATAGGCGCACAATGACCGCATTGTCTTGATAGAATGAATGGCCAACGCTGGATAAAACCAACGGCTGATTCAGTTCAAACAGGCTGAATTTTTTCTCAGCTTCGCGGCTATCGAATCGAACCTGAAAACGTTCGAGACGATTCTCAAAGCTGTTGAGCGACTGTTTCTGGTAGCTGAATGGGCTATCAAGATATTCACTTTCAAGCTCTCGGATTGTCTGATGCTCAGCGTTGTCTGCCAAAGCAATAGCAAAGCTGAACTGCATGGATTTTTGCAACTGAGCATCTGGCGTGTACACCACTGTGTTGTTGATGCCAGAGGCGCGACCAGGTCTCCATAACAGATCATCTTTACCGCGCTTGCCTGTTGCCTTAAACAAGGTCAGAGCAATCAAGTCACTGGTATCACCTTTCAAGATCTGGAACTCTTTCAAACCACGACCATTAATAATCAGGGCTCTATCCTCTGCCGCTACCGCAACCGCGCCATCGGTGGTTTCAATATCTACCGGACATTCGCGGAAGCTGTCACGCCAGCCATCAACATTTGGTGCCACCGGACGAGTCATCAACGCAAATGGCTGAGTGCTGATGGATGTATCGGTCTGAACATCTGAATTAATCAAAACGCGCACACGGTGATCATCAACCTGATTAAGCGTATCGATCTCTACTTCCAGCGATTGTTTACCTTGAGTAAGCGTCAAGCGAACGATAAATTCCGCTAGAACAGATTTTTCACCGTTCTGGCGAGCTTCTAGGTTTTCCGGAAGCTCAAGACTTGCTTTCAGGATCATCGACTGCTGGCTTTCTCCCACATGAGATTCAACCCAGCTCAGTTCGCTAGTGTACAGTGGCGTATCGCCTTCCAGCGGTGAGAAGTCATAAGAATCGCCGTCATCGGCTTGCTCTTCAAAGCGGACAAGCTGCTCAATGATGCGCTCTGAACATTTATCTTTCAGCTTAAGCTGACCATTCTCCAGCCAAAGCGCCAGTTGACTGTTTTCAATCTGAGTTGCAGAAGAAGCCTGTACTTGAGTAACGCAATCTTCTTCCACGACCTGAAATACCTGATAACCCATTGCTGGCAGATCCGCCACATCGACATTGAGCTCAAATCGGTAGTAAGGAGGAACAGGCACTTCTTTTTCGCCATCTTTAGTCACTTCGATGACTTTACCGCCATCCAGTGTTTCACGAGTGACCACTTCACTGCGAAGGGTTTTCTCGCCATCACTTAGCGCGATGGATTCATGCTTGGAAAACGCAACAACACGGGTAATGCCGCTAAATGGCGTCGTCTGGCCGTTGAAGATCATCACTTCCGAATCATCACAGGCATTGGTGGCAATTTCTTTGACCACCAAGTTGTACAAGCCATGACAGATTTCTTCCGCCTGCTTAAGACGATGCATGATATCAGCGTTGGTGGCATCGCTATTACAACCACCCATGCTGTCGTGCGCATGGCACTCAAGGATCTTCTTCCATGCCAAGTCCACAAGCTCTGTGTGCACCGTCATGCCCTGTGCTTTGGCGATCGCAATCACCACTTCCAGCTTCTTGAGCAAGAACTGTTCAATCTCAAAGTTAAGCTTTTTGATGTCGTAGCGCACAGAACCAATGGTCTTGTGAATACGTGTGTAGCGCGGTGTTTTGAACTCACCATAGTAAGTCTCAAACCCTTCACTATTGCGGCGTAGGAAATCGACGAAGTTTTCCATCGAAGAAATAGAATACACATCGCCAGATGGAGAACGCTCAGACGCTGCCGCTAACGTGGCTGGCAAATTAGGGTCGATATTGACTTGGTCGCCACCCGATGGAATCAATACTTCATCAAGGCCTGATAACGAC

>NODE_95_length_2890_cov_2.442943

CTTGCACCGTGACCGACTTCGAAGTGTGTAAGCGCCACGGGTTAGAGCAAACCATTGCTGATACCTTAGGCCCGGGCGGTATTATGCGTTCGTTACGCACCATTCCGCACCTGTGGAGTGTGTGTGAAGACATGAAACAAGTCTGCCCGGATGCCACCATGCTGAACTACGTGAACCCAATGGCGATGAATACCTGGGCCATGTACGAAAAGTATCCAGAGATTAAACAGGTTGGCTTGTGTCACTCCGTGCAGGGGACGGCAGAAGAGTTAGCACGCGATCTGGATTTGGATTACGCCGACTTGCGTTATACCTGTGCGGGCATCAACCACATGGCTTACTACCTGACGTTTGAGAAGAAGGACGAGCAGGGCAATTACGTTGATATCTATCCAGATTTGCTCGATGCGTTTGAGCGTGGCGAGGCTCCTAAACCAGGTTTGCATCACGATGGCCGTTGTACCAATTTAGTTCGCTACGAGATGTTCAAGAAGCTGGGCTACTTTGTCACGGAATCTTCCGAGCATTTTGCTGAATACACGCCTTACTTTATCAAGCCAAATCGACCGGATCTGATTGAGCGCTATAAAGTGCCAATTGATGAGTATCCAAAACGTTGTGTTGAACAGATTGCTGCATGGAAACAAGATTTAGAAGACTACAAGCAAGCTGACCAAATCACAGTGAAAGAGTCTCAGGAGTACGCCAGTACGATCATGAACTCGATCTGGACAGGTACGCCAAGTGTTATCTACGGCAACGTCAAGAATGAGGGTTTAATCGACAACTTACCGCAAGGCTGCTGCGTTGAAGTCGCTTGTCTGGTTGACGCCAATGGCATTCAGCCGACCAAAGCAGGACGCTTGCCGAGCCATTTAGCGGCATTGATGCAGACTAACGTTAATGTTCAAACGCTGCTAACCGAGGCCATCTTGACAGAAAACCGTGAGCGTATTTATCACGCTGCGATGTTGGATCCACATACCGCGGCTGTTCTCGGTATCGATGAAATCTATGCGTTGGTGGATGACTTAATCGAAGCACATCAGGGATGGCTTCCTGAATGGGTTTATCAATAAAAATAATAATATCCCTTTCAATAACGTGAATCATATACGCTTCCAGTGATGGGAGCGTTAAGGAGCAAACATGAGTATTTCGATGAGCACCAAACTCAGCTATGGGTTTGGTGCCTACGGCAAGGATTTCGCCATCACAATCGTCTACATGTATCTGATGTTTTACTATACGGATGTAGTCGGTATTTCAGCGGGCATTGTTGGCACCATCTTTTTGGTGGCGAGAATCTGGGATGCAGTCAATGACCCACTCATGGGCTGGGTGGTAAACAATACCCGAACCCGTTGGGGGAAATTCAAACCCTGGATTCTGATTGGTACCCTAACGAATTCTGTCGTCTTGTACATGGTGTTCAGTGCCCACCTGATTGAAGGCCCATGGCTTATCGCCTACGCAGCCATTACCTACATTCTATGGGGTATGACTTACACCCTGATGGACATCCCTTTCTGGTCTTTGGTACCAACGCTGACGATTGATAAGCGAGAGCGTGAAGAGCTGGTCCCTTTCCCGAGGTTCTTTGCTAGCTTAGCGTGGACGGTAACCGCTGCGATTGCGATGCCATTCATTAACTATGTTGGTGGTGAAGACAACAAAGGCTTTGGTTTTCAGATGTTCACCCTGTTACTGATCGTTTGCTTCCTGATCTCCACTTTTGTCACTCTACGCAACGTAAAAGAGCGTTACTCAACCACCAAACTCAATAAAGCTCAGCCTGAAGAGAAGGTATCTCTGAAGAAAATGCTGTCGTTGATCTACAAGAACGATCAGCTTAGCAGCCTGCTGTGTATGGCTCTATCTTACAACCTAGCGACCAACATTATTACCGCCTTTGCCGTGTACTACTTCACCTATGTTGTAGGGGACGAGAGCCTGTTCCCTTACTACATGGCGTACGCTAGCATCGCTAACCTCATTACGCTGGTCCTATTCCCTAAACTGGCTCAGTGGTTCTCTCGCCGAGTTCTTTGGGCCTGTGCGTCTGGCTTCCCGATTATCGGTAGTGCAGTGTTGATCTACATTGGCATGAACGATACCCAAAGTGTGCTGCTTATCTCTACTGCAGGTGTGTTCCTGCAAATAGGTACCGCACTGTTCTGGGTGCTTAACGTAATTATGGTTGCCGATACGGTTGATTACGGTGAAGTGAAGCTTGGTGTGCGTTGTGAAAGCATTGCCTACTCAGTACAGACTCTGGTAGTAAAAGCGGGCTCAGCGTTTGCGGCTTTCTTTATAGGTATTGCCTTAACCGCTGTGCACTACGTGCCTAATGTAGAGCAAACGCCGGAAACCATCTTTGGCATGCAGTGTATCATGATTGGCTTACCAAGTTTGTTCTTTGCTATCGCGCTGTTTATGTACTTCCGTTACTACAAATTGAATGGCGCGTATCATCAACAGATCCAGCAACAGTTGATGGAAAAATATGAGCTAGTCGACAACGATGAGCATAATGAAGACATCGTTTCTTCCGACAAACCTCAGCCTGCTCAGGCGTAATCTGTTCAGCCCTTTTATCCAAAGGGCTGCTTTTCTAATTTCTGAACATCCCCATGGAATAGGGAGCTAAACAAGATGAAACACTCATTGATTGCCAGCATGATCCTAGGAGCTCTGCTGAGTGGAGCGGTGTCTGCTAACACTTTACCTGAGACAGTACTTGCAGCTGGAGAAAACCACAGCGTTCTGGCTAACCCAGGGTTTGAAGACAGTGGCCTAGGTTGGCATTACTGGTTTGCCAATATCAAAGACGATAAAGCGTTTGAAGGCCAATATCGCGGCAGAATTGCCATTGGTGCGGGACACAGCA

>NODE_96_length_2855_cov_2.379050

CAGGTCAACCCAAACGCCTTAGTATCAGTCAAACTGGTTTCTGAACCTGGTGTTGGCACTATTGCCACTGGTGTGGCAAAAGCGTATGCAGACTTGATCACCATCTCAGGCTATGACGGGGGAACAGCTGCCAGCCCACTGACATCGGTAAAATACGCAGGTTGCCCTTGGGAGCTAGGCCTTGCCGAAACTCAACAAGCACTGGTGGCTAACGGGCTTCGTCACAAGATTCGCCTTCAAGTGGATGGCGGTCTAAAAACAGGTTTAGACGTTGTAAAAGCCGCGATACTCGGCGCTGAAAGCTTCGGCTTTGGAACAGCTCCTATGGTAGCGATGGGCTGTAAGTTCCTGCGTATTTGTCACCTCAATAACTGCGCAACTGGCGTCGCCACTCAAGACGAAATGCTACGCAAAGAATTCTTTAAAGGTCTACCTGAGCAAGTGATGAATTACTTTGTCGGTTTGGCTGATGAAGTACGCGCCTTGCTAGCAGAGCTGGAAGTGGAAAAACTCACCGATCTCATCGGTCGTACTGAACTGCTTGAGACAGTAGAGGGCATGACCGCCAAGCAAACCAAGCTCGACTTAAGCAATATTCTTGAAGCGCCTGTATCACCGGAAGGTCATCCACTATTTTGGACAGAACCTAACACACCGTTTGATAAGGCTGAGTTGAACCAGAAGATCATCGATGATGCCCTTTCAGCCGTAGAGGCACGCGAGTCCGCTAGCTTCTTCTATAACGTCATCAATACCGATCGCTCGATTGGTGCACGTCTATCAGGTGAAATTGCTAAGCGCTATGGAAACCAAGGCATGGCGGCAACACCCATTAAGCTCCACCTTGATGGCACCGCTGGACAATCATTTGGTGTCTGGAATGCCGGTGGCGTAGAACAGTATCTCACTGGCGATGCCAATGACTATGTTGGTAAAGGCATGGCTGGGGGCAAGTTGGTCATCAAACCACACTTAGGGACAGCATTCCGCTGTAATGAAGCCACCATCATTGGTAACACCTGCTTGTACGGTGCAACCGGGGGCAAACTCTTTGCAGCAGGTACGGCTGGCGAGCGCTTTGGCGTACGCAACTCAGGAACCATCGCGGTTATTGAAGGCGCAGGCGACAATGCTTGTGAATATATGACTGGTGGAATTGTGGCAATTCTTGGCGCAACCGGAGTCAATTTTGGCGCAGGTATGACAGGCGGGTTTGCCTACGTTCTTGATAAGAACGAAGACTTCCAAGGCCGAGTGAACAATGAATCAGTCGAGGCACTGGCGCTGTCTGATCTCTATATCCATCAGGAACATTTACGTGGCCTGATTGCAGAGCATCTTGAAGAGACAAACTCAGTTCACGCTGAAGATATTCTGGCGAACTTTGATGAATGGATTCCAAAGTTCTATCTGGTGAAACCTAAAGCCGCGGATCTTCGTACCCTGCTTGGTCACCAAAGTCGTAGTGCCGCAGAACTTCGCGTTCAAGCGCAGTAATCATGGAGGATGTTTGAATCATGAGCCAGAACGTATACCAATTTATTGACGTACAACGTGTCGATCCTGCGAAGAAACCGATTAAAGTTCGCAAGATTGAATTCGTCGAGATTTACGAACCCTTTACTAAACAGCAAGCCACTGCCCAAGCTGATCGCTGCCTCGATTGTGGTAACCCATACTGCGAGTGGAAGTGTCCTGTTCATAACTACATTCCGCAGTGGCTGAAACTTGCCAATGAGGGGCGTATTCTTGAAGCAGTAGAGCTATCTCATCAGACAAACAGCCTGCCTGAAGTGTGTGGCCGAGTCTGTCCACAGGATCGATTGTGTGAGGGTTCTTGTACCCTCAATAACGATTTTGGTGCCGTCACGATTGGTAACATAGAGAAGTACATTACCGACAAAGCGTTTGAAATGGGTTGGAAGCCCGACATGTCACAAGTCGAATGGACAGACAAAAAGGTGGCCATTATCGGCGCAGGCCCTGCAGGACTTGCTGCCGCAGATATTTTAGTGCGCAATGGCGTCAAACCTGTCGTTTTTGATCGCTACCCTGAAATCGGAGGCCTACTCACTTTTGGCATCCCATCGTTCAAACTGGAAAAAGGCGTGATGGAAAATCGTCGCCGCGTCTTCACAGATATGGGCGTTGAATTTCGCCTCAACGTTGAAGTCGGCCAGGATATAACGATGCAGCAACTGCTCGACGATTATGATGCTGTTTTTCTAGGGGTCGGAACATACAAGTCTATGCGAGCTGGATTAGAAAACGAAGACGCGCCGGGCGTATACGATGCACTGCCGTTTTTGATTTCCAACACTTACAAAGTCATGAACTTAGAAGACACACAACCCTTCGTAGATATGGCGGGTAAAAAAGTGGTCGTACTCGGGGGTGGCGATACCGCAATGGACTGTGTTCGCACGTCAATCCGACAAGGCGCCTCTAACGTCATTTGTGCCTATCGACGTGATGAAGCCAATATGCCGGGCTCTCGTCGAGAAGTAAAAAATGCCAAGGAAGAAGGCGTCGACTTTAAGTTTAACCTTCAACCTTTGGGTGTCGAAGTGGATGTCTCTGGCAAAGTATCGGGTGTGAAAGTGGTTAAAACGGCCCTCGGAGAACCCGATGAAGCCGGACGTCGTCGCCCAGAACCTGTGGAAGGGAGTGAACATGTTCTGCCAGCAGACGCTGTAATTATGGCGTTTGGATTCCAGCCGCATAAGATGGAATGGCTAGCGCCTTATGATGTGGAGCTCGATCAGTGGGGAAGAATCAAAGCCCCTTCTGAACAAGAGTTTCAGTATCAGACCAGCAATGAGAAAATCTTCGCGGGTGGTGATGCTGTGCGAGGC

>NODE_97_length_2834_cov_2.338774

CATCATCTGCGTATCGACTTGCTCTTGCTTGAGTGTCTGTTGCATAAAACGGCCGAGGGGATCTTGCCCGACGCGACCAATAAAAGCAGCATCACCGCCCAATCTGGCGATGGCGACTGCCACATTAGCAGGCGCGCCACCTGGGCATTTCAAGTAGCTGGACTCGCTATCTGGAATCAGGTCTACAACTGCATCTCCTGTTACCCAAACTCTGTTTTTCATCATTGCATCTCTTACTTAACTAAATTAAGCTGATTGTATAGCTAAAACGGTTTAGCGATCTAGGTTTGCAGAGATCATTTATCACGATAAAGATCAAATTGTGATCATTGTCAGAGTCTAAACATGAGAGCGCAGTGGCGTTGATTGAGCTAGCAGCAAGGCAGGCTGATAAAGACACCGAGTAGGAAAAGAGAATAATGGCAGGTAAAAAACTGAAATTGGCAGACATCGCAGCATTGGCGGGTGTTTCCAAGTCAACAGTGAGTTTTGTGCTCAATGGACACGCAAAAAAGCACCGTATTCATGAAGACACGGTGGAGAAAGTACAGGCTATTGCTGAACAGTATCATTACAAGCCGAGTATCTACGCCAGAGCGCTCAAGTCTAAGCAGACCTATACCGTAGGCTTAGTCATTCCAGATTTAGCGAACATGGGCTTTGCGAACATTGCTAAACGGTTGGAAATACTGTGTCGGAATAATGGTTATCAGCTACTTATCGCCTCGTCCGACGATGATCATGAACAGGAACAACAGGCAGTAAAGAGCTTGATTGAACGGCAGGTGGATGTCCTGCTAGTGGCAACTTGCATGATGGATGACACCTTCTATCAACCTATCAGTCCAAGCACTCCAGTAATTTTCTTTGACCGTATATTAGAAGATAGCGATTTCATCAATATCAAAACCAATGCTGAAAAAGCGACAAAGGAAGTGGTGGCTCAATTAGCCTTAAGTGGTGAAGAGTGCGTGTATATCGGTGGTCAGCTTAACTTGTCTACGAGCCGAGATCGTTTTAAGGGCTATCAGCTCGGGCTTAAGGATGCAGGTGTGCGTTTTGATGATCGCTACGTGTATACCAAGGATTATCAACCTGAGTCAGGCTACGAGCTCATGGCACTGGCTGTAGAAGATCTAGGGCGTCTACCTAAAGCCGTGTATACCGCCTCGTATTCATTGTTGGAAGGGGTTTTGCGTTTTTTGACCGAGAAGAACATGCTGGATGAAGATATTCGCTTGGCGACCTTCGATAACTACGACATTCTGGATTGCTTACCTGTGAAAATTGACTCGGTTGAACAGGACTGCGATCTGATTGCTCAGACACTATTTGATTACGGACAAGCCATTCTAAAGCAGCCGGAGATGAAAACAGGAATTACGGTGTTGCCCGCCAGAATTCATCGTAGGCGCTAATCTCTTGATTGGTCTTGCATCTCGATATTTGCGGCTGCGAACCTTGATATCGGTGATTTGTCTTTGGATTGATACCAAAAGGCTGGCATTGCGTTGAGAAAGCCTGCTTGTTTTAAGACGTTCTTTTCTGCTTTGGTAATGGTCGCGTAATCCAGAAGGCTGTCGAGAATACGGCGTAATGCTCTTGGTGTGAGTAAACCATCACGCCAGTAACGTTTGATTTCTCCGGTGATCACTTCGTGCGGAACCAGCGGCTCAAGCTCAGCGTGCTTAATGATAGTAAGAGAGCGGGAACGAGGGCAGCGCTGGCGGGCGAGTTCCCAATCTGGCTTGCCTTTCTTAGTGAGGTAATTTTCCCGGAAGCAAAAATAGCTGAAATAGAATCGCTCGATGCTGTGCCAGCGTTCGGCTTTTACTTCCAGAAAAATATGTTCAAATTCAATATCGAGGTCTGTCATCGCTTCTCTCACTTATGGTCGAAGCGATACTACCGACTAACCCATGGCTACGGCAAGAGAATCTGTATTGCGTTGCTTTTTAATCGCCAGCAGAAGCTGAACAAGTAGCAACATACCTGTGACGCCTAGACCAATACCGATACCGCCCCAGATGCCGCCTAAACCAAAGTCTGGCAGTAGCAGCCATGCAGCAGGCAGACCAAACAGCCAGTAGCCGACAGCCGTCACTACAGTCGGGCCAACAACGATCTTCATGCCGCGAAGTAGGTTAATCGCCAGTAGCTGCCACGCATCAACAATGAAAGAGAGCGCGACTACCCATAGAACGGCGACGAGCAGTGTTGAAAGCTCAGAATCCAGATTGAAAATGGAGCCGATCAGCTCAGGCCATAACATGAACACACCTGCCAGCACTACGCTGATCACTGTCGTCAGGCCAAAGCTACGAAGGGCTGTGCGCTTAATACCATCATAGTCTTCCGCACCATAGTCCTGCCCGACCAAAATTGCTGCGGCTTGCGAGAAACCAAAGTTGATGTTCCAGGTGAAGCTCAGACATTGCAGAAGAATTTGGTGCAATGCGAGCGCAGCGATACTGATGGTGCCTGCCATCAAAGTGCCACCGTAAATCAAACCGTGTTCAAGCAGCGCAGCCAGTGCAATAGGCAAGCCCATGGCCAGTAAAGGTTTAAGCAATGATAGCGAGTATTCTTCTGTATTTTTCCACGGCGCAAACGGCATGTATTCAGGGCTCTTAAACACCCAGTAGCCGTAGCCCAGCATCACAATGAATGCCGCTAAAGCGGTACCCGCGCCAAGTCCTGCCAAGCCAAGCTCTAGCTGGAAAGCAAGCAAATAGCTGATAGGTACGTTGAGCGCAACCGTCAGTAATGACATCACCATGATTGAACGTACATTACCGAGCGCACTGGTTAAGCCGCGCAGCACCAGTAGCATC

>NODE_98_length_2833_cov_2.736938

CATTGGGTCATAGATACATAAATAAAATGCTATTGGCAGTATTGTAGCGCTTTTCGGAGGCAATTTTGGATAAGATTTTTGTAGAAGAAGCGGTATCAGAGTTACACACGCTTCAGGATATGATTCGTTGGACTGTGAGTCGTTTCAACGCCGCAAACCTATTTTATGGTCACGGCACTGATAACGCATGGGATGAAGCGGTTCAGCTGATCCTACCAACGTTGTACTTGCCAATTGATGTGCCTCCGCATGTATTAAACTCTCGTTTAACCAGCAGCGAGCGCCTGCGCATTGTTGAGCGCGTTGTGAAACGAATTAATGAGCGTACTCCAACCGCTTATCTAACAAACCGAGCTTGGTTCTGTGGTTTAGAGTTTTTTGTCGATGAACGTGTTCTCGTGCCTCGTTCTCCAATTGGTGAGCTGATTCAGGCAGAGTTCCAACCTTGGTTAGTCGAAGAGCCAACACGAATCATGGACCTATGTACCGGGAGTGGCTGTATCGCTATCGCCTGTGCACATGCTTTCCCAGATGCAGAAGTTGATGCCATTGATATCTCGACAGATGCACTACAGGTAGCCGAACAGAATGTTCAGGACTATGGTATGGAGCAACAGGTATTCCCGATTCGCTCAGATTTATTCCGTGATCTGCCGAAAGAGAAGTACAACCTGATTGTGTCTAACCCACCTTATGTGGATCAAGAAGACATGAACAGTTTGCCTGATGAGTTTACTCATGAGCCTGAGCTTGGCCTCGCTGCGGGTACGGATGGCCTGAAACTGGTGCGTCGTATTCTGGCTAATGCGCCAGATTACCTAACGGATAACGGTATTCTTATCTGTGAGGTAGGTAATTCTATGGTTCATATGATGGAACAATATCCACATATCCCGTTCACTTGGCTTGAGTTTGAAAATGGTGGTCATGGCGTATTCATGCTGACGCGTGAACAGATGCTGCAACATGCAGAAGAATTCTCAATCTATAAAGACTGAATATCATTCAGTTCTCTTGATTTGATAAAACGCCGGACAGCCCTGTCCGGCGTTTTTTATTGGTAAAGAAAAGCAAAATAGATCGGCTTTAGGGCTTTACATCAAATCGCAATAACGCCACTATGAATTTACGAACAATTGTAATGTACAGCTTTGTTTACAGAGCTGAAGAAATTTGAGGAAGTAATGGCAGGAAACAGTATCGGACAGCATTTCCGAGTGACGACATTCGGAGAAAGTCACGGTATCGCACTAGGATGTATCGTCGACGGGTGCCCTCCAGGGCTGGAAGTAACTGAAGCGGATATCCAGACGGATCTAGACCGTCGTCGTCCGGGTACTTCTCGTTATACCACTCAACGTCGCGAACCAGATGAAGTGAAGATTCTGTCTGGCGTATTCGAAGGTAAAACCACAGGTACTTCGATTGGTCTGTTGATTGAAAACACAGATCAACGCTCAAAAGATTATTCGGAAATCAAAGACAAGTTCCGTCCAGGTCACGCTGACTATACCTACCACCAGAAATACGGTGTGCGTGATTACCGAGGCGGAGGCCGCTCTTCCGCACGTGAAACCGCTATGCGTGTAGCTGCGGGTGCTATCGCTAAGAAATACCTGAAGCAAGAGTTTGGTATCGAAATCCGCGCTTACCTATCTCAAATGGGTGATGTGTCGATTGAGAAAGTGGATTGGGACGAGATCGAAAATAACGCGTTTTTCTGCCCAGATGTCGACAAAGTCGATGCGTTAGATCAGCTGATTCGTGATCTTAAAAAGCAAGGCGACTCGATTGGTGCCAAACTACAGGTTGTCGCAACCAACGTACCTGTTGGTTTAGGCGAACCTGTATTTGACCGTCTGGATGCTGATATCGCTCATGCGCTGATGAGCATCAACGCGGTGAAAGGTGTTGAAATCGGCGATGGATTTGATGTTGTTAGCCAAAAGGGCAGCGAGCACCGTGATACTTTGTCTCCAGACGGTTTCGGTAGTAACCATGCTGGTGGCATTTTAGGTGGTATCTCGACTGGTCAGGATATTGTTGCGAACATTGCCCTAAAGCCGACGTCTAGTATCACCGTTCCGGGTGAAACCATCACCAGAGAAGGCGAACCTACTCAGCTGATCACCAAAGGTCGTCATGACCCATGTGTTGGTATTCGTGCAGTGCCTATTGCAGAAGCCATGCTGGCAATTGTCGTGATGGATCACTTACTGCGTCATCGTGGTCAGAATCAAGGTGTGACGACAGAAACGCCTAAGATTTAGTGAATAGAAACGGCTTCCACAAGGAAGCCGTTTTTATTTAGGCTTGTTGTTTTGTTGAAATCCAGTTGTTGATGTTGCTTTTCATCACTTCCATCGGTTGTTGCCCGTAGCGTAGCAGTTGGTCGTGGAATTCAGCGTATTCAAACTTATCTCCTAGCGCTTGTTGAGCCTTTTCAAGCAATTCTCGGATAACCAGATAACCGGACTTATAAGAAACCGCCTGACCGACATAGGCGGCGTAACGGAAGCTTTCGGATTCTATGTCACCTTCGCCGAGCGCGGAATTGGCGTGCATATAATCGCGTGCCTGCTGAATACTCCAGCTGTTGGCATGAATGCCGGTATCGACCGCTAATCGCATATTCCTCAGTTGTGCTTCGTTTAGACTGCCAAAGTACTGTAGAGCATTACATTCCTGCTCATCCGTATAGATACCGCCTTGGAACTGGCTATAGTCAGTGTCGGAAGTGCACATGCCTTTGCCGTTAACAAACGTCGGCTGATTCTGCTCGTTAAGCTCTCCATAAATGCCCATTTCGATACCAAGCCACTCGGTATAGAGC

>NODE_99_length_2820_cov_2.553773

CGATCTGACAGCGACATCAGCATCTGTTTAGCATGTTCCAGATCATCGGGCTTTTCCAGAACGGTGTTGTCACAAACAACGATGGTATCTGAGCCAATGACAACAGCTTCACTTGCGAGCGCTAACCCCGCGAGCGCTTTATCTTTAGATAGGCGTGCGACGTACTGCTTTGGATTCTCATCTGACGATTGACTTTCTTCGACATCGGTTCGAATAACAGTAAACGAATGACCTAATTGTGATAACAGTTCTTTGCGTCTCGGTGATCCTGACGCCAACACCAACTTTTTCATCATCAATTACCTTACATGCCAATGTCGTCTGACTCTGCGCATCAACAGGAATAACCACGGCCACAGAATACAGTTTATCAAGCCACTCCACAGAGACAGCAGATTAAATGTAATGTCCTGTATCAGGTATTCTCCTGAGAAAATCAGAACATCCAAAACAATAGAGAGCAGACCAATTAGCATCGCTTGCTGCCAAAGGGCCATATTCCTCAAGACCAAGAAATTAAGCGCCACCAGATAAACGACGATCGACATCATCATACCGCGAATACCCAAGGTGGAACCGAGCAATAAGTCCCACAGCAAGCCAAGTATCAAAGCGGTACCGACATTTACTCGATGTGGCAATGCCAACACCCAGTAACAAACCACAAGAAACAGCCAAGAAGGTCTTAACACATCCAATACGCCAGGCCATGGAATAGTCTGAAGGGTTAAGGCTATTAAAAACGAAAAGGCAATGACCATTTTGCCTTTAAATACACTATTCGCCATTACCGCTTACCTCTTGCATATTACTTGGTAATGCCTGTTGAATCTTGTCCTGACGAGAATCATTTGGCCAGATGAGAAGCAAATAACGTAGGCGTTCAAAATCGACAACCGGTTCAGCTTCTATCGCTGCGAATTCACGCGCAGTGTCACGGACAACATTCACCACGTGCGCCACTGGATATCCTTCTGGGTACACTCCCCCCAAACCGGAAGTAACCAGTAAGTCGTCTTCCTGAATATCGAGGCTGATCGGAATATGATCTAAGTTGATTCTGTCTATCTTGCTGTTGCCTGACGCGATAACACGGATGTCATTACGAATCACTTGGACAGGAATAGCACTCTGGCTATCTGTAAGTAACAATACGCGGCTGTTATGGGCGGAAACAAAGGTTACCTGACCAACAATCCCTTTTTCGTTCGCCACAGGCTGGCCGACATAAACGCCATCTATCTGGCCTTTATCAATAACTACTTGCTGACGATATGGTGATGTATCCACGGCCATCACTTCAGTGACTACTTTACGCTCGTCTCGTACAAACGAGGAGCCCAGCAGTTTACGCAAGCGCTGGTTTTCTTCACGATACTGATCCAGCAGCAACATATCATTCTTAAGACGCAGAATTTCACGCTTAAGCTGATGGTTACTCTCCATAAAGTCTTTGCGAATATTGAAACGCTCATAGACCCCATCAAACATAGTGCGAGGAACATCAGCTAAATACTGAATCGGGGCGACCATGCTGTTGAGTAAATAACGTACCTGAGCAAATGCGCCTAAACGACTATCAGCCAGCATAAGGCTGGCTGATATAATGACTGCGAAAAACAGGCGTAATTGAAGTGAAGGTCCTCGGCCAAAAATCGGCTTCATTGAGTATGAGTTCCTTAATCGCTCGAAATAGCCTAGTGGTTTGCTAGGCCAATTCGATTATTCTTCGCTGAATAGATCGCCACCGTGCATGTCGATCATTTCTAGAGCTTTACCGCCACCACGTGCTACACATGTCAGTGGATCTTCCGCGATAACCACTGGAATACCAGTTTCTTCCGTTAGAAGTCGATCAAGATCTTTCAATAGCGCGCCGCCACCTGTAAGAACCATACCATTTTCTGAAATATCAGACGCAAGTTCTGGTGGACATTGTTCCAGCGCAACCATTACAGCGGAAACGATACCAGTCAGTGGCTCTTGTAGCGCTTCAAGAATTTCATTTGAATTTAGGCTAAAGCTACGCGGCACACCTTCAGCAAGGTTACGACCACGTACTTCAATTTCTTCAACTTCATCACCTGGGTATGCAGAACCAATCTCGTGTTTGATCTTTTCAGCGGTTGCCTCACCAATCAAGCTTCCGTAGTTACGACGAACATAGTTGATGATCGCTTCATCAAAACGGTCACCACCAATACGTACTGAAGATGAGTAAACCACACCGTTTAGCGAGATAACCGCCACTTCCGTAGTACCACCACCGATATCAACGACCATAGAACCCGTAGGCTCAGAAACACGTAGGCCAGCACCGATTGCTGCCGCCATTGGCTCATCGATCAAATAAACTTCACGCGCGCCAGCACCAAGTGCAGACTCACGAATAGCACGACGCTCAACCTGAGTTGAGCCACAAGGTACACATACCAAAACACGAGGGCTTGGTTTAAGAACACTGTTGTCATGCACTTGCTTAATAAAGTGTTGCAGCATTTTTTCGGTAACGTAGAAGTCTGCAATCACACCGTCTTTCATAGGACGAATTGCCGAGATGTTACCTGGAGTACGGCCCAGCATTTGTTTTGCGGCATGGCCGACAGCAGCGACACTTTTGCCTGCTCTGTTACGGTCCTGACGGATAGCGACTACTGAAGGCTCGTCTAGGACAATACCCTGTCCCTTGACGTAGATAAGAGTGTTGGCAGTACCTAAATCGATTGATAGATCATTAGAAAATATGCCACGAAGTTTTTTGAACATATTCTTCGCTCGTCCTGAAAGAATTAGAAGATAGAAAATTGCACTAAATGTACC

>NODE_100_length_2817_cov_2.433942

CCATAAACTCCTCCATCAATCAAGCGGCCGTTCACCGTAGTAGCAGATACAACGTTAACGTTTTTCTCTATTATTGAACCAGGATTAATCGTACAGTTAGAACCAATATAAACACCGCTACCTATCCGGATACTTTCTAATTTGATGAATCTTGTTTTTCCCTCATTGACATGAAGAAAACCATGTGTCCAAAACTTACTATCACGTCCTCCAATAACAGAAAAATCTCCCACGTAAATTGACTCCGACAAATCAAAATAGTGAGAGCTGGTTATTATACTGCTATCTCCAAGAACCAAAGAGCTATTATGTTTGAAAACGCTTCGCCTAGGGCCGCGGAATAGTACAAAATTTGATATTCTCGCGCTTTCGTTTAGCCTAAGGGAAAAATTACCTGTTATTTGGTTGAAGTGGCCAAGGGAAGCATTCGATTGTAGTTCTAATTTCCCTCTAGAAGTGATTAAGTTAAAGTGGCCTATTGAGGCACCAGCTTCTAAATCTAGATCTGTATTAAGAATTAAACTAAAACCAACCTTGGCTGAAGGTGATACTTTAGCCCCCATCAATCGAAATAAATGGTTTTTAATTCTACTTTTAGGCACAAAAAGAGATATAATAGCGAAAAATATTTTATGCATACACTAGGACTCTTTAAGCTTTTTCAATAATTGCCCAGCCACAAGCTTAACATTTGTATCAAGGGTACATGTTTTAGAGTAAATAGGGTCAGATATTTCACCAAAGTATGAGTTACCTCTAGAGTGGGTAATTATTCGTTCAAGAGGAAACTTGACCTCAGCTGGCAAAATCTGACTGTCTTCCACATAATGCGAATTTGGGTAACCATAGAAGAGAGACTCTTTCTCAGTAATCAATGCTTTTTGGGCCAAAATTGTTTCTATTCCGACGGTTCCAGTAACAGAATAAACTCTACATACAGAATCAAAAATATCTGTCATTTTTGTTTTTTGGCTGAGACATACAACACGATCGCAACTTTCTACATAATCTAAAAGCTCATCAGAAAGCTCATATTTTGACCTCGGATTCGGCTTAACAATAACTTTTTCGCCTTCTTGCAATACTGATACTAGTCTCTTTAAGTTCTTAAGCTGATCATTGTGAGGATAGCCCCATACATCCAAATTTGCTTCAGGTTGCATTTGGAGTGGGTACAGGATGCCGTTAACAAGATCATCATGGGATACTTGATCCCTTTGAGCGCACCTTTCAAGACGAGACTTGTTGTTAGCTAGCATCTTTTGAAACTTATATTTAACAACAGGGCTTGGCGTATTGTAACATTCCCCTGTTAAACGCGAACGCAATGAAGTGTATGTATTGTAGAGTTTATATATTGTACCTCTAACGGAAGTAAAACCTATGGAGAAACTCATATAACTAGGAACAATACTTTTATTAACTATACCGTCAATGATCGCCAGATCTTCATCATTAACTTCAAACTCCTTTTCGTCGTAGCTATTGAGCGAGTCAGAGTCTAACAGGGCAAACTTGCCCAATGGATAACGAGTGGGGGAAGGGTGCAAAAATTGAATATTGAGCTTTTTACACGCTTCCACAACCATTTGCTCGTGAAATAACGTCACTTCGCCGATTACAAAATCCGGTTTCTCGTTTTCTAAGTATCTATATATAACATCGTAATAATATTTATAATGGTCAACACGCCCGCCATAAATATAAACTGATCGATCTCTTTTTTCGAGATCTGAAAAAAAAGGAGTTTCGCAATATTCTAAATCCACTTTTTTTGGATAAGGTATATTAAAACAGTCGCTAGTCGGAGAAAAATAATGGTTTTGAGCTAGCCATCTAATTTCAACATTATTATCTAGTTTTTTTATTTCACTCGCACACCGTGCCCAGAACACAGTTTTAAACCGGTTCTCTACAAACAAAACTTTCACGTCAAACACCACTTTTATTCTTAGATAACAAAAACGAACCTAGCGATATAACAGAAATACAGAGTATCTGAAATATACCTAGCCCACCATAGAAAAAGTTACCTATCGGGCTCAGCATCATGATAACTAAAAAAACAAAAATATCTACGCTAAAATATTTAACAGATAAACTTAACGCAAAAACAAATAAGAATATAGTTCCTAATATAAAACTAGCGTAAAAAACCCCGAAGTCATAAATAAAGTCGAAATATATAGTTTTGTAAACACCTGCACGATAGAAATCTTCATCTAGTGTACAACCAAGCACTGAACATGTTGCATTTTTAGATATTTGACCATTAAGATCAAGTATATTAAGTATGTCGGCTGCTAAATAAGTCGAAAACTCACCTATAGAATGGTAAAGATAATGTGACAAAAATACATATCCTTTAAAATACTCGAAGCCATCAAGAGCTATTACCCAATCACTAATTCCTACGATTTGCGTAGATATGGTACTTTGTAAGTGCACACTCCAATCAAAATCTTCCGCATTAAATGCTTTCATTTCGGTCGTTTCTTTGAATATCACAACTATTAACAATACAAATATGATCATTAAAAACCAAGACAGCTTGCTTTTTAGCATTTTTTTCACATCGAAAGTAAAAACGATCCAAAATAACAGAACAAAGGTAGGTGTCATTCGCATTGCAAGAAAAAGTGCATCAAAAATCAAAAATCCAAAAATAGCAAAATAGATCAAATGATGAAACTTGTATATTCTATTGAGGTAAAAGATTATAATACAGCTTGGGTAAAAGAATATAGCCAGATAAGAAAACCAACCTCCCCCGGCTGCATTGTTCCGAATATCATAGACAGATGAGTAGTCAAAAAA

>NODE_101_length_2815_cov_2.331629

ACCATGAGCAATGCGACAATACCAAGGGCAATGGCGCTGGTATATCCGCCAAGCACGCTGACTTTACCTGTACCGAACGAGAATCGATCGTTGCCGGCATTTTTCTTCGCATAACGATAGGCAAACAGAGTGATACAAAATGCCGCAGCGTGAGTTCCCATATGCCAGCCGTCAGCCAGCAATGCCATCGAGCCGTAAACCGTACCTGCAACAATTTCAGCCACCATAGTCGCCAATGTCAGCAGTAGCACGTAAAACGTTCTCTTCTCGCCTTCCTGATTATGTGAAGAAAAATCGTGATGATGTTCGGTGGCAAATTTCAAAACTGAACCTTTAGTTTGATTATCAAATTATTTGAAAGTTAATTTAGTTTGACTGTCAAACAAAGTCAACCTTAAAAAGTGCGCCAGACCACTCTCTCACTTATATGAACGGTAACGTACAGCCAAATAAAACAAAAACACCGCCACTGATGCGATTGAACGTGCCACCATGATTATGAAGAAAGCTCTGCATATGGTGCGCGAGCAGTACATAGCCATACTCAACCGCAAATTCCACCACGGCAAAAGTGACCGCTAAAACAAGGATTTGTGGCGCCAATGGCTGTGCTGGGTCAATGAACTGGGGCAAAAACGCAACGTAGAACAACAGAACTTTAGGGTTGGCTACGGCTGTAAAGAAACCGCGTCGAAACAGGCCTTTACTCGCTTGAACATCATTCTCTTCACCTTCACTTTTCGTCAGCGTGCGTGTTTTCACCAGCCCAATGCCCAACCAAATCAAATACAGCGCACCTGCAACCTTCAAACCATTAATGACAGCGGGAGAGAGTATGGCTAAAGCGCCGACACCAATGCTGGCAATCAATATCAGCCCGAAAAATCCGGTAATACCGCCAAGCACTGTGTACTTAGCAGCGCGGCGACCGTAATTTACCCCGTGAGTCAGGGCGAGAATGCCATTCGGGCCGGGGGCTAAGCTCACCCCAATCACTGCTACCAGATAGACAAGCCACATCTCAAATTGCATTACATTCTCCCATTCATTGTTCACTTGATAGCCTGACGATAGCGTCAATCTTGTATTGTGACTTGGTAAAATTTGGACGTAATACGTGAAAATATTACGTGGTCGTTTATAGTGGTAGGAAGATTGCTTAGCGGAATACACTCATGATCACCACTCCGAAAGTTCGATTTTTAATCTTGCCCTGCGAAAGCTTTTCCCTGCTGGCATTAGGAGGGTTTATCGACAAACTGCGCTTTACCGCAGACGAAGCCGATCTGAGTGAACAGAAACTGTGTTCGTGGACAGTGGTAGGCCATGAGCATCGTCAGCTTGCCAGCTGTGGCATTCATATCGAAATCGATCACCTGATCGATGACATAGAGCTTCAACATTTTGATTATTTAGTTTTGTTTGGCTCACGCACCGCCAGACAAGCTCATCAGTGTGCCTCCCTGTATGCGAGTTGGTTGCGAAAAGCCGCCGCTCACAACATGCCGTTGGTTGCGATTGATAACGCAACCTTTACCCTTGCCGAGCTTGGCTTACTTGAAGGGTACGAAGCAACGATCCACTGGCGCCACGAGCAAGAGTTTCGTGACCTGTACCCTAAGATACAGGTGCATTCAGACCGATTATTCAGTTTTGATCGCAAGCGAATTACCTGTAGCGGTGGCAGTGCCAGTGTCGATCTGGCGGCAGAGATCCTCTCGCTTCACTTTGACAAATCTCGTGCGCTGAAAGGCCTAGCAGACATGCTGATTGATGAGAGCCGAGAACCATTCCACCAGCTCCGTTCAGACAAAGATGAGCAATATCATCACCCCATTCTTAACCGAGCTATCCGAACCATGCGCCAGCAACTGGATAGGAAGCAGACCATTGAACAGATAGCCGATTCCGTAGGCCTGAGTCGACGCCAGTTGGATCGCCTATTTGAGGCCCAAACTCAGCAGAGCGCGCATCAGTACTGGCTTGAAATGAAGCTTCAGTATGTCTACTGGCGACTGCTCAACTCCAGCACACCACTGACCACAATCGCCGATGAAGTCGCGATCTGCGATACCAGTTATCTGAGCAAACTGATAACAAAACGCTTCGGCGACTCACCGAGTCACATCCGCTCAGGTAGACAAAATAACGCCCTGGGACAAATCAGGGCGTACAGGGAAAGTTAGAGGCGCAAAGTTCACCGTTAAAACTCGTCACTGACCTCATAAGAATCCTGCAAAGTATGGAATCGTATTATTCGGTAATTCCGCGAATTCGGGCATTGCGCCTGCGTAACATTTCCAATGTCAGCAACAGCATAACGGACATCAAAATAAGCAAGCTGGCAACGGCCAGAATGGTTGGGCTAATTTGTTCTCGGATACCTGACCACATCTGACGAGGAACGGTTCTTTGTTCAACACCAGTGATAAACAGCGCCACCACCACTTCATCAAACGAGGTTCCAAACGCAAACAGGCCGCCGGAAATCATTCCTGGGCGAATCAGAGGGAACGTGACATGGCGAAAGGTATAAACCGGACTCGCGCCCAAGCTGTAAGCGGCTCTCACCAGTGAATGATCAAACCCACTAAGTGTGGCGGTCACGGTAATCACCACGAAAGGTGTCCCAAGTGCCGCGTGAGCAAGGACGATTCCGATAAAGGTTTGAGATAAGTCGAGCCGAGTGTAAAAGAAGTACATTGCGGCAGCGGAAATAATCACTGGGACAATCATCGGCGAAATCAAAAATGCCATGATGGTATTGCGAAACGGAACATTGTTAGCTGCCAGTCCCATCGCAGCCAAAGTACCG

>NODE_102_length_2813_cov_2.420687

CCGTCAACTTCTAGCGCTTCAAGAGCCGCTTCAGTTGTCACTTCTTCGCTGTCTGCACTTTCAGGAAGTACATCTTTGTTACGCGGGCCAGAAACAATCTGCTTGATCTCACGAGCACCAATTTCAGAACGTAGTACGTCGGTAACTTTACGTTCAAGTAGCGCTTCCGCTGTCAACGTATTCCCACCACCAGTGGTTAGGTAGTAACGCCCGAAGTCTGAGATACGCCACTTAACGTAAGTATCGATCAGCACGTCTTTTTTCTCAGACGTTACGAAACGGTCAGAACGGCTATCCATTGTTTGAATACGCGCATCCAGAGTTTTCACTCGATCGAATAGCGGCATCTTAAAATGCAAGCCTGGCTCGTAGATTTTTGATGCGCCGTTATCATCAAGAACACGACCAAATCGAATCACCAATCCACGCTCGCCTTCTTGAATGACAAATACTGACATTAGCAGTAGAACAATGGTAACGACTAATACAGGGATCATTAACTTACGCATTCTTAATATCTCCCTTGACGTGAACCAGTTGAACGAGATTGCGAGTCTGAAGTTGTCTCATTTTTCTGAGTTTCTAGCTCAATTTGATCGTATGCAGAAGAGGACTTCGTGCTACGTTTGGTTTGAGTCTTACCTTCTTGACCCGCCAGTTTATCAATAGGCAGATACAGAAGGTTACCGCTTGATTCCGAATCTATCAGGACTTTAGACGTGCTTGAATAAACGCGTTCCATAGTATCTAGGTACAAACGGTTACGAGTAACTTCAGGCGCTGCTTGGTATTCAGGAAGTAGTTTCTCAAACTGCGCTACTTGACCAAGTGCTTCATTCACTACGCGCTCAGAGTAACCTAATGCTTCTTTCTTCAGACGCTCAGCTCGACCCGTCGCTTTTGGAAGAATATCGTTACGGTAAGCTTCTGCTTCACGTTCAAAACGCTCTTCATCCTCACGTGCTGCGATAGCATCGTCGAATGCGTCTTTAACTTGCTCAGGTGGACGCGCAGACTGGAAGTTGACGTCAACAATGACAACACCCATATCGTAGCTATCTACGATTTCATTTAACGTTTCCTGAGTACTTTGACGGATTTGCTGACGACCAGTCGTCAGGATGCTATCCATCAGAGAGTCACCAATCACTGCGCGAAGCGCAGAGCCGGTCGCTTGGCGTAAGCTATCATCTGCGTTCGTCACGCGGAATAGGGATTTGTATGGGTCAGCAACACGGTACTGAACATCCATCGCTATCGTCACAACGTTTTCATCTTTAGTCAGCATTAGACCAGATGAACGCAGTGAGCGAATTGCCTGTACGTTGACAGGAGTAACTTCATCAATGAAGCGAGGGCGCCAGTTCAGACCAGGATCTACGACGCGATCGTATTTACCCAATCGTAGAACAACACCACGTTCGGCTTCACCGATGGTGTAAAAGCCCGCAAAGAACCAGATTGCTATCGCGATTACTGCAATGACACCAAAACCTAGTGCACCACCGCCGCCACCGATAGAAGACCCTTTGCCTCCTTTTCCACCACCAAACTTTCCACCCAGTTTCTGACTCAGTTTATTAAATACTTCGTCTAAGTCTGGCGGTCCTTGATCACGGCCACCTTTGTTACCACGATTATTACTCCCCCAAGGGTCGTTATCGCGGCCATTGTTGCCGTTGTTATTACCAGGCTCATTCCACGCCATTAGAAAGCTCCATCATTTGATATGACGTTATACTGTAGCAGTCATTTAAGTAACTATAAAGTCACGTAAAACTGCCCCTTCTCTTTTTTCAAGTCTAGACCAATCCACTTGTTGCATGCGAACATCAATCAACAAGTTACCATCCTGATCATATTCTTCTCGTTGAATACATTTCATTTGGAAGAATGTACTACGAATGCGCCCCTGATGTTGTGGCGGTATTCGCAATTGATACTGAACCACTTGACTAAGTAAGAGCCCCGACAGGGCGTAAAACAGCAGGTCAAAACCAAAACCATACATTGCCGCAAACCCAAACGGCACGAGGAATACCTTCTTCGTCCCTTTCAATTCGAGGTTTCTGGTCTTCGAGATTATCAATTTTGTTCATTACGAGAAGAGCAGGTACCTCATGGGCACCAATCTCTTCAAGCACTTCATGAACAGCATGAATATTCTCACGAAAACGCTCATCACTGGCATCGACAACATGTAACAAAATGTCAGCTTCCTGCGTTTCTTGCAGGGTCGCTTTAAAAGCGGCGACTAGGTCATGGGGTAAATGTCGGATAAATCCAACCGTGTCAGCCAAAATGGCTGTTCCCACATCCGCAAGCTCAATTTTTCTCAACGTTGGATCCAAAGTGGCAAACAGCTGGTCTGCTGCATATACGCCCGCTTCGGTAATGCGGTTAAACATGGTGGATTTACCCGCGTTGGTATAACCAACCAGAGAGATAGTTGGGATTTCCGCTCGATTTCTGGATCGGCGACCTTGTTCACGCTGCTTTGCCACTTTTTCCAAGCGGCGCAAGATAGCTTTTATACGGTCACGCAAAAGACGTCGGTCAGTTTCTAACTGGGTTTCACCTGGACCACGTAAACCAATACCACCTTTTTGTCGCTCAAGGTGAGTCCAACCACGAATAAGTCGAGTAGAGATATGGCGAAGCTGAGCGAGCTCAACCTGCAATTTACCTTCGTGTGTTCGGGCACGTTGAGCAAAGATATCGAGGATCAGACCGGTACGATCAAGGACTCGACATTTACACAACGATTCTAGGTTACGTTCTTGGGCAGGAGAGAGGGCGTGATTGAATATCAC

>NODE_103_length_2805_cov_2.471041

ACATAGAGGCGGCTGAGTGCGTGTCGATCGAGCGTCAGAGTGACGGTGTCACCAGAGGCAAAGGGGGGCTGTGTCGCCGGTAGGCTGAGCACCGTTATCGGCGTAAGCCCCAGCAGCGTCGCTATCAGGAGGCGAGAGATCATTCTCTGGCCTCCTGATGTATTGCCAACAGACGGATGGTCCCTTGGTGATAATCCATTTTGACGTGCACGTTCACCGCTTGTGTCGGGAGCTGACGCTCTCCGACGGCTTTGCTTAATATGCCTGACACTTTGACCTTCAGCGCGTTGATATCGACCTGGGTTTCATCCGGTTGAATATCTAAGTGACTCGATACATTTTGGTGTCGAATTCGCTCGGCGTCTTTTAGCAGTGTGGGTTGGACAAGCGGCCATAAGGAAGCGGGGATATAGTCAAGTAATCGCCCGTATTGACGGTGTACATTACTGGGTGTAACGTCGAATTTGAGGTGCAGAAAATAATCACTCATCATGGTGAGATACGCATTGTCTACTTGCACTTGGCTCAATGTAAAAGCCCCGGATACTGTCGGTGGTACGATGGTGTAGCGTTGGTGAGACAACGCATAGGCCAATCCAGCGGCTAAGGTGACGCTGACAAGGCTCAATGCGAGAAAACCCACGGCAAGAAAAACGTTGAGTAGTCGGCTGATTTCCAAAAACTGGCGTCGGATAGCGCGGTCCATCTCTGACTCCTATGACAACCAGTACCGATACTCGGCAGGGGGAGTGAGGGTAAAGAAGAGACGAGAAAGTGGCGCTATGGACCACCAGTAGCCCCACCGTACGAGCACAGTTTGACCATATTGGACCTTCAGGTAACGCAATCCCAGAAACCAAAGTAACCCAAGAGCTCCACCGAGCAGATGATGGTGCGCCGCAAAGCATATCCCAAACCATAATAACGCCGGGGCCACTTCGTCACGCGGTAAACCAACGAGTCGGGGACCGTGGTTGAGGTGCATTGGAATGCTGTAAAACCCTGTGTTGATTGTCATGGTGTATGTCCTCTACGATAGTCCGACTAACGGCTTGACCACTTCCCAGAAGATCATGCCACCAATAAATCCACCCACAGCCCCCATCCAATTCCGCGTCATAAACCCGGCCGACACCGCCCCAATTGCACCTGATGCAAGCAGGGCATTTTCAACCGCGGAGCCCGAACCGGCGGTGTCTTTGATGGTTTGTTTGCCCGCACTGAAGAGGTCAGCCCCTAGAGCACTATGAATGGCGAGCAACACCAGCACCACGCCGGCCGCCATGAGCAGAAAGGGTTGCCGCGTTCGAAGGCTTGATGAGTGGCTCTTCTCTGGTTCCCGCTTGACGGTCCGGTTGCGTTGTTCGTTTGACACCCGTATTCATCCTGTCGGTCGGTGGGCCAGCTAGAGTGCTGGTGTGCTACCAGTTGAATATGATGAAAACAGGCAGAGTTTGAAAATTAATAGAGATTATTGGTGATTATTAGTGTTTGTTGGCGGCGGGGATCCCCGTCTTATCGTATACACTGGTACATGATAGAGCACAGGCGAGGCCACACACTTCGCGAAATAGGCTCTCAACCATGGTGGGTACAAGACTTTGGACTTATACTGTGCTCTCTAACCGTAGCCCGCTGGCCACAAGGATGTTTTTCAAAATCGTCTGAGATTTTGATAAAGAATGATCGCACTTCATGAAGATTCACAAAGATTTTGAGTATTTTTACCGTTTAAGTACCTTGGTGACGGATGCTCTGCTGCATTTGCTGTGCCGCGTCGCCGCTTCCCAACGACACGTCCCCGTTGCCAAACGCAATGAGATATTGGTGAAGTTCCTAAAAAAGAAGCTCAATGACAAGGCTTTCGCTAACATCAAGAAAGACATCAAGCTGATGATTCGAGTTGGGCGAGACAAACACGGTAATCTCGAGCAGAAATTGTATGAATTGCACGATCGCGCCAGTCGAACCAAAGTCGGTGGCGTCGAGCGTCTGTATCAACTGCTGAATACGCTTCACGACGAAAAAGGCATCGATTCTCGTTTGTTCGAAGAAGGAACCGTGCCTGAACCTGGCGTGGTTTACATGTTAGAGGAACACATCGAAAACGGATTTGATGGTGCGAACGACCAAGTCGCCCCCATTTCGTTGTTATACCAATCGGAAGATGCCAAAGAGCTGGCGTCGTGGGTAGACGAACGCAGTCAGTTTAGCTCCGAAATCAAAGAATGGAATGAAAGCAATCATCAAATTCACATTGTATTGCATCCCAAAGAACGTCGACCGACCGCTGATGTTACTTAGGCGACACTGAAAGGTTGTCAAAACGCGACAGTTCGCCGTGGAATGCTAATCGTATCGCACCAACCGGGCCATTGCGCTGTTTGCCTATGATGATTTCAGCAATTCCTTTTTGTTTGCTATTGGAGTCGTAAAGCTCATCACGATAAATGAACATGATCACATCCGCATCTTGCTCGATGGCGCCTGAATCACGCAAGTCAGAGTTGATTGGGCGCTTGTCTGGACGCTGCTCTAGCGAACGATTGAGCTGTGACAAAGCCACTACAGGGACGTTGAGTTCTTTGGCTAGCGCTTTGAGAGCACGAGAAATCTCCGCAATCTCTAAGGTACGATTGTTGGCTAATTCAGGGATGCGCATCAGCTGCAGATAGTCGACCACGATCAAGGACAAGCCACCGTGCTCTCTTGCTAAACGTCGCGCTTTGGCTCTCAGCTCTGTGGGTGTCAGGCCTGCACTGTCATCAATCAACAAATTGTTTTTCCACTGCAGCATCTCCAGCG

>NODE_104_length_2800_cov_2.390011

CTGTTTGCTTGTGAGCCCGAGTGAGGTTGAACGTTAGCGTACTCACAGCCGAATAGCTGACATGCACGATCAATTGCCAAAGCTTCCGCTTTATCAACGTATTCACAACCGCCATAGTAACGCTTGCCAGGGTAGCCTTCAGCGTATTTGTTTGTTAGCTGAGAACCTTGAGCTTCCATTACACGTGGGCTCGTGTAGTTTTCAGAAGCGATTAGTTCAATGTGTTCTTCCTGGCGAAGAGTTTCTTCTTGGATTGCTGCGAACAGTTCCGCATCGTAATCTGCGATGTTCATATCACGCTTTAGCATCTGTATCTCCTGACTCAGATTTAAACTGAAATTTGCAAAAAACCTACCAATGACCCTATTAATCTACGTAAATTGATTCACGCAAACGTTTGCATCACGTTAATAGCGCGCATTCTACATAATTTGATCTTGGCCATAAAGAGAAAGTTTTACTTTTTCTCATGCAGATTCTTCATGTGGTTCTACAAGGATTGCCATATTGTCACCTTTCTAAATCGTATAAATATGAAAAGTGTCAATTTTTCCCTTTACACCCCGTAAAACGATAATTACATAGGTTTACCTTAAATTTGCTTCTCAAGCTACCGAAAACCTCATTTTTTAAATAGTATGCGCGCCATTATCTACTGTAAAAGTTTCAAATTGATGGAAAAACACTCACGCAAAGAAGATTGGATTGCTATTTTAACTGGAACATTTCTGGTCGCTCAGGGCGTGTTCTTCCTCCAATCTGCAAATCTGTTAACCGGAGGCACCACAGGTTTGGCCCTGCTTATGAGCCAGTTTGTGTCACTTTCCTTCGGTACGCTTTACTTTTTGGCCAACAGTCCTTTTTATTTACTCGCGTGGAAGCGTTTTGGCTCAAGATTTGCCTTCAACAGTGCTATTTCTGGTGCCTTGGTTTCTGTGTTTGCAGACCACCTATCTCTGGTCGTCAGCCTTGAAACGGTCAACGTTGTGTATTGCGCGGTTGCCGGTGGGTTACTGATGGGTTTGGGTATGCTTATACTGTTCCGTCATCGCTCAAGTCTCGGCGGTTTCAACGTACTTTGCCTGTTTATTCAGGATAAGTTCGGCATTTCAGTGGGCAAATCTCAGCTAGCCATCGATTGTTGCATCTTGTTCGCTTCATTCTTTTTTGTCTCTCCTGAAGTCATCGGGTTATCCATTCTGGGCGCTATCCAGCTCAATGTTGTGTTAGCTATGAATCACAAACCAACCCGTTATTCCGTCAATTACGGTTGAGAACCACTAGATAAAAAAAGCCCGCGAATTGCGGGCTTTTTTGCGTTATTAGTTGCTGCGATTAGTGTGAGGTTCTGGCCTTCACATCGTGACTTAGCTCTTTGTTCAGTTCAGCTTCCACATGTCCCGGTGATTGCGTACTTGCTGAAATCAATCGGTACATCGCAGGAATAACAAACAAAGTCACCAGCGTTGCGAAGCCCATACCGAAGAAGATCCCCGTACCTACCGCCACTCGGCTTTCATAACCTGCCCCTGTCGATAAGAGCAACGGAATTGCACCAGCAAGCGTAGTAAATGCCGTCATCATGATTGGGCGCAGACGACGTGCCGCAGCATCGACTATTGCTTTCTCAAATTCGAAGCCGCGATCACGAAGCTGGTTCGCAAACTCAACGATCAGGATACCGTTCTTGGTCACCATACCAATCAACATGATCATGCCAATCTGGCTGTAGATATTCAGCCCCTGACTCATAAAGAACAAGCCCAAGAAGCCGCCAAATACCCCCATAGGGACAGTGAACATCACCACCAGTGGGTTAATGAAACTTTCAAACTGCGCTGCCAAGACTAGGTACGCGACCAGCAGTGCTAAGCCAAACACCACCAGAATACTGGATTGGTTCTCTTTATAATCTTTCGATTCACCAGAGTAGCTGACTGAGATATCGCCCGGCAGAATTTCCACCGCTTGTTGATCTAGGAAGTCCAGTGCCTGACCTAAGGTATACCCGTCAGACAAGTTAGCCGTTACGGTAATCGATTTCTGTTTGTTGTAGTGTGACAAACGAATCGACGAAGCCACTTCTTCAATCTTGGTGACCACATCCAGTGTCACCAACTCGCCAGAGCTGGTTCGCAGATAAATCTGGCTTAGATCCGTCGCGTTATTGAAGCTGTTCTCATCACCGCGTAAATAAACATCATATTCTTCACCACGCTCGACAAAAGTCGTTTCACTCTTACCGCCAAGCATAATTTCTAAGGTCTCAGAAATCTCTGATACGCTGATACCCAACTCTGCCGCACGTTGTTTATCAACCGTCACCACCAGCTCAGGGGTTCGCTCAGAATAGTTAATGTCCGCCCCTTCCATGATCGGAGACTCTTCCGCTAACTGCTCAAGCTTTTCGGCCCACTGTTTCAGCTCAGGGTAATCAGAACCACCTAACACGAACTGGACGGGCTCACTAGAACCACCTCTGAAGCCTGGCATAAATGGGAAGACACGAACATCAGGAATACCAACCAGCGCCTTTCTTACTTCTCCTAGCGCCTGCTGAGCCGTTACATCACGATCATTCCAGTCATCCAGAATCATGATGACAAAACCCGTCTGGTCACCGGCGTTACCGCCAAAGGCTGGCGATTGAATACTGAAGGACTTAAGGAAACCTTCGCCCAGTAGCGGCAACAAGCGCTCCTCGACGATATCCATGTTCGCAGCCATGCGGTTGTAACTGGTAGCATCAGCGCCACGAACAAAGGCAAAGATCACCCCGCGGTCTTCAGATGGCGTGAGC

>NODE_105_length_2797_cov_2.166544

ACGTAGATCTGGCGGAAGTACCGGAAGCACAGTTAGGATCATCCACTCTGGGTTGTTACCAGATTGAACGAACGCTTCAACTAGCTTCAAACGCTTAGTGATCTTCTTACGCTTAGTCTCTGAGTTAGTAGACTGAAGCTCTTCACGCATTTCTTCGATTTCAGCAGGCAGATCCATAGACGCAAGTAGGTCTTTGATCGCTTCTGCACCCATCTTAGCCGTGAATTCATCACCCCACTCTTCTAGACGATCCAGATACTCTTCTTCAGTCAGCATCTGAGATTTTTCTAGGTCAGTCATACCCGGTTCAGTTACTACGTACATTTCGAAGTAAAGAACGCGTTCGATATCACGTAGAGGGATATCCATTAGTAGACCGATACGAGATGGCAGTGATTTTAGGAACCAGATGTGAGCAACTGGTGAAGCAAGCTCGATGTGGCCCATACGGTCACGACGAACTTTAGTTTGTGTAACTTCAACGCCACACTTCTCACAGATAACACCACGGTGTTTCAGACGCTTATATTTGCCACAAAGACATTCGTAGTCTTTTACTGGACCAAAGATACGCGCACAGAACAGACCATCACGCTCAGGTTTGAACGTACGATAGTTGATCGTTTCAGGTTTTTTAACTTCACCGAAAGACCATGAACGGATCATGTCTGGTGAAGATAGACCGATTTTGATTGCATCAAACTCTTCGGTCTTATGCTGTGCTTTTAGAAAGTTTAATAAGTCTTTCACAATCAGCTCCTGTAAGGAGTTAAAAGGAGCTCACCCGCAAAAGCGAGCACCTTCAACCAAATAACCCCGTGGGGATTACTCTTCGTCTTCTAGCTCGATGTTGATACCTAGCGAGCGAATCTCTTTCAACAGTACGTTGAACGATTCTGGCATGCCAGGTTCCATGCTGTGGTTACCGTCTACGATGTTCTTATACATCTTAGTACGGCCGTTAACGTCATCCGACTTAACGGTTAGCATTTCTTGTAGGGTGTAAGCCGCACCGTATGCTTCCAGTGCCCATACTTCCATCTCACCGAAACGCTGACCACCGAACTGAGCTTTACCACCAAGTGGTTGCTGAGTTACTAGGCTGTACGAGCCAGTAGAACGAGCGTGCATCTTGTCATCAACAAGGTGGTTCAGTTTCAGCATGTACATGTAACCAACCGTTACAGGACGCTCAAACGCATCACCTGTGCGACCATCAAACAGCGTTAGCTGACCAGATTCTGGTAGGTCACCTAGTTTCAATAGCTCTTTGATTGATTGCTCAGACGCACCGTCAAATACTGGAGTCGCGATCGGTAGACCACCACGTAGGTTCTTGATCAGCGTACGAACTTCATCATCAGATAGAGACGCAACGTCTACTTTCTGACGAGTATCGCCAAGGTCGTAAACTTTTTGTAGGAAGTCACGGAACTTAGCCAGCTCTTGCTGCTCTTTCACCATCTGGTTGATCTTGTCACCGATGCCTTTCGCTGCCAGACCTAAGTGAACTTCTAAGATCTGACCGATGTTCATACGCGAAGGTACACCCAGTGGGTTCAGTACGATGTCAACCGGCTGACCTTTCTCATCGTAAGGCATGTCTTCAACAGGGTTGATCTTAGAGATTACACCCTTGTTACCGTGACGACCGGCCATCTTATCACCAGGCTGGATACGACGTTTAACCGCTAGGTAAACCTTAACGATCTTCAGTACACCAGGTGCTAGATCATCACCTTGTGTGATCTTACGACGCTTAGTTTCAAACTTCTTATCGAAGTCAGCACGTAGCTCGTCGTACTGCTCTGCTAGCTGCTCAAGCTGAGTCTGTTGAGCATCATCTTCAAGCGTTAGCTCTAGCCACTTCTTACGATCAATAGCGTCTAGTTTCGCTTCAGTGTAACCACCGTCAAGAAGTACAGCTTTAACACGGTTTAGAAGGCCACCCTCAAGAATCTGGAATTCTTCAGTAAGGTCTTTCTTCGCTTCTTTAAGCTGCATCTGTTCAATTTCAAGTGCACGTTTGTCTTTTTCAACACCGTCACGAGTAAATACTTGAACGTCGATGATCGTACCTGATACAGAGTTTGGTACGCGTAGAGACGTGTCTTTAACGTCTGACGCTTTCTCACCGAAGATAGCACGTAGTAGCTTCTCTTCAGGCGTTAGCTGAGTTTCACCTTTAGGCGTTACTTTACCTACAAGGATGTCACCGCCTTTCACTTCCGCACCGATGTAAACGATACCTGACTCGTCTAGTTTAGACAGAGCAGACTCACCTACGTTTGGAATGTCAGCTGTGATTTCTTCAGCACCAAGCTTAGTATCACGCGCCACACAAGATAGCTCTTGAATGTGGATCGTAGTGAAACGGTCTTCTTGAACTACGCGCTCAGATACTAAGATCGAGTCTTCGAAGTTGTAGCCGTTCCAAGGCATGAATGCGATACGCATGTTCTGACCAAGTGCTAGCTCACCAAGGTCTGTTGAAGGACCATCAGCAAGAACGTCACCACGTGCAACTGGCTCACCCGGCATCACACATGGACGCTGGTTGATACACGTATTTTGGTTAGAGCGAGTGTATTTAGTTAGGTTGTAGATATCGATACCTGCTTCACCAGGGATCAGCTCGTCTTCGTTTACTTTAACAACGATACGAGAAGCATCTACAGACTGGATTACACCACCACGTTTCGCTACTGCTGTAACACCAGAGTCAACTGCGATGTTACGCTCGATACCAGTACCAACTAGAGGCTTGTCAGCCTTAAGTGTTGGAACGGCCTGACGT

>NODE_106_length_2796_cov_2.244575

AAGGTGAGATTATTACCATGTCGGAGATTTTTAAATTTCACCGTAAAGGCTTAGATGAAGATGGCAATGTGCTAGGGTACTTCACGGCGACTGGTGTGGTACCTCAGTGCCATGATCAGTTGTCGAAAAAAGGGCTCCATCTGTCTTTCGATATCTTCAATGAGTCATACTCATAAGGAGAAGAATTATGCATCAGGACGCAACATTCTTTTTCATACTCTTGTTTTTTGCTGTGGTGTTTATTTCACAAGCGTTGATTTTGCCTGCAGCGGGGAGCAAAGCTAAGCACAAAGAGTTGTCCGAACGTCTTAAAGAATCCCAATCGAAGCTCGATGAAGAAGCCTTGTCTTTGCTCCAAGAGCACTATCTTAAAAGTTTAACGCCGTTAGACAGAAAGCTGGTTCGGTTTGAGGTGTTCTCTTCACTCAAAAAAATGATTGAGCTTTCCGGTTATGATTGGAGTTTGACACAGACTTTGACAGTGACATCGATTCTATCAGTGTGTGGCTTCTTTATTGTTCTATTGATAAGCCAACCTGTCTATGTGGCTCTAGCAGTAGTAGTTGCCATCTGGGCATTTCTTTATTTCTGGCTCAGCAAGAAAATATCGGACCGCCTTTCCGCGTTTGAAGGACAGCTACCGGAGGCATTAGATATTATGCGCCGTATGCTGCAAGCGGGACAGCCCGTTACTCAAGCCTTCAGTGAGGTTGGGGAAGAGATGCCAGCTCCAATTGGTGTTGAGTTCAAAAATACCTTTAATCTTTTGAATTATGGCTATGACATGCGTATGGCGATCATGCAGATGGCAGAACGAACGCCGACCGTTTCCATGCTGGCGTTTTCCAGTGCAGTGCTGCTGCAAAAGGAGACCGGGGGTAATTTATCTGAGAATCTGGAAAAAGTGTCTAGGGTATTGAGAGCTCGTTTTAAGTTGCAGCGAAAAATTAAGACATTATCAGCAGAAAGCCGTTTATCTGCATGGATTTTGGTGTTGTCACCATTTGTGCTGTTTCTTTTCCTCAGTTTTGTTAACCCTGGTTATGTTGAACCCCTGTATACCGACCCCAGAGGCATGAAGCTGATCAGTGGTGGTGTCGTTAGCTTATTTATCGGTTCTCTGTGGATACGGAAAATCATTAATTTCGAGGTGTAGACATGGAAACGATTCTGGAACAGTTTGCAGTTTACAACTTAGACGAGCAGACAGTCTTTTTGAGCCTAATCCTCTTGGCGACGGTGTTGGTGGTGTTCACCCTGTCGCTGTTGCTGGTCGGGCCTAAATCGCCTCTAAAATTAAAGCTTGAACAACTCAGGCAGTCTACTCGTTCAGACAAACTGAAAAAAAGTCGCAGGCTGGATCATACCTTAGAGTCACTGGCTCCTGTGATGAAGCCTTCTAACTCAAAAGAGAGCGAGAGTATACGGCAAAAACTCATGCATGCTGGCTATCATGATGCGAATGCATTGACGGTTTTCTATGCGTTTAAAGGCATGACAGTCGCGGTCGGTTTAGGGTTAGCGGCAGGCTTTTACTTTCTTACCCCAGACATGGCGATGCTGAATTTAGTCATGGTAGCGAGTGTTGGGGGCGGGCTTTATGTGCCCAATATACTTCTCGGACATTTGGTCAAAAAGCGTCAATCAAGAATTCGTGGTGGTATCCCGGATGCATTAGATTTAATGGTGGTGTGTACGGAGTCGGGTTTGGGATTTAATTCAGCACTTCGCCGCGTCGCTGACGAGGTTGCAATCTCTCATCCAGATTTTGCTGATGAGCTGGATACCGTATGTGTCAAAATTAATGCAGGTGTGGAAATGTCAGATGCATTTAATGATTTGATTGAGCGTACTGGGCTTATTGAGATTGCGGGTCTGGTCACCATGCTGGCACATGCCTCGCGCATTGGTGGTAGTTTGGCACAAACATTACGTGAATATACCGATGATTATCGGGACAAGAGAAATCAAGAAGTCGAAGAAATAGCAGCGAAGATACCGACTAAAATGATATTTCCTCTGCTGCTCTTTATCTGGCCGTGCTTTTTCATTGTAGCGATCGGCCCTTCTATGCTGCTATTCACATCGACCCTAGGAAACTAAAAGGAGTGGAGCAATGAAAGGATTTCATCTGATAGTGCTCACCCTATCTGGAGCGCTGCTAACGGCTTGCGCATCGAATGAGCCGAGTATTGATACGTCGCTTTATGAAGGTAAACCCATTGACTCTTTGACTTCAGACGAGCCACCGAAAACAGAAAAAGAAGCCATTACTCGGGGCGATGATGCTCTGAATGCTGGCAATGTTGATTTGGCTTTGTATGAATACATTCGCTCGTTGGCATTTGAAAATGGCCAATTTCACGACCGTTCGCTGTACAACATTGGTCGAATTCACCAATCTCGCAACAATATACCGATGGCGCAAAAAGCGTATCAGCTGGCGCTGAAAGAAAATCCGAATAATGTGAAGGTGCTTGAACAGCTCGGTACCATTTACAGCCGCTCGGGCGATGTGGAATCTGGTGGTAGTTATTTCCTCAAAGCGATTAATGCCGACCAAATTCGATTGGGGAGCAAAAAAACACTCAGCGAGGAAGATTTAGCGACTTCGAAAGAGATAGCTATGTTGAAAACAGATAACTATTCTCCAGAGTTTGCCTACATGGGGCTAGGAGTCATTGCCGATATTGATACTGATCATAAGCGAGCTCAGGCGTTCTATAAAAAAGCGCTAAGTATCAAACCAAATTCGGTAAAAACCTTAACCAACGTAGGTTACTCATTTTATATGGA

>NODE_107_length_2775_cov_3.138992

ACGTAGCTCTTCACCAATGTACTCTACTGGGTGGTTACGGATAGTTTCGTTCACAGCGATTAGCGTCGCGTTGTCAACTTGGTTAGATTCTTCACCTAGGCCTTTACCGATAACGTCAGTACCAATTGAAGGCATGAACTTCTCACGCAGTAGCGGAGTTGCTACGTTAGCGAATAGGTAGTTACCGTATTCAGCTGTATCAGAGATAACAACGTTCATTTCGTACAGACGCTTACGTGCTACTGTGTTTGCGATTAGTGGTAGCTCGTGTAGTGACTCGTAGTACGCAGACTCATCAATGATGCCTGATGCCGTCATTGCTTCAAATGCTAGCTCAACACCTGCGCGAACCATTGCAACCATTAGGATACCGTTGTCGAAGTACTCTTGCTCTGAGATCTCAACATCACCTGCTGGGTAGTTTTCAAATGCTGTTTCACCTGTCTCTGCACGCCAGCCTAGTAGGTTCGCATCGTCGTTTGCCCAGTCAGCCATCATGGTGCTAGAGAAGTGGCCTGTGATTACGTCATCCATGTGCTTGTTGTACAGTGGACGCATCAGCTCTTTCAGCTCTTCAGAAAGCTCGAACGCTTTGATTTTCGCCGGGTTTGATAGGCGATCCATCATGTGTGTGATGCCACCAAACTTCAGTGCTTCCGTGATGGTTTCCCAACCGTACTGAATCAGTTTACCTGCGTAGCTTGGGTCAATACCTTCGGCAACCATCTTCTCGTAAGAAACGATTGAACCTGCTTGTAGCATGCCACATAGGATAGTTTGCTCACCCACTAGGTCAGATTTCACTTCAGCAACGAATGAAGACTCTAGACAACCCGCGCGGTGACCACCAGTACCAGCAGCCCAAGCTTTCGCGATATCCCAACCTTCACCTTTTGGATCGTTCTCTGGGTGAACCGCGATTAGTGTAGGAACACCGAAGCCACGCTTGTACTCTTCACGTACTTCTGTACCTGGACACTTAGGTGCAACCATCACGACTGTTAGGTCTTTACGAATCTGCATACCTTCTTCAACAATGTTGAAGCCGTGTGAGTAACCTAGTGCTGCGCCTTCTTTCATTAGAGGCATTACCGTCTCAACCACGTTCGTGTGCTGCTTGTCTGGCGTTAGGTTAACCACTAGGTCTGCTTGAGGAATAAGCGTCTCGTAGCTACCTACTTCAAAACCATTTTCTTTTGCGTTTTTGAACGACTGACGCTGCTCATCAATCGCTGCCTGACGCAGTGCGTACGCCACATCAAGACCTGAGTCACGCATGTTCAGACCTTGGTTAAGACCTTGTGCACCACAACCAACGATAACCACTTTCTTACCTTTCAGGTAATCCGCTTCTGTAGCGAATTCACTGCGGTCCATAAAGCGACAACGGCCAAGCTGATCTAGCTGCTCACGTAGGTTTAAAGTATTGAAATAGTTAGCCATGTTAGGGCTCTCCTTTGAGAATTTATCCGTTTGGGTCGGTGTTTCATTACCGAATGCCTCGATACTAAATCAGACATCGGGTTGCTTAAAGTGATATATTCACAATTAGTTATTGCAAAAAATGCAACAAGGAAGCACTTATTTCATGAACATTAAAAGTCTGCAATTGTTCATTCATCTGTGTGATAGCAAAAGCTTCGCCAAAACAGCTTCTGCCATGCACATCAGCCCTTCTGCCCTCAGCCGTCAGGTACAAAAGTTGGAAGCAGAAACCGGTCAAGAGTTGTTCTTACGGGATAACCGCAGCGTTGAGCTGACCCCAGCGGCAAAAAAACTTTTGCCAGTGGCTTTAAAGATTCTTGGAGAATGGCAGAACTTCAATGCGCAGCTTCAAGGGCACGAAGAAGAGTTAAAAGGTGAGATTCGCCTGTTTTGCTCGGTGACTGCCAGCTACAGCCACCTGCCTGAGCTGTTGTCAGAGTTTCGCCTTCAGCATCCGTTTATCGAGTTTAAGCTGTCTACCGGCGATCCGGCTCAGGCAATAAACAAAGTCCTTAATGATGAAGTGGATGTCGCAATCTCCGCTCAACCAGAAGCACTACCCGCCAAGCTCAAATTTGAAACCATTAGTGAAATTCCTCTTTCAGTAATCGCCCCTGTCGGCGTGAGCAACTTTGCAGAAGCACTGCAAAAGGACAAACCTGACTGGTCGTCCATTCCTTTCATCGTGCCCGAATCAGGTACCGCACGAGACCGCGCCAATACTTGGTTTAAGGCGATGAGAATCAAACCAAACATCTATGCGCAAGTTTCAGGGCATGAGGCTATTGTCAGCATGGTCGCGCTCGGCTGTGGTGTTGGGATTGCACCAGATGTGGTGATCAACAACAGCCCTGTCAAAGAGAAGATTCAACGCCTTAAAGTAGCGTCGATTAAACCCTTTAAGCTCGGAGTATGCTGCAAGCGTTCGCAACTGGATAATCCGCTGGTTAAAGCTTTATGGGAAGTCGCACAGGACGCTTACATCGCGCCTTAACCACTTACCAGCTAAAACAATAAAGCCACCGTTTTCGGTGGCTTTTAAATTCATACTTATCGAAACAATGTCCGCAATTAAATCACTCGCGAGAATTGCTGCTGACGCGCCTTTGCTCGGAGATATTTATCAAAACACATACAGATATTGCGAATCAGTAGACGACCGCGTAATGTCACTCGGATCTCTTTGTTGTCGACTTCCACTAGCTCATCATCAATAAAGGTTTGCAGCAGCTCGAGATCTTCTTTGAAGTAGTGATTAAACTCGACATCAAACTCCGTCTCAACTGATAC

>NODE_108_length_2774_cov_2.176492

GGATACCTCTCAGGGGGATTGGAAGTGCAGCTACCGCTTGTTGCAGTCAACAGATGTTATGCCTTTGGTGTTCGATTATGCGTGGCTAATGAATGGTCAGTTTGCGTTTTATCTTGTTGATTCAGCAAGTGAAGGCCAGCATGGTGCGGCAACTACTTTACTAGTCCGCGCTCTATTCCATGACTATTTACGTAACCTTAAATGTTTTAACGCGGATCTCAAAGACATTGCAGATTTGCTGGAGAAAGGTTTAGCCTGTTCAGACTGTGCTAGCCCAGTACATGCCATGTTTGGTATTGCTGATGTCTCTGAAGGTACTTTGTCTATTTTGCCGGCAGGGCTGGATGCCCAATGGTCTAATGGCTATTTTTCTCAGCACATCCCGGCGGGTGTGAGGCTAGGGGATAGTTGCGCGAAGAACTTTATTACCACCGATTTGCCAATACAGTCTGCTTGCCAGCTAACTTTGAGCTGTCTTGGGTCAAGTAGTTTCAGCTTGGACATTTTCCGCGGAGGCAGTGCATAAGCGCTGCCTCGTTATTGAGCTCAATATCGAAATAACCCTTCTTTTGCTCGGTGTTTATGAGCAAGAGATTAAGGTATAGTCCGCCAAAACAAAAATGACCCAATGTTTTAACGAAAGTTTAACTCGTGGTTTGAAAAATCGTCACTTACATTAGAGAAGTAATCCCATGGCAGACAAAGATTTCAAAGAACCTTATAATATTTTCTACTTTCTAGGTTTTATTGCAGCTCTATTGATTCCAACACTACCAGCCACGTTAACTTGGATTCGTGTAGTAAACGGATACGCGGGCTTCTAACTGATTTCTCGGAGCTTACCATCAGTATTCAAAGATTTGGCTTAAATATTGTTGGTGGCTTAAGTTTGTGCCTAGGCTTTCTGGGCATATTCCTCCCTCTTCTTCCAACGACTCCTTTTATTCTTCTTGCTAGCGCTTGTTTTATGCGCAGTAGTCCAACTTTTCATCGTTGGTTGCATGAACACAAAACGTTCGGACCTATTCTAGACAATTGGCATCAGCATGGTGCTGTAACCAGCAAAGTAAAAACACGTGGTGCTATCTGTATGGTCGCGAGCTTTACTTTTTCTATTTGGATTGTTCCTCACTTTTGGTTAAAGATTATGCTGGTGGTCATGCTGATCATCTTATTGACTTGGTTTATTCGTTTGCCTGTGATTGAGCGCCTTGCTGACAAGCAAGAAAATCACTAAGATTGCAGGGAAGTGTGCCCGCTCTTCGAGTGGGCGTTTGTTATTGTAGGCACAGATAATTTAAGTCAGCTGTTAATGACCCAATTGTGCTGAGAACCCCGCTAGCGCTTGCCACATAATGACACAATAAAGTACAAGCTTAGCCAATCTAAGAAATGAGTTATGACAACTGAAACAATCTCACTGATCAAATCTAGCATCAAAAGTATTCCAGATTACCCAAAGCCAGGCATTCTATTTCGTGACGTGACTAGCTTGATGGAAGACGCTCCTGCTTACCAAGCGACGATCCAACTATTAGTTGAAAAGTACAAGGACATGGGCTTTACCAAGATTGTTGGTACAGAAGCGCGCGGTTTTCTTTTTGGCGCTCCTCTTGCACTTGAGCTAGGGCTAGGCTTTGTGCCTGTTCGTAAGCCGGGTAAATTGCCACGTGAGACGGTTGCACAATCTTATGAGCTTGAGTACGGCACTGACACACTGGAAATTCACACTGACGCGATTGCAGAAGGTGATAAAGTGTTAGTTGTTGATGATCTGCTTGCGACTGGTGGAACGATTGAAGCTACCACTAAACTGATTCGTCAGTTGGGTGGCGTTGTAGAACATGCTGCATTCGTTATCAATCTTCCAGAGATTGGTGGCGACAAGCGTCTAGAAGGTTTAGGCCTCGACGTATACAGTATTTGTGAATTTGACGGCCATTAATCGGATATATTCATGAGTTATCTTGCTTTAGCTCGAAAGTGGCGACCTACAAAGTTTAATGAAGTTGTAGGGCAACGCCATGTTTTGACAGCATTAGAAAATGCTTTAGCCCAGAACAGGCTTCATCATGCCTACTTGTTTAGCGGGACACGTGGTGTCGGTAAAACGACCATAGGTCGATTGTTTGCCAAGGGGCTGAACTGTGAAACAGGGATTACCGCTACGCCATGTGGCATCTGTGATACTTGTAGAGAAATCGATGAAGGTCGATTTGTCGATCTATTAGAAATCGACGCAGCGTCACGCACTAAAGTTGAAGATACTCGTGAATTGCTTGATAACGTGCAATACAAACCTGCACGTGGTCGATTTAAGGTCTATCTAATCGATGAAGTTCACATGCTCTCGCGCCATAGTTTTAATGCGTTGCTTAAAACACTTGAAGAGCCGCCTGAGTATGTGAAATTCCTTCTGGCGACAACCGATCCTCAGAAACTGCCAGTTACCATTTTATCTCGCTGTTTGCAGTTCCATCTTAAGCCAATCAGTGTTGATAATATCCATGAGCAATTGGATCATATTTTAAAGCATGAGCAAGTTTCTTCTGAGCCCCGAGCGCTTGGAATGATTGCGCATGCCGCAGATGGCAGTATGCGTGATGCACTAAGCTTAAGTGATCAGGCTATTGCATTGGGGAATGGACAGGTGCTTGCCGACAGTGTGGCGCATATGTTGGGTACACTGGATACCGATCAGGCGTTGCATTTGCTTGAGGCGATCAGCTCAAAGCAACCTCAGGTTGCGATGGACAGTTTATCGGCTTTAGCGC

>NODE_109_length_2769_cov_2.352155

CGCGTATGTTGCTCCGGGAGATAAACATTTAGTCGTCATTAAGCGTGGCCAACATTTGTATTGCCAACTGGATGATCGTCCAACAGTGAACCGACACAAACCATCGGTCGATGTGATGTTTGATACTGTTGCAGAAACGGTCGGAAAGAATGCCATCGGCGTCATCTTGACTGGTATGGGGCAAGATGGAGCGCGAGGAATGCTTAAGATGAGAGAAGAAGGGGCAATGACGATCGCTCAGGACAAAGCTTCTTCCGTTGTTTGGGGAATGCCGCGAGTTGCGGTTGAAATGAACGCTGCGCAACAAGTGCTGAGTCTTAAACAGATACCTCAGCAGATATGTGATTGTCTAAAGAAATAAGCGCCTGTGCGGGCACAACGTCAGGGATGTACTATGAATAAAATAACAATGACCCCAAAAATCCGCTACGCCCTTGCTATAGGGACGCAGATTGTATTGCTTGCTATCGGGATGACGTTGTCATCGTCGATTGCGCATGTCGCTGTTATGGTGCTTGCGGCGGCTATACCATGGTTTGTTTTCCCTAGAGAGGCTTCTACGCCAAATAAGTCTGAGTTAACTCAGTTCCGTGAAAAGTCATCTCAGGACGAAAAACAGATCAGCCTACATGTCAGTGAAATATTGAAAAAAATATCCCTACAATTAAATGAGCCTTTAGATCATCAACATGGTGTTGTTGATGAATCTGCAGAGACGCTAAATGAAAGCTTCTTTGAGTTGCAGAAGTTAGCAGAGGGACAAAATGCAATTGCTGAAGAGCTAGTGAGTAACATTATGGGTAATAAAGATAATGCCTACGATATTACTCAGGTCCTGCCCAAAACAGAGACCATCATTCGCCAGTTCGTTGACACACTCATTAATATCTCTGAGAAGAGCATTTCCGCAGTACATAGTATTCATGACATGTCCGATAAGTTGGATGCGGTGTTTAAATTGTTAGCGCAAGTTCGTGGCCTATCCGAACAAACCAACCTGCTCGCTTTGAATGCTGCGATTGAAGCAGCACGTGCTGGGGAGGCTGGGAGAGGTTTTGCTGTTGTGGCGCAAGAAGTAAGAAACCTTTCTGTAAAAGCAGAAGAGCTTAACTGCGAAATAGAGAAGGAAATTAATGTCGCTCAGGATACGGTCAAAGAAGCGAATAAAACAGTTGGGGAGATGGCATCAATCGATATGACGGAAGCGATAGAGTCTAAGGAAAAAGTGGATGATATGCTTCAAGGTGTTAAAGCGGCTAATGCTGTCGTTGAACAGGAAGTTCAGAAAATCAAATCCATTGGGGTCACGCTGCATACACGTGTTGGTGACGGTATTCGGGCGTTACAGTTTACAGACATCATTGTCCAACAGGGTGATTACGCCAAGATCAGTGTTGATTTTCTCGATCAGGTAACTGAAATACTGGTGGCTCATGCCAGCGGCAGCCTAGACCCTGATGAGATGAAACAGGCTGTGGCAGATCTGAAATCAAATATAGACCATCGCGGGGCTCCAGCTGCTTCTCAGGAAAGTATTGAAGAAGGAGAGGTAGAGCTATTTTAATGGGTCATGCGTATACATATTGTCGTGAAGGCGGTGCAGTCTTCGTTGTTGAAAAGGAAATATTATGTCGGTAGAAGCTGAAGTTGATTCAAATTCCAAGCAAGTAACGATCTTAATTGAAGGGGCTTTTGGATTTAATCTAGTCCAAGAGTTCCGTCGTTGTTATTCAGATAAGAAGGATTTTCGTTTTACCATCGATTTACGGAAAGTCGATTATATAGATAGTGCAGGGCTAGGTATGCTGCTTAATATGCACAACTATCTTGAGCAGGATGACGGGATGATCCGGATTACCAATACCTTGCCACAGGTGCGAAAGATACTGACCATTTCTCGTTTTGATAAGAAGTTTTCTATCGAATAGCGGTTAAGGAGGGTCGTATGCATGTCATGATAATTGACGACCACGCTACGAATCGAGAATTGTGTCGATTCATGCTCAGCGAAATGGCGCAAGAAGTCACGACGTTCGAGAATGGTGAAGGGGTCGTGGAGGCAATGCTCAAGATGCCGTCTCTGCCAGATGTCATCTTGTTGGATGTAATGATGCCAGTGAAGGATGGCTTTACGACAGCACAGGAAATTCGTGATGCGTTCCCTAATCTTCATATGCCGATCATCTTTCTCACCGTACTGGATGATCACGATTCGTTCGAGCGATGCCTGACGTTAGGTGAAGACTTTATTCCTAAGCCCGTTGAGAGAAGTGTGCTAGTGGCGAAAGTTCAAGCTCACTATCGAGTTGTAAAGATGCACAATGAGGTGAAGGAACATCGTGATGAGCTAAGTAAGTTTCACGAGCAAGTTCGCTACGACTATGCGATCGCGGAGTCGATTTTCTCCAATTTAATGGATGAAATGAGTTCTCAAGTGAAAAGCATTTATGGCATCAACTACATTTCTACCCCATCAACAGTTTTCAACGGAGACTTGATCGTTGTGGCAAACCGGCCGCATGGGGGAGTATACGTGATGATCGCGGATGCTACGGGCCACGGCCTTCCGGCTGCTATTTCGGCTATTCCTGCAACCCGAGCGTTTTTCTCTATGGCTTCTAAAGGTCTTTCTTTGGGAGAGATTGTTCGCGAGCTCAATGACGTATTGGTACGTTTCTTACCCATGGGGATGATGTTGGCGGCAAGCGTGTTTGAAATTCGTGCTAACGGGTTTGAAGTTTCTTGGTGGGGCGGTGGACTACCTGATGG

>NODE_110_length_2760_cov_2.306373

GGTAAGCCATCTTCATCGGCAATCGCTTTGCGTAACTTACGCAACTTGGCAAACAGCTTTTTGTCGTAGTTTTTACTGGTGAGTTTGTCGGACTTAGCCGCTCTTGCGGCGGTATCCAGACGAGGCACGGCTAATTCAAGAGCGGTATCGCCACGCAATAGTGGGCGCGCTTCCTCAGTAAGCTGTAAGGTCGAGTTGCGAGTAATGTTCTGGAACAACATCCCTTTATGGATCAACTGACGAAAAATACTCACCCAGTAATCGTGACTGTTTTCACGCCCAATACCATAGGTAGTGAGCTTGTCGTGGCCATTTTCACGGATGCGGATATTCTGCATTCCGCGCAGGACTTCAACCACATACCCCATACCAAAGCTCTGATTAACTCGGTAAACACAAGACAACGCTTTACGTGCCTGCTCGGTGGCATCAAAATGCTTGGGCGGGTCCAAACAGATATCGCAGTTGCCACAAGGCTGCTCACGGTACTCACCAAAATAATTCAACAGCACCTGACGACGACACGTCTGCGCTTCAGCAAAAGCACTCATCGCATTGAGCTTATGCGCTTCAACCTGCTTTTGTGGCCCATCGTCTTTTTCATCCAACATCCGCCTTAGCCAGCTGATGTCCGCTGGGTCATACAGCATCATCGCTTCCGCAGGCAAGCCGTCCCGACCTGCGCGACCAGTTTCCTGATAGTAGGATTCAATGTTGCGCGGAATATCAAAGTGAACCACAAAGCGCACGTTAGGTTTGTTGATCCCCATACCAAACGCCACCGTCGCGACCACAATTTGGATATCATCACGCTGAAATGCTTCTTGCACATAAGCGCGCTCGTCTGCCTCTAAGCCAGCATGGTAACTGGCGGCACGAATATGGTTGTTGCACAGTTTTTCCGTCACCATTTCAACTTTCTTACGGCTACCGCAATAGATGATGCCGCAGTTGCCTTTCTGCGTGTCGAGAAAACGGATCACCTGAGAAATTGGTTTGTGTTTCTCAACCAAGGTGTAACGGATGTTGGGGCGATCGAAACTGCTTAGGTGGATATGCGGCTCGTTCAATTGCAGACGATGCATGATGTCATTGCGCGTCGCATCATCGGCCGTCGCCGTCAGCGCCATAAAAGGCACATGAGGAAAGTGCTGTTTGATCTGGCCGAGCGACGCATATTCAGGTCTGAAATCATGTCCCCACTGAGAAATACAGTGTGCTTCGTCAACGGCGATCATGGACAGCGATAGGCTTTCAAGACGCTCAATAAAGTCACGCATCAGCACACGCTCAGGTGACACATACACTAGCTTCAACTGGCCTGACTGCATTCGATTGTAGACCGAAATCAACTCATCGCGGGGCATGGAAGAGTTGACACACTCTGCCGAAACACCATTTGCTTTAAGCTGATCGACCTGATCTTTCATCAGTGATATCAGAGGAGAAATCACCAGTGTTAAACCGCCACGAACTAAGGCCGGAACCTGATAGCAGAGTGATTTACCGCCACCCGTCGGCATGATGACCAAACTATCCTGACCGTTTACGGCTGCTTCAATGACTTCTTGCTGACCATCTCGAAACGACTGATAACCAAACACATCCTCCAGTATGCTTTGTGGAGTCGGTGAAGGATCAGATTGTTCGGCAAGCAGTGTCGCGGTCATTGATATCTCGATTGAGCAAAGAGTGGGGAAATGCAAAGGGCACATTGTAGTAGTGTTCGGATGTGAATAAAACCGCAAATTGCTAGGTGATTAACTGACTCATTCGTATACTGGATGCTTCCTTTTGCAAAAATCATCCTTTGAGTAAACGTCAATGACACCAGAAGAACAGCAAAGAGCACGGCAAGGCGTCCTTCTCGCCATAGGTGCCTACACCATGTGGGGCATTGCACCGATCTATTTTAAAGCCATTAGTGAGGTTTCACCGTTTGAAATCCTCAGCCATCGAGTGGTGTGGTCTTTTGTTCTATTAGCGGTATTGCTACATGTAGGTCGTCGCTGGCGTGGTGTACGAGATGTGATTCGCTCCAAAACCAAAATGCTGTTTCTGGTTTCTACCTCACTACTCGTGGGAGTGAACTGGCTGATTTTTATCTGGGCGGTAAATGCCAATCACATGCTAGATGCCAGTCTCGGTTACTACATCAACCCTCTGATTAATGTGCTGCTCGGCATGTTGTTCTTAGGCGAGCGGCTACGTAAATTGCAGTGGTTTGCTGTGGCACTGGCAGCTTGTGGTGTGATGGTTCAGCTGATCGCTTTTGGCTCTGTGCCTACCGTCGCCATTGCTCTGGCACTGACATTTGGTTTTTACGGACTATTGCGTAAGAAAGTCAGCTTAGATGCTCAAACGGGCCTATTCATTGAAACACTGGTAATGCTGCCTGCCGCAGCGGTGTATTTATTGTGGATTGCCGATTCTCCGACTTCCGATTTTTCAGCGAACCCGATGAACCTAAACCTACTGCTGATCTCCGCTGGTGTTATCACCACTTTGCCGCTGTTGTGTTTCACTGGCGCTGCCACACGCCTTAAGCTCTCAACTCTGGGCTTTTTCCAGTACATTGGCCCAAGCCTGATGTTCTTGCTGGCGGTACTGGTGTACGGCGAGGCCTTTACCACAGACAAGGCCATCACCTTCGCCTTTATCTGGGGCGCGCTAGTGGTCTTTAGCTTCGACGGCCTTAAAACCAGCAAGCAATCACGCAAAGTTGCAACGGCAAAGTGATCGTTTTTTACAAGACAATGT

>NODE_111_length_2757_cov_1.813806

GTTGTCTGCTGCTACGGTGAATGTACCAAGATTGGCCGCACATCGATGAGTATTGATCTTGAAGTCTGGGTAAAACCTGTCAAAGAGCACGGGATAGAAGATCGCTTCATGGTATGTGATGCGACGTTCAACTATGTGGCCATCGACACTGAGGGGAAACCTCGTCCAATCAAACAAAACTAATCTCACACTTTGCTGTCAGAAAGTCTTTAAAAAGCTGTTACATTCTGTAGAGTTAGGCTAATCACACAATAAGCGGCTGCGCTGTCGCTTATTCATTATAAGGACATCAATACATGTGGTATGTAATTTTCTCTCAAGACGTTGAAAACTCACTGGAAAAACGTTTAAGTGCGCGCCCTGCTCATCTAGAGCGCCTTCAAACACTTCAAAACGAAGGCCGACTGCTTACCGCAGGCCCAATGCCAGCTATTGATTCGGACAATCCTGGCGAAGCGGGTTTTACAGGCTCTACCGTTATTGCTGAGTTTGATTCTCTTGAACAAGCTCAAAACTGGGCTGATGCGGATCCGTATATCGAAGCAGGTGTCTACGAGAAAGTGATCGTCAAACCTTTCAAGAAAGTATTTTAAGATGAGAATGAAGATTGCGTTAGGTGTGCTTTCTGCCGTTCTTTTGGCAGGATGTGCGTCACCGGAAGGAGACAAGCAAAGAGAACTGGAGCTGCTCGCAAGCAACCGAGCCAGTCTGCTGGCCTCAGAACTGCCAATGGAAGCTGGCCCGCTTTCTATTATGAGAGCCACTGCGAAAGGCACCATGATAGAAATTATGATGGTATACAATCAAGATGCTAGAGGTGCTAAACCCATCCAGCAAGTGCTTCGCCACAGCATCAACAGTTACTGTACGAATCACCAAAAACAATTTGGATGTCGGGTTGAGCTATCGATTGAAGATGCGTAACAGCCGCGGTCAATTAATGGTTGACGAAATAGTGACTCAACAAACCTGCCAAGCTAAACCGTAACCTTCCCTTCAGCAGGGTAACGACTCCTTAGACCTCGCAATGCGGGGTCTAAGTCATTGAAACATCTGCTCACGTAATCTAGCCCATCAACTTGATAACACTCTAGTTGCATAATGGGCATAGCACGAATAAATGAAGTTTTTATGACACCGATATTGAATCATAAGATGGATAAAAACCGCTAAGTGCCTATCGATTATTAAAATAATTCACCTAAAGCCGGCCATTTATCCAACAAATAAAAGATATATAATCTTTAAAACTGTGATCTTGCATTGACGCTTATCCAATCGCCTTCTAACTTTTCATATTAGTGTCATAACCAAGGAGGATCACCCAATGAAGCGCGCAAGCAATTCCTACCGTCTGCAATTAATAAAAGAAGTTGCCACCCGGCAAGAGCGGCTGAGCTGCGGTGATCCCATGGCCAACTATATCCAACAACTATTAGACTCCAAATCTGAGTCTGAGCTGCATGAAGAAAAAAATTATCGCTTTAGTGGCAACCACTTCGACGATCATGTCGGGGGGTGGGTAAGTGACAAGTGGGGATTAAAGTAACCCGTAAGGTTAGGACTATAAGCGAGAGGGAACATTTCTCGCTTTCCTGTTTATTGAGCATGTTTAAAGCAGTTAAAAGAGATCTTGCTGAGGGCTTTTCGGATCGGACTCCGCTTGCTGCTCTATTCTAGAGCCTAGCATTTTTCGACGATAACGCTGCCGACAAAGTTTAATAATGTGCAGTTGCTCGGCAGGGGTAAAAGTCAGCCAATTAAAGCGTTCTTCACGCTTACGCATACAGCCCTTACAGTAGCCTTTTTCATCTACTGTGCATACACCTACACATGGGCTAGGGACGACAAAAAATTCTAGCTGCTCCATCTGTGCCTCTTCATAGTTTTCTCAGCTACGCGTTAAGCACCCATCACTACTTTATTGCATCTATAATTAAATAAAATTCAATAACTTTCATTATGTGACTGTAAACAAACTATGACCTTAACCTGACAAAAACAGTGTTTTATAACCTATACTTTGTGCCATTAAAGTCGACAAAGTGTCGATTAACGTTTCTATATTTCGGAGTTCATTACTATGAAGTTTAGTCCAAAAACATTACTTGCAGCTGTCACCCTACCGTTTGTCATGACGGCTTGCGCGAGCAATGGAGGCGATGTGAAGCAAATAACAGTTAAAGATCTTCAGCATCATAACTGGGAACTGGTTAGTATTGATGGAAAAGAAGTAAAAGCGGAAGGTCACCAAAAACGACCACGCCTAGAATTGGGCGAGAACATGATGGCTAACGGTAACGCAGGTTGTAACAACTTCTTCGGTCAGGCTGAGCTCAAAGATAATCAGTTCCGTATTGAGAAAATGGGCATGACGATGAAGATGTGTATGGGTGACGCGATGGATTTAGAGCAGTCATTCTCGAAAACACTCGCTGAATGGAGCGACATGTCCCTGACCAATGAAGGTATGGTACTAAAAAATGACCTTCATACTCTGACTTTCAAGCTAAGTGATTGGAAAAACTAATCACCCACAACTAGAAACCAAAGCAGTCAATTATGACTGCTTTTTTGTTGCCCGTAGAAAAAGAGAACAAATCTCATACAACCCGCCTACAATTCCTTCAAATACATAAAAAAATTAAATTTTTTCAGTCATAATGAATGAAAAACATGGATAGCACGGTCTATCACAAAACTTATAGATTCATTCAAAGGGTAAGGACAACCATGAAAAGGCTGATCACTCTCATC

>NODE_112_length_2756_cov_2.346398

GCTTCATCTCGCTCAACCCTGAAATCTCCAACCCAGATTTAGCCGCCAAGCTTCTGACAAAACCGGAAATGGTTAACCTCTTTAGAGTAACCAACAAACAGGCGAAAAAGGACACAATTGTAAGTCAGCTAGAAGAGAGCCCATTCTCACAATACGGCACTTTGCCCTTCACCATCGTTAAGCTTGAATCACCGCAAGTGATCGATGTTTTGCTCACACTCTTCTTTGCTAATACTCATCAGGATTTAAGCCAGTTTGTATTAGACGATCTTGGCTTGCATCAATTTGAGAACTATCAACTCAGCGAACAATGCCGTTTCTTTTCTTCACGTGATGAGCTAGATCAACTGCTTTCATTATCGTTATTGCAGCAAAGTTATCATGACGCTGATCGAAAATGCGTAAATACAATACTCCTCCACCTTGAGCAACTTACACATCCTATTGACCACAACTACATAGAGCGTAAACGTCAACATCTGATCAATGACATGGCGCGCGACTTAGAAAGATTGCAAGAGTTCGAACTCTCGACTTTCTGGTTCGAACAAACAGTATTACCACCTTCACGAGAACGCCGAGCTCGCATGTATGACAAACAAAGCGATATAAATGCCATGAGTGACATTGTCACTTCAATATTAAGTTCCCCATATGATACGTCAGAACATGAAGTGGGTGTGAAATTGCAACAACGCTTGGATCGTCTCAAAGGTATAAAAGTCCCGAGAGTAAGGAAACCTAATATAGAGGAACACCACCTTACACTTGATTTGCAACAGCAACGCGTCGAGTTGGTGGTCAAAGATTATTTTGAGCAACAAGGTTATGACGTCTATTACTCAGAAAATATTCTGCTAAATGGATTGTTTGGCTTAGCATTTTGGGAAGTCATTTTCAGCCCTGTAGAAGGCGCTTTTATCAACCGATATCAATATCGCCCTTTGGATCTTTACCATCACGACTTTGCTTCCAAACGTCAGTCAATGATAGATGATGTTTTCACTCGACTAGAAGAAAACGGTAGCCATTATCTGAAGCAAATTTATCTGGAAAAAGTAGGGCTCAGTAATCCATTTGTTGCCTGGAATGCTTTCACTCCAGAACTTTTGGACAAGGCGAGCCACAGTATCTCTTCTGAATTACTGTGCCAGTTGTTTAAAGTCATGCTACAAGAGCTCAAATTATTCCGAAGTGGTATGCCTGATCTGGTTCTCTTTAAAGACGACCGTTTCGAATGGGTTGAAGTTAAAGGCCCCGGAGATCGTTTGCAGGATAACCAGTGGCGCTGGTTCCATGAGTTCATCAAACTGAAAGTGCCGTTTTCGGTTTGCTATGTAGCCAACTCATAACTAAATACGATTGATAATGCCCAAACCAATAAGCTCACCCATTTATTGGCATTGAAAGTTCAACCTCTTTTATTGATAATAACAATTATTATCAATAAAAGAGGTGTGTTCCATGTTTGAAGGTGGAGGAAGATTATCATTTAACGATCATAAATCTTATGGCCAAATACTGTCAGGCAATGAGCCATCAATACTATTGGTTGAAGACGATAGACATCTGAATCAGCAACTCACCTCTCTTCTTCGAGACAGTCGATACCAGGTTACCAATCTGGATTGTGGTGCCGAAGCATTGAATTGCCTAAAGCAATCCAACTTTGACCTAATCATTCTTGACATTAACCTGCCAGAGGTCGACGGCTTTGGTTTGTTGAACTTTATCCGCTTTCATTCAAATACACCGGTCACCATGCTGACCGCATACGGCGCAGAAGAACACCGCATACGTGGGCTGCGTTATGGCGCGGATGACTACATCAGCAAACCATGTAACTTTACTGAGGTGAGTTTACGGATCGAAGCGATTTTGCGCCGAACTGGTCACAATCAAGCATCGAGCTCTAACCGTTATCAAGAATATAAAGAACTCAAGCTTGATAAGAACGAACTGACTGTAACCGTCAACGAGTCCGCTCCCATCACCCTCACACCAATTCAGTTTAAATTGCTCTGGACACTTGTAGCCAACCACGGCGCAGTACAAAGCAAACCGTTTTTGTACCAGGTTGTACTAGAACGCGACTTTAGCTCATACGACAGAAGCTTAGACATGCATTTAAGTCGAGTGAGAAAAAGCTTAGTCGCTTTGGGTATGAGCTCAGAACGAATTCAGACCGTTCACGGCAAAGGTTATTTATTACGATGAAAAAGTTACTTTTCTGGCGACTGTTTGTCGTACTCTCATTGGGAGGAGTGATCTTTTTCTCCCTACTCCATAGCGCCGCTCTACTCTCCAATGAAAAGATGAGTTACATCGATAAAAACCATCAGCAAGAAATTCTCGCTTGGGGTGAGCACGTTCAGTCACTGCTCGGGCAAGGGAAATATGATGAAATGAATCAGTGGGTAAATCAGCTAGCGTTGCGAGAGGAAACGTGGGCAACGGTGGTACGTTCCAATGTCTCCGTTGTCAGCGGTAACCCTCTCAATAAGCGATTTTGGGATGGTTATGGCATAGGTAGAAACGTCGAGTGGAAGATTCACCTTTATTTTCCTGAAAACCCGATAATGGAAGTCGCTCTTACTCCTTCTGATTGTCACTTCTTGGTTAAATTGCCAGCTCGTATGCGCCCCGGAACCTACATGTCGCACGCATTCTGGACTTTCCGAGTCGTCATTCCGTTCTTAGCTCTATTGGCGCTAACTATCTACCTTTATCGCTACGTCATGACACCATTACAGCAC

>NODE_113_length_2749_cov_2.187874

GCGCAAGCCAGTGAACTCGCGGGTTTGGATGGCCATGATCACTATGCTATCCCCGTTCGTTCTTCAGATAAAATACGCTGGGTTATCAATAACGTCGCTGCGGATCAGAAGTTGGATACGTTGGATTTAGGCAGTGGTGTCACGGTGAACCAGTTAGCCTTTGTGAGACAGGGCGATGATCTAGAAATACAGATCTCAGGGCAACCAACCAAAACAGTGATGCTACAGAATTATGTCGCAGACGTTCGCGCGCAGCATCTTCAGTTAAAGCTGGGGGAGACGGTGATGCGTTTACCAGCGGTGGACCCTGTCAGCGGTTACTTTGTTCATTCACAGGATAGTGATGTCGGTATTCTAGGCGATGGGACACATATCTTGCGCAGTGAGGCCCAAGGGGGCAGTAAGCAGACCCTGCACTTATCGGGGCCGGTTGATGGTTATCAACAAGAAGTACAAGGTCTCGATTTGAAGCTATCGAAAGGGGATCAGACGCTCTTTGTTCGAGACTACTACCGCAAACCAGATTCGGTTCGTTTTGCGTGGCAGGGCGGATCCCATGATGAGGTGCTCATTCCAACGGATTATCATCAAGCCGAACAGGCCTTGTTTACCGAACTCAACGTGGAGCGTCGAGACTGGGTGGCCTATATACAGAATGGCGTTACAAGCCGAGAGCAAGTGACGTCATTATTGGCATTAGGGCAAGAGCGCGGCAGTGAGGCGTCAGATTTCTACACCATGCCAGACAGTACCGATTTCACGGTATACGTCAAACATTACCCAGCAAACTATAACGTGGGTGAACGTGCGCTGTTTAGTACGGGCTACCGGGGGCAAAATGGCTTTAAGTTTTACCTCGATGACGAGGGTTACCTTTGCTACAAATCCTACACGCGAGAGGGGTATTATAACGGTGTAGAGCAAACCATCTACACTCAAACGCCTCGAATCAGTGGTCGCCCCGTCAGCCAGCCTAATGACGAGATAGTGATGAAATTTGAGGGGAACCATACCCTGACAGTGATCGCCAAAGGCTGGGGAGGAAAACGGGTCACCACGATTAATTTGAACGATTACGGTTCATCACGAATTCGCTTTGGCTCGACTCGCTATCTAAACGATAGGGTTTACAGTAGTGGCTACAGAGAGCGTCGAATTACTGAAGGGCTAGCGAGCGATCGTCAAATTGATAGTATCTTCCAAACGGATGGTCAAACATGGGGAGCCAGGGATAGCGACGTGGCGACTTATCTTCAGGTTAAAGGCTTCTCAGCGCAGGGCGCGCAAACCCTGATCGATGAAGGCCTAACCAGCCTTGGTCAGTTAGAGCGGGCGGTATCTGTGCTGAAGGCCGCCAATGGGCAGTTGTCTGAATCGTTCATTGCGGCGTATGTGAAATCGGGTCAGAGTTGGCTATACAAAGAGACGAGCATTGCCTATGCCGTCGCCTTGGAAAATATGGGAAGAACCCCACAGTTCATCTTTGATGCGCACCGATATGGCTTATCAGTGCAGAATGTTGAAGCGTATAGCAAGGTAGAAAAACAGTACCCAGGTGAAGACCGCTTAGCTGATTTTGCTCTGGCATTGCGCGGTGATGATAACGCTTTCTTTATGCAAGATCGCGCATCAACGGGAACCCTTACTCACTCTCAAACACAGTTGCTTGATAGCTTACTGACCACGCTTGAAATCCCTGAGTGGGATAGAGAACGGATTCGTCACACCATCGCGGGTAACGCACCGGAAGAGCGTTTTGATGAGCTCGCGCGCCGAATGTACCAAAGCTATCCGACCGAAAGAGAAAAGGAGCTCAATCTATACAATTGGCTGGGCAATGCACTGGGAGGGGATTTTAGGCAGAAGCATACCTTTGAACCGAGTTTACCTAACGATGTGAATCTCTTGACTCAAGCGCTGATCCAAAAAGGATTCTTAGCGAAACGAGCAAGCGAACTGGCGTGGAAACTTTCGGAGTTAGGCGTGTTGGACTACGAGCGTCTTGATAGTCTGATGAAAGTAGGGATTAGTGATAATTCTCAGCTACTTAGGTTGCTGGAAGCGAACGTGAATGGGGAAGATATTCTGGCCGCGAACGCCCACCGGGAGCAATACGAAAGTGGTGCCAGAGGGAATCTCATTCAAGTCTCTACCAGCGGTATATTATCGTCCTACTCCCACTTGGAGCAGACCGTTTATTATGCCAAAGAGTACTTAGGCATTGATGGGGACGGCAACGTCACCCGATTGGGAACCAGTCCTTCAAGTGGTACATTGGCGGGATACGAACAGGTGATCAACCCCGGCCCAGTCTTAGGCTCAGGAGGAGGCTCTCCGACACCCATTCAGATTGCTCAAGGTCAAATTGATGGTGTCGTTAAACTGTGGGAGCAAGGCTATATCGATCGAAGTGTGCGTCAAGTGCCATCGGAACAAAGTTGGTTTGGTCGGAGTAATCCGAGTAATTTGGTGGATGGCTCAGACCGAACGGGAGAGGCGTTTGCTTGGCGGGCGGCGAGCAATAAGGTCGACGACAACATCTATGATGTGGCTATGCAGGCGCCTGATCTGACCAACCCTAACACAGACGATCGTGCAGCGTTTATTCGTTTCGATATGAAGCACAAGGTGGCGTTATCCTCACTGACATTGCGCACGGCTCATGCCGTGGCCGATGCGGATCAACCCGATACCACGCGTTCCGGTAGCTACCGTGTTGAAGCATTGGATGCGAGAGGACAATGGATAA

>NODE_114_length_2745_cov_2.507496

CAAAACGTATTGTTCAAAGCCTTGTCGGTTGGCTTGCGGTGAATCGGGCTTAATGATGTAAAACGTTGCGTTTGGCATAATCATTCCGAATAAAAAAGGGCCTTTCGGCCCTTTAGAATTTGTTCAGAAATTACTCTTCTGTCTCTTGGCCACTGCGGTTCAGTAAGAACTGGACAAGCATTGAGACTGGGCGGCCTGTTGAGCCTTTTGCTGCGCCTGACTTCCACGCAGTACCTGCGATGTCGACGTGTGCCCAGTTGTATTTCTTAGCAAATTTAGACAGGAAGCAACCTGCTGTAATTGTACCGCCAGGGCGACCACCGATGTTTGCCATGTCAGCAAATGGGCTCTTCAGCTGCTCATGGTATTCGTCTGCCATTGGTAGACGCCATGCACGATCGCTTGCTTGTTCAGAAGCGTTCACTAGCTCGTGAGACAGTGGGTTGTGGTTTGAGATAACACCGCTGATGTGGTGGCCCAGTGCAATTACACAAGCGCCTGTTAGCGTTGCTACGTCTACCACACAATCTGGTTCGAAGCGCTCAACATAAGTCAGTGCATCACAAAGAACCAAACGGCCTTCAGCGTCAGTATTAAGTACTTCAACAGTTTGGCCTGACATCGTTGTCAGAATGTCACCTGGACGGTAAGCATTGCTGCCTGGCATGTTTTCACATCCAGCAAGAACACCAATGACGTTGATTGGTAGGTTAAGTTTCGCCAGAGATTTCATGGTACCGAAAACAGATGCCGCACCACACATGTCGTACTTCATCTCATCCATACCTTCACCTGGTTTAAGTGAGATACCGCCTGAATCAAAAGTCAGACCTTTACCAACCAACACGATTGGTTTCGCTTCTGGATCTGGGTTGCCATTGTATTCCATGATAGACATCATAGATTCATTCTTAGAGCCGCGGCCTACTGCAAGGTATGAAGTCATACCCAGTTTTTCCATCTCTTGCTCACCAATAATCTTGGTTGTGATGGTGTCATAGTCATCAGCCAGACGACGAGCCTGAGAAGCAAGGTAAGCAGGGTTTGCGATGTTTGGCGGCATGTTGCCGAGATCTTTCGATGCTTTTACCCCTGAAGCAATGGCAAGACCATGAGTGATCGCTTTTTCACCTAGGTTAAGTTCACGGCGAGTTGGTACGTTGAATACCAGCTTGCGCAGTGGACGGCGAGTCTCTGGCTTGACGCTCTTGAACTGATCAAAAGTGTACAGGCCATCTTTGGTTGCTTCGACTGCTTGACGAACCTTCCAGTAAGTATCGCGGCCTTTAACGTGTAGCTCAGTCAGGAAACATACCGCTTCCATTGAACCTGTTTCATTCAGAGTGTTAATCGTTTTCTGAATAATTTCTTTGTACTGACGCTCGCCCAATTCGCGTTCTTTGCCACAACCGACAAGTAAAACACGTTCAGACAAAACACCTGGTACTTGATGCAGAAGTAGCATCTGCCCTGGTTTACCTTCCAGATCACCACGGCGAAGTAGTGAACTGATATAGCCGTCGCTGATTTTATCGAGCTGTTCAGCAACTGGAGAAAGGCGACGTGGTTCGAATACACCCACAACGATACATGCACTACGTTGCTTTTCCGGGCTACCACTCTTTACACTGAACTCCATGCGTACTCCTACATCCTGAAGACAAATAGAACTAAATGTTAGATAATGGCGTCTTACTTGTTGATTCCTTAATCGGAACTACTTTTAAATAAGCGCGTTAACAGTTAGCCAAAAAATTTAAAAATAAAAGGTTCAACGGGAAATTATAGTGATTCAATTAAAAAAACAAGTTTTGTATAGGTAATTTCAGCGTGATTATTGTTAGATATTTGATCCGCGAGACACTCAAGAGCCAGTTAGCGATATTTTTTATCCTGTTTTTAGTGTTTTTAAGCCAAAAATTGATCAAAGTTTTAGCGGATGCTTCGGATGGAGATATTCCTGCCAGTCTGGTTATGCACTATGTGAGTTTGAGCATGCCGTCAATGGGGCTTTTGATGCTGCCATTGAGTCTTTTCCTCGGTATTTTGCTGACTTTTGGCCGACTGTATGCAGAAAGTGAAATAACTGTCATGAATGCGACCGGAATAGGAAATAAGTTTCTAATCCGCGCCGCACTCTATCTTGCGGTGATCACTGGCGTGATTGCTGCAGCCAATGCATTCGTATTTTCGCCAATGAGTCAGGAAAAGCTGATTCAGTTACAAGAAGAAGTCGAAGCGGAGAACAGTGTTGACCTATTGCAGAAAGGCCAGTTCCAGGGTACTCCGGACGGTTCCTCTGTGGTCTTTATCGACGATATTGAAGGCAAGCAGCTCAAGCATGTGTTTGTAGCTCAGATGCGCCCACGTGATTCAATTCTACCAAGTGTGTCCTTCTCGCAATCTGGTGAGGTGAAAGAACTGAGTGACGGACGTCAGGTTATTCGCATGTACGATGGTACGCGTTATGAAGGTGTGCCAACGCGCGTTGATTACATGGTGACCGATTTTGACCAGTATGAAGGCCTAATTGGCCAGCGTGACGTGAAGCCGAAAGGGCGTGACTGGGAAGCTATCCCGACGATGCAGTTGGTATCTAACCCAGATCCGCGTGCTCAGGCCGAATTGCAATGGCGTATTTCTCTGGTTGTCTGTATTCCGCTTCTGACTATGTTGGTCGTTCCGTTGTCAGCAGTTAACCCACGTCAGGGACGCTTCGCTAAAATGGGCCCAGCGATTCTCATC

>NODE_115_length_2737_cov_2.468421
[truncated: 2,447,630 more chars]
