# Supplementary material for: Disease Diagnostics and Potential Coinfections by Vibrio coralliilyticus During an Ongoing Coral Disease Outbreak in Florida
Source: Front Microbiol. 2020 Oct 26;11:569354. doi: 10.3389/fmicb.2020.569354 (PMC7649382; doi:10.3389/fmicb.2020.569354)
Supplement: Supplementary file 2 [file Data_Sheet_2.zip › S files2/Supplementary File (S9).pdf]

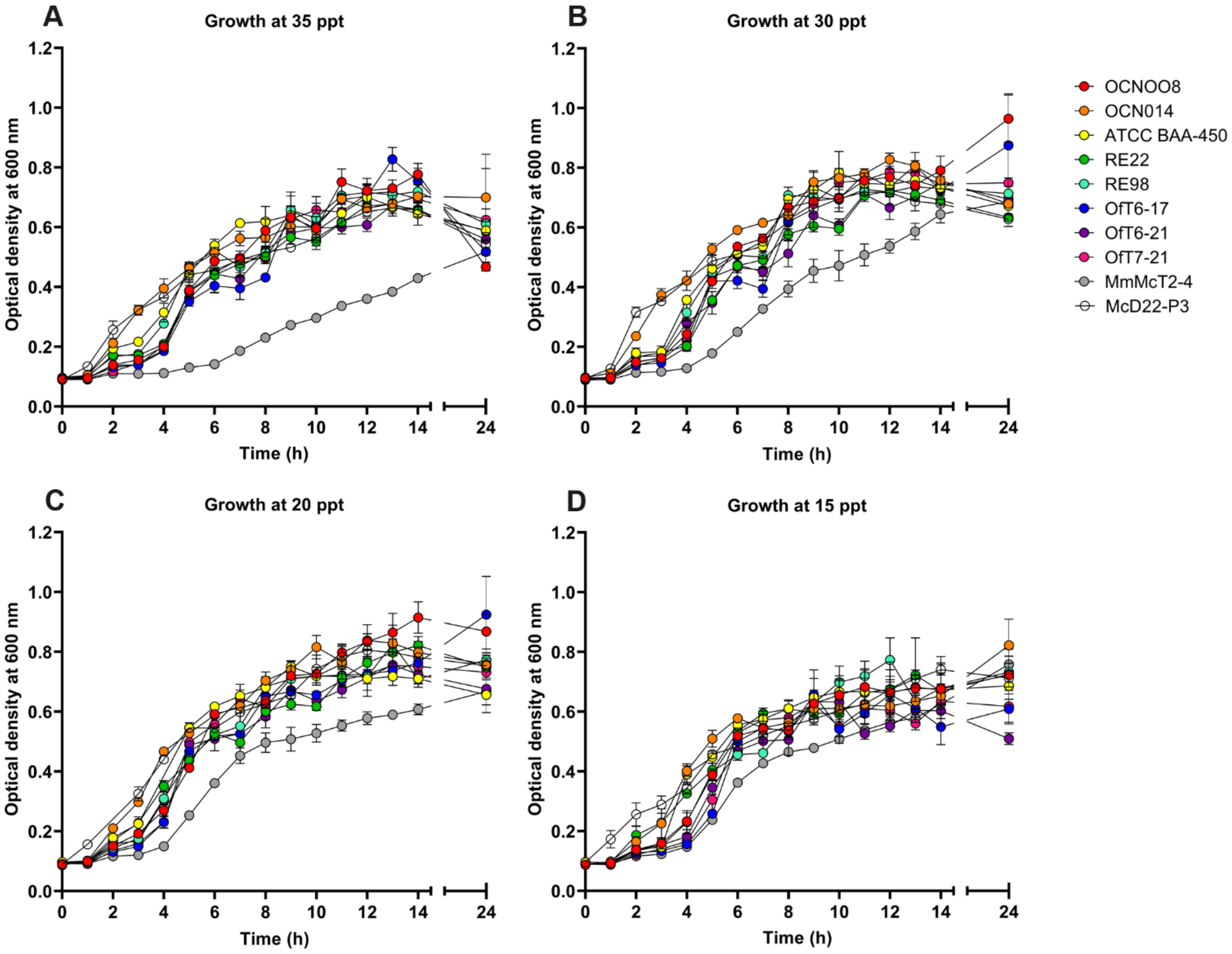

Supplementary File S9. Growth of various *V. coralliilyticus* strains at different salinities. The mean OD_{600} of various strains of *V. coralliilyticus* in SWB adjusted to A) 35 ppt, B) 30 ppt, C) 20 ppt, or 15 ppt over a 14 h period with a final measurement taken at 24 h. A total of 6 replicates were conducted for each strain and condition. The error bars represent the standard error of the mean.
