# Supplementary material for: Disease Diagnostics and Potential Coinfections by Vibrio coralliilyticus During an Ongoing Coral Disease Outbreak in Florida
Source: Front Microbiol. 2020 Oct 26;11:569354. doi: 10.3389/fmicb.2020.569354 (PMC7649382; doi:10.3389/fmicb.2020.569354)
Supplement: Supplementary file 2 [file Data_Sheet_2.zip › S files2/Supplementary File Legends.pdf]

Supplementary Files

Supplementary File S1. List of all diseased corals use this study. All the diseased corals used in this study with accompanying metadata.

Supplementary File S2. DNA oligonucleotides used in this study.

Supplementary File S3. Video demonstration of the VcpA *RapidTest* (mAbDx, Inc.). A time lapse video (depicting a duration of 7 min) of samples run on the VcpA *RapidTest*. The left assay is a sample of seawater and the right assay has 300 ng of recombinant VcpA suspended in seawater added to it. The units on the ruler is cm while the time (min) is depicted in the lower right corner.

Supplementary File S4. Diagram of the improved VcpA *RapidTest*. The diagram illustrates the layout to the newest version of the *RapidTest* with a description of the improvements made on the original design described in (Gharaibeh et al., 2013).

Supplementary File S5. Visual reference card for the VcpA RapidTest and usage protocol. The visual reference card for the *RapidTest* depicting the semi-quantitative reaction to varying amounts of recombinant VcpA. The usage protocols that come with purchased assays are included.

Supplementary File S6. Disease progression results for the diseased corals divided up by general collection location and VcpA test result.

Supplementary File S7. Physical reaction of healthy *M. cavernosa* to bacterial inoculation. A time lapse video depicting apparently healthy *M. cavernosa* inoculated with bacterial cultures (final concentration of 10^8 CFU/ml in 5 L of FSW). A) a fragment inoculated with the non-pathogenic strain MCH1-7 that was isolated from healthy *M. cavernosa*. B) fragment of M. cavernosa inoculated with *V. coralliilyticus* strain OfT6-21. The actual elapsed time is depicted in the lower center of the video.

Supplementary File S8. Physical reaction of healthy M. cavernosa feeding on bacterial cultures mixed with coral feed. A time lapse video depicting apparently healthy M. cavernosa consuming Reef Roids coral feed mixed with A) non-pathogenic bacterium McH1-7 and B) *V. coralliilyticus* OfT6-21. A bacterial pellet was mixed with 50 mg of feed so that the final concentration of bacteria in the 5 L tank was 10^8 CFU/ml. The actual elapsed time is depicted in the lower center of the video.

Supplementary File S9. Growth of various *V. coralliilyticus* strains at different salinities. The mean OD_{600} of various strains of *V. coralliilyticus* in SWB adjusted to A) 35 ppt, B) 30 ppt, C) 20 ppt, or 15 ppt over a 14 h period with a final measurement taken at 24 h. A total of 6 replicates were conducted for each strain and condition. The error bars represent the standard error of the mean.

Supplementary File S10. Growth of various *V. coralliilyticus* at different temperature. The mean OD_{600} of various strains of *V. coralliilyticus* in SWB incubated at A) 23 °C or B) 29 °C. A

total of 6 replicates were conducted for each strain and condition. The error bars represent the standard error of the mean.

Supplementary File S11. The swarming radii of various *V. coralliilyticus* strains at different salinities. The mean swarming radii of various strains over a 4-day period were measured on SWA adjusted to A) 35 ppt, B) 20 ppt, or C) 10 ppt. A total of 3 replicates were conducted for each strain and condition. The error bars represent the standard error of the mean.

Supplementary File S12. The API 20E results for each *V. coralliilyticus* strain.

Supplementary File S13. Average nucleotide identity shared between *V. coralliilyticus* isolates from Florida.

Supplementary File S14. Draft genome of *V. coralliilyticus* strain MmMcT204. The draft genome was too incomplete to be accepted on GenBank, so it is provided here.

Supplementary File S15. Genome metrics of sequenced *V. coralliilyticus* strains from Florida.
